# Supplementary material for: Synthesis of I(III)/S(VI) reagents and their reactivity in photochemical cycloaddition reactions with unsaturated bonds
Source: Nat Commun. 2022 Nov 3;13:6588. doi: 10.1038/s41467-022-34401-7 (PMC9633813; doi:10.1038/s41467-022-34401-7)
Supplement: Supplementary file 1 — Supplementary Information [file 41467_2022_34401_MOESM1_ESM.pdf]

Supplementary Information

Synthesis of $I^{(III)}/S^{(VI)}$ Reagents and Their Reactivity in Photochemical Cycloaddition Reactions with Unsaturated Bonds

Li Li,[1] Kun Deng,[2] Yajie, Xing[1], Cheng Ma[3], Shaofei Ni[3], Zhaofeng Wang,[2]* and Yong Huang[1]*

[1]Department of Chemistry, The Hong Kong University of Science and Technology, Clear Water Bay, Kowloon, Hong Kong SAR, PR China

[2]State Key Laboratory of Chemo/Biosensing and Chemometrics, College of Chemistry and Chemical Engineering, Hunan University, Changsha, Hunan 410082, PR China

[3]Department of Chemistry and Key Laboratory for Preparation and Application of Ordered Structural Materials of Guangdong Province, Shantou University, Shantou 515063, Guangdong, PR China

correspondence to: zfwangchem@hnu.edu.cn; yonghuang@ust.hk

Table of Contents

1. Supplementary Notes. S2
2. Supplementary Methods. S3
   2.1 Synthesis of I(III)/S(VI) Reagents S3
   2.2 Light-mediated Tandem [4+2] Cycloaddition: reaction scope. S20
   2.3 Light-mediated Tandem [3+2] Cycloaddition: reaction scope. S41
   2.4 Light-mediated and/or Rh-mediated cycloaddition with nitriles, alkynes and allene: reaction scope. S52
   2.5 Mechanistic experiments. S62
   2.6 Synthetic Application of [4+2] and [3+2] Products. S65
   2.7 Computational studies and computational details. S69
3. Supplementary Figures. S74
   3.1 NMR spectra. S74
4. Supplementary References. S220

1. Supplementary Notes.

Chemicals were purchased from commercial suppliers and used without further purification unless otherwise stated. Anhydrous solvents were dried by passing through an activated alumina column on a PureSolv™ solvent purification system (Innovative Technologies, Inc., MA). Analytical thin-layer chromatography (TLC) was performed using Huanghai silica gel plates with HSGF 254. Visualization of the developed chromatogram was performed by irradiation with UV light or treatment with a solution of appropriate stains. Flash column chromatography was performed on silica gel (Qindao Puke Co., China., 200-300 mesh) or neutral silica gel (Bio-Gene Tech. Ltd., 230-400 mesh). Organic solutions were concentrated under reduced pressure on an EYELA rotatory evaporator. Unless otherwise stated, reactions were carried out under an argon atmosphere. Yields refer to purified compounds unless otherwise noted. NMR spectra were recorded at 298 K (unless otherwise stated) on a Bruker AV 400 MHz NMR spectrometer. Chemical shifts (δ) are quoted in ppm relative to residual solvent signals, $CDCl_3$ referenced at δ 7.26 and 77.16 ppm, DMSO-d_6 referenced at δ 2.50 and 39.52 ppm, CD_3CN referenced at δ 1.94 and 1.39, 118.69 ppm, Acetone-d_6 referenced at δ 2.05 and 29.92, 206.68 ppm. Coupling constants (*J*) are quoted in hertz (Hz). Multiplicity is reported with the following abbreviations: s = singlet, brs = broad singlet, d = doublet, t = triplet, q = quartet, p = quintet, dt = doublet of triplets, td = triplet of doublets, tt = triplet of triplets, sp = septet, m = multiplet, app = apparent. Mass spectra were collected on an Agilent GC/MS 5975C system, a MALDI Micro MX mass spectrometer, or an API QSTAR XL System.

2. Supplementary Methods.

2.1 Synthesis of I(III)/S(VI) Reagents

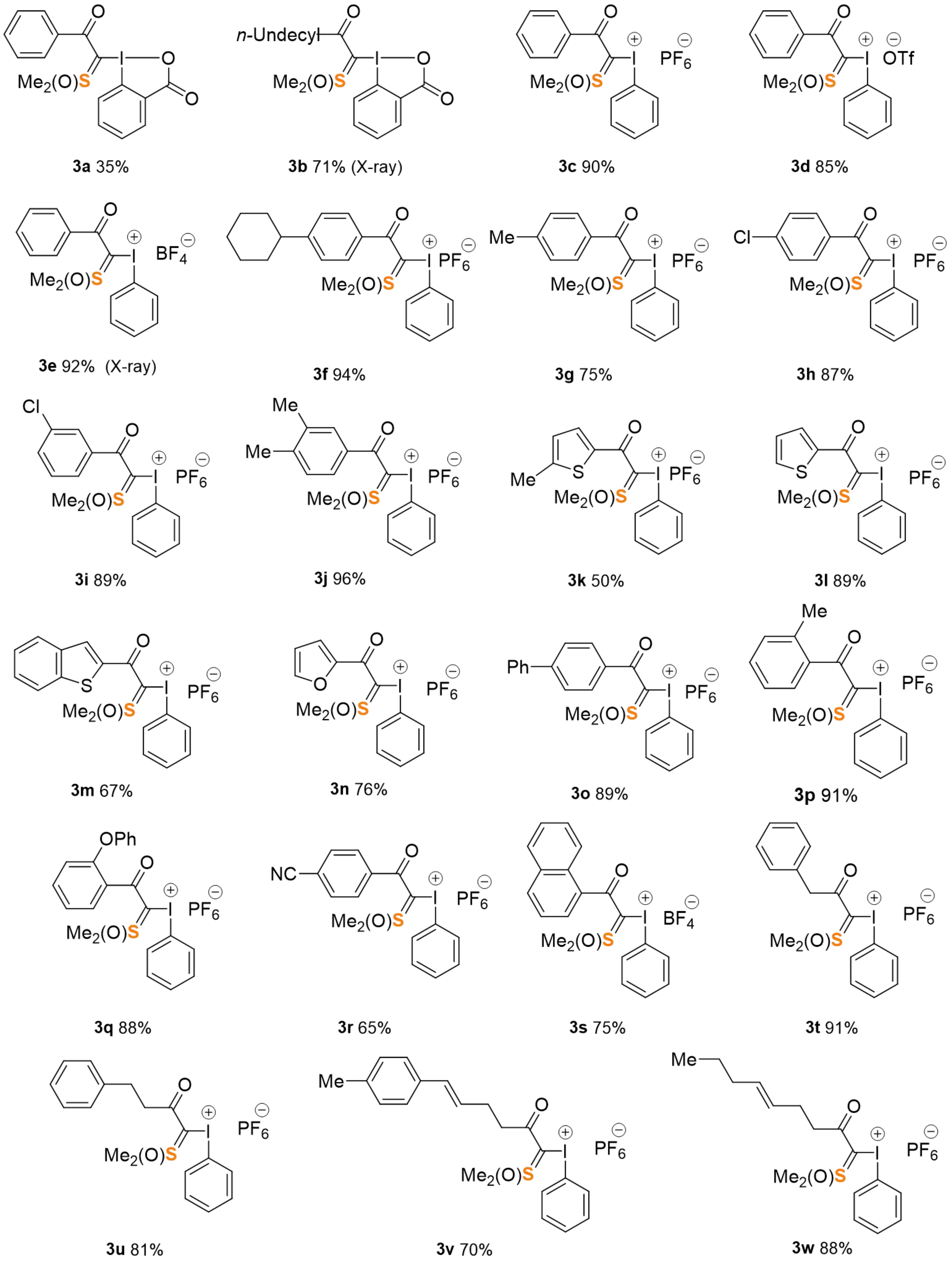

Supplementary Figure 1. Cyclic and acyclic hypervalent iodine reagents **3a-3w**

General procedure for the synthesis of ylides **2**

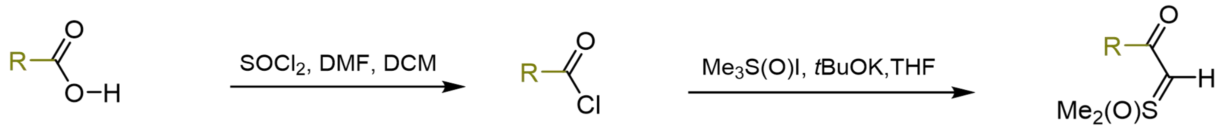

2

According to the literature,[1] the slightly modified method is as follows. Under N_2, acid (5.0 mmol) in CH_2Cl_2 (10 mL) at 0 °C before adding $SOCl_2$ (7.5 mmol, 1.5 equiv) and one drop of DMF. After 60 minutes stirring at 50 °C, and the volatiles were carefully evaporated under high-vacuum. During that time, trimethylsulfoxonium iodide (3.3 g, 16.5 mmol, 3.0 equiv) was suspended under N_2 in dry THF (40 mL) in a flame-dried 100 mL round bottom flask that was protected from light with aluminium foil. Potassium tertbutoxide (1.8 g, 16.5 mmol, 3.3 equiv) was added, and the mixture was stirred at reflux for 3 hours. After cooling to 0 °C, a solution of acid chloride obtained above in THF (10 mL) was added dropwise to the mixture. The mixture was stirred at room temperature for another hour and then solvents were removed under vacuum. Then 80 ml water were added, extraction with CH_2Cl_2 (50 × 3 mL). Purification by flash chromatography (dichloromethane/MeOH = 30/1) provided the ylide.

General procedure A for the synthesis of cyclic I, S-ylides **3**

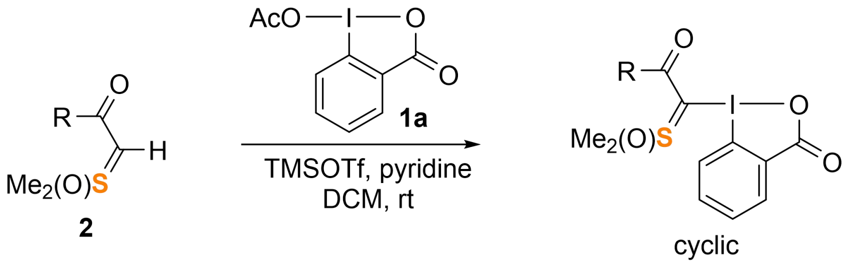

cyclic
3a and **3b**

A solution of 1-acetoxy-1,2-benziodoxol-3(*1H*)-one (1.53 g, 5.0 mmol, 1.0 equiv.) in dichloromethane (10.0 mL, 0.5 M) was treated with trimethylsilyl trifluoromethanesulfonate (0.9 mL, 5.0 mmol, 1.0 equiv.) at room temperature. After 10 minutes, a solution of pyridine (0.44 mL, 5.5 mmol, 1.1 equiv.) in dichloromethane (2.0 mL) was added dropwise over 10 minutes and the resulted suspension was stirred for 30 min at room temperature. A solution of the corresponding sulfoxonium ylides (5.0 mmol, 1.0 equiv.) in dichloromethane (2.0 mL) was added dropwise over 10 minutes and the resulting reaction mixture was stirred until TLC indicated all the ylide was consumed (usually 8 hours). The reaction solution was washed

with distilled water (200 mL × 2) and dried with anhydrous sodium sulfate. The solvent was removed under *vacuum* and the residue was purified by flash column chromatography (DCM/acetone = 10:1) to provide **3** as white solid.

General procedure B for the synthesis of acyclic I, S-ylide **3**

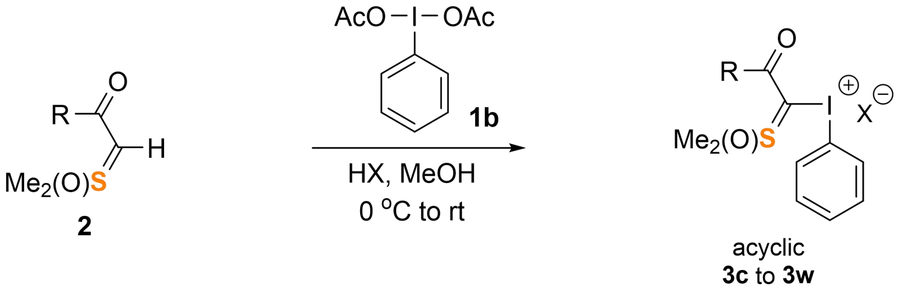

A solution of aryliodoso diacetate (5.0 mmol, 1.0 equiv.) in MeOH (5.0 mL, 1.0 M) was treated with corresponding acid HX (5.0 mmol, 1.0 equiv) at room temperature. This clear solution was added dropwise to the ice bath-cooled solution of sulfoxonium ylides (5.0 mmol, 1.0 equiv.) in MeOH (5.0 mL, 1.0 M) over 10 min with stirring. The resulting reaction mixture was stirred at 0 °C for 1 hour. During this period, a large amount of a white precipitate was formed. The solid was collected by filtration, washed successively with MeOH (5 mL × 3) and Et_2O (5 ml × 3), dried under high *vacuum* and stored at -20 °C. If the hypervalent iodine reagent failed to precipitate, it was subjected to flash column chromatography, eluting with DCM/Acetone mixtures.

1-(1-(dimethyl(oxo)-λ⁶-sulfaneylidene)-2-oxo-2-phenylethyl)-1,2-benziodoxol-3(1*H*)-one (3a)

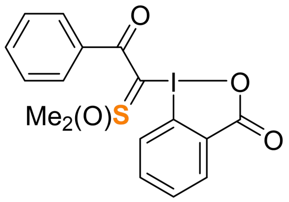

Prepared according to the general procedure A using 2-(dimethyl(oxo)-λ^6-sulfaneylidene)-1-phenylethan-1-one (981.3 mg, 5.0 mmol). Purification by flash chromatography (dichloromethane/acetone = 4/1) provided **3a** as a white solid (773.9 mg, 35% yield).

^{1}H NMR (400 MHz, DMSO-d_6) δ 8.12 (d, J = 8.1 Hz, 1H), 8.04 (dd, J = 7.4, 1.7 Hz, 1H), 7.82 (ddd, J = 8.3, 7.1, 1.7 Hz, 1H), 7.69 (t, J = 7.3 Hz, 1H), 7.43 – 7.35 (m, 3H), 7.31 (dd, J = 8.5, 6.4 Hz, 2H), 4.03 (s, 3H), 3.76 (s, 3H).

^{13}C NMR (101 MHz, DMSO-d_6) δ 188.97, 166.51, 140.48, 134.76, 134.27, 131.69, 130.96, 130.70, 128.47, 127.22, 126.29, 118.76, 66.08, 42.42, 42.37.

HRMS (ESI) calculated for $C_{17}H_{16}IO_4S^+$ $[M+H]^+$ m/z: 442.9808, found: 442.9810.

1-(1-(dimethyl(oxo)-λ⁶-sulfaneylidene)-2-oxododecyl)-1,2-benziodoxol-3(1H)-one (3b)

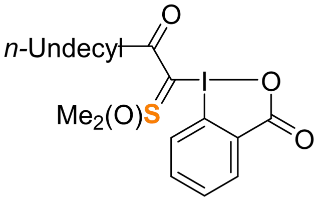

Prepared according to the general procedure A using 1-(dimethyl(oxo)-λ^6-sulfaneylidene)tridecan-2-one (823.4 mg, 3.0 mmol). Purification by flash chromatography (dichloromethane/acetone = 4/1) provided **3b** as a white solid (1.1 g, 71% yield).

^{1}H NMR (400 MHz, Acetone-d_6) δ 8.21 (dd, J = 7.3, 1.7 Hz, 1H), 7.93 (dd, J = 8.1, 1.0 Hz, 1H), 7.73 (td, J = 8.1, 7.6, 1.7 Hz, 1H), 7.66 (td, J = 7.3, 1.0 Hz, 1H), 3.94 (s, 3H), 3.79 (s, 3H), 2.66 (dt, J = 15.0, 7.4 Hz, 1H), 2.48 (dt, J = 15.1, 7.4 Hz, 1H), 1.53 (p, J = 7.4, 6.8 Hz, 2H), 1.32 – 1.15 (m, 16H), 0.86 (t, J = 6.8 Hz, 3H);

^{13}C NMR (101 MHz, Acetone-d_6) δ 193.55, 167.48, 136.03, 134.18, 132.69, 131.02, 125.85, 119.02, 64.00, 43.70, 43.10, 40.13, 32.69, 30.41, 29.97, 26.31, 23.39, 14.44.

HRMS (ESI) calculated for $C_{22}H_{34}IO_4S^+$ $[M+H]^+$ m/z: 521.1217, found:521.1213.

The crystal structure of **3b** has been deposited at the Cambridge Crystallographic Data Centre, CCDC 2068912.

(1-(dimethyl(oxo)-λ^6-sulfaneylidene)-2-oxo-2-phenylethyl)(phenyl)iodonium hexafluorophosphate (3c)

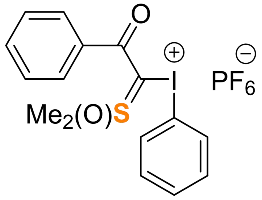

Prepared according to the general procedure B using phenyliodoso diacetate (1.6 g, 5.0 mmol), HPF_6 (0.68 ml, 65% w/w, 5.0 mmol) and 2-(dimethyl(oxo)-λ^6-sulfaneylidene)-1-phenylethan-1-one (981.3 mg, 5.0 mmol). After filtration, **3c** was collected as a white solid (2.5 g, 90% yield).

^{1}H NMR (400 MHz, CD_3CN) δ 7.79 (d, J = 8.5 Hz, 2H), 7.75 – 7.71 (m, 1H), 7.63 – 7.55 (m, 3H), 7.55 – 7.44 (m, 4H), 3.73 (s, 6H).

^{13}C NMR (101 MHz, CD_3CN) δ 188.84, 138.55, 133.02, 132.57, 132.19, 131.44, 128.59, 127.41, 118.18, 60.83, 42.54.

^{19}F NMR (376 MHz, CD_3CN) δ -72.40 (d, J = 706 Hz).

^{31}P NMR (162 MHz, CD_3CN) δ -144.54 (h, J = 706 Hz).

HRMS (ESI) calculated for $C_{16}H_{16}IO_2S^+$ $[M-PF_6]^+$ m/z: 398.9910, found: 398.9917.

(1-(dimethyl(oxo)-λ^6-sulfanylidene)-2-oxo-2-phenylethyl)(phenyl)-λ^3-iodanyl trifluoromethanesulfon-ate (3d)

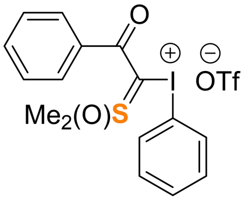

Prepared according to the general procedure B using phenyliodoso diacetate (1.6 g, 5.0 mmol), HOTf (0.44 ml, 5.0 mmol) and 2-(dimethyl(oxo)-λ^6-sulfaneylidene)-1-phenylethan-1-one (981.3 mg, 5.0 mmol). After filtration, **3d** was collected as a white solid (2.3 g, 85% yield).

^{1}H NMR (400 MHz, CD_3CN) δ 7.78 (d, J = 8.1 Hz, 2H), 7.73 – 7.67 (m, 1H), 7.61 – 7.52 (m, 3H), 7.52 – 7.44 (m, 4H), 3.74 (s, 6H).

^{13}C NMR (101 MHz, CD_3CN) δ 188.87, 138.77, 132.96, 132.33, 132.02, 131.29, 128.51, 127.41, 120.90 (q, J = 316.6 Hz, $OSO_2\mathit{C}F_3$), 117.42, 61.47, 42.44.

^{19}F NMR (376 MHz, CD_3CN) δ -79.12.

HRMS (ESI) calculated for $C_{16}H_{16}IO_2S^+$ [M-OTf]$^+$ m/z: 398.9910, found: 398.9917.

(1-(dimethyl(oxo)-λ^6-sulfaneylidene)-2-oxo-2-phenylethyl)(phenyl)iodonium tetrafluoroborate (3e)

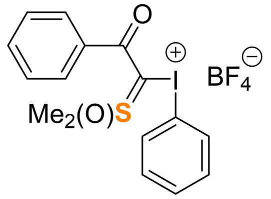

Prepared according to the general procedure B using phenyliodoso diacetate (1.6 g, 5.0 mmol), HBF_4 (0.66 ml, 48% w/w, 5.0 mmol) and 2-(dimethyl(oxo)-λ^6-sulfaneylidene)-1-phenylethan-1-one (981.3 mg, 5.0 mmol). After filtration, **3e** was collected as a white solid (2.2 g, 92% yield).

^{1}H NMR (400 MHz, CD_3CN) δ 7.80 – 7.75 (m, 2H), 7.72 (tt, J = 6.9, 1.1 Hz, 1H), 7.62 – 7.53 (m, 3H), 7.53 – 7.43 (m, 4H), 3.74 (s, 6H).

^{13}C NMR (101 MHz, CD_3CN) δ 188.90, 138.66, 132.93, 132.48, 132.15, 131.37, 128.57, 127.40, 118.14, 61.00, 42.44.

^{19}F NMR (376 MHz, CD_3CN) δ -150.08 (d, J = 18.8 Hz).

HRMS (ESI) calculated for $C_{16}H_{16}IO_2S^+$ [M-BF_4]$^+$ m/z: 398.9910, found: 398.9919.

The crystal structure of **3c** has been deposited at the Cambridge Crystallographic Data Centre, CCDC 2068911.

(1-(dimethyl(oxo)-λ^6-sulfaneylidene)-2-oxo-2-(4-cyclohexylphenyl)ethyl)(phenyl)iodonium hexafluorophosphate (3f)

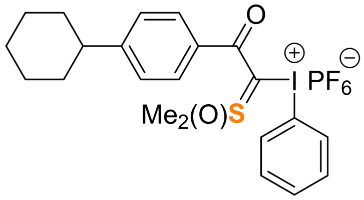

Prepared according to the general procedure B using phenyliodoso diacetate (966.0 mg, 3.0 mmol), HPF_6 (0.41 ml, 65% w/w, 3.0 mmol) and 1-(4-cyclohexylphenyl)-2-(dimethyl(oxo)-λ^6-sulfanylidene)ethan-1-one

(834.4 g, 3.0 mmol). After flash column chromatography, eluting with DCM/Acetone mixtures, **3f** was collected as a white solid (1.77 g, 94% yield).

^{1}H NMR (400 MHz, Acetone-*d_6*) δ 7.96 – 7.90 (m, 2H), 7.74 (tt, *J* = 6.6, 1.1 Hz, 1H), 7.66 – 7.60 (m, 2H) 7.52 – 7.45 (m, 2H), 7.38 – 7.32 (m, 2H), 3.91 (s, 6H), 2.65 – 2.59 (m, 1H), 1.89 – 1.82 (m, 4H), 1.79 – 1.71 (m, 1H), 1.54 – 1.38 (m, 4H), 1.35 – 1.27 (m, 1H).

^{13}C NMR (101 MHz, Acetone-*d_6*) δ 188.76, 151.80, 136.30, 132.61, 132.34, 132.22, 127.86, 126.81, 118.65, 61.45, 44.34, 42.32, 34.08, 26.58, 25.83.

^{19}F NMR (376 MHz, Acetone-*d_6*) δ -71.28 (d, *J* = 709 Hz).

^{31}P NMR (162 MHz, Acetone-*d_6*) δ -144.12 (h, *J* = 709 Hz).

HRMS (ESI) calculated for $C_{22}H_{26}IO_2S^+$ $[M-PF_6]^+$ m/z: 481.0693, found: 481.0691.

(1-(dimethyl(oxo)-λ^6-sulfaneylidene)-2-oxo-2-(*p*-tolyl)ethyl)(phenyl)iodonium hexafluorophosphate (3g)

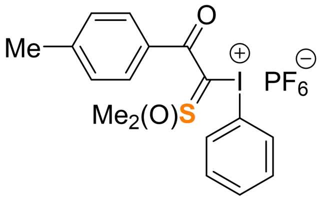

Prepared according to the general procedure B using phenyliodoso diacetate (966.0 mg, 3.0 mmol), HPF_6 (0.41 ml, 65% w/w, 3.0 mmol) and 2-(dimethyl(oxo)-λ^6-sulfanylidene)-1-(p-tolyl)ethan-1-one (630.0 mg, 3.0 mmol). After filtration, **3g** was collected as a white solid (1.3 g, 75% yield).

^{1}H NMR (400 MHz, Acetone-*d_6*) δ 8.03 – 7.92 (m, 2H), 7.76 (tt, *J* = 6.7, 1.1 Hz, 1H), 7.71 – 7.60 (m, 2H), 7.51 – 7.41 (m, 2H), 7.32 (d, *J* = 7.7 Hz, 2H), 3.94 (s, 6H), 2.42 (s, 3H).

^{13}C NMR (101 MHz, Acetone-*d_6*) δ 188.76, 141.88, 135.97, 132.66, 132.33, 132.18, 128.96, 127.74, 118.72, 61.33, 42.30, 20.60.

^{19}F NMR (376 MHz, Acetone-*d_6*) δ -71.80 (d, *J* = 708 Hz).

^{31}P NMR (162 MHz, Acetone-*d_6*) δ -144.19 (h, *J* = 709 Hz).

HRMS (ESI) calculated for $C_{17}H_{18}IO_2S^+$ $[M-PF_6]^+$ m/z: 413.0067, found: 413.0070.

(1-(dimethyl(oxo)-λ^6-sulfaneylidene)-2-oxo-2-(4-chloorophenyl)ethyl)(phenyl)iodonium hexafluoro-ophosphate (3h)

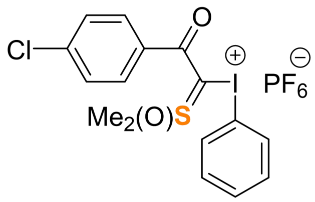

Prepared according to the general procedure B using phenyliodoso diacetate (966.0 mg, 3.0 mmol), HPF_6 (0.41 ml, 65% w/w, 3.0 mmol) and 1-(4-chlorophenyl)-2-(dimethyl(oxo)-λ^6-sulfanylidene)ethan-1-one (690.0 mg, 3.0 mmol). After flash column chromatography, eluting with DCM/Acetone mixtures, **3h** was collected as a light yellow solid (1.5 g, 87% yield).

^{1}H NMR (400 MHz, Acetone-d_6) δ 8.01 (d, J = 7.8 Hz, 2H), 7.77 (t, J = 7.4 Hz, 1H), 7.65 (t, J = 7.8 Hz, 2H), 7.58 – 7.53 (m, 4H), 3.96 (s, 6H).

^{13}C NMR (101 MHz, Acetone-d_6) δ 187.54, 137.63, 136.67, 132.82, 132.42, 132.25, 129.36, 128.64, 118.67, 61.87, 42.18.

^{19}F NMR (376 MHz, Acetone-d_6) δ -71.80 (d, J = 708 Hz).

^{31}P NMR (162 MHz, Acetone-d_6) δ -144.20 (h, J = 709 Hz).

HRMS (ESI) calculated for $C_{16}H_{15}IClO_2S^+$ $[M-PF_6]^+$ m/z: 432.9520, found: 432.9522.

(1-(dimethyl(oxo)-λ^6-sulfaneylidene)-2-oxo-2-(3-chlorophenyl)ethyl)(phenyl)iodonium hexafluorophosphate (3i)

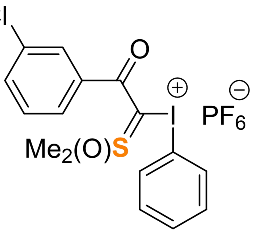

Prepared according to the general procedure B using phenyliodoso diacetate (966.0 mg, 3.0 mmol), HPF_6 (0.41 ml, 65% w/w, 3.0 mmol) and 1-(3-chlorophenyl)-2-(dimethyl(oxo)-λ^6-sulfanylidene)ethan-1-one (690.1 mg, 3.0 mmol). After filtration, **3i** was collected as a white solid (1.59 g, 89% yield).

^{1}H NMR (400 MHz, Acetone-d_6) δ 7.99 (d, J = 8.0 Hz, 2H), 7.82 – 7.75 (m, 1H), 7.66 (t, J = 7.8 Hz, 2H), 7.63 – 7.59 (m, 1H), 7.58 – 7.54 (m, 1H), 7.52 – 7.45 (m, 2H), 3.98 (s, 6H).

^{13}C NMR (101 MHz, Acetone-d_6) δ 187.13, 140.97, 133.76, 132.97, 132.45, 132.22, 131.08, 130.50, 127.39, 125.92, 118.74, 62.13, 42.15.

^{19}F NMR (376 MHz, Acetone-d_6) δ -71.89 (d, J = 708 Hz).

^{31}P NMR (162 MHz, Acetone-d_6) δ -144.21 (h, J = 708 Hz).

HRMS (ESI) calculated for $C_{16}H_{15}IClO_2S^+$ $[M\text{-}PF_6]^+$ m/z: 432.9520, found: 432.9521.

(1-(dimethyl(oxo)-λ^6-sulfaneylidene)-2-oxo-2-(3,4-dimethylphenyl)ethyl)(phenyl)iodonium hexafluorophosphate (3j)

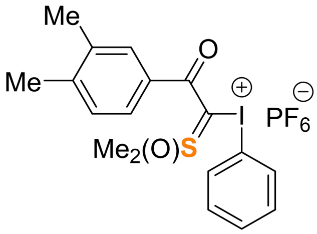

Prepared according to the general procedure B using phenyliodoso diacetate (966.0 mg, 3.0 mmol), HPF_6 (0.41 ml, 65% w/w, 3.0 mmol) and 2-(dimethyl(oxo)-λ^6-sulfanylidene)-1-(3,4-dimethylphenyl)ethan-1-one (720.4 mg, 3.0 mmol). After flash column chromatography, eluting with DCM/Acetone mixtures, **3j** was collected as a white solid (1.69 g, 96% yield).

^{1}H NMR (400 MHz, Acetone-d_6) δ 8.00 – 7.94 (m, 2H), 7.80 – 7.74 (m, 1H), 7.68 – 7.63 (m, 2H), 7.30 – 7.21 (m, 3H), 3.95 (s, 6H), 2.33 (s, 3H), 2.23 (s, 3H).

^{13}C NMR (101 MHz, Acetone-d_6) δ 189.05, 140.54, 136.66, 136.42, 132.66, 132.30, 132.15, 129.53, 128.73, 125.19, 118.90, 61.36, 42.31, 18.97, 18.85.

^{19}F NMR (376 MHz, Acetone-d_6) δ -71.77 (d, J = 708 Hz).

^{31}P NMR (162 MHz, Acetone-d_6) δ -144.19 (h, J = 709 Hz).

HRMS (ESI) calculated for $C_{18}H_{20}IO_2S^+$ $[M\text{-}PF_6]^+$ m/z: 427.0223, found: 427.0232.

(1-(dimethyl(oxo)-λ^6-sulfaneylidene)-2-oxo-2-(5-methylthiophen)ethyl)(phenyl)iodonium hexafluorophosphate (3k)

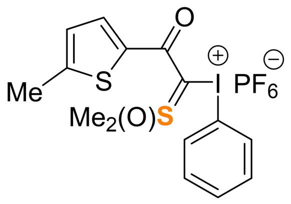

Prepared according to the general procedure B using phenyliodoso diacetate (966.0 mg, 3.0 mmol), HPF_6 (0.41 ml, 65% w/w, 3.0 mmol) and 2-(dimethyl(oxo)-λ^6-sulfanylidene)-1-(5-methylthiophen-2-yl)ethan-1-one (648.1 mg, 3.0 mmol). After flash column chromatography, eluting with DCM/Acetone mixtures, **3k** was collected as a white solid (869.9 mg, 50% yield).

^{1}H NMR (400 MHz, Acetone-*d*$_6$) δ 8.13 – 8.08 (m, 2H), 7.77 – 7.70 (m, 1H), 7.68 – 7.62 (m, 3H), 6.90 (dd, *J* = 3.8, 1.2 Hz, 1H), 3.92 (s, 6H), 2.53 (s, 3H).

^{13}C NMR (101 MHz, Acetone-*d*$_6$) δ 178.49, 147.57, 138.61, 132.28, 132.25, 132.18, 132.12, 126.65, 118.60, 58.42, 42.90, 14.66.

^{19}F NMR (376 MHz, Acetone-*d*$_6$) δ -71.55 (d, *J* = 708 Hz).

^{31}P NMR (162 MHz, Acetone-*d*$_6$) δ -144.17 (h, *J* = 709 Hz).

HRMS (ESI) calculated for $C_{15}H_{16}IO_2S^+$ $[M-PF_6]^+$ m/z: 418.9631, found: 418.9632.

(1-(dimethyl(oxo)-λ^6-sulfaneylidene)-2-oxo-2-(thiophen-2-yl)ethyl)(phenyl)iodonium hexafluorophosphate (3l)

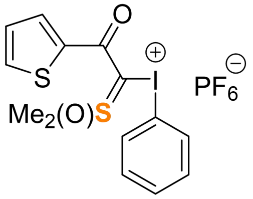

Prepared according to the general procedure B using phenyliodoso diacetate (1.6 g, 5.0 mmol), HPF_6 (0.68 ml, 65% w/w, 5.0 mmol) and 2-(dimethyl(oxo)-λ^6-sulfanylidene)-1-(thiophen-2-yl)ethan-1-one (1.0 g, 5.0 mmol). After filtration, **3l** was collected as a white solid (2.50 g, 89% yield).

^{1}H NMR (400 MHz, Acetone-*d*$_6$) δ 8.15 – 8.10 (m, 2H), 7.85 (dd, *J* = 5.0, 1.1 Hz, 1H), 7.81 (dd, *J* = 3.8, 1.1 Hz, 1H), 7.78 – 7.73 (m, 1H), 7.69 – 7.63 (m, 2H), 7.22 (dd, *J* = 5.0, 3.8 Hz, 1H), 3.96 (s, 6H).

^{13}C NMR (101 MHz, Acetone-*d*$_6$) δ 178.88, 141.05, 132.29, 132.26, 132.10, 131.50, 127.86, 118.61, 59.29, 42.77.

^{19}F NMR (376 MHz, Acetone-*d*$_6$) δ -71.96 (d, *J* = 707 Hz).

^{31}P NMR (162 MHz, Acetone-*d*$_6$) δ -144.21(h, *J* = 708 Hz).

HRMS (ESI) calculated for $C_{14}H_{14}IO_2S_2^+$ $[M-PF_6]^+$ m/z: 404.9474, found: 404.9481.

(1-(dimethyl(oxo)-λ^6-sulfaneylidene)-2-oxo-2-(benzo[b]thiophen-2-yl)ethyl)(phenyl)iodonium hexafluorophosphate (3m)

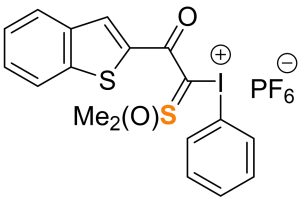

Prepared according to the general procedure B using phenyliodoso diacetate (966.0 mg, 3.0 mmol), HPF_6 (0.41 ml, 65% w/w, 3.0 mmol) and 1-(benzo[b]thiophen-2-yl)-2-(dimethyl(oxo)-l6-sulfanylidene)ethan-1-one (756.1 mg, 3.0 mmol). After flash column chromatography, eluting with DCM/Acetone mixtures, **3m** was collected as a light yellow solid (1.24 g, 67% yield).

^{1}H NMR (400 MHz, Acetone-d_6) δ 8.18 – 8.15 (m, 2H), 8.05 (s, 1H), 8.03 (d, J = 7.4 Hz, 1H),7.96 – 7.94 (m, 1H), 7.82 – 7.75 (m, 1H), 7.69 – 7.65 (m, 2H), 7.57 – 7.51 (m, 1H), 7.49 (td, J = 7.5, 1.3 Hz, 1H), 3.98 (s, 6H).

^{13}C NMR (101 MHz, Acetone-d_6) δ 179.75, 141.03, 140.72, 138.83, 132.60, 132.40, 132.31, 128.22, 127.09, 125.65, 125.30, 122.62, 118.94, 60.77, 42.59.

^{19}F NMR (376 MHz, Acetone-d_6) δ -71.88 (d, J = 708 Hz).

^{31}P NMR (162 MHz, Acetone-d_6) δ -144.18 (h, J = 709 Hz).

HRMS (ESI) calculated for $C_{18}H_{16}IO_2S_2^+$ $[M-PF_6]^+$ m/z: 454.9631, found: 454.9635.

(1-(dimethyl(oxo)-λ^6-sulfaneylidene)-2-oxo-2-(furan-2-yl)ethyl)(phenyl)iodonium hexafluorophosphate (3n)

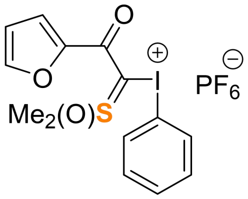

Prepared according to the general procedure B using phenyliodoso diacetate (966.0 mg, 3.0 mmol), HPF_6 (0.41 ml, 65% w/w, 3.0 mmol) and 2-(dimethyl(oxo)-λ^6-sulfanylidene)-1-(furan-2-yl)ethan-1-one (558.1 mg, 3.0 mmol). After filtration, **3n** was collected as a white solid (1.3 g, 76% yield).

^{1}H NMR (400 MHz, Acetone-d_6) δ 8.26 – 8.18 (m, 2H), 7.96 – 7.93 (m, 1H), 7.74 (tt, J = 6.7, 1.2 Hz, 1H), 7.65 – 7.60 (m, 2H), 7.28 (d, J = 3.6 Hz, 1H), 6.73 (dd, J = 3.6, 1.8 Hz, 1H), 3.93 (s, 6H).

^{13}**C NMR** (101 MHz, Acetone-d_6) δ 172.88, 151.24, 145.88, 133.17, 132.22, 131.93, 119.01, 116.69, 112.55, 57.07, 42.81.

^{19}**F NMR** (376 MHz, Acetone-d_6) δ -72.07 (d, J = 707 Hz).

^{31}**P NMR** (162 MHz, Acetone-d_6) δ -144.22 (h, J = 708 Hz).

HRMS (ESI) calculated for $C_{14}H_{14}IO_3S^+$ $[M-PF_6]^+$ m/z: 388.9703, found: 388.9704.

(1-(dimethyl(oxo)-λ^6-sulfaneylidene)-2-oxo-2-([1,1'-biphenyl]-4-yl)ethyl)(phenyl)iodonium hexafluorophosphate (3o)

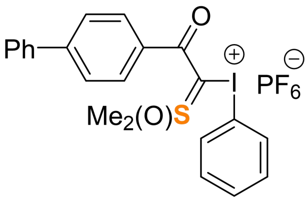

Prepared according to the general procedure B using phenyliodoso diacetate (644.0 mg, 2.0 mmol), HPF_6 (0.28 ml, 65% w/w, 2.0 mmol) and 1-([1,1'-biphenyl]-4-yl)-2-(dimethyl(oxo)-λ^6-sulfanylidene)ethan-1-one (544.2 mg, 2.0 mmol). After filtration, **3o** was collected as a white solid (1.1 g, 89% yield).

^{1}**H NMR** (400 MHz, Acetone-d_6) δ 8.11 – 8.00 (m, 2H), 7.85 – 7.81 (m, 2H), 7.81 – 7.74 (m, 3H), 7.73 – 7.63 (m, 4H), 7.57 – 7.49 (m, 2H), 7.49 – 7.39 (m, 1H), 4.00 (s, 6H).

^{13}**C NMR** (101 MHz, Acetone-d_6) δ 188.40, 143.84, 139.58, 137.60, 132.88, 132.39, 132.21, 129.04, 128.38, 128.18, 127.06, 126.76, 118.86, 61.62, 42.31.

^{19}**F NMR** (376 MHz, Acetone-d_6) δ -72.14 (d, J = 707 Hz).

^{31}**P NMR** (162 MHz, Acetone-d_6) δ -144.22 (h, J = 709 Hz).

HRMS (ESI) calculated for $C_{22}H_{20}IO_2S^+$ $[M-PF_6]^+$ m/z: 475.0223, found: 475.0227.

(1-(dimethyl(oxo)-λ^6-sulfaneylidene)-2-oxo-2-(*o*-tolyl)ethyl)(phenyl)iodonium hexafluorophosphate (3p)

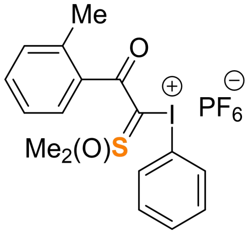

Prepared according to the general procedure B using phenyliodoso diacetate (966.0 mg, 3.0 mmol), HPF_6 (0.41 ml, 65% w/w, 3.0 mmol) and 1-([1,1'-biphenyl]-2-yl)-2-(dimethyl(oxo)-λ^6-sulfanylidene)ethan-1-one (630.2 mg, 3.0 mmol). After flash column chromatography, eluting with DCM/Acetone mixtures, **3p** was collected as a light yellow solid (1.5 g, 91% yield).

^{1}H NMR (400 MHz, Acetone-d_6) δ 7.78 – 7.73 (m, 3H), 7.59 (t, J = 7.7 Hz, 2H), 7.42 (t, J = 7.5 Hz, 1H), 7.33 (d, J = 7.6 Hz, 1H), 7.28 (t, J = 7.5 Hz, 1H), 7.21 (d, J = 7.5 Hz, 1H), 3.98 (s, 6H), 2.13 (s, 3H).

^{13}C NMR (101 MHz, Acetone-d_6) δ 189.70, 139.19, 135.04, 133.48, 132.43, 131.96, 130.89, 130.02, 126.67, 125.53, 118.57, 63.16, 42.31, 17.99.

^{19}F NMR (376 MHz, Acetone-d_6) δ -71.77 (d, J = 708 Hz).

^{31}P NMR (162 MHz, Acetone-d_6) δ -144.19 (h, J = 709 Hz).

HRMS (ESI) calculated for $C_{17}H_{18}IO_2S^+$ $[M-PF_6]^+$ m/z: 413.0067, found: 413.0063.

(1-(dimethyl(oxo)-λ^6-sulfaneylidene)-2-oxo-2-(-phenoxyphenyl)ethyl)(phenyl)iodonium hexafluorophosphate (3q)

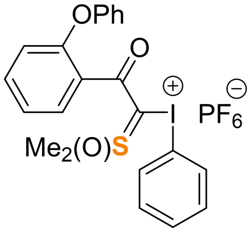

Prepared according to the general procedure B using phenyliodoso diacetate (966.0 mg, 3.0 mmol), HPF_6 (0.41 ml, 65% w/w, 3.0 mmol) and 2-(dimethyl(oxo)-λ^6-sulfanylidene)-1-(2-phenoxyphenyl)ethan-1-one (864.2 g, 3.0 mmol). After filtration, **3q** was collected as a white solid (1.68 g, 88% yield).

^{1}H NMR (400 MHz, Acetone-d_6) δ 7.91 – 7.90 (m, 2H), 7.74 (tt, J = 6.9, 1.1 Hz, 1H), 7.63 – 7.51 (m, 3H), 7.44 (dd, J = 7.6, 1.8 Hz, 1H), 7.34 – 7.27 (m, 3H), 7.15 – 7.09 (m, 1H), 7.05 – 7.03 (m, 1H), 6.81 (d, J = 7.5 Hz, 2H), 3.79 (s, 6H).

^{13}C NMR (101 MHz, Acetone-d_6) δ 186.33, 156.82, 152.86, 133.18, 132.36, 132.10, 132.00, 131.61, 129.91, 129.30, 124.10, 123.74, 119.34, 118.43, 118.09, 62.96, 42.15.

^{19}F NMR (376 MHz, Acetone-d_6) δ -71.56 (d, J = 708 Hz).

^{31}P NMR (162 MHz, Acetone-d_6) δ -144.14 (h, J = 709 Hz).

HRMS (ESI) calculated for $C_{22}H_{10}IO_3S^+$ $[M-PF_6]^+$ m/z: 491.0172, found: 491.0177.

(1-(dimethyl(oxo)-λ^6-sulfaneylidene)-2-oxo-2-(4-nitrilephenyl)ethyl)(phenyl)iodonium hexafluorophosphate (3r)

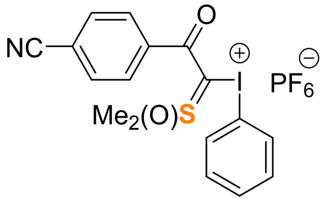

Prepared according to the general procedure B using phenyliodoso diacetate (966.0 mg, 3.0 mmol), HPF_6 (0.41 ml, 65% w/w, 3.0 mmol) and 4-(2-(dimethyl(oxo)-λ^6-sulfanylidene)acetyl)benzonitrile (663.2 mg, 3.0 mmol). After filtration, **3r** was collected as a white solid (1.14 g, 65% yield).

^{1}H NMR (400 MHz, Acetone-d_6) δ 8.00 (d, J = 7.9 Hz, 2H), 7.95 (d, J = 7.9 Hz, 2H), 7.77 (t, J = 7.4 Hz, 1H), 7.71 (d, J = 7.9 Hz, 2H), 7.65 (t, J = 7.8 Hz, 2H), 3.98 (s, 6H).

^{13}C NMR (101 MHz, Acetone-d_6) δ 187.03, 143.19, 133.01, 132.52, 132.50, 132.28, 128.24, 118.66, 117.84, 114.36, 62.44, 42.11.

^{19}F NMR (376 MHz, Acetone-d_6) δ -71.77 (d, J = 708 Hz).

^{31}P NMR (162 MHz, Acetone-d_6) δ -144.20 (h, J = 709 Hz).

HRMS (ESI) calculated for $C_{17}H_{15}INO_2S^+$ $[M-PF_6]^+$ m/z: 423.9863, found: 423.9864.

(1-(dimethyl(oxo)-λ^6-sulfaneylidene)-2-oxo-2-(naphthalen-1-yl)ethyl)(phenyl)iodonium tetrafluoroborate (3s)

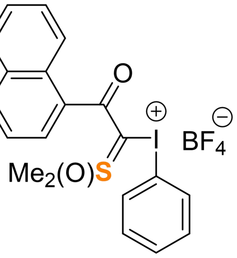

Prepared according to the general procedure B using phenyliodoso diacetate (966.0 mg, 3.0 mmol), HBF_4 (0.40 ml, 48% w/w, 3.0 mmol) and 2-(dimethyl(oxo)- λ ^{6}-sulfanylidene)-1-(naphthalen-1-yl)ethan-1-one (738.2 g, 3.0 mmol). After filtration, **3s** was collected as a white solid (1.24 g, 75% yield).

^{1}H NMR (400 MHz, Acetone-d_6) δ 8.05 (d, J = 7.9 Hz, 1H), 8.01 (d, J = 8.2 Hz, 1H), 7.84 (d, J = 8.4 Hz, 1H), 7.68 – 7.48 (m, 6H), 7.42 – 7.38 (m, 3H), 4.20 – 3.97 (m, 6H).

^{13}C NMR (101 MHz, Acetone-d_6) δ 189.15, 137.24, 133.59, 132.96, 132.00, 131.66, 130.21, 129.59, 128.44, 127.08, 126.62, 125.07, 125.05, 124.79, 118.36, 64.62, 42.26.

^{19}F NMR (376 MHz, Acetone-d_6) δ -149.05.

HRMS (ESI) calculated for $C_{20}H_{18}IO_2S^+$ $[M-BF_4]^+$ m/z: 449.0067, found: 449.0065.

(1-(dimethyl(oxo)-λ^6-sulfaneylidene)-2-oxo-2-(3-phenyl)propan)(phenyl)iodonium hexafluorophosphate (3t)

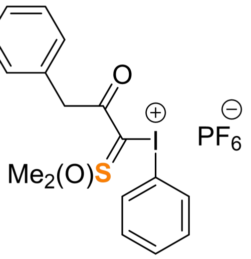

Prepared according to the general procedure B using phenyliodoso diacetate (1.6 g, 5.0 mmol), HPF_6 (0.68 ml, 65% w/w, 5.0 mmol) and 1-(dimethyl(oxo)-λ^6-sulfanylidene)-3-phenylpropan-2-one (1.0 g, 5.0 mmol). After flash column chromatography, eluting with DCM/Acetone mixtures, **3t** was collected as a white foamy solid (2.5 g, 91% yield).

^{1}H NMR (400 MHz, Acetone-d_6) δ 7.90 (d, J = 8.2 Hz, 2H), 7.70 (t, J = 7.4 Hz, 1H), 7.59 – 7.48 (m, 2H), 7.35 – 7.21 (m, 5H), 4.22 (s, 2H), 3.83 (s, 6H).

^{13}C NMR (101 MHz, Acetone-d_6) δ 189.36, 135.15, 132.59, 132.05, 131.89, 129.33, 128.58, 126.88, 118.28, 59.43, 45.61, 42.41.

^{19}F NMR (376 MHz, Acetone-d_6) δ -72.18 (d, J = 707 Hz).

^{31}P NMR (162 MHz, Acetone-d_6) δ -144.22 (h, J = 708 Hz).

HRMS (ESI) calculated for $C_{17}H_{18}IO_2S^+$ $[M-PF_6]^+$ m/z: 413.0067, found: 413.0082.

(1-(dimethyl(oxo)-λ^6-sulfaneylidene)-2-oxo-2-(4-phenylbutan))(phenyl)iodonium hexafluorophosphate (3u)

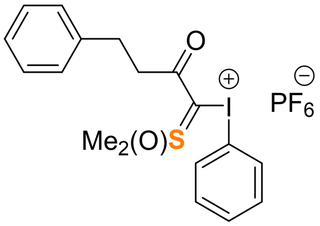

Prepared according to the general procedure A using phenyliodoso diacetate (966.0 mg, 3.0 mmol), HPF_6 (0.41 ml, 65% w/w, 3.0 mmol) and 1-(dimethyl(oxo)-λ^6-sulfanylidene)-4-phenylbutan-2-one (672.3 mg, 3.0 mmol). After flash column chromatography, eluting with DCM/Acetone mixtures, **3u** was collected as a white foamy solid (1.4 g, 81% yield).

^{1}H NMR (400 MHz, CD_3CN) δ 7.83 (d, J = 8.3 Hz, 2H), 7.69 (t, J = 7.5 Hz, 1H), 7.54 (t, J = 7.9 Hz, 2H), 7.34 – 7.26 (m, 2H), 7.25 – 7.21 (m, 3H), 3.58 (s, 6H), 3.07 – 3.03 (m, 2H), 2.94 – 2.90 (m, 2H).

^{13}C NMR (101 MHz, CD_3CN) δ 190.88, 140.95, 132.70, 132.35, 132.13, 128.49, 126.22, 117.89, 59.13, 42.85, 39.97, 30.77.

^{19}F NMR (376 MHz, CD_3CN) δ -72.45 (d, J = 708 Hz).

^{31}P NMR (162 MHz, CD_3CN) δ -144.54 (h, J = 707 Hz).

HRMS (ESI) calculated for $C_{18}H_{20}IO_2S^+$ $[M-PF_6]^+$ m/z: 427.0223, found: 427.0229.

(*E*)(1-(dimethyl(oxo)-λ^6-sulfaneylidene)-2-oxo-2-(6-(*p*-tolyl)hex-5-en-2-one))(phenyl)iodonium hexafluorophosphate (3v)

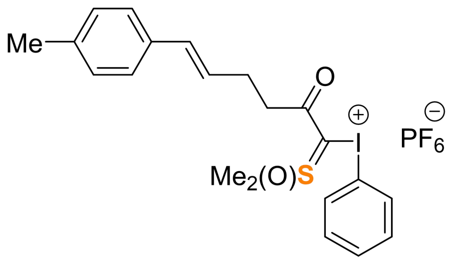

Prepared according to the *general procedure B* using phenyliodoso diacetate (966.0 mg, 3.0 mmol), HPF_6 (0.41 ml, 65% w/w, 3.0 mmol) and (*E*)-1-(dimethyl(oxo)-λ^6-sulfaneylidene)-6-(p-tolyl)hex-5-en-2-one (792.3 mg, 3.0 mmol). After flash column chromatography, eluting with DCM/Acetone mixtures, **3v** was collected as a white foamy solid (1.3 g, 70% yield).

^{1}H NMR (400 MHz, Acetone-d_6) δ 8.18 – 8.08 (m, 2H), 7.76 – 7.66 (m, 1H), 7.63 – 7.53 (m, 2H), 7.20 (d, J = 8.1 Hz, 2H), 7.10 (d, J = 7.9 Hz, 2H), 6.37 (d, J = 15.9 Hz, 1H), 6.21 (dt, J = 15.9, 6.8 Hz, 1H), 3.79 (s, 6H), 3.02 (t, J = 7.3 Hz, 2H), 2.48 (dt, J = 6.8, 7.3 Hz, 2H), 2.28 (s, 3H).

^{13}C NMR (101 MHz, Acetone-d_6) δ 190.86, 136.61, 134.77, 132.74, 132.20, 132.10, 130.63, 129.11, 127.77, 125.92, 118.45, 59.71, 42.57, 38.32, 20.25.

^{19}F NMR (376 MHz, Acetone-d_6) δ -71.94 (d, J = 707 Hz).

^{31}**P NMR** (162 MHz, Acetone-*d$_6$*) δ -144.21 (h, *J* = 708 Hz).

HRMS (ESI) calculated for $[C_{21}H_{24}IO_2S]^+$ $[M\text{-}PF_6]^+$ m/z: 467.0536, found: 467.0538.

(*E*)(1-(dimethyl(oxo)-λ^6-sulfaneylidene)-2-oxo-2-(non-5-en-2-one))(phenyl)iodonium hexafluorophosphate (3w)

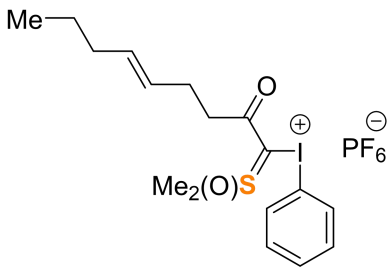

Prepared according to the *general procedure B* using phenyliodoso diacetate (966.0 mg, 3.0 mmol), HPF_6 (0.41 ml, 65% w/w, 3.0 mmol) and (*E*)-1-(dimethyl(oxo)-λ^6-sulfaneylidene)non-5-en-2-one (648.9 mg, 3.0 mmol). After flash column chromatography, eluting with DCM/Acetone mixtures, **3w** was collected as a white foamy solid (1.5 g, 88% yield).

^{1}**H NMR** (400 MHz, Acetone-*d$_6$*) δ 8.17 – 8.09 (m, 2H), 7.78 – 7.69 (m, 1H), 7.64 – 7.59 (m, 2H), 5.40 (td, *J* = 3.7, 1.8 Hz, 2H), 3.79 (s, 6H), 2.89 (t, *J* = 7.3 Hz, 2H), 2.31 – 2.22 (m, 2H), 1.91 – 1.86 (m, 2H), 1.29 (h, *J* = 7.4 Hz, 2H), 0.83 (t, *J* = 7.4 Hz, 3H).

^{13}**C NMR** (101 MHz, Acetone-*d$_6$*) δ 191.02, 132.68, 132.20, 132.09, 131.08, 128.62, 118.41, 59.52, 42.60, 38.53, 34.37, 27.99, 22.37, 13.00.

^{19}**F NMR** (376 MHz, Acetone-*d$_6$*) δ -71.97 (d, *J* = 707 Hz).

^{31}**P NMR** (162 MHz, Acetone-*d$_6$*) δ -144.23 (h, *J* = 708 Hz).

HRMS (ESI) calculated for $[C_{17}H_{24}IO_2S]^+$ $[M\text{-}PF_6]^+$ m/z: 419.0536, found: 419.0539.

2.2 Light-mediated Tandem [4+2] Cycloaddition: reaction scope.

General Procedure C

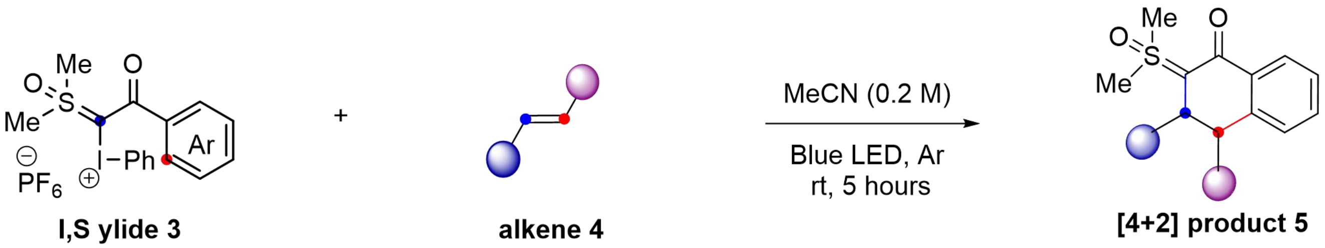

To a 10 mL oven-dried tube equipped with a stirring bar was added reagent **3** (0.2 mmol, 1.0 equiv.), alkene **4** (0.8 mmol, 4.0 equiv.), anhydrous MeCN (1.0 mL). The reaction vial was capped with a rubber septum under an argon atmosphere, and it was fixed on a SynLED 4x4 photoreactor (SynLED discover™ 452 nm, 1W, designed and manufactured by Shenzhen SynLED Tech. Ltd., see (see the picture below for reaction setup) for 5 hours. Solvent was removed under *vacuum* and the crude mixture was purified by flash column chromatography to yield the corresponding products **5**.

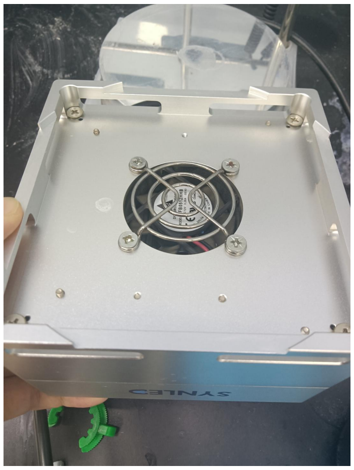

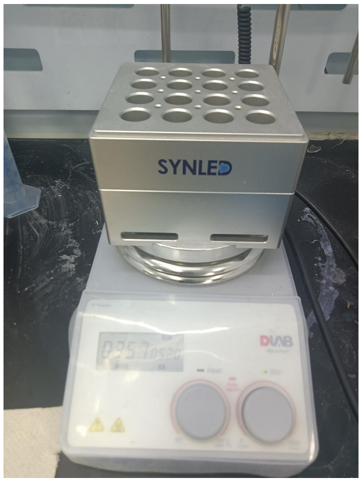

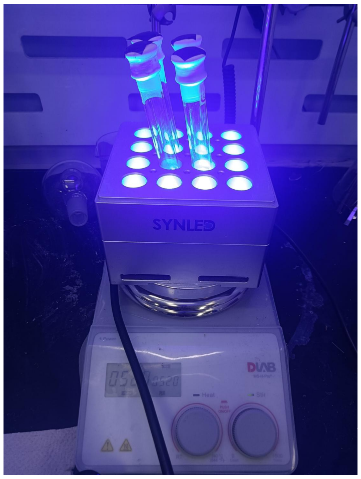

Supplementary Figure 2. Experimental setup for light-mediated cloaddition reaction.

2-(dimethyl(oxo)-λ^6-sulfanylidene)-4-ethyl-3,4-dihydronaphthalen-1(2*H*)-one (5a)

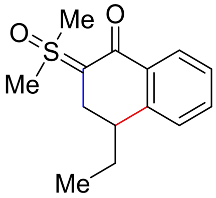

This compound was synthesized following the *general procedure C* using reagent **3c** (108.8 mg, 0.2 mmol), and but-1-ene (0.8 ml, 0.8 mmol， 10% in hexane). Purification by flash chromatography on silica gel (DCM/ MeOH: 30/1) provided the title compound as white solid (39.0 mg, 78% yield). Ratio of isomers was determined to be >20:1 from both the crude reaction mixture and purified products using ^{1}H NMR spectroscopy.

^{1}H NMR (400 MHz, $CDCl_3$) δ 7.91 (dd, *J* = 7.4, 1.7 Hz, 1H), 7.34 (td, *J* = 7.3, 1.7 Hz, 1H), 7.28 (td, *J* = 7.4, 1.5 Hz, 1H), 7.16 (d, *J* = 6.6 Hz, 1H), 3.57 (s, 3H), 3.54 (s, 3H), 2.80 – 2.71 (m, 2H), 2.70 – 2.62 (m, 1H), 1.64 (p, *J* = 7.3 Hz, 2H), 0.92 (t, *J* = 7.4 Hz, 3H).

^{13}C NMR (101 MHz, $CDCl_3$) δ 176.15, 144.33, 134.55, 130.34, 127.60, 126.51, 124.92, 71.23, 42.65, 42.40, 40.76, 27.18, 23.89, 12.21.

HRMS (ESI-TOF) $[M+H]^+$ calculated for $[C_{14}H_{19}O_2S]^+$ m/z: 251.1100, found 251.1092.

2-(dimethyl(oxo)-λ^6-sulfanylidene)-4-propyl-3,4-dihydronaphthalen-1(2*H*)-one (5b)

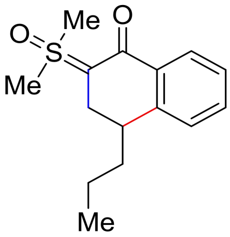

This compound was synthesized following the *general procedure C* using reagent **3c** (108.8 mg, 0.2 mmol), and pent-1-ene (56.0 mg, 0.8 mmol). Purification by flash chromatography on silica gel (DCM/ MeOH: 30/1) provided the title compound as white solid (45.4 mg, 86% yield). Ratio of isomers was determined to be >20:1 from both the crude reaction mixture and purified products using ^{1}H NMR spectroscopy.

^{1}H NMR (400 MHz, $CDCl_3$) δ 7.91 (dd, *J* = 7.5, 1.7 Hz, 1H), 7.33 (td, *J* = 7.3, 1.7 Hz, 1H), 7.28 (td, *J* = 7.3, 1.4 Hz, 1H), 7.15 (d, *J* = 7.3 Hz, 1H), 3.57 (s, 3H), 3.54 (s, 3H), 2.86 (tt, *J* = 7.5, 3.9 Hz, 1H), 2.80 – 2.59 (m, 2H), 1.65 – 1.53 (m, 2H), 1.47 – 1.33 (m, 1H), 1.30 – 1.21 (m, 1H), 0.89 (t, *J* = 7.3 Hz, 3H).

^{13}C NMR (101 MHz, $CDCl_3$) δ 176.26, 144.66, 134.63, 130.42, 127.57, 126.55, 125.01, 71.27, 42.71, 42.49, 38.84, 36.69, 24.34, 20.75, 14.25.

HRMS (ESI-TOF) [M] calculated for [$C_{15}H_{20}O_2S$] m/z: 264.1184, found 264.1123.

4-butyl-2-(dimethyl(oxo)-λ^6-sulfanylidene)-3,4-dihydronaphthalen-1(2*H*)-one (5c)

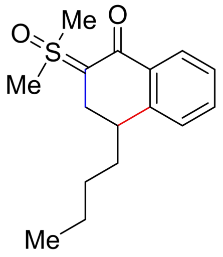

This compound was synthesized following the *general procedure C* using reagent **3c** (108.8 mg, 0.2 mmol), and hex-1-ene (67.2 mg, 0.8 mmol). Purification by flash chromatography on silica gel (DCM/ MeOH: 30/1) provided the title compound as white solid (50.0 mg, 90% yield). Ratio of isomers was determined to be >20:1 from both the crude reaction mixture and purified products using ^{1}H NMR spectroscopy.

^{1}H NMR (400 MHz, $CDCl_3$) δ 7.93 (dd, *J* = 7.5, 1.6 Hz, 1H), 7.36 (td, *J* = 7.4, 1.3 Hz, 1H), 7.31 (td, *J* = 7.4, 1.5 Hz, 1H), 7.17 (dd, *J* = 7.3, 1.5 Hz, 1H), 3.59 (s, 3H), 3.56 (s, 3H), 2.6 (tt, *J* = 7.4, 4.0 Hz, 1H), 2.81 – 2.62 (m, 2H), 1.69 – 1.55 (m, 2H), 1.40 – 1.25 (m, 4H), 0.89 (t, *J* = 7.0 Hz, 3H).

^{13}C NMR (101 MHz, $CDCl_3$) δ 176.27, 144.73, 134.61, 130.46, 127.56, 126.55, 124.99, 71.22, 42.71, 42.49, 39.10, 34.11, 29.83, 24.28, 22.86, 14.14.

HRMS (ESI-TOF) [M] calculated for [$C_{16}H_{22}O_2S$] m/z: 278.1341, found 278.1277.

2-(dimethyl(oxo)-λ^6-sulfanylidene)-4-pentyl-3,4-dihydronaphthalen-1(2*H*)-one (5d)

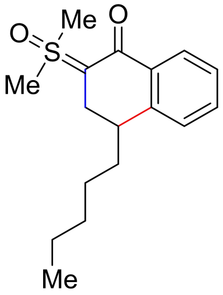

This compound was synthesized following the *general procedure C* using reagent **3c** (108.8 mg, 0.2 mmol), and hept-1-ene (79.0 mg, 0.8 mmol). Purification by flash chromatography on silica gel (DCM/ MeOH:

30/1) provided the title compound as white solid (53.1 mg, 91% yield). Ratio of isomers was determined to be >20:1 from both the crude reaction mixture and purified products using ^{1}H NMR spectroscopy.

^{1}H NMR (400 MHz, $CDCl_3$) δ 7.92 (dd, *J* = 7.5, 1.6 Hz, 1H), 7.35 (td, *J* = 7.3, 1.7 Hz, 1H), 7.35 (td, *J* = 7.5, 1.6 Hz, 1H), 7.17 (dd, *J* = 7.4, 1.5 Hz, 1H), 3.58 (s, 3H), 3.56 (s, 3H), 2.86 (tt, *J* = 7.4, 4.3 Hz, 1H), 2.81 – 2.61 (m, 2H), 1.65 – 1.55 (m, 2H), 1.45 – 1.35 (m, 1H), 1.33 – 1.22 (m, 5H), 0.88 (t, *J* = 6.9 Hz, 3H).

^{13}C NMR (101 MHz, $CDCl_3$) δ 176.24, 144.73, 134.60, 130.45, 127.54, 126.53, 124.99, 71.28, 42.71, 42.47, 39.10, 34.36, 31.99, 27.27, 24.26, 22.64, 14.15.

HRMS (ESI-TOF) $[M+H]^+$ calculated for $[C_{17}H_{25}O_2S]^+$ m/z: 293.1570, found 293.1568.

2-(dimethyl(oxo)-λ^6-sulfanylidene)-4-hexyl-3,4-dihydronaphthalen-1(2*H*)-one (5e)

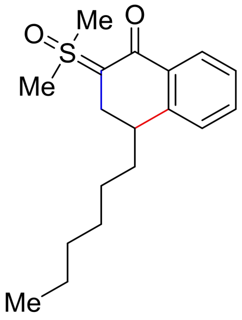

This compound was synthesized following the *general procedure C* using reagent **3c** (108.8 mg, 0.2 mmol), and oct-1-ene (89.6 mg, 0.8 mmol). Purification by flash chromatography on silica gel (DCM/ MeOH: 30/1) provided the title compound as white solid (58.2 mg, 95% yield). Ratio of isomers was determined to be >20:1 from both the crude reaction mixture and purified products using ^{1}H NMR spectroscopy.

^{1}H NMR (400 MHz, Acetone-*d_6*) δ 7.90 (dd, *J* = 7.6, 1.6 Hz, 1H), 7.33 (td, *J* = 7.4, 1.6 Hz, 1H), 7.26 (td, *J* = 7.5, 1.4 Hz, 1H), 7.20 (d, *J* = 7.5 Hz, 1H), 3.63 (s, 3H), 3.60 (s, 3H), 2.88 – 2.80 (m, 1H), 2.76 – 2.62 (m, 2H), 1.74 – 1.62 (m, 1H), 1.61 – 1.51 (m, 1H), 1.45 – 1.37 (m, 1H), 1.34 – 1.28 (m, 8H), 0.88 (t, *J* = 6.4 Hz, 3H).

^{13}C NMR (101 MHz, Acetone-*d_6*) δ 174.89, 144.73, 135.49, 129.70, 127.26, 125.92, 125.06, 70.65, 70.61, 41.12, 41.06, 39.04, 34.29, 31.70, 27.43, 24.34, 22.41, 13.47.

HRMS (ESI-TOF) $[M+Na]^+$ calculated for $[C_{18}H_{26}NaO_2S]^+$ m/z: 329.1546, found 329.1543.

2-(dimethyl(oxo)- λ^6-sulfanylidene)-4-octyl-3,4-dihydronaphthalen-1(2*H*)-one (5f)

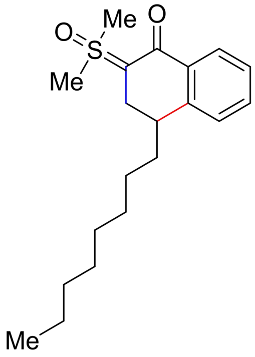

This compound was synthesized following the *general procedure C* using reagent **3c** (108.8 mg, 0.2 mmol), and dec-1-ene (112.0 mg, 0.8 mmol). Purification by flash chromatography on silica gel (DCM/ MeOH: 30/1) provided the title compound as white solid (61.6 mg, 92% yield). Ratio of isomers was determined to be >20:1 from both the crude reaction mixture and purified products using ^{1}H NMR spectroscopy.

^{1}H NMR (400 MHz, $CDCl_3$) δ 8.32 (dd, *J* = 7.5, 1.7 Hz, 1H), 7.75 (td, *J* = 7.3, 1.7 Hz, 1H), 7.70 (td, *J* = 7.5, 1.6 Hz, 1H), 7.57 (dd, *J* = 7.3, 1.5 Hz, 1H), 3.98 (s, 3H), 3.96 (s, 3H), 3.28 – 3.22 (m, 1H), 3.25 (tt, *J* = 7.5, 4.2 Hz, 1H),, 2.04 – 1.97 (m, 2H), 1.82 – 1.74 (m, 1H), 1.70 – 1.63 (m, 11H), 1.28 (t, *J* = 6.8 Hz, 3H).

^{13}C NMR (101 MHz, $CDCl_3$) δ 176.28, 144.75, 134.62, 130.46, 127.56, 126.54, 124.99, 71.24, 42.73, 42.50, 39.12, 34.42, 31.95, 29.82, 29.62, 29.39, 27.63, 24.27, 22.74, 14.19.

HRMS (ESI-TOF) $[M+H]^+$ calculated for $[C_{20}H_{31}O_2S]^+$ m/z: 335.2039, found 335.2039.

4-decyl-2-(dimethyl(oxo)-λ^6-sulfanylidene)-3,4-dihydronaphthalen-1(2*H*)-one (5g)

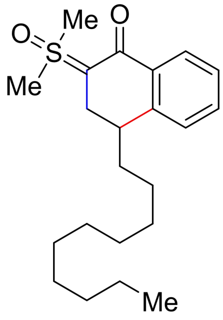

This compound was synthesized following the *general procedure C* using reagent **3c** (108.8 mg, 0.2 mmol), and dodec-1-ene (134.4 mg, 0.8 mmol). Purification by flash chromatography on silica gel (DCM/ MeOH: 30/1) provided the title compound as white solid (65.3 mg, 90% yield). Ratio of isomers was determined to be >20:1 from both the crude reaction mixture and purified products using ^{1}H NMR spectroscopy.

^{1}**H NMR** (400 MHz, $CDCl_3$) δ 7.91 (dd, *J* = 7.6, 1.6 Hz, 1H), 7.35 (td, *J* = 7.3, 1.7 Hz, 1H), 7.29 (td, *J* = 7.4, 1.5 Hz, 1H), 7.16 (dd, *J* = 7.3, 1.5 Hz, 1H), 3.58 (s, 3H), 3.55 (s, 3H), 2.85 (tt, *J* = 7.4, 3.9 Hz, 1H), 2.80 – 2.61 (m, 2H), 1.65 – 1.56 (m, 2H), 1.42 – 1.34 (m, 1H), 1.30 – 1.22 (m, 15H), 0.88 (t, *J* = 6.8 Hz, 3H).

^{13}**C NMR** (101 MHz, $CDCl_3$) δ 176.21, 144.73, 134.58, 130.45, 127.54, 126.53, 124.99, 71.39, 42.71, 42.48, 39.09, 34.40, 31.97, 29.81, 29.72, 29.67, 29.65, 29.39, 27.62, 24.27, 22.74, 14.18.

HRMS (ESI-TOF) [M] calculated for [$C_{22}H_{34}O_2S$] m/z: 362.2280, found 362.2232.

4-(cyclohexylmethyl)-2-(dimethyl(oxo)-λ^6-sulfanylidene)-3,4-dihydronaphthalen-1(2*H*)-one (5h)

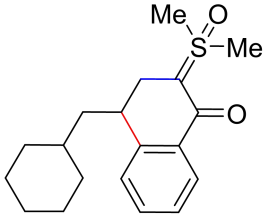

This compound was synthesized following the *general procedure C* using reagent **3c** (108.8 mg, 0.2 mmol), and allylcyclohexane (99.4 mg, 0.8 mmol). Purification by flash chromatography on silica gel (DCM/ MeOH: 30/1) provided the title compound as white solid (44.6 mg, 70% yield). Ratio of isomers was determined to be >20:1 from both the crude reaction mixture and purified products using ^{1}H NMR spectroscopy.

^{1}**H NMR** (400 MHz, $CDCl_3$) δ 7.91 (dd, *J* = 7.6, 1.6 Hz, 1H), 7.35 (td, *J* = 7.4, 1.7 Hz, 1H), 7.30 (td, *J* = 7.4, 1.5 Hz, 1H), 7.15 (dd, *J* = 7.4, 1.4 Hz, 1H), 3.58 (s, 3H), 3.56 (s, 3H), 3.06 – 2.95 (m, 1H), 2.80 – 2.57 (m, 2H), 1.78 – 1.64 (m, 5H), 1.57 – 1.50 (m, 1H), 1.44 – 1.40 (m, 1H), 1.33 – 1.12 (m, 5H), 1.01 – 0.83 (m, 2H).

^{13}**C NMR** (101 MHz, $CDCl_3$) δ 176.18, 145.10, 134.56, 130.51, 127.26, 126.42, 124.96, 71.33, 42.67, 42.39, 42.16, 35.69, 34.88, 34.13, 33.08, 26.64, 26.29, 26.15, 24.10.

HRMS (ESI-TOF) $[M+H]^+$ calculated for $[C_{19}H_{27}O_2S]^+$ m/z: 319.1726, found 319.1680.

4-benzyl-2-(dimethyl(oxo)-λ^6-sulfanylidene)-3,4-dihydronaphthalen-1(2*H*)-one (5i)

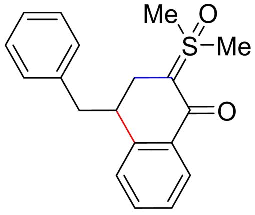

This compound was synthesized following the *general procedure C* using reagent **3c** (108.8 mg, 0.2 mmol), and allylbenzene (94.5 mg, 0.8 mmol). Purification by flash chromatography on silica gel (DCM/ MeOH: 30/1) provided the title compound as white solid (56.0 mg, 86% yield). Ratio of isomers was determined to be >20:1 from both the crude reaction mixture and purified products using ^{1}H NMR spectroscopy.

^{1}H NMR (400 MHz, $CDCl_3$) δ 7.98 (dd, *J* = 7.1, 2.0 Hz, 1H), 7.36 – 7.31 (m, 2H), 7.29 (dd, *J* = 4.6, 2.1 Hz, 2H), 7.25 – 7.19 (m, 1H), 7.08 (d, *J* = 7.0 Hz, 2H), 6.99 (dd, *J* = 7.0, 1.8 Hz, 1H), 3.65 (s, 3H), 3.54 (s, 3H), 3.19 – 3.11 (m, 1H), 2.98 – 2.85 (m, 2H), 2.73 (dd, *J* = 14.2, 4.9 Hz, 1H), 2.65 (dd, *J* = 14.3, 3.0 Hz, 1H).

^{13}C NMR (101 MHz, $CDCl_3$) δ 176.16, 143.49, 140.16, 134.53, 130.53, 129.31, 128.38, 127.88, 126.86, 126.24, 125.06, 71.44, 42.88, 42.47, 41.33, 41.15, 23.80.

HRMS (ESI-TOF) $[M+H]^+$ calculated for $[C_{19}H_{21}O_2S]^+$ m/z: 313.1257, found 313.1269.

2-(dimethyl(oxo)-λ^6-sulfanylidene)-4-phenethyl-3,4-dihydronaphthalen-1(2*H*)-one (5j)

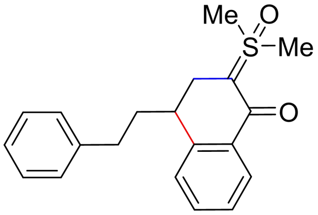

This compound was synthesized following the *general procedure C* using reagent **3c** (108.8 mg, 0.2 mmol), and but-3-en-1-ylbenzene (105.6 mg, 0.8 mmol). Purification by flash chromatography on silica gel (DCM/ MeOH: 30/1) provided the title compound as white solid (56.0 mg, 86% yield). Ratio of isomers was determined to be >20:1 from both the crude reaction mixture and purified products using ^{1}H NMR spectroscopy.

^{1}H NMR (400 MHz, $CDCl_3$) δ 7.95 (dd, *J* = 7.4, 1.7 Hz, 1H), 7.42 – 7.26 (m, 4H), 7.24 – 7.10 (m, 4H), 3.59 (s, 3H), 3.55 (s, 3H), 2.93 (tt, *J* = 7.4, 3.8 Hz, 1H), 2.83 (dd, *J* = 14.2, 4.7 Hz, 1H), 2.79 – 2.68 (m, 2H), 2.67 – 2.54 (m, 1H), 2.00 – 1.94 (m, 2H).

^{13}C NMR (101 MHz, $CDCl_3$) δ 176.16, 144.12, 142.19, 134.65, 130.51, 128.46, 128.41, 127.64, 126.74, 125.87, 125.12, 71.15, 42.63, 42.56, 38.72, 36.10, 33.82, 24.38.

HRMS (ESI-TOF) [M] calculated for [$C_{20}H_{22}O_2S$] m/z: 326.1341, found 326.1276.

4-((benzyloxy)methyl)-2-(dimethyl(oxo)-λ^6-sulfanylidene)-3,4-dihydronaphthalen-1(2*H*)-one (5k)

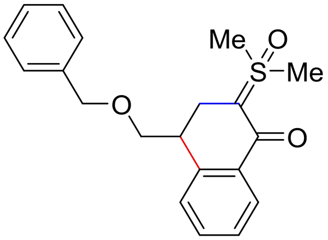

This compound was synthesized following the *general procedure C* using reagent **3c** (108.8 mg, 0.2 mmol), and ((allyloxy)methyl)benzene (118.4 mg, 0.8 mmol). Purification by flash chromatography on silica gel (DCM/ MeOH: 30/1) provided the title compound as white solid (47.8 mg, 70% yield). Ratio of isomers was determined to be >20:1 from both the crude reaction mixture and purified products using ^{1}H NMR spectroscopy.

^{1}H NMR (400 MHz, $CDCl_3$) δ 7.92 (dd, *J* = 7.4, 1.8 Hz, 1H), 7.40 – 7.32 (m, 6H), 7.32 – 7.29 (m, 1H), 7.24 (dd, *J* = 7.1, 1.7 Hz, 1H), 4.65 (d, *J* = 12.2 Hz, 1H), 4.41 (d, *J* = 12.2 Hz, 1H), 3.70 – 3.60 (m, 1H), 3.54 (s, 3H), 3.42 (dd, *J* = 9.3, 4.8 Hz, 1H), 3.36 (s, 3H), 3.29 – 3.25 (m, 1H), 3.00 (dd, *J* = 14.2, 3.0 Hz, 1H), 2.74 (dd, *J* = 14.2, 4.9 Hz, 1H).

^{13}C NMR (101 MHz, $CDCl_3$) δ 175.72, 140.33, 138.39, 135.31, 130.72, 128.55, 128.10, 127.74, 127.26, 125.06, 73.12, 71.32, 42.57, 42.18, 39.57, 21.37.

HRMS (ESI-TOF) $[M+H]^+$ calculated for $[C_{20}H_{23}O_3S]^+$ m/z: 343.1362, found 343.1360.

4-(3-(dimethyl(oxo)-λ^6-sulfanylidene)-4-oxo-1,2,3,4-tetrahydronaphthalen-1-yl)butyl benzoate (5l)

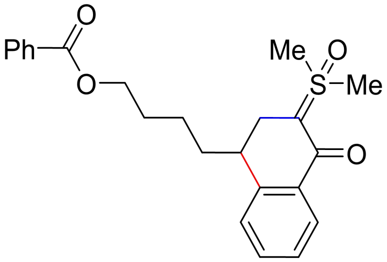

This compound was synthesized following the *general procedure C* using reagent **3c** (108.8 mg, 0.2 mmol), and hex-5-en-1-yl benzoate (163.2 mg, 0.8 mmol). Purification by flash chromatography on silica gel (DCM/ MeOH: 30/1) provided the title compound as white solid (45.4 mg, 62% yield). Ratio of isomers was determined to be >20:1 from both the crude reaction mixture and purified products using ^{1}H NMR spectroscopy.

^{1}H NMR (400 MHz, Acetone-d_6) δ 8.08 – 7.99 (m, 2H), 7.90 (dd, *J* = 7.6, 1.6 Hz, 1H), 7.64 (tt, *J* = 6.8, 1.4 Hz, 1H), 7.54 – 7.50 (m, 2H), 7.32 (td, *J* = 7.3, 1.6 Hz, 1H), 7.26 (td, *J* = 7.4, 1.5 Hz, 1H), 7.21 (dd, *J* = 7.4, 1.4 Hz, 1H), 4.31 (t, *J* = 6.6 Hz, 2H), 3.63 (s, 3H), 3.60 (s, 3H), 2.87 – 2.82 (m, 1H), 2.78 – 2.63 (m, 2H), 1.84 – 1.75 (m, 2H), 1.74 – 1.67 (m, 1H), 1.67 – 1.56 (m, 1H), 1.56 – 1.45 (m, 2H), 1.41 – 1.33 (m, 1H).

^{13}C NMR (101 MHz, Acetone-d_6) δ 174.88, 165.86, 144.59, 135.52, 132.88, 130.60, 129.69, 129.24, 128.50, 127.29, 125.95, 125.07, 70.48, 64.62, 41.11, 41.03, 38.98, 34.19, 27.12, 25.95, 24.42.

HRMS (ESI-TOF) $[M+H]^+$ calculated for $[C_{23}H_{27}O_4S]^+$ m/z: 399.1625, found 399.1630.

3-(dimethyl(oxo)-λ^6-sulfanylidene)-4-oxo-1,2,3,4-tetrahydronaphthalen-1-yl acetate (5m)

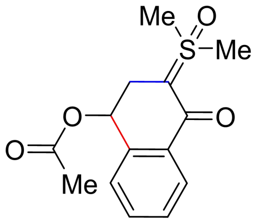

This compound was synthesized following the *general procedure C* using reagent **3c** (108.8 mg, 0.2 mmol), and vinyl acetate (68.8 mg, 0.8 mmol). Purification by flash chromatography on silica gel (DCM/ MeOH: 30/1) provided the title compound as white solid (25.8 mg, 46% yield). Ratio of isomers was determined to be >20:1 from both the crude reaction mixture and purified products using ^{1}H NMR spectroscopy.

^{1}H NMR (400 MHz, $CDCl_3$) δ 8.03 – 7.95 (m, 1H), 7.48 – 7.43 (m, 2H), 7.43 – 7.39 (m, 1H), 6.07 (dd, *J* = 6.2, 4.6 Hz, 1H), 3.61 (s, 3H), 3.59 (s, 3H), 2.95 (dd, *J* = 14.7, 4.6 Hz, 1H), 2.87 (dd, *J* = 14.7, 6.2 Hz, 1H), 2.08 (s, 3H).

^{13}C NMR (101 MHz, $CDCl_3$) δ 175.23, 170.43, 137.04, 135.22, 130.81, 128.79, 127.13, 125.04, 69.55, 42.88, 42.30, 25.68, 21.28.

HRMS (ESI-TOF) $[M+Na]^+$ calculated for $[C_{14}H_{16}NaO_4S]^+$ m/z: 303.0662, found 303.0659.

4-(3-(dimethyl(oxo)-λ⁶-sulfanylidene)-4-oxo-1,2,3,4-tetrahydronaphthalen-1-yl)butyl (7,7-dimethylbicyclo[2.2.1]heptan-1-yl)methanesulfonate (5n)

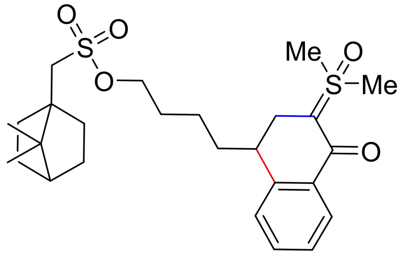

This compound was synthesized following the *general procedure C* using reagent **3c** (108.8 mg, 0.2 mmol), and hex-5-en-1-yl (7,7-dimethylbicyclo[2.2.1]heptan-1-yl)methanesulfonate (240.0 mg, 0.8 mmol). Purification by flash chromatography on silica gel (DCM/ MeOH: 30/1) provided the title compound as white solid (68.8 mg, 70% yield). Ratio of isomers was determined to be >20:1 from both the crude reaction mixture and purified products using ^{1}H NMR spectroscopy.

^{1}H NMR (400 MHz, $CDCl_3$) δ 7.93 (d, *J* = 7.4 Hz, 1H), 7.35 (t, *J* = 7.3 Hz, 1H), 7.30 (t, *J* = 7.3 Hz, 1H), 7.16 (d, *J* = 7.2 Hz, 1H), 4.31 – 4.21 (m, 2H), 3.60 (s, 3H), 3.54 (s, 3H) 2.99 (d, *J* = 15.1 Hz, 1H), 2.90 – 2.74 (m, 2H), 2.66 – 2.60 (m, 1H), 2.53 – 2.36 (m, 2H), 2.17 – 2.00 (m, 2H), 1.96 (d, *J* = 18.4 Hz, 1H), 1.76 – 1.70 (m, 2H), 1.67 – 1.60 (m, 3H), 1.48 – 1.38 (m, 4H), 1.34 – 1.27 (m, 1H), 1.12 (s, 3H), 0.89 (s, 3H).

^{13}C NMR (101 MHz, $CDCl_3$) δ 176.16, 144.32, 134.60, 130.50, 127.56, 126.67, 125.08, 71.24, 70.60, 70.58, 58.02, 48.06, 46.71, 42.82, 42.65, 42.59, 39.02, 34.18, 29.20, 27.08, 26.95, 25.65, 24.94, 24.38, 19.88, 19.78.

HRMS (ESI-TOF) $[M+H]^+$ calculated for $[C_{26}H_{39}O_5S_2]^+$ m/z: 495.2233, found 495.2239.

3-(3-(dimethyl(oxo)-λ⁶-sulfanylidene)-4-oxo-1,2,3,4-tetrahydronaphthalen-1-yl)propyl 4-methylbenzenesulfonate (5o)

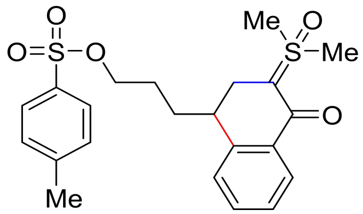

This compound was synthesized following the *general procedure C* using reagent **3c** (108.8 mg, 0.2 mmol), and pent-4-en-1-yl 4-methylbenzenesulfonate (96.6 mg, 0.8 mmol). Purification by flash chromatography on silica gel (DCM/ MeOH: 30/1) provided the title compound as white solid (55.6 mg, 64% yield). Ratio of isomers was determined to be >20:1 from both the crude reaction mixture and purified products using ^{1}H NMR spectroscopy.

^{1}H NMR (400 MHz, $CDCl_3$) δ 7.93 – 7.90 (m, 1H), 7.76 (d, *J* = 8.3 Hz, 2H),7.34 – 7.30 (m, 4H), 7.08 – 7.06 (m, 1H), 4.07 – 3.94 (m, 2H), 3.59 (m, 3H), 3.54 (m, 3H), 2.84 – 2.73 (m, 2H), 2.62 – 2.58 (m, 1H), 2.44 (s, 3H), 1.82 – 1.67 (m, 2H), 1.65 – 1.52 (m, 2H).

^{13}C NMR (101 MHz, $CDCl_3$) δ 175.89, 144.77, 143.47, 134.56, 133.00, 130.47, 129.86, 127.87, 127.42, 126.84, 125.15, 70.93, 70.53, 42.58, 42.36, 38.48, 30.02, 26.98, 24.21, 21.64.

HRMS (ESI-TOF) $[M+H]^+$ calculated for $[C_{22}H_{27}O_5S_2]^+$ m/z: 435.1294, found 435.1299.

2-(dimethyl(oxo)-λ^6-sulfanylidene)-4-(trimethylsilyl)-3,4-dihydronaphthalen-1(2H)-one (5p)

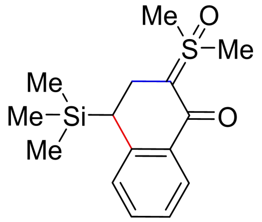

This compound was synthesized following the *general procedure C* using reagent **3c** (108.8 mg, 0.2 mmol), and trimethyl(vinyl)silane (80.0 mg, 0.8 mmol). Purification by flash chromatography on silica gel (DCM/ MeOH: 30/1) provided the title compound as white solid (29.4 mg, 50% yield). Ratio of isomers was determined to be >20:1 from both the crude reaction mixture and purified products using ^{1}H NMR spectroscopy.

^{1}H NMR (400 MHz, $CDCl_3$) δ 7.91 (dd, *J* = 7.7, 1.5 Hz, 1H), 7.30 (td, *J* = 7.4, 1.5 Hz, 1H), 7.20 (td, *J* = 7.6, 1.3 Hz, 1H), 7.02 (d, *J* = 7.5 Hz, 1H), 3.62 (s, 3H), 3.47 (s, 3H), 2.92 (dd, *J* = 13.6, 5.6 Hz, 1H), 2.83 (dd, *J* = 13.6, 1.8 Hz, 1H), 2.49 (dd, *J* = 5.6, 1.8 Hz, 1H), -0.00 (s, 9H).

^{13}C NMR (101 MHz, $CDCl_3$) δ 177.03, 143.69, 134.25, 130.38, 127.05, 125.03, 124.95, 72.07, 43.10, 42.14, 32.15, 21.25, -1.79.

HRMS (ESI-TOF) $[M+H]^+$ calculated for $[C_{15}H_{23}O_2SSi]^+$ m/z: 295.1183, found 295.1187.

2-(3-(dimethyl(oxo)-λ⁶-sulfanylidene)-4-oxo-1,2,3,4-tetrahydronaphthalen-1-yl)ethyl cinnamate (5q)

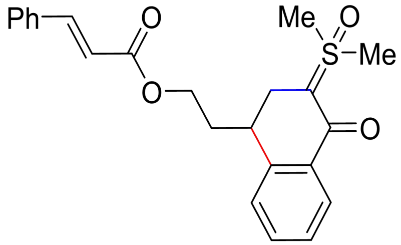

This compound was synthesized following the *general procedure C* using reagent **3c** (108.8 mg, 0.2 mmol), and but-3-en-1-yl cinnamate (161.6 mg, 0.8 mmol). Purification by flash chromatography on silica gel (DCM/ MeOH: 30/1) provided the title compound as white solid (44.4 mg, 56% yield). Ratio of isomers was determined to be >20:1 from both the crude reaction mixture and purified products using ^{1}H NMR spectroscopy.

^{1}H NMR (400 MHz, Acetone-d_6) δ 7.93 (dd, J = 7.5, 1.6 Hz, 1H), 7.76 – 7.68 (m, 3H), 7.49 – 7.43 (m, 3H), 7.36 (td, J = 7.4, 1.6 Hz, 1H), 7.29 (td, J = 7.4, 1.4 Hz, 1H), 7.25 (dd, J = 7.3, 1.4 Hz, 1H), 6.61 (d, J = 16.1 Hz, 1H), 4.28 – 4.15 (m, 2H), 3.70 (d, J = 0.9 Hz, 3H), 3.62 (d, J = 0.9 Hz, 3H) 2.80 (dd, J = 14.2, 4.5 Hz, 1H), 2.74 (dd, J = 14.2, 3.1 Hz, 1H), 2.15 – 2.08 (m, 1H), 2.00 – 1.92 (m, 1H).

^{13}C NMR (101 MHz, Acetone-d_6) δ 174.68, 166.22, 144.35, 143.48, 135.59, 134.58, 130.28, 129.90, 128.93, 128.17, 127.39, 126.33, 125.25, 118.29, 70.54, 62.52, 41.07, 40.97, 36.01, 33.05, 24.60.

HRMS (ESI-TOF) $[M+H]^+$ calculated for $[C_{23}H_{25}O_4S]^+$ m/z: 397.1468, found 397.1475.

2-(dimethyl(oxo)-λ⁶-sulfanylidene)-3,4-diethyl-3,4-dihydronaphthalen-1(2*H*)-one (5r)

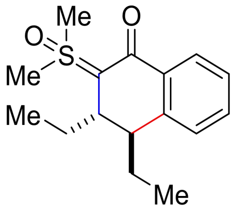

This compound was synthesized following the *general procedure C* using reagent **3c** (108.8 mg, 0.2 mmol), and hex-3-ene (E/Z = 50:50, 67.2 mg, 0.8 mmol). Purification by flash chromatography on silica gel (DCM/ MeOH: 30/1) provided the title compound as yellow oil (30.6 mg, 55% yield). Ratio of isomers was determined to be >20:1 from both the crude reaction mixture and purified products using ^{1}H NMR spectroscopy.

^{1}**H NMR** (400 MHz, $CDCl_3$) δ 7.87 (dd, *J* = 7.4, 1.7 Hz, 1H), 7.33 (td, *J* = 7.3, 1.6 Hz, 1H), 7.28 (td, *J* = 7.4, 1.6 Hz, 1H), 7.13 (dd, *J* = 7.2, 1.5 Hz, 1H), 3.60 (s, 3H), 3.55 (s, 3H), 2.76 (td, *J* = 7.3, 1.9 Hz, 1H), 2.66 (td, *J* = 7.2, 1.9 Hz, 1H), 1.67 – 1.53 (m, 2H), 1.43 – 1.36 (m, 2H), 0.91 (t, *J* = 7.4Hz, 3H), 0.87 (t, *J* = 7.4Hz, 3H).

^{13}**C NMR** (101 MHz, $CDCl_3$) δ 175.60, 143.14, 133.83, 130.31, 128.99, 126.49, 124.80, 45.83, 43.84, 42.11, 37.87, 29.71, 29.04, 12.34, 12.14.

HRMS (ESI-TOF) $[M+H]^+$ calculated for $[C_{16}H_{23}O_2S]^+$ m/z: 279.1413, found 279.1413.

6-(dimethyl(oxo)-λ^6-sulfanylidene)-6a,7,8,9,10,11,12,13,14,15,16,16a-dodecahydrocyclododeca[a]naphthalen-5(6*H*)-one (5s)

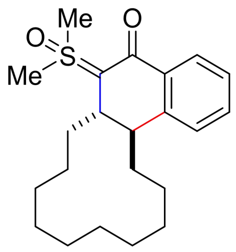

This compound was synthesized following the *general procedure C* using reagent **3c** (54.4 mg, 0.1 mmol), and cyclododecene (*E*/*Z* = 63:37, 133.0 mg, 0.8 mmol). Purification by flash chromatography on silica gel (DCM/ MeOH: 30/1) provided the title compound as white solid (32.4 mg, 45% yield). Ratio of isomers was determined to be >20:1 from both the crude reaction mixture and purified products using ^{1}H NMR spectroscopy.

^{1}**H NMR** (400 MHz, $CDCl_3$) δ 7.88 (dd, *J* = 7.6, 1.5 Hz, 1H), 7.35 (td, *J* = 7.4, 1.4 Hz, 1H), 7.28 – 7.24 (m, 1H), 7.14 (dd, *J* = 7.5, 1.3 Hz, 1H), 3.58 (s, 3H), 3.56 (s, 3H), 3.13 – 3.08 (m, 2H), 1.82 – 1.80 (m, 1H), 1.70 – 1.48 (m, 6H), 1.46 – 1.20 (m, 13H).

^{13}**C NMR** (101 MHz, $CDCl_3$) δ 175.29, 144.01, 133.89, 130.86, 128.92, 126.39, 124.54, 44.06, 42.82, 37.88, 35.24, 34.39, 30.17, 26.44, 26.35, 23.06, 22.82, 22.71, 22.37, 22.31, 22.24.

HRMS (ESI-TOF) $[M+H]^+$ calculated for $[C_{22}H_{33}O_2S]^+$ m/z: 361.2196, found 361.2183.

4-(dimethyl(oxo)-λ^6-sulfanylidene)-1,2,3,3a,4,9b-hexahydro-5H-cyclopenta[a]naphthalen-5-one (5t)

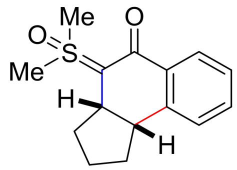

This compound was synthesized following the *general procedure C* using reagent **3c** (108.8 mg, 0.2 mmol), and cyclopentene (54.4 mg, 0.8 mmol). Purification by flash chromatography on silica gel (DCM/ MeOH: 30/1) provided the title compound as white solid (21.0 mg, 40% yield). Ratio of isomers was determined to be >20:1 from both the crude reaction mixture and purified products using ^{1}H NMR spectroscopy.

^{1}H NMR (400 MHz, $CDCl_3$) δ 7.99 (dd, *J* = 7.7, 1.5 Hz, 1H), 7.40 (td, *J* = 7.4, 1.5 Hz, 1H), 7.32 – 7.25 (m, 2H), 3.64 (s, 3H), 3.56 (s, 3H), 3.54 – 3.47 (m, 1H), 3.11 (dt, *J* = 10.2, 8.0 Hz, 1H), 2.31 – 2.16 (m, 2H), 2.03 – 1.95 (m, 1H), 1.68 – 1.57 (m, 2H), 1.46 – 1.36 (m, 1H).

^{13}C NMR (101 MHz, $CDCl_3$) δ 142.49, 133.69, 130.94, 127.07, 125.95, 124.68, 43.54, 42.88, 41.75, 36.57, 35.10, 32.48, 23.45.

HRMS (ESI-TOF) $[M+H]^+$ calculated for $[C_{15}H_{18}O_2S]^+$ m/z: 262.1028, found 262.1025.

10-(dimethyl(oxo)-λ^6-sulfanylidene)-2,3,4,4a,10,10a-hexahydrophenanthren-9(1*H*)-one (5u)

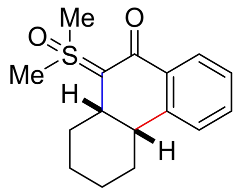

This compound was synthesized following the *general procedure C* using reagent **3c** (108.8 mg, 0.2 mmol), and cyclohexene (65.7 mg, 0.8 mmol). Purification by flash chromatography on silica gel (DCM/ MeOH: 30/1) provided the title compound as white solid (33.1 mg, 60% yield). Ratio of isomers was determined to be >20:1 from both the crude reaction mixture and purified products using ^{1}H NMR spectroscopy.

^{1}H NMR (400 MHz, $CDCl_3$) δ 7.98 (dd, *J* = 7.6, 1.5 Hz, 1H), 7.44 (td, *J* = 7.5, 1.5 Hz, 1H), 7.37 (d, *J* = 7.8 Hz, 1H), 7.32 (tt, *J* = 7.4, 1.2 Hz, 1H), 3.63 (s, 3H), 3.54 (s, 3H), 3.33 – 3.28 (m, 1H), 2.92 – 2.84 (m, 1H), 2.60 – 2.55 (m, 1H), 1.82 (tt, *J* = 13.7, 4.0 Hz, 1H), 1.61 – 1.45 (m, 3H), 1.42 – 1.27 (m, 3H).

^{13}C NMR (101 MHz, $CDCl_3$) δ 175.18, 140.40, 135.41, 130.77, 125.88, 125.81, 125.05, 43.66, 43.49, 38.74, 35.07, 31.33, 27.61, 25.75, 20.17.

HRMS (ESI-TOF) $[M+H]^+$ calculated for $[C_{16}H_{21}O_2S]^+$ m/z: 277.1257, found 277.1252.

The crystal structure of **5u** has been deposited at the Cambridge Crystallographic Data Centre, CCDC 2068911.

10-(dimethyl(oxo)-λ⁶-sulfanylidene)-2,3,4,4a,10,10a-hexahydro-1,4-methanophenanthren-9(1H)-one (5v)

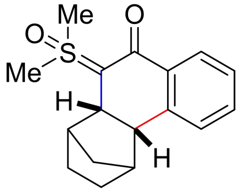

This compound was synthesized following the *general procedure C* using reagent **3c** (108.8 mg, 0.2 mmol), and bicyclo[2.2.1]hept-2-ene (75.2 mg, 0.8 mmol). Purification by flash chromatography on silica gel (DCM/ MeOH: 30/1) provided the title compound as white solid (46.2 mg, 80% yield). Ratio of isomers was determined to be >20:1 from both the crude reaction mixture and purified products using ^{1}H NMR spectroscopy.

^{1}H NMR (400 MHz, Acetone-d_6) δ 7.93 (dd, J = 7.7, 1.5 Hz, 1H), 7.32 (td, J = 7.4, 1.5 Hz, 1H), 7.22 (d, J = 7.6 Hz, 1H), 7.18 (t, J = 7.4 Hz, 1H), 3.68 (s, 3H), 3.60 (s, 3H), 3.22 (d, J = 9.8 Hz, 1H), 3.00 – 2.97 (m, 1H), 2.30 – 2.23 (m, 1H), 2.21 – 2.14 (m, 1H), 1.72 – 1.63 (m, 1H), 1.62 – 1.47 (m, 3H), 1.45 – 1.38 (m, 1H), 1.10 – 1.00 (m, 1H).

^{13}C NMR (101 MHz, Acetone-d_6) δ 172.55, 141.98, 133.76, 129.98, 128.42, 125.19, 123.97, 77.38, 48.46, 47.46, 46.17, 43.65, 41.16, 40.63, 31.74.

HRMS (ESI-TOF) $[M+H]^+$ calculated for $[C_{17}H_{21}O_2S]^+$ m/z: 289.1257, found 289.1259.

4-benzyl-2-(dimethyl(oxo)-λ⁶-sulfanylidene)-4-methyl-3,4-dihydronaphthalen-1(2*H*)-one (5w)

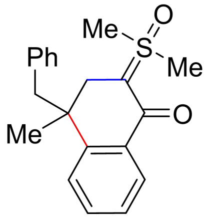

This compound was synthesized following the *general procedure C* using reagent **3c** (108.8 mg, 0.2 mmol), and (2-methylallyl)benzene (105.6 mg, 0.8 mmol). Purification by flash chromatography on silica gel (DCM/ MeOH: 30/1) provided the title compound as yellow oil (52.8 mg, 81% yield). Ratio of isomers was determined to be >20:1 from both the crude reaction mixture and purified products using ^{1}H NMR spectroscopy.

^{1}H NMR (400 MHz, $CDCl_3$) δ 8.03 (dd, J = 7.4, 1.8 Hz, 1H), 7.34 (td, J = 7.3, 1.5 Hz, 1H), 7.30 (td, J = 7.2, 1.7 Hz, 1H), 7.22 – 7.15 (m, 3H), 6.95 (dd, J = 7.5, 1.4 Hz, 1H), 6.82 – 6.80 (m, 2H), 3.67 (s, 3H), 3.55 (s, 3H), 3.03 (d, J = 13.0 Hz, 1H), 2.81 (d, J = 13.0 Hz, 1H), 2.65 (d, J = 14.3 Hz, 1H), 2.59 (d, J = 14.2 Hz, 1H), 1.35 (s, 3H).

^{13}C NMR (101 MHz, $CDCl_3$) δ 175.97, 146.32, 138.13, 134.39, 130.61, 130.44, 127.58, 126.42, 126.08, 125.68, 125.17, 71.89, 46.38, 42.84, 42.39, 38.91, 32.65, 25.09.

HRMS (ESI-TOF) $[M+H]^+$ calculated for $[C_{20}H_{23}O_2S]^+$ m/z: 327.1413, found 327.1422.

4-butyl-7-cyclohexyl-2-(dimethyl(oxo)-λ^6-sulfanylidene)-3,4-dihydronaphthalen-1(2*H*)-one (5x)

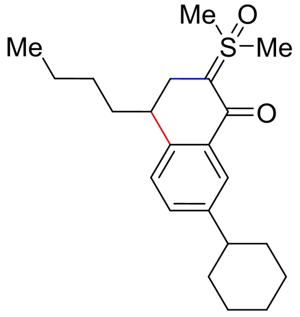

This compound was synthesized following the *general procedure C* using reagent **3f** (125.2 mg, 0.2 mmol), and hex-1-ene (67.2 mg, 0.8 mmol). Purification by flash chromatography on silica gel (DCM/ MeOH: 30/1) provided the title compound as white solid (50.4 mg, 70% yield). Ratio of isomers was determined to be >20:1 from both the crude reaction mixture and purified products using ^{1}H NMR spectroscopy.

^{1}H NMR (400 MHz, $CDCl_3$) δ 7.83 (d, J = 7.9 Hz, 1H), 7.15 (dd, J = 7.9, 1.8 Hz, 1H), 6.99 (d, J = 1.8 Hz, 1H), 3.57 (s, 3H), 3.53 (s, 3H), 2.84 – 2.79 (m, 1H), 2.79 – 2.74 (m, 1H), 2.64 (dd, J = 13.9, 3.1 Hz, 1H), 2.55 – 2.48 (m, 1H), 1.90 – 1.83 (m, 3H), 1.80 – 1.72 (m, 1H), 1.64 – 1.55 (m, 2H), 1.48 – 1.37 (m, 4H), 1.37 – 1.21 (m, 6H), 0.89 (t, J = 6.9 Hz, 3H).

^{13}C NMR (101 MHz, $CDCl_3$) δ 176.55, 150.80, 144.79, 132.44, 126.07, 125.05, 70.58, 44.74, 42.81, 42.60, 39.29, 34.38, 34.14, 29.89, 26.94, 26.23, 24.31, 22.85, 14.16.

HRMS (ESI-TOF) $[M+H]^+$ calculated for $[C_{20}H_{33}O_2S]^+$ m/z: 361.2196, found 361.2186.

4-butyl-2-(dimethyl(oxo)-λ^6-sulfanylidene)-7-methyl-3,4-dihydronaphthalen-1(2*H*)-one (5y)

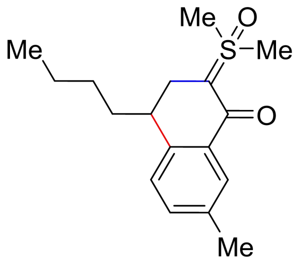

This compound was synthesized following the *general procedure C* using reagent **3g** (111.6 mg, 0.2 mmol), and hex-1-ene (63.2 mg, 0.8 mmol). Purification by flash chromatography on silica gel (DCM/ MeOH: 30/1) provided the title compound as white solid (56.0 mg, 96% yield). Ratio of isomers was determined to be >20:1 from both the crude reaction mixture and purified products using ^{1}H NMR spectroscopy.

^{1}H NMR (400 MHz, $CDCl_3$) δ 7.80 (d, J = 7.8 Hz, 1H), 7.10 (d, J = 7.8 Hz, 1H), 6.97 (s, 1H), 3.57 (s, 3H), 3.54 (s, 3H), 2.79 – 2.60 (m, 3H), 2.37 (s, 3H), 1.70 – 1.51 (m, 2H), 1.42 – 1.18 (m, 4H), 0.89 (t, J = 7.0 Hz, 3H).

^{13}C NMR (101 MHz, $CDCl_3$) δ 176.43, 144.85, 140.67, 132.02, 128.16, 127.31, 125.08, 70.76, 42.75, 42.51, 39.10, 34.06, 29.82, 24.28, 22.83, 21.65, 14.11.

HRMS (ESI-TOF) $[M+H]^+$ calculated for $[C_{17}H_{25}O_2S]^+$ m/z: 293.1570, found 293.1579.

4-butyl-7-chloro-2-(dimethyl(oxo)-λ^6-sulfanylidene)-3,4-dihydronaphthalen-1(2*H*)-one (5z)

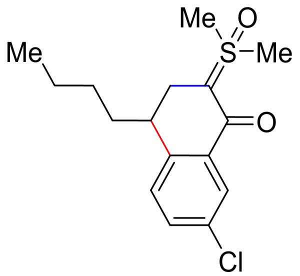

This compound was synthesized following the *general procedure C* using reagent **3h** (115.6 mg, 0.2 mmol), and hex-1-ene (63.2 mg, 0.8 mmol). Purification by flash chromatography on silica gel (DCM/ MeOH:

30/1) provided the title compound as white solid (34.3 mg, 55% yield). Ratio of isomers was determined to be >20:1 from both the crude reaction mixture and purified products using ^{1}H NMR spectroscopy.

^{1}H NMR (400 MHz, $CDCl_3$) δ 7.84 (d, *J* = 8.2 Hz, 1H), 7.25 (dd, *J* = 8.2, 2.1 Hz, 1H), 7.15 (d, *J* = 2.1 Hz, 1H), 3.56 (s, 3H), 3.54 (s, 3H), 2.84 – 2.78 (m, 1H), 2.74 (dd, *J* = 14.1, 4.8 Hz, 1H), 2.63 (dd, *J* = 14.1, 3.5 Hz, 1H), 1.64 – 1.53 (m, 2H), 1.39 – 1.20 (m, 4H), 0.89 (t, *J* = 6.9 Hz, 3H).

^{13}C NMR (101 MHz, $CDCl_3$) δ 175.11, 146.48, 136.14, 133.18, 127.37, 126.71, 126.66, 71.46, 42.65, 42.42, 39.06, 33.82, 29.68, 24.14, 22.78, 14.08.

HRMS (ESI-TOF) $[M+H]^+$ calculated for $[C_{16}H_{22}ClO_2S]^+$ m/z: 313.1024, found 313.1031.

4-butyl-5-chloro-2-(dimethyl(oxo)-λ^6-sulfanylidene)-3,4-dihydronaphthalen-1(2*H*)-one and 4-butyl-7-chloro-2-(dimethyl(oxo)-λ^6-sulfaneylidene)-3,4-dihydronaphthalen-1(2*H*)-one (5aa)

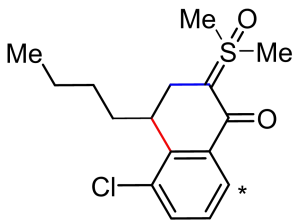

This compound was synthesized following the *general procedure C* using reagent **3i** (115.6 mg, 0.2 mmol), and hex-1-ene (63.2 mg, 0.8 mmol). Purification by flash chromatography on silica gel (DCM/ MeOH: 30/1) provided the title compound as a mixture of isomers (white solid, 28.2 mg, 45% yield). Ratio of isomers was determined to be 2:1 from both the crude reaction mixture and purified products using ^{1}H NMR spectroscopy. Further purification of mixture by carefully column chromatography on silica gel (DCM/ MeOH: 30/1) afforded major product as pure white solid.

Major isomer **^{1}H NMR** (400 MHz, $CDCl_3$) δ 7.84 (dd, *J* = 7.7, 1.4 Hz, 1H), 7.41 (dd, *J* = 7.9, 1.3 Hz, 1H), 7.22 (t, *J* = 7.8 Hz, 1H), 3.59 (s, 3H), 3.55 (s, 3H), 3.40 – 3.35 (m, 1H), 2.79 (dd, *J* = 14.6, 2.1 Hz, 1H), 2.65 (dd, *J* = 14.6, 4.8 Hz, 1H), 1.70 – 1.55 (m, 1H), 1.52 – 1.26 (m, 5H), 0.91 (t, *J* = 6.9 Hz, 3H).

^{13}C NMR (101 MHz, $CDCl_3$) δ 175.13, 142.57, 136.65, 132.57, 131.62, 127.15, 123.80, 71.18, 42.49, 42.40, 35.29, 31.54, 29.79, 22.67, 22.22, 14.12.

HRMS (ESI-TOF) $[M+H]^+$ calculated for $[C_{16}H_{22}ClO_2S]^+$ m/z: 313.1024, found 313.1024.

4-butyl-2-(dimethyl(oxo)-λ^6-sulfanylidene)-6,7-dimethyl-3,4-dihydronaphthalen-1(2*H*)-one and

4-butyl-2-(dimethyl(oxo)-λ[6]-sulfanylidene)-7,8-dimethyl-3,4-dihydronaphthalen-1(2*H*)-one (5ab)

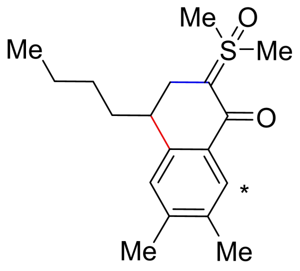

This compound was synthesized following the *general procedure C* using reagent **3j** (114.4 mg, 0.2 mmol), and hex-1-ene (67.2 mg, 0.8 mmol). Purification by flash chromatography on silica gel (DCM/ MeOH: 30/1) provided the title compound as a mixture of isomers (white solid, 50.2 mg, 82% yield). Ratio of isomers was determined to be 1:1.3 from both the crude reaction mixture and purified products using ^{1}H NMR spectroscopy.

^{1}H NMR (400 MHz, $CDCl_3$) δ 7.72 (d, *J* = 7.8 Hz, 1.3*1H), 7.69 (s, 1H), 7.11 (d, *J* = 7.8 Hz, 1.3*1H), 6.94 (s, 1H), 3.58 (s, 3.9*3H), 3.54 – 3.53 (m, 3.9*3H, 3H), 3.18 – 3.10 (m, 1.3*1H), 2.82 – 2.71 (m, 1.3*1H, 2H), 2.65 – 2.59 (m, 1.3*1H, 1H), 2.33 (s, 4.1*3H), 2.29 (s, 3H), 2.28 (s, 3H), 2.25 (s, 4.1*3H), 1.67 – 1.53 (m, 1.3*1H, 2H), 1.42 – 1.24 (m, 6.5*5H, 4H), 0.93 – 0.88 (m, 3.9*3H, 3H).

^{13}C NMR (101 MHz, $CDCl_3$) δ 177.05, 176.65, 143.43, 142.29, 139.90, 139.24, 134.63, 132.63, 128.75, 127.74, 126.03, 122.64, 70.57, 69.47, 42.74, 42.67, 42.51, 38.55, 34.95, 34.11, 32.00, 30.03, 29.79, 24.31, 22.80, 22.74, 22.57, 21.27, 19.87, 19.34, 14.82, 14.08, 14.05.

HRMS (ESI-TOF) $[M+H]^+$ calculated for $[C_{18}H_{27}O_2S]^+$ m/z: 307.1726, found 307.1729.

5-(dimethyl(oxo)-λ[6]-sulfanylidene)-2-methyl-5a,6,7,8,9,9a-hexahydro-6,9-methanonaphtho[2,1-b]thiophen-4(5*H*)-one (5ac)

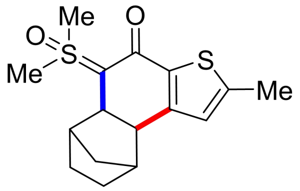

This compound was synthesized following the *general procedure C* using reagent **3k** (112.8 mg, 0.2 mmol), and bicyclo[2.2.1]hept-2-ene (75.2 mg, 0.8 mmol). Purification by flash chromatography on silica gel (DCM/ MeOH: 30/1) provided the title compound as white solid (53.0 mg, 86% yield).

^{1}H NMR (400 MHz, $CDCl_3$) δ 6.53 (s, 1H), 3.56 (s, 3H), 3.53 (s, 3H), 3.13 (d, J = 10.1 Hz, 1H), 3.02 (d, J = 10.0 Hz, 1H), 2.46 (s, 3H), 2.27 (d, J = 3.6 Hz, 1H), 2.11 (d, J = 1.9 Hz, 1H), 1.71 – 1.61 (m, 2H), 1.56 – 1.38 (m, 3H), 1.16 (dt, J = 10.0, 1.6 Hz, 1H).

^{13}C NMR (101 MHz, $CDCl_3$) δ 171.41, 145.84, 143.71, 135.39, 126.16, 48.11, 45.67, 45.49, 44.96, 42.98, 42.71, 33.30, 29.38, 29.14, 16.00.

HRMS (ESI-TOF) $[M+Na]^+$ calculated for $[C_{16}H_{20}NaO_2S]^+$ m/z: 331.0797, found 331.0795.

6-(dimethyl(oxo)-λ^6-sulfanylidene)-4-propyl-5,6-dihydrobenzo[b]thiophen-7(4*H*)-one (5ad)

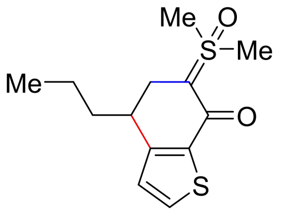

This compound was synthesized following the *general procedure C* using reagent **3l** (110.0 mg, 0.2 mmol), and pent-1-ene (56.0 mg, 0.8 mmol). Purification by flash chromatography on silica gel (DCM/ MeOH: 30/1) provided the title compound as white solid (27.0 mg, 50% yield). Ratio of isomers was determined to be >20:1 from both the crude reaction mixture and purified products using ^{1}H NMR spectroscopy.

^{1}H NMR (400 MHz, $CDCl_3$) δ 7.34 (d, J = 4.9 Hz, 1H), 6.93 (d, J = 4.9 Hz, 1H), 3.53 (s, 3H), 3.51 (s, 3H), 2.93 – 2.88 (m, 1H), 2.83 (dd, J = 13.8, 5.7 Hz, 1H), 2.53 (dd, J = 13.8, 6.7 Hz, 1H), 1.75 – 1.67 (m, 1H), 1.58 – 1.47 (m, 1H), 1.46 – 1.38 (m, 1H), 1.37 – 1.28 (m, 1H), 0.92 (t, J = 7.3 Hz, 3H).

^{13}C NMR (101 MHz, $CDCl_3$) δ 173.00, 149.03, 138.67, 128.12, 126.92, 69.04, 43.19, 42.79, 36.06, 35.82, 26.50, 20.49, 14.28.

HRMS (ESI-TOF) [M] calculated for $[C_{13}H_{18}O_2S]$ m/z: 270.0748, found 270.0682.

1-butyl-3-(dimethyl(oxo)-λ^6-sulfanylidene)-2,3-dihydrodibenzo[b,d]thiophen-4(1H)-one (5ae)

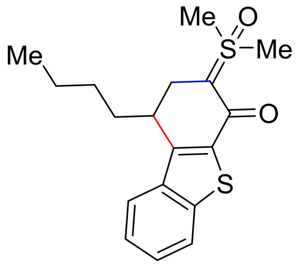

This compound was synthesized following the *general procedure C* using reagent **3m** (120.0 mg, 0.2 mmol), and hex-1-ene (67.2 mg, 0.8 mmol). Purification by flash chromatography on silica gel (DCM/ MeOH: 30/1) provided the title compound as white solid (40.33 mg, 60% yield). Ratio of isomers was determined to be >20:1 from both the crude reaction mixture and purified products using ^{1}H NMR spectroscopy.

^{1}H NMR (400 MHz, $CDCl_3$) δ 7.93 – 7.85 (m, 1H), 7.79 – 7.70 (m, 1H), 7.45 – 7.34 (m, 2H), 3.61 (s, 6H), 3.55 (s, 6H), 3.27 – 3.21 (m, 1H), 2.95 – 2.84 (m, 2H), 1.74 – 1.67 (m, 1H), 1.62 – 1.54 (m, 1H), 1.52 – 1.42 (m, 1H), 1.40 – 1.29 (m, 3H), 0.90 (t, *J* = 7.0 Hz, 3H).

^{13}C NMR (101 MHz, $CDCl_3$) δ 173.16, 143.73, 141.38, 138.63, 138.42, 125.69, 124.27, 123.51, 122.74, 69.27, 43.10, 42.76, 33.95, 31.88, 30.09, 25.01, 22.87, 14.10.

HRMS (ESI-TOF) $[M+H]^+$ calculated for $[C_{18}H_{23}O_2S_2]^+$ m/z: 335.1134, found 335.1123.

5-(dimethyl(oxo)-λ^6-sulfanylidene)-5a,6,7,8,9,9a-hexahydro-6,9-methanonaphtho[2,1-*b*]furan-4(5*H*)-one (5af)

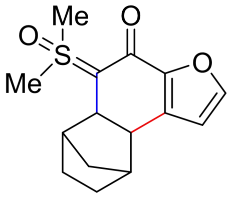

This compound was synthesized following the *general procedure C* using reagent **3n** (106.8 mg, 0.2 mmol), and bicyclo[2.2.1]hept-2-ene (75.2 mg, 0.8 mmol). Purification by flash chromatography on silica gel (DCM/ MeOH: 30/1) provided the title compound as white solid (33.4 mg, 60% yield).

^{1}H NMR (400 MHz, $CDCl_3$) δ 7.37 (d, *J* = 1.8 Hz, 1H), 6.24 (d, *J* = 1.8 Hz, 1H), 3.55 (s, 3H), 3.53 (s, 3H), 3.13 (d, *J* = 10.1 Hz, 1H), 3.01 (dd, *J* = 10.1, 1.7 Hz, 1H), 2.27 – 2.21 (m, 1H), 2.10 – 2.08 (m, 1H), 1.72 – 1.63 (m, 2H), 1.56 – 1.37 (m, 3H), 1.19 (dt, *J* = 10.0, 1.6 Hz, 1H).

^{13}C NMR (101 MHz, $CDCl_3$) δ 167.82, 147.43, 144.27, 129.80, 110.32, 48.15, 45.64, 44.42, 43.22, 42.66, 42.36, 33.41, 29.33, 29.25.

HRMS (ESI-TOF) $[M+H]^+$ calculated for $[C_{15}H_{19}O_3S]^+$ m/z: 279.1049, found 279.1046.

2.3 Light-mediated Tandem [3+2] Cycloaddition: reaction scope.

General Procedure D

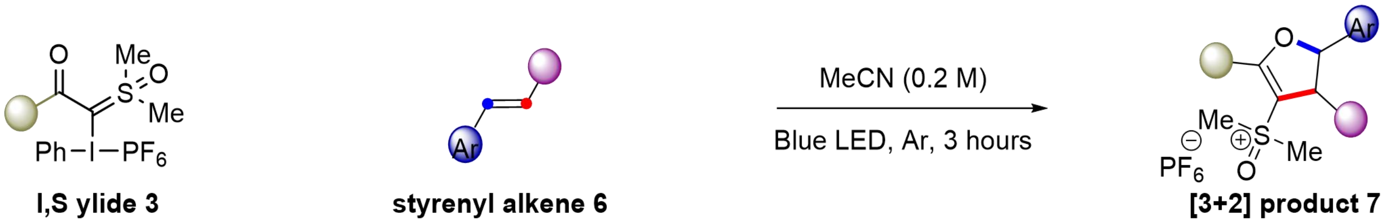

To a 10 mL oven-dried tube equipped with a stirring bar was added reagent **3** (0.2 mmol, 1.0 equiv.), alkene **6** (0.4 mmol, 2.0 equiv.), anhydrous MeCN (1.0 mL). The reaction vial was capped with a rubber septum under an argon atmosphere, and it was fixed on a blue LED light reaction equipment (1 W, 452 nm) for 3 hours. Solvent was removed under *vacuum* and the crude mixture was purified by flash column chromatography to yield the corresponding products **7**.

4-(dimethylethylsulfoxonium)-2,2,5-triphenyl-2,3-dihydrofuran hexafluorophosphate (7a)

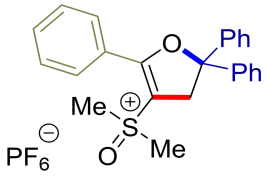

This compound was synthesized following the *general procedure D* using reagent **3c** (108.8 mg, 0.2 mmol), and ethene-1,1-diyldibenzene (72.0 mg, 0.4 mmol). Purification by flash chromatography on silica gel (DCM/ Acetone: 4/1) provided the title compound as white solid (98.8 mg, 95% yield).

^{1}H NMR (400 MHz, Acetone-d_6) δ 7.97 – 7.90 (m, 2H), 7.75 (tt, *J* = 6.6, 1.3 Hz, 1H), 7.68 – 7.58 (m, 6H), 7.51 – 7.44 (m, 4H), 7.40 (tt, *J* = 6.3, 1.4 Hz, 1H), 4.49 (s, 2H), 4.13 (s, 6H).

^{13}C NMR (101 MHz, Acetone-d_6) δ 171.02, 143.10, 133.01, 129.53, 128.91, 128.82, 128.48, 126.94, 125.57, 99.84, 95.07, 44.14, 40.87.

^{19}F NMR (376 MHz, Acetone-d_6) δ -72.20 (d, *J* = 707 Hz).

^{31}P NMR (162 MHz, Acetone-d_6) δ -144.21 (h, *J* = 708 Hz).

HRMS (ESI-TOF) $[M\text{-}PF_6]^+$ calculated for $[C_{24}H_{23}O_2S]^+$ m/z: 375.1413, found 375.1417.

5-(4-chlorophenyl)-4-(dimethylethylsulfoxonium)-2,2-diphenyl-2,3-dihydrofuran hexafluorophosphate (7b)

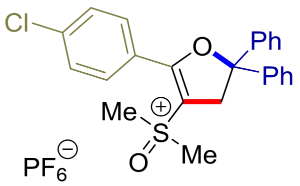

This compound was synthesized following the *general procedure D* using reagent **3h** (115.6 mg, 0.2 mmol), and ethene-1,1-diyldibenzene (72.0 mg, 0.4 mmol). Purification by flash chromatography on silica gel (DCM/ Acetone: 4/1) provided the title compound as white solid (95.2 mg, 86% yield).

^{1}H NMR (400 MHz, Acetone-*d*6) δ 8.00 – 7.94 (m, 2H), 7.71 – 7.65 (m, 2H), 7.62 – 7.57 (m, 4H), 7.50 – 7.44 (m, 4H), 7.44 – 7.38 (m, 2H), 4.45 (s, 2H), 4.15 (s, 6H).

^{13}C NMR (101 MHz, Acetone-*d*6) δ 169.84, 142.99, 138.59, 131.46, 129.01, 128.84, 128.53, 125.58, 125.50, 99.78, 95.17, 44.17, 40.90.

^{19}F NMR (376 MHz, Acetone-*d*6) δ -72.48 (d, *J* = 707 Hz).

^{31}P NMR (162 MHz, Acetone-*d*6) δ -144.22 (h, *J* = 708 Hz).

HRMS (ESI-TOF) $[M-PF_6]^+$ calculated for $[C_{24}H_{22}ClO_2S]^+$ m/z: 409.1024, found 409.1022.

5-([1,1'-biphenyl]-4-yl)-4-(dimethylethylsulfoxonium)-2,2-diphenyl-2,3-dihydrofuran hexafluorophosp-hate (7c)

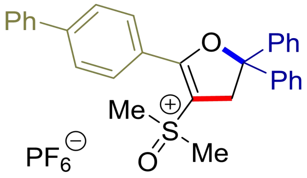

This compound was synthesized following the *general procedure D* using reagent **3o** (124.0 mg, 0.2 mmol), and ethene-1,1-diyldibenzene (72.0 mg, 0.4 mmol). Purification by flash chromatography on silica gel (DCM/ Acetone: 4/1) provided the title compound as white solid (93.0 mg, 78% yield).

^{1}H NMR (400 MHz, DMSO-*d*6) δ 8.00 – 7.89 (m, 4H), 7.82 – 7.75 (m, 2H), 7.58 – 7.52 (m, 6H), 7.50 – 7.44 (m, 5H), 7.44 – 7.37 (m, 2H), 4.28 (s, 2H), 4.04 (s, 6H).

^{13}C NMR (101 MHz, DMSO-*d*6) δ 169.00, 144.74, 143.53, 139.09, 130.58, 129.65, 129.32, 129.05, 128.87, 127.49, 127.37, 125.81, 125.76, 100.19, 94.37, 44.22, 40.92.

^{19}**F NMR** (376 MHz, DMSO-d_6) δ -70.11 (d, J = 710 Hz).

^{31}**P NMR** (162 MHz, DMSO-d_6) δ -144.16 (h, J = 712 Hz).

HRMS (ESI-TOF) $[M{-}PF_6]^+$ calculated for $[C_{30}H_{27}O_2S]^+$ m/z: 451.1726, found 451.1723.

4-(dimethylethylsulfoxonium)-2,2-diphenyl-5-(*o*-tolyl)-2,3-dihydrofuran hexafluorophosphate (7d)

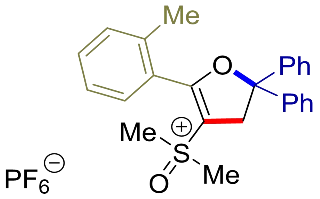

This compound was synthesized following the *general procedure D* using reagent **3p** (111.6 mg, 0.2 mmol), and ethene-1,1-diyldibenzene (72.0 mg, 0.4 mmol). Purification by flash chromatography on silica gel (DCM/ Acetone: 4/1) provided the title compound as white solid (70.5 mg, 66% yield).

^{1}**H NMR** (400 MHz, Acetone-d_6) δ 7.66 – 7.55 (m, 6H), 7.54 – 7.38 (m, 8H), 4.49 (s, 2H), 4.02 (s, 6H), 2.33 (s, 3H).

^{13}**C NMR** (101 MHz, Acetone-d_6) δ 171.59, 143.03, 137.42, 132.08, 131.01, 129.66, 128.82, 128.57, 126.63, 126.19, 125.69, 102.70, 96.13, 43.01, 40.56, 18.76.

^{19}**F NMR** (376 MHz, Acetone-d_6) δ -72.21 (d, J = 707 Hz).

^{31}**P NMR** (162 MHz, Acetone-d_6) δ -144.22 (h, J = 708 Hz).

HRMS (ESI-TOF) $[M{-}PF_6]^+$ calculated for $[C_{25}H_{25}O_2S]^+$ m/z: 389.1570, found 389.1564.

4-(dimethylethylsulfoxonium)-5-(2-phenoxyphenyl)-2,2-diphenyl-2,3-dihydrofuran hexafluorophosphate (7e)

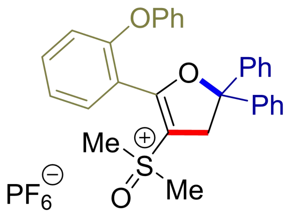

This compound was synthesized following the *general procedure D* using reagent **3q** (127.2 mg, 0.2 mmol), and ethene-1,1-diyldibenzene (72.0 mg, 0.4 mmol). Purification by flash chromatography on silica gel (DCM/ Acetone: 4/1) provided the title compound as white solid (74.6 mg, 61% yield).

^{1}H NMR (400 MHz, Acetone-d_6) δ 7.76 (dd, J = 7.7, 1.7 Hz, 1H), 7.66 – 7.62 (m, 1H), 7.58 – 7.51 (m, 4H), 7.50 – 7.44 (m, 2H), 7.43 – 7.33 (m, 7H), 7.7 (tt, J = 7.4, 1.1 Hz, 1H), 7.15 – 7.09 (m, 2H), 7.02 (d, J = 8.4 Hz, 1H), 4.40 (s, 2H), 4.08 (s, 6H).

^{13}C NMR (101 MHz, Acetone-d_6) δ 167.76, 155.76, 155.65, 143.02, 133.80, 130.92, 130.30, 128.71, 128.42, 125.61, 124.72, 123.37, 119.61, 118.61, 117.96, 101.10, 95.69, 43.66, 40.49.

^{19}F NMR (376 MHz, Acetone-d_6) δ -72.26 (d, J = 707 Hz).

^{31}P NMR (162 MHz, Acetone-d_6) δ -144.22 (h, J = 708 Hz).

HRMS (ESI-TOF) $[M-PF_6]^+$ calculated for $[C_{30}H_{27}O_3S]^+$ m/z: 467.1675, found 467.1692.

5-(3,4-dimethylphenyl)-4-(dimethylethylsulfoxonium)-2-phenyl-2,3-dihydrofuran hexafluorophosphate (7f)

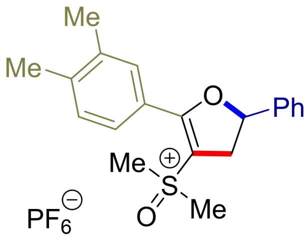

This compound was synthesized following the *general procedure D* using reagent **3j** (114.4 mg, 0.2 mmol), and styrene (41.6 mg, 0.4 mmol). Purification by flash chromatography on silica gel (DCM/ Acetone: 4/1) provided the title compound as white solid (86.8 mg, 92% yield).

^{1}H NMR (400 MHz, Acetone-d_6) δ 7.63 – 7.58 (m, 4H), 7.54 – 7.43 (m, 3H), 7.37 (d, J = 7.7 Hz, 1H), 6.28 (t, J = 10.0 Hz, 1H), 4.09 (dd, J = 13.6, 10.6 Hz, 1H), 4.02 (s, 3H), 3.98 (s, 3H), 3.68 – 3.62 (m, 1H), 2.36 (s, 3H), 2.34 (s, 3H).

^{13}C NMR (101 MHz, Acetone-d_6) δ 173.28, 142.67, 139.02, 137.51, 130.14, 129.92, 129.17, 128.99, 127.14, 126.40, 124.57, 98.69, 85.83, 40.92, 40.59, 38.78, 19.12, 18.80.

^{19}F NMR (376 MHz, Acetone-d_6) δ -72.05 (d, J = 707 Hz).

^{31}P NMR (162 MHz, Acetone-d_6) δ -144.21 (h, J = 708 Hz).

HRMS (ESI-TOF) $[M-PF_6]^+$ calculated for $[C_{20}H_{23}O_2S]^+$ m/z: 327.1413, found 327.1412.

4-(dimethylethylsulfoxonium)-5-(4-Cyanophenyl)-2,2-diphenyl-2,3-dihydrofuran hexafluorophosphate (7g)

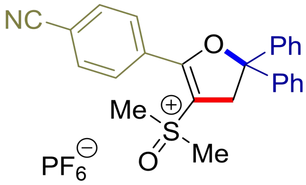

This compound was synthesized following the *general procedure D* using reagent **3r** (113.8 mg, 0.2 mmol), and ethene-1,1-diyldibenzene (72.0 mg, 0.4 mmol). Purification by flash chromatography on silica gel (DCM/ Acetone: 4/1) provided the title compound as white solid (30.6 mg, 28% yield).

^{1}**H NMR** (400 MHz, Acetone-d_6) δ 8.12 (d, J = 8.5 Hz, 2H), 8.03 (d, J = 8.5 Hz, 2H), 7.62 – 7.56 (m, 4H), 7.50 – 7.45 (m, 4H), 7.44 – 7.39 (m, 2H), 4.49 (s, 2H), 4.20 (s, 6H).

^{13}**C NMR** (101 MHz, Acetone-d_6) δ 168.93, 142.87, 132.35, 131.05, 130.65, 128.86, 128.59, 125.52, 117.53, 115.88, 100.96, 95.70, 44.12, 40.93.

^{19}**F NMR** (376 MHz, Acetone-d_6) δ -72.27 (d, J = 707 Hz).

^{31}**P NMR** (162 MHz, Acetone-d_6) δ -144.23 (h, J = 708 Hz).

HRMS (ESI-TOF) $[M-PF_6]^+$ calculated for $[C_{25}H_{22N}O_2S]^+$ m/z: 400.1366, found 400.1362.

4-(dimethylethylsulfoxonium)-5-(naphthalen-1-yl)-2,2-diphenyl-2,3-dihydrofuran tetrafluoroborate (7h)

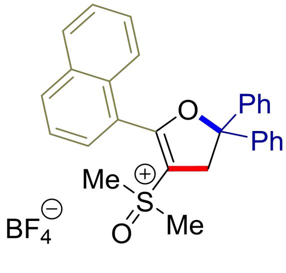

This compound was synthesized following the *general procedure D* using reagent **3s** (107.2 mg, 0.2 mmol), and ethene-1,1-diyldibenzene (72.0 mg, 0.4 mmol). Purification by flash chromatography on silica gel (DCM/ MeOH: 30/1) provided the title compound as white solid (51.2 mg, 50% yield).

^{1}H NMR (400 MHz, CD_3CN) δ 8.20 (d, *J* = 8.3 Hz, 1H), 8.06 (d, *J* = 8.2 Hz, 1H), 7.80 (dd, *J* = 7.1, 1.2 Hz, 1H), 7.74 (d, *J* = 8.4 Hz, 1H), 7.70 – 7.63 (m, 2H), 7.61 – 7.54 (m, 5H), 7.53 – 7.48 (m, 4H), 7.47 – 7.42 (m, 2H), 4.37 (s, 2H), 3.66 (s, 6H).

^{13}C NMR (101 MHz, CD_3CN) δ 170.88, 142.86, 133.35, 132.51, 130.30, 128.96, 128.88, 128.82, 128.71, 127.88, 127.25, 125.60, 125.14, 124.33, 123.99, 103.34, 96.45, 42.90, 40.88.

^{19}F NMR (376 MHz, CD_3CN) δ -151.33.

HRMS (ESI-TOF) $[M-BF_4]^+$ calculated for $[C_{28}H_{25}O_2S]^+$ m/z: 425.1570, found 425.1567.

4-(dimethylethylsulfoxonium)-2-phenyl-5-(thiophen-2-yl)-2,3-dihydrofuran hexafluorophosphate (7i)

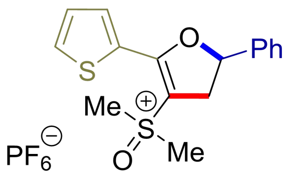

This compound was synthesized following the *general procedure D* using reagent **3l** (110.0 mg, 0.2 mmol), and ethene-1,1-diyldibenzene (72.0 mg, 0.4 mmol). Purification by flash chromatography on silica gel (DCM/ MeOH: 30/1) provided the title compound as white solid (80.1 mg, 89% yield).

^{1}H NMR (400 MHz, Acetone-d_6) δ 8.17 (dd, *J* = 3.9, 1.1 Hz, 1H), 8.09 (dd, *J* = 5.0, 1.1 Hz, 1H), 7.61 – 7.57 (m, 2H), 7.53 – 7.44 (m, 3H), 7.37 (dd, *J* = 5.0, 3.9 Hz, 1H), 6.26 (dd, *J* = 10.4, 9.3 Hz, 1H), 4.20 – 4.18 (s, 3H), 4.17 (s, 3H), 4.15 (dd, *J* = 13.7, 10.4 Hz, 1H), 3.73 (dd, *J* = 13.7, 9.3 Hz, 1H).

^{13}C NMR (101 MHz, Acetone-d_6) δ 165.15, 138.74, 135.37, 134.49, 129.24, 128.99, 128.86, 127.57, 126.34, 96.02, 85.46, 40.86, 40.47, 39.40.

^{19}F NMR (376 MHz, Acetone-d_6) δ -72.28 (d, *J* = 707 Hz).

^{31}P NMR (162 MHz, Acetone-d_6) δ -144.25 (h, *J* = 708 Hz).

HRMS (ESI-TOF) $[M-PF_6]^+$ calculated for $[C_{16}H_{17}O_2S_2]^+$ m/z: 305.0664, found 305.0662.

3-(dimethylethylsulfoxonium)-5,5-diphenyl-4,5-dihydro-2,2'-bifuran hexafluorophosphate (7j)

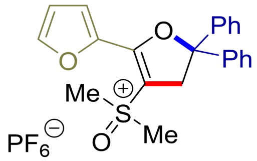

This compound was synthesized following the *general procedure D* using reagent **3n** (106.8 mg, 0.2 mmol), and ethene-1,1-diyldibenzene (72.0 mg, 0.4 mmol). Purification by flash chromatography on silica gel (DCM/ Acetone: 4/1) provided the title compound as white solid (77.6 mg, 76% yield).

^{1}H NMR (400 MHz, $CDCl_3$) δ 7.88 (d, *J* = 1.8 Hz, 1H), 7.45 – 7.41 (m, 4H), 7.40 – 7.35 (m, 5H), 7.34 – 7.29 (m, 2H), 6.68 (dd, *J* = 3.7, 1.8 Hz, 1H), 4.09 (s, 2H), 3.80 (s, 6H).

^{13}C NMR (101 MHz, $CDCl_3$) δ 157.92, 149.49, 141.67, 141.31, 128.89, 128.76, 125.71, 120.13, 113.25, 95.58, 95.55, 43.92, 42.67.

^{19}F NMR (376 MHz, $CDCl_3$) δ -70.53 (d, *J* = 712 Hz).

^{31}P NMR (162 MHz, $CDCl_3$) δ -144.17 (h, *J* = 713 Hz).

HRMS (ESI-TOF) $[M-PF_6]^+$ calculated for $[C_{22}H_{21}O_3S]^+$ m/z: 365.1206, found 365.1198.

5-benzyl-4-(dimethylethylsulfoxonium)-2,2-diphenyl-2,3-dihydrofuran hexafluorophosphate (7k)

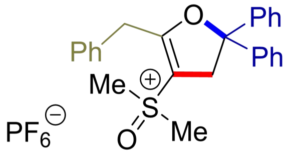

This compound was synthesized following the *general procedure D* using reagent **3t** (111.6 mg, 0.2 mmol), and ethene-1,1-diyldibenzene (72.0 mg, 0.4 mmol). Purification by flash chromatography on silica gel (DCM/ MeOH: 30/1) provided the title compound as white solid (53.4 mg, 50% yield).

^{1}H NMR (400 MHz, CD_3CN) δ 7.41 – 7.39 (m, 4H), 7.39 – 7.34 (m, 7H), 7.33 – 7.30 (m, 4H), 4.12 (s, 2H), 3.91 (s, 2H), 3.71 (s, 6H).

^{13}C NMR (101 MHz, CD_3CN) δ 174.69, 142.76, 133.87, 129.47, 128.86, 128.70, 128.46, 127.65, 125.29, 96.36, 95.14, 42.86, 41.47, 33.75.

^{19}F NMR (376 MHz, CD_3CN) δ -72.78 (d, *J* = 705 Hz).

^{31}P NMR (162 MHz, CD_3CN) δ -144.59 (h, *J* = 707 Hz).

HRMS (ESI-TOF) $[M-PF_6]^+$ calculated for $[C_{25}H_{25}O_2S]^+$ m/z: 389.1570, found 389.1570.

2-methyl-4-(dimethylethylsulfoxonium)-2,5-diphenyl-2,3-dihydrofuran hexafluorophosphate (7l)

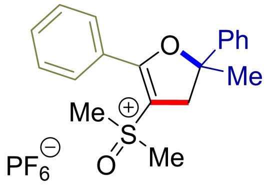

This compound was synthesized following the *general procedure D* using reagent **3c** (108.8 mg, 0.2 mmol), and prop-1-en-2-ylbenzene (47.2 mg, 0.4 mmol). Purification by flash chromatography on silica gel (DCM/ Acetone: 4/1) provided the title compound as white solid (69.6 mg, 76% yield).

^{1}H NMR (400 MHz, Acetone-*d*$_6$) δ 7.92 – 7.88 (m, 2H), 7.74 (tt, *J* = 6.6, 1.3 Hz, 1H), 7.67 – 7.61 (m, 2H), 7.60 – 7.56 (m, 2H), 7.52 – 7.46 (m, 2H), 7.44 – 7.38 (m, 1H), 4.06 (s, 3H), 4.03 (s, 3H), 3.90 (d, *J* = 3.0 Hz, 2H), 2.01 (s, 3H).

^{13}C NMR (101 MHz, Acetone-*d*$_6$) δ 171.50, 143.93, 132.85, 129.45, 128.82, 128.81, 128.20, 127.29, 124.34, 99.07, 92.65, 44.46, 40.88, 40.73.

^{19}F NMR (376 MHz, Acetone-*d*$_6$) δ -72.25 (d, *J* = 707 Hz).

^{31}P NMR (162 MHz, Acetone-*d*$_6$) δ -144.24 (h, *J* = 708 Hz).

HRMS (ESI-TOF) $[M{-}PF_6]^+$ calculated for $[C_{19}H_{21}O_2S]^+$ m/z: 313.1257, found 313.1256.

4-(dimethylethylsulfoxonium)-5-phenyl-3',4'-dihydro-2'*H*,3*H*-spiro[furan-2,1'-naphthalene] hexafluor–ophosphate (7m)

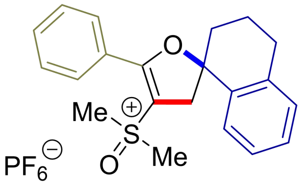

This compound was synthesized following the *general procedure D* using reagent **3c** (108.8 mg, 0.2 mmol), and 1-methylene-1,2,3,4-tetrahydronaphthalene (57.6 mg, 0.4 mmol). Purification by flash chromatography on silica gel (DCM/ Acetone: 4/1) provided the title compound as white solid (58.0 mg, 60% yield).

^{1}H NMR (400 MHz, $CDCl_3$) δ 7.74 (dd, *J* = 7.1, 1.7 Hz, 2H), 7.66 – 7.59 (m, 1H), 7.57 – 7.48 (m, 3H), 7.31 (td, *J* = 7.5, 1.5 Hz, 1H), 7.26 (td, *J* = 7.4, 1.4 Hz, 1H), 7.13 (d, *J* = 7.6 Hz, 1H), 3.67 – 3.64 (m, 1H), 3.57 (s, 3H), 3.56 – 3.54 (m, 1H), 3.51 (s, 3H), 2.91 – 2.78 (m, 2H), 2.41 – 2.28 (m, 2H), 2.09 – 1.99 (m, 1H), 1.98 – 1.86 (m, 1H).

^{13}C NMR (101 MHz, $CDCl_3$) δ 172.79, 137.37, 136.57, 133.30, 129.21, 129.13, 129.11, 127.35, 126.96, 126.58, 98.50, 92.33, 44.37, 40.85, 40.55, 35.79, 29.06, 19.36.

^{19}F NMR (376 MHz, $CDCl_3$) δ -70.57 (d, *J* = 712 Hz).

^{31}P NMR (162 MHz, $CDCl_3$) δ -144.25 (h, *J* = 713 Hz).

HRMS (ESI-TOF) $[M-PF_6]^+$ calculated for $[C_{21}H_{23}O_2S]^+$ m/z: 339.1413, found 339.1409.

4'-(dimethylethylsulfoxonium)-5'-phenyl-10,11-dihydro-3'*H*-spiro[dibenzo[*a,d*][7]annulene-5,2'-furan] hexafluorophosphate (7n)

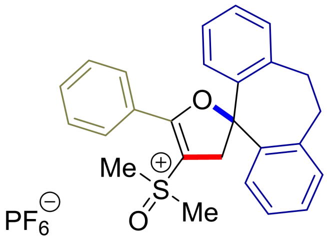

This compound was synthesized following the *general procedure D* using reagent **3c** (108.8 mg, 0.2 mmol), and 5-methylene-10,11-dihydro-5H-dibenzo[*a,d*][7]annulene (41.2 mg, 0.2 mmol). Purification by flash chromatography on silica gel (DCM/ Acetone: 4/1) provided the title compound as white solid (41.5 mg, 76% yield).

^{1}H NMR (400 MHz, $CDCl_3$) δ 7.98 – 7.89 (m, 2H), 7.74 (tt, *J* = 6.2 , 1.3 Hz 1H), 7.68 – 7.63 (m, 2H), 7.54 – 7.50 (m, 2H), 7.23 – 7.19 (m, 6H), 3.94 (s, 2H), 3.59 – 3.47 (m, 2H), 3.39 (s, 6H), 3.08 – 2.92 (m, 2H).

^{13}C NMR (101 MHz, $CDCl_3$) δ 171.68, 140.31, 137.24, 133.53, 131.50, 129.47, 129.36, 128.68, 126.59, 126.26, 123.77, 98.96, 94.36, 48.37, 40.80, 32.40.

^{19}F NMR (376 MHz, $CDCl_3$) δ -70.63 (d, *J* = 712 Hz).

^{31}P NMR (162 MHz, $CDCl_3$) δ -144.43 (h, *J* = 713 Hz).

HRMS (ESI-TOF) $[M-PF_6]^+$ calculated for $[C_{26}H_{25}O_2S]^+$ m/z: 401.1570, found 401.1569.

4-(dimethylethylsulfoxonium)-2,3,5-triphenyl-2,3-dihydrofuran hexafluorophosphate (7o)

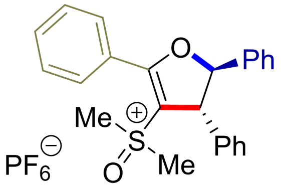

This compound was synthesized following the *general procedure D* using reagent **3c** (108.8 mg, 0.2 mmol), and (*E*)-1,2-Diphenylethene (72.0 mg, 0.4 mmol). Purification by flash chromatography on silica gel (DCM/ Acetone: 4/1) provided the title compound as white solid (73.8 mg, 71% yield).

Note: This compound was also synthesized following the *general procedure D* using (*Z*)-1,2-Diphenylethene (72.0 mg, 0.4 mmol). Purification by flash chromatography on silica gel (DCM/ Acetone: 4/1) provided the identical title compound as white solid (67.5 mg, 65% yield).

^{1}H NMR (400 MHz, Acetone-d_6) δ 8.08 – 8.01 (m, 2H), 7.82 (tt, *J* = 6.7, 1.4 Hz, 1H), 7.76 – 7.67 (m, 4H), 7.62 – 7.57 (m, 4H), 7.55 – 7.50 (m, 4H), 6.06 (d, *J* = 6.9 Hz, 1H), 5.14 (d, *J* = 6.9 Hz, 1H), 3.87 (s, 3H), 3.71 (s, 3H).

^{13}C NMR (101 MHz, Acetone-d_6) δ 174.50, 139.23, 138.10, 133.50, 130.08, 129.75, 129.58, 129.23, 129.11, 128.16, 126.99, 126.09, 103.32, 94.45, 58.05, 41.29, 41.24.

^{19}F NMR (376 MHz, Acetone-d_6) δ -72.42 (d, *J* = 707 Hz).

^{31}P NMR (162 MHz, Acetone-d_6) δ -144.25 (h, *J* = 708 Hz).

HRMS (ESI-TOF) $[M-PF_6]^+$ calculated for $[C_{24}H_{23}O_2S]^+$ m/z: 375.1413, found 375.1422.

3-methyl-4-(dimethylethylsulfoxonium)-2,5-diphenyl-2,3-dihydrofuran hexafluorophosphate (7p)

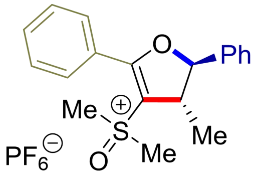

This compound was synthesized following the *general procedure D* using reagent **3c** (108.8 mg, 0.2 mmol), and (*E*)-β-methyl styrene (47.2 mg, 0.4 mmol). Purification by flash chromatography on silica gel (DCM/ MeOH: 30/1) provided the title compound as white solid (77.9 mg, 85% yield).

Note: This compound was also synthesized following the *general procedure D* using (*Z*)-β-methyl styrene (47.2 mg, 0.4 mmol). Purification by flash chromatography on silica gel (DCM/ Acetone: 4/1) provided the identical title compound as white solid (76.1 mg, 83% yield).

^{1}H NMR (400 MHz, Acetone-d_6) δ 7.90 (t, J = 1.4 Hz, 1H), 7.88 (t, J = 1.8 Hz, 1H), 7.77 (tt, J = 6.7, 1.3 Hz, 1H), 7.70 – 7.62 (m, 4H), 7.55 – 7.48 (m, 3H), 5.83 (d, J = 5.9 Hz, 1H), 4.17 (s, 3H), 4.02 – 3.96 (m, 1H), 3.90 (s, 3H), 1.80 (d, J = 6.7 Hz, 3H).

^{13}C NMR (101 MHz, Acetone-d_6) δ 172.88, 138.50, 133.22, 129.61, 129.34, 129.11, 129.06, 127.37, 125.93, 104.86, 92.75, 47.36, 41.78, 41.12, 20.50.

^{19}F NMR (376 MHz, Acetone-d_6) δ -72.43 (d, J = 707 Hz).

^{31}P NMR (162 MHz, Acetone-d_6) δ -144.54 (h, J = 708 Hz).

HRMS (ESI-TOF) $[M-PF_6]^+$ calculated for $[C_{19}H_{21}O_2S]^+$ m/z: 313.1257, found 313.1257.

The crystal structure of **7p** has been deposited at the Cambridge Crystallographic Data Centre, CCDC 2128864.

2-cyclopropyl-4-(dimethylethylsulfoxonium)-2,5-diphenyl-2,3-dihydrofuran hexafluorophosphate (7q)

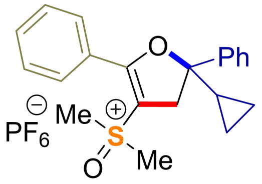

This compound was synthesized following the *general procedure D* using reagent **3c** (108.8 mg, 0.2 mmol), and (1-cyclopropylvinyl)benzene (57.7 mg, 0.4 mmol). Purification by flash chromatography on silica gel (DCM/ Acetone: 4/1) provided the title compound as white solid (79.4 mg, 82% yield).

^{1}H NMR (400 MHz, Chloroform-d) δ 7.72 – 7.68 (m, 2H), 7.64 (d, J = 7.5 Hz, 1H), 7.55 (t, J = 7.6 Hz, 2H), 7.46 – 7.36 (m, 4H), 7.34 (d, J = 7.0 Hz, 1H), 3.75 (d, J = 13.6 Hz, 1H), 3.68 (d, J = 13.6 Hz, 1H), 3.57 (s, 3H), 3.54 (s, 3H), 1.71 – 1.65 (m, 1H), 0.88 (t, J = 6.7 Hz, 1H), 0.75 – 0.71 (m, 1H), 0.65 – 0.61 (m, 1H), 0.48 – 0.44 (m, 2H).

^{13}C NMR (101 MHz, $CDCl_3$) δ 173.07, 141.29, 133.57, 129.38, 129.28, 128.80, 128.64, 126.76, 125.33, 99.13, 95.98, 42.98, 41.35, 40.95, 21.30, 2.06, 1.69.

^{19}F NMR (376 MHz, $CDCl_3$) δ -70.89 (d, J = 710 Hz).

^{31}P NMR (162 MHz, $CDCl_3$) δ -144.28 (h, J = 713 Hz).

HRMS (ESI-TOF) $[M-PF_6]^+$ calculated for $[C_{21}H_{23}O_2S]^+$ m/z: 339.1413, found 339.1420.

2.4 Light-mediated and/or Rh-mediated cycloaddition with nitriles, alkynes and allene: reaction scope.

With nitriles:

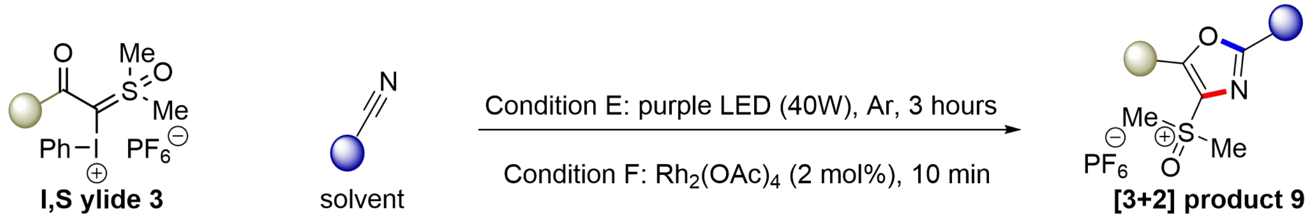

General Procedure E

To a 10 mL oven-dried tube equipped with a stirring bar was added reagent **3** (0.2 mmol, 1.0 equiv.), anhydrous nitriles solvent (1.0 mL). The reaction vial was capped with a rubber septum under an argon atmosphere, and it was fixed on a Purple LED light reaction equipment (40 W, 390 nm) for 3 hours. Solvent was removed under *vacuum* and the crude mixture was purified by flash column chromatography to yield the corresponding products **9**.

General Procedure F

To a 10 mL oven-dried tube equipped with a stirring bar was added reagent **3** (0.2 mmol, 1.0 equiv.), $Rh_2(OAc)_4$ (2.2 mg, 0.02 equiv.), nitriles solvent (0.5 mL). The reaction vial was vigorously stirred in the open air for ten minutes. Solvent was removed under *vacuum* and the crude mixture was purified by flash column chromatography to yield the corresponding products **9**.

2-methyl-4-(dimethylethylsulfoxonium)-5-phenyloxazole hexafluorophosphate (9a)

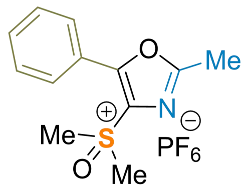

This compound was synthesized following the *general procedure E* using reagent **3c** (108.8 mg, 0.2 mmol), and acetonitrile (1.0 mL). Purification by flash chromatography on silica gel (DCM/ Acetone: 4/1) provided the title compound as white solid (51.1 mg, 67% yield).

Note:This compound was also synthesized following the *general procedure F* using reagent **3c** (108.8 mg, 0.2 mmol), and acetonitrile (0.5 mL). Purification by flash chromatography on silica gel (DCM/ Acetone: 4/1) provided the title compound as white solid (72.4 mg, 95% yield).

^{1}H NMR (400 MHz, Acetone-d_6) δ 7.93 (d, J = 6.9 Hz, 2H), 7.72 – 7.67 (m, 1H), 7.67 – 7.61 (m, 2H), 4.32 (s, 6H), 2.69 (s, 3H).

^{13}C NMR (101 MHz, Acetone-d_6) δ 162.98, 158.93, 132.33, 129.19, 129.08, 124.52, 124.10, 40.71, 40.64, 13.00, 12.94.

^{19}F NMR (376 MHz, Acetone-d_6) δ -71.85 (d, J = 707 Hz).

^{31}P NMR (162 MHz, Acetone-d_6) δ -144.33 (h, J = 708 Hz).

HRMS (ESI-TOF) $[M-PF_6]^+$ calculated for $[C_{12}H_{14}NO_2S]^+$ m/z: 236.0740, found 236.0745.

2-methyl-4-(dimethylethylsulfoxonium)-5-phenethyloxazole hexafluorophosphate (9b)

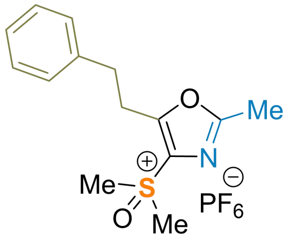

Following the *general procedure E* using reagent **3u** (114.4 mg, 0.2 mmol), and acetonitrile (1.0 mL). According to NMR spectroscopy of crude reaction mixture, trace product was formed.

This compound was synthesized following the *general procedure F* using reagent **3u** (114.4 mg, 0.2 mmol), and acetonitrile (0.5 mL). Purification by flash chromatography on silica gel (DCM/ Acetone: 4/1) provided the title compound as white solid (49.9 mg, 61% yield).

^{1}H NMR (400 MHz, Acetone-d_6) δ 7.36 – 7.32 (m, 2H), 7.29 – 7.20 (m, 3H), 4.07 (s, 3H), 4.06 (s, 3H), 3.40 (t, J = 7.5 Hz, 2H), 3.11 (t, J = 7.5 Hz, 2H), 2.62 (s, 3H).

^{13}C NMR (101 MHz, Acetone-d_6) δ 163.38, 162.43, 139.55, 128.68, 128.63, 126.77, 124.63, 40.52, 33.28, 27.40, 12.94.

^{19}F NMR (376 MHz, Acetone-d_6) δ -71.53 (d, J = 17.9 Hz).

^{31}P NMR (162 MHz, Acetone-d_6) δ -144.30 (h, J = 708 Hz).

HRMS (ESI-TOF) $[M-PF_6]^+$ calculated for $[C_{14}H_{18}NO_2S]^+$ m/z: 264.1053, found 264.1059.

4-(dimethylethylsulfoxonium)-2,5-diphenyloxazole hexafluorophosphate (9c)

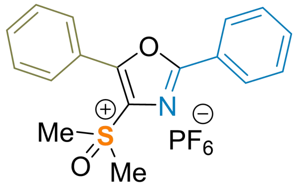

This compound was synthesized following the *general procedure E* using reagent **3c** (108.8 mg, 0.2 mmol), and Benzonitrile (0.5 mL). Purification by flash chromatography on silica gel (DCM/ Acetone: 4/1) provided the title compound as white solid (31.0 mg, 35% yield).

This compound was also synthesized following the *general procedure F* using reagent **3c** (108.8 mg, 0.2 mmol), and Benzonitrile (0.5 mL). Purification by flash chromatography on silica gel (DCM/ Acetone: 4/1) provided the title compound as white solid (80.0 mg, 79% yield).

^{1}H NMR (400 MHz, Acetone-d_6) δ 8.27 – 8.22 (m, 2H), 8.14 – 8.08 (m, 2H), 7.74 – 7.66 (m, 6H), 4.49 (s, 6H).

^{13}C NMR (101 MHz, Acetone-d_6) δ 161.46, 158.75, 132.78, 132.58, 129.46, 129.36, 129.19, 127.17, 126.14, 124.78, 124.04, 40.87.

^{19}F NMR (376 MHz, Acetone-d_6) δ -72.40 (d, J = 707 Hz).

^{31}P NMR (162 MHz, Acetone-d_6) δ -144.25 (h, J = 708 Hz).

HRMS (ESI-TOF) $[M-PF_6]^+$ calculated for $[C_{17}H_{16}NO_2S]^+$ m/z: 298.0896, found 298.0892.

2-(*tert*-butyl)-4-(dimethylethylsulfoxonium)-5-(naphthalen-1-yl)oxazole hexafluorophosphate (9d)

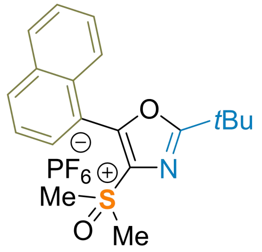

This compound was synthesized following the *general procedure E* using reagent **3s** (118.8 mg, 0.2 mmol), and trimethylacetonitrile (0.5 mL). Purification by flash chromatography on silica gel (DCM/ Acetone: 4/1) provided the title compound as white solid (52.0 mg, 55% yield).

This compound was also synthesized following the *general procedure F* using reagent **3s** (118.8 mg, 0.2 mmol), and trimethylacetonitrile (0.5 mL). Purification by flash chromatography on silica gel (DCM/ Acetone: 4/1) provided the title compound as white solid (89.9 mg, 95% yield).

^{1}**H NMR** (400 MHz, Acetone-d_6) δ 8.29 (d, J = 8.3 Hz, 1H), 8.15 – 8.12 (m, 1H), 8.02 – 8.00 (m, 1H), 7.96 – 7.94 (m, 1H), 7.76 – 7.67 (m, 3H), 4.33 (s, 6H), 1.54 (s, 9H).

^{13}**C NMR** (101 MHz, Acetone-d_6) δ 172.99, 157.96, 133.63, 133.01, 131.47, 131.21, 128.85, 128.14, 127.53, 127.14, 125.06, 124.65, 121.08, 40.44, 34.40, 27.46.

^{19}**F NMR** (376 MHz, Acetone-d_6) δ -72.39 (d, J = 707 Hz).

^{31}**P NMR** (162 MHz, Acetone-d_6) δ -144.27 (h, J = 708 Hz).

HRMS (ESI-TOF) $[M-PF_6]^+$ calculated for $[C_{19}H_{22}NO_2S]^+$ m/z: 328.1366, found 328.1367.

2-(*tert*-butyl)-4-(dimethylethylsulfoxonium)-5-phenyloxazole hexafluorophosphate (9e)

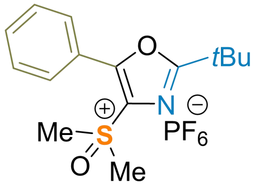

This compound was synthesized following the *general procedure E* using reagent **3c** (108.8 mg, 0.2 mmol), and trimethylacetonitrile (0.5 mL). Purification by flash chromatography on silica gel (DCM/ Acetone: 4/1) provided the title compound as white solid (52.5 mg, 62% yield).

This compound was also synthesized following the *general procedure F* using reagent **3c** (108.8 mg, 0.2 mmol), and Trimethylacetonitrile (0.5 mL). Purification by flash chromatography on silica gel (DCM/ Acetone: 4/1) provided the title compound as white solid (81.2 mg, 96% yield).

^{1}**H NMR** (400 MHz, Acetone-d_6) δ 8.00 – 7.92 (m, 2H), 7.73 – 7.67 (m, 1H), 7.67 – 7.62 (m, 2H), 4.35 (brs, 6H), 1.51 (s, 9H).

^{13}**C NMR** (101 MHz, Acetone-d_6) δ 171.76, 158.66, 132.32, 129.20, 129.10, 124.25, 40.66, 34.21, 27.41.

^{19}**F NMR** (376 MHz, Acetone-d_6) δ -72.29 (d, J = 707 Hz).

^{31}**P NMR** (162 MHz, Acetone-d_6) δ -144.30 (h, J = 708 Hz).

HRMS (ESI-TOF) $[M-PF_6]^+$ calculated for $[C_{15}H_{20}NO_2S]^+$ m/z: 278.1209, found 278.1207.

The crystal structure of **9e** has been deposited at the Cambridge Crystallographic Data Centre, CCDC 2152681.

2-(*tert*-butyl)-5-(furan-2-yl)-4-(dimethylethylsulfoxonium)oxazole hexafluorophosphate (9f)

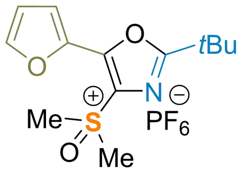

Following the *general procedure E* using reagent **3n** (106.8 mg, 0.2 mmol), and Trimethylacetonitrile (0.5 mL). According to NMR spectroscopy of crude reaction mixture, trace product was formed.

This compound was synthesized following the *general procedure F* using reagent **3n** (106.8 mg, 0.2 mmol), and Trimethylacetonitrile (0.5 mL). Purification by flash chromatography on silica gel (DCM/ Acetone: 4/1) provided the title compound as white solid (49.6 mg, 60% yield).

^{1}H NMR (400 MHz, Acetone-*d*6) δ 8.08 (s, 1H), 7.46 (d, *J* = 3.6 Hz, 1H), 6.91 – 6.83 (m, 1H), 4.43 (s, 3H), 4.42 (s, 3H), 1.49 (s, 9H).

^{13}C NMR (101 MHz, Acetone-*d*6) δ 170.59, 148.68, 148.00, 139.52, 123.17, 116.74, 113.19, 40.44, 34.19, 27.39.

^{19}F NMR (376 MHz, Acetone-*d*6) δ -72.46 (d, *J* = 707 Hz).

^{31}P NMR (162 MHz, Acetone-*d*6) δ -144.30 (h, *J* = 708 Hz).

HRMS (ESI-TOF) $[M-PF_6]^+$ calculated for $[C_{13}H_{18}NO_3S]^+$ m/z: 268.1002, found 268.1003.

With alkynes:

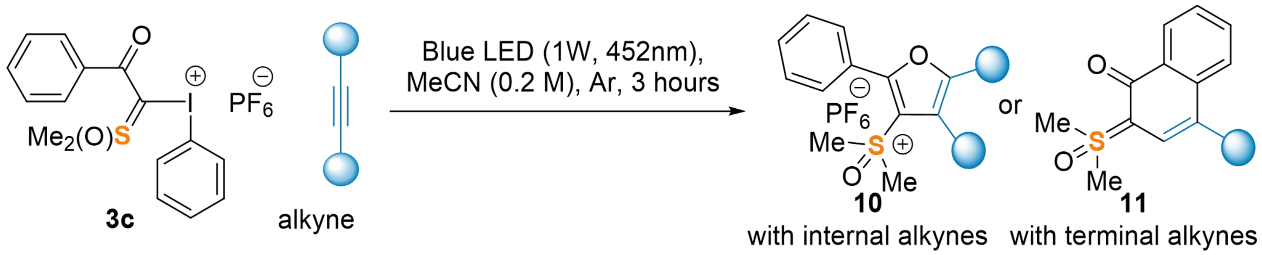

3-methyl-4-(dimethylethylsulfoxonium)-2,5-diphenylfuran hexafluorophosphate (10a)

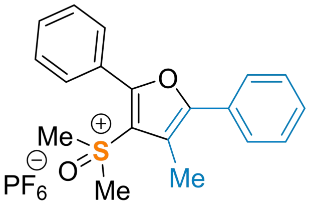

This compound was synthesized following the *general procedure D* using reagent **3c** (108.8 mg, 0.2 mmol), and prop-1-yn-1-ylbenzene (46.4 mg, 0.4 mmol). Purification by flash chromatography on silica gel (DCM/ Acetone: 4/1) provided the title compound as white solid (68.4 mg, 75% yield). Ratio of isomers was determined to be >20:1 from both the crude reaction mixture and purified products using ^{1}H NMR spectroscopy.

^{1}H NMR (400 MHz, Acetone-d_6) δ 7.91 – 7.86 (m, 2H), 7.79 – 7.75 (m, 2H), 7.74 – 7.69 (m, 1H), 7.68 – 7.58 (m, 4H), 7.57 – 7.52 (m, 1H), 4.35 (s, 6H), 2.67 (s, 3H).

^{13}C NMR (101 MHz, Acetone-d_6) δ 159.17, 152.10, 131.82, 130.44, 129.54, 129.12, 128.85, 128.29, 127.28, 114.60, 114.44, 42.82, 10.07.

^{19}F NMR (376 MHz, Acetone-d_6) δ -72.45 (d, J = 706 Hz).

^{31}P NMR (162 MHz, Acetone-d_6) δ -144.27 (h, J = 707 Hz).

HRMS (ESI-TOF) $[M{-}PF_6]^+$ calculated for $[C_{19}H_{19}O_2S]^+$ m/z: 311.1100, found 311.1102.

3-butyl-4-(dimethylethylsulfoxonium)-2,5-diphenylfuran hexafluorophosphate (10b)

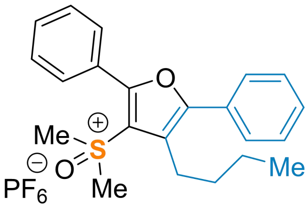

This compound was synthesized following the *general procedure D* using reagent **3c** (108.8 mg, 0.2 mmol), and hex-1-yn-1-ylbenzene (63.2 mg, 0.4 mmol). Purification by flash chromatography on silica gel (DCM/ Acetone: 4/1) provided the title compound as white solid (69.7 mg, 70% yield). Ratio of isomers was determined to be >20:1 from both the crude reaction mixture and purified products using ^{1}H NMR spectroscopy.

^{1}H NMR (400 MHz, Acetone-d_6) δ 7.89 (d, J = 7.3 Hz, 2H), 7.80 – 7.76 (m, 2H), 7.24 – 7.22 (m, 1H), 7.69 – 7.65 (m, 2H), 7.61 – 7.57 (m, 2H), 7.55 – 7.51 (m, 1H), 4.27 (s, 6H), 3.10 – 3.01 (m, 2H), 1.81 – 1.73 (m, 2H), 1.53 – 1.45 (m, 2H), 0.95 (t, J = 7.3 Hz, 3H).

^{13}C NMR (101 MHz, Acetone-d_6) δ 157.97, 151.92, 132.04, 130.48, 129.57, 129.18, 128.61, 127.32, 126.94, 120.75, 114.24, 43.48, 32.44, 23.72, 22.34, 13.02.

^{19}F NMR (376 MHz, Acetone-d_6) δ -72.32 (d, J = 707 Hz).

^{31}P NMR (162 MHz, Acetone-d_6) δ -144.26 (h, J = 708 Hz).

HRMS (ESI-TOF) $[M-PF_6]^+$ calculated for $[C_{22}H_{25}O_2S]^+$ m/z: 353.1570, found 353.1577.

3-cyclohexyl-4-(dimethylethylsulfoxonium)-2,5-diphenylfuran hexafluorophosphate (10c)

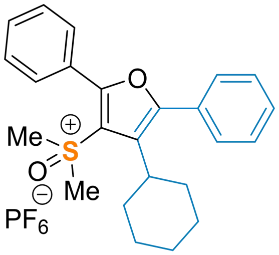

This compound was synthesized following the *general procedure D* using reagent **3c** (108.8 mg, 0.2 mmol), and (cyclohexylethynyl)benzene (73.7 mg, 0.4 mmol). Purification by flash chromatography on silica gel (DCM/ Acetone: 4/1) provided the title compound as white solid (65.0 mg, 62% yield). Ratio of isomers was determined to be >20:1 from both the crude reaction mixture and purified products using ^{1}H NMR spectroscopy.

^{1}H NMR (400 MHz, Acetone-d_6) δ 7.87 – 7.81 (m, 2H), 7.73 – 7.69 (m, 1H), 7.69 – 7.62 (m, 4H), 7.62 – 7.57 (m, 3H), 4.26 (s, 3H), 4.25 (s, 3H), 3.05 – 2.98 (m, 1H), 2.05 – 1.97 (m, 2H), 1.77 – 1.71 (m, 2H), 1.68 – 1.61 (m, 3H), 1.48 – 1.36 (m, 2H), 1.17 – 1.07 (m, 1H).

^{13}**C NMR** (101 MHz, Acetone-d_6) δ 157.83, 152.94, 131.89, 130.51, 130.17, 129.97, 129.92, 129.08, 128.69, 127.51, 125.48, 113.66, 43.87, 35.62, 32.75, 26.34, 25.41.

^{19}**F NMR** (376 MHz, Acetone-d_6) δ -72.40 (d, J = 707 Hz).

^{31}**P NMR** (162 MHz, Acetone-d_6) δ -144.26 (h, J = 708 Hz).

HRMS (ESI-TOF) $[M-PF_6]^+$ calculated for $[C_{24}H_{27}O_2S]^+$ m/z: 379.1726, found 379.1721.

4-cyclopentyl-2-(dimethyl(oxo)-λ^6-sulfanylidene)naphthalen-1(2*H*)-one (11a)

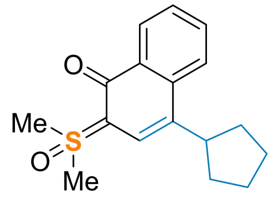

This compound was synthesized following the *general procedure C* using reagent **3c** (108.8 mg, 0.2 mmol), and ethynylcyclopentane (75.3 mg, 0.8 mmol). Purification by flash chromatography on silica gel DCM/ MeOH: 30/1) provided the title compound as light yellow solid (37.5 mg, 65% yield). Ratio of isomers was determined to be >20:1 from both the crude reaction mixture and purified products using ^{1}H NMR spectroscopy.

^{1}**H NMR** (400 MHz, $CDCl_3$) δ 8.47 (dd, J = 8.1, 1.5 Hz, 1H), 7.96 (d, J = 8.3 Hz, 1H), 7.64 – 7.60 (m, 1H), 7.46 – 7.42 (m, 1H), 7.17 (s, 1H), 3.80 (s, 6H), 3.52 – 3.44 (m, 1H), 2.25 – 2.10 (m, 2H), 1.88 – 1.65 (m, 6H).

^{13}**C NMR** (101 MHz, $CDCl_3$) δ 170.59, 138.34, 131.64, 129.79, 125.12, 124.43, 124.35, 124.11, 115.49, 94.38, 40.90, 40.84, 33.01, 25.03.

HRMS (ESI-TOF) $[M+H]^+$ calculated for $[C_{17}H_{21}O_2S]^+$ m/z: 289.1257, found 289.1258.

The crystal structure of **11a** has been deposited at the Cambridge Crystallographic Data Centre, CCDC 2144537.

4-butyl-2-(dimethyl(oxo)-λ^6-sulfanylidene)naphthalen-1(2*H*)-one (11b)

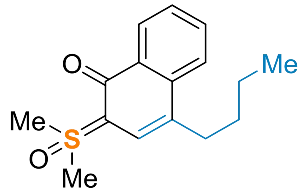

This compound was synthesized following the *general procedure C* using reagent **3c** (108.8 mg, 0.2 mmol), and 1-Hexyne (67.3 mg, 0.8 mmol). Purification by flash chromatography on silica gel DCM/ MeOH: 30/1) provided the title compound as white solid (37.6 mg, 68% yield). Ratio of isomers was determined to be >20:1 from both the crude reaction mixture and purified products using ^{1}H NMR spectroscopy.

^{1}H NMR (400 MHz, $CDCl_3$) δ 8.46 (d, J = 8.1 Hz, 1H), 7.83 (d, J = 8.3 Hz, 1H), 7.62 (t, J = 7.6 Hz, 1H), 7.45 (t, J = 7.5 Hz, 1H), 7.08 (s, 1H) 3.79 (s, 6H), 2.89 – 2.80 (m, 2H), 1.72 – 1.65 (m, 2H), 1.52 – 1.42 (m, 2H), 0.99 (t, J = 7.3 Hz, 3H).

^{13}C NMR (101 MHz, $CDCl_3$) δ 170.94, 137.92, 131.68, 129.87, 125.16, 124.46, 123.94, 121.20, 118.71, 94.13, 40.78, 32.50, 32.27, 22.89, 14.08.

HRMS (ESI-TOF) $[M+H]^+$ calculated for $[C_{16}H_{21}O_2S]^+$ m/z: 277.1257, found 277.1263.

2-(dimethyl(oxo)-λ^{6}-sulfanylidene)-4-pentylnaphthalen-1(2*H*)-one (11c)

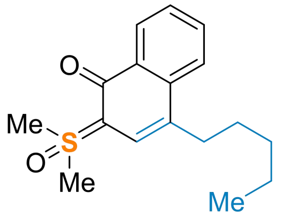

This compound was synthesized following the *general procedure C* using reagent **3c** (108.8 mg, 0.2 mmol), and 1-Heptyne (78.6 mg, 0.8 mmol). Purification by flash chromatography on silica gel (DCM/ MeOH: 30/1) provided the title compound as little yellow oil (36.6 mg, 63% yield). Ratio of isomers was determined to be >20:1 from both the crude reaction mixture and purified products using ^{1}H NMR spectroscopy.

^{1}H NMR (400 MHz, $CDCl_3$) δ 8.46 (d, J = 7.4 Hz, 1H), 7.83 (d, J = 8.3 Hz, 1H), 7.62 (t, J = 7.3 Hz, 1H), 7.45 (t, J = 7.5 Hz, 1H), 7.08 (s, 1H), 3.80 (s, 6H), 2.88 – 2.79 (m, 2H), 1.74 – 1.67 (m, 2H), 1.50 – 1.35 (m, 4H), 0.94 (t, J = 6.9 Hz, 3H).

^{13}C NMR (101 MHz, $CDCl_3$) δ 170.93, 137.92, 131.66, 129.88, 125.16, 124.46, 123.93, 121.29, 118.68, 94.13, 40.79, 32.57, 32.06, 30.04, 22.64, 14.15.

HRMS (ESI-TOF) $[M+H]^+$ calculated for $[C_{17}H_{23}O_2S]^+$ m/z: 291.1413, found 291.1419.

With allene:

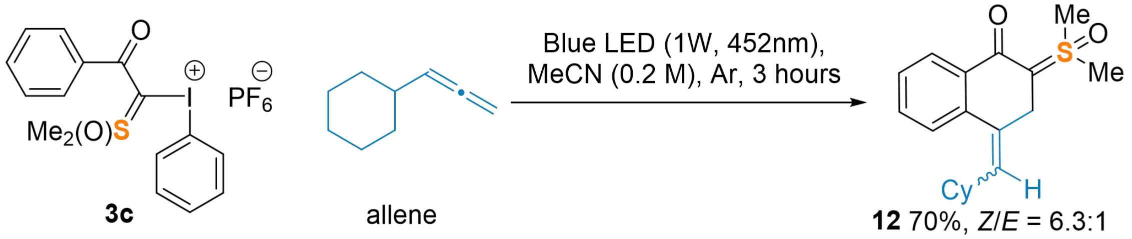

Mixture of (Z/*E*)-4-(cyclohexylmethylene)-2-(dimethyl(oxo)-λ^6-sulfanylidene)-3,4-dihydronaphthalen-1(2*H*)-one (12)

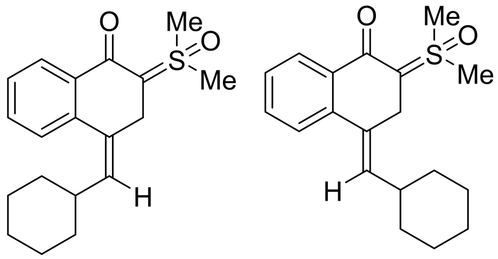

This compound was synthesized following the *general procedure C* using reagent **3c** (108.8 mg, 0.2 mmol), and 1-Cyclohexylallene (97.8 mg, 0.8 mmol). Purification by flash chromatography on silica gel (DCM/ Acetone: 4/1) provided the title compound as little yellow oil (44.3 mg, 70% yield). Ratio of isomers was determined to be 6.3:1 (*Z*:*E*) from both the crude reaction mixture and purified products using ^{1}H NMR spectroscopy.

major isomer ^{1}**H NMR** (400 MHz, Acetone-d_6) δ 7.95 – 7.93 (m, 1H), 7.53 – 7.50 (m, 1H), 7.41 – 7.35 (m, 1H), 7.32 – 7.27 (m, 1H), 5.77 (d, *J* = 9.0 Hz, 1H), 3.64 (s, 6H), 3.43 (d, *J* = 1.8 Hz, 2H), 2.51 – 2.44 (m, 1H), 1.78 – 1.71 (m, 4H), 1.45 – 1.34 (m, 2H), 1.31 – 1.19 (m, 4H).

^{13}**C NMR** (101 MHz, Acetone-d_6) δ 173.94, 139.58, 134.80, 132.80, 131.55, 130.13, 126.80, 124.81, 123.59, 41.13, 37.01, 32.84, 25.83, 25.68, 22.64.

HRMS (ESI-TOF) $[M+H]^+$ calculated for $[C_{19}H_{25}O_2S]^+$ m/z: 317.1570, found 317.1572.

2.5 Mechanistic experiments.

UV-Visible absorption analysis

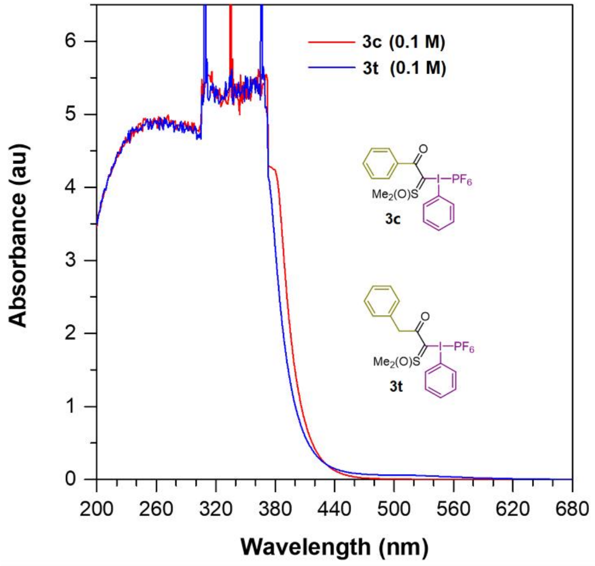

Supplementary Figure 3. UV-Vis spectra of $I^{(III)}/S^{(VI)}$ Reagents **3c** and **3t** at 0.1M in MeCN.

Cation trap experiment

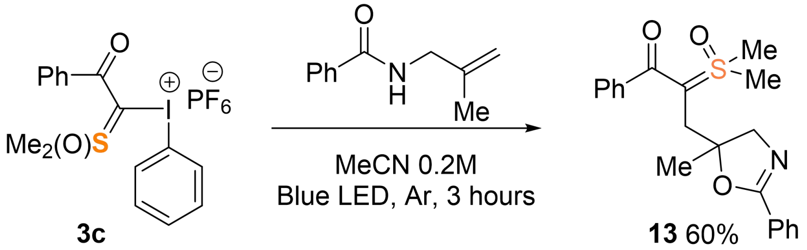

2-(dimethyl(oxo)-λ⁶-sulfaneylidene)-3-(5-methyl-4,5-dihydrooxazol-5-yl)-1-phenylpropan-1-one, 13: Compound **13** was synthesized following the *general procedure C* using reagent **3c** (108.8 mg, 0.2 mmol), and *N*-(2-Methylallyl)benzamide[4] (140.0 mg, 0.8 mmol). Purification by flash chromatography on silica gel (DCM/ MeOH: 30/1) provided the title compound as white solid (35.2 mg, 60% yield). No [4+2] product has been detected in this process.

^{1}H NMR (400 MHz, Acetonitrile-d_3) δ 7.89 – 7.80 (m, 2H), 7.75 – 7.70 (m, 2H), 7.68 (t, *J* = 7.5 Hz, 1H), 7.60 – 7.55 (m, 3H), 7.53 – 7.49 (m, 2H), 3.87 (dd, *J* = 14.4, 6.5 Hz, 1H), 3.80 (dd, *J* = 14.4, 6.5 Hz, 1H), 3.59 (s, 3H), 3.56 (s, 3H), 3.45 (d, *J* = 13.7 Hz, 1H), 3.15 (d, *J* = 13.8 Hz, 1H), 1.70 (s, 3H).

^{13}C NMR (400 MHz, Acetonitrile-d_3) δ 172.52, 168.13, 134.18, 132.89, 131.75, 129.39, 128.73, 128.57, 127.24, 98.23, 92.58, 46.22, 41.13, 41.07, 39.54, 23.48.

Intramolecular cyclopropanation

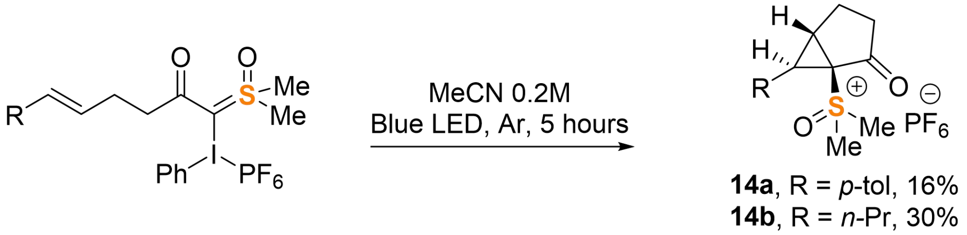

1-dimethylethylsulfoxonium-6-(*p*-tolyl)bicyclo[3.1.0]hexan-2-one hexafluorophosphate (14a)

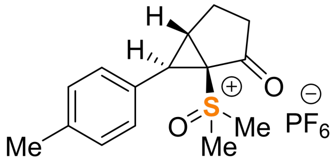

14a

14a was synthesized following the *general procedure C* using reagent **3v** (122.4 mg, 0.2 mmol). Ratio of diastereoisomers was determined to be >20:1 from the crude reaction mixture using ^{1}H NMR spectroscopy. Purification by flash chromatography on silica gel (DCM/ MeOH: 30/1) provided the title compound as white solid (13.0 mg, 16% yield).

^{1}H NMR (400 MHz, Acetone-*d$_6$*) δ 7.55 (d, *J* = 8.2 Hz, 2H), 7.25 (d, *J* = 7.9 Hz, 2H), 4.29 – 4.20 (m, 2H), 3.94 (s, 3H), 3.79 (s, 3H), 2.85 (dd, *J* = 18.2, 9.3 Hz, 1H), 2.73 (dtd, *J* = 12.5, 9.1, 4.8 Hz, 1H), 2.61 (ddd, *J* = 18.2, 9.4, 1.2 Hz, 1H), 2.49 – 2.40 (m, 1H), 2.34 (s, 3H).

^{13}C NMR (400 MHz, Acetone-*d$_6$*) δ 203.08, 139.19, 130.05, 129.50, 125.33, 55.00, 40.01, 39.86, 39.47, 33.94, 33.82, 21.11, 20.30.

^{19}F NMR (376 MHz, Acetone-*d$_6$*) δ -72.68 (d, *J* = 707 Hz).

^{31}P NMR (162 MHz, Acetone-*d$_6$*) δ -144.26 (h, *J* = 708 Hz).

HRMS (ESI-TOF) $[M+H]^+$ calculated for $[C_{15}H_{19}O_2S]^+$ m/z: 263.1100, found 263.1104.

1-dimethylethylsulfoxonium-6-propylbicyclo[3.1.0]hexan-2-one hexafluorophosphate (14b)

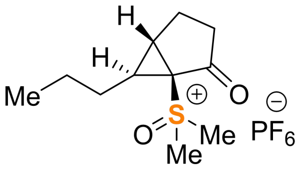

14b

14b was synthesized following the *general procedure C* using reagent **3w** (112.8 mg, 0.2 mmol). Ratio of diastereoisomers was determined to be >20:1 from the crude reaction mixture using ^{1}H NMR spectroscopy.

Purification by flash chromatography on silica gel (DCM/ MeOH: 30/1) provided the title compound as white solid (21.6 mg, 30% yield).

^{1}H NMR (400 MHz, Acetone-*d$_6$*) δ 4.16 (d, *J* = 0.9 Hz, 3H), 4.05 (d, *J* = 0.9 Hz, 3H), 3.38 – 3.45 (m, 1H), 2.82 – 2.71 (m, 2H), 2.61 – 2.46 (m, 2H), 2.35 – 2.26 (m, 1H), 2.16 (dtd, *J* = 13.7, 7.0, 3.7 Hz, 1H), 1.81 – 1.72 (m, 1H), 1.68 – 1.57 (m, 2H), 1.00 (t, *J* = 7.3 Hz, 3H).

^{13}C NMR (101 MHz, Acetone-*d$_6$*) δ 203.98, 53.28, 40.39, 39.74, 37.85, 37.48, 33.88, 27.96, 22.46, 21.22, 12.94.

^{19}F NMR (376 MHz, Acetone-*d$_6$*) δ -72.36 (d, *J* = 707 Hz).

^{31}P NMR (162 MHz, Acetone-*d$_6$*) δ -144.28 (h, *J* = 708 Hz).

HRMS (ESI-TOF) $[M+H]^+$ calculated for $[C_{11}H_{19}O_2S]^+$ m/z: 215.1100, found 215.1102.

2.6 Synthetic Application of [4+2] and [3+2] Products.

Large scale experiment.

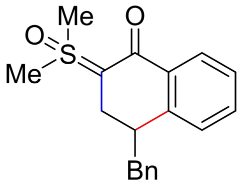

This compound was synthesized following the *general procedure C* using reagent **3c** (544.0 mg, 1.0 mmol), and allylbenzene (945.0 mg, 8.0 mmol). Purification by flash chromatography on silica gel (DCM/ MeOH: 30/1) provided the title compound as white solid (296.5 mg, 95% yield).

4-benzyl-3,4-dihydronaphthalen-1(2*H*)-one (15)

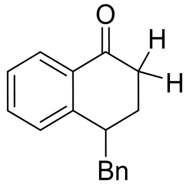

This compound was synthesized using following procedure[2], wet Raney nickel (230 μL of heterogeneous solution) was added to a solution of **5i** (62.4 mg, 0.2 mmol) in *i*-PrOH (3 mL) and the system was then warmed until the reflux temperature. After 1 hour, the crude product is filtered through a pad of celite and washed with 5 mL of *i*-PrOH. Solvent was removed under *vacuum* and the crude mixture was purified by flash column chromatography to yield the product **15** (34.0 mg, 72%). Its spectra are consistent the reported literature[3], and this result further serves as a demonstration of the regioselectivity for compound 5i.

^{1}H NMR (400 MHz, $CDCl_3$) δ 8.09 (dd, *J* = 7.9, 1.5 Hz, 1H), 7.49 (td, *J* = 7.5, 1.5 Hz, 1H), 7.39 – 7.32 (m, 3H), 7.30 – 7.26 (m, 1H), 7.24 – 7.18 (m, 3H), 3.29 – 3.23 (m, 1H), 3.17 – 3.12 (m, 1H), 2.94 – 2.80 (m, 2H), 2.64 – 2.56 (m, 1H), 2.22 – 2.13 (m, 1H), 2.01 – 1.94 (m, 1H).

^{13}C NMR (101 MHz, $CDCl_3$) δ 198.30, 147.46, 139.82, 133.55, 132.03, 129.11, 128.63, 128.47, 127.48, 126.99, 126.52, 41.33, 40.05, 34.81, 26.14.

HRMS (ESI-TOF) $[M+H]^+$ calculated for $[C_{17}H_{17}O]^+$ m/z: 237.1274, found 237.1278.

4-benzyl-2-(dimethylethylsulfoxonium)-3,4-dihydronaphthalen-1(2*H*)-one trifluoromethanesulfonate (16)

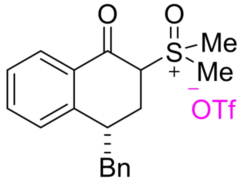

(dr 1:1.5)

To a 10 mL oven-dried tube equipped with a stirring bar was added **5i** (62.4 mg, 0.2 mmol), HOTf (19.5 µL, 0.22 mmol) and anhydrous MeCN (1.0 mL). The reaction vial was capped with a rubber septum under an air atmosphere for 10 minutes. Solvent was removed under *vacuum* and the crude mixture was washed with hexane (10 mL) and Et_2O (1mL) to yield white solid **16** (83.2 mg, 90% yield).

^{1}H NMR (400 MHz, CD_3CN) δ 8.09 – 8.06 (m, 2H), 7.90 – 7.79 (m, 2H), 7.70 – 7.60 (m, 2H), 7.58 – 7.46 (m, 3H), 7.44 – 7.37 (m, 7H), 7.34 – 7.30 (m, 7H), 5.63 (dd, *J* = 14.5, 4.5 Hz, 2H), 5.36 (dd, *J* = 14.2, 4.3 Hz, 1H), 3.88 (s, 4H), 3.87 (s, 5H), 3.83 – 3.82 (m, 1H), 3.79 (s, 3H), 3.78 (s, 3H), 3.74 – 3.70 (m, 3H), 3.16 – 3.13 (m, 3H), 2.88 – 2.70 (m, 3H), 2.59 – 2.51 (m, 3H), 2.39 – 2.29 (m, 1H).

^{13}C NMR (101 MHz, CD_3CN) δ 188.00, 187.39, 146.59, 146.08, 138.84, 138.24, 136.23, 136.02, 130.91, 129.95, 129.75, 129.38, 129.08, 128.78, 128.68, 128.03, 127.95, 127.82, 127.77, 127.31, 126.92, 126.77, 122.00, 118.83, 68.35, 65.34, 40.53, 39.66, 39.36, 39.29, 39.06, 38.27, 37.86, 37.81, 26.42, 24.36.

^{19}F NMR (376 MHz, CD_3CN) δ -79.49.

HRMS (ESI-TOF) $[M{-}OTf]^+$ calculated for $[C_{19}H_{21}O_2S]^+$ m/z: 313.1257, found 313.1254.

4-benzyl-2-((4-methoxyphenyl)thio)-3,4-dihydronaphthalen-1(2*H*)-one (17)

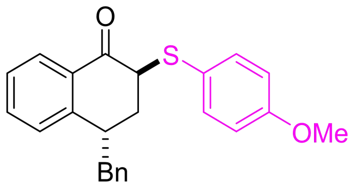

To a 15 mL oven-dried tube equipped with a stirring bar was added **5i** (31.2 mg, 0.1 mmol), 4-hydroxydinaphtho[2,1-d:1',2'-*f*][1,3,2]dioxaphosphepine 4-oxide (3.4 mg, 0.01 mmol), 4-methoxybenzenethiol (70.0 mg, 0.5 mmol) and anhydrous DCM (1.0 mL). The reaction vial was capped with a rubber septum under an air atmosphere and it was fixed on oil bath at 80 ℃ for 10 hours. Solvent

was removed under *vacuum* and the crude mixture was purified by flash column chromatography (Hexane/ EA : 10/1) to provide the title compound as yellow oil (21.0 mg, 56% yield).

^{1}H NMR (400 MHz, $CDCl_3$) δ 8.11 (dd, *J* = 7.9, 1.5 Hz, 1H), 7.57 – 7.53 (m, 1H), 7.46 – 7.32 (m, 4H), 7.32 – 7.20 (m, 4H), 7.07 – 7.04 (m, 2H), 6.89 – 6.79 (m, 2H), 4.11 – 4.07 (m, 1H), 3.82 (s, 3H), 3.45 – 3.41 (m, 1H), 3.27 – 3.23 (m, 1H), 2.80 – 2.73 (m, 1H), 2.25 – 2.15 (m, 2H).

^{13}C NMR (101 MHz, $CDCl_3$) δ 194.04, 160.07, 146.04, 139.14, 136.61, 133.74, 131.30, 129.23, 128.99, 128.58, 128.26, 127.79, 127.08, 126.44, 122.98, 114.63, 114.48, 55.32, 52.64, 41.19, 38.09, 33.16.

HRMS (ESI-TOF) $[M+Na]^+$ calculated for $[C_{24}H_{22}NaO_2S]^+$ m/z: 397.1233, found 397.1233.

2,2,5-triphenyl-4-(p-tolyl)-2,3-dihydrofuran (18)

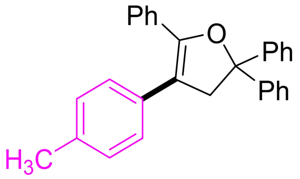

To a 15 mL oven-dried tube equipped with a stirring bar was added **7a** (52.0 mg, 0.1 mmol), *p*-tolylboronic acid (27.2 mg, 0.2 mmol), cesium carbonate (65.0 mg, 0.2 mmol), bis(acetonitrile)dichloropalladium(II) (0.5 mg, 0.002 mmol) and anhydrous MeOH (1.0 mL). The reaction vial was capped with a rubber septum and degassed with nitrogen 3 times. The tube was fixed on oil bath at 50 ℃ for 24 hours. Solvent was removed under *vacuum* and the crude mixture was purified by flash column chromatography (Hexane/ EA : 5/1) to provide the title compound as yellow oil (20.2 mg, 52% yield).

^{1}H NMR (400 MHz, $CDCl_3$) δ 7.62 (dd, *J* = 4.1, 1.9 Hz, 2H), 7.57 – 7.52 (m, 4H), 7.36 – 7.29 (m, 7H), 7.26 – 7.22 (m, 2H), 7.11 (d, *J* = 8.1 Hz, 2H), 7.02 (d, *J* = 8.0 Hz, 2H), 3.88 (s, 2H), 2.30 (s, 3H).

^{13}C NMR (101 MHz, $CDCl_3$) δ 146.33, 135.80, 132.48, 131.81, 128.54, 128.23, 128.13, 128.05, 127.24, 127.14, 125.84, 109.42, 88.25, 50.00, 21.13.

HRMS (ESI-TOF) $[M+Na]^+$ calculated for $[C_{29}H_{24}NaO]^+$ m/z: 411.1719, found 411.1720.

4-(methylsulfinyl)-2,2,5-triphenyl-2,3-dihydrofuran (19)

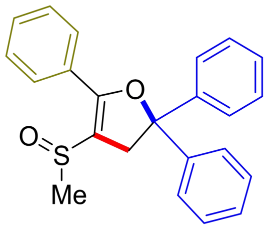

To a 10 mL oven-dried flask equipped with a stirring bar was added **7a** (52.0 mg, 0.1 mmol), potassium iodide (49.8 mg, 0.3 mmol) and anhydrous acetone (2.0 mL). The reaction mixture was heated until reflux for 2 hours. After cooling down to room temperature, solvent was removed under *vacuum* and the crude mixture was purified by flash column chromatography (Hexane/ EA : 2/1) to provide the title compound as yellow oil (31.7 mg, 88% yield).

^{1}H NMR (400 MHz, $CDCl_3$) δ 7.75 – 7.69 (m, 2H), 7.51 – 7.43 (m, 7H), 7.39 – 7.28 (m, 6H), 4.19 (d, J = 14.7 Hz, 1H), 3.74 (d, J = 14.7 Hz, 1H), 2.72 (s, 3H).

^{13}C NMR (101 MHz, $CDCl_3$) δ 159.40, 144.94, 144.11, 130.80, 128.59, 128.51, 128.47, 127.89, 127.72, 125.88, 125.50, 111.20, 91.74, 39.98, 38.36.

HRMS (ESI-TOF) $[M+Na]^+$ calculated for $[C_{23}H_{20}NaO_2S]^+$ m/z: 383.1076, found 383.1081.

4-(2,2-diphenylvinyl)-2,2,5-triphenyl-2,3-dihydrofuran (20)

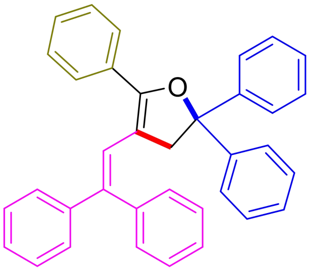

To a 15 mL oven-dried tube equipped with a stirring bar was added **7a** (52.0 mg, 0.1 mmol), $\{Ir[dF(CF_3)ppy]_2(dtbbpy)\}PF_6$ (2.2 mg, 0.002 mmol) and anhydrous MeCN (1.0 mL). 1,1-diphenyl ethylene (36.0 mg, 0.2 mmol) was added and the reaction tube was capped with a rubber septum and degassed with nitrogen 3 times. The tube was placed close to blue LED and stirred at room temperature for 16 hours. Solvent was removed under *vacuum* and the crude mixture was purified by flash column chromatography (Hexane/ EA : 10/1) to provide the title compound as yellowish solid (33.3 mg, 70% yield).

^{1}H NMR (400 MHz, $CDCl_3$) δ 7.83 – 7.77 (m, 2H), 7.49 – 7.30 (m, 14H), 7.25 (m, 9H), 6.98 (s, 1H), 2.99 (d, J = 1.1 Hz, 2H).

^{13}C NMR (101 MHz, $CDCl_3$) δ 145.96, 143.13, 140.88, 139.14, 131.32, 130.88, 128.82, 128.35, 128.29, 128.11, 128.08, 127.87, 127.35, 127.08, 126.72, 125.77, 122.16, 110.72, 89.74, 46.55.

HRMS (ESI-TOF) $[M+Na]^+$ calculated for $[C_{36}H_{28}NaO]^+$ m/z: 499.2032, found 499.2035.

2.7 Computational studies and computational details.

All calculations were performed using Gaussian 16, Revision A.03 package.[5] All of the intermediates were optimized by the DFT with the ωb97xd functional.[6] For geometry optimizations calculations, we employed LANL2DZ basis set for I with effective core potentials, 6-31G(d) basis sets for H, C, S, F, B, and O. All the stationary structures were characterized with no imaginary frequency. For all intermediates, the Mayer Bond Order and NBO charge of atoms were calculated using Multiwfn.[7] Electrostatic potential maps for each compound were mapped by Gaussview.

Supplementary Table 1: Cartesian coordinates of the optimized structures of **3a:**

E = -1366.810098 a.u.

0 1

I	9.55196900	3.28473400	6.33458900
S	7.35500000	2.52246000	4.08451200
O	5.31103200	2.50501700	6.05212300
O	8.57659300	2.63194800	3.27647900
C	7.55142300	3.06337300	5.69252600
C	6.36221100	3.00253500	6.49721500
C	6.36656700	3.52438900	7.90271700
C	7.00396100	4.71034700	8.27461500
H	7.53531400	5.30392000	7.53746200
C	6.93361500	5.16117800	9.58911500
H	7.42714400	6.08740500	9.86653900
C	6.23375100	4.42678500	10.54222100
H	6.18871400	4.77420700	11.57008400
C	5.58335000	3.25053900	10.17375000
H	5.02841700	2.68049300	10.91271300
C	5.63804400	2.80926400	8.85742600
H	5.11687500	1.90788300	8.55159800
C	6.02583500	3.43675000	3.30717100

H	6.39088400	4.45941100	3.19592100
H	5.14905200	3.38623900	3.95350000
H	5.84228900	2.99305600	2.32616700
C	6.76137800	0.83166400	4.04347800
H	5.86929600	0.77739900	4.66886300
H	7.57062700	0.22004100	4.44770000
H	6.56318800	0.56160500	3.00390100
C	9.69013900	5.32760900	5.75986400
C	8.64009000	6.02187000	5.18385500
H	7.68206900	5.54129300	5.01209100
C	8.85135400	7.36011000	4.85698100
H	8.04576000	7.93283700	4.40759500
C	10.08568500	7.96114400	5.10552000
H	10.23972500	9.00351100	4.84442200
C	11.11519100	7.23278800	5.69047800
H	12.08506700	7.67089900	5.90323400
C	10.92158400	5.89440400	6.02914000
C	12.01562800	5.07273400	6.68815900
O	13.09932700	5.57701200	6.92258000
O	11.64577600	3.85736100	6.94598000

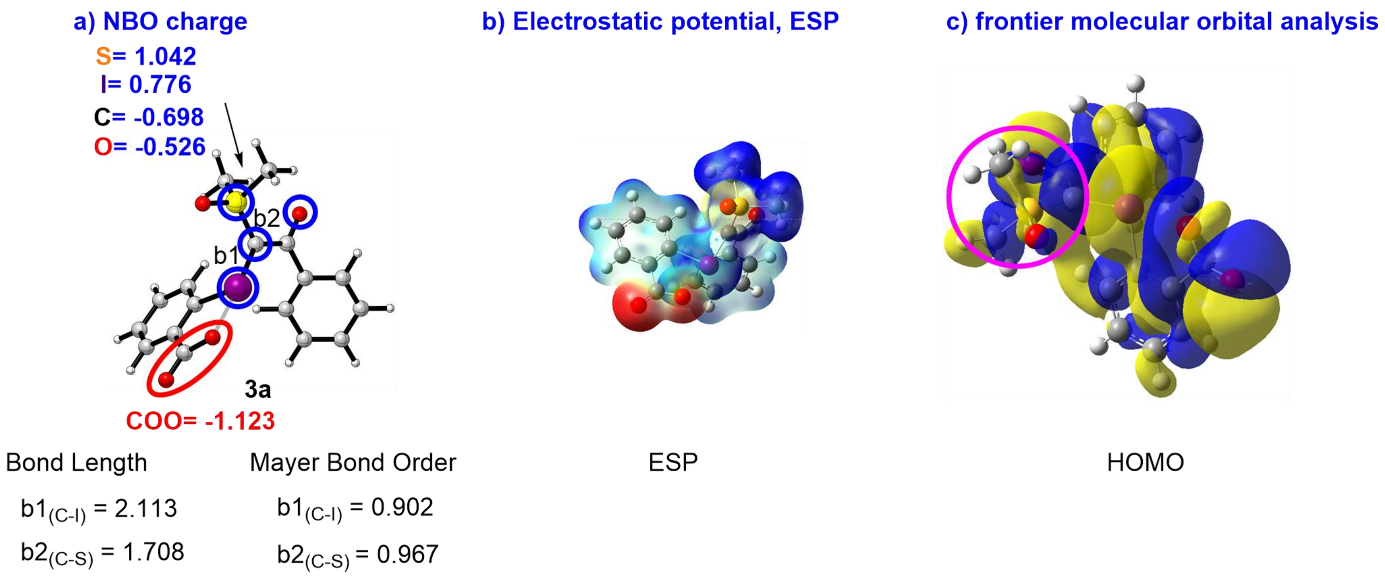

Supplementary Figure 4. DFT analysis results of reagent **3a**. a) NBO charge distribution; b) Electrostatic potential map; c) Frontier molecular orbital analysis.

Supplementary Table 2: Cartesian coordinates of the optimized structures of **3e:**

E = -1603.231248 a.u.

0 1

I	9.31367400	3.15979800	5.82071700
S	6.88203900	2.71073100	3.87242700
O	5.25623300	2.49478800	6.26705900
O	7.90195500	3.04494500	2.86742100
C	7.29608100	3.28543100	5.44471800
C	6.34117500	3.03505200	6.50178600
C	6.67888400	3.49207500	7.88491200
C	7.17890800	4.77469500	8.12229200
H	7.33176100	5.45680700	7.28997400
C	7.46004000	5.18148400	9.42244800
H	7.83387800	6.18457100	9.60646900
C	7.25623900	4.30323800	10.48489800
H	7.48500400	4.61656800	11.49916800

C	6.75474300	3.02491300	10.24830600
H	6.59795500	2.34016600	11.07583500
C	6.45441800	2.62192500	8.95164600
H	6.06388500	1.62951600	8.75215400
C	5.33037500	3.49836600	3.47290300
H	5.52075200	4.57276400	3.47790200
H	4.59576100	3.21905300	4.22848200
H	5.05033600	3.15812500	2.47342000
C	6.52194900	0.96546800	3.84767900
H	5.82624400	0.76708900	4.66492500
H	7.47418900	0.44419500	4.00650200
H	6.09357600	0.73161000	2.87034000
C	9.76504500	5.19108400	5.47086300
C	9.70380700	5.69125600	4.17221900
H	9.40927400	5.04941900	3.34743100
C	10.01286300	7.03156700	3.95889400
H	9.96818800	7.43742100	2.95310400
C	10.38473900	7.84643000	5.02676100
H	10.62856400	8.88958200	4.85096600
C	10.44530100	7.32921200	6.31783300
H	10.73298900	7.96516900	7.14914600
C	10.13222500	5.99144200	6.55002100
H	10.16215400	5.58832600	7.55802700
F	9.20960400	-0.34086700	4.47502400
F	8.39499600	0.80291500	6.28495600
F	9.29468500	-1.30662700	6.56803000
F	10.64732800	0.49568500	6.06339100

B	9.42428600	-0.15078200	5.85044900

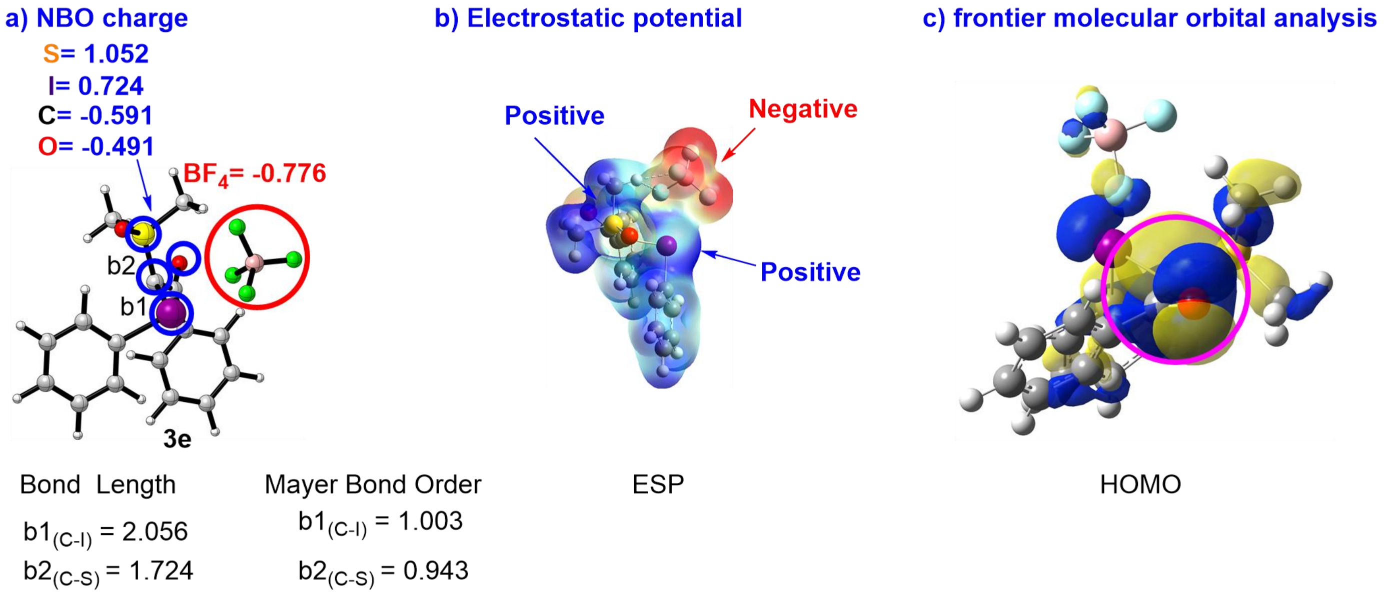

Supplementary Figure 5. DFT analysis results of reagent **3e**. a) NBO charge distribution; b) Electrostatic potential map; c) Frontier molecular orbital analysis.

3. Supplementary Figures.

3.1 NMR spectra.

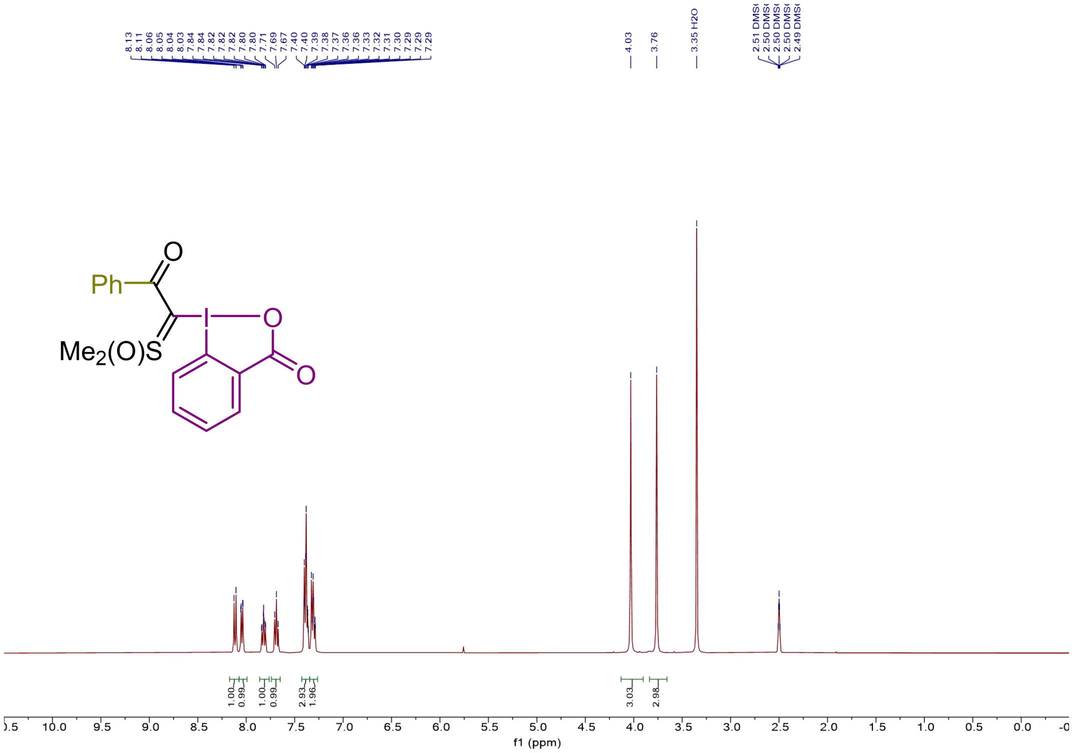

Supplementary Figure 6. ^{1}**H NMR** of the **3a** (400 MHz, 25 °C in DMSO-d_6)

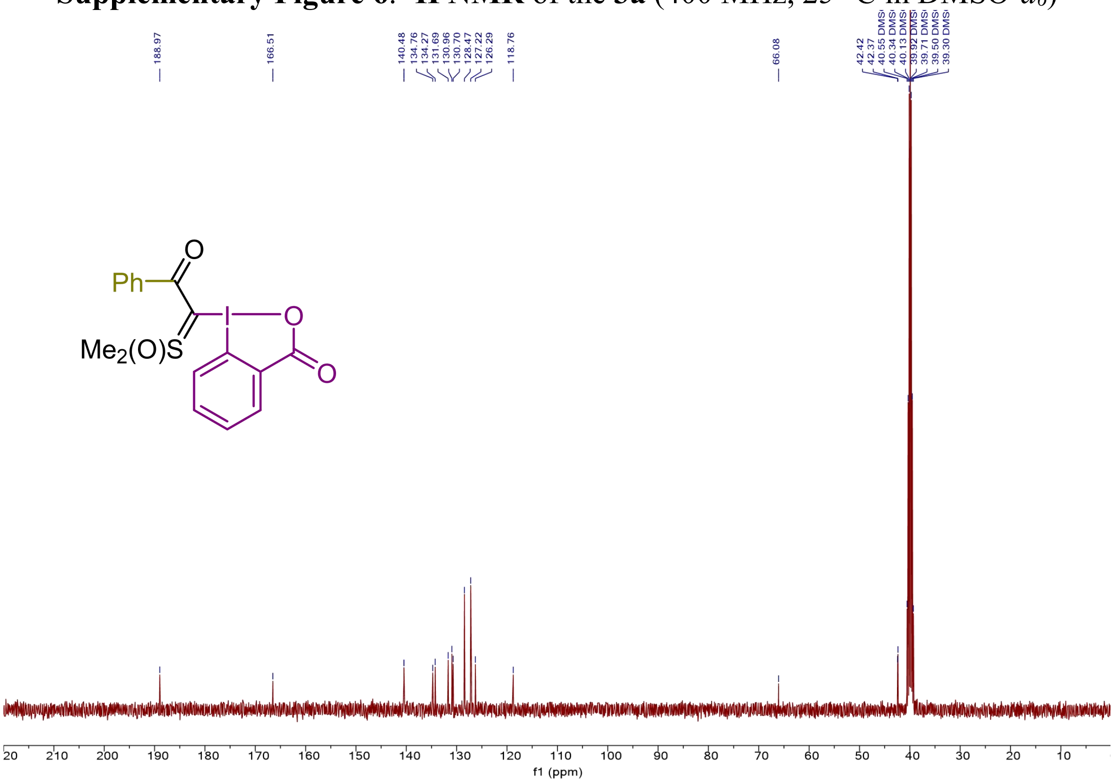

Supplementary Figure 7. ^{13}**C NMR** of the **3a** (101 MHz, 25 °C in DMSO-d_6)

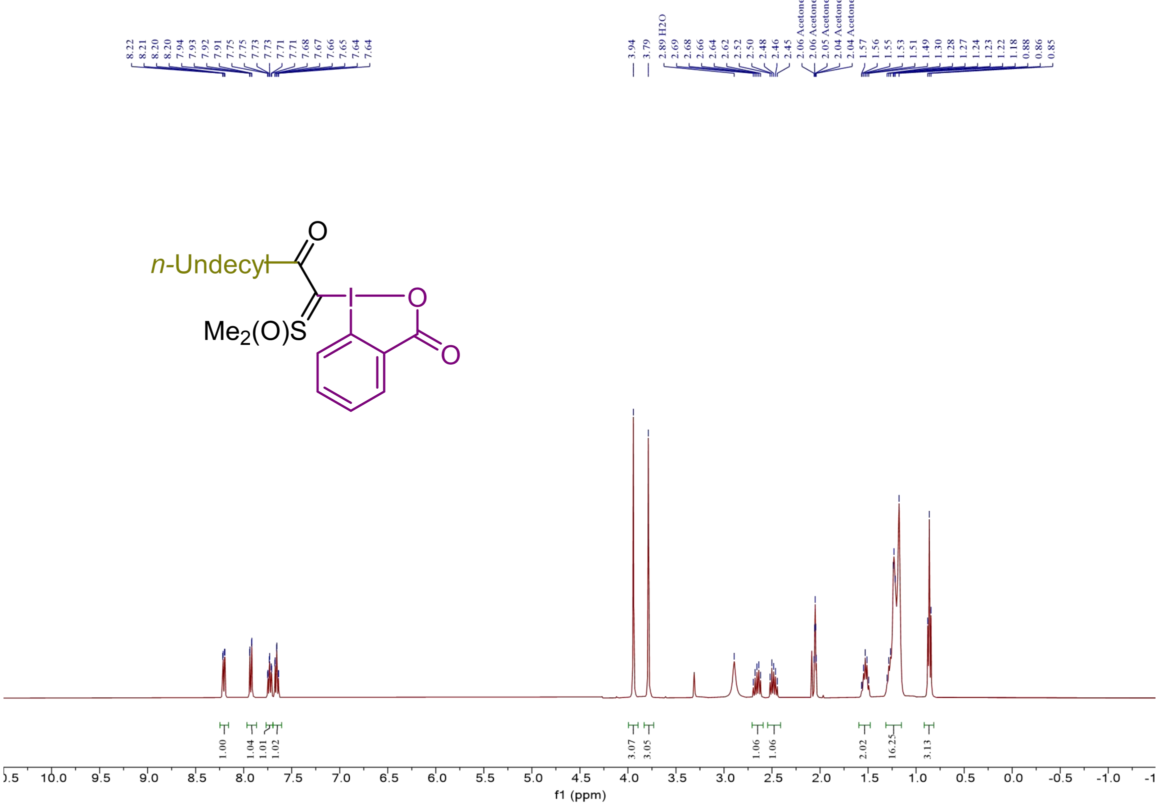

Supplementary Figure 8. **^{1}H NMR** of the **3b** (400 MHz, 25 °C in Acetone-d_6)

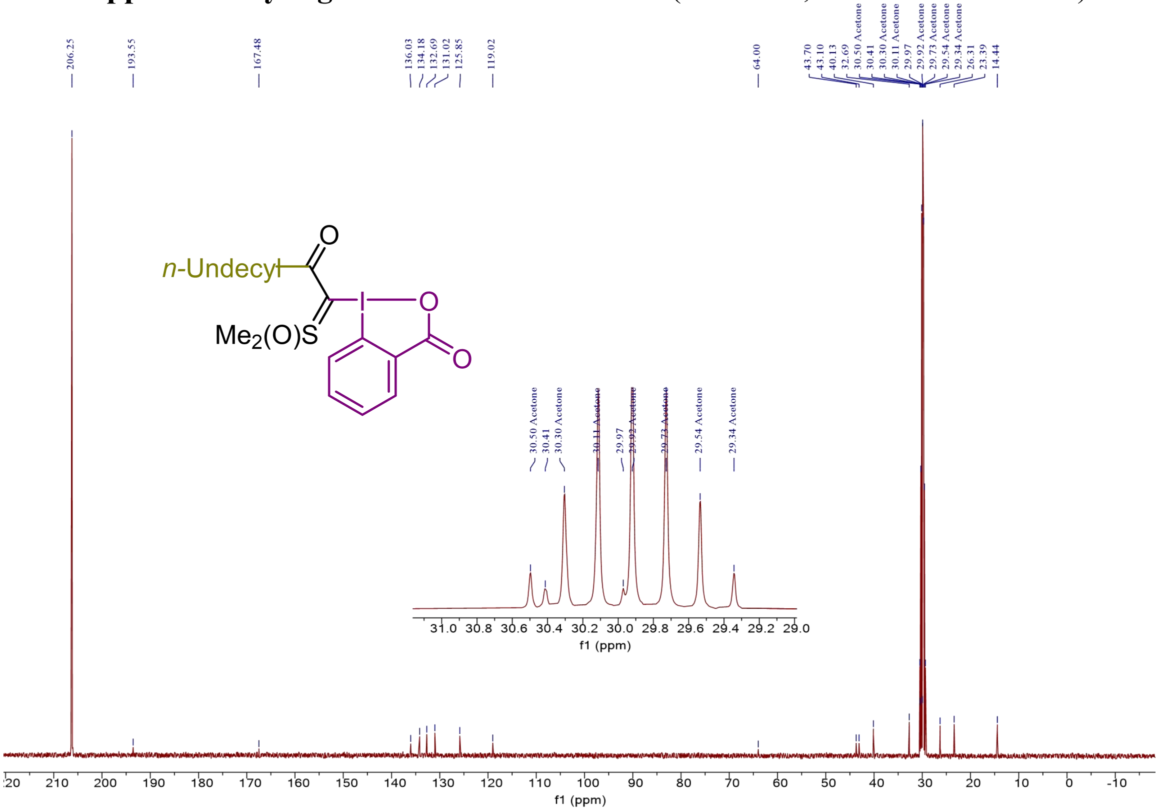

Supplementary Figure 9. **^{13}C NMR** of the **3b** (101 MHz, 25 °C in Acetone-d_6)

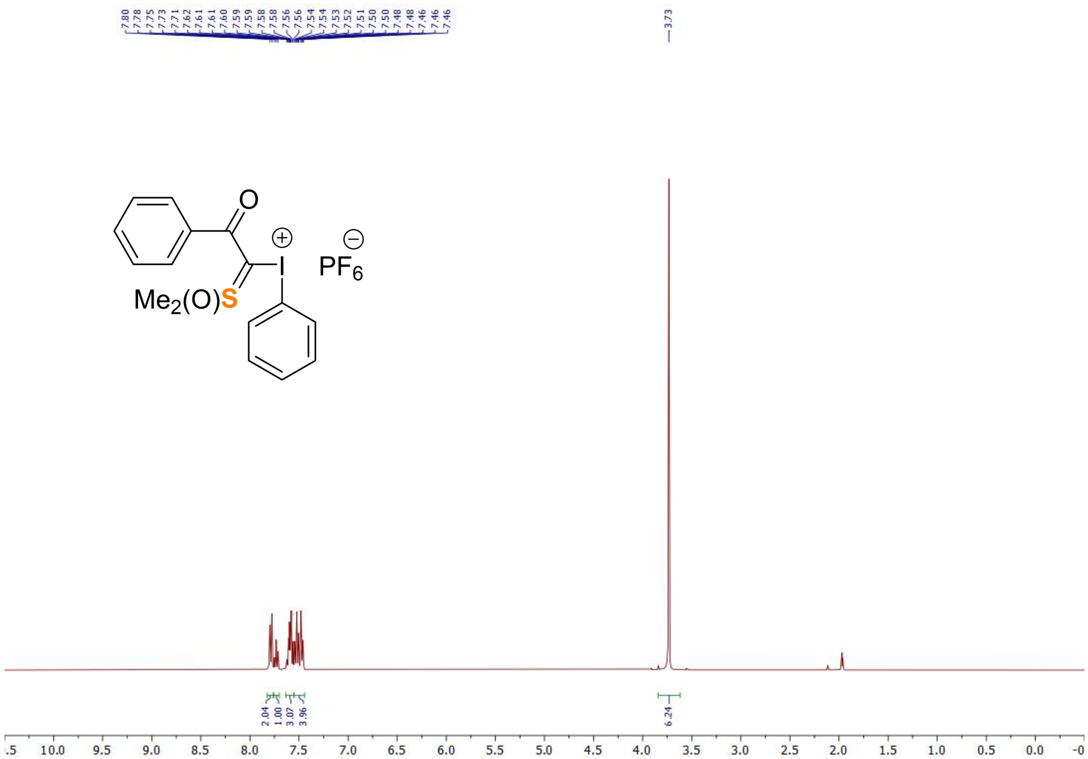

Supplementary Figure 10. **^{1}H NMR** of the **3c** (400 MHz, 25 °C in CD_3CN)

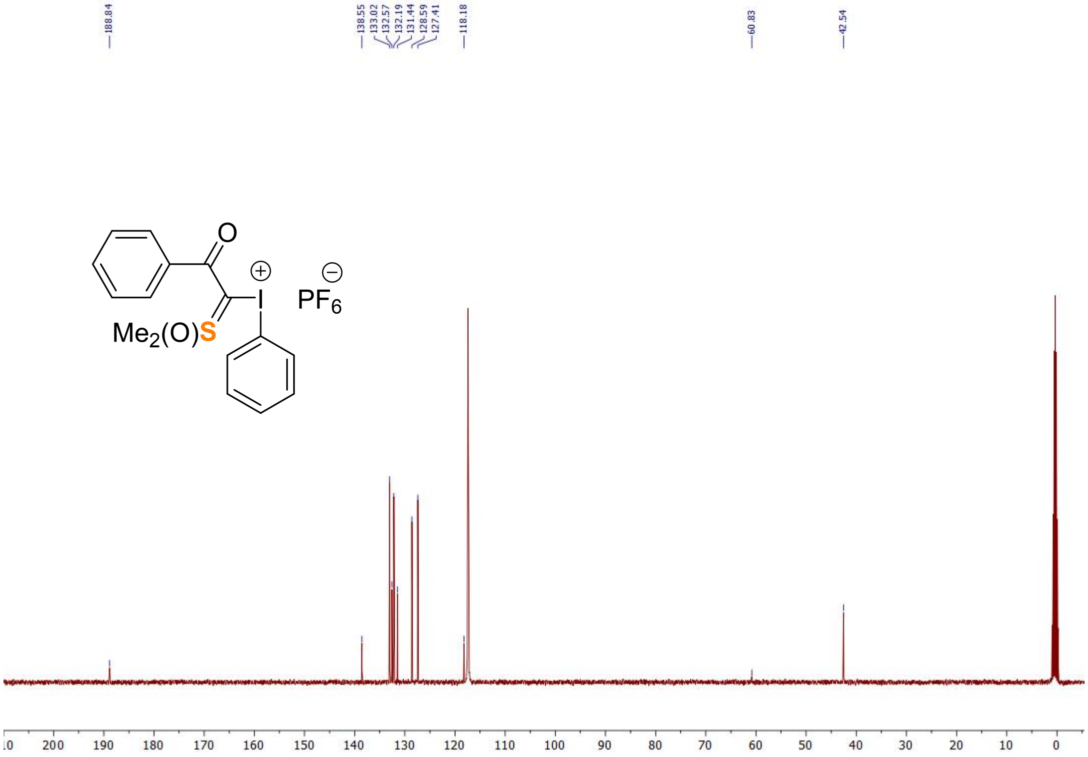

Supplementary Figure 11. **^{13}C NMR** of the **3c** (101 MHz, 25 °C in CD_3CN)

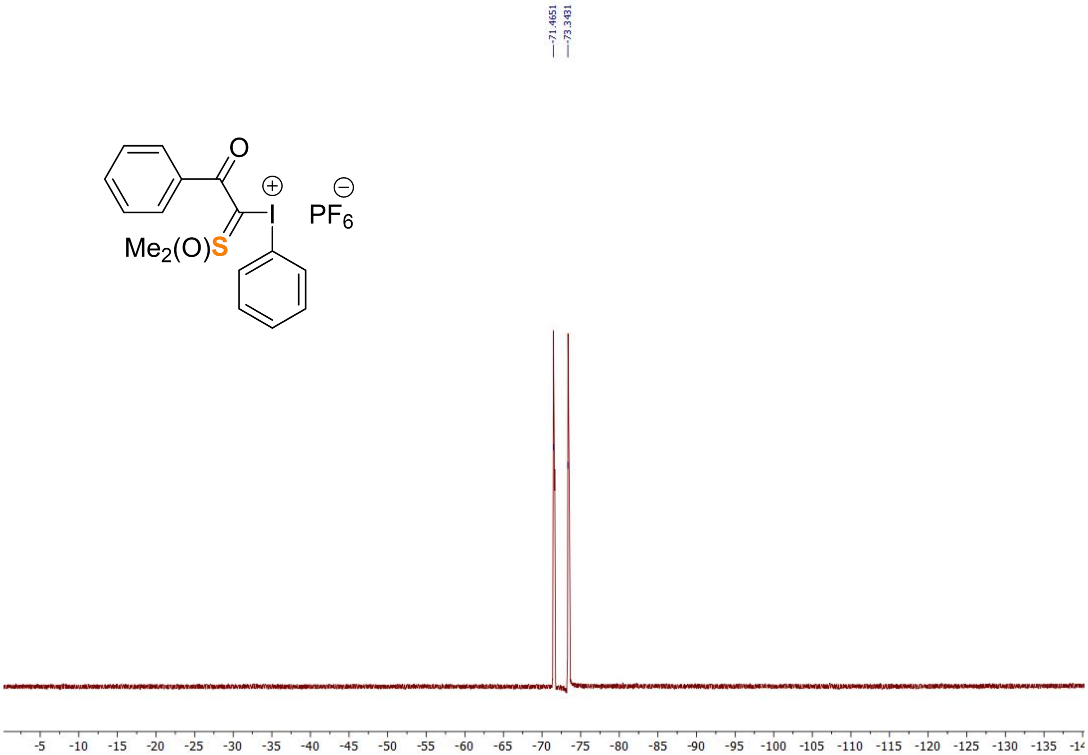

Supplementary Figure 12. **^{19}F NMR** of the **3c** (376 MHz, 25 °C in CD_3CN)

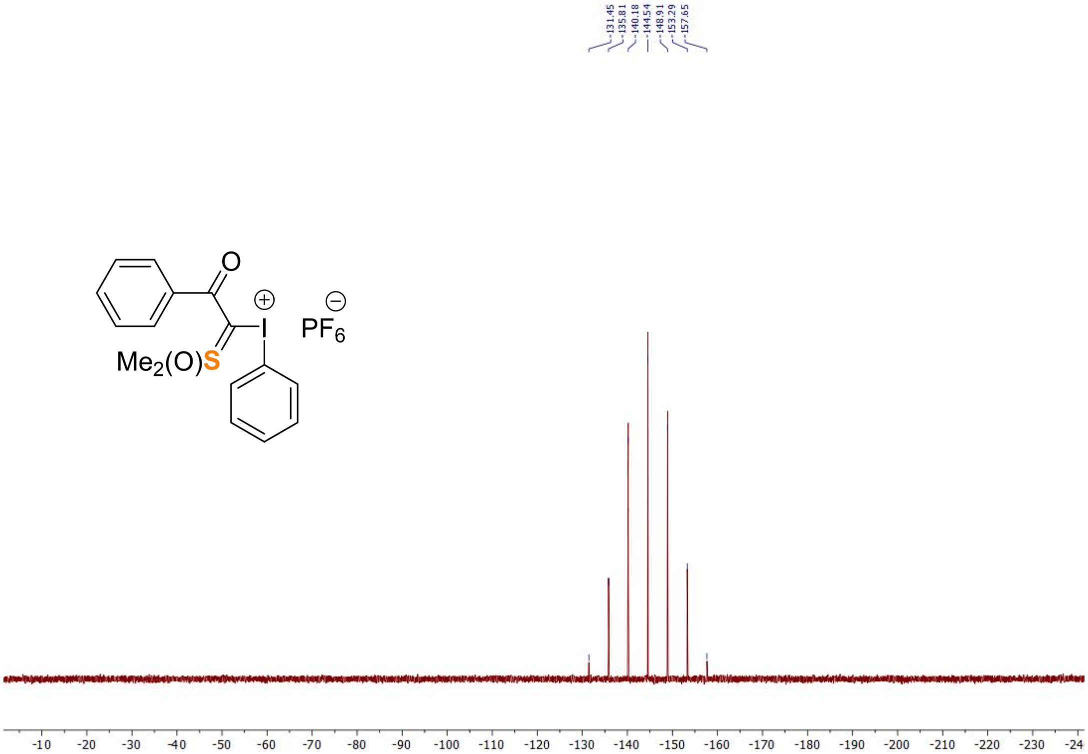

Supplementary Figure 13. **^{31}P NMR** of the **3c** (162 MHz, 25 °C in CD_3CN)

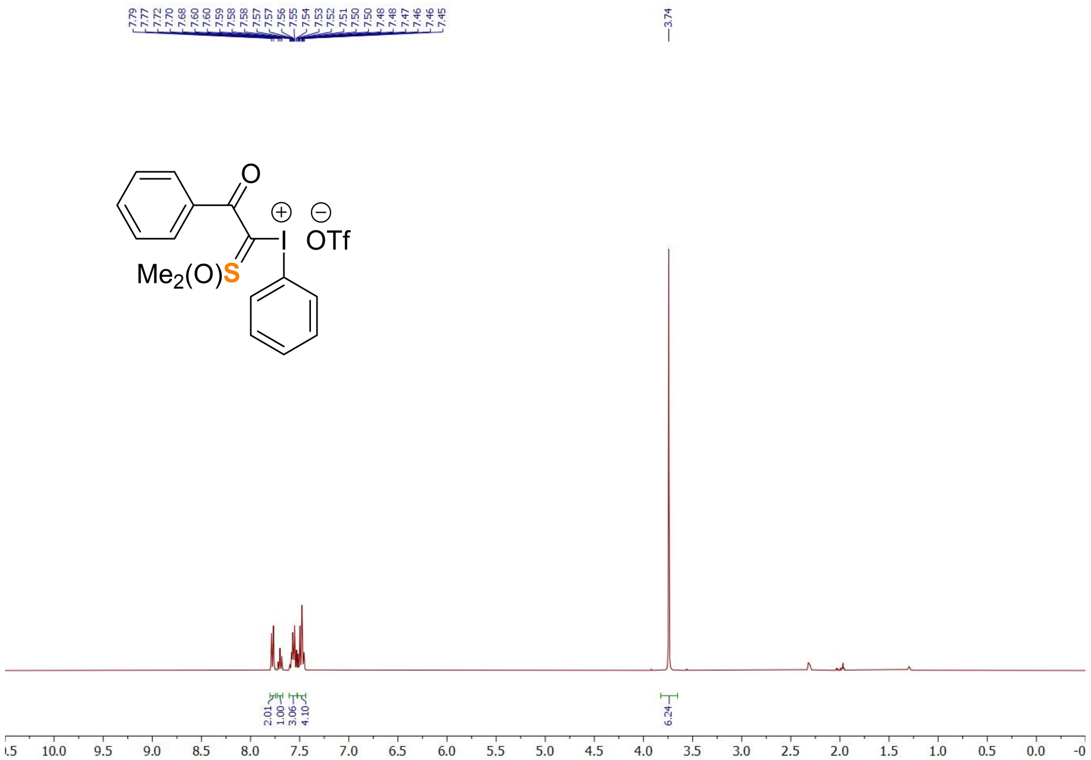

Supplementary Figure 14. **^{1}H NMR** of the **3d** (400 MHz, 25 °C in CD_3CN)

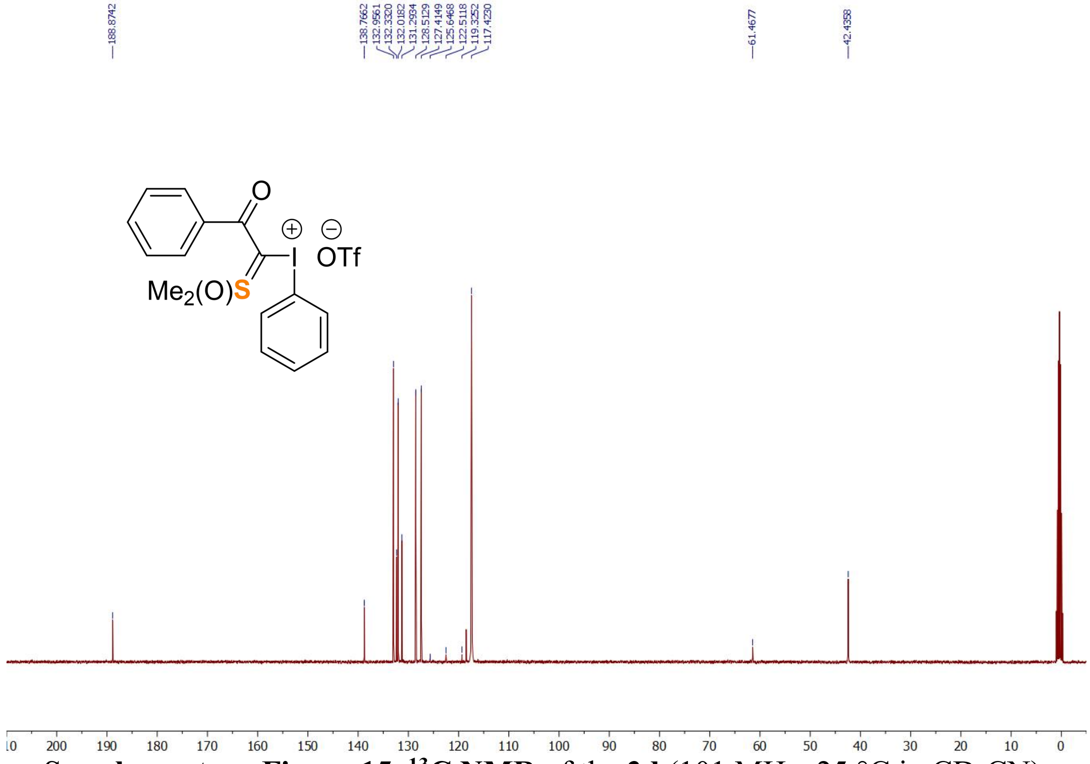

Supplementary Figure 15. **^{13}C NMR** of the **3d** (101 MHz, 25 °C in CD_3CN)

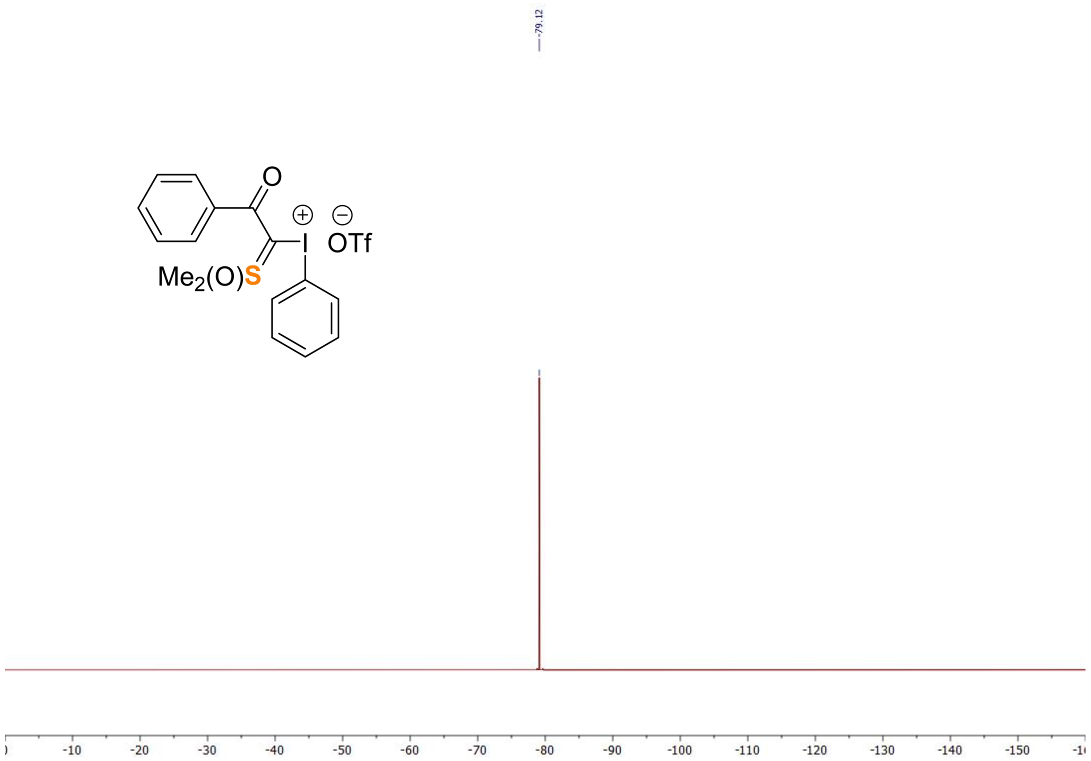

Supplementary Figure 16. **^{19}F NMR** of the **3d** (376 MHz, 25 °C in CD_3CN)

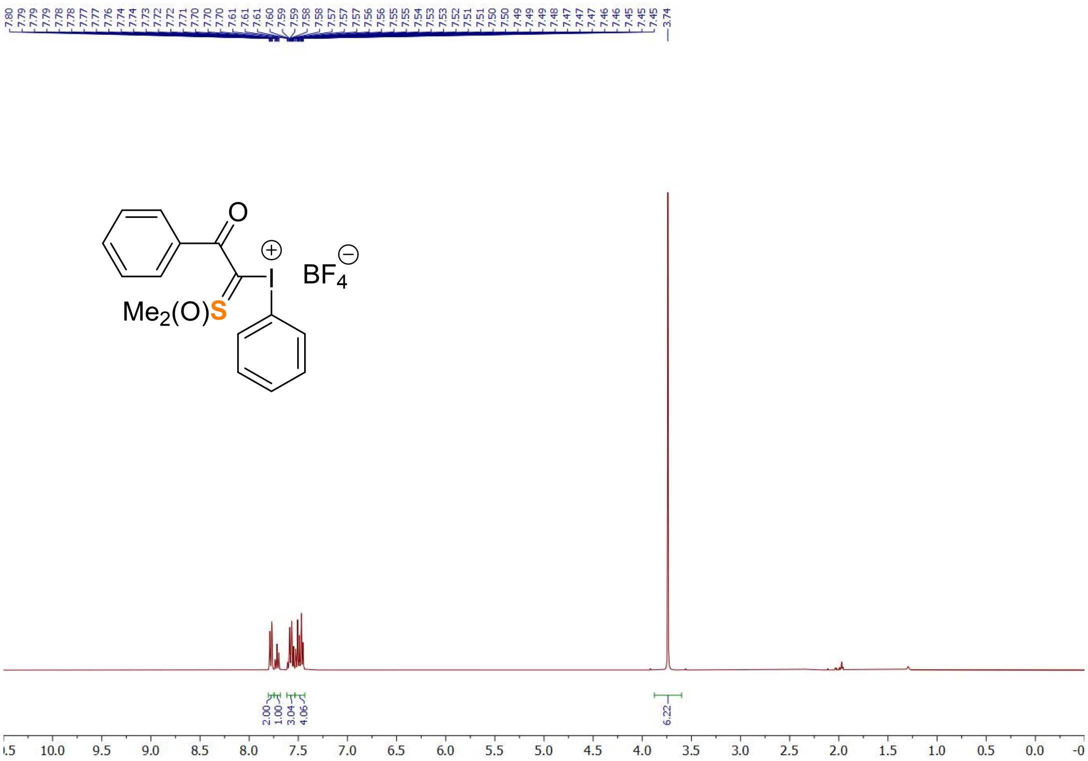

Supplementary Figure 17. **^{1}H NMR** of the **3e** (400 MHz, 25 °C in CD_3CN)

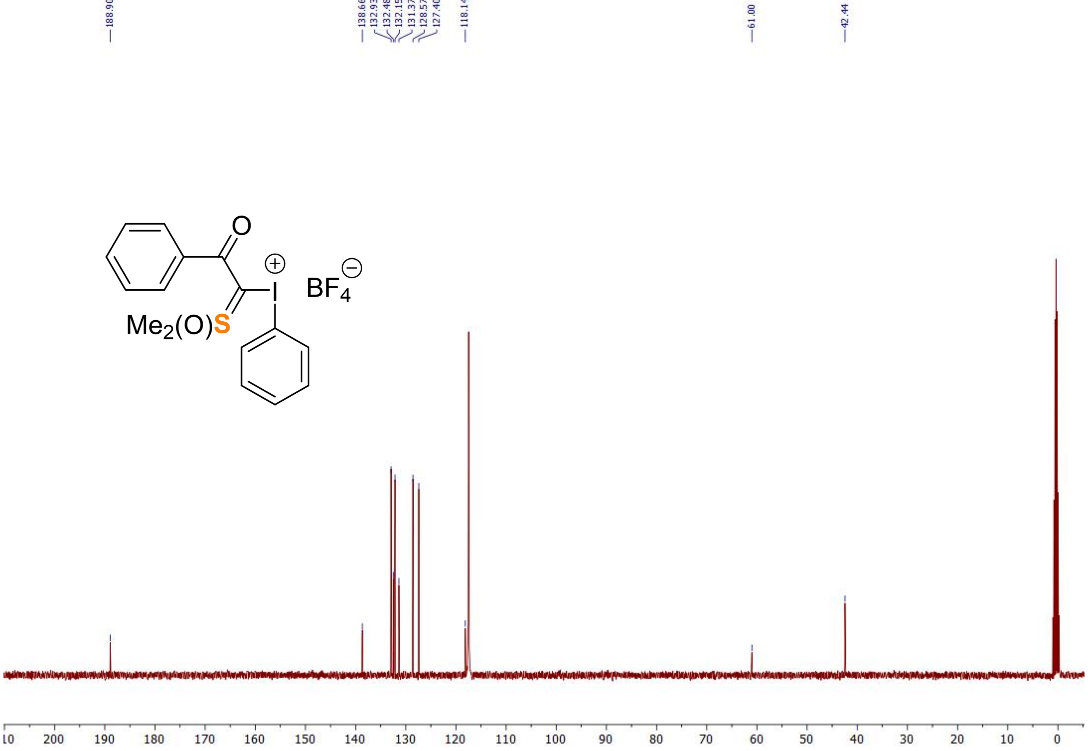

Supplementary Figure 18. **^{13}C NMR** of the **3e** (101 MHz, 25 °C in CD_3CN)

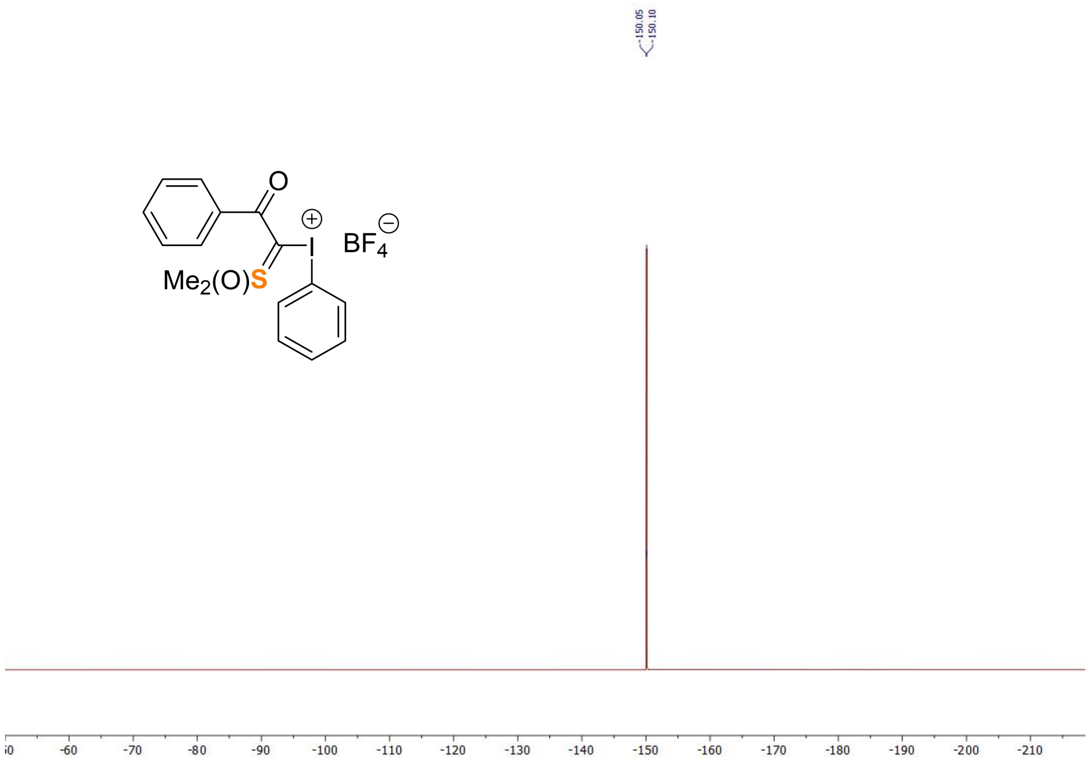

Supplementary Figure 19. **^{19}F NMR** of the **3e** (376 MHz, 25 °C in CD_3CN)

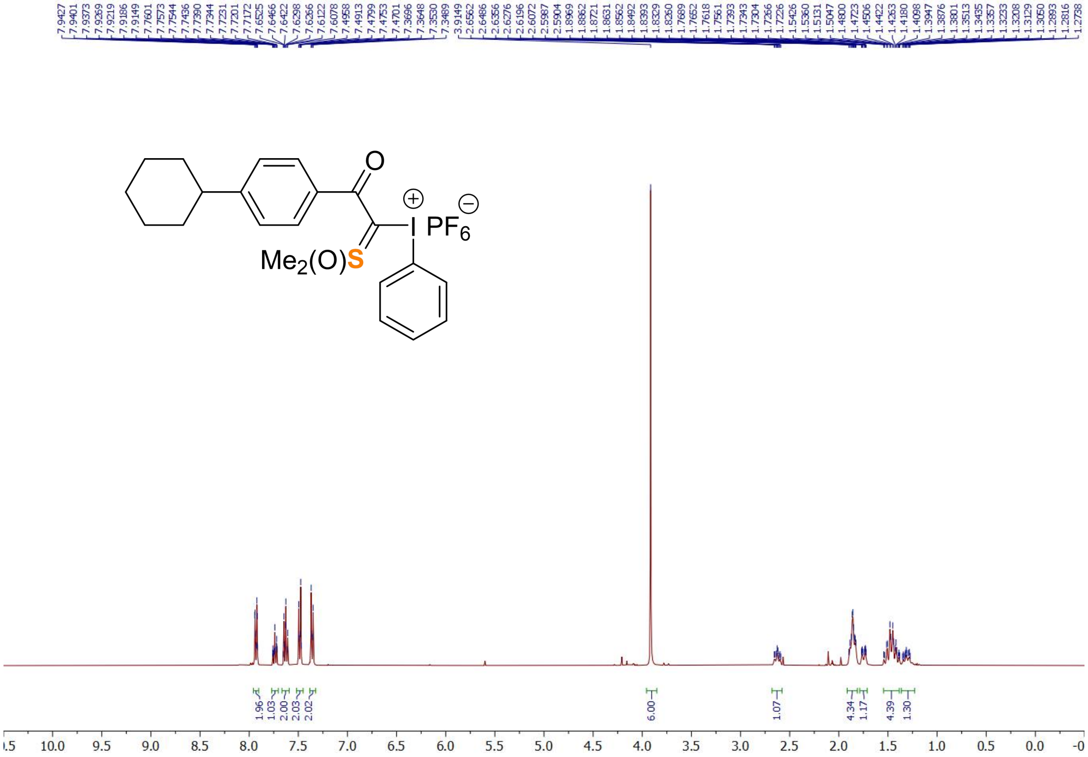

Supplementary Figure 20. ^{1}H NMR of the **3f** (400 MHz, 25 °C in Acetone-d_6)

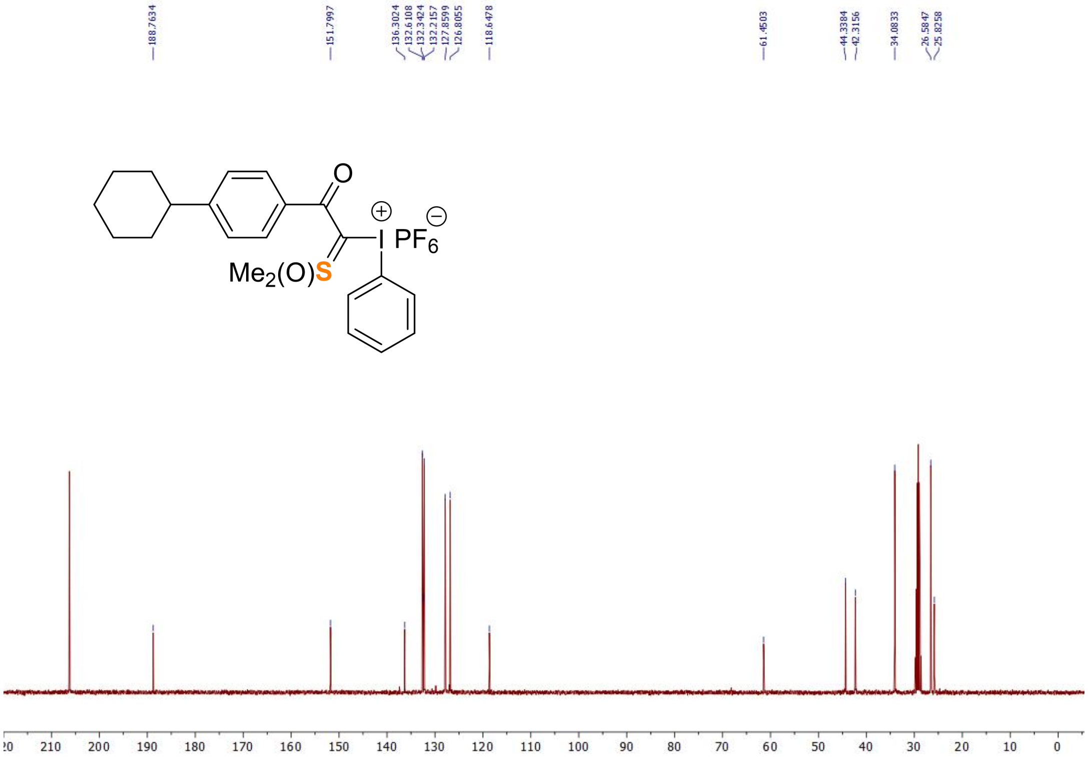

Supplementary Figure 21. **^{13}C NMR** of the **3f** (101 MHz, 25 °C in Acetone-d_6)

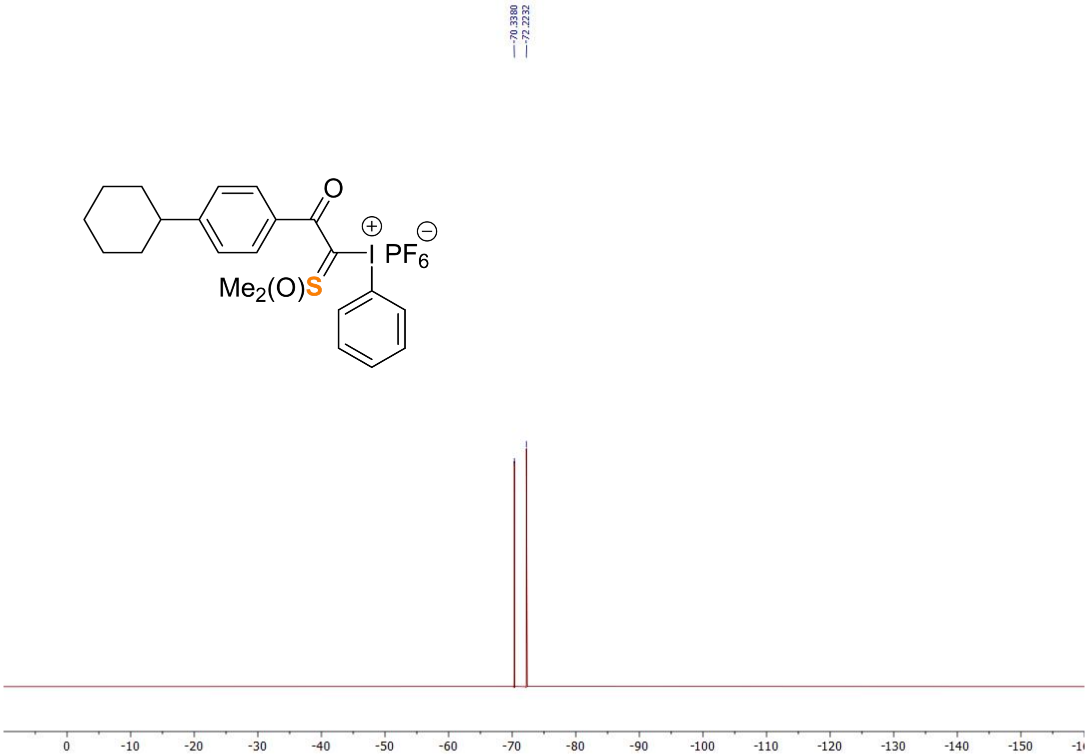

Supplementary Figure 22. **^{19}F NMR** of the **3f** (376 MHz, 25 °C in Acetone-d_6)

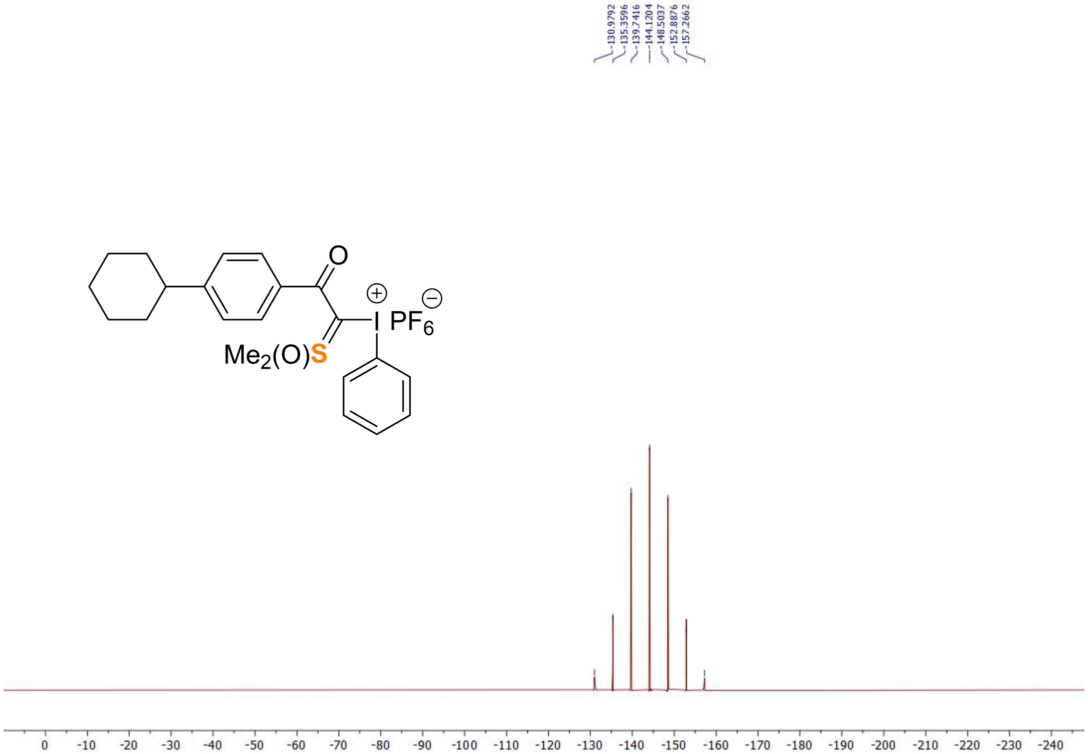

Supplementary Figure 23. ^{31}P NMR of the **3f** (162 MHz, 25 °C in Acetone-d_6)

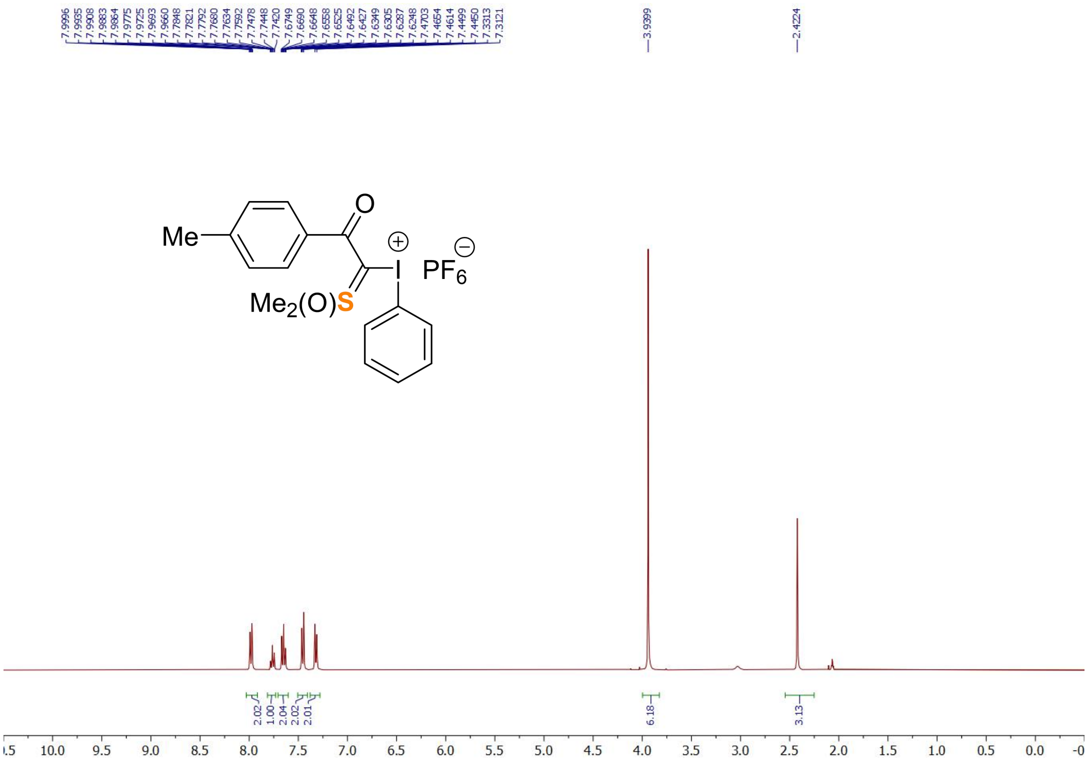

Supplementary Figure 24. ^{1}**H NMR** of the **3g** (400 MHz, 25 °C in Acetone-d_6)

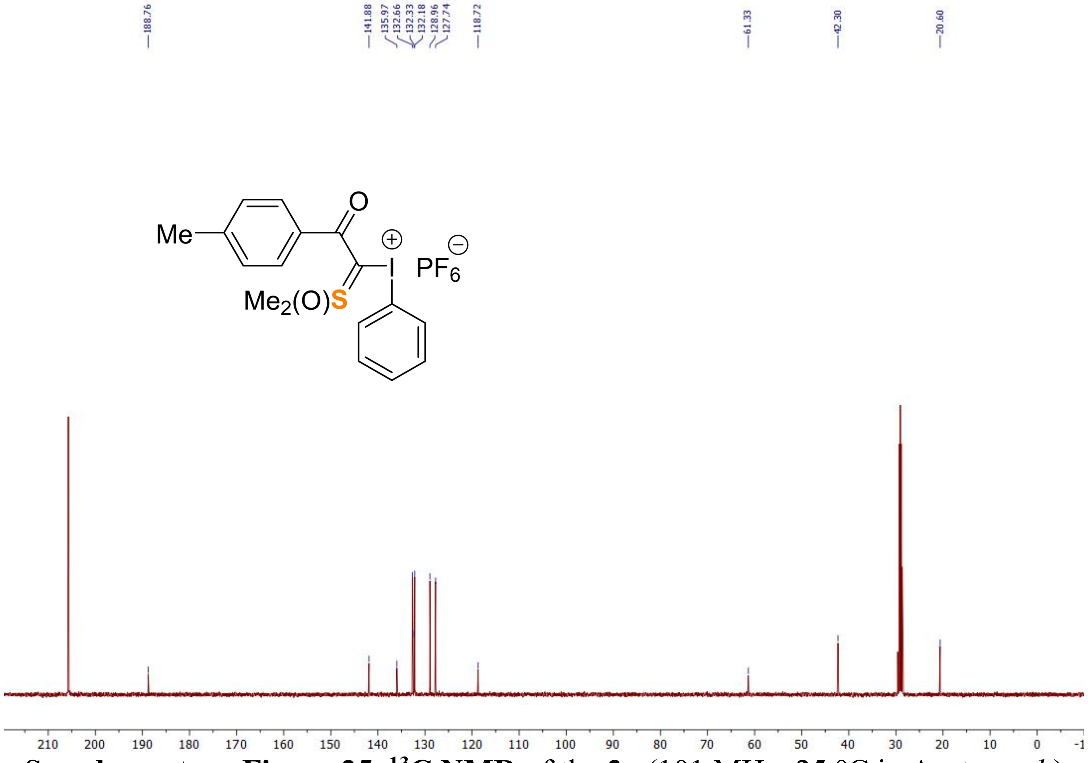

Supplementary Figure 25. ^{13}**C NMR** of the **3g** (101 MHz, 25 °C in Acetone-d_6)

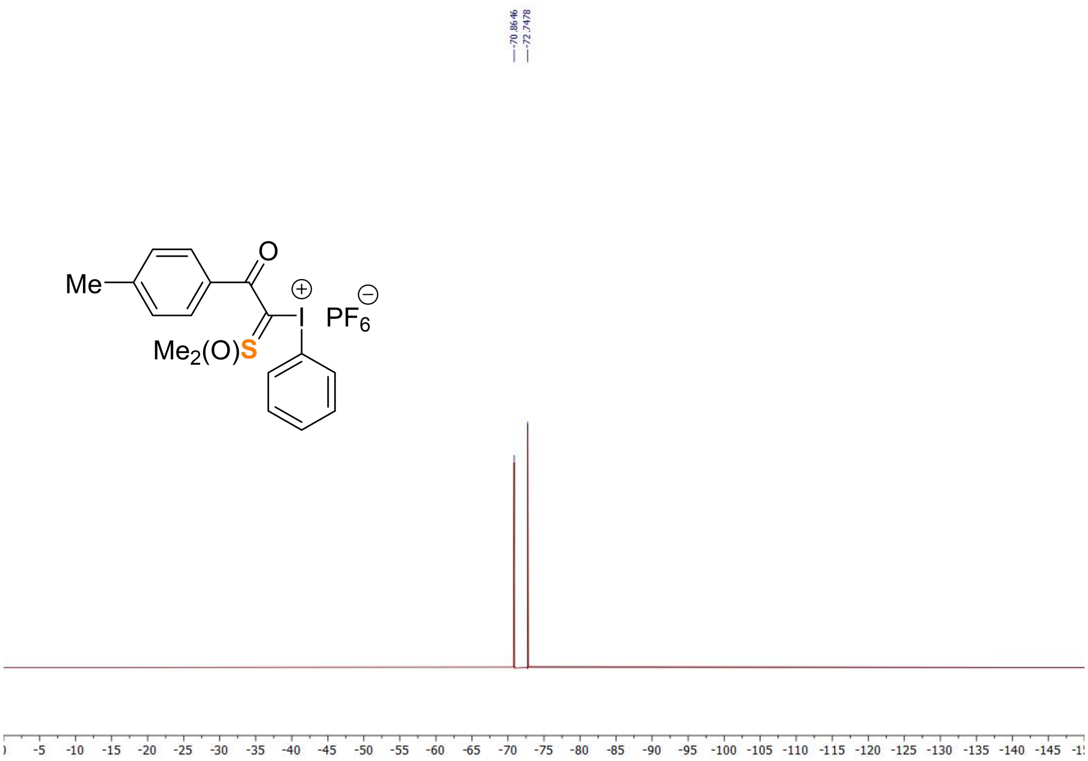

Supplementary Figure 26. ^{19}**F NMR** of the **3g** (376 MHz, 25 °C in Acetone-d_6)

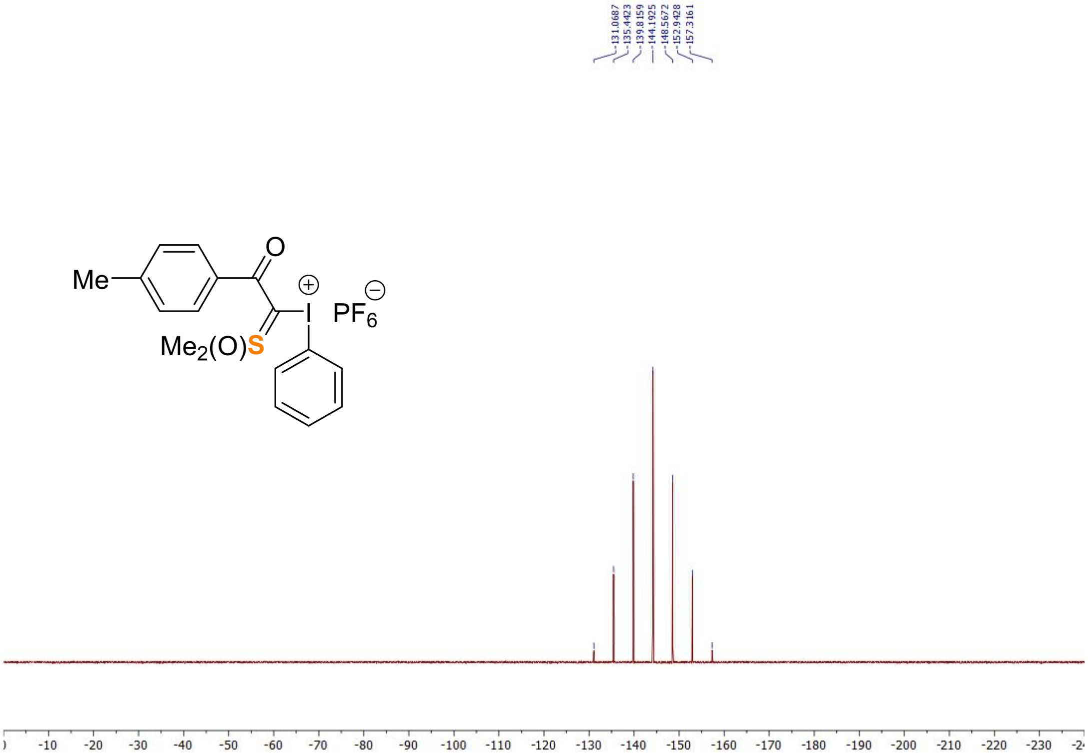

Supplementary Figure 27. ^{31}**P NMR** of the **3g** (162 MHz, 25 °C in Acetone-d_6)

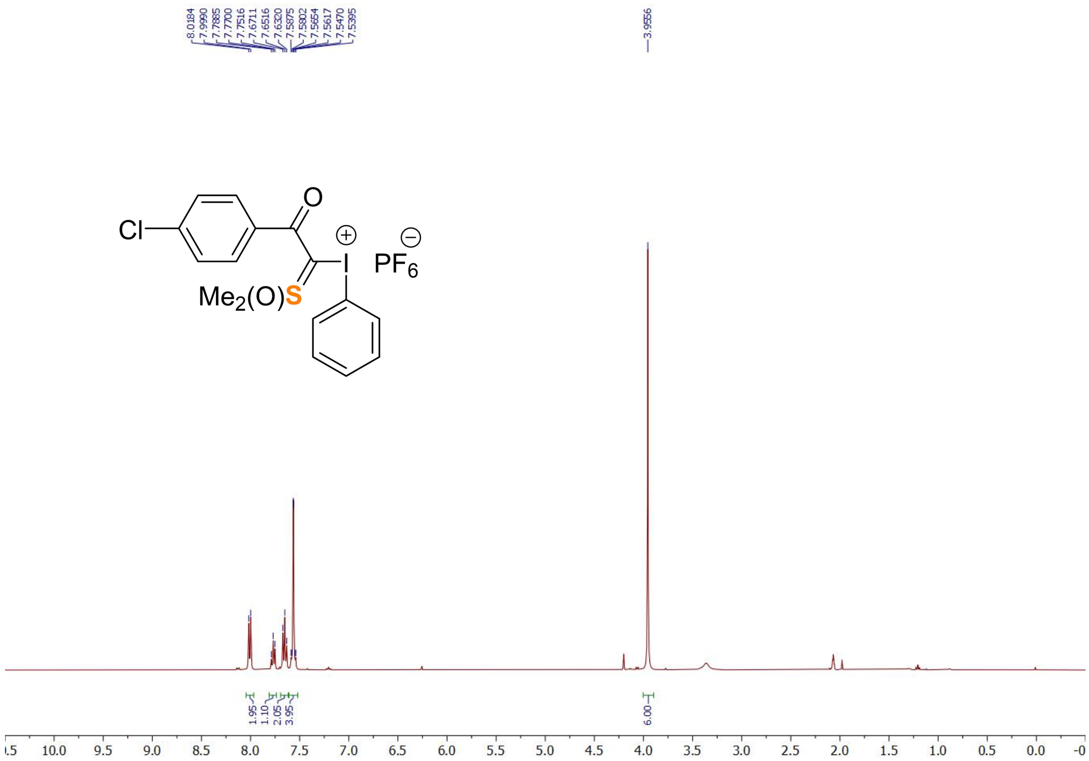

Supplementary Figure 28. **^{1}H NMR** of the **3h** (400 MHz, 25 °C in Acetone-d_6)

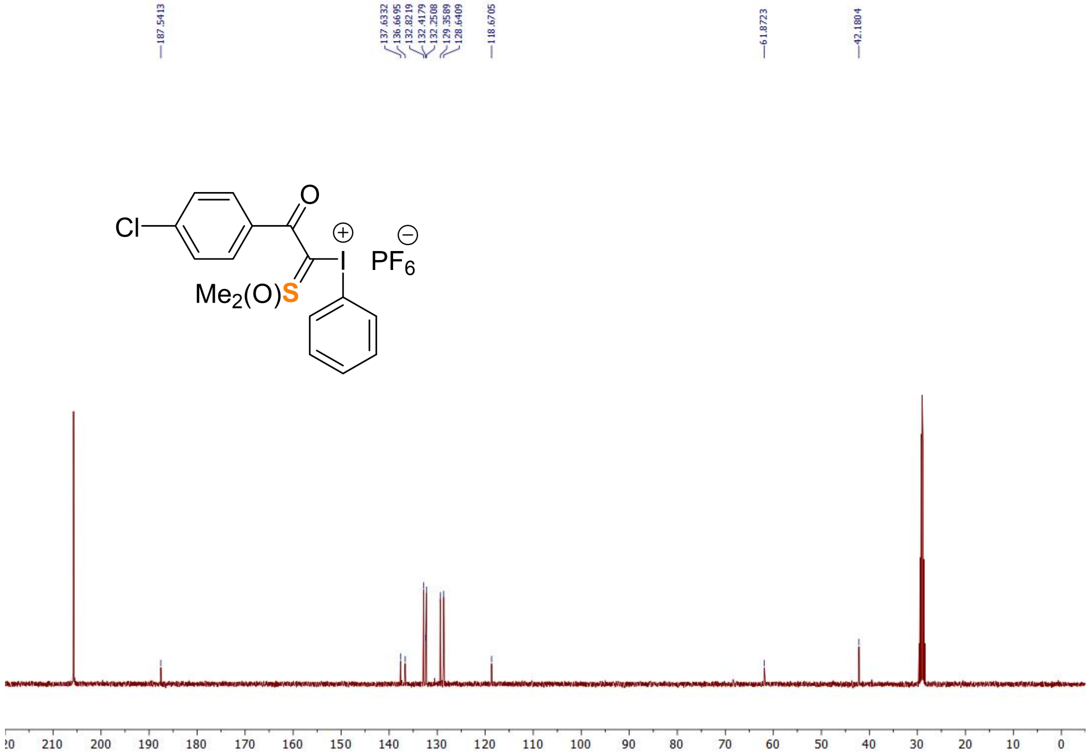

Supplementary Figure 29. ^{13}C NMR of the **3h** (101 MHz, 25 °C in Acetone-d_6)

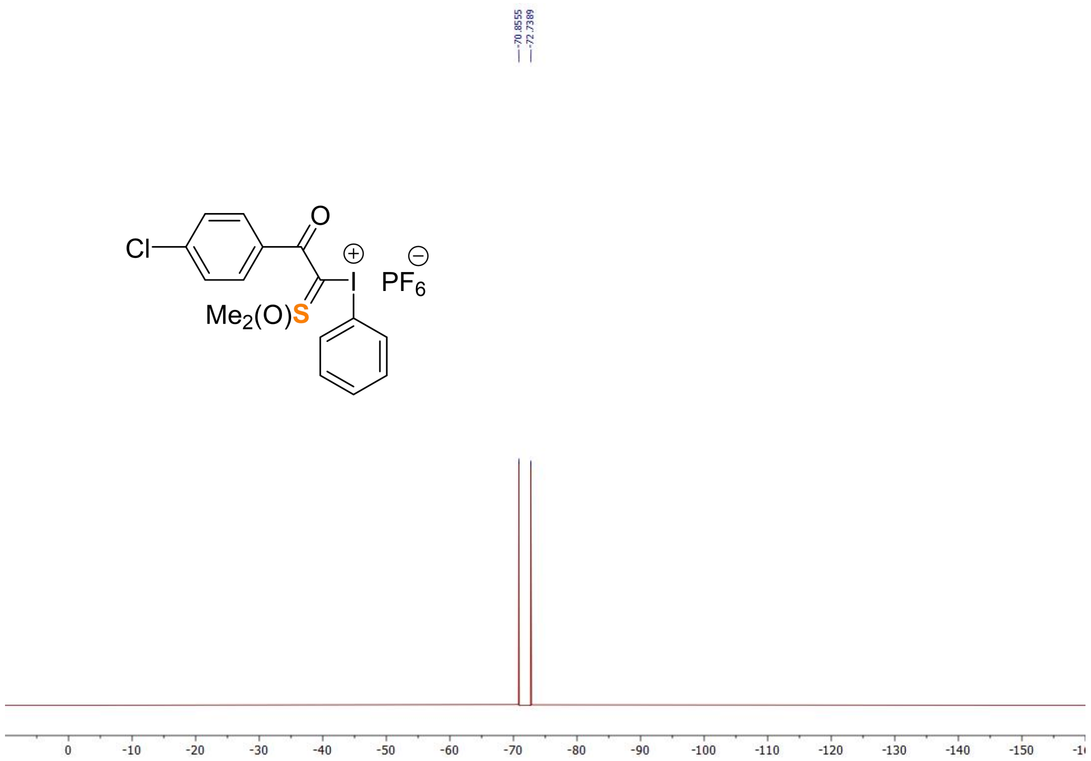

Supplementary Figure 30. ^{19}**F NMR** of the **3h** (376 MHz, 25 °C in Acetone-d_6)

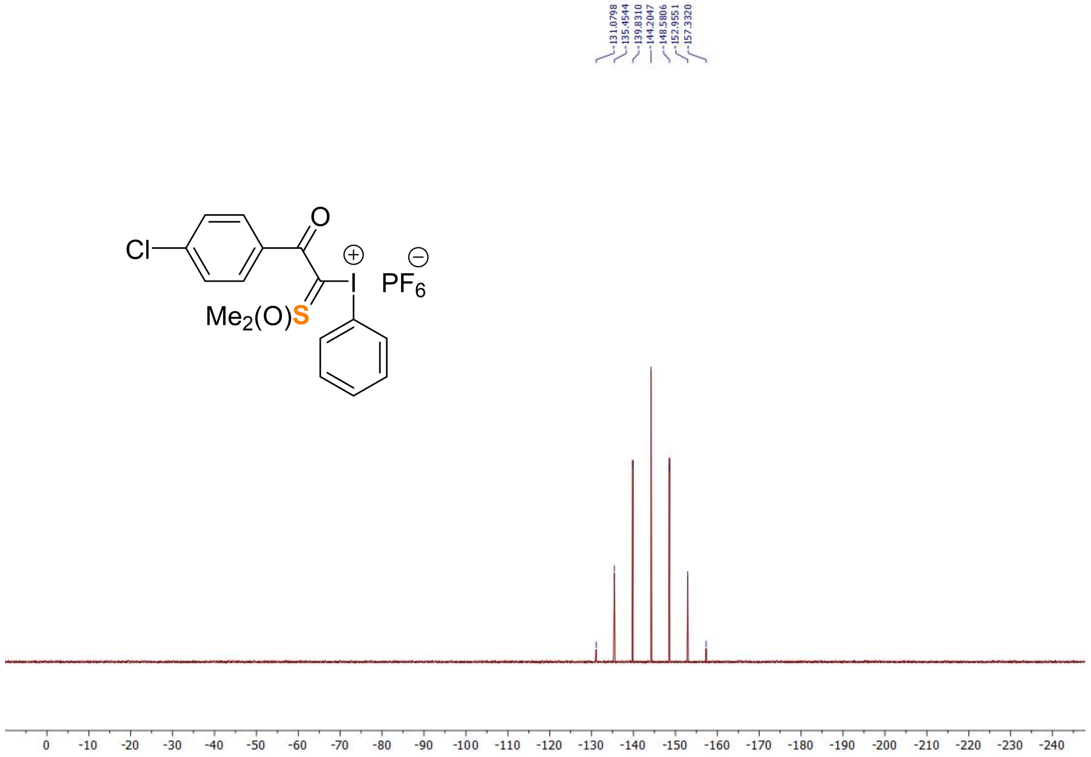

Supplementary Figure 31. ^{31}**P NMR** of the **3h** (162 MHz, 25 °C in Acetone-d_6)

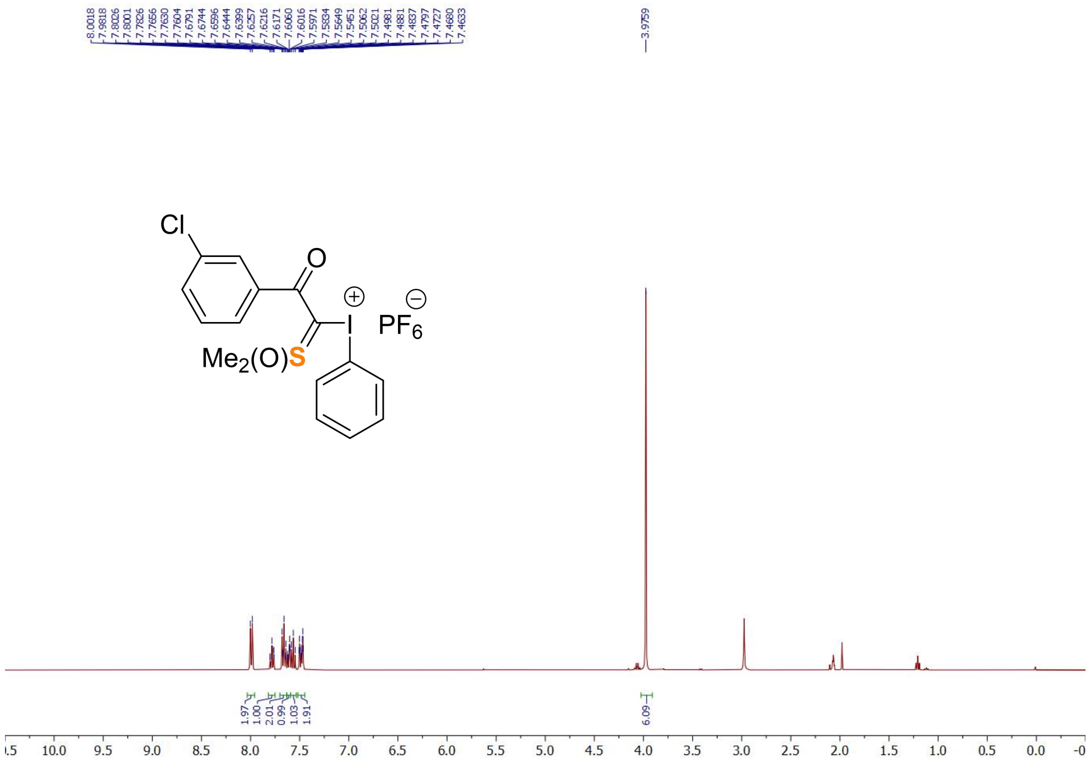

Supplementary Figure 32. ^{1}H NMR of the **3i** (400 MHz, 25 °C in Acetone-d_6)

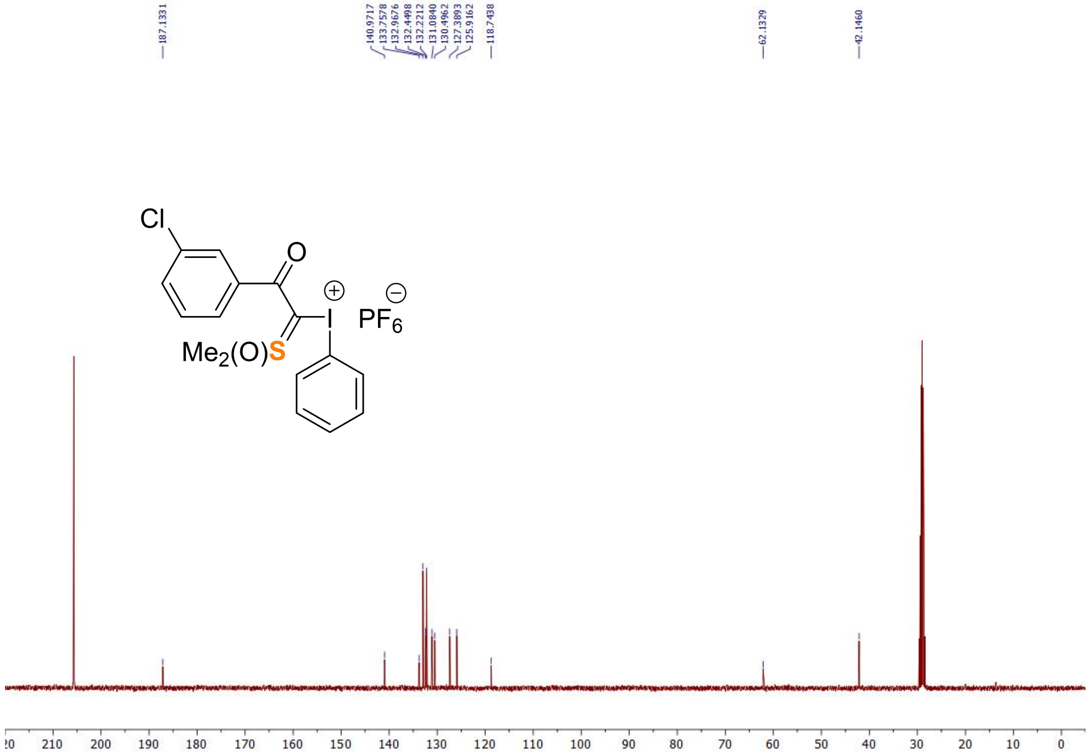

Supplementary Figure 33. ^{13}C NMR of the **3i** (101 MHz, 25 °C in Acetone-d_6)

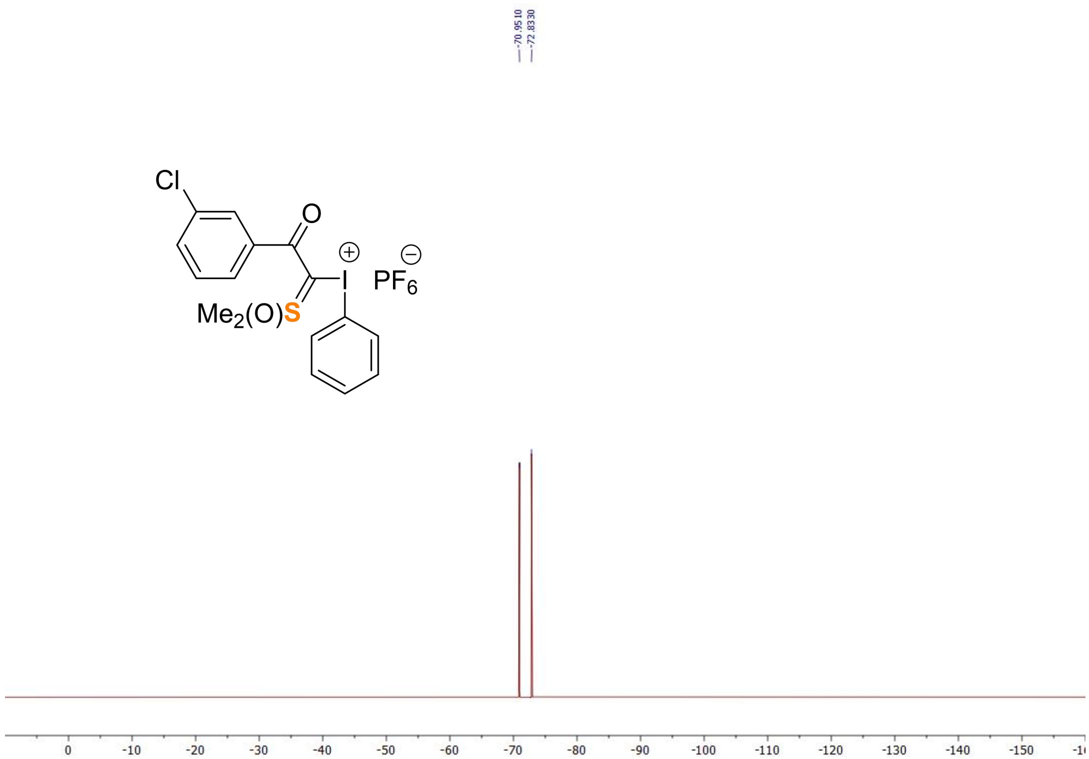

Supplementary Figure 34. ^{19}F NMR of the **3i** (376 MHz, 25 °C in Acetone-d_6)

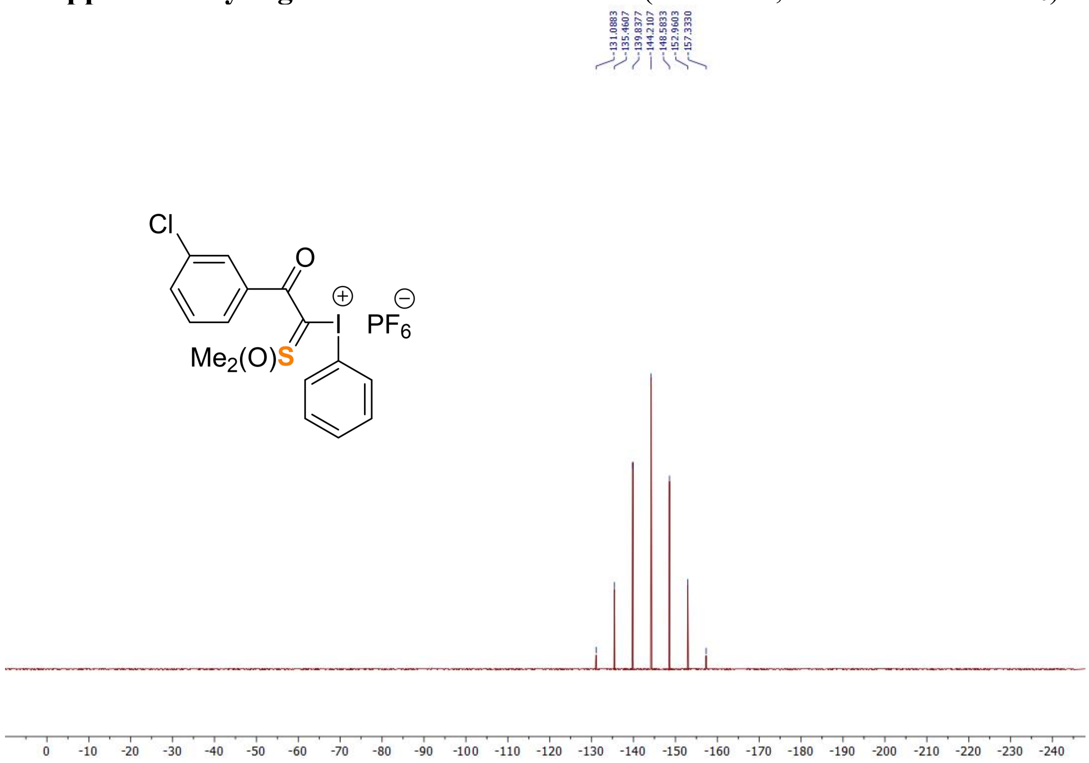

Supplementary Figure 35. ^{31}P NMR of the **3i** (162 MHz, 25 °C in Acetone-d_6)

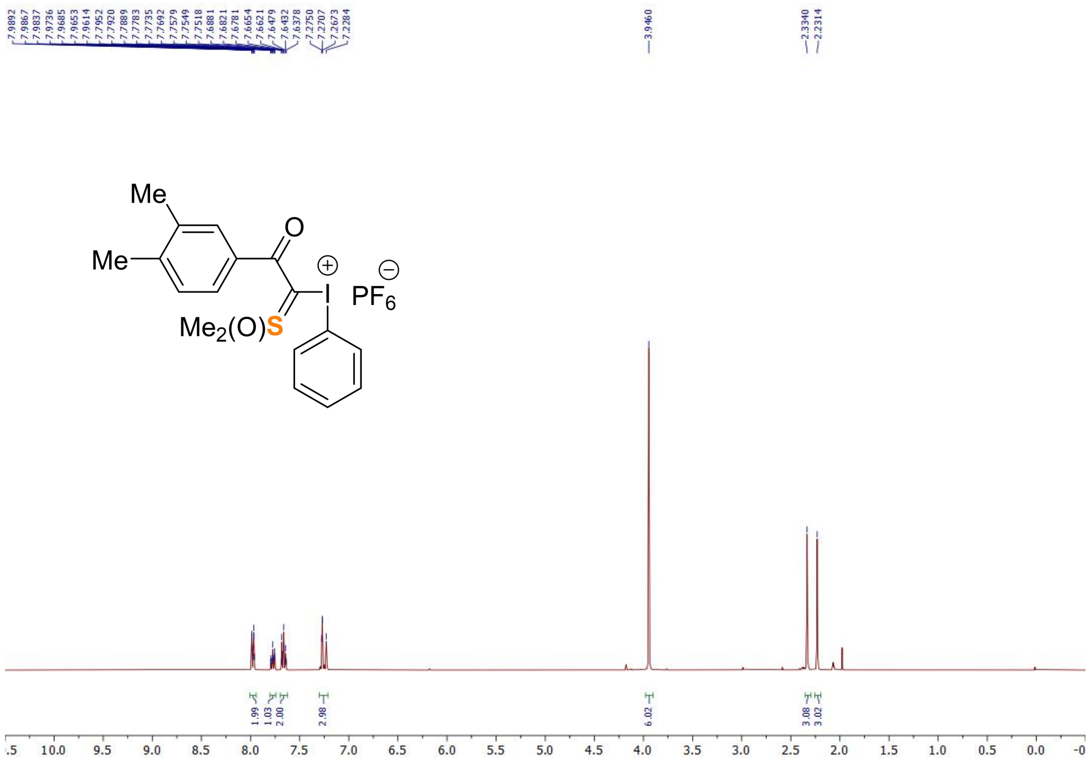

Supplementary Figure 36. ^{1}H NMR of the **3j** (400 MHz, 25 °C in Acetone-*d*$_6$)

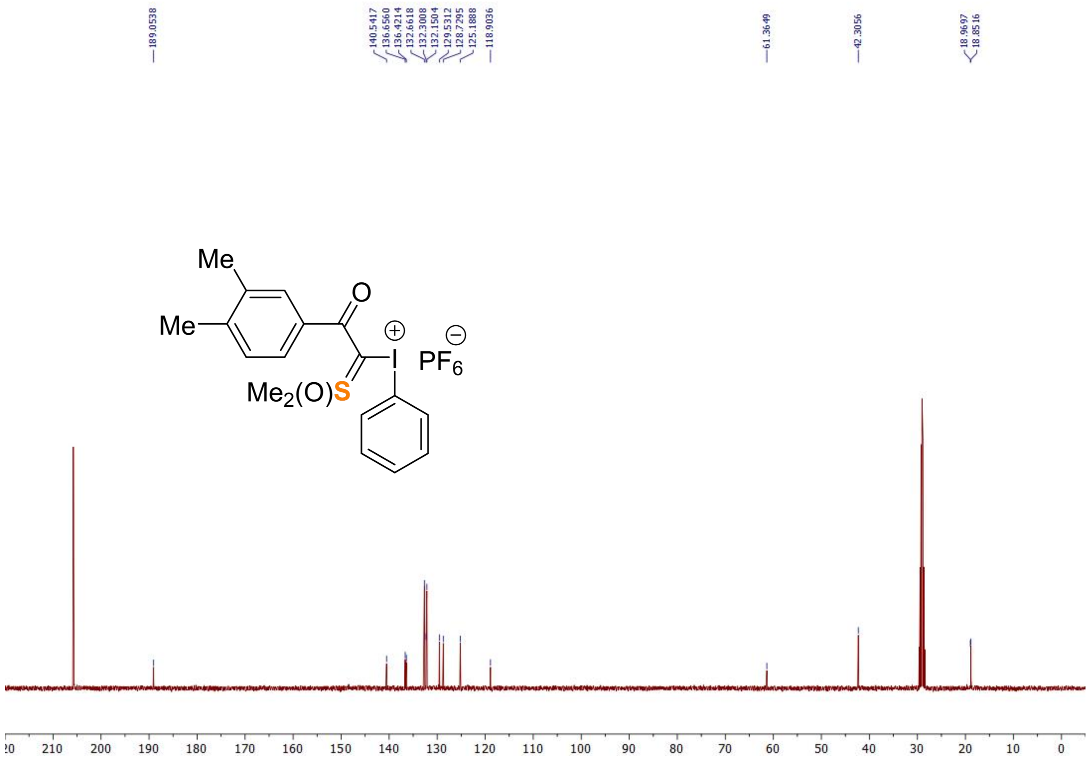

Supplementary Figure 37. ^{13}C NMR of the **3j** (101 MHz, 25 °C in Acetone-*d*$_6$)

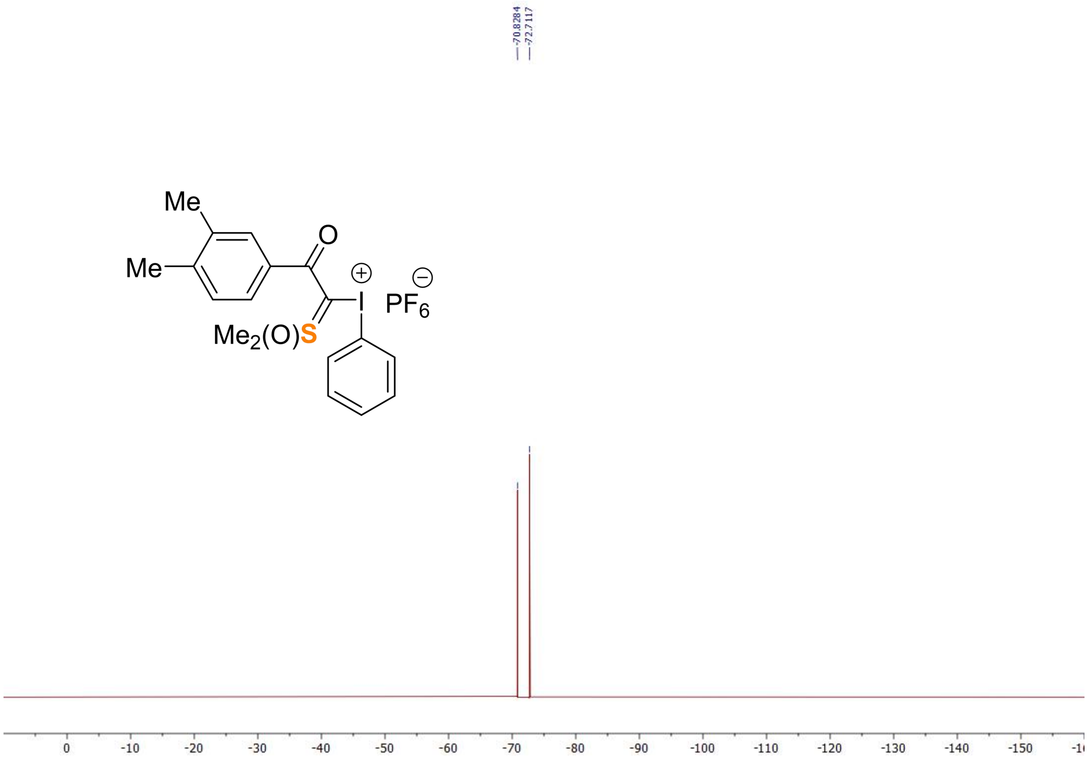

Supplementary Figure 38. ^{19}**F NMR** of the **3j** (376 MHz, 25 °C in Acetone-d_6)

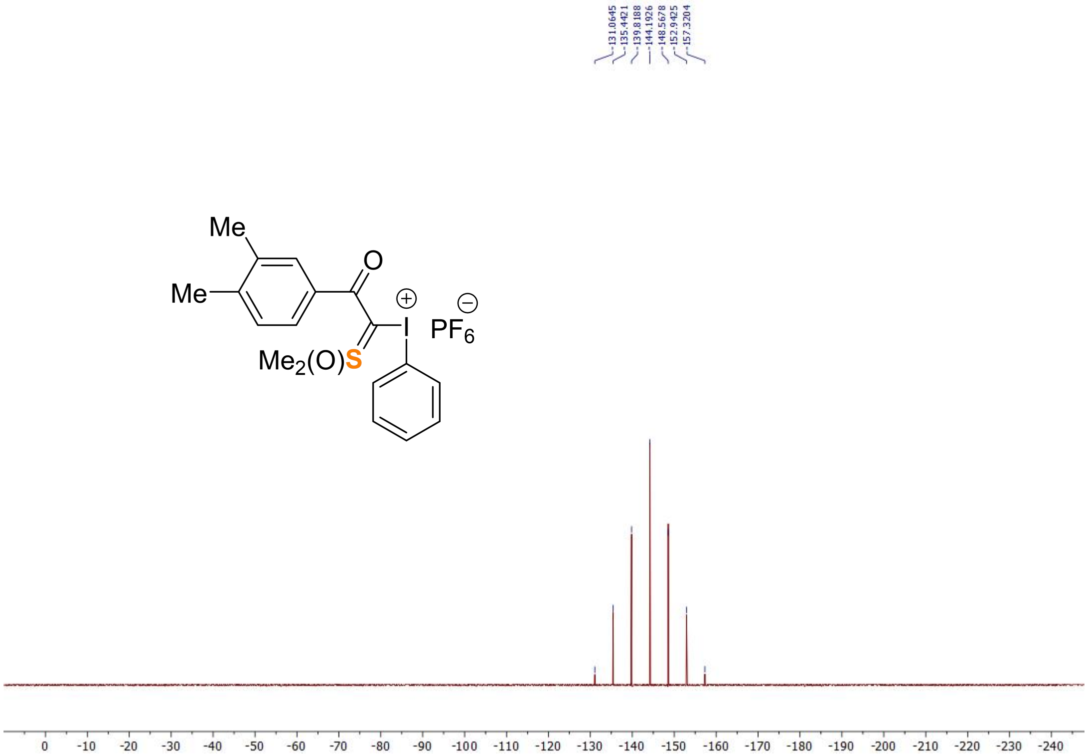

Supplementary Figure 39. ^{31}**P NMR** of the **3j** (162 MHz, 25 °C in Acetone-d_6)

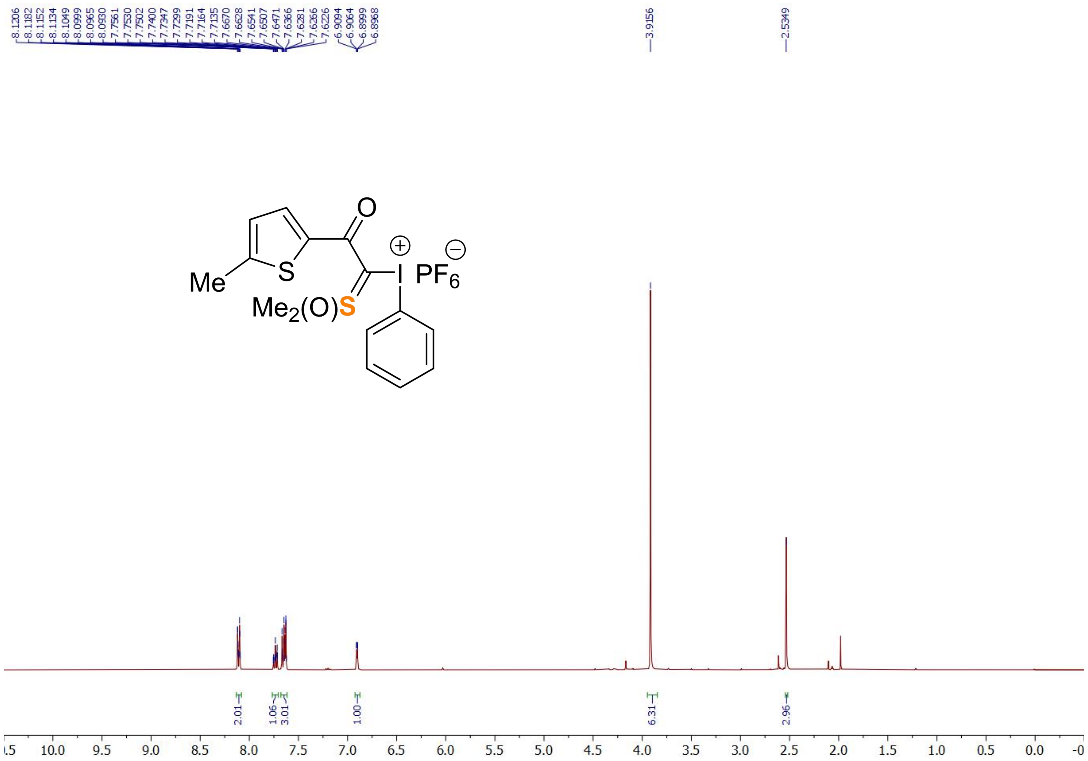

Supplementary Figure 40. ^{1}H NMR of the **3k** (400 MHz, 25 °C in Acetone-*d*$_6$)

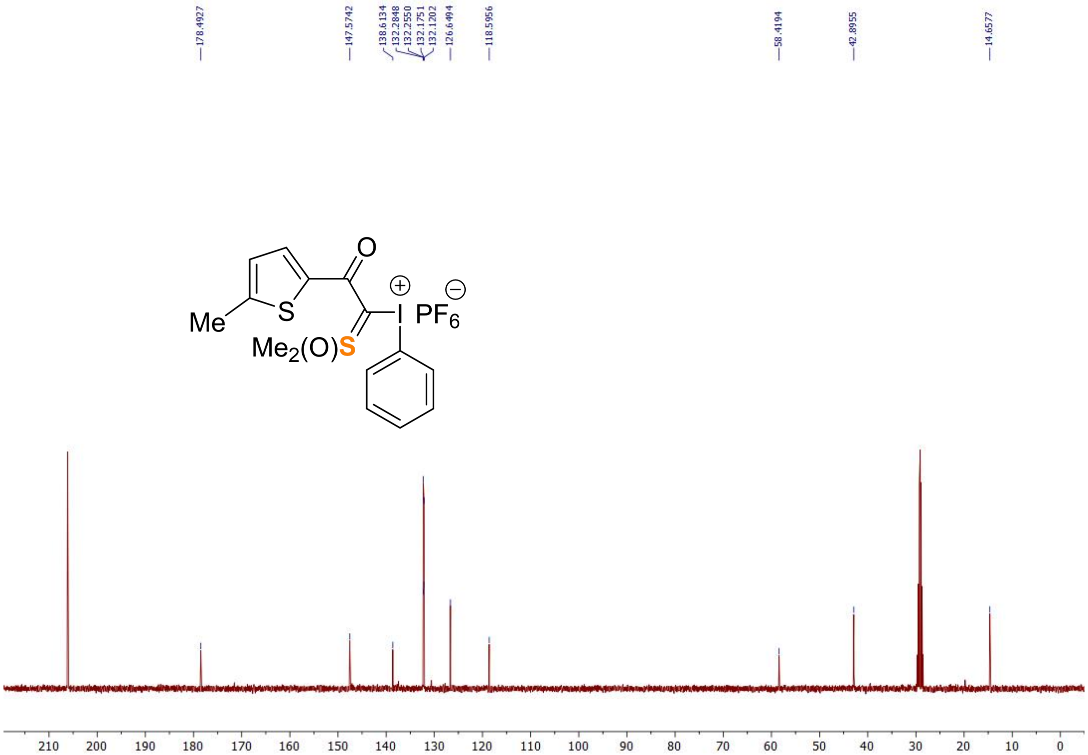

Supplementary Figure 41. ^{13}C NMR of the **3k** (101 MHz, 25 °C in Acetone-*d*$_6$)

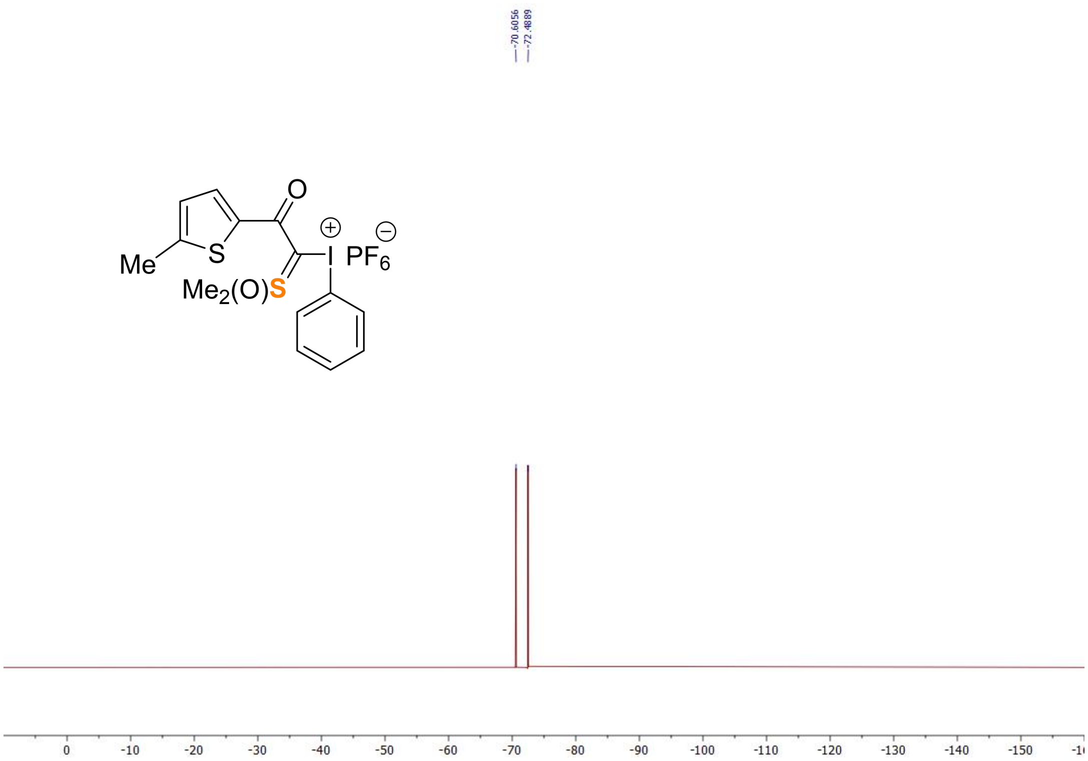

Supplementary Figure 42. ^{19}**F NMR** of the **3k** (376 MHz, 25 °C in Acetone-d_6)

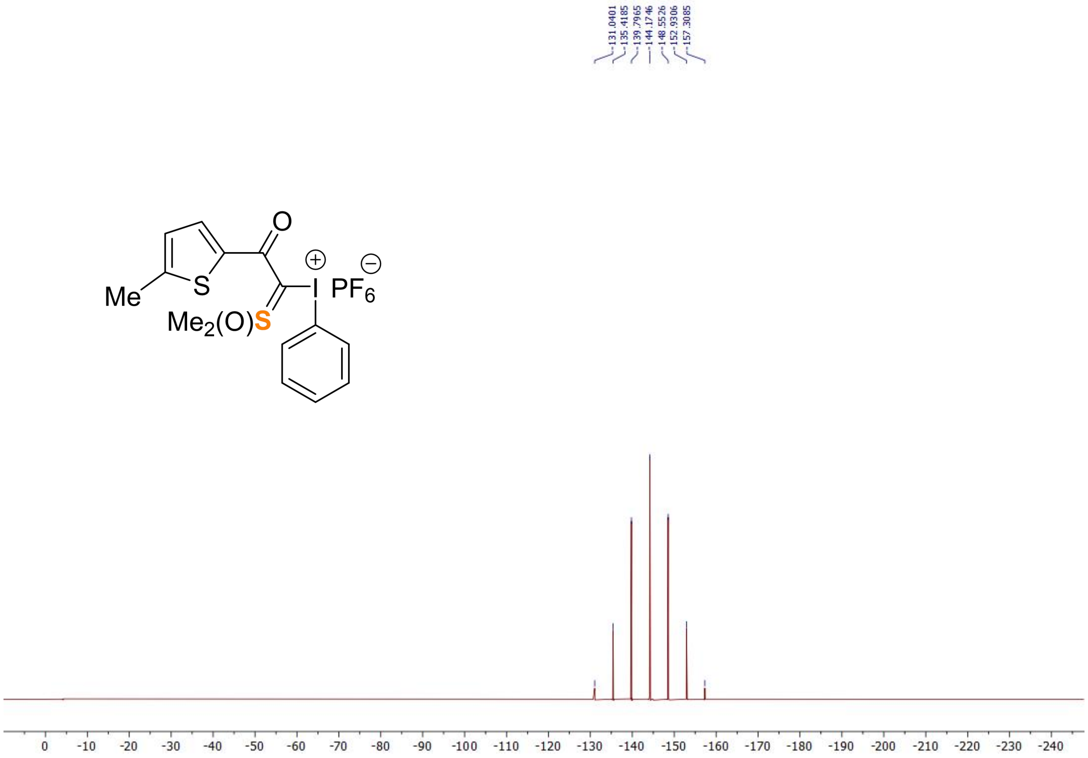

Supplementary Figure 43. ^{31}**P NMR** of the **3k** (162 MHz, 25 °C in Acetone-d_6)

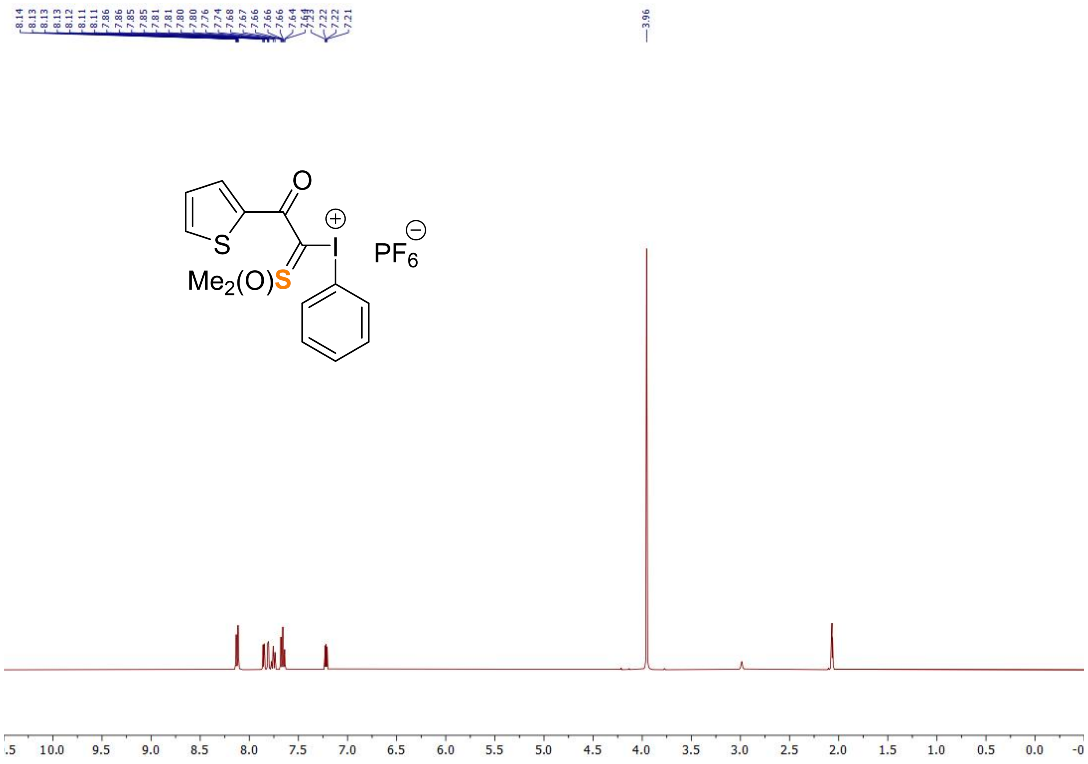

Supplementary Figure 44. ^{1}**H NMR** of the **3l** (400 MHz, 25 °C in Acetone-d_6)

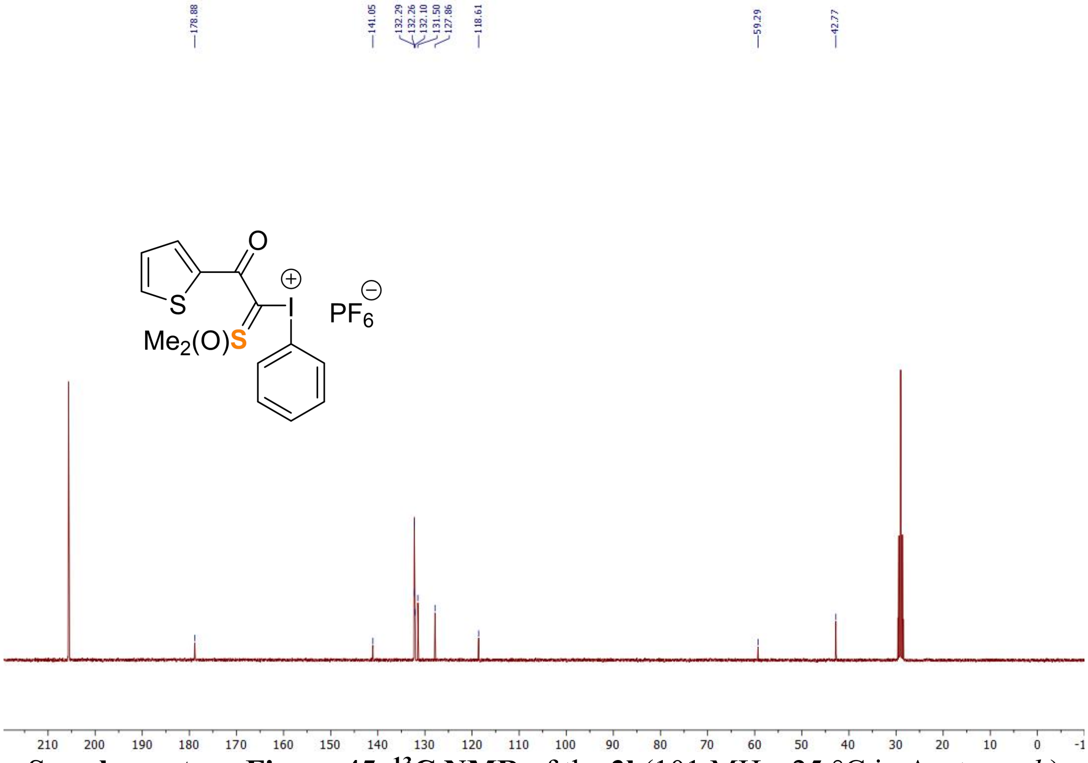

Supplementary Figure 45. ^{13}**C NMR** of the **3l** (101 MHz, 25 °C in Acetone-d_6)

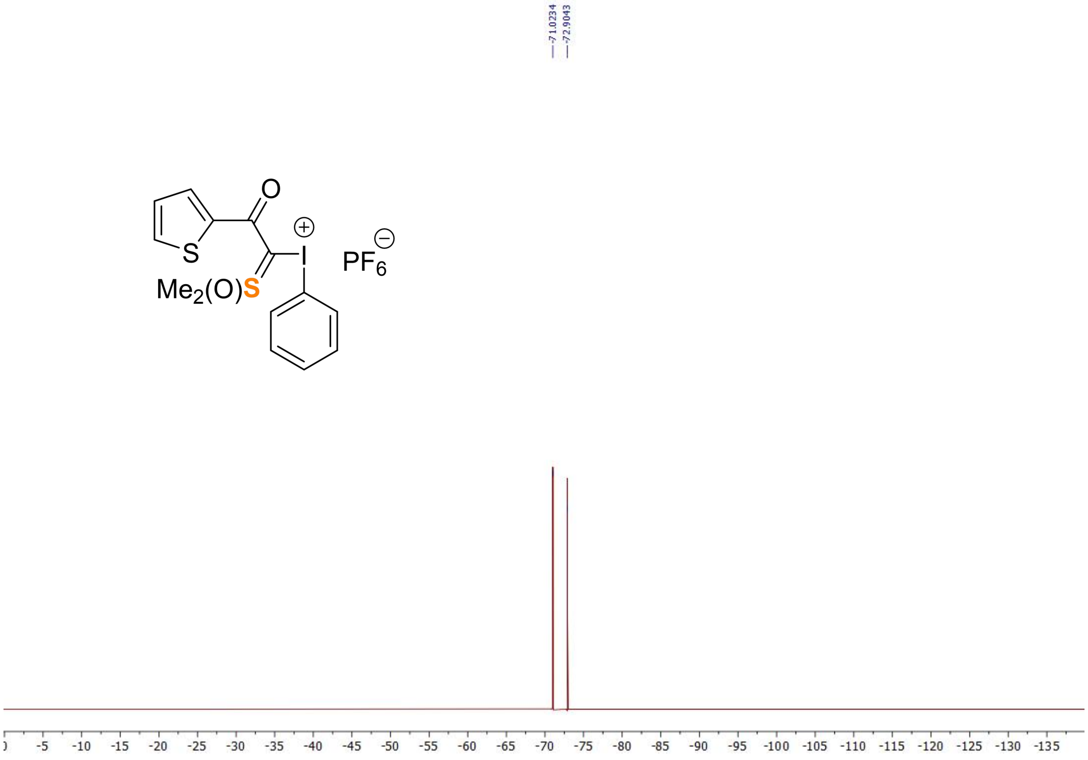

Supplementary Figure 46. ^{19}F NMR of the **3l** (376 MHz, 25 °C in Acetone-d_6)

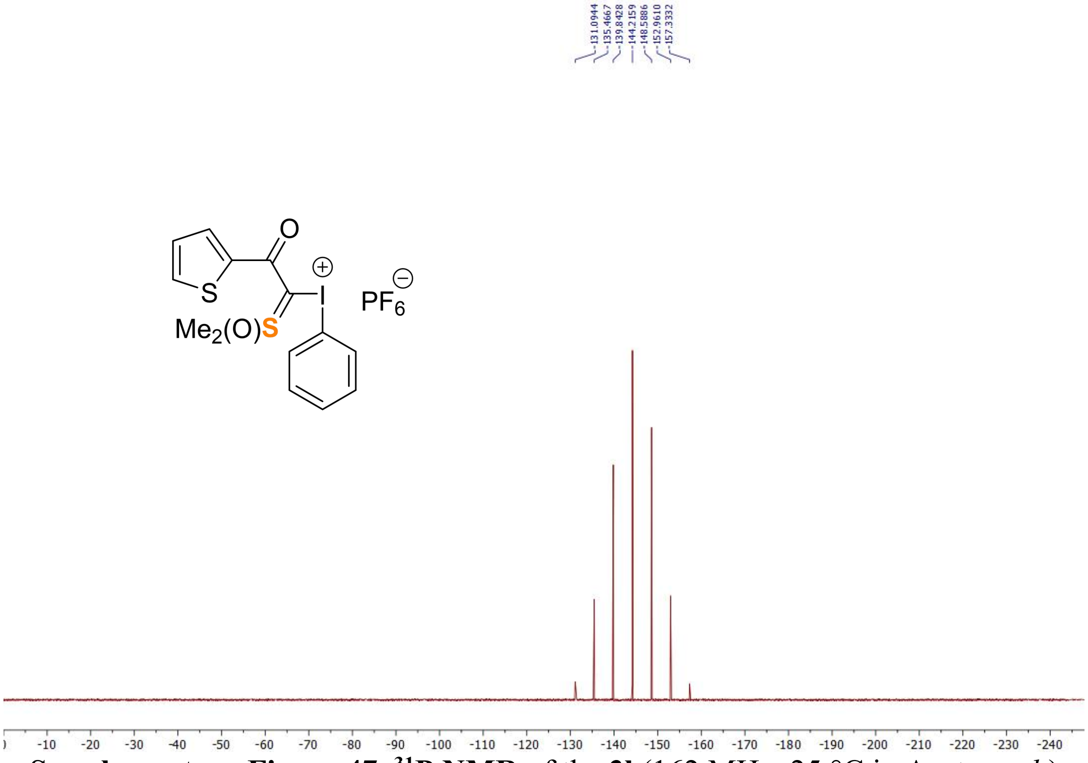

Supplementary Figure 47. ^{31}P NMR of the **3l** (162 MHz, 25 °C in Acetone-d_6)

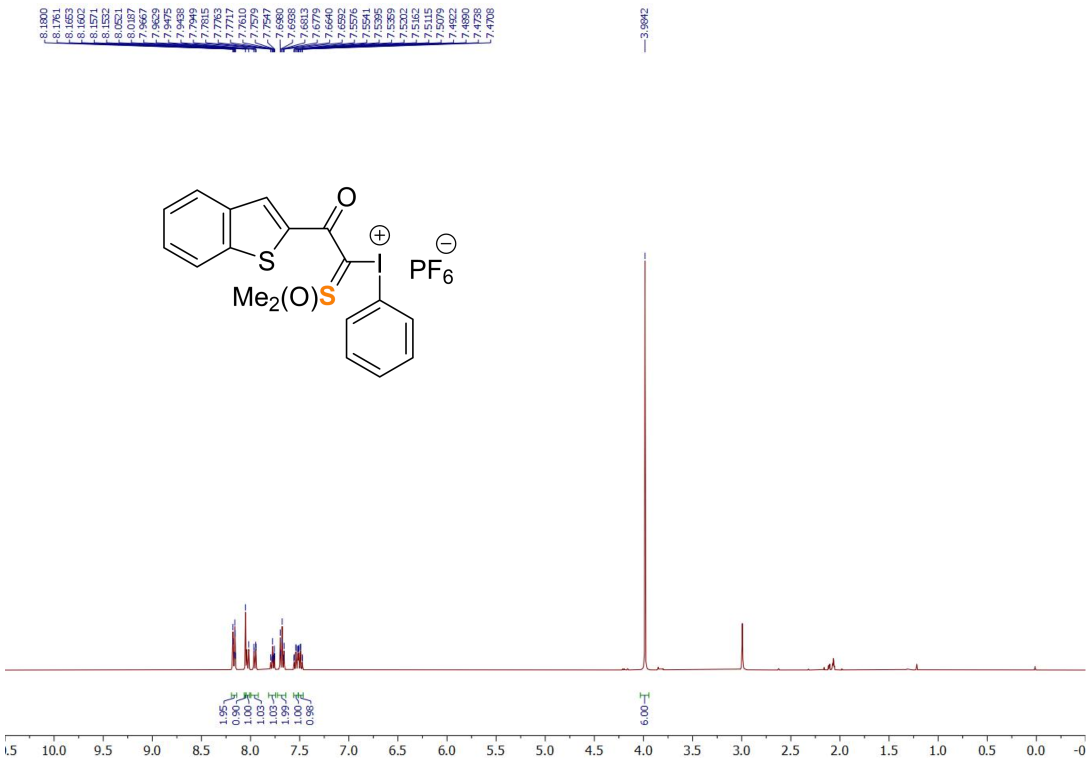

Supplementary Figure 48. **^{1}H NMR** of the **3m** (400 MHz, 25 °C in Acetone-d_6)

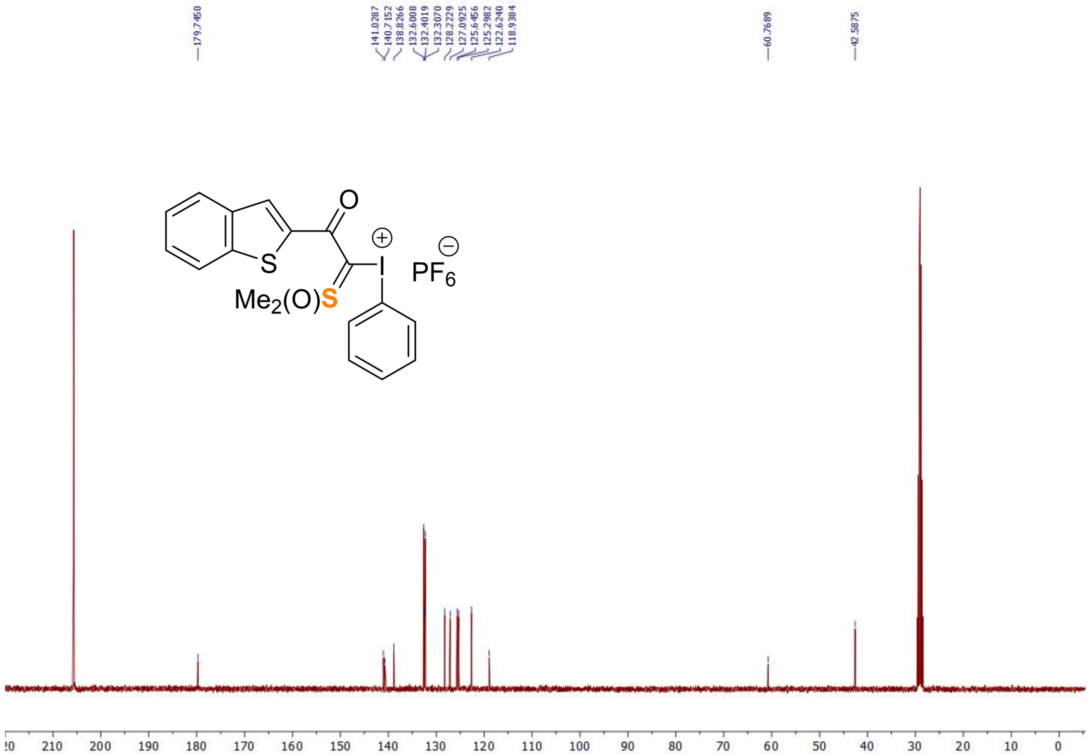

Supplementary Figure 49. **^{13}C NMR** of the **3m** (101 MHz, 25 °C in Acetone-d_6)

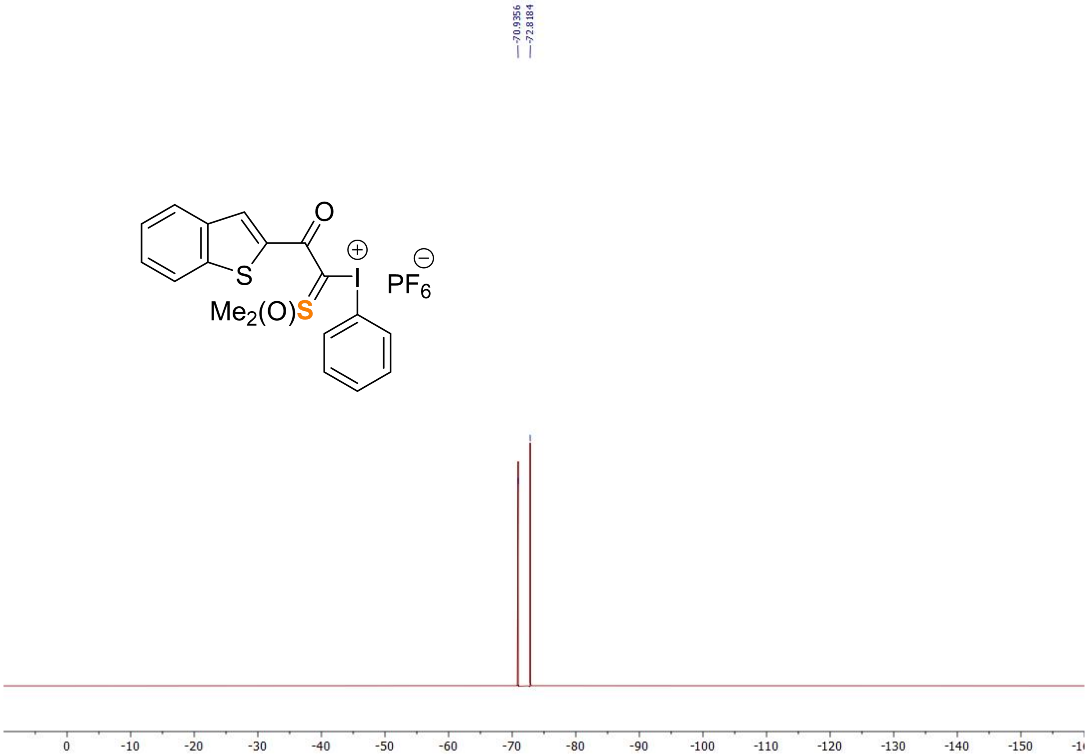

Supplementary Figure 50. ^{19}**F NMR** of the **3m** (376 MHz, 25 °C in Acetone-d_6)

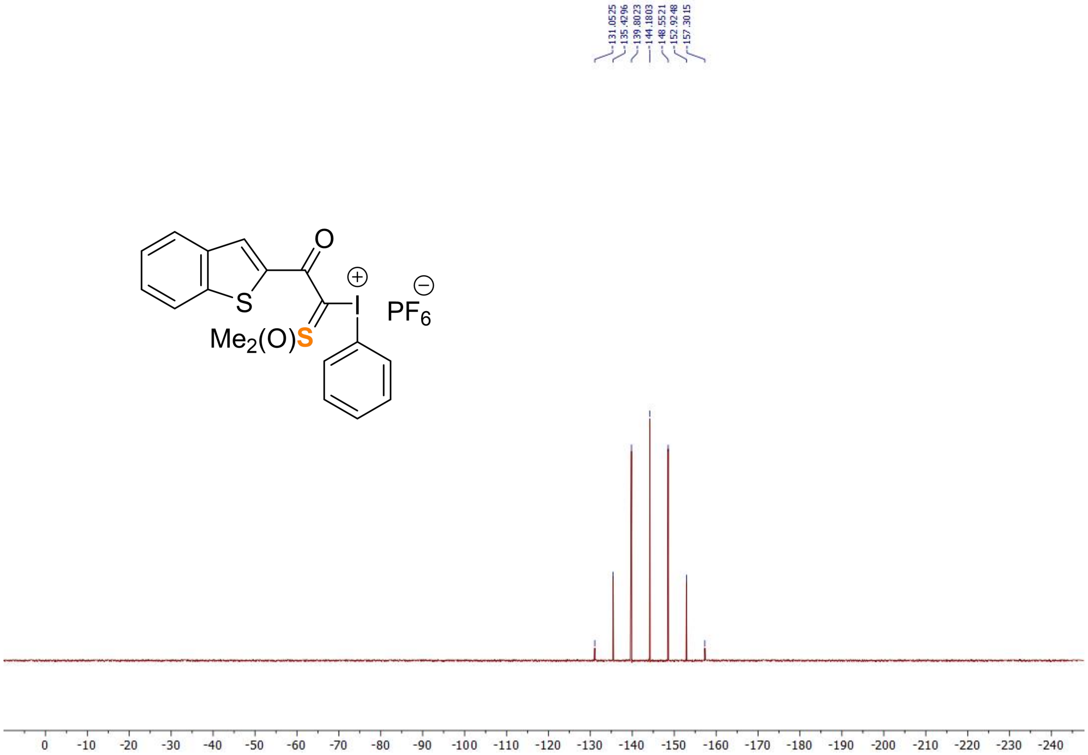

Supplementary Figure 51. ^{31}**P NMR** of the **3m** (162 MHz, 25 °C in Acetone-d_6)

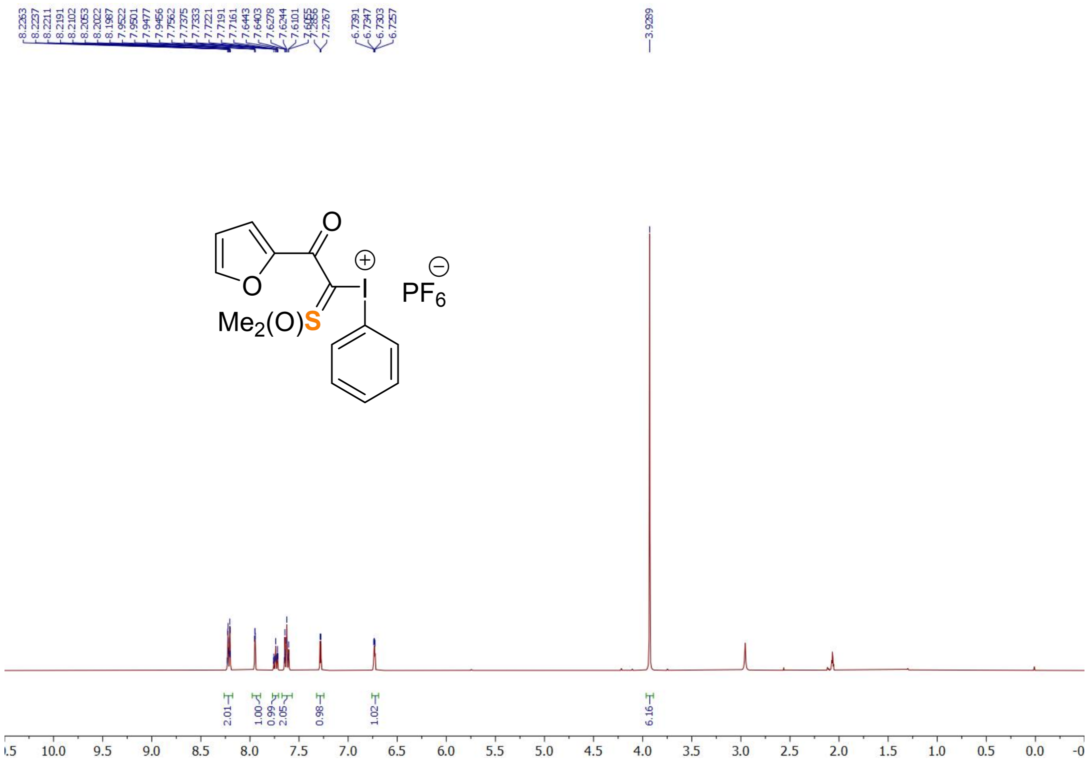

Supplementary Figure 52. **^{1}H NMR** of the **3n** (400 MHz, 25 °C in Acetone-d_6)

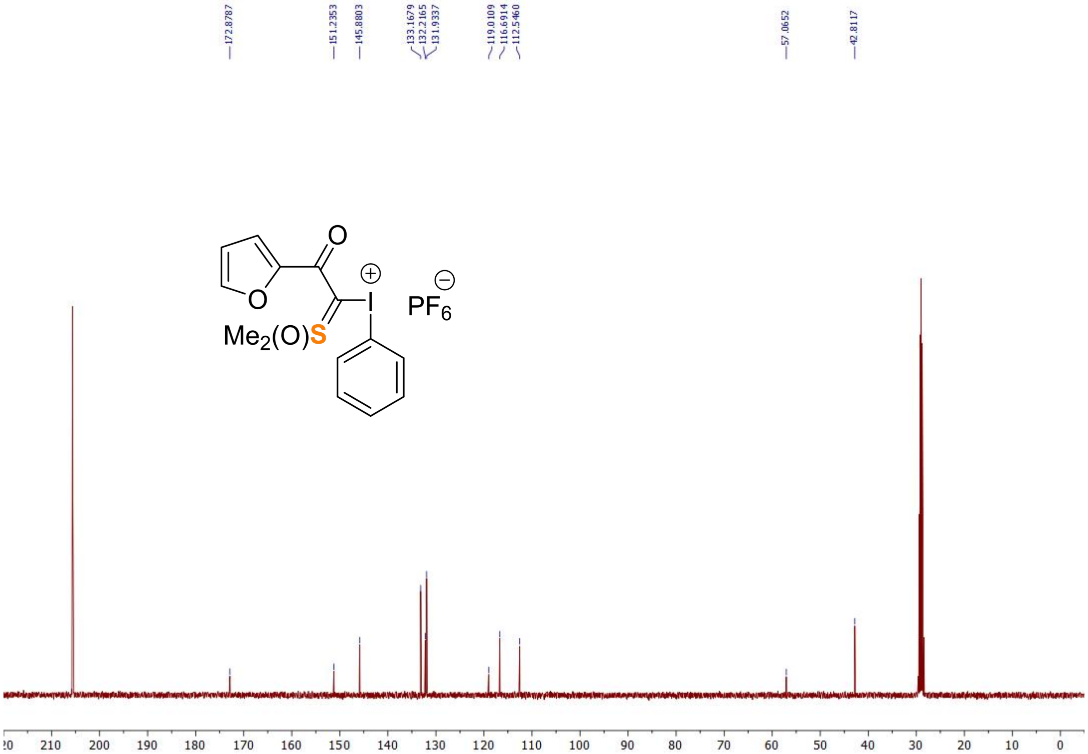

Supplementary Figure 53. **^{13}C NMR** of the **3n** (101 MHz, 25 °C in Acetone-d_6)

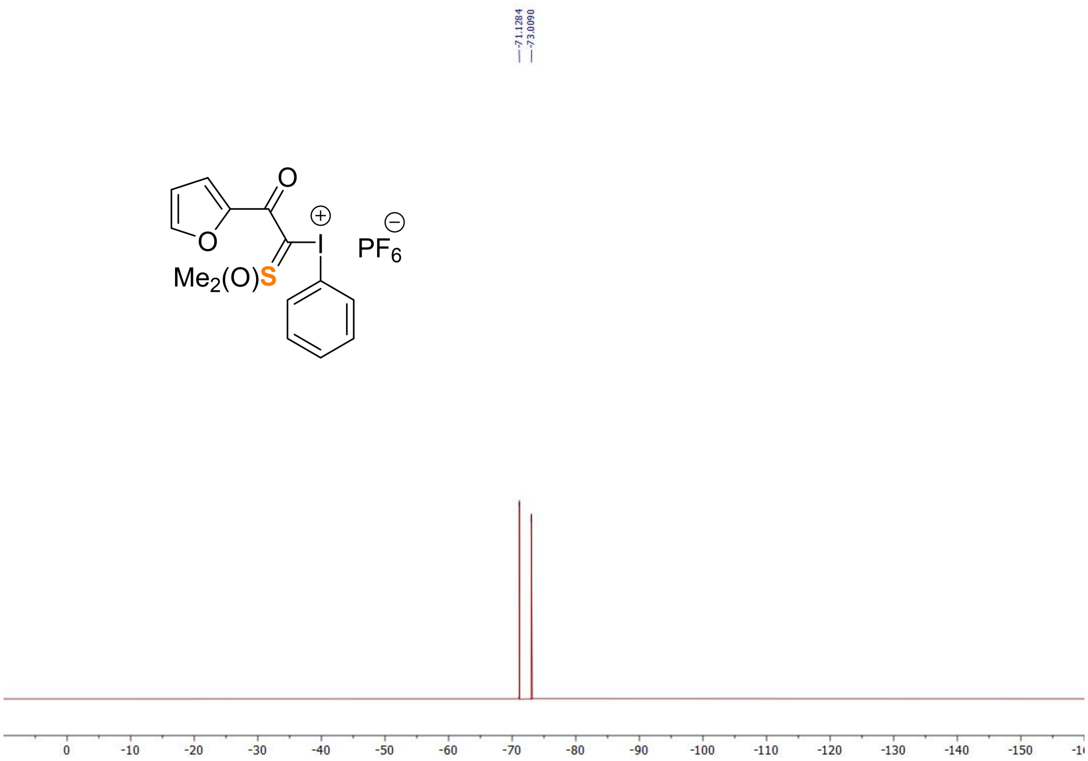

Supplementary Figure 54. ^{19}**F NMR** of the **3n** (376 MHz, 25 °C in Acetone-d_6)

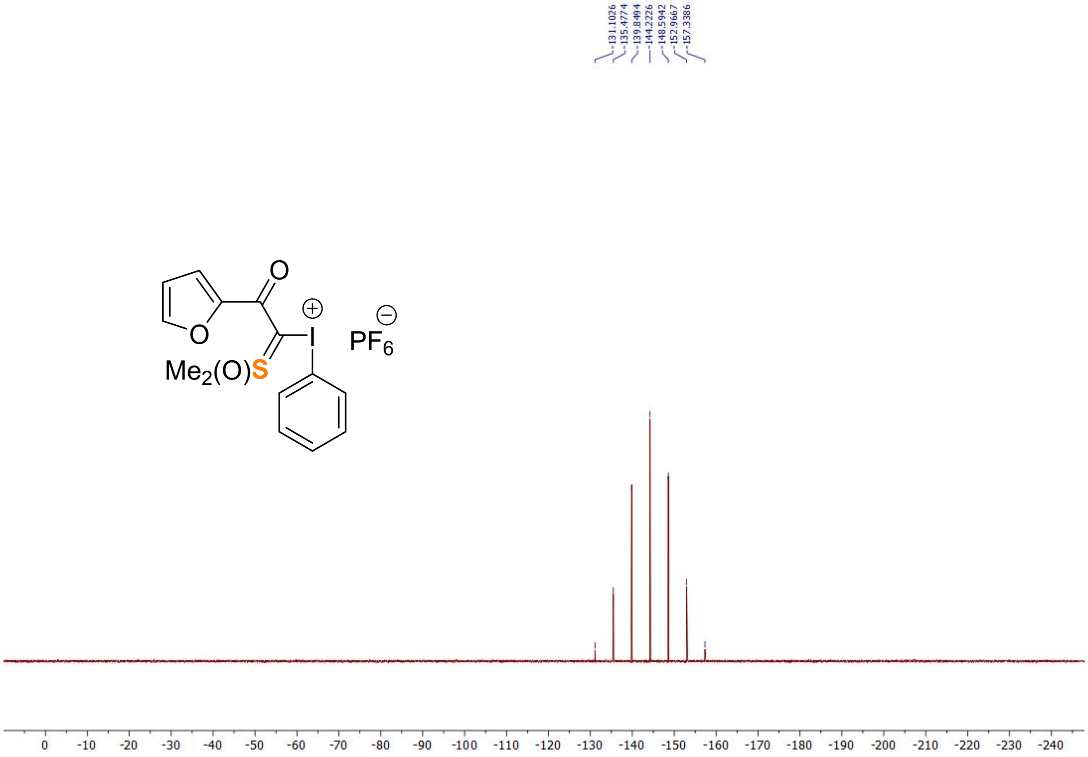

Supplementary Figure 55. ^{31}**P NMR** of the **3n** (162 MHz, 25 °C in Acetone-d_6)

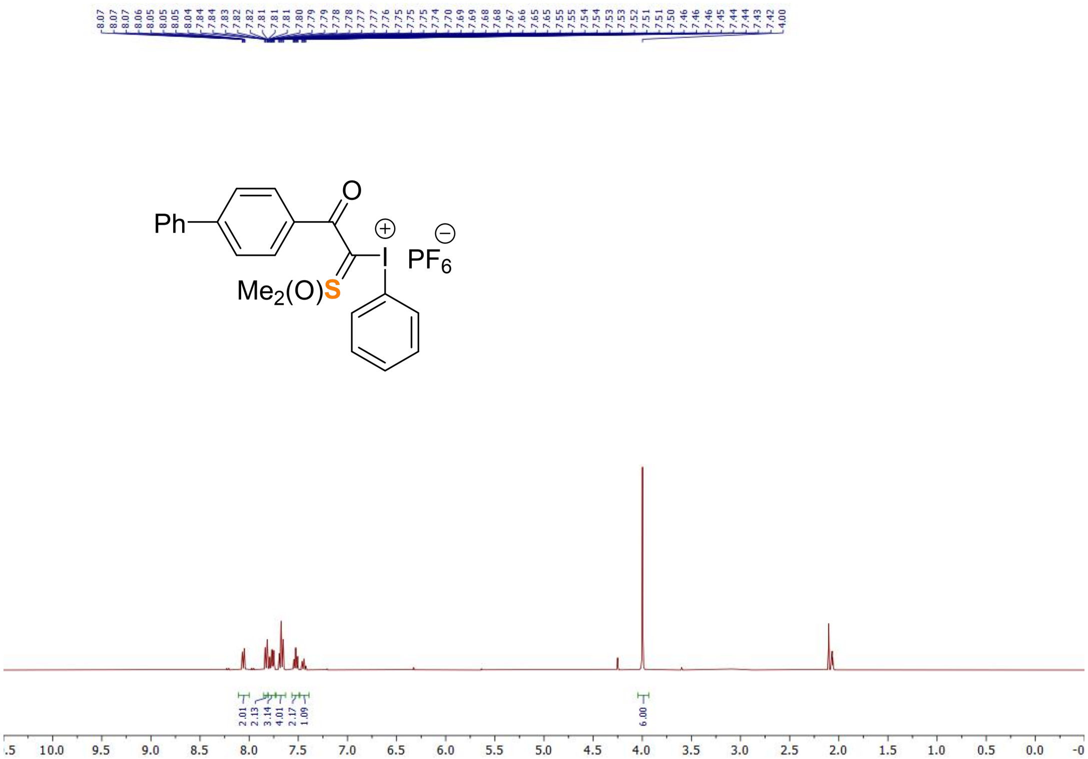

Supplementary Figure 56. ^{1}H NMR of the **3o** (400 MHz, 25 °C in Acetone-d_6)

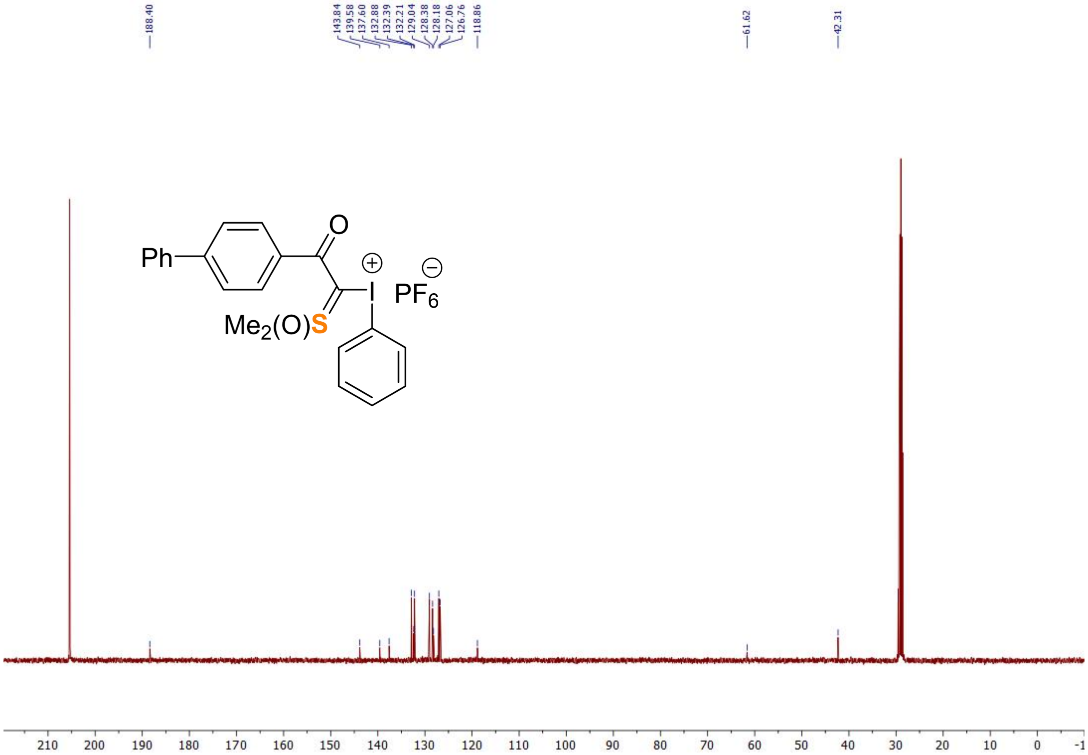

Supplementary Figure 57. ^{13}C NMR of the **3o** (101 MHz, 25 °C in Acetone-d_6)

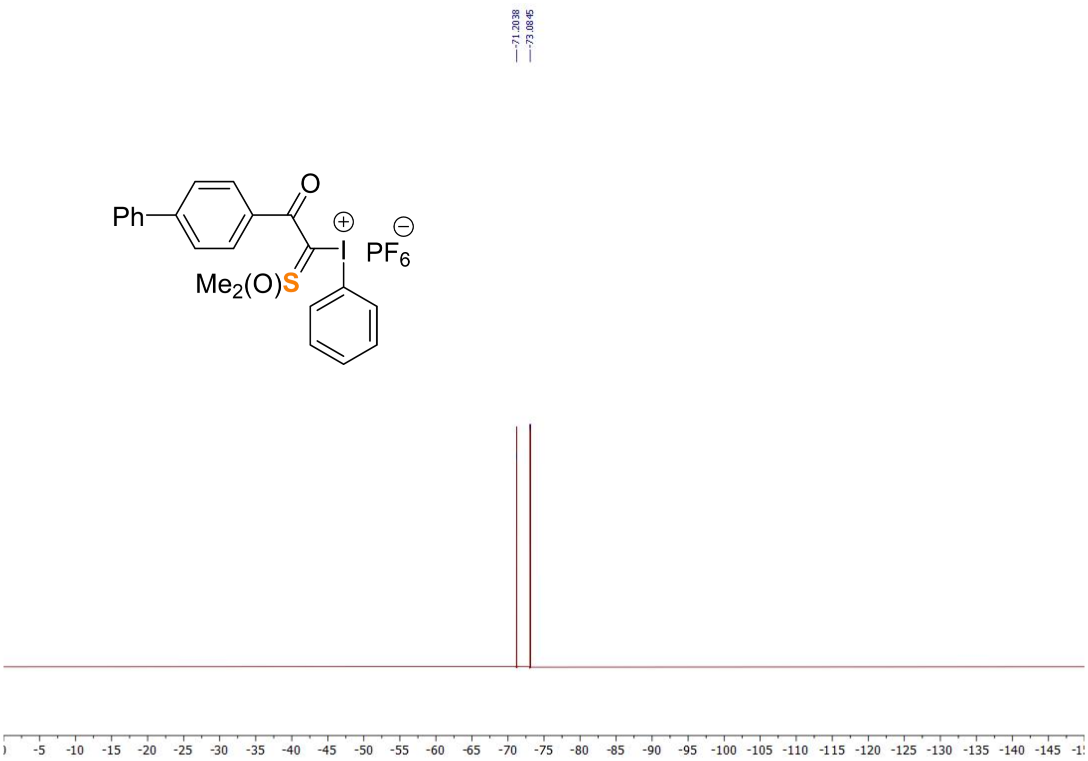

Supplementary Figure 58. ^{19}**F NMR** of the **3o** (376 MHz, 25 °C in Acetone-d_6)

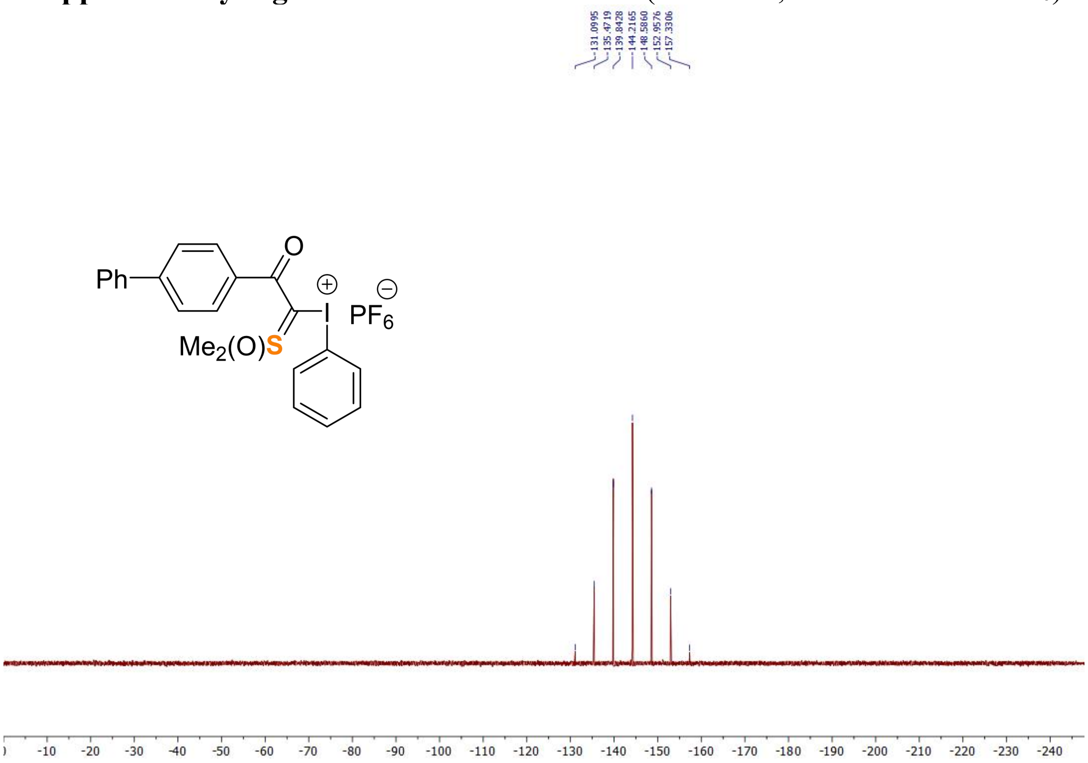

Supplementary Figure 59. ^{31}**P NMR** of the **3o** (162 MHz, 25 °C in Acetone-d_6)

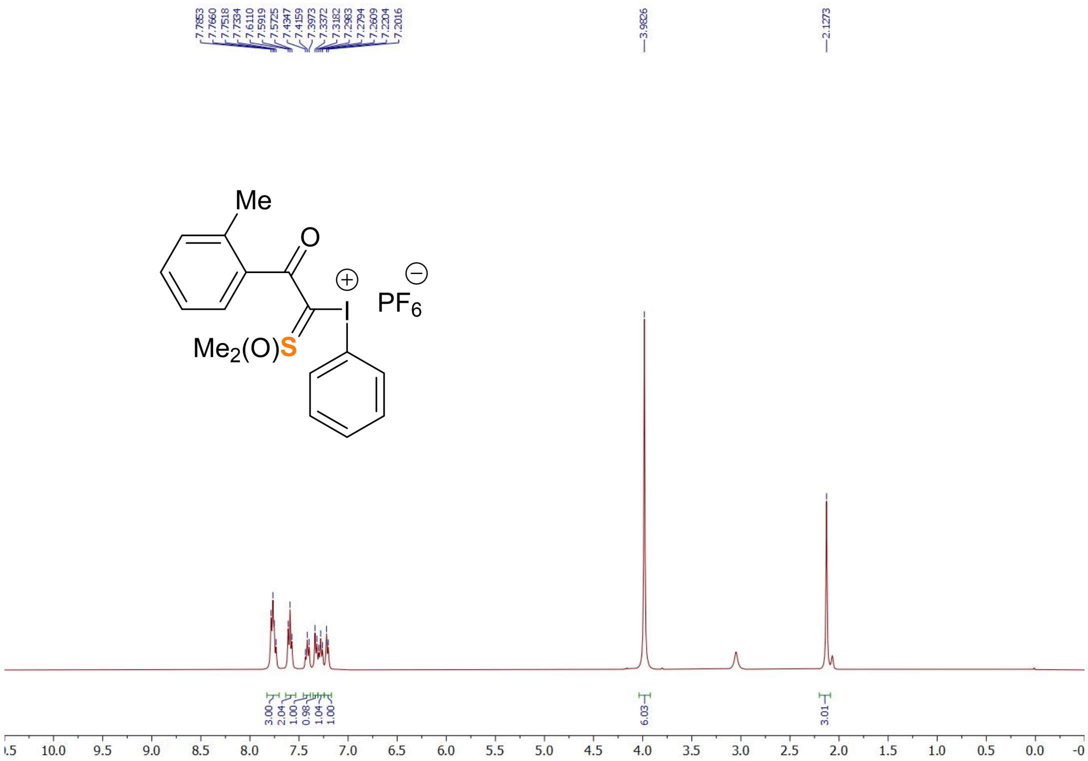

Supplementary Figure 60. ¹H NMR of the **3p** (400 MHz, 25 °C in Acetone-d_6)

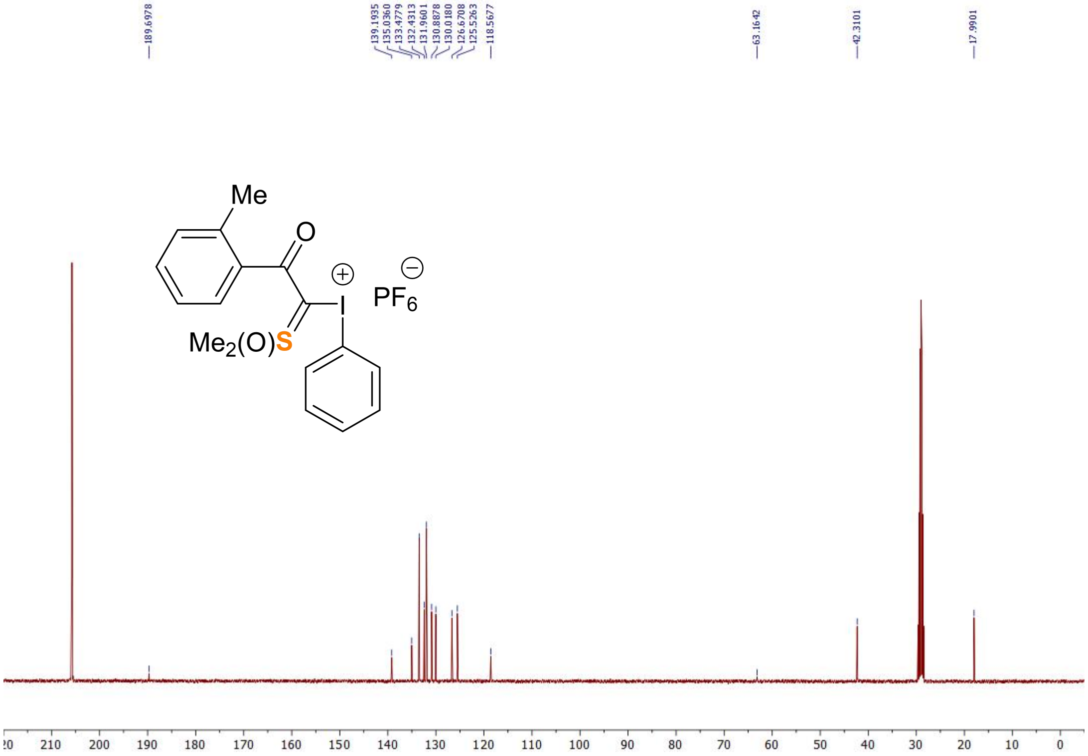

Supplementary Figure 61. ¹³C NMR of the **3p** (101 MHz, 25 °C in Acetone-d_6)

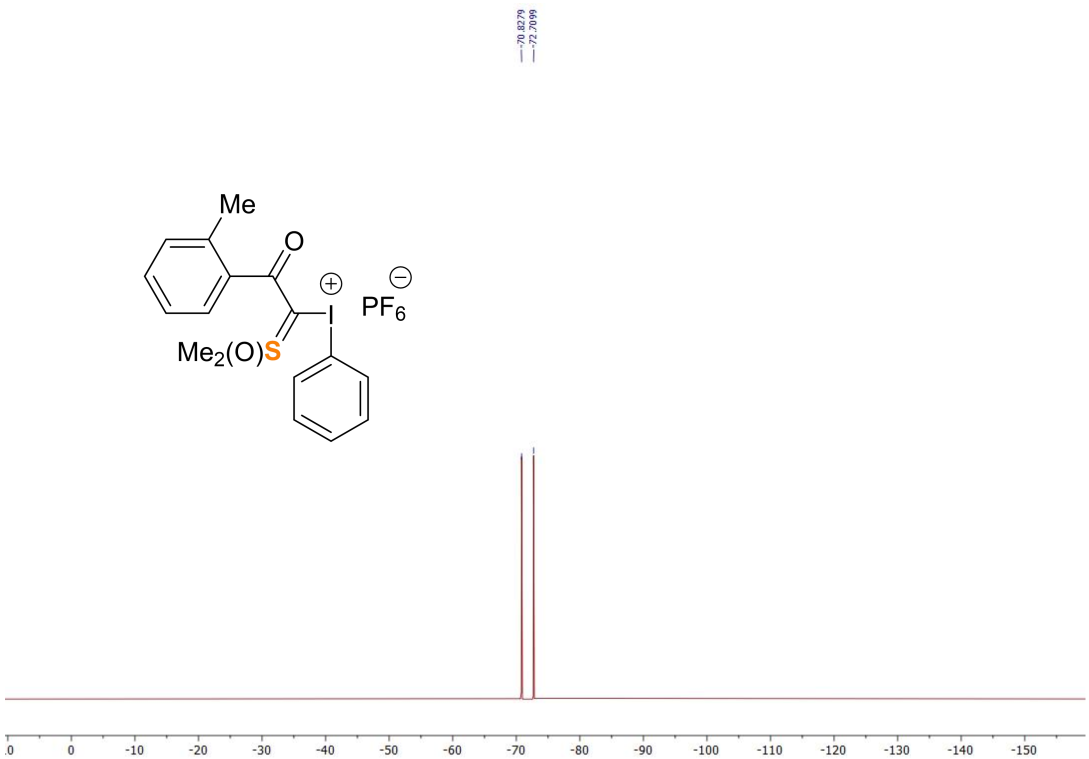

Supplementary Figure 62. ^{19}**F NMR** of the **3p** (376 MHz, 25 °C in Acetone-d_6)

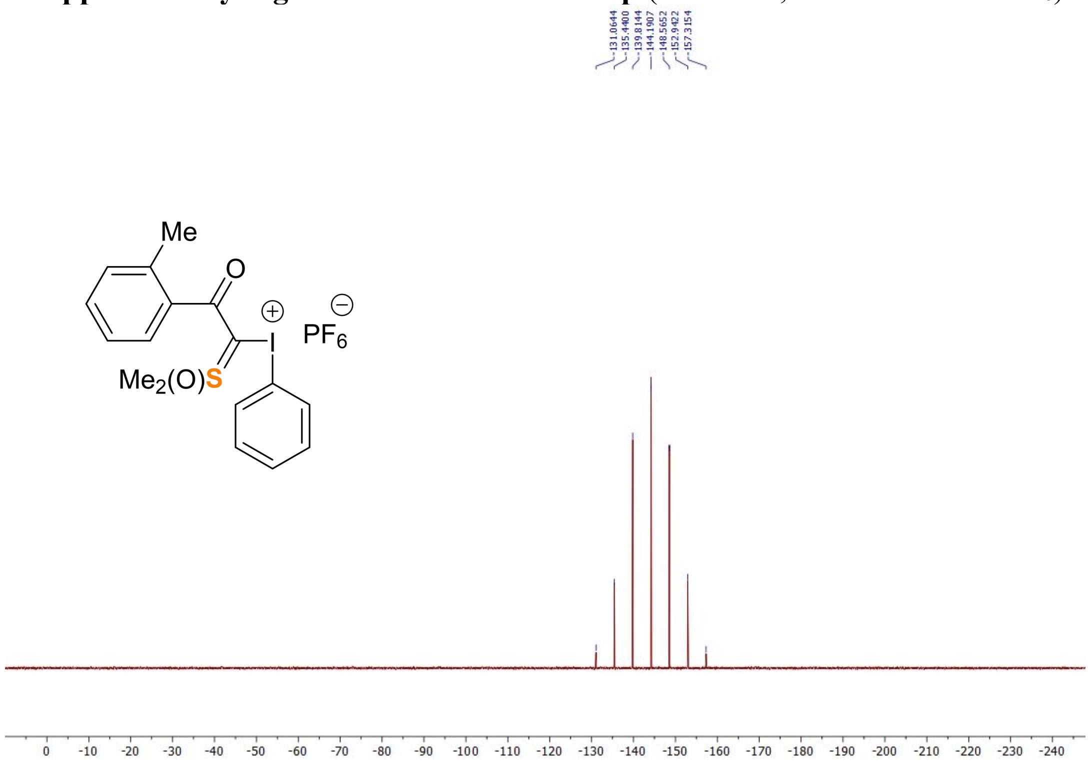

Supplementary Figure 63. ^{31}**P NMR** of the **3p** (162 MHz, 25 °C in Acetone-d_6)

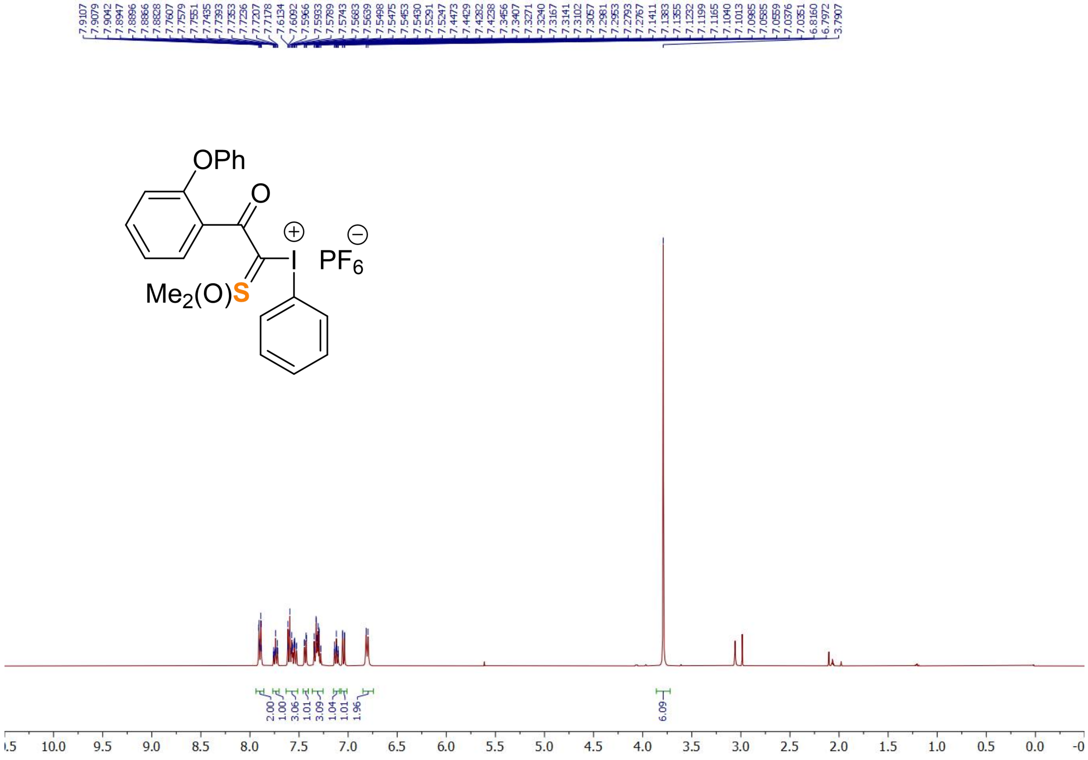

Supplementary Figure 64. ¹H NMR of the **3q** (400 MHz, 25 °C in Acetone-d_6)

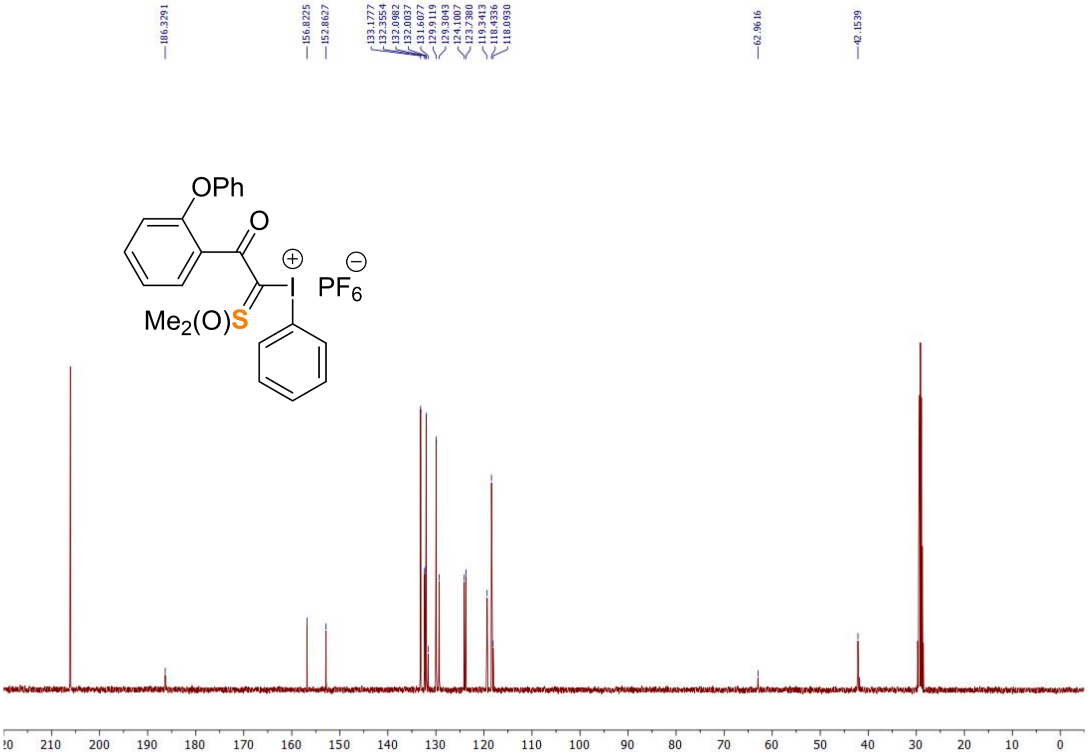

Supplementary Figure 65. ¹³C NMR of the **3q** (101 MHz, 25 °C in Acetone-d_6)

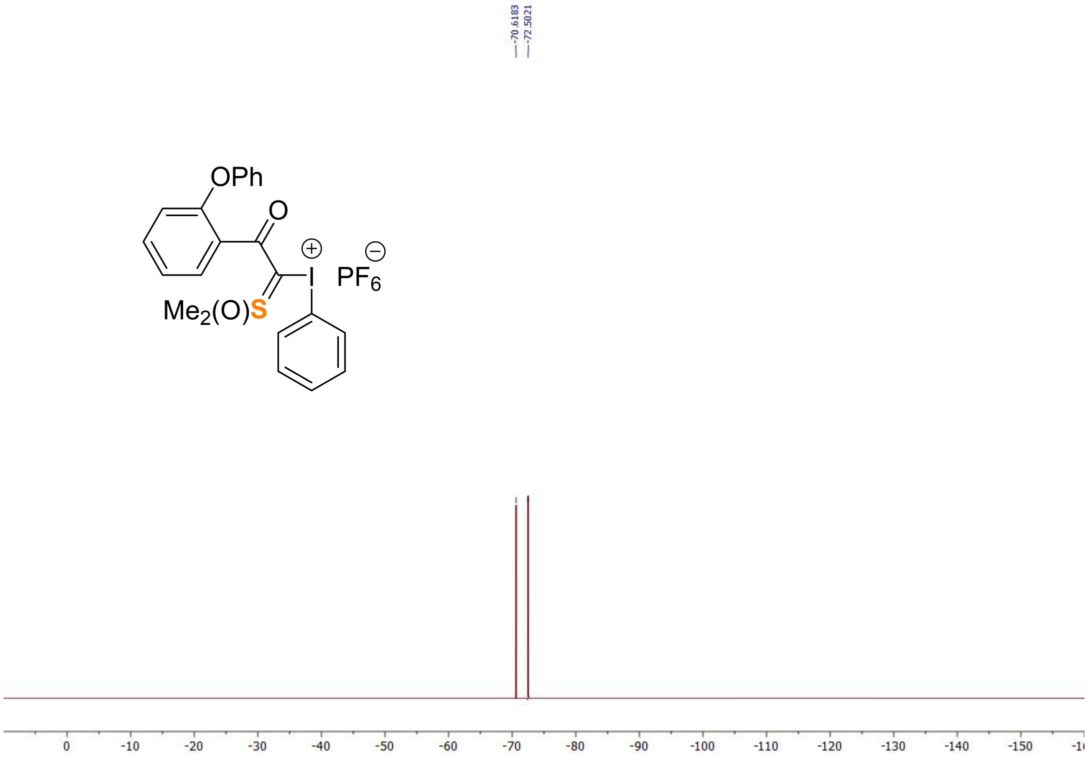

Supplementary Figure 66. ^{19}**F NMR** of the **3q** (376 MHz, 25 °C in Acetone-d_6)

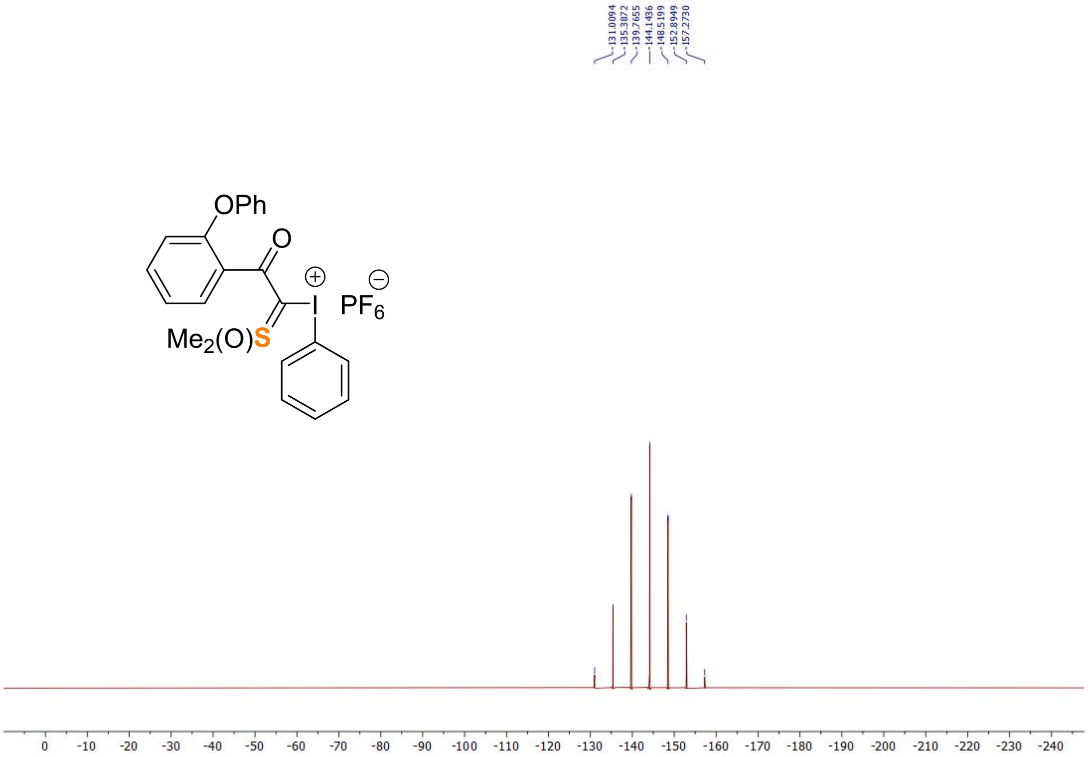

Supplementary Figure 67. ^{31}**P NMR** of the **3q** (162 MHz, 25 °C in Acetone-d_6)

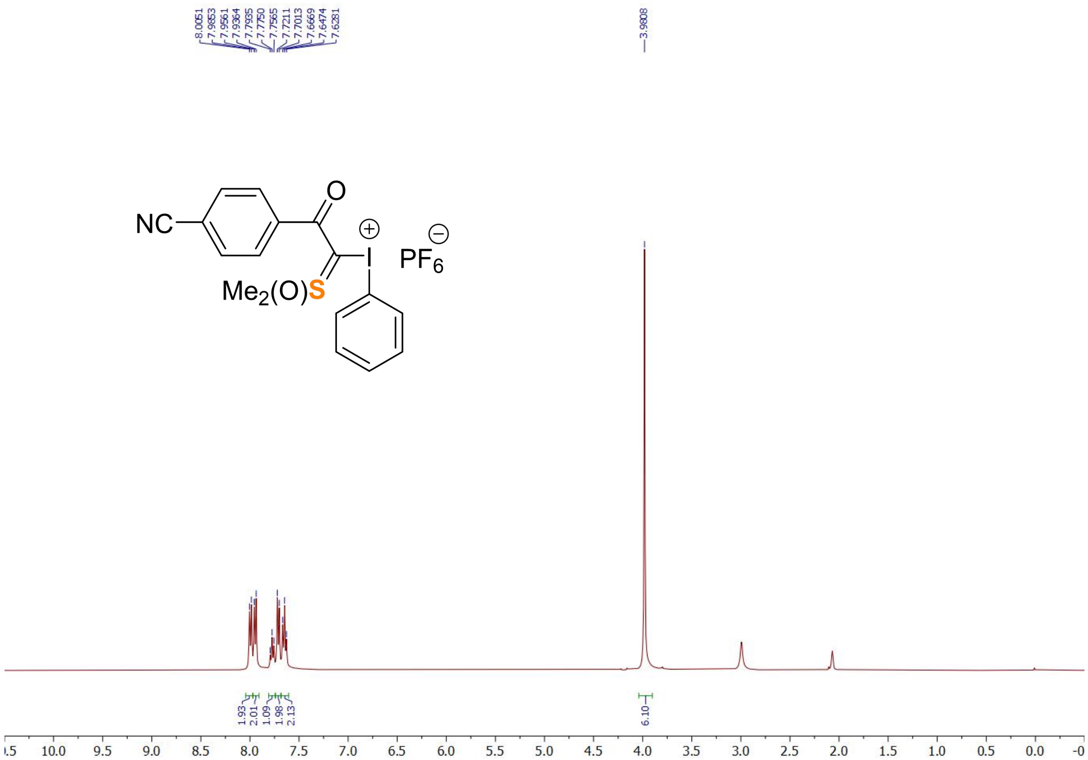

Supplementary Figure 68. ^{1}H NMR of the **3r** (400 MHz, 25 °C in Acetone-d_6)

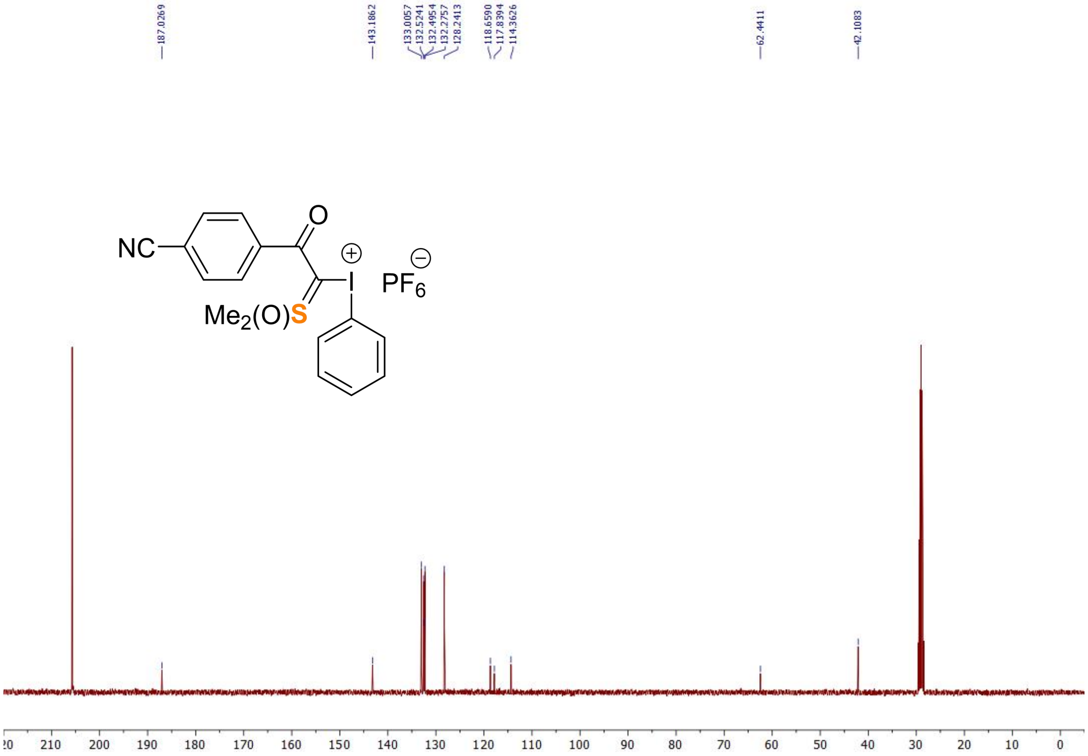

Supplementary Figure 69. ^{13}C NMR of the **3r** (101 MHz, 25 °C in Acetone-d_6)

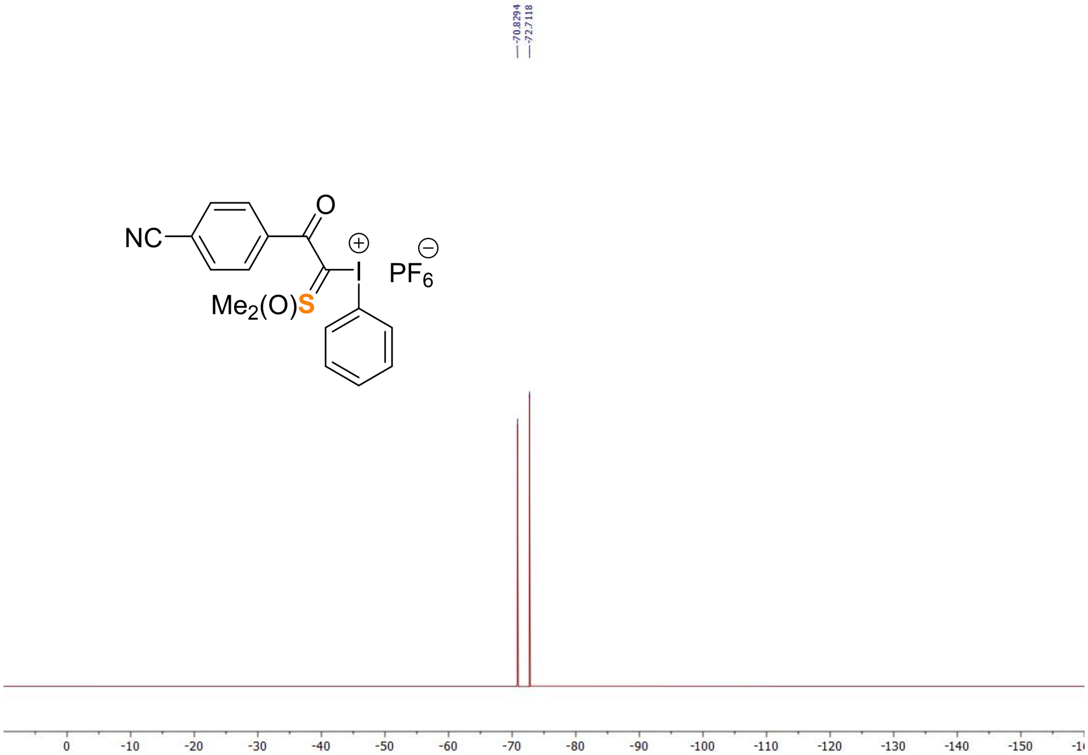

Supplementary Figure 70. ^{19}**F NMR** of the **3r** (376 MHz, 25 °C in Acetone-d_6)

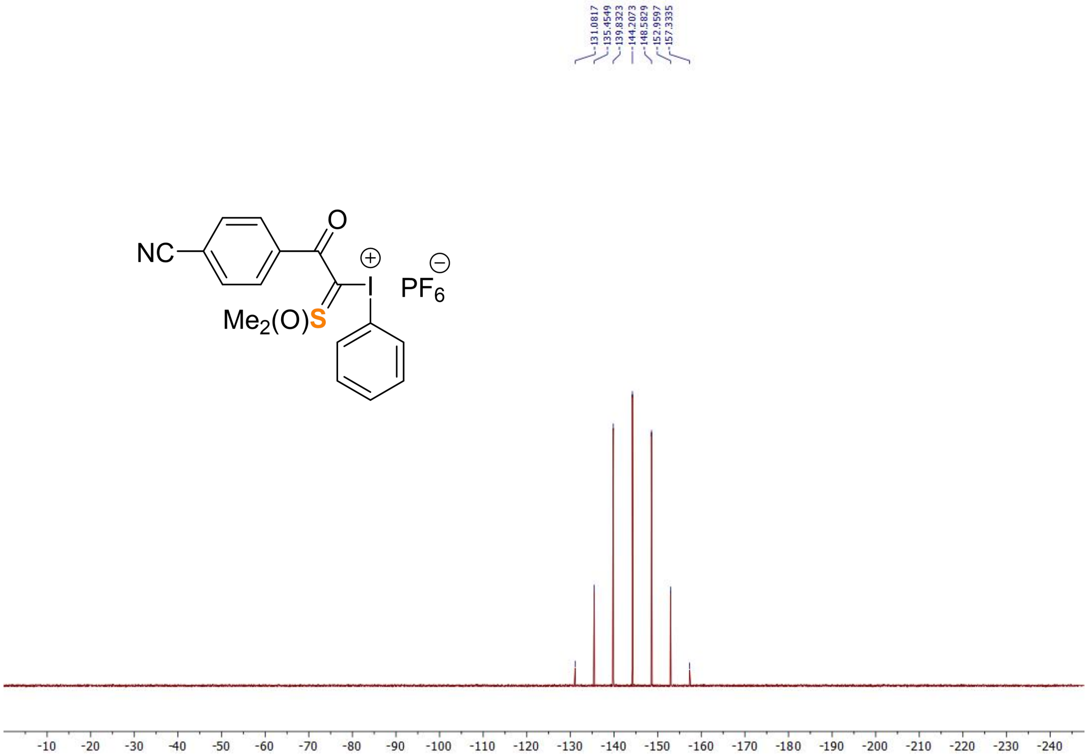

Supplementary Figure 71. ^{31}**P NMR** of the **3r** (162 MHz, 25 °C in Acetone-d_6)

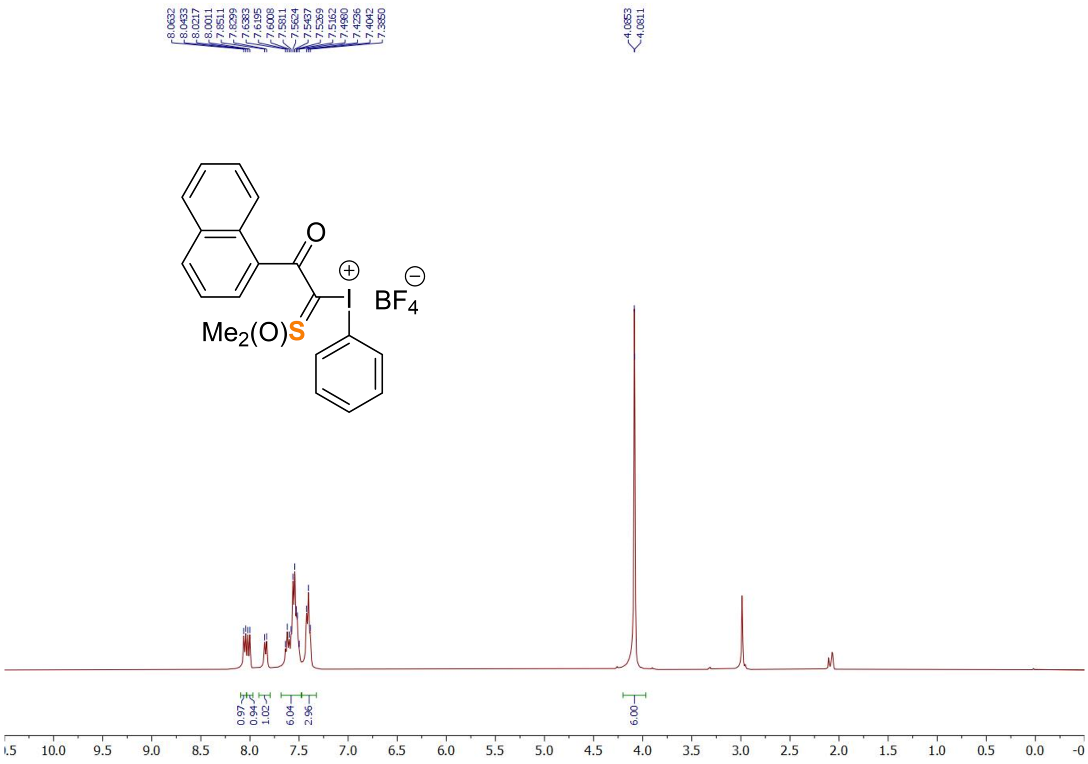

Supplementary Figure 72. ^{1}H NMR of the **3s** (400 MHz, 25 °C in Acetone-*d*$_6$)

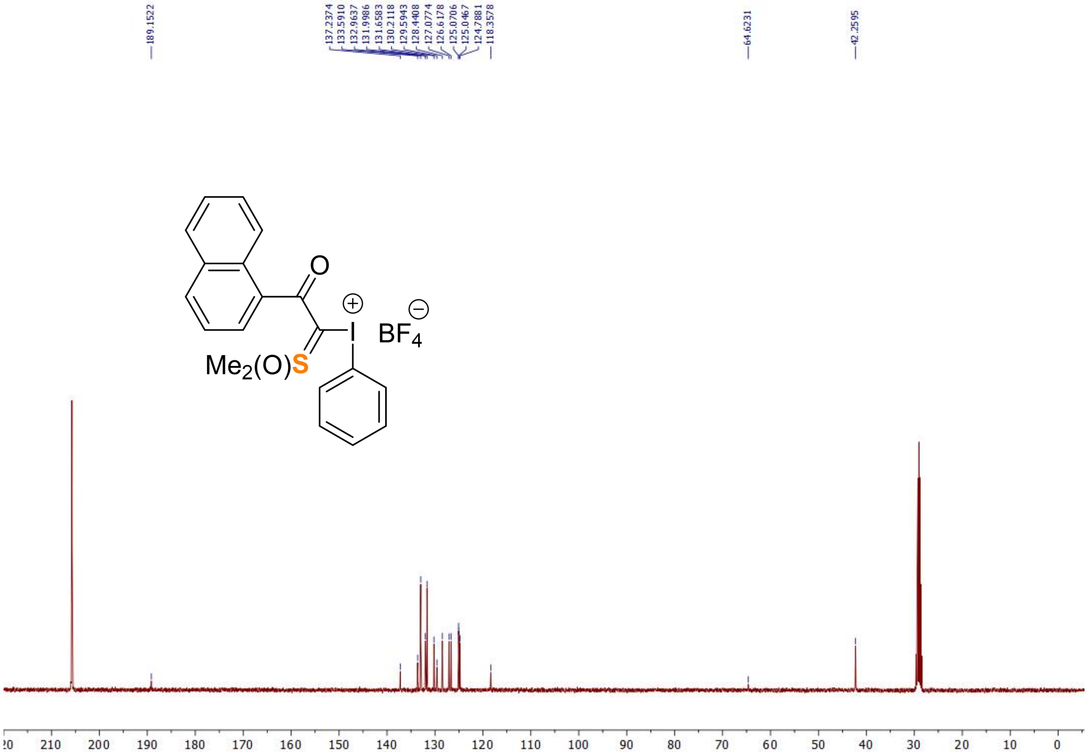

Supplementary Figure 73. ^{13}C NMR of the **3s** (101 MHz, 25 °C in Acetone-*d*$_6$)

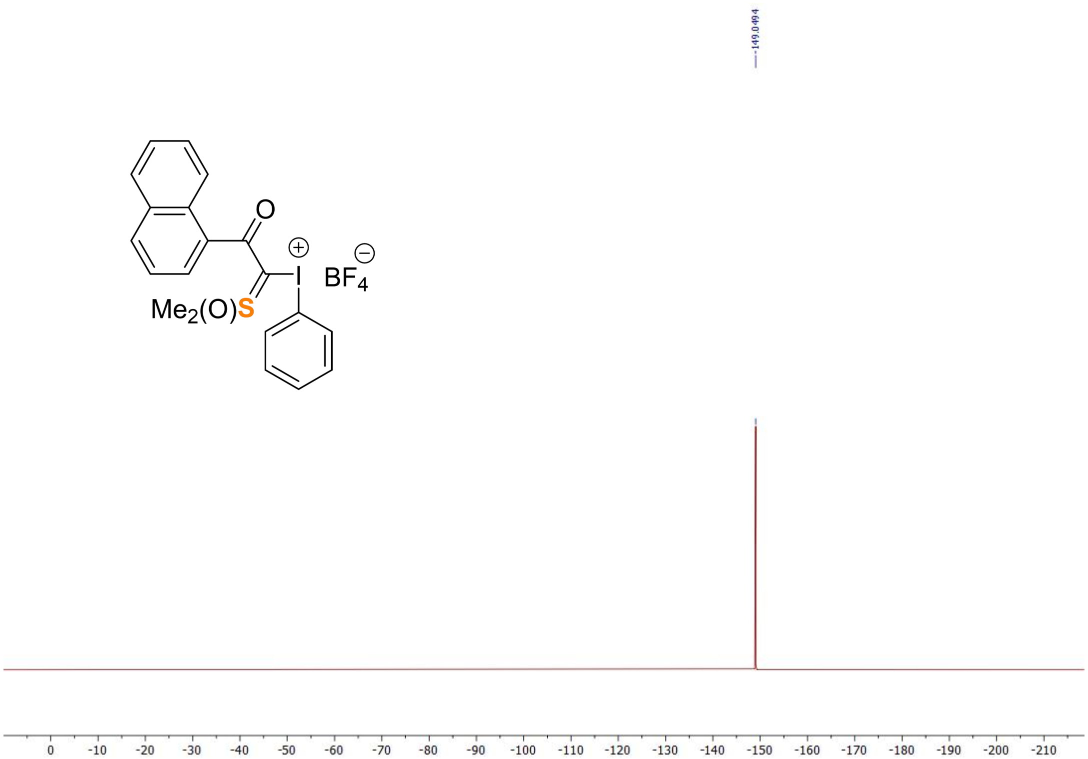

Supplementary Figure 74. ^{19}F NMR of the **3s** (376 MHz, 25 °C in Acetone-d_6)

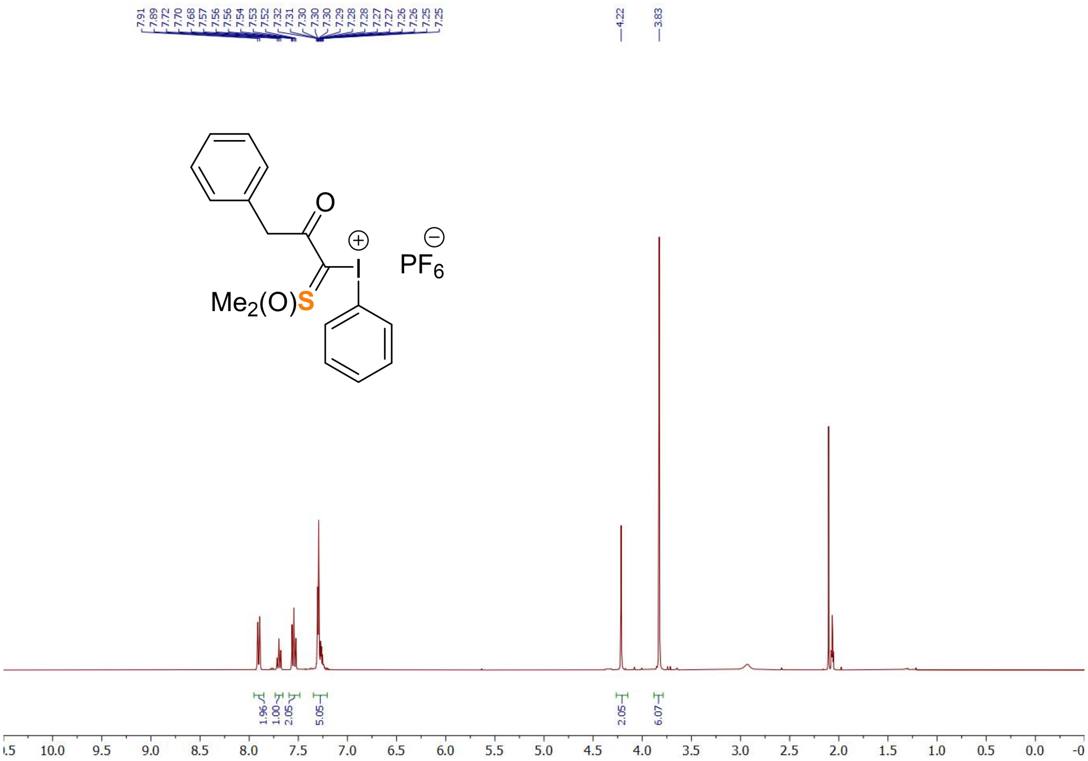

Supplementary Figure 75. ^{1}H NMR of the **3t** (400 MHz, 25 °C in Acetone-d_6)

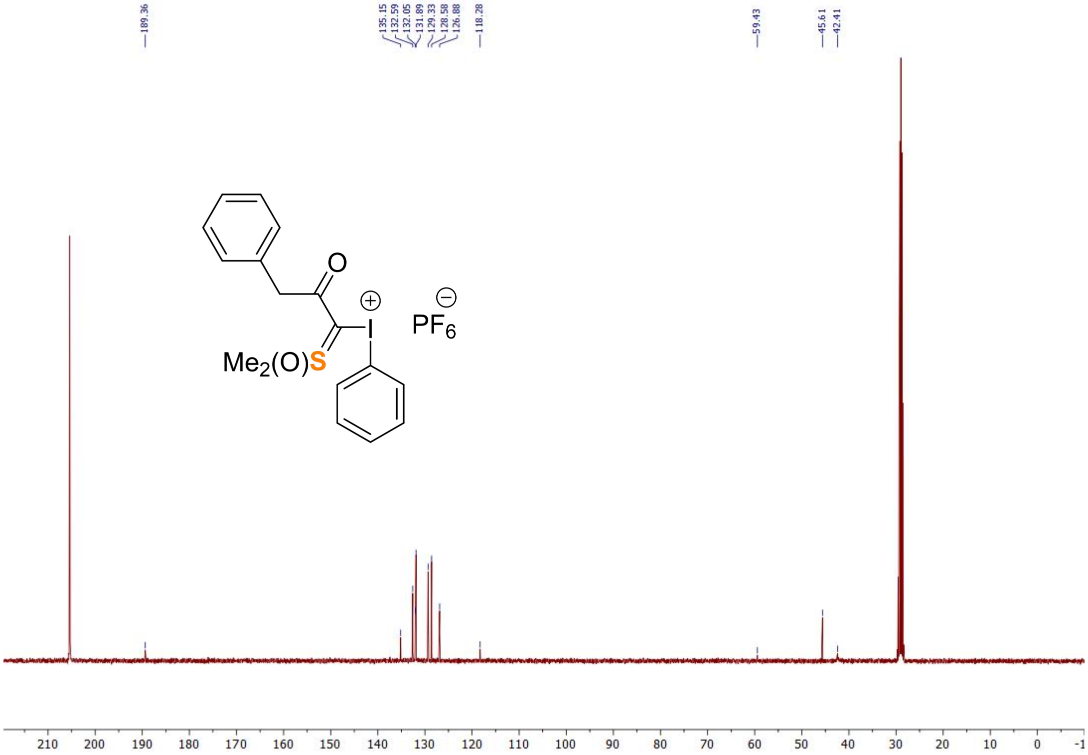

Supplementary Figure 76. ^{13}C NMR of the **3t** (101 MHz, 25 °C in Acetone-d_6)

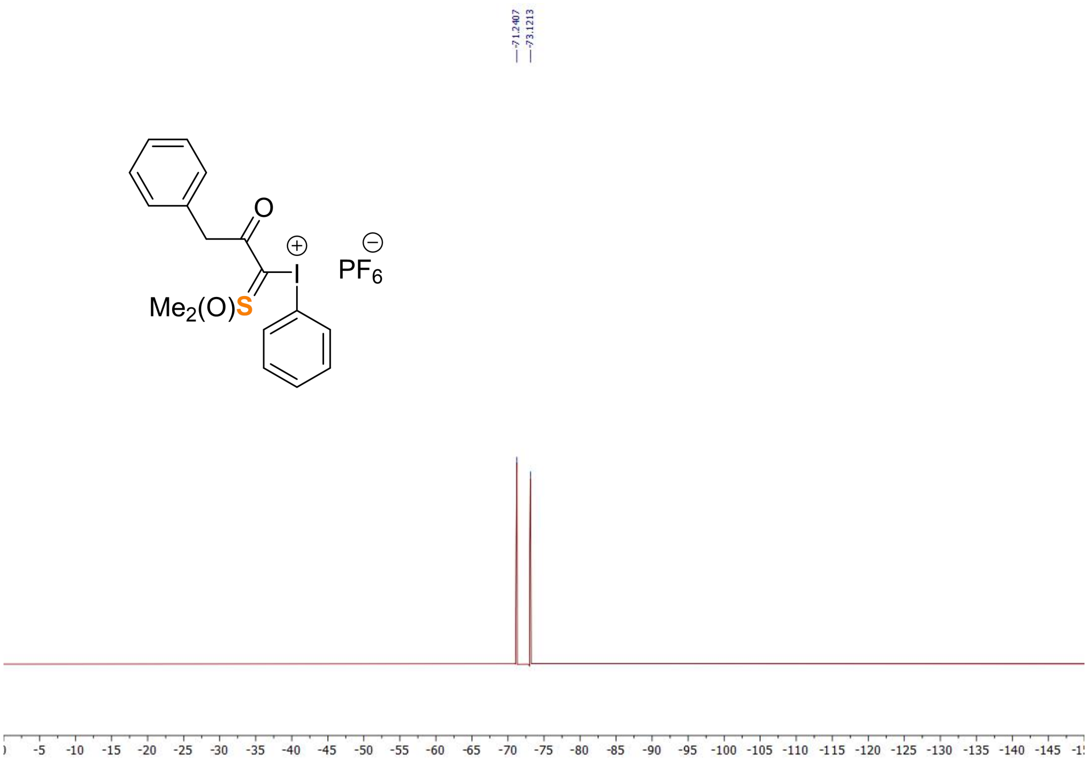

Supplementary Figure 77. ^{19}**F NMR** of the **3t** (376 MHz, 25 °C in Acetone-d_6)

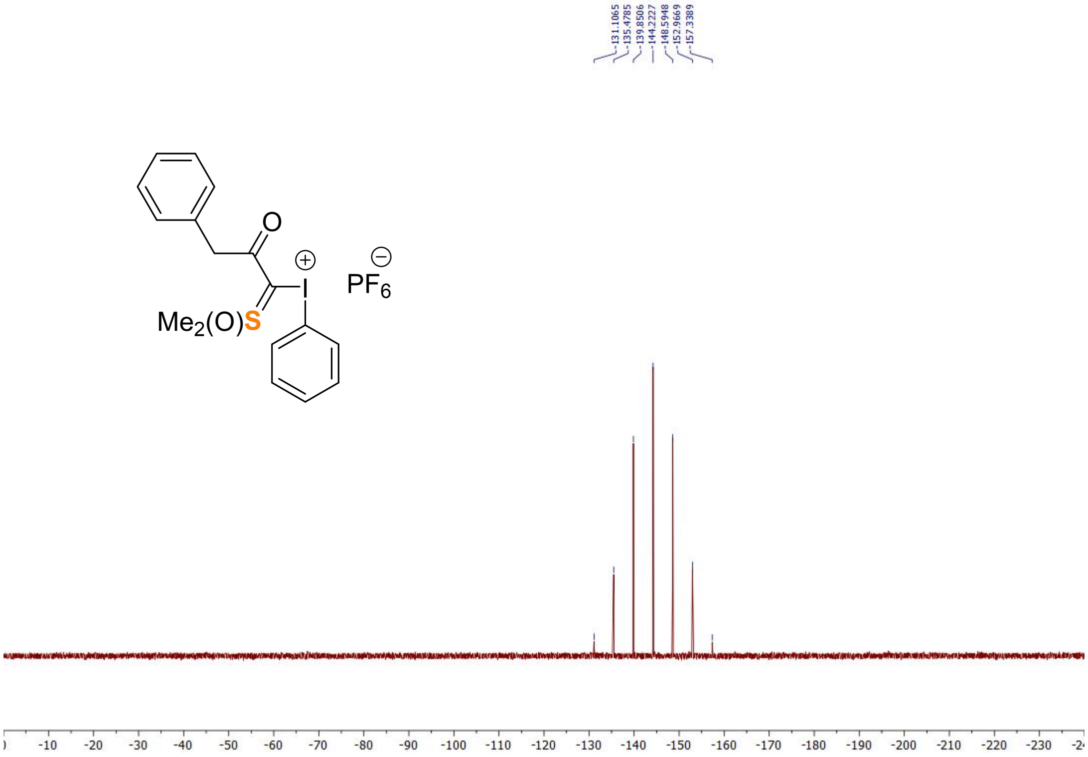

Supplementary Figure 78. ^{31}**P NMR** of the **3t** (162 MHz, 25 °C in Acetone-d_6)

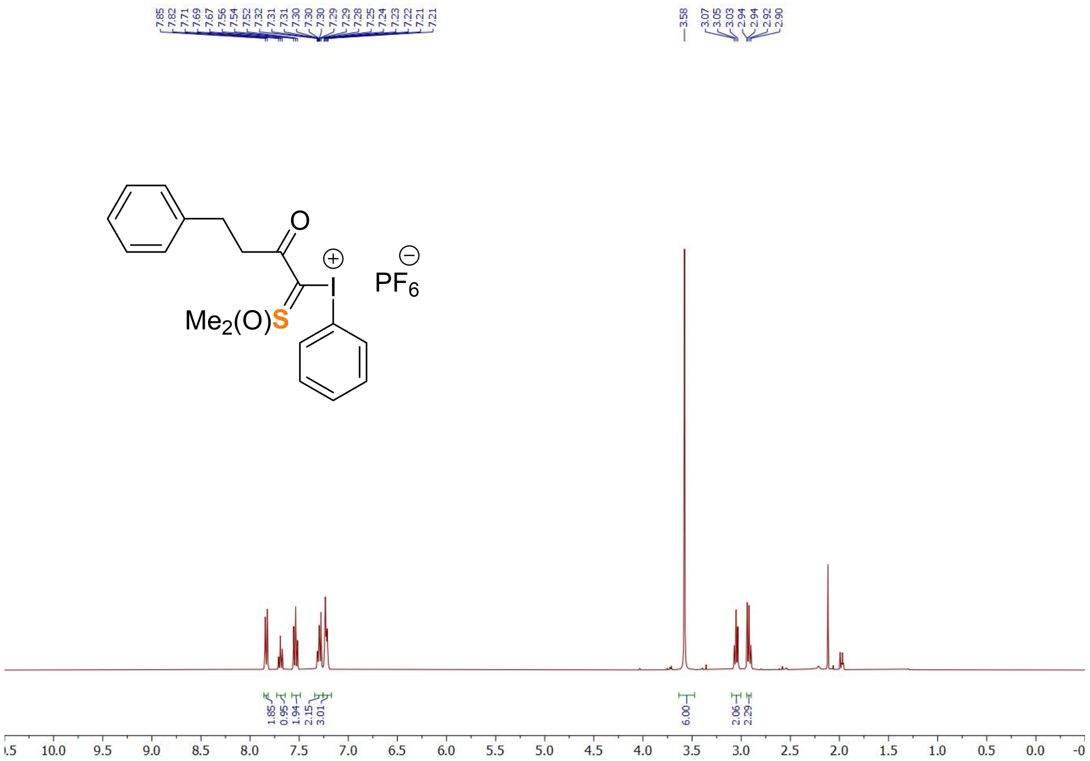

Supplementary Figure 79. ^{1}**H NMR** of the **3u** (400 MHz, 25 °C in CD_3CN)

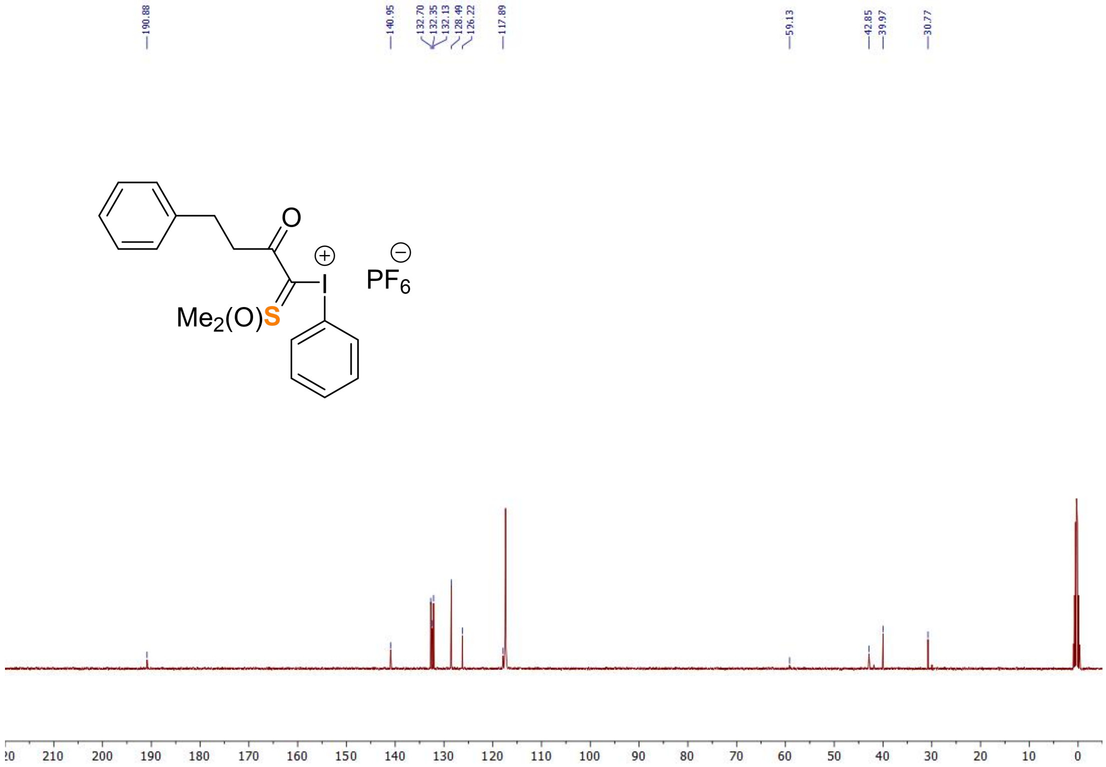

Supplementary Figure 80. ^{13}**C NMR** of the **3u** (101 MHz, 25 °C in CD_3CN)

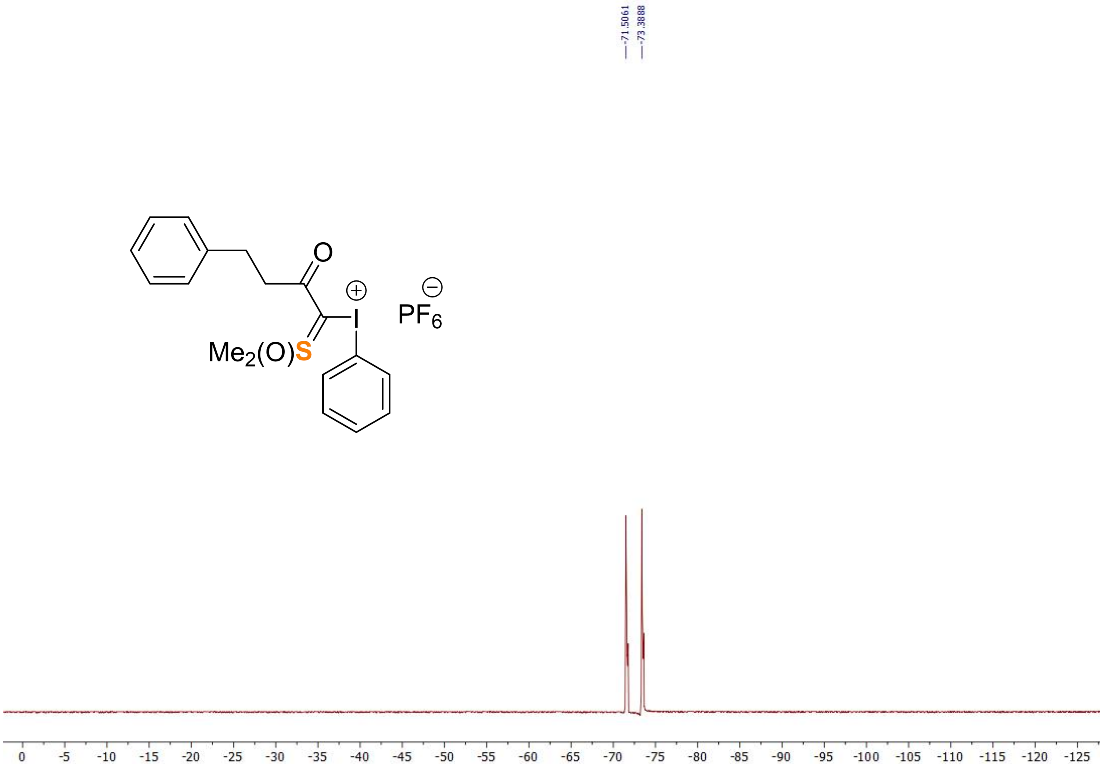

Supplementary Figure 81. ^{19}F NMR of the **3u** (376 MHz, 25 °C in CD_3CN)

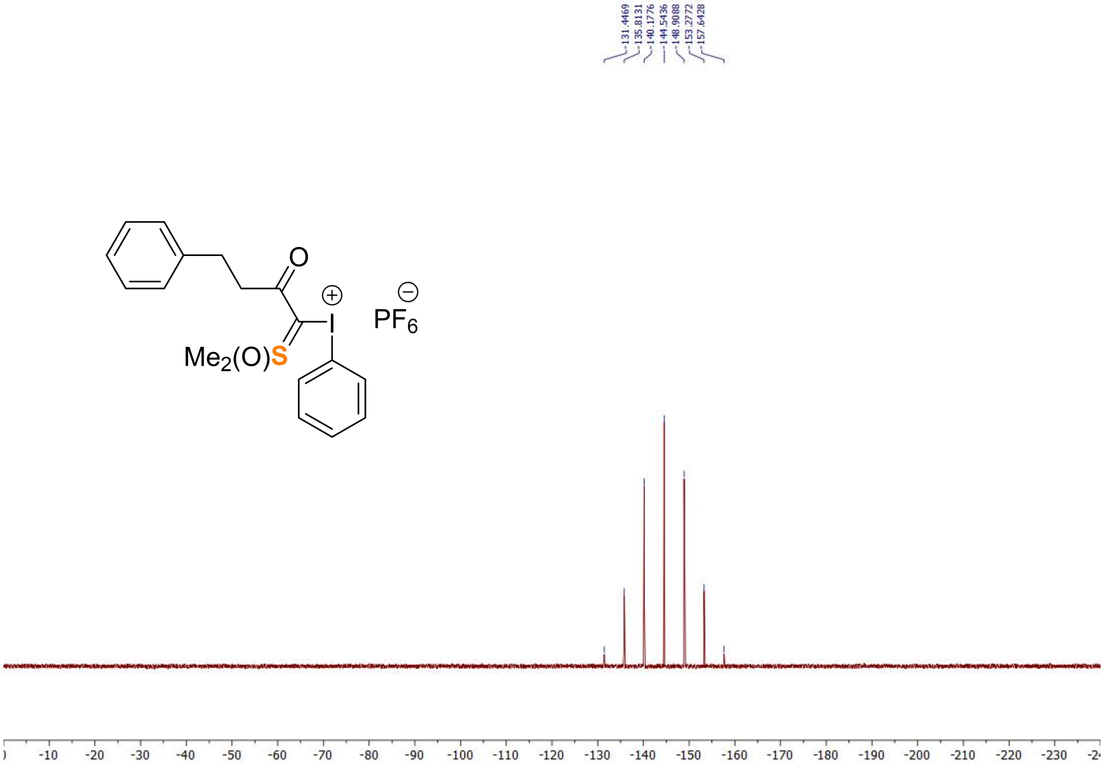

Supplementary Figure 82. ^{31}P NMR of the **3u** (162 MHz, 25 °C in CD_3CN)

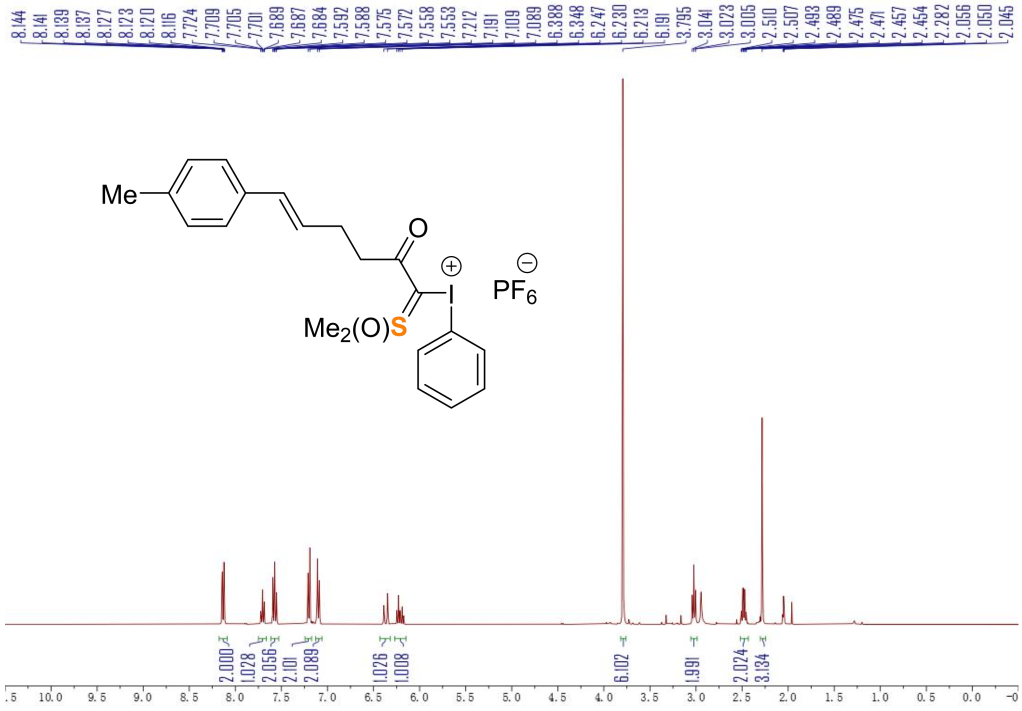

Supplementary Figure 83. ^{1}H NMR of the **3v** (400 MHz, 25 °C in Acetone-d_6)

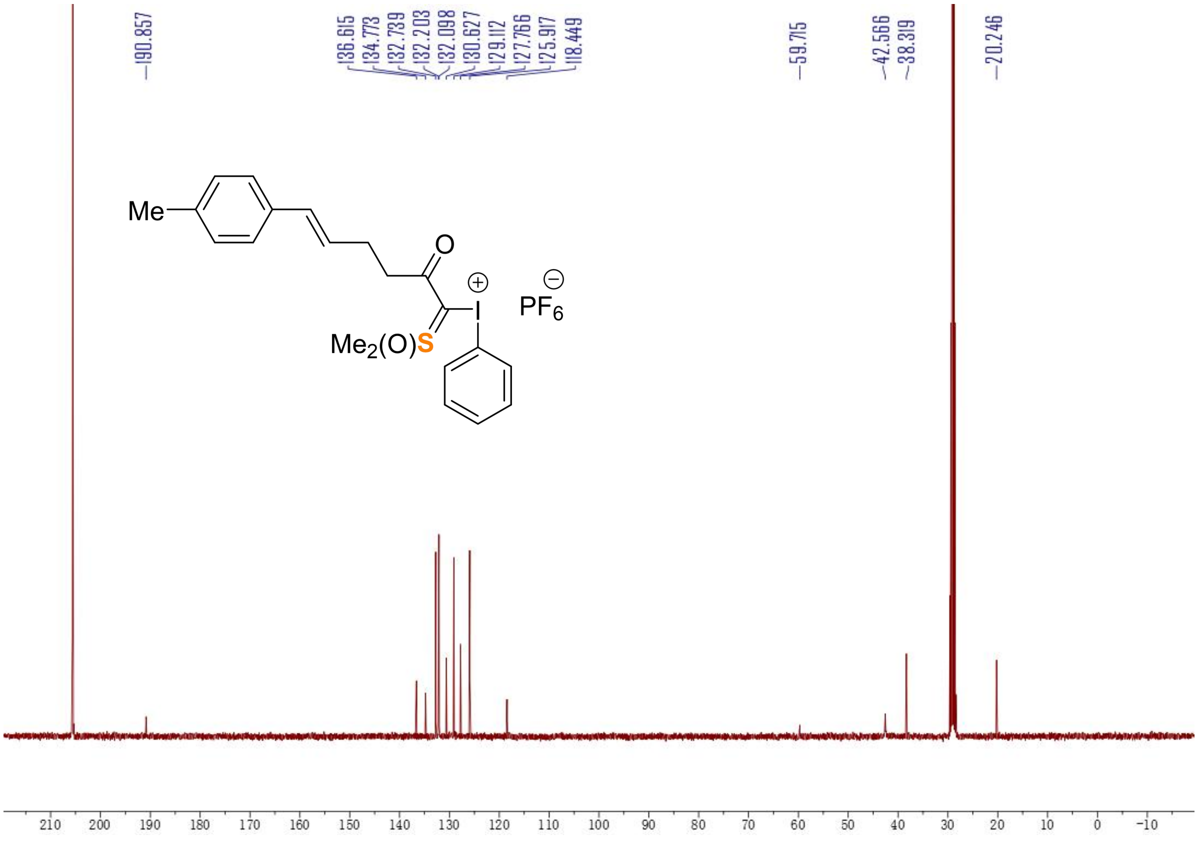

Supplementary Figure 84. ^{13}C NMR of the **3v** (101 MHz, 25 °C in Acetone-d_6)

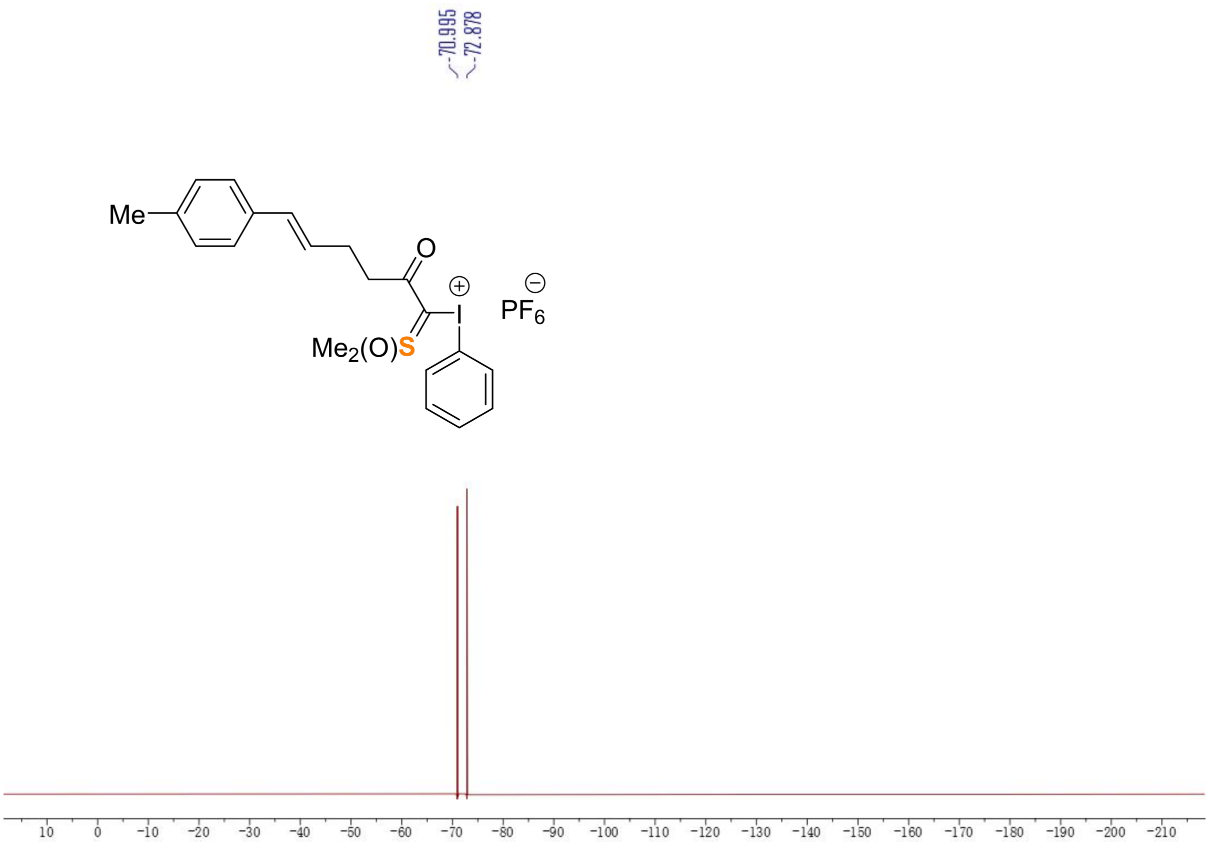

Supplementary Figure 85. ^{19}**F NMR** of the **3v** (376 MHz, 25 °C in Acetone-d_6)

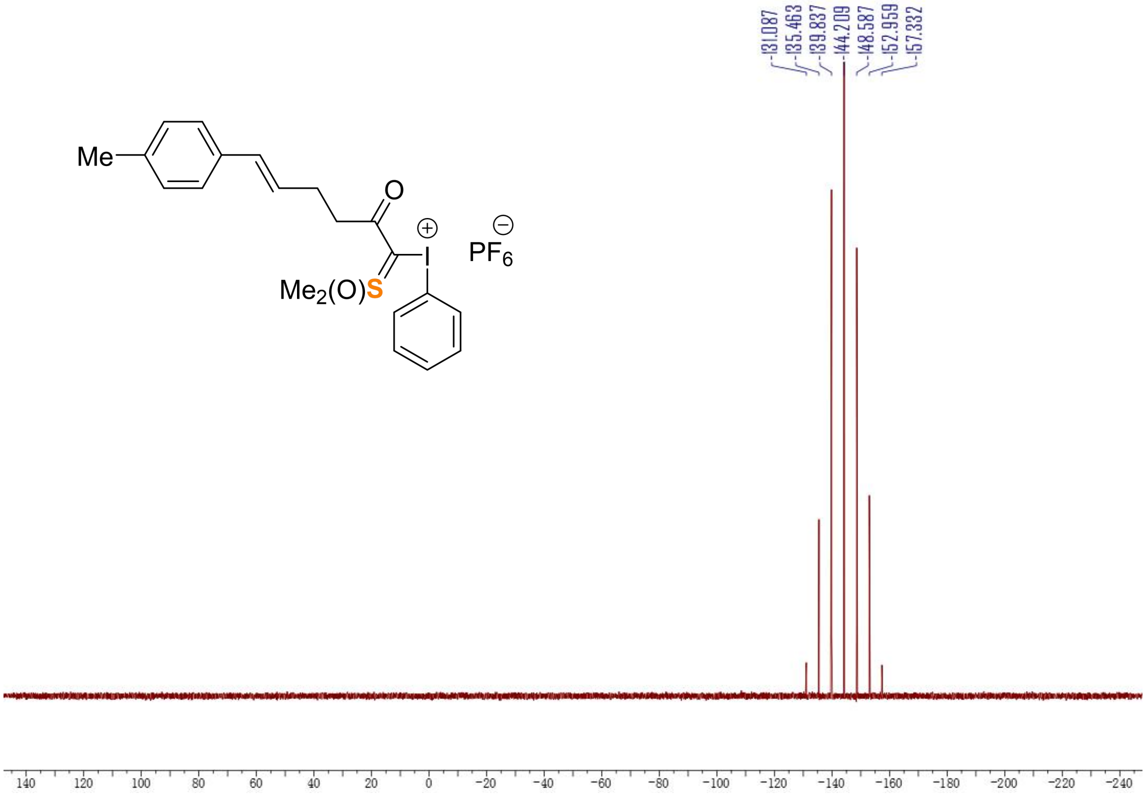

Supplementary Figure 86. ^{31}**P NMR** of the **3v** (162 MHz, 25 °C in Acetone-d_6)

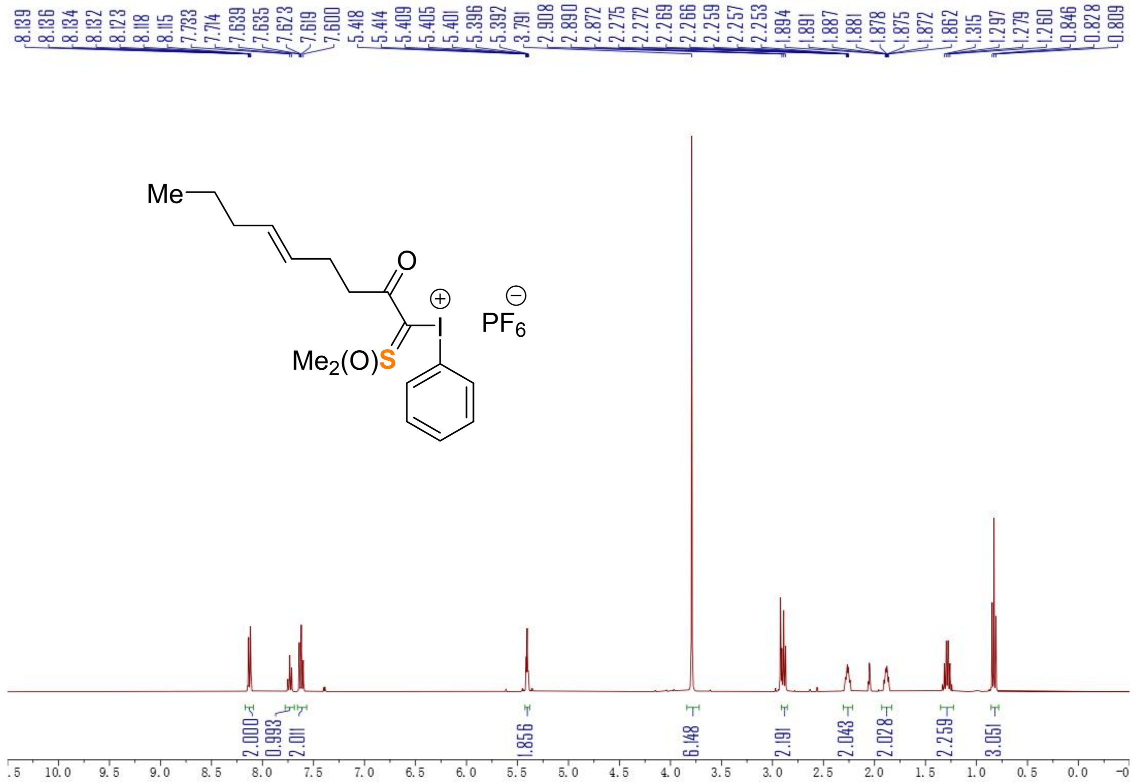

Supplementary Figure 87. ^{1}H NMR of the **3w** (400 MHz, 25 °C in Acetone-d_6)

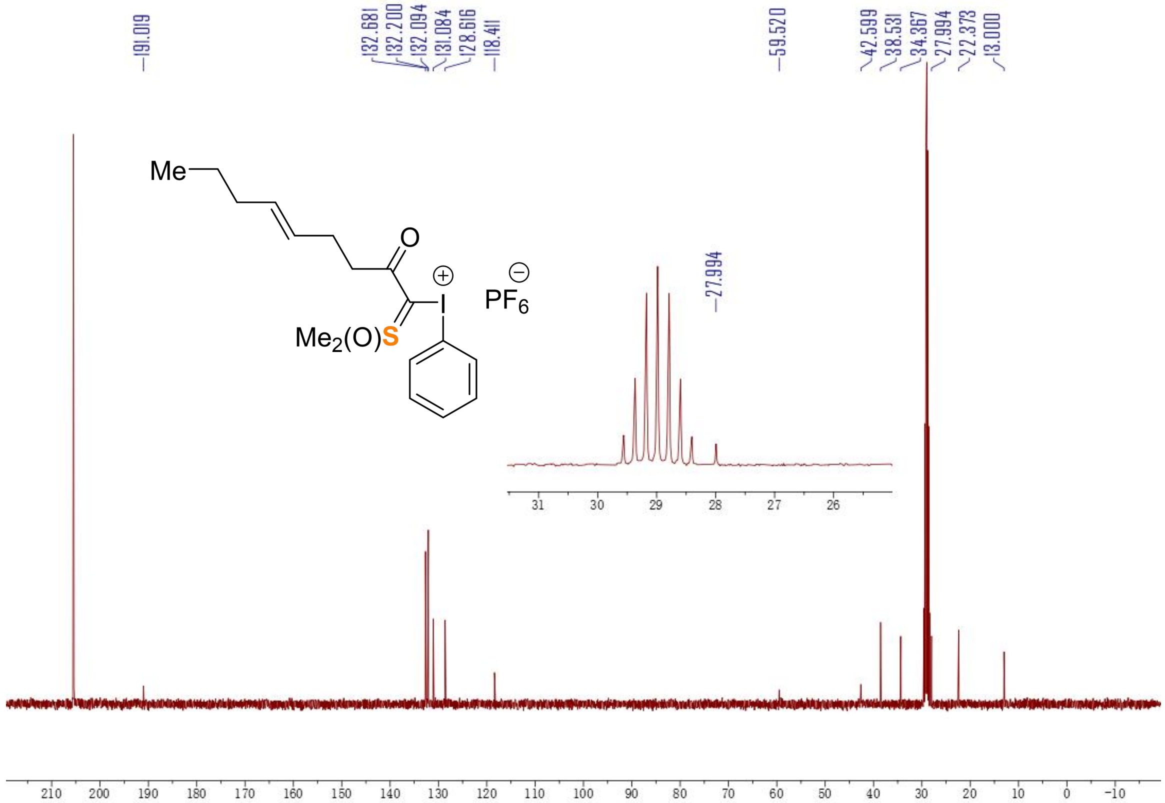

Supplementary Figure 88. ^{13}C NMR of the **3w**(101 MHz, 25 °C in Acetone-d_6)

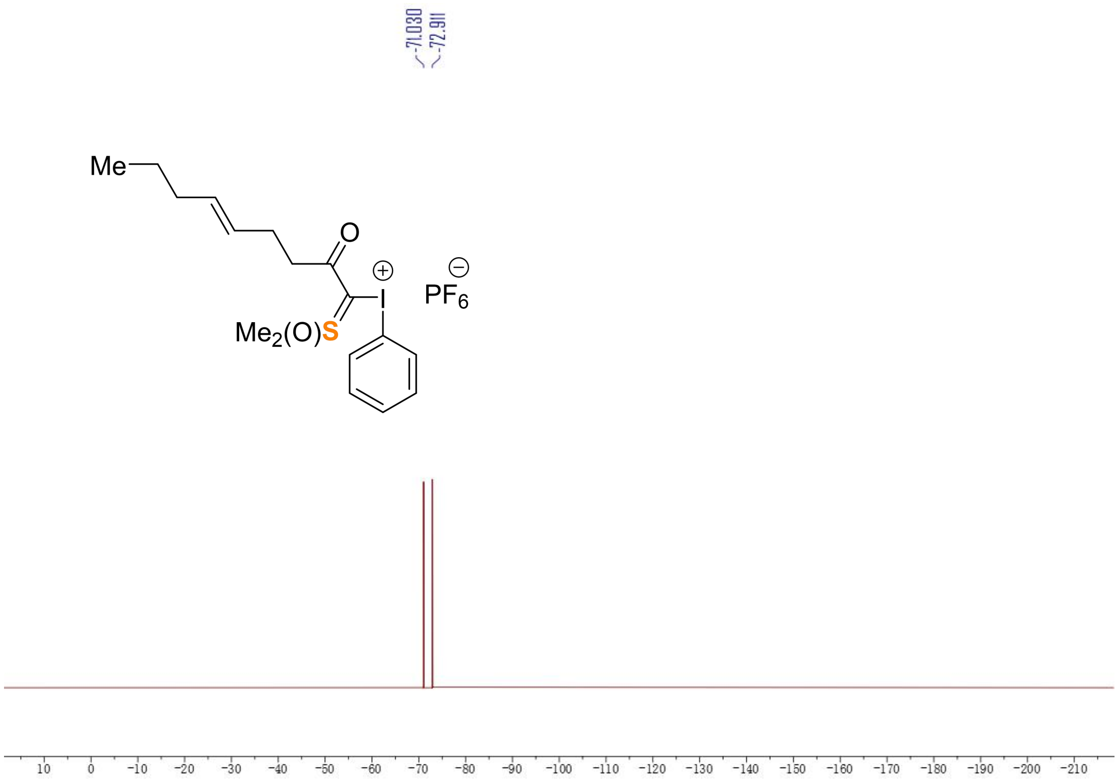

Supplementary Figure 89. **^{19}F NMR** of the **3w** (376 MHz, 25 °C in Acetone-d_6)

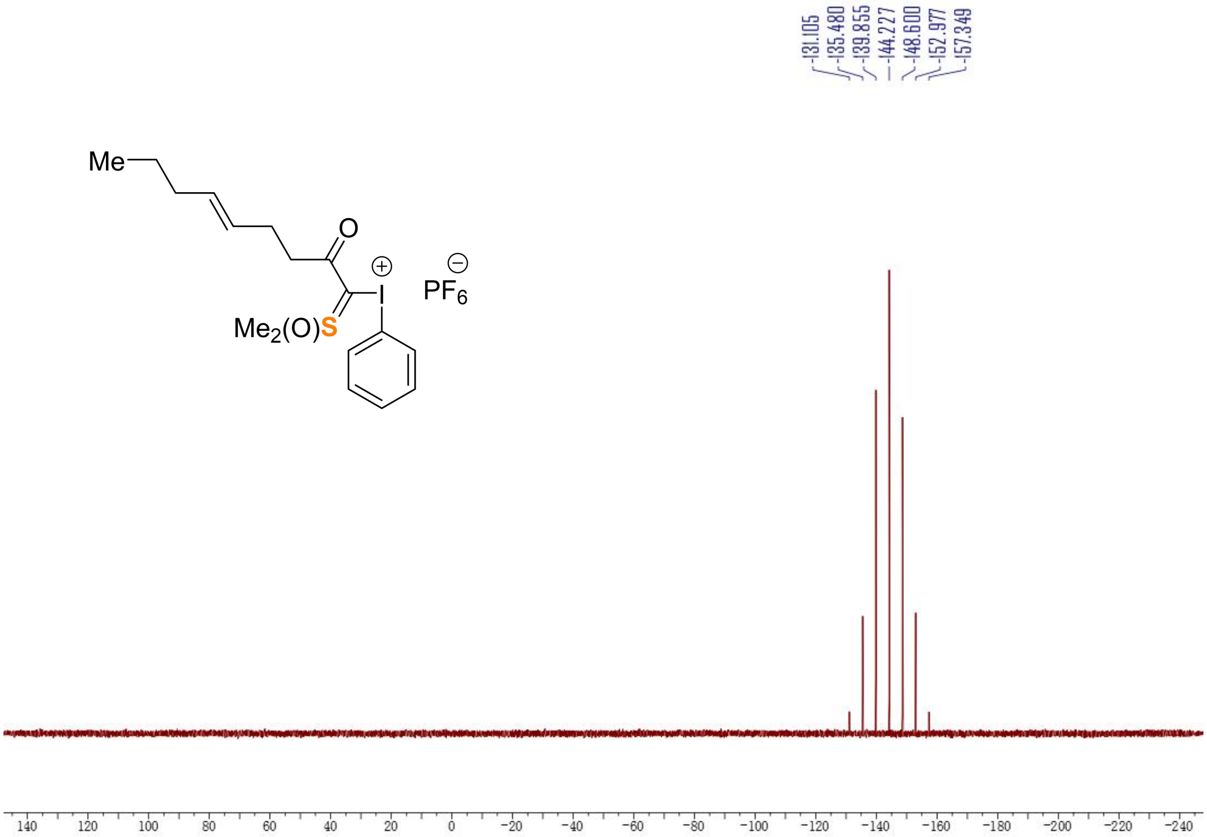

Supplementary Figure 90. **^{31}P NMR** of the **3w** (162 MHz, 25 °C in Acetone-d_6)

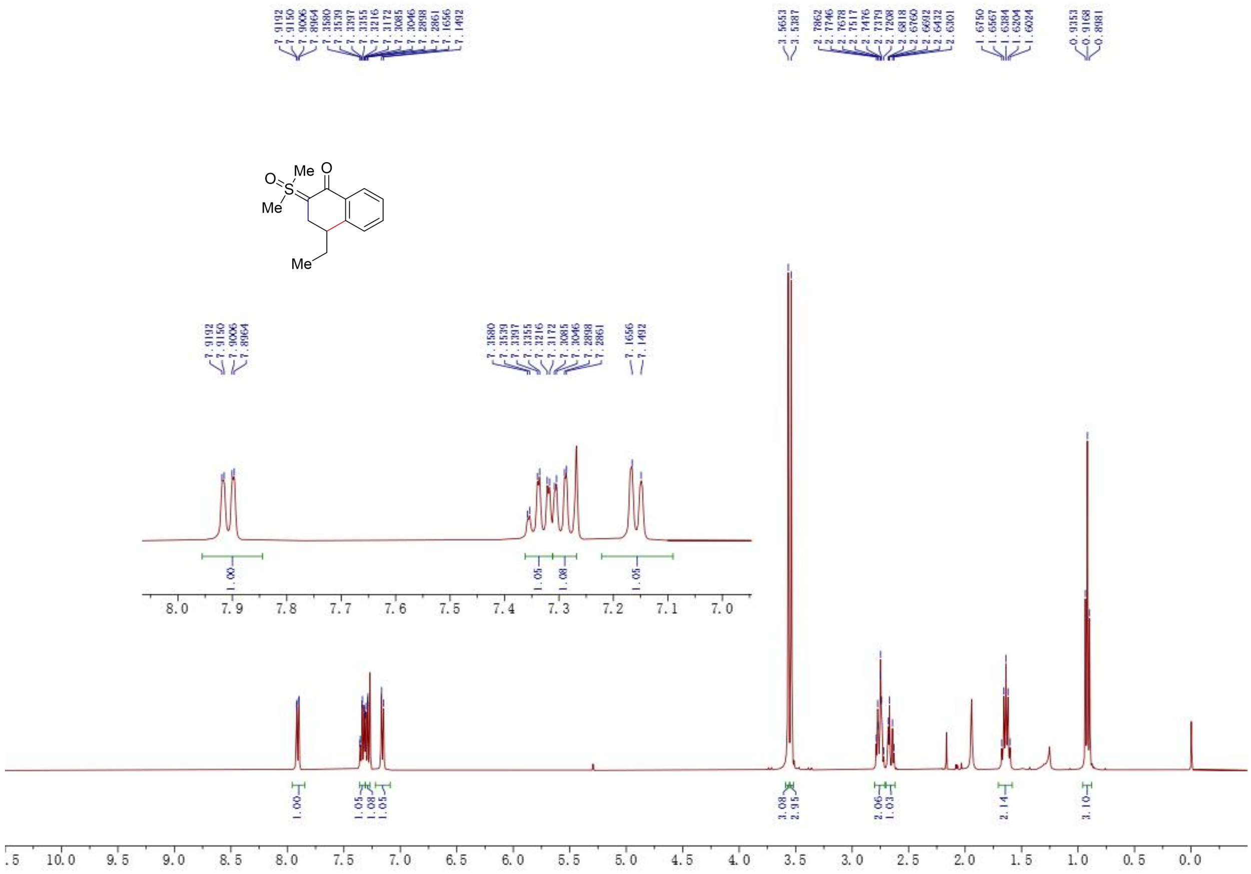

Supplementary Figure 91. ^{1}H NMR of the **5a** (400 MHz, 25 °C in $CDCl_3$)

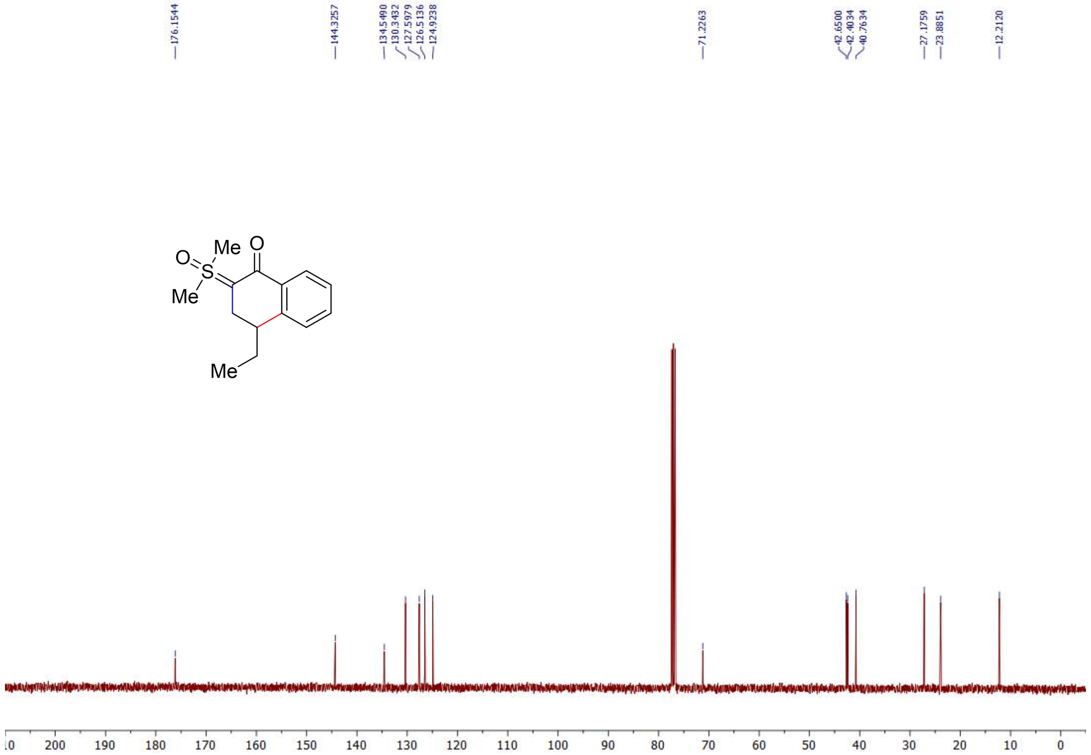

Supplementary Figure 92. ^{13}C NMR of the **5a** (101 MHz, 25 °C in $CDCl_3$)

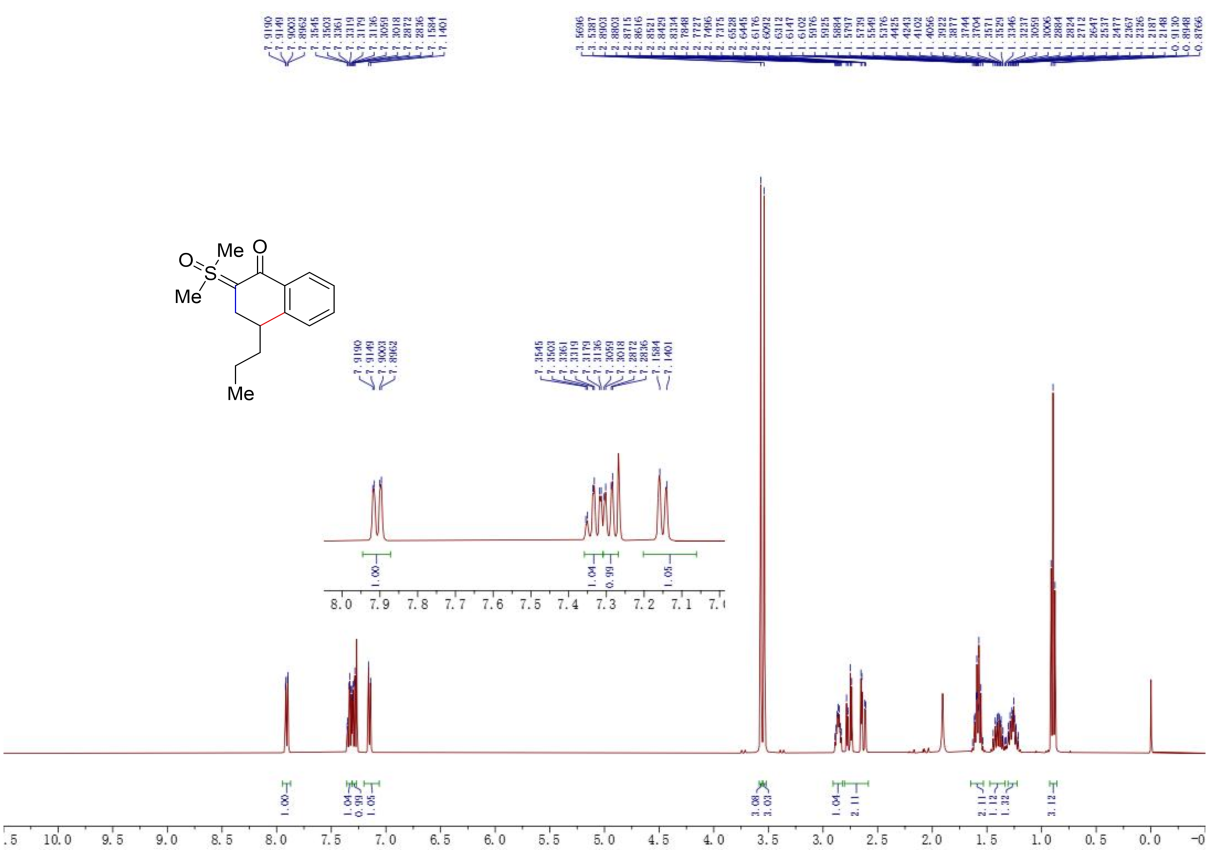

Supplementary Figure 93. ^{1}H NMR of the **5b** (400 MHz, 25 °C in $CDCl_3$)

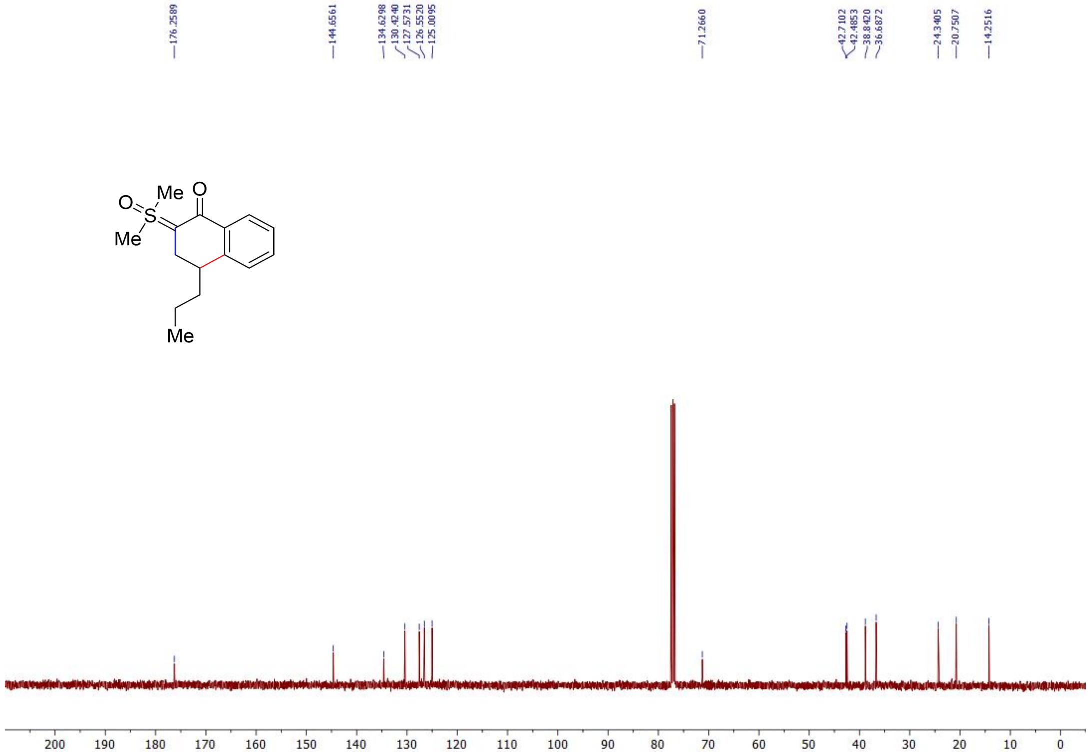

Supplementary Figure 94. ^{13}C NMR of the **5b** (101 MHz, 25 °C in $CDCl_3$)

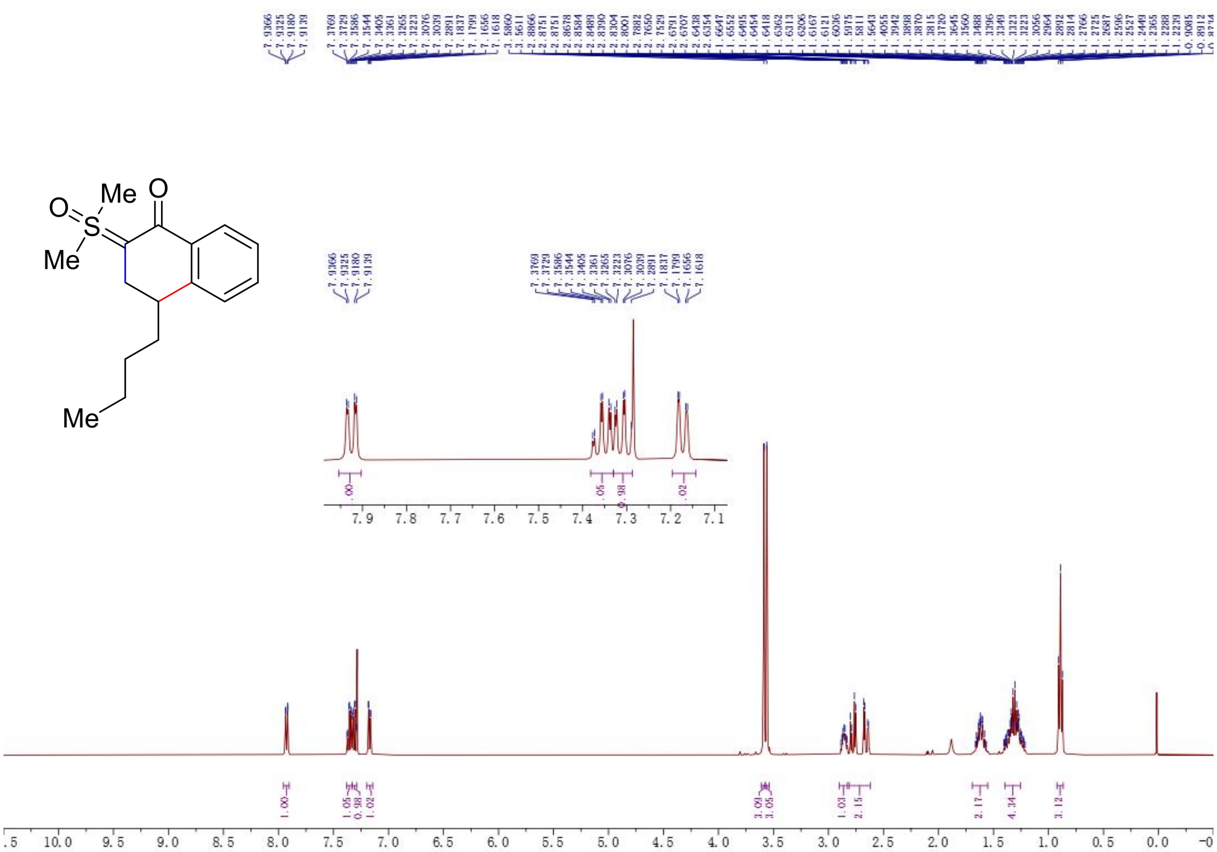

Supplementary Figure 95. ^{1}H NMR of the **5c** (400 MHz, 25 °C in $CDCl_3$)

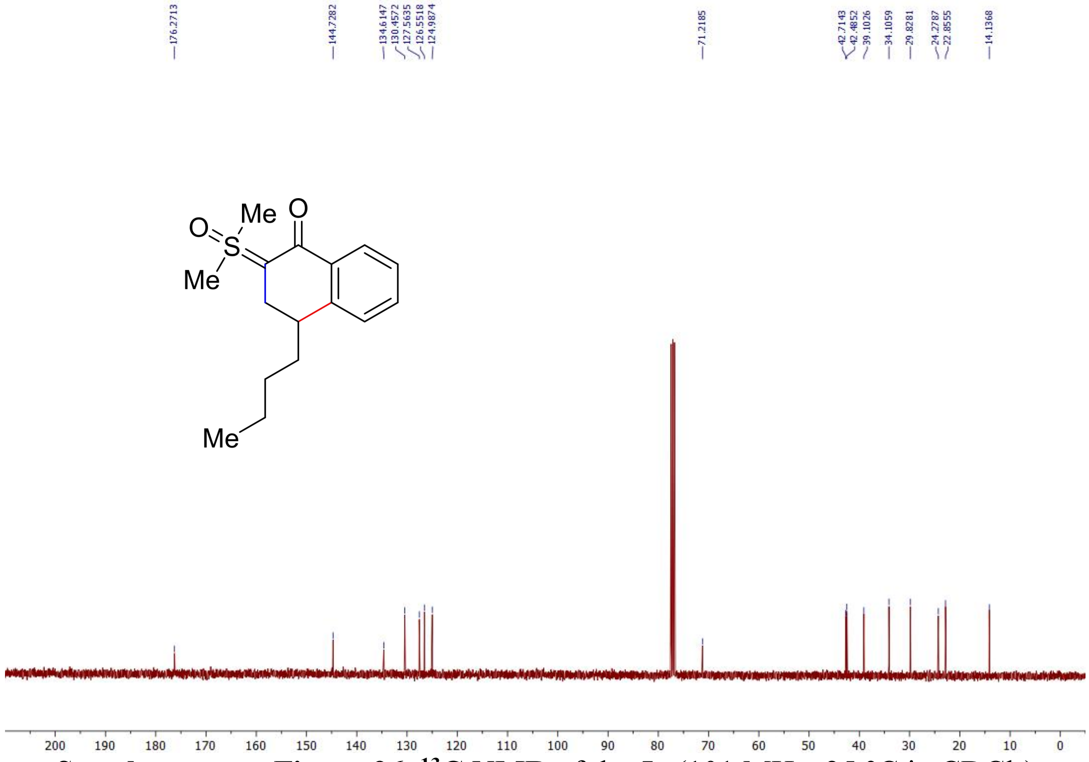

Supplementary Figure 96. ^{13}C NMR of the **5c** (101 MHz, 25 °C in $CDCl_3$)

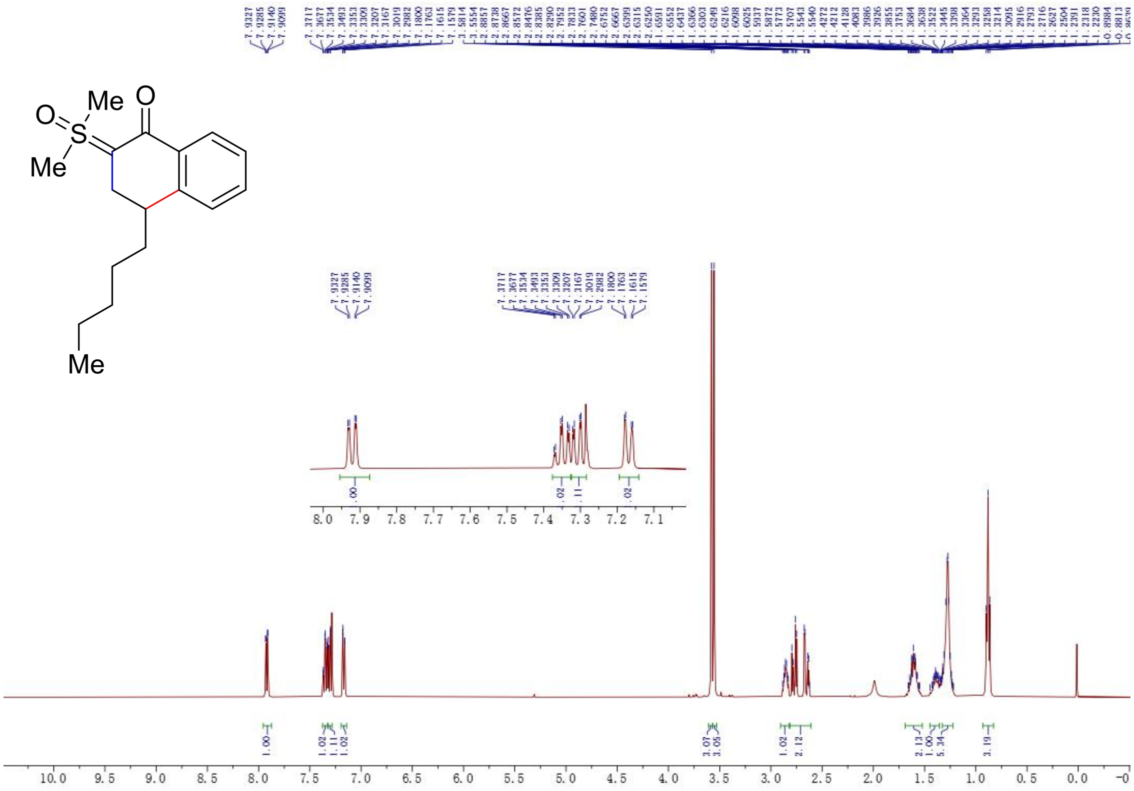

Supplementary Figure 97. ^{1}H NMR of the **5d** (400 MHz, 25 °C in $CDCl_3$)

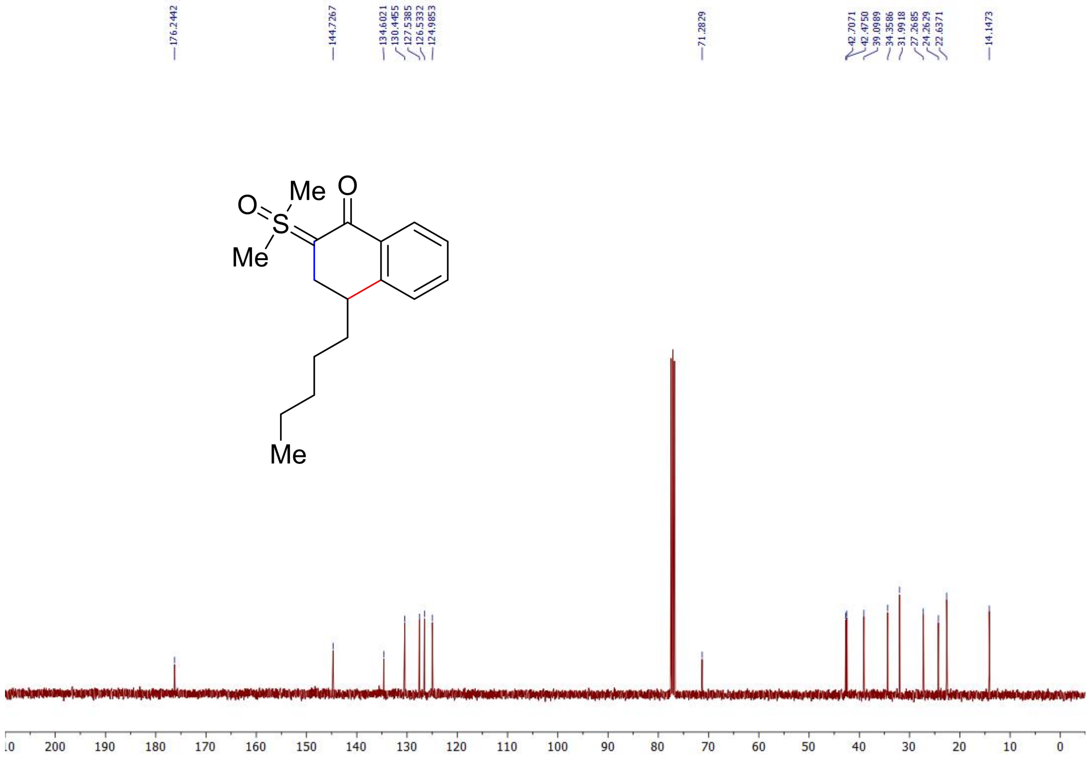

Supplementary Figure 98. ^{13}C NMR of the **5d** (101 MHz, 25 °C in $CDCl_3$)

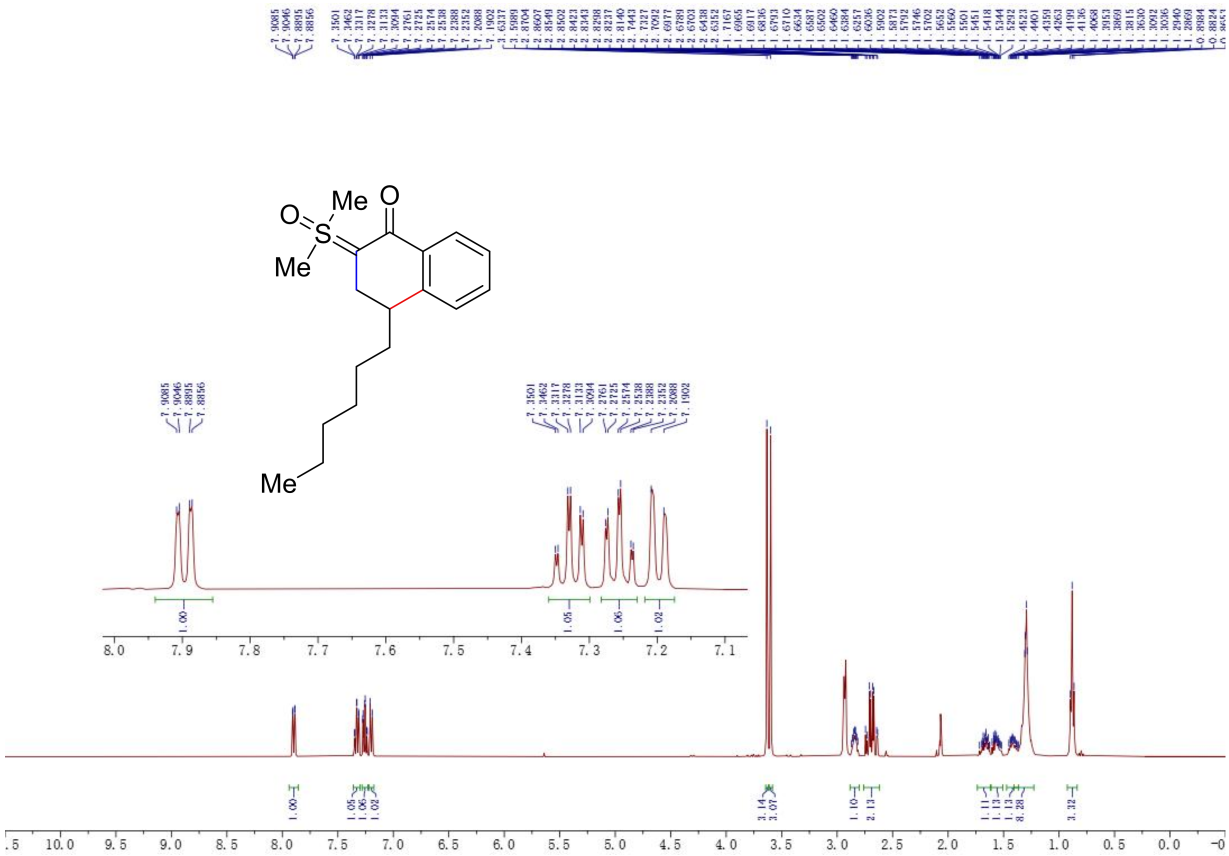

Supplementary Figure 99. ^{1}H NMR of the 5e (400 MHz, 25 °C in Acetone-d_6)

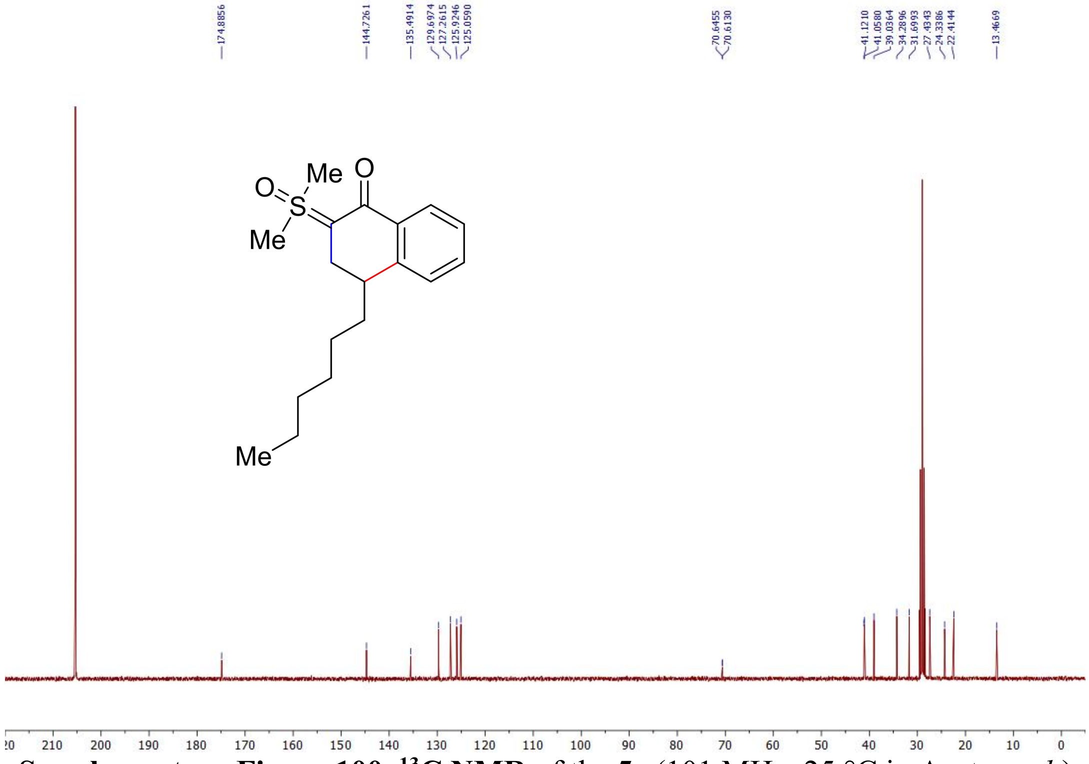

Supplementary Figure 100. ^{13}C NMR of the 5e (101 MHz, 25 °C in Acetone-d_6)

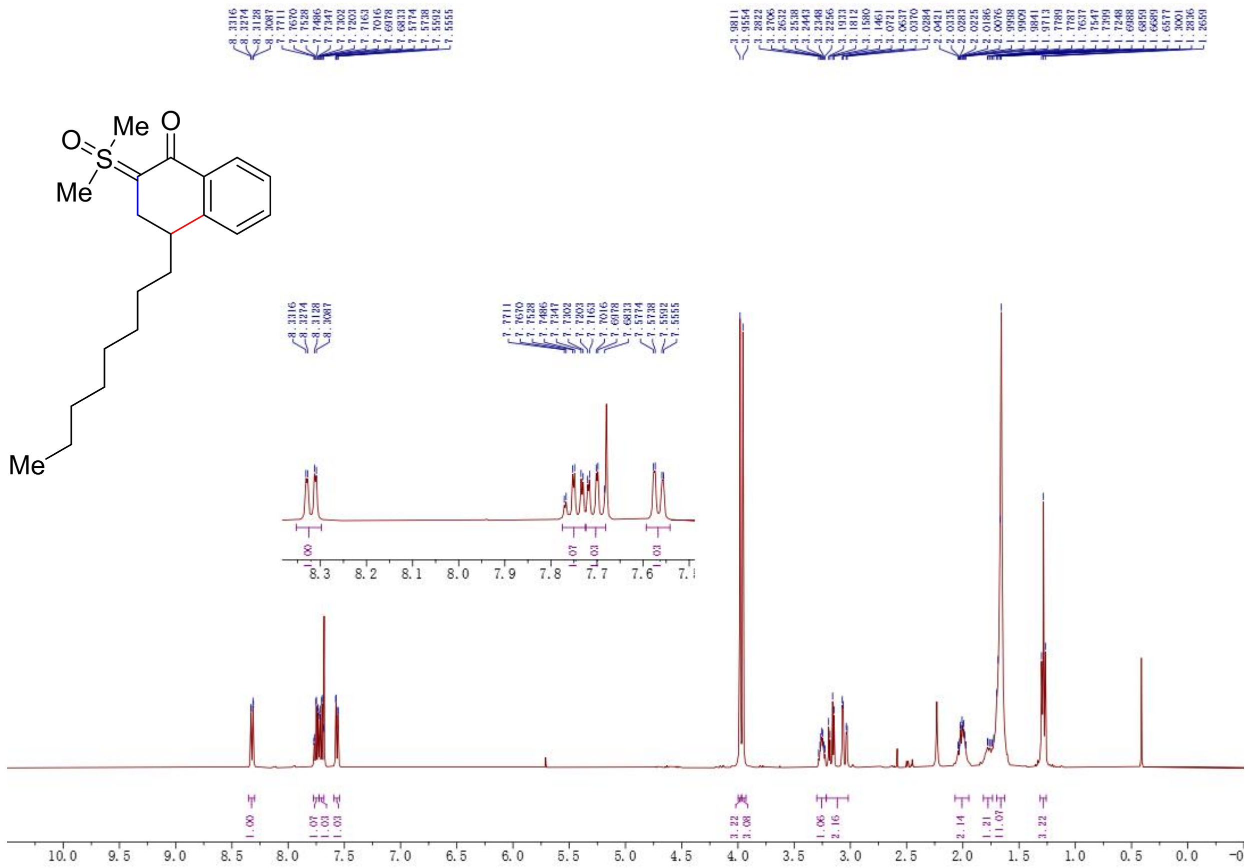

Supplementary Figure 101. ^{1}**H NMR** of the **5f** (400 MHz, 25 °C in $CDCl_3$)

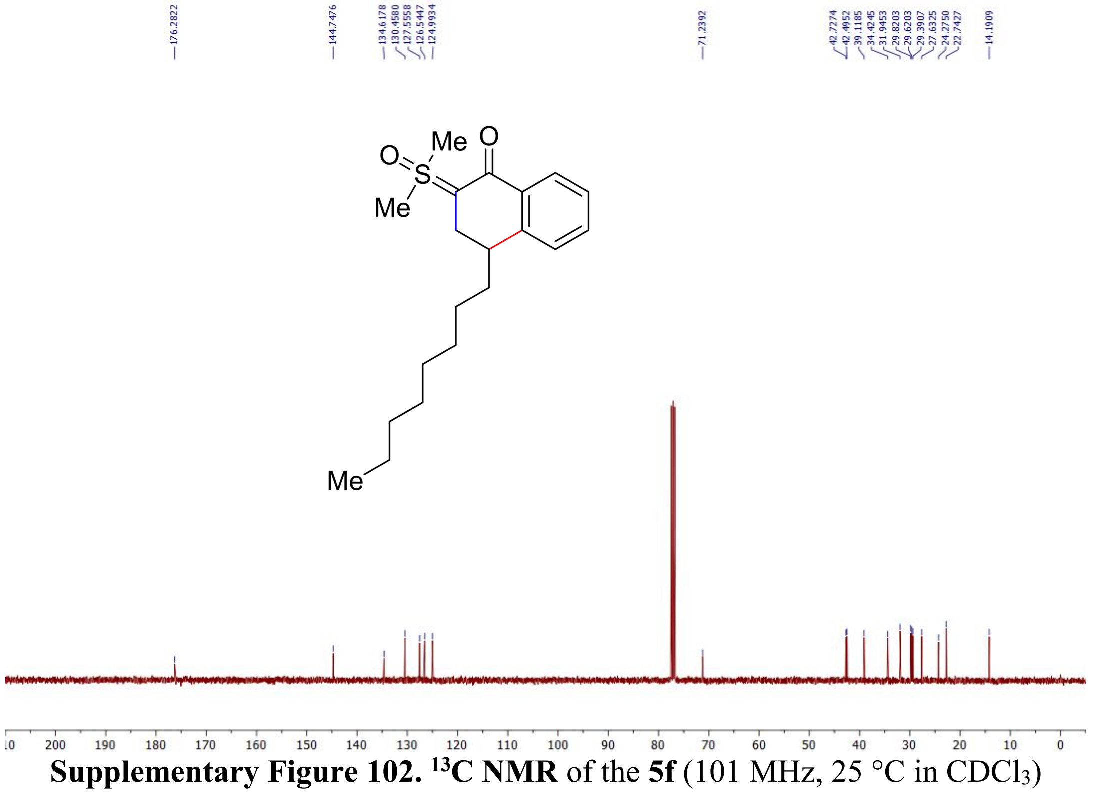

Supplementary Figure 102. ^{13}**C NMR** of the **5f** (101 MHz, 25 °C in $CDCl_3$)

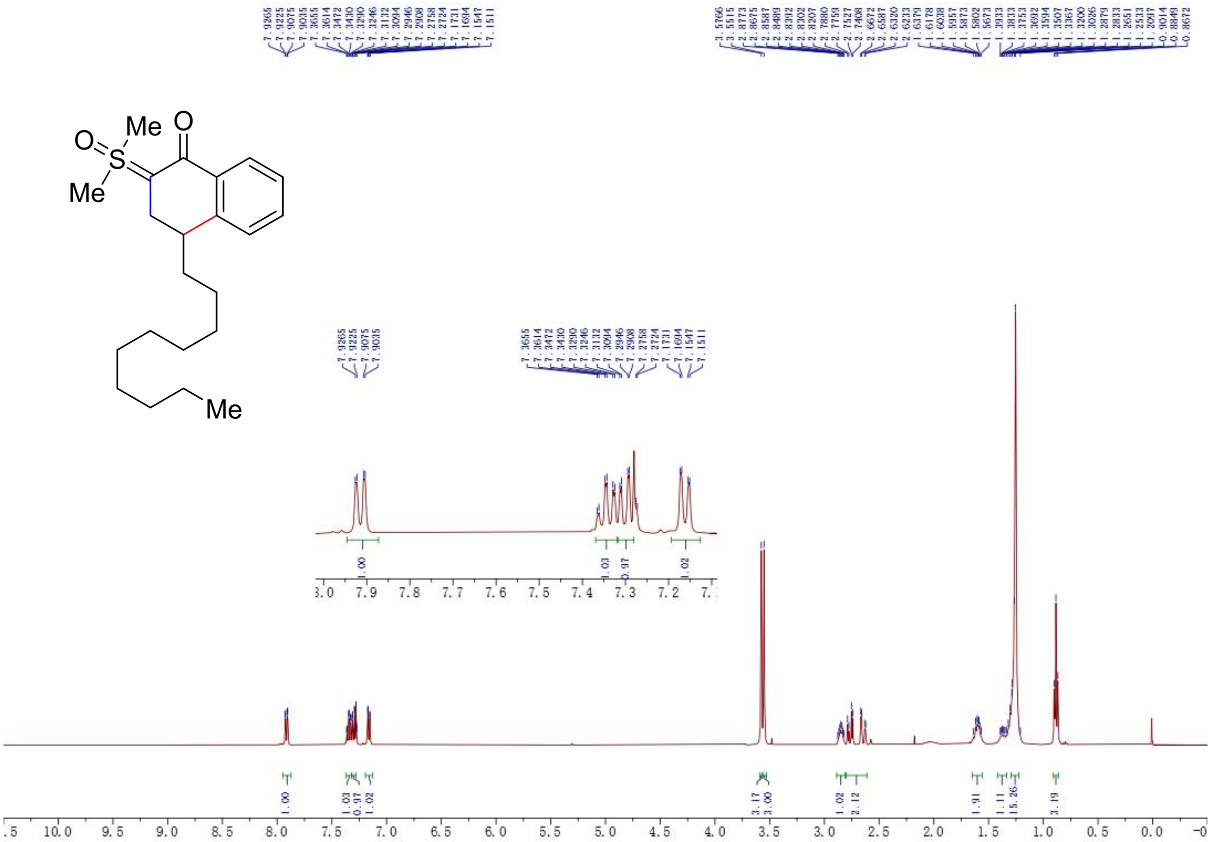

Supplementary Figure 103. ^{1}H NMR of the **5g** (400 MHz, 25 °C in $CDCl_3$)

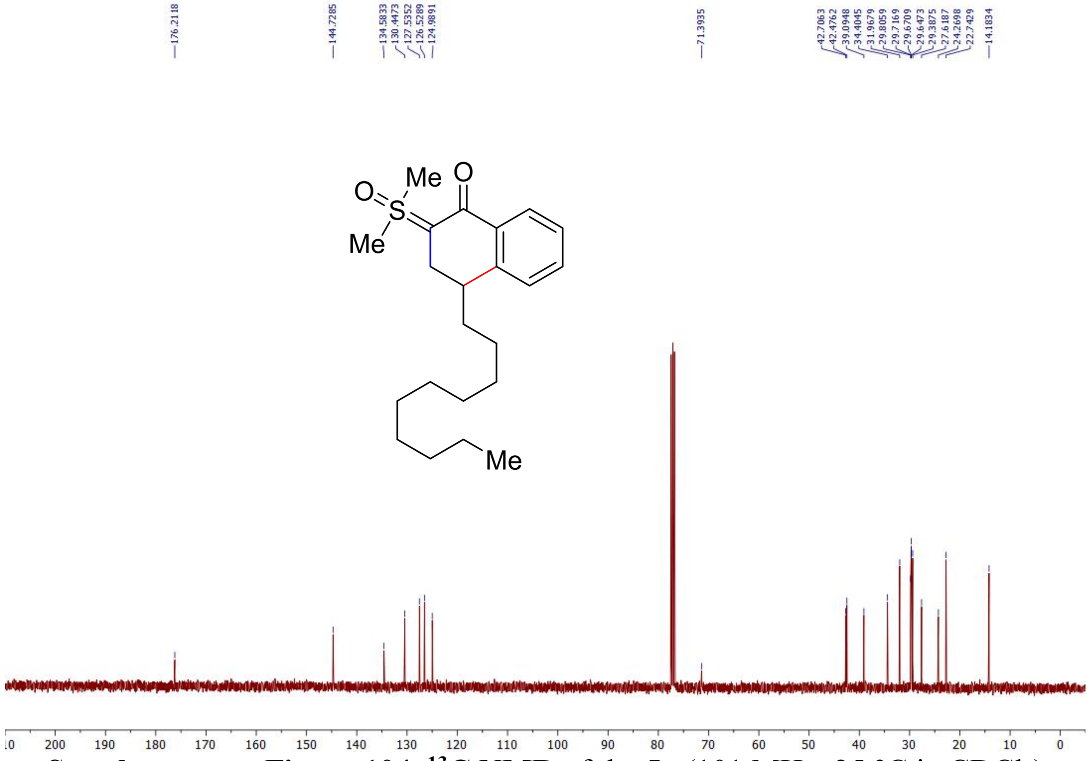

Supplementary Figure 104. ^{13}C NMR of the **5g** (101 MHz, 25 °C in $CDCl_3$)

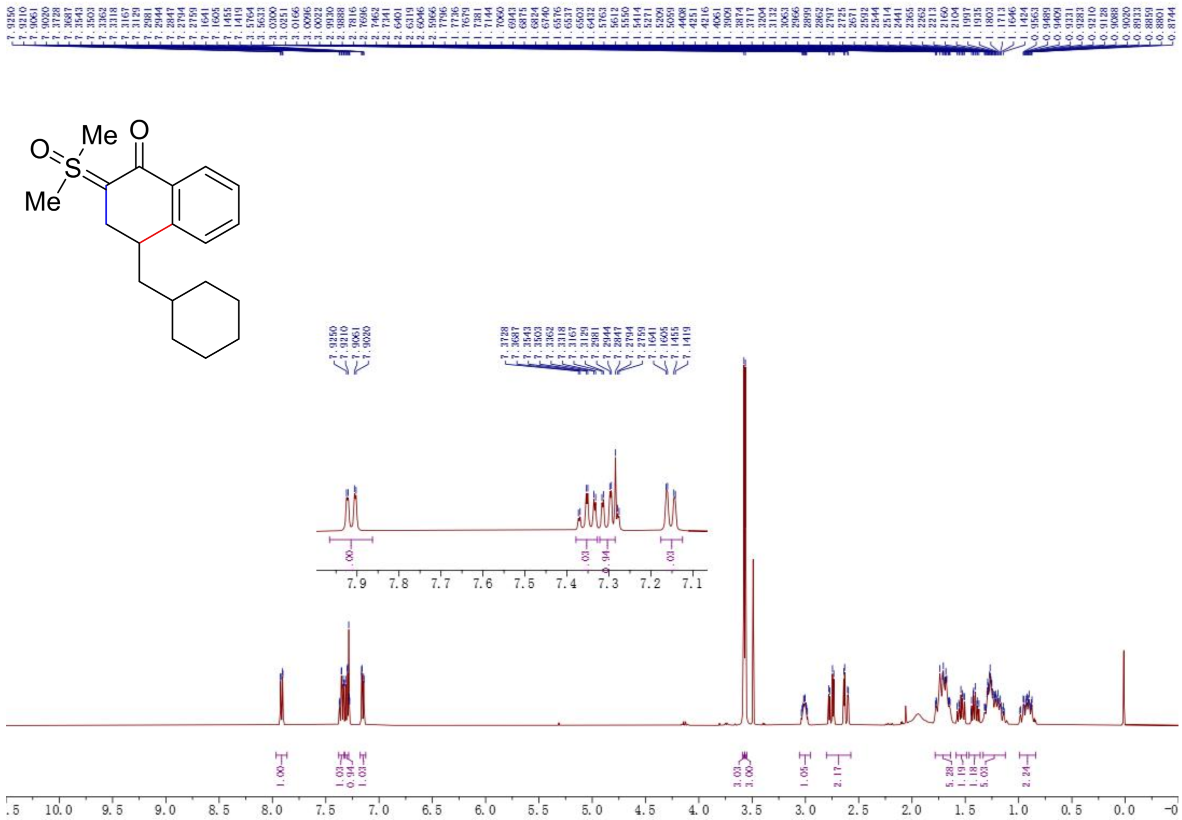

Supplementary Figure 105. ^{1}**H NMR** of the **5h** (400 MHz, 25 °C in $CDCl_3$)

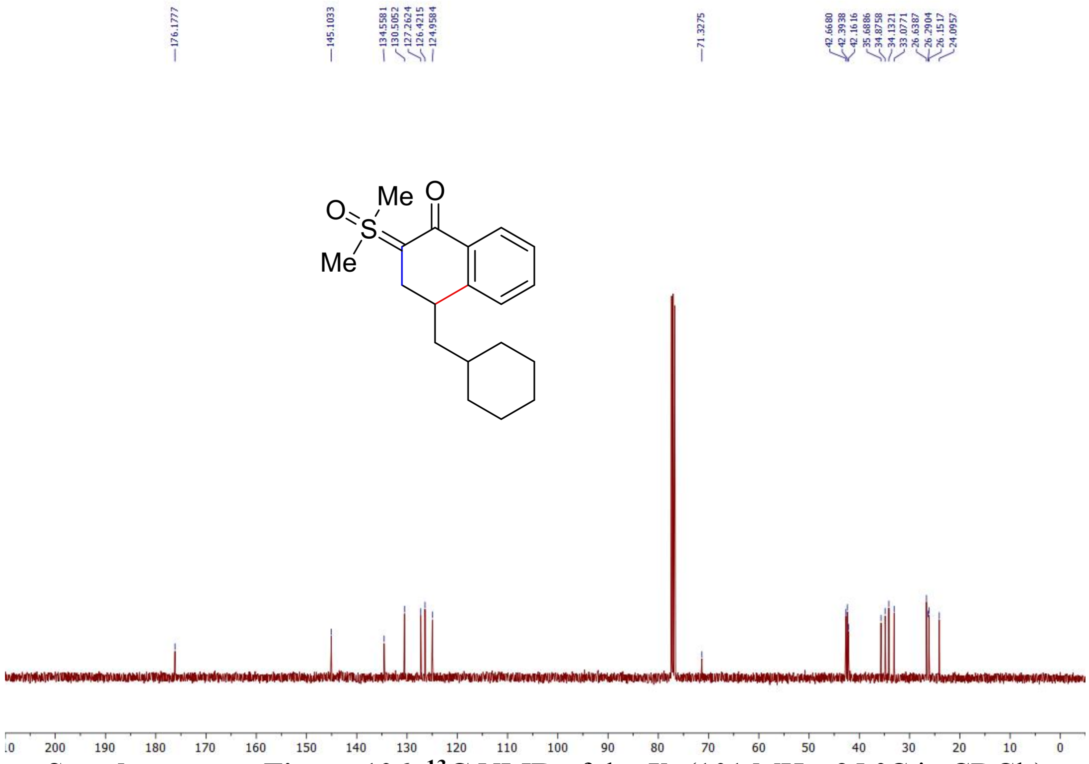

Supplementary Figure 106. ^{13}**C NMR** of the **5h** (101 MHz, 25 °C in $CDCl_3$)

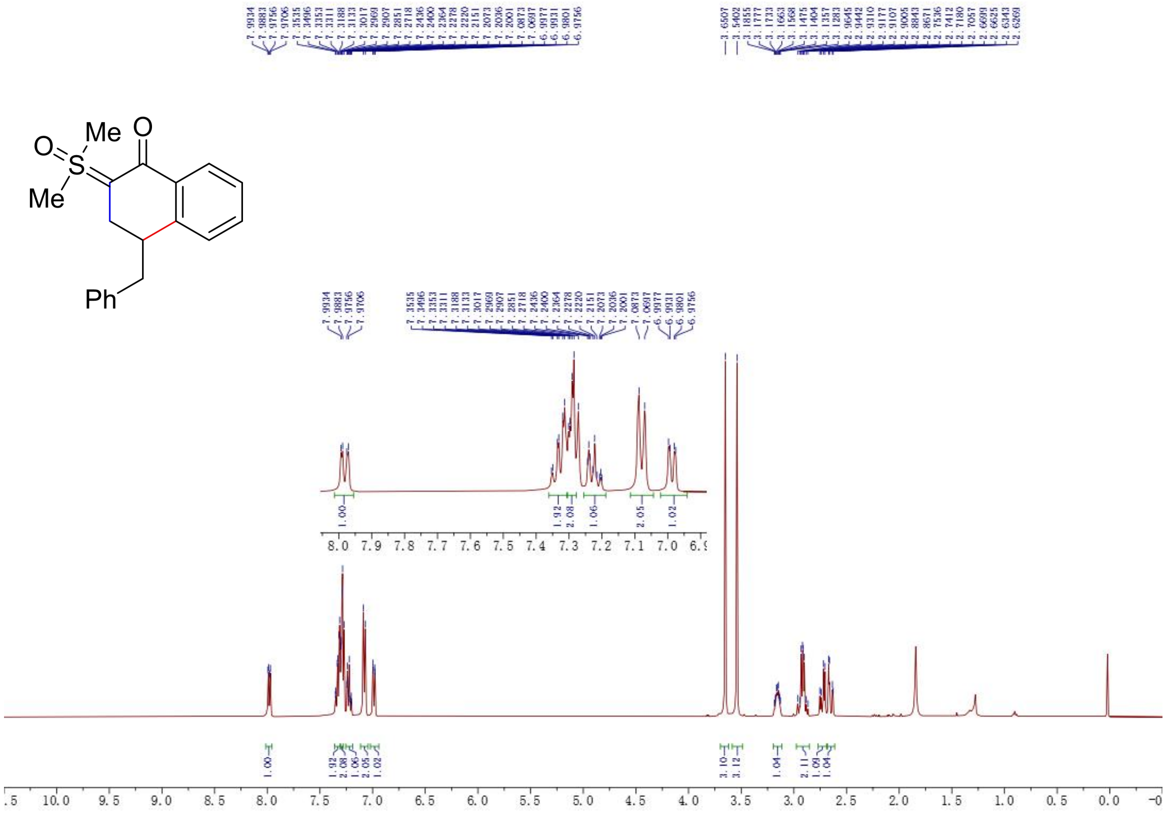

Supplementary Figure 107. ^{1}H NMR of the **5i** (400 MHz, 25 °C in $CDCl_3$)

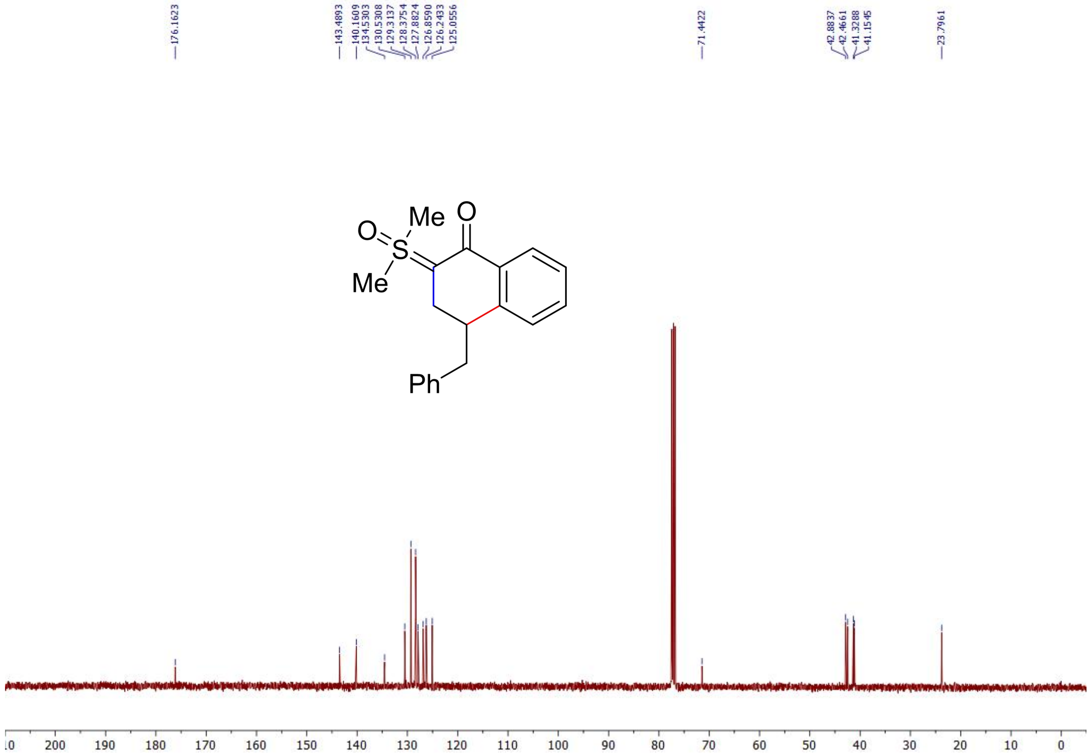

Supplementary Figure 108.^{13}C NMR of the **5i** (101 MHz, 25 °C in $CDCl_3$)

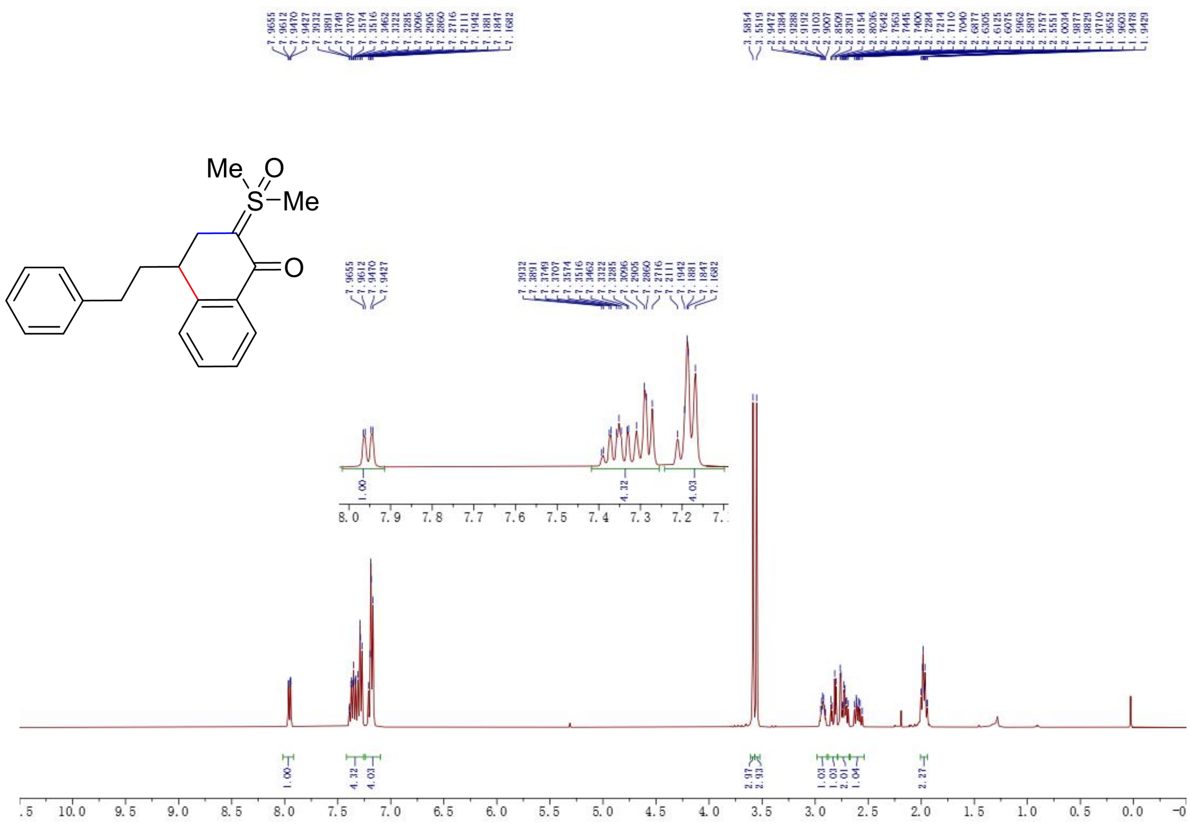

Supplementary Figure 109. ^{1}H NMR of the **5j** (400 MHz, 25 °C in $CDCl_3$)

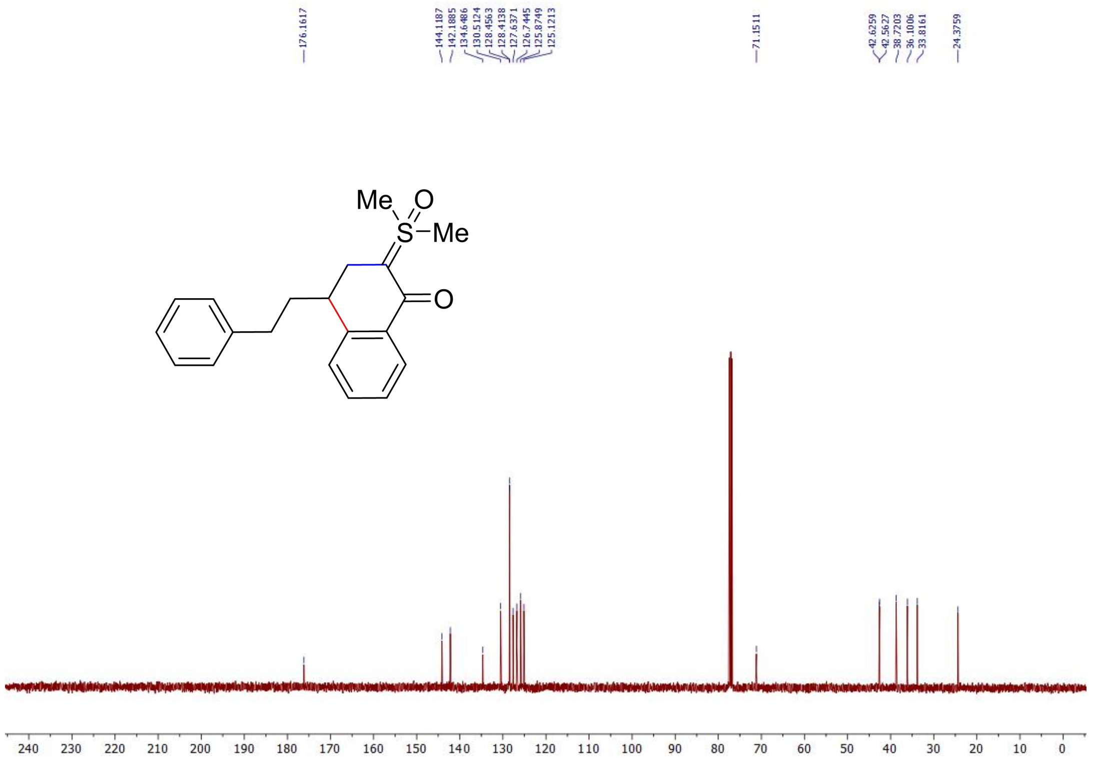

Supplementary Figure 110. ^{13}C NMR of the **5j** (101 MHz, 25 °C in $CDCl_3$)

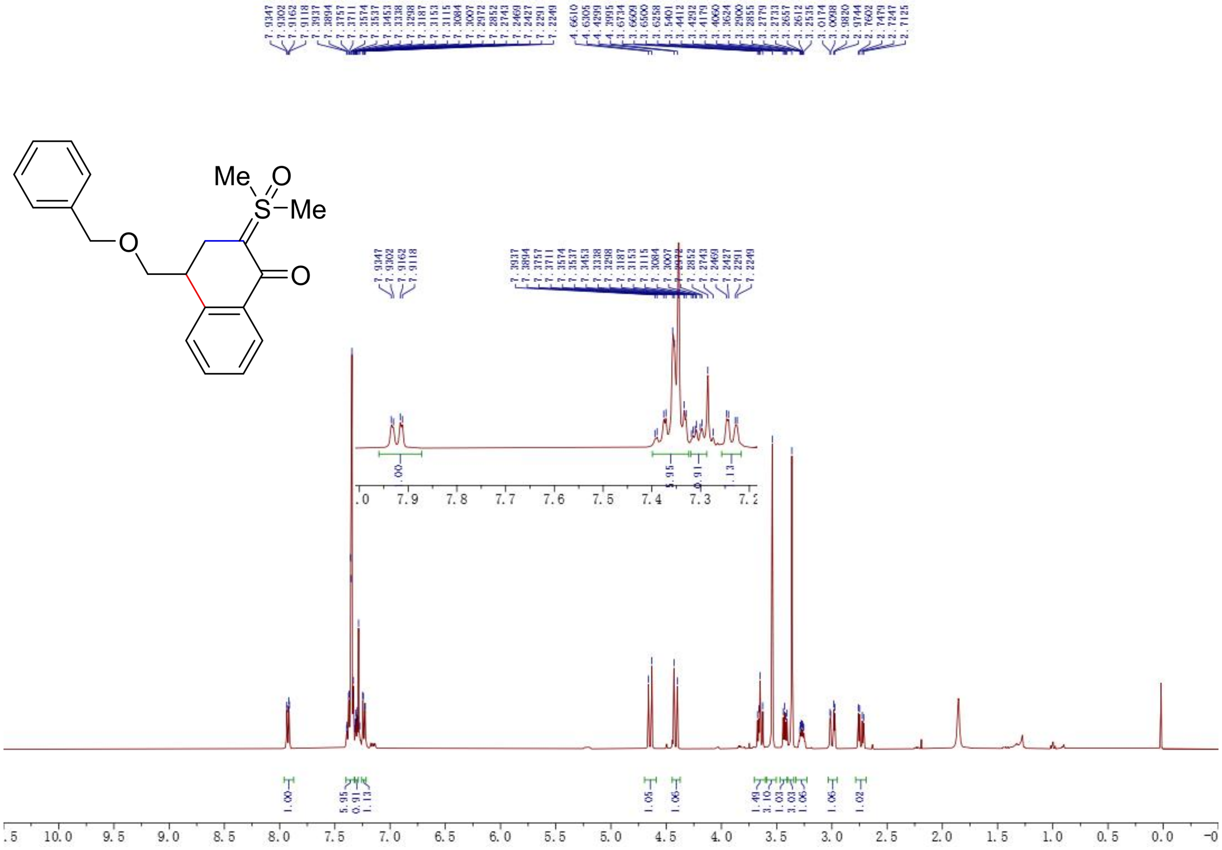

Supplementary Figure 111. ^{1}**H NMR** of the **5k** (400 MHz, 25 °C in $CDCl_3$)

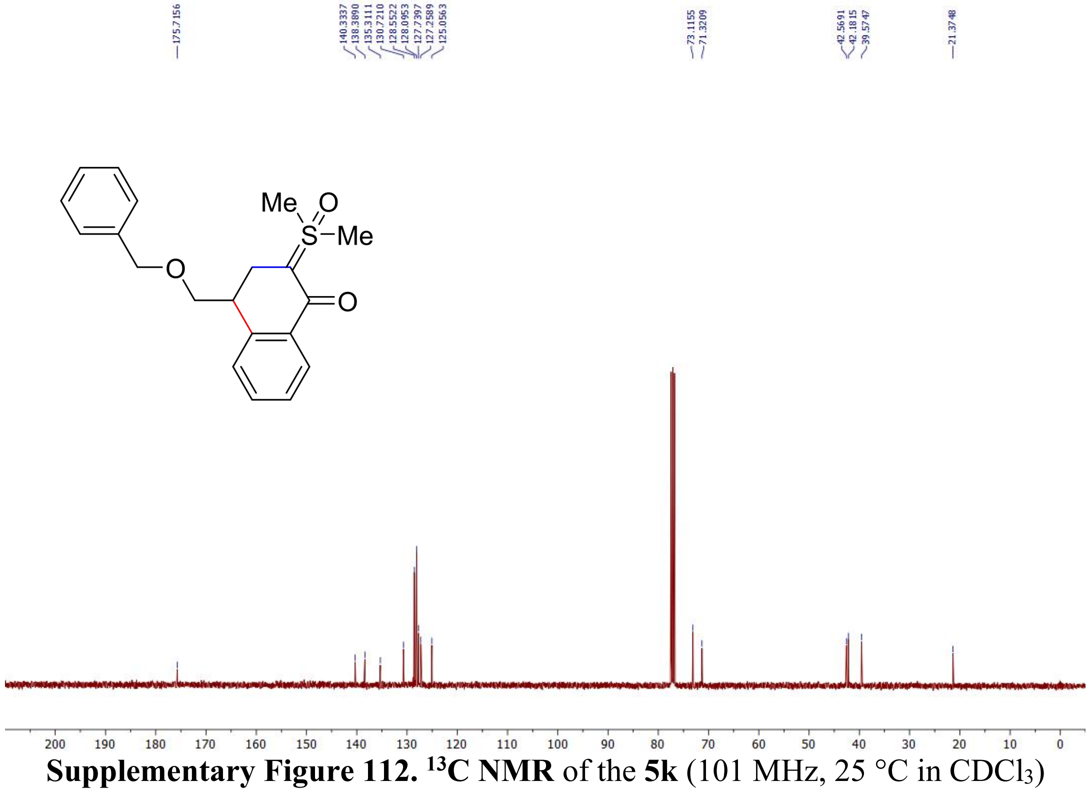

Supplementary Figure 112. ^{13}**C NMR** of the **5k** (101 MHz, 25 °C in $CDCl_3$)

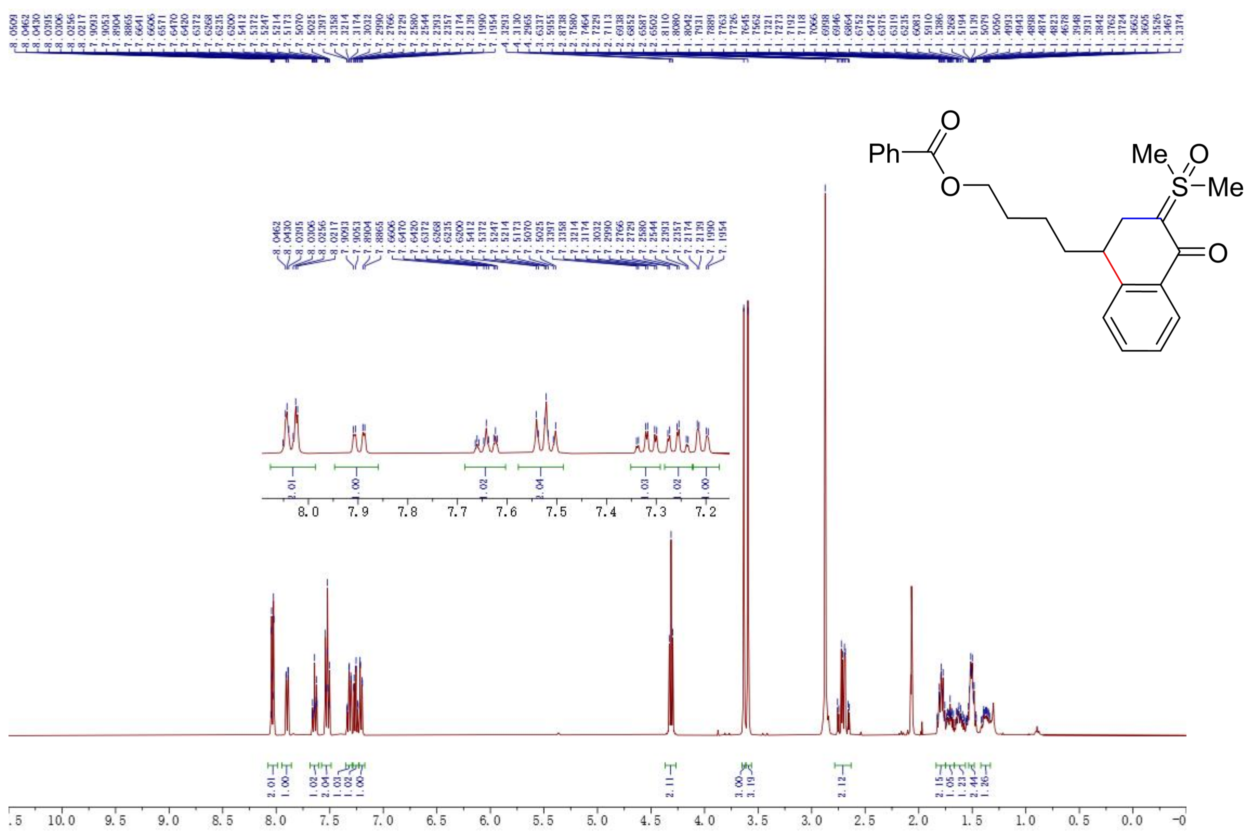

Supplementary Figure 113. ^{1}H NMR of the 5l (400 MHz, 25 °C in Acetone-d_6)

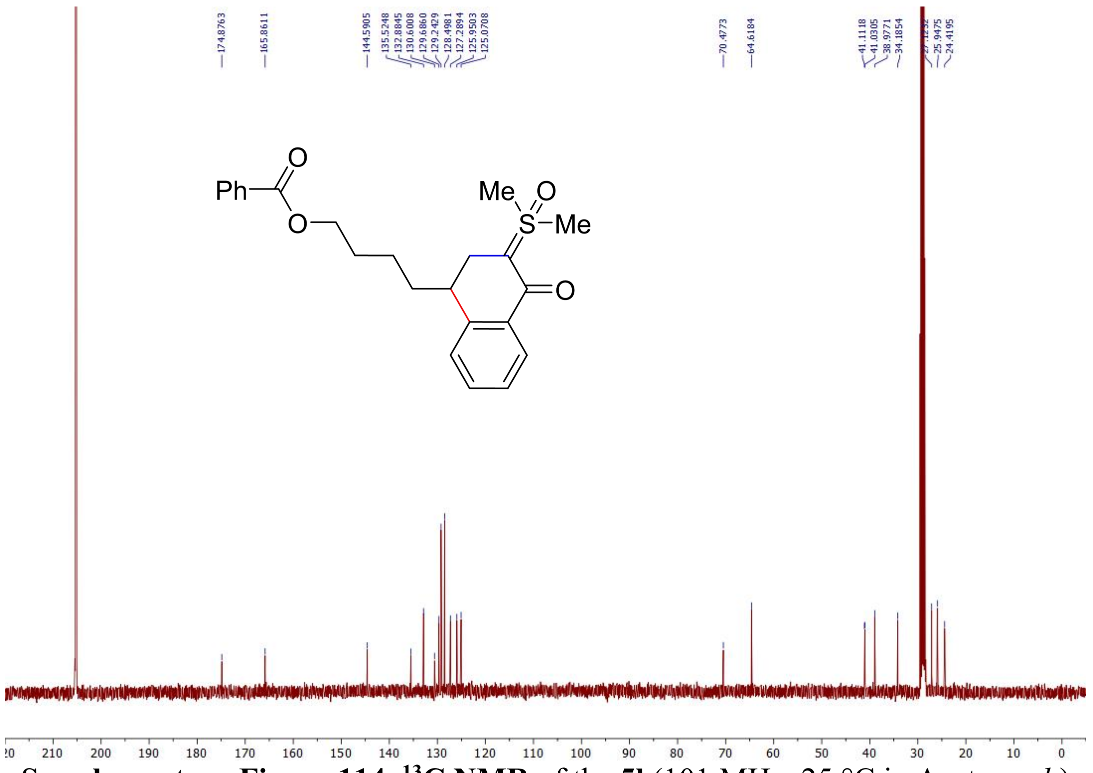

Supplementary Figure 114. ^{13}C NMR of the 5l (101 MHz, 25 °C in Acetone-d_6)

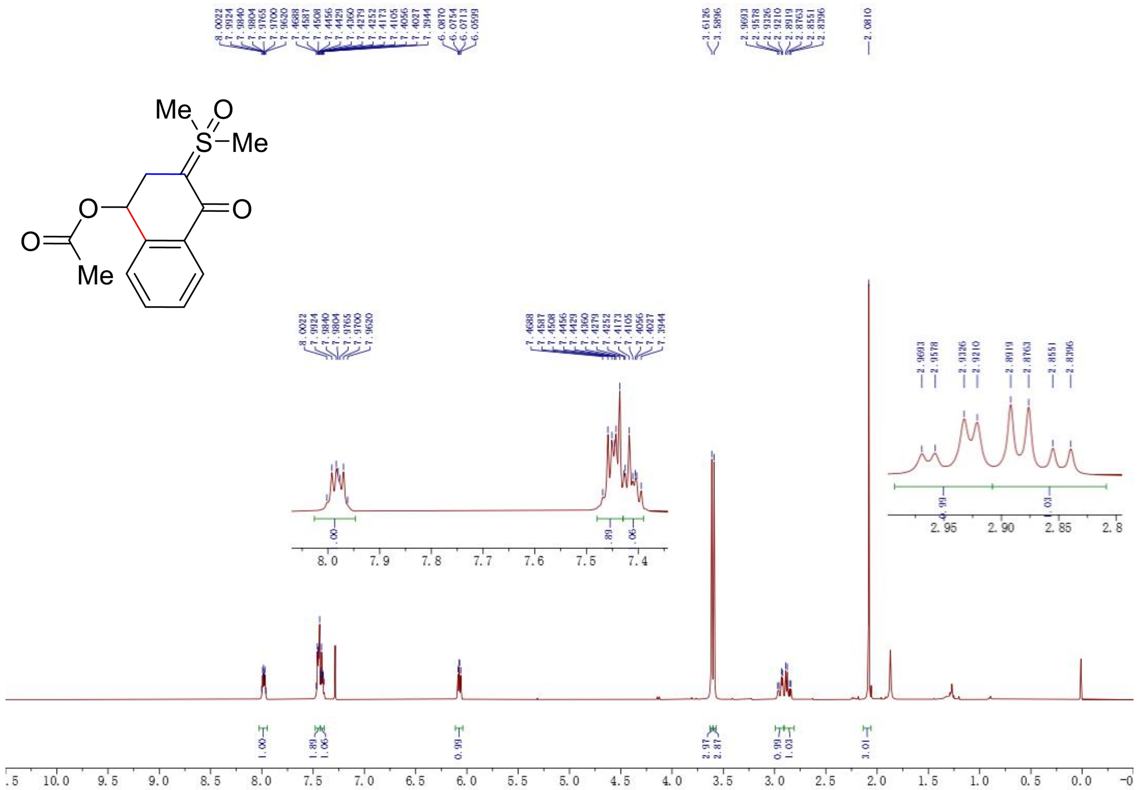

Supplementary Figure 115. **^{1}H NMR** of the **5m** (400 MHz, 25 °C in $CDCl_3$)

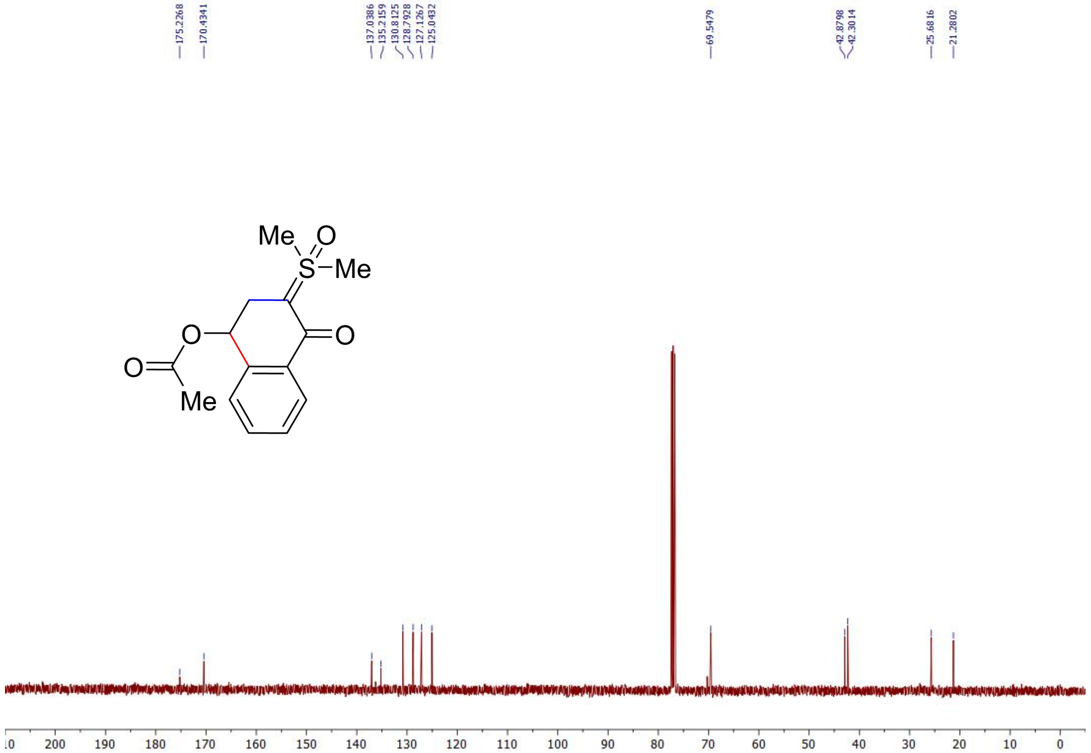

Supplementary Figure 116. **^{13}C NMR** of the **5m** (101 MHz, 25 °C in $CDCl_3$)

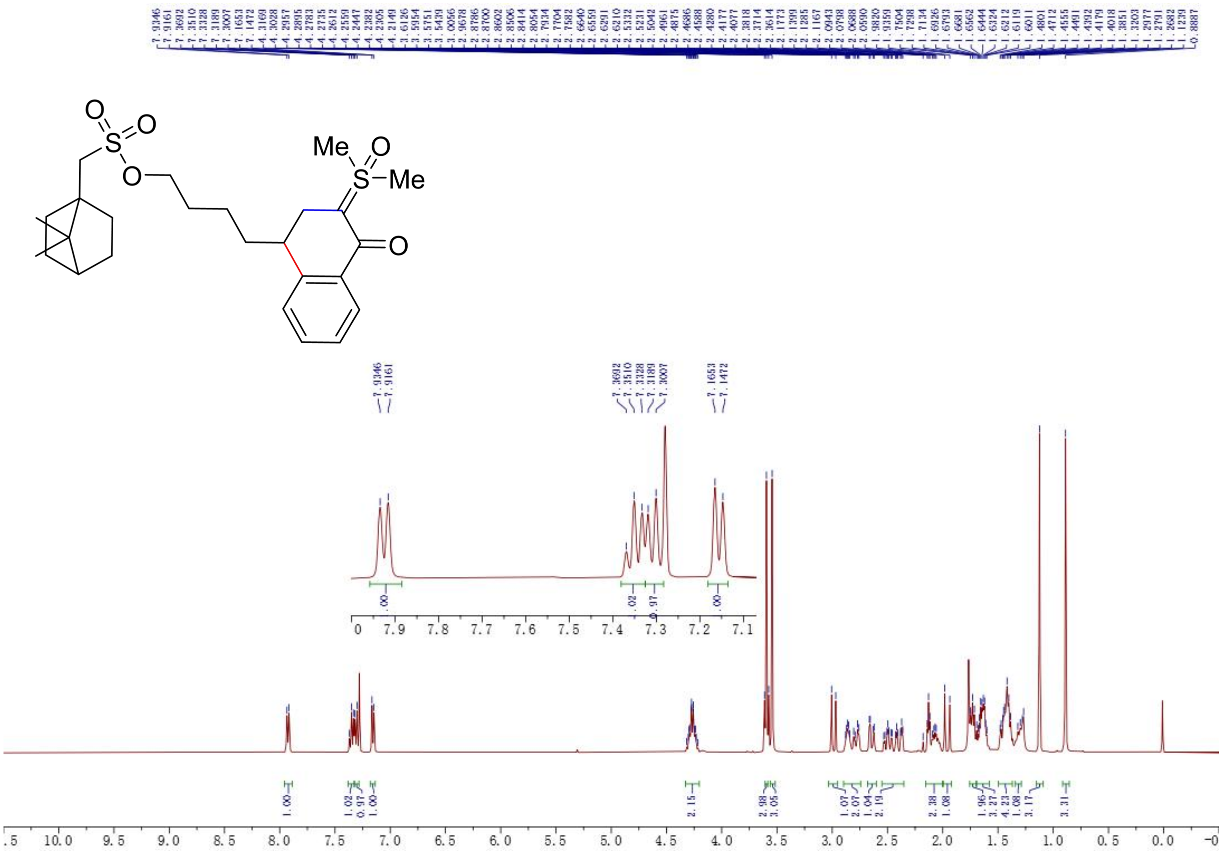

Supplementary Figure 117. ^{1}H NMR of the **5n** (400 MHz, 25 °C in $CDCl_3$)

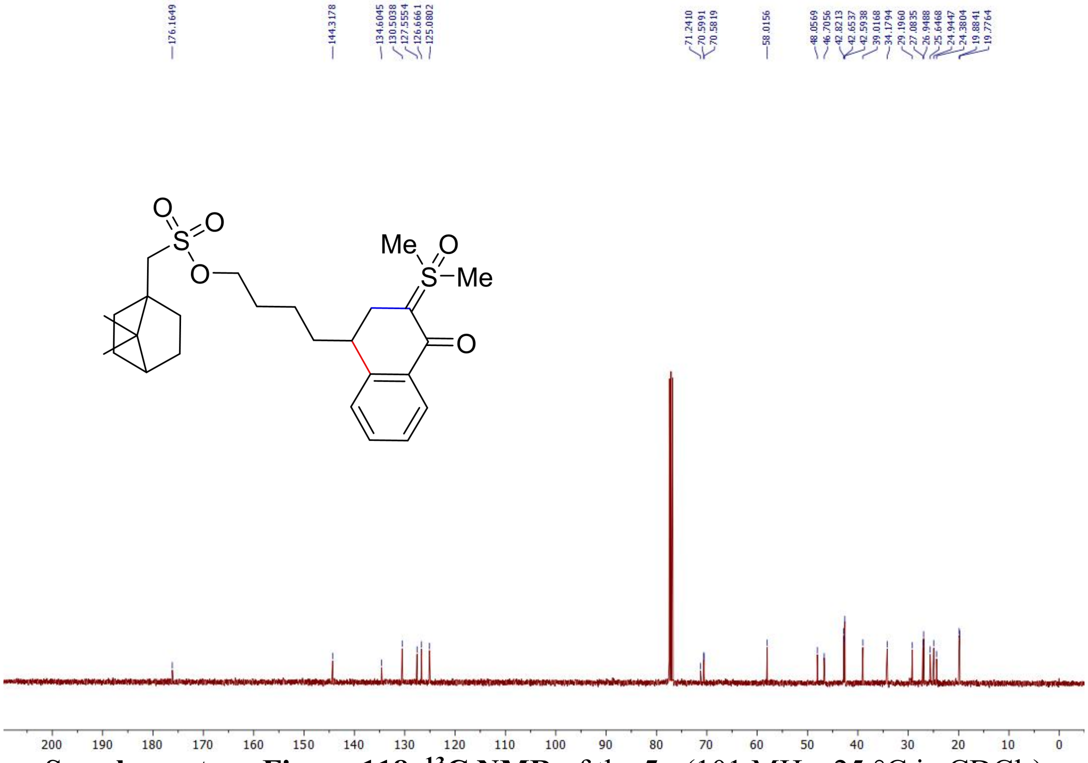

Supplementary Figure 118. ^{13}C NMR of the **5n** (101 MHz, 25 °C in $CDCl_3$)

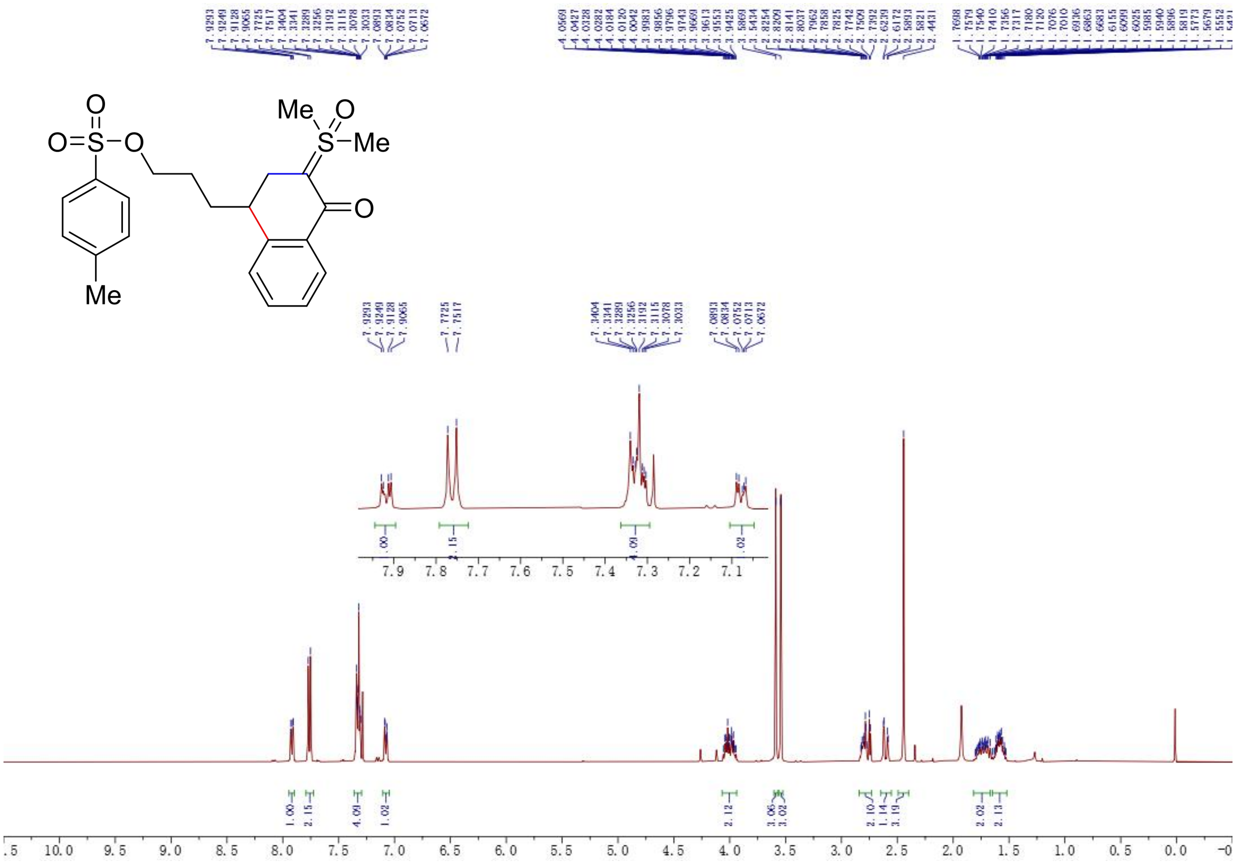

Supplementary Figure 119. ^{1}H NMR of the **5o** (400 MHz, 25 °C in $CDCl_3$)

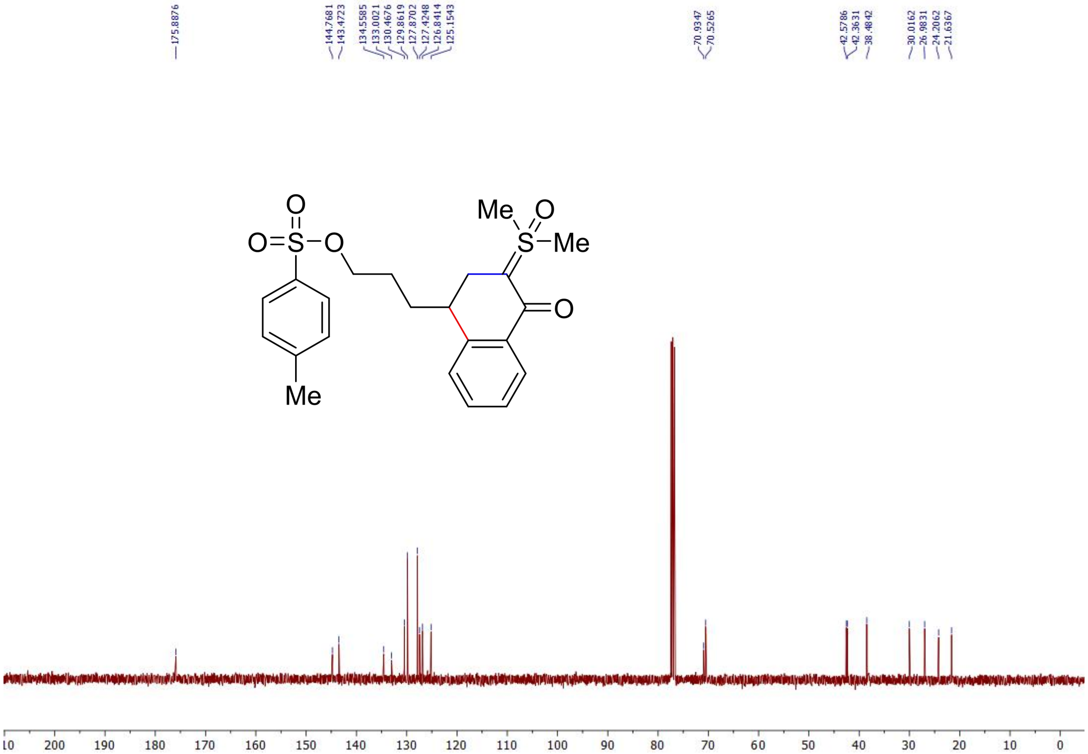

Supplementary Figure 120. ^{13}C NMR of the **5o** (101 MHz, 25 °C in $CDCl_3$)

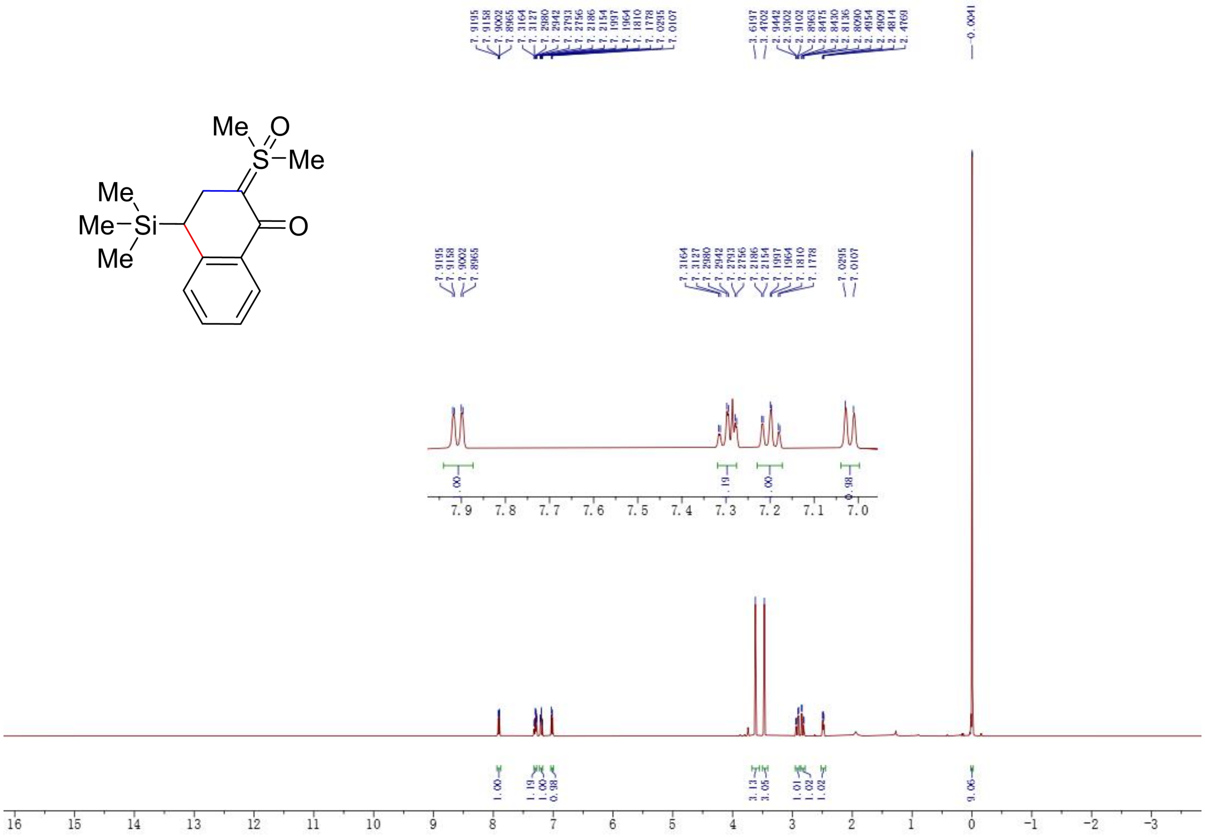

Supplementary Figure 121. **^{1}H NMR** of the **5p** (400 MHz, 25 °C in $CDCl_3$)

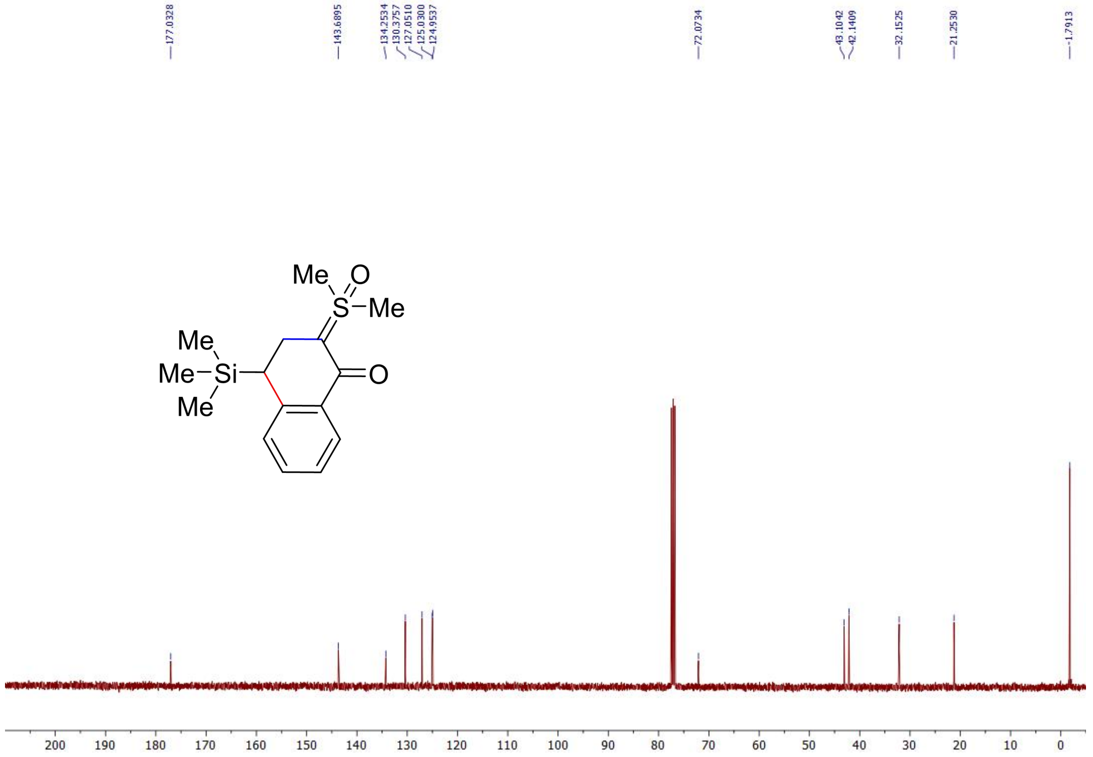

Supplementary Figure 122. **^{13}C NMR** of the **5p** (101 MHz, 25 °C in $CDCl_3$)

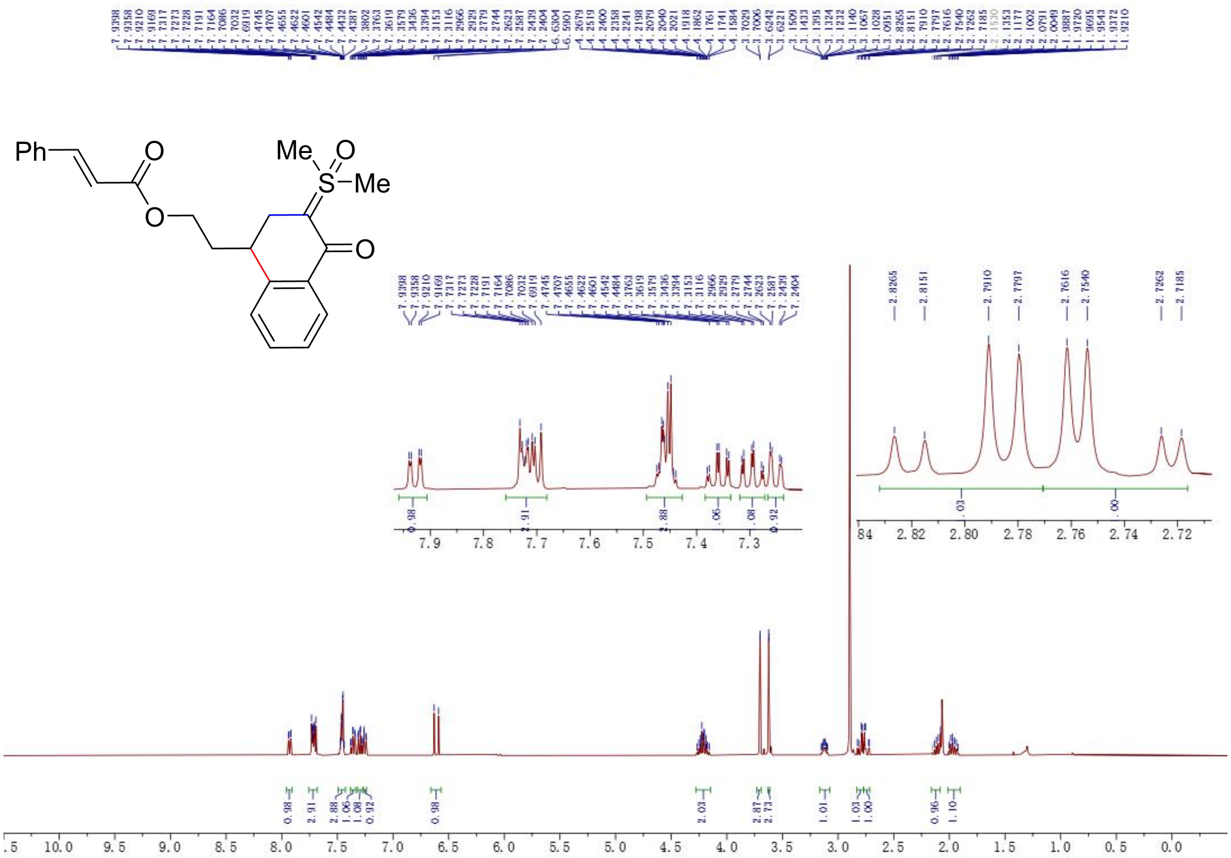

Supplementary Figure 123. ^{1}**H NMR** of the **5q** (400 MHz, 25 °C in Acetone-d_6)

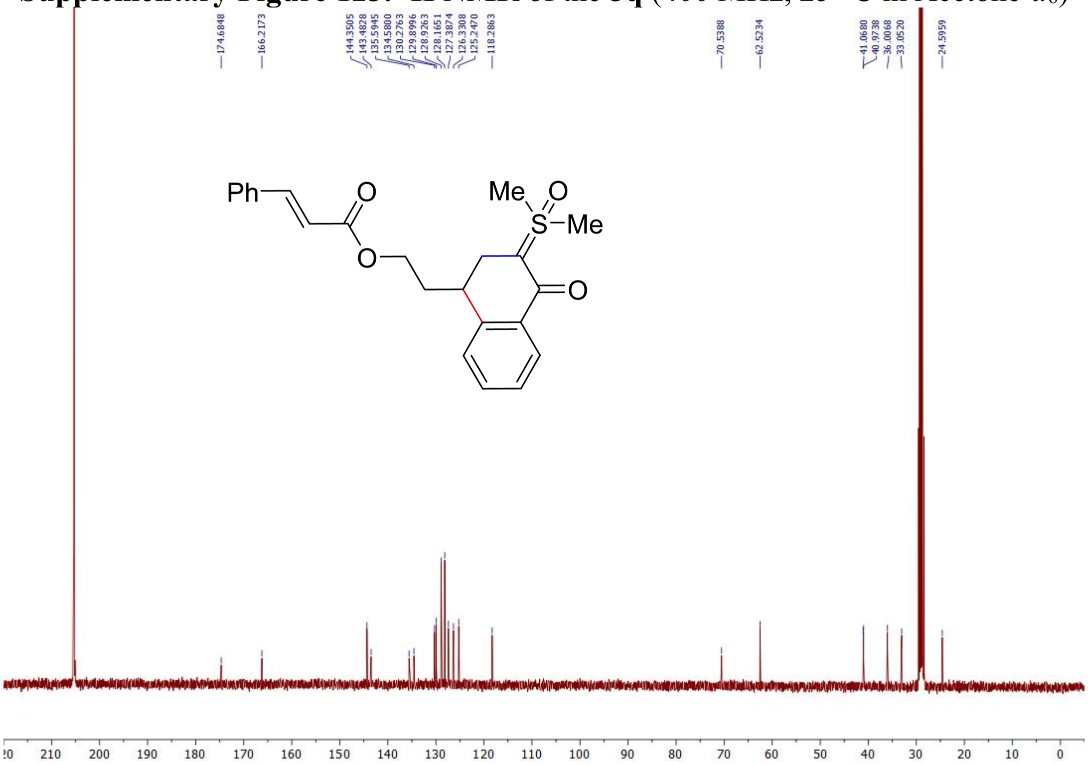

Supplementary Figure 124. ^{13}**C NMR** of the **5q** (101 MHz, 25 °C in Acetone-d_6)

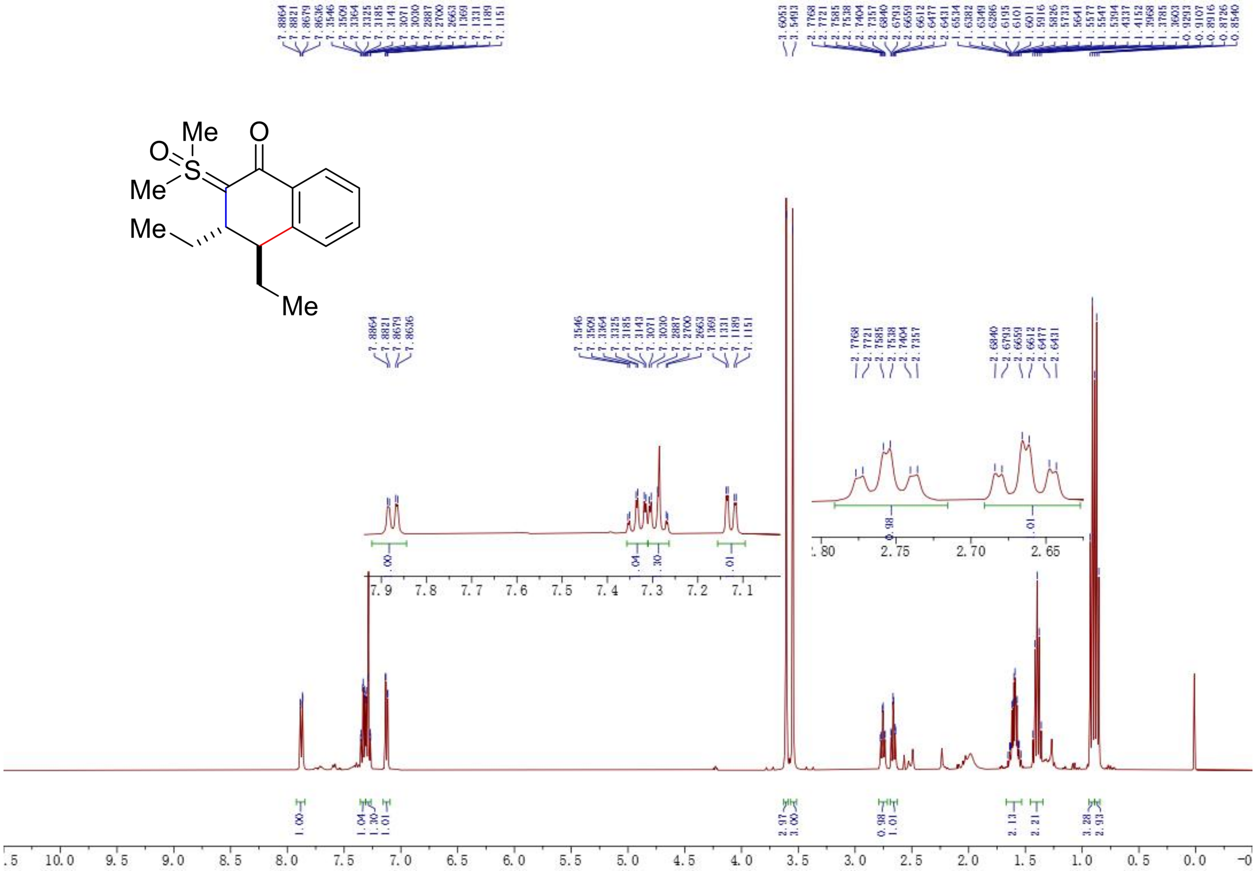

Supplementary Figure 125. ^{1}H NMR of the **5r** (400 MHz, 25 °C in $CDCl_3$)

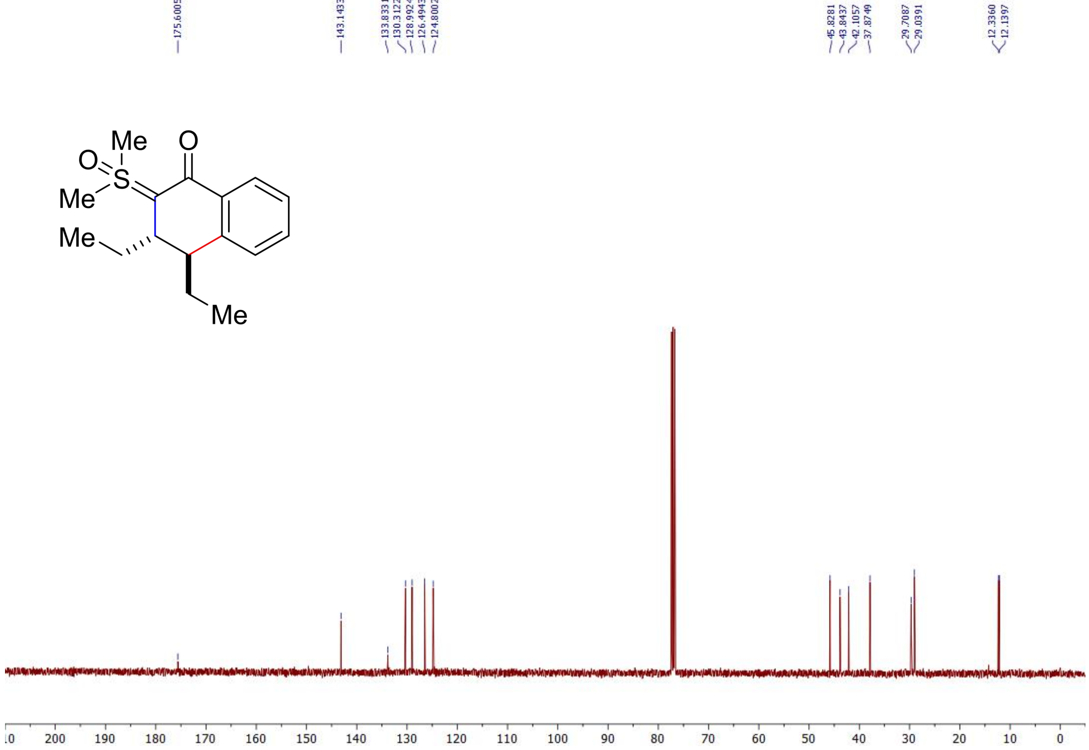

Supplementary Figure 126. ^{13}C NMR of the **5r** (101 MHz, 25 °C in $CDCl_3$)

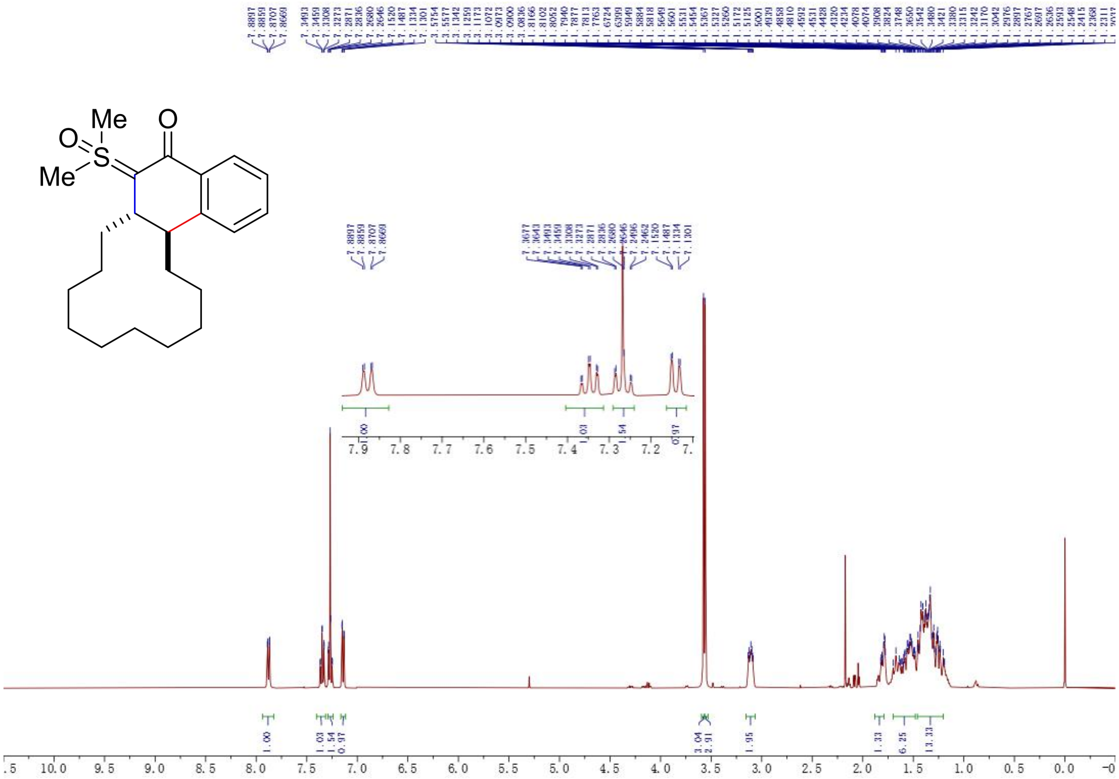

Supplementary Figure 127. ^{1}H NMR of the 5s (400 MHz, 25 °C in $CDCl_3$)

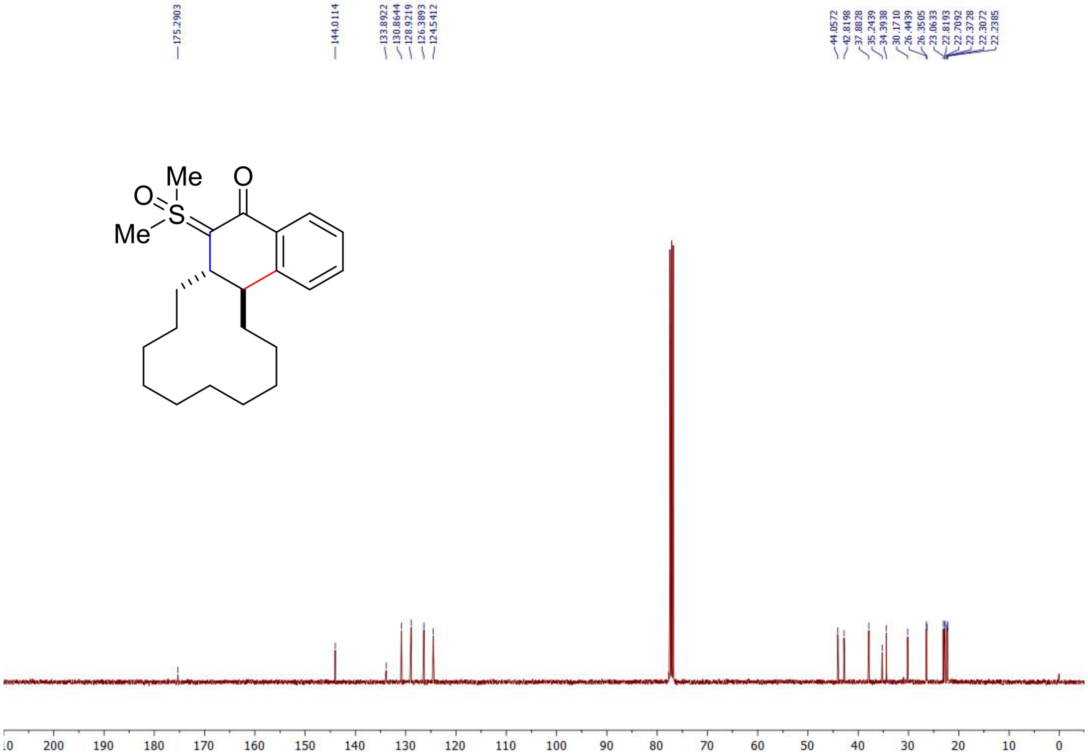

Supplementary Figure 128. ^{13}C NMR of the 5s (101 MHz, 25 °C in $CDCl_3$)

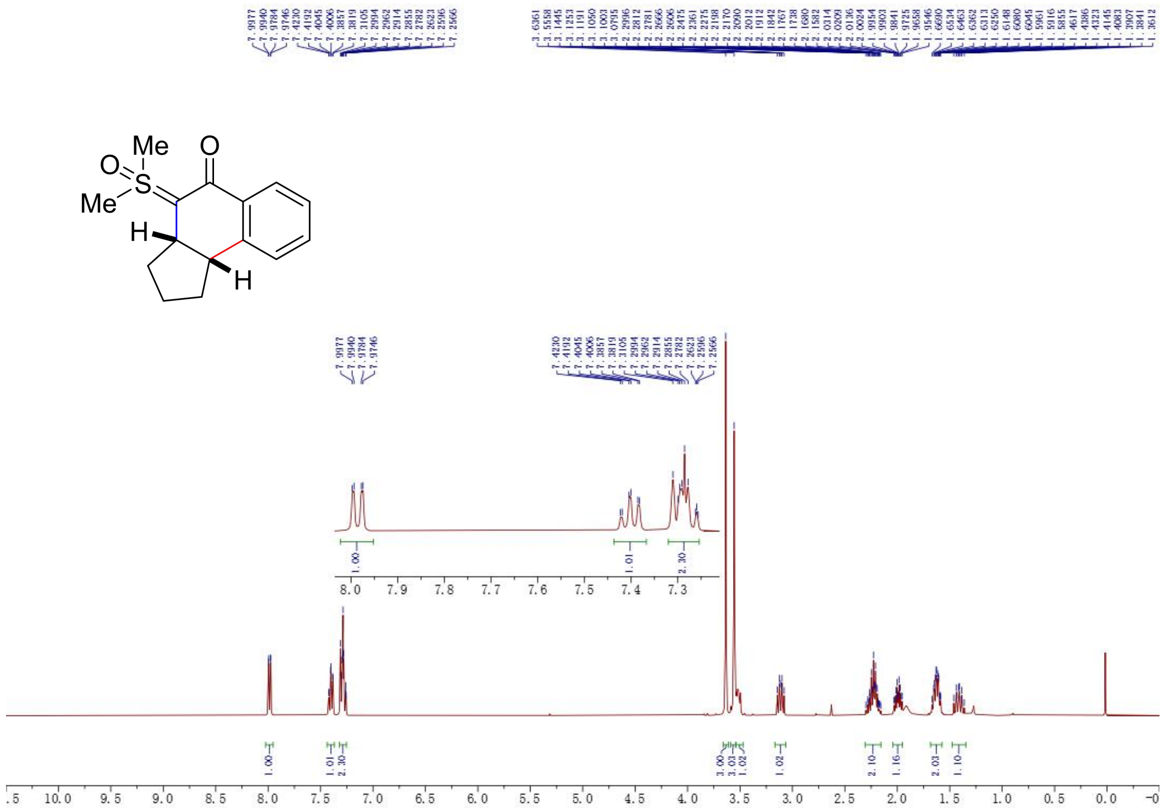

Supplementary Figure 129. ^{1}H NMR of the **5t** (400 MHz, 25 °C in $CDCl_3$)

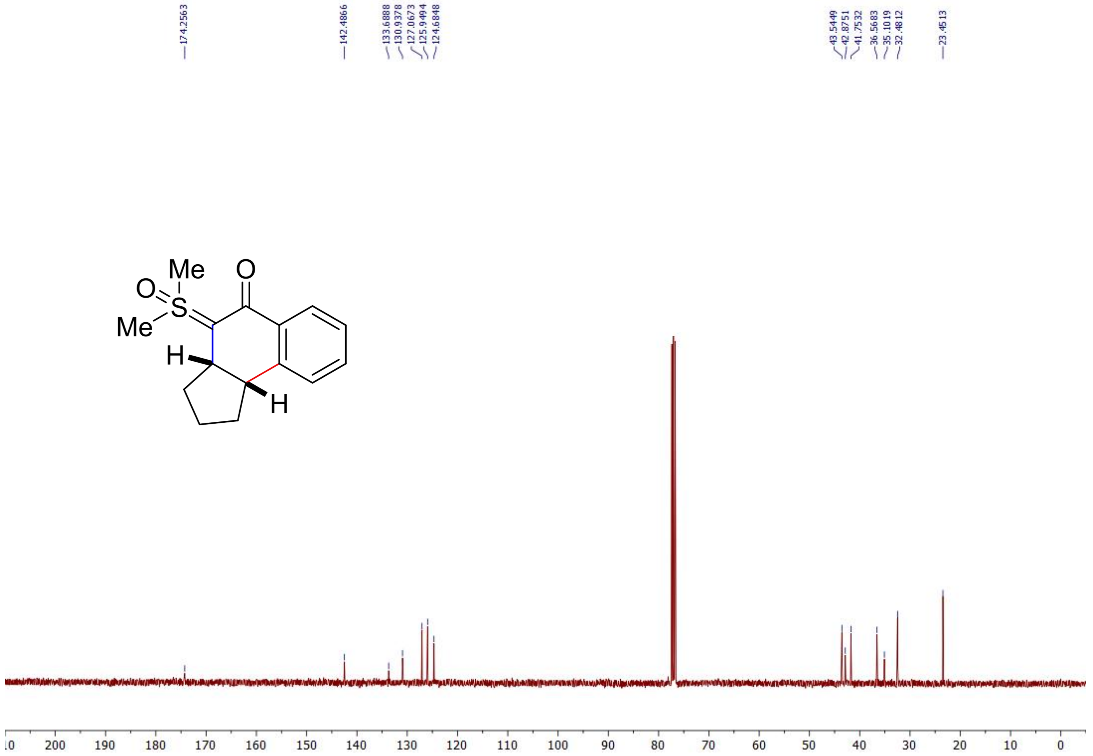

Supplementary Figure 130. ^{13}C NMR of the **5t** (101 MHz, 25 °C in $CDCl_3$)

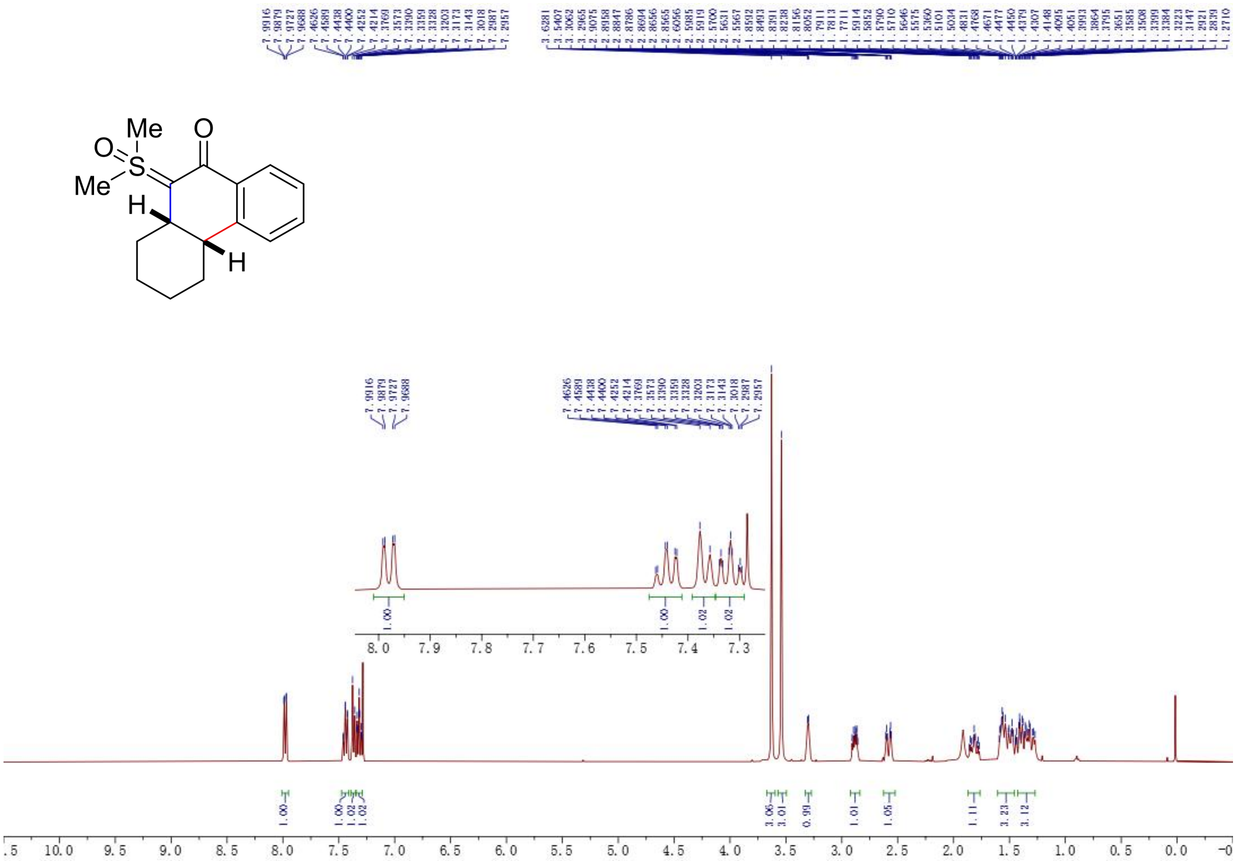

Supplementary Figure 131. ^{1}**H NMR** of the **5u** (400 MHz, 25 °C in $CDCl_3$)

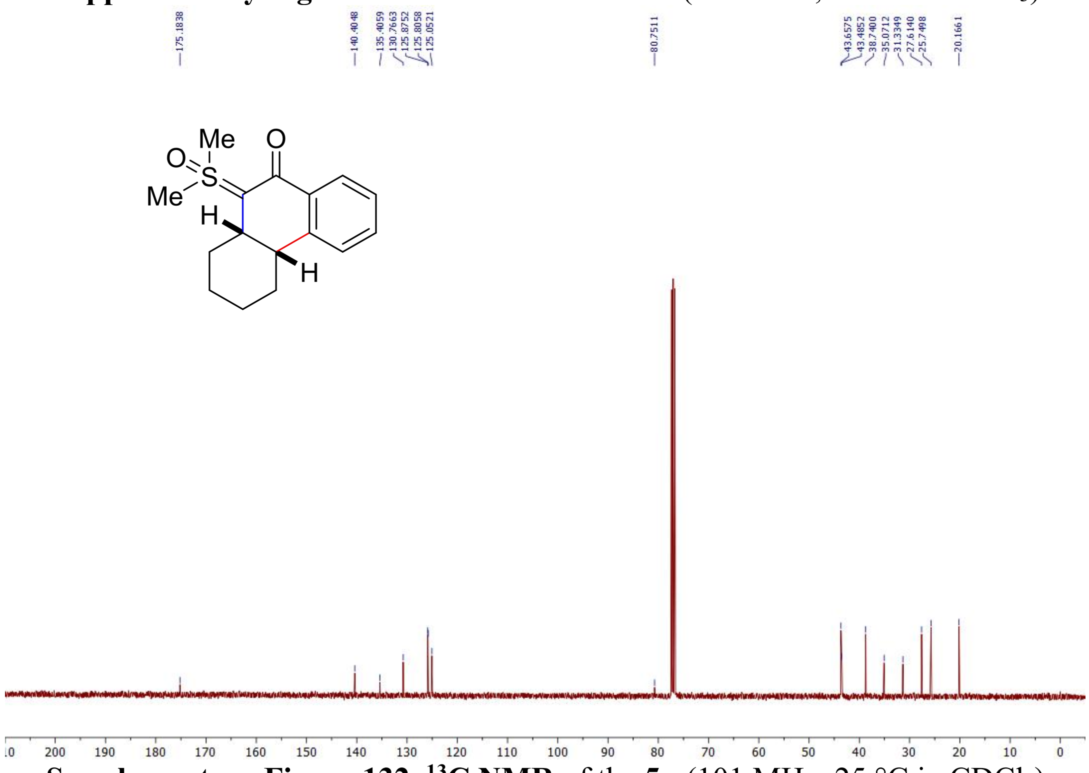

Supplementary Figure 132. ^{13}**C NMR** of the **5u** (101 MHz, 25 °C in $CDCl_3$)

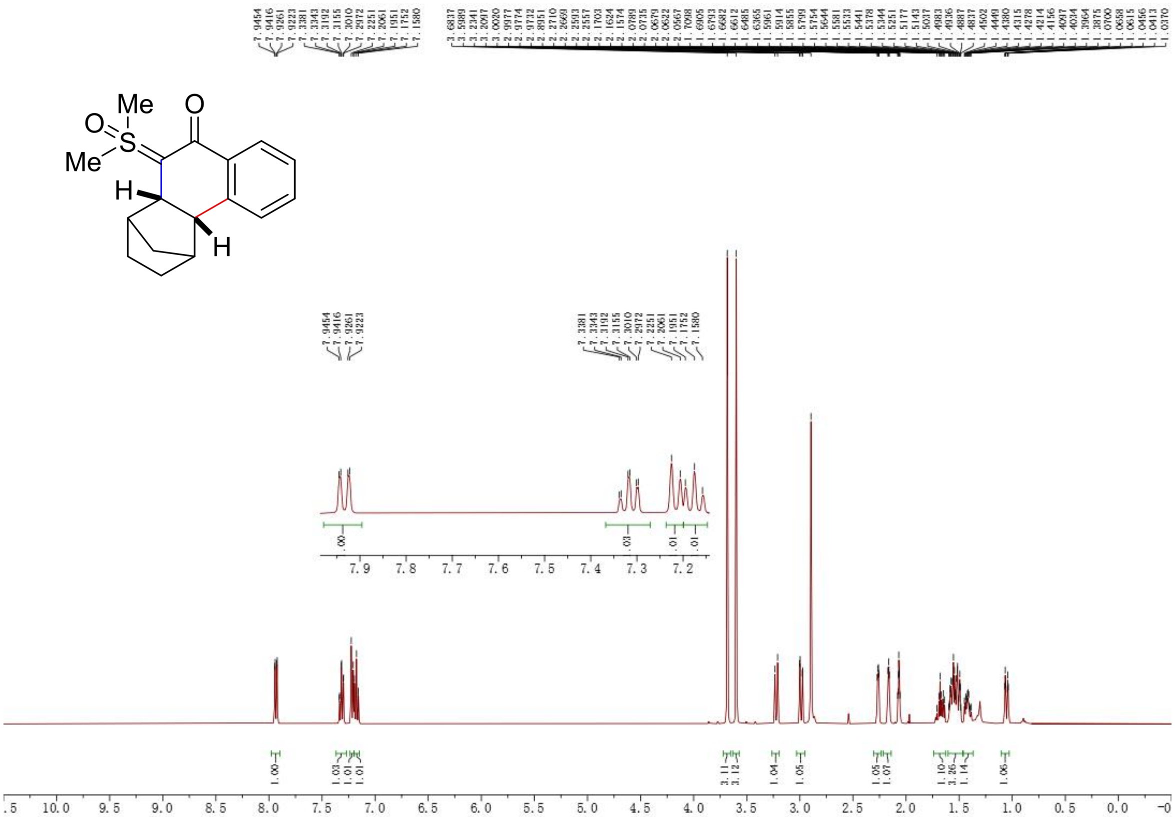

Supplementary Figure 133. ^{1}**H NMR** of the **5v** (400 MHz, 25 °C in Acetone-d_6)

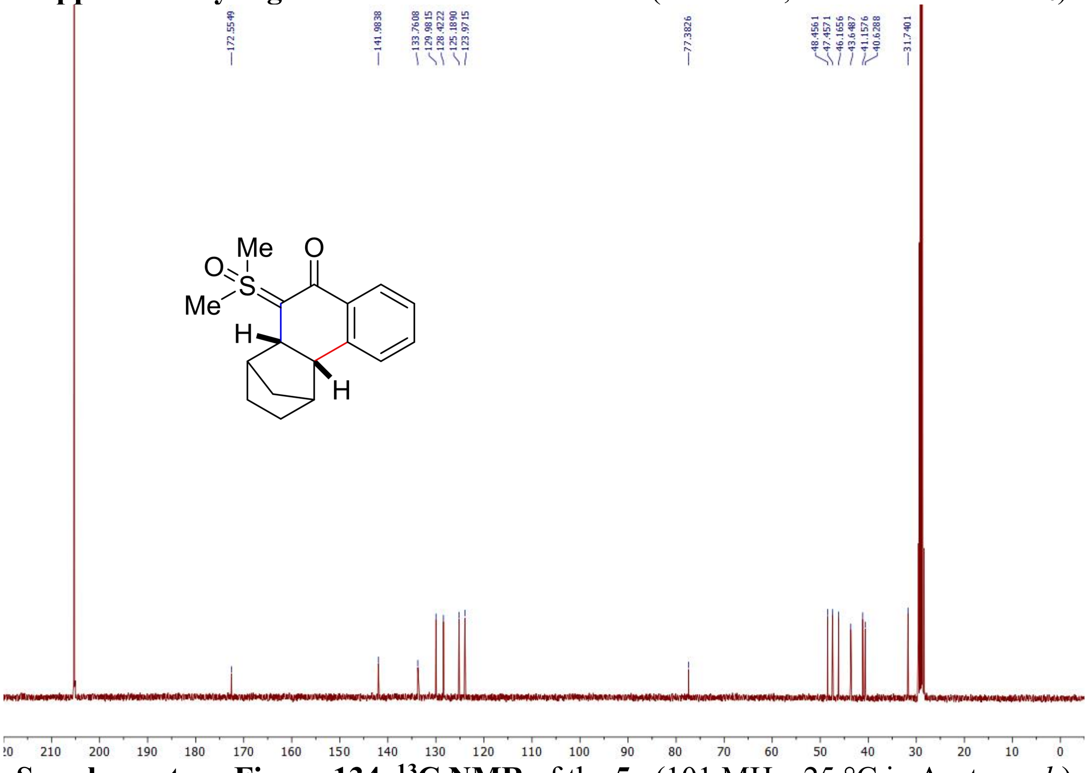

Supplementary Figure 134. ^{13}**C NMR** of the **5v** (101 MHz, 25 °C in Acetone-d_6)

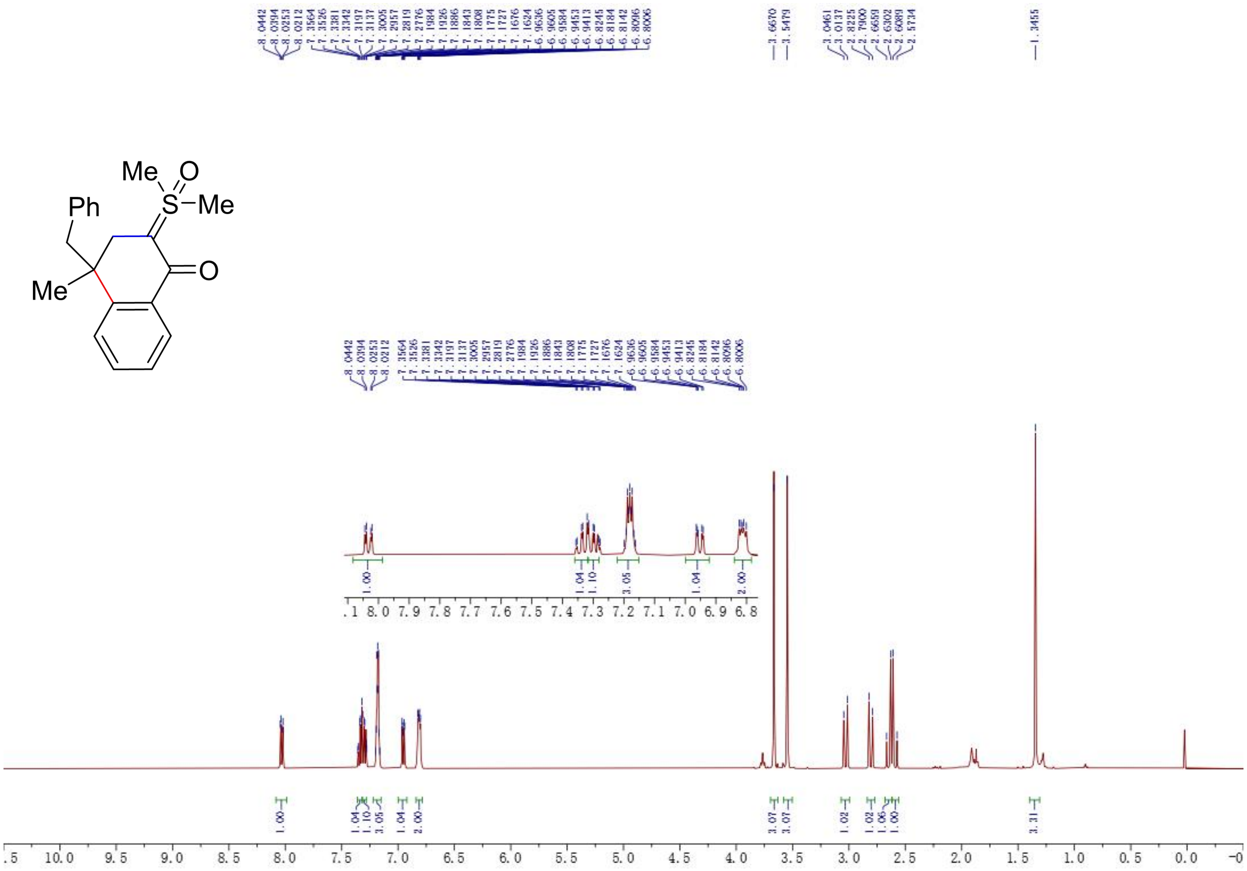

Supplementary Figure 135. ^{1}H NMR of the **5w** (400 MHz, 25 °C in $CDCl_3$)

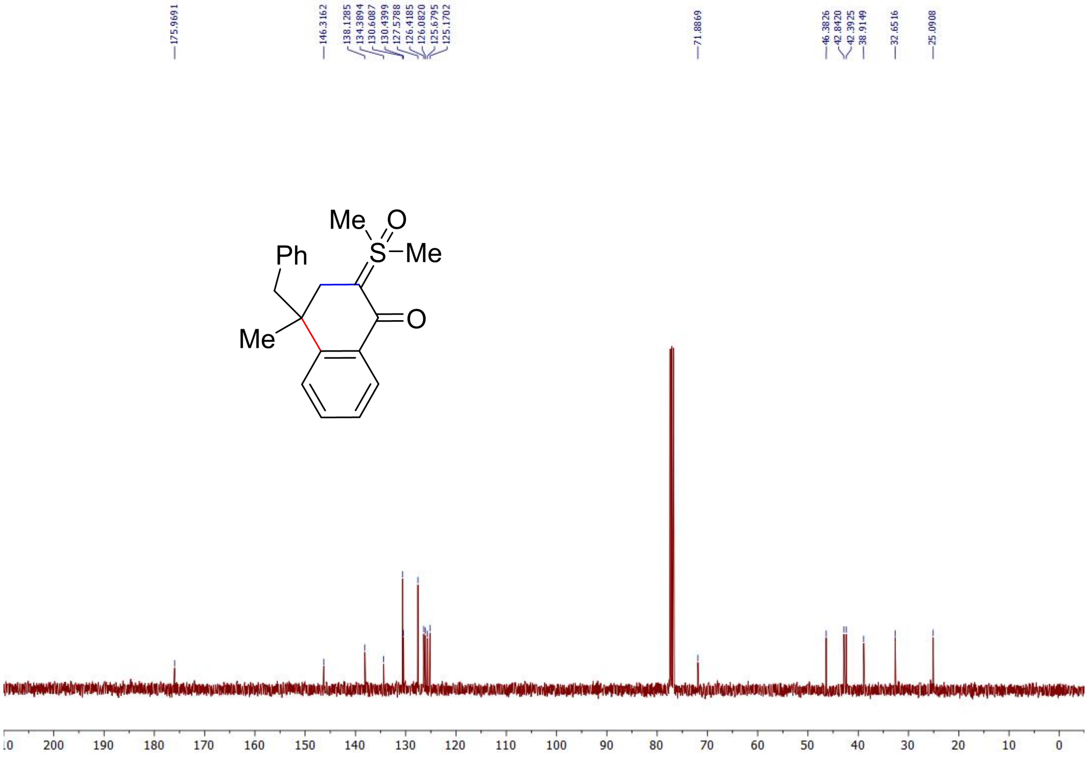

Supplementary Figure 136. ^{13}C NMR of the **5w** (101 MHz, 25 °C in $CDCl_3$)

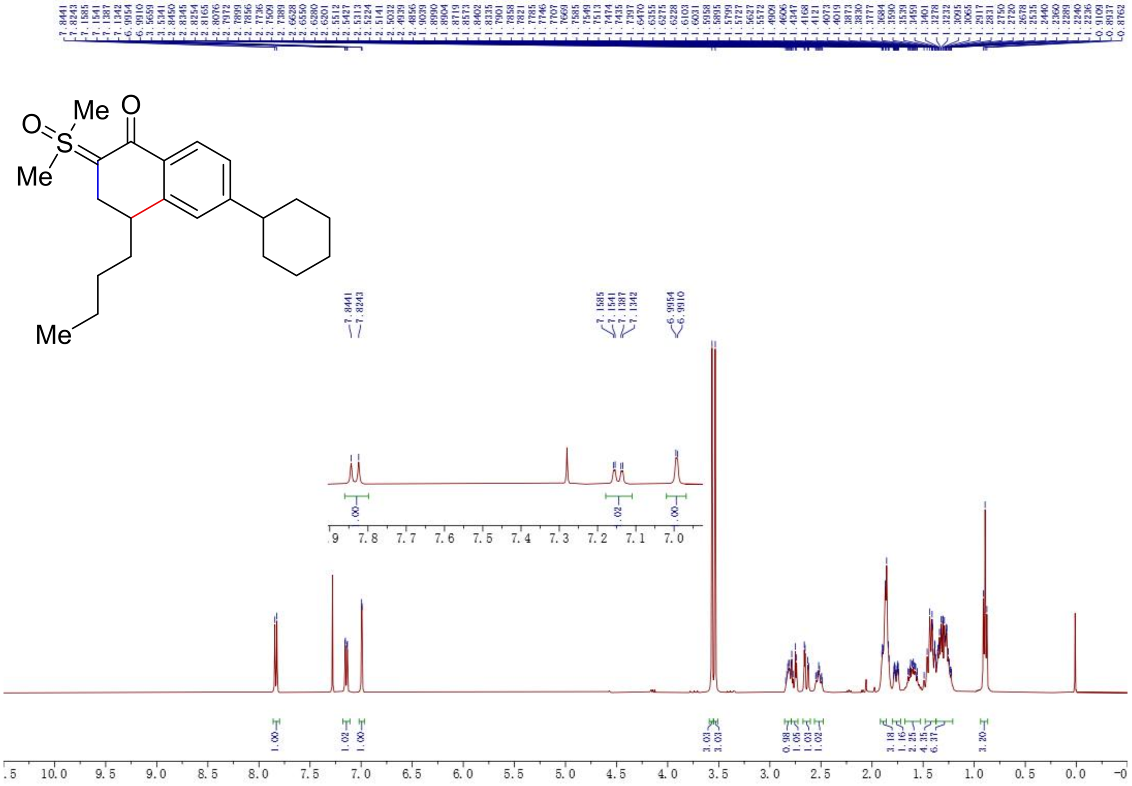

Supplementary Figure 137. ^{1}H NMR of the **5x** (400 MHz, 25 °C in $CDCl_3$)

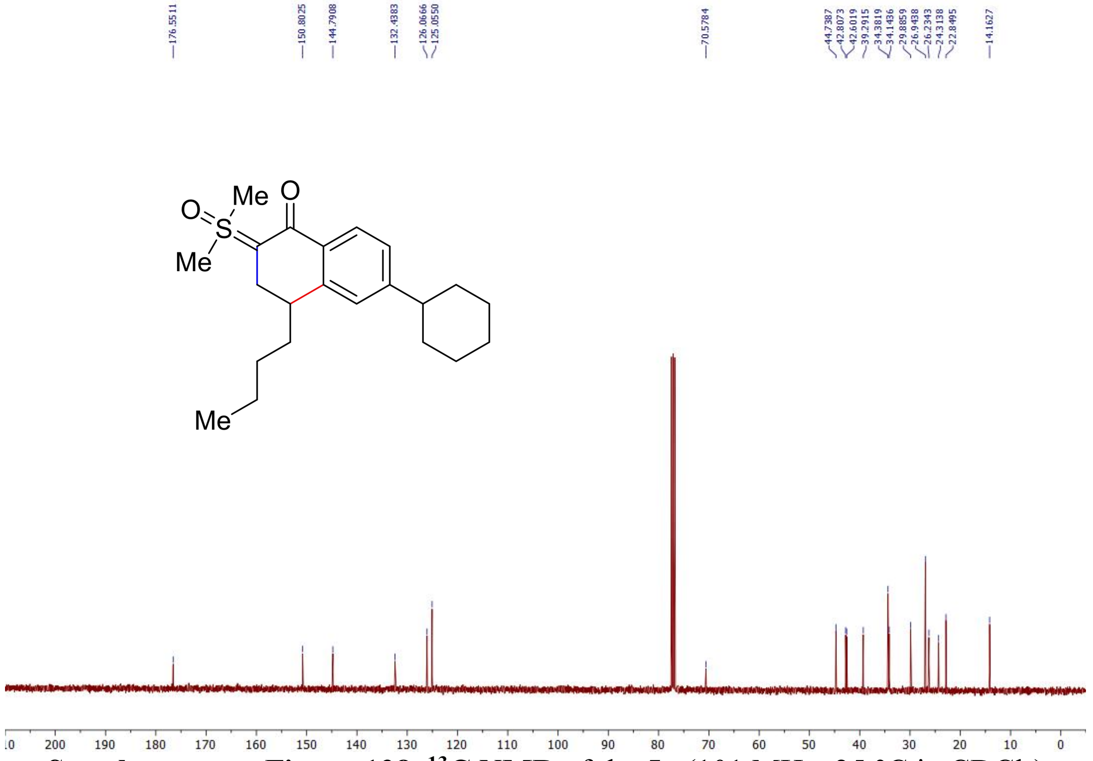

Supplementary Figure 138. ^{13}C NMR of the **5x** (101 MHz, 25 °C in $CDCl_3$)

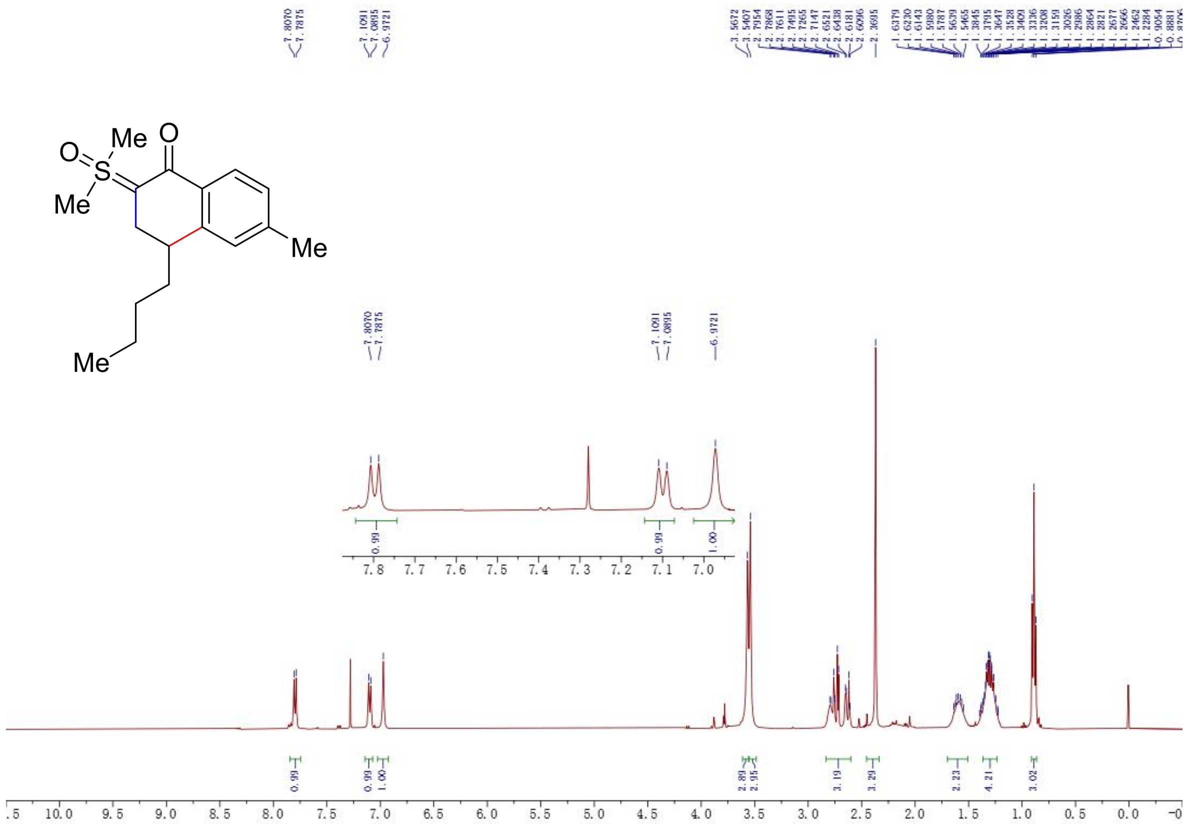

Supplementary Figure 139. ^{1}H NMR of the **5y** (400 MHz, 25 °C in $CDCl_3$)

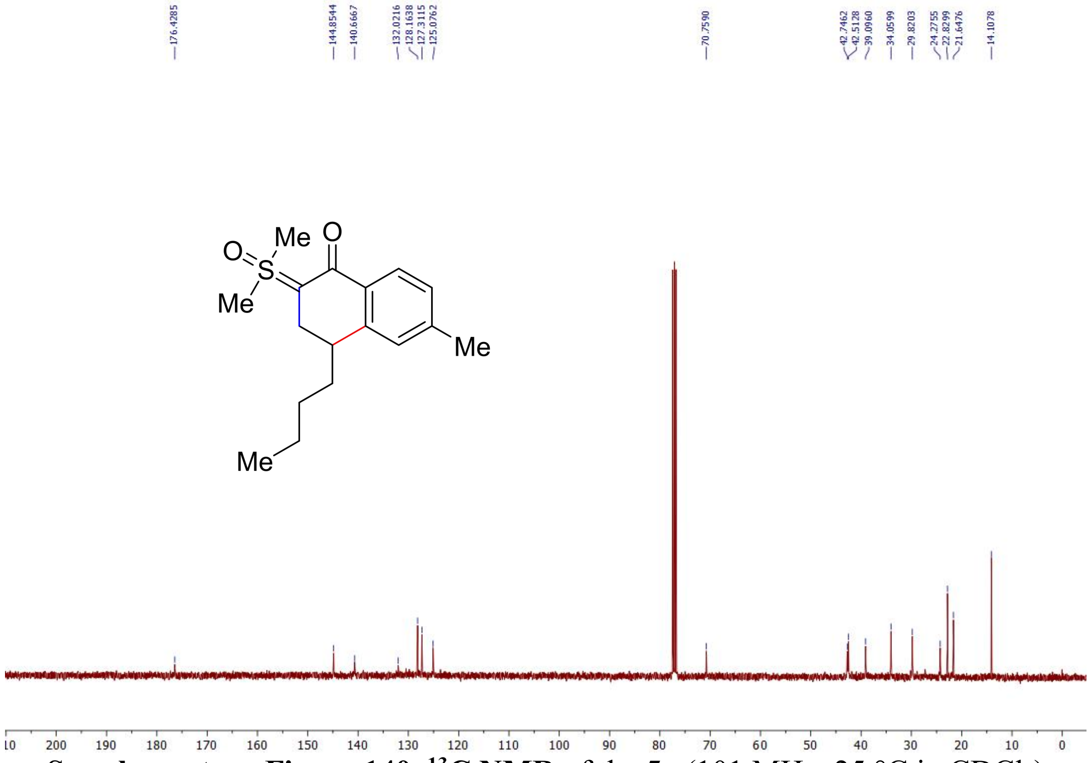

Supplementary Figure 140. ^{13}C NMR of the **5y** (101 MHz, 25 °C in $CDCl_3$)

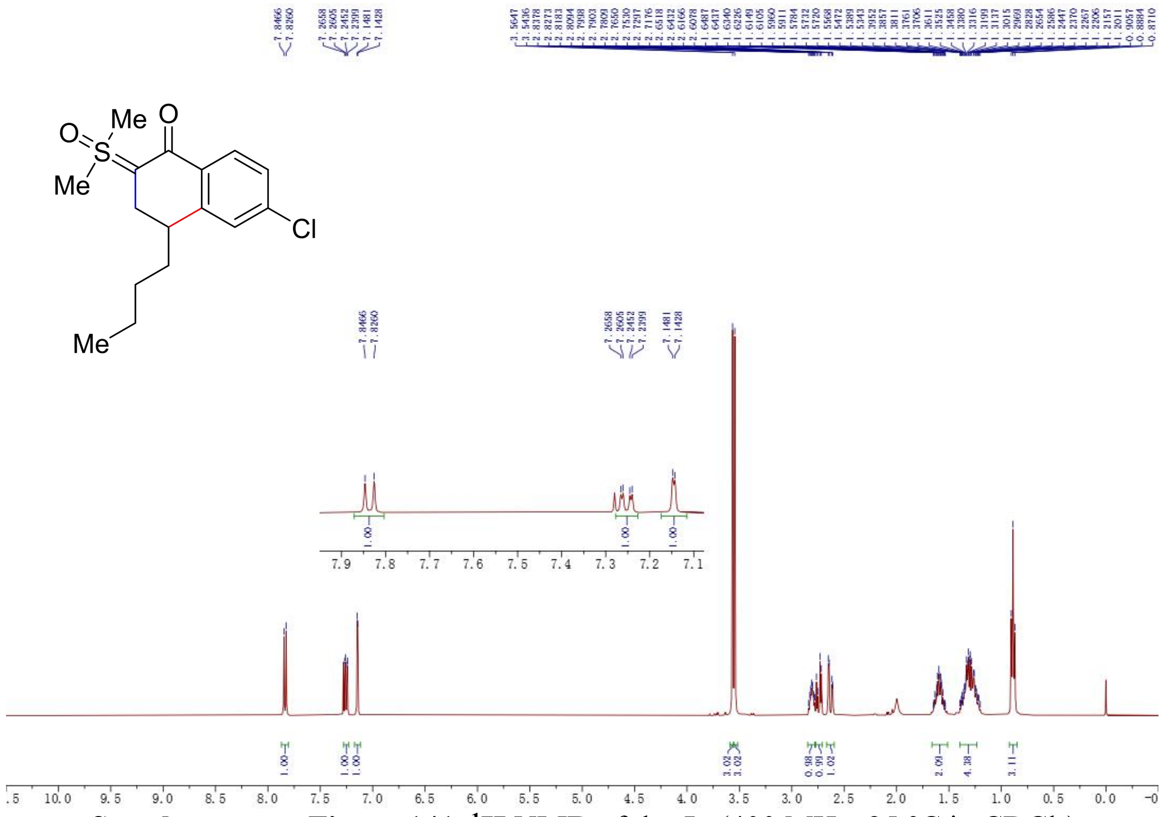

Supplementary Figure 141. ^{1}H NMR of the **5z** (400 MHz, 25 °C in $CDCl_3$)

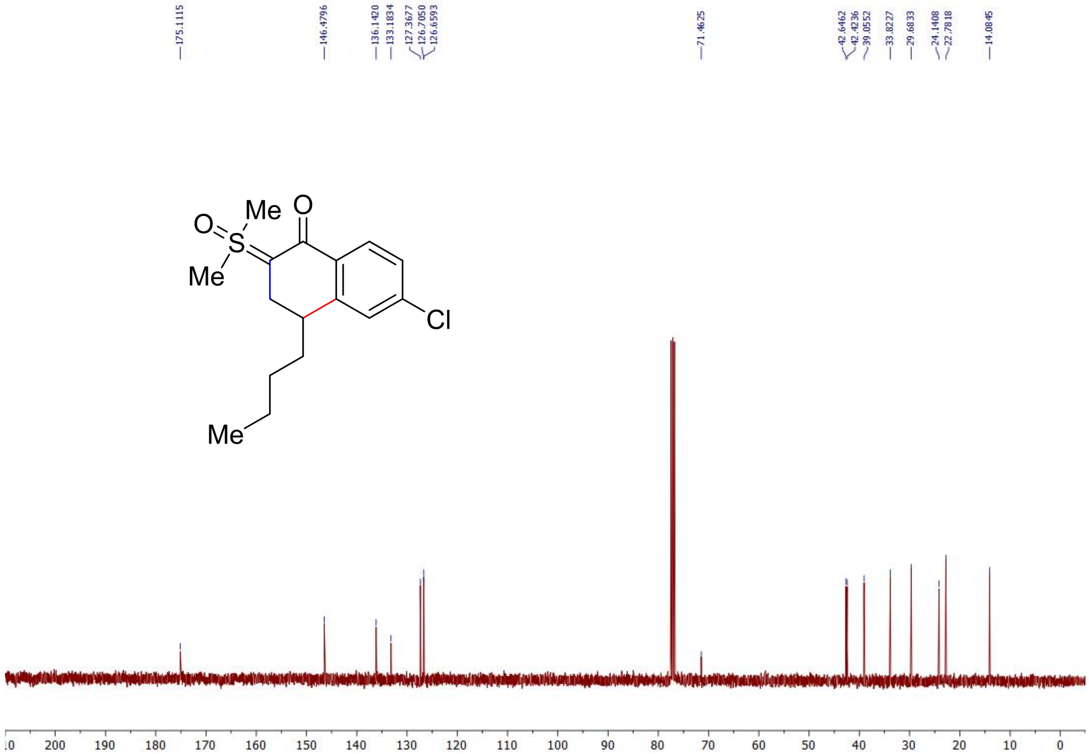

Supplementary Figure 142. ^{13}C NMR of the **5z** (101 MHz, 25 °C in $CDCl_3$)

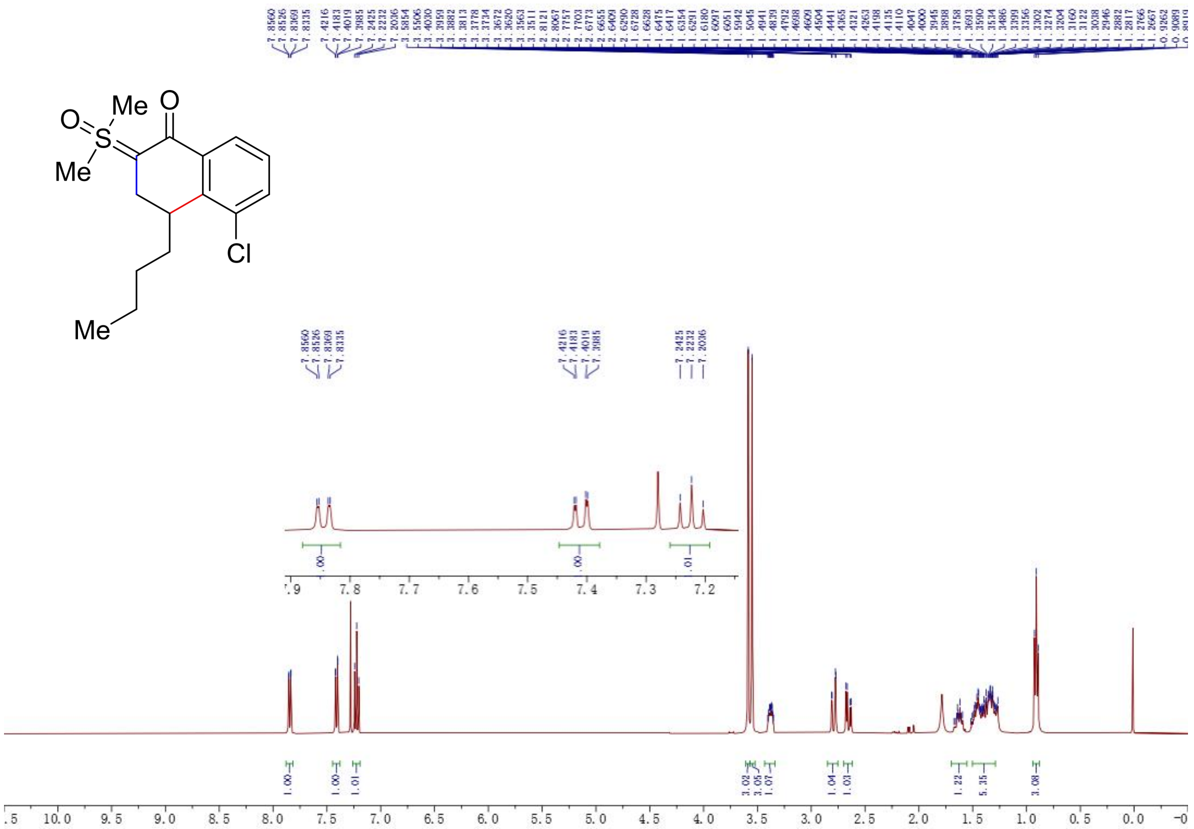

Supplementary Figure 143. ^{1}H NMR of the **5aa** (400 MHz, 25 °C in $CDCl_3$)

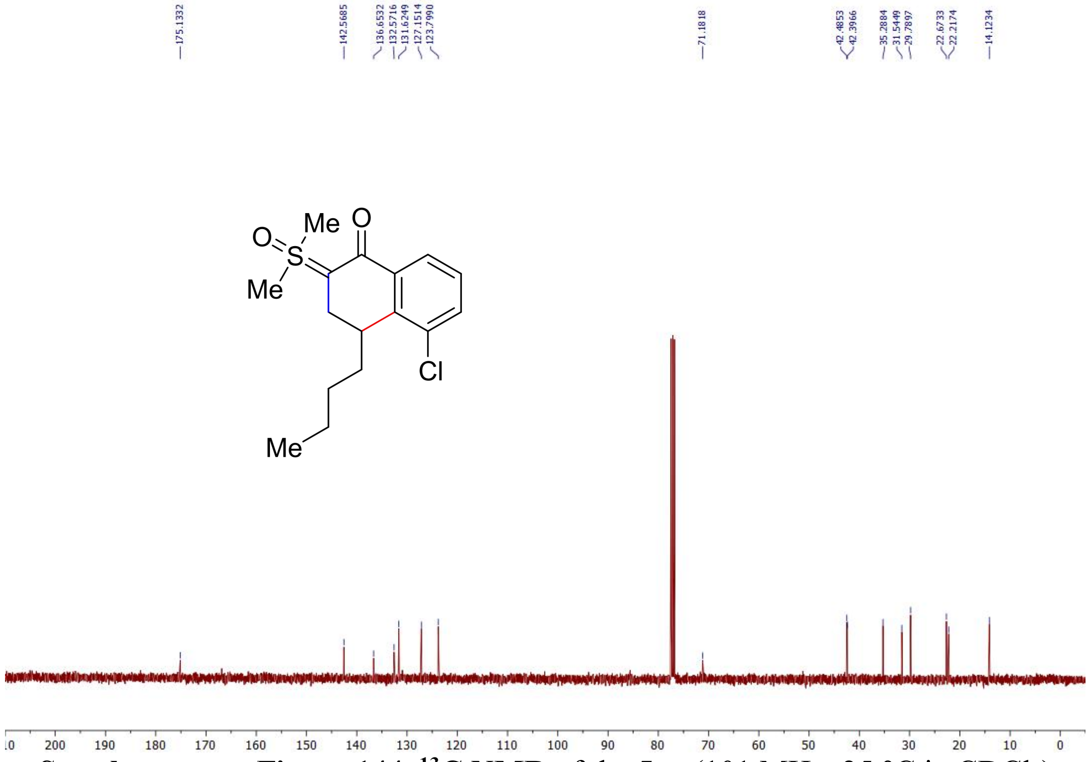

Supplementary Figure 144. ^{13}C NMR of the **5aa** (101 MHz, 25 °C in $CDCl_3$)

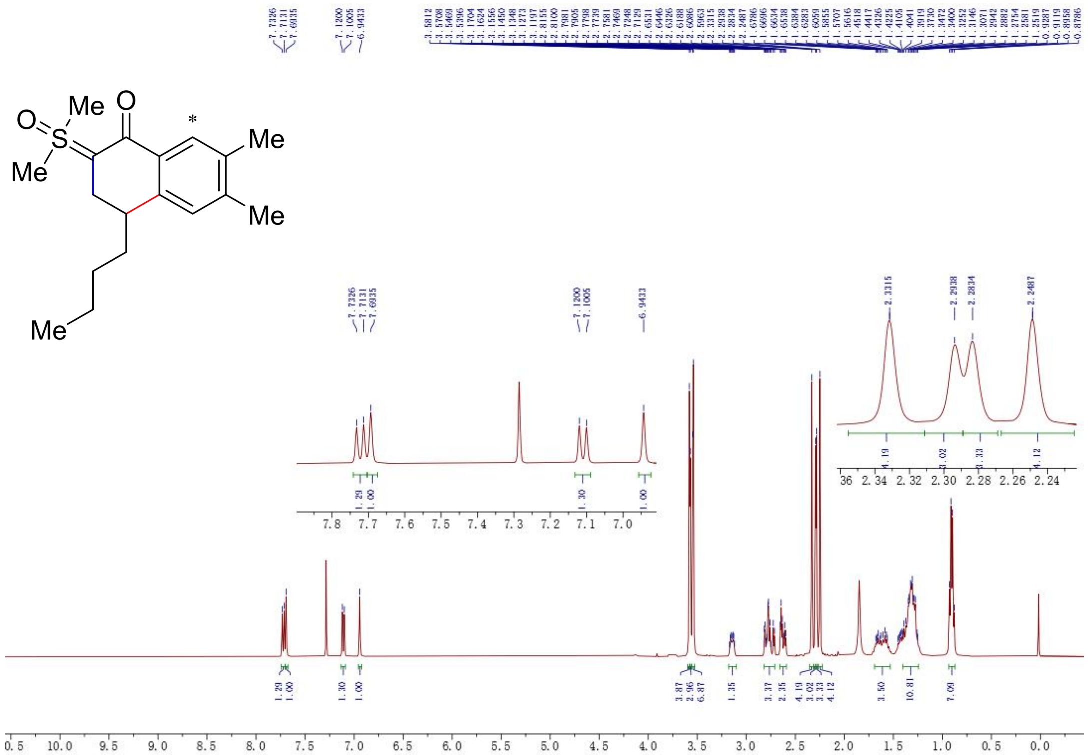

Supplementary Figure 145. **^{1}H NMR** of the **5ab** (400 MHz, 25 °C in $CDCl_3$)

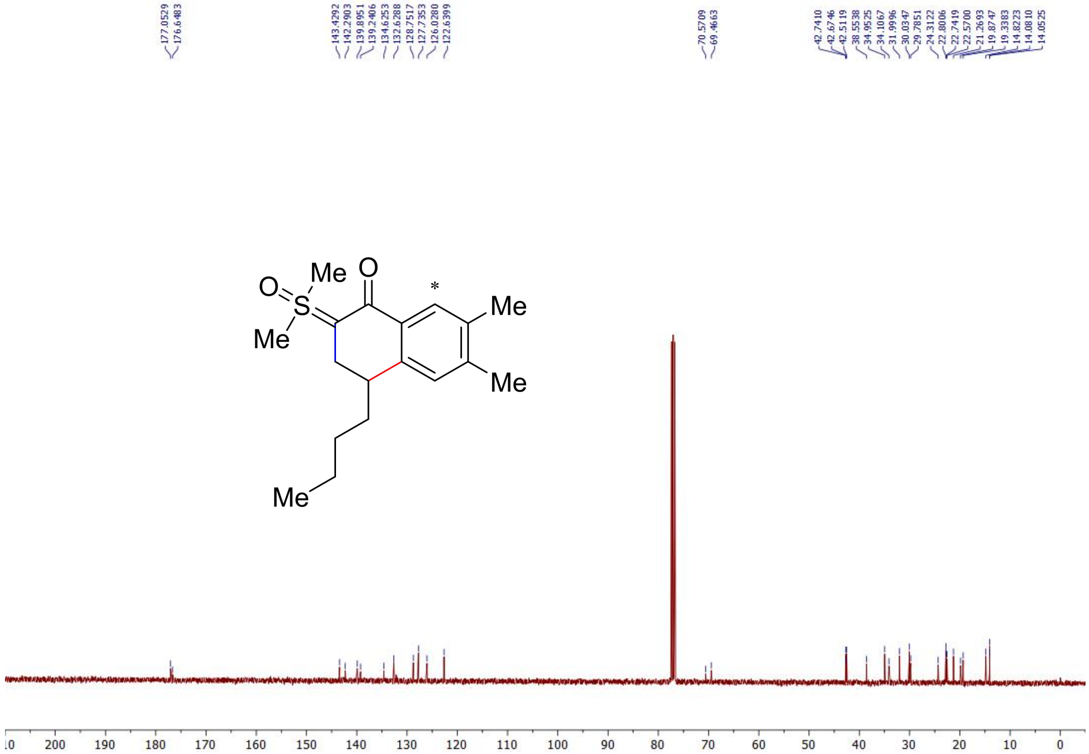

Supplementary Figure 146. **^{13}C NMR** of the **5ab** (101 MHz, 25 °C in $CDCl_3$)

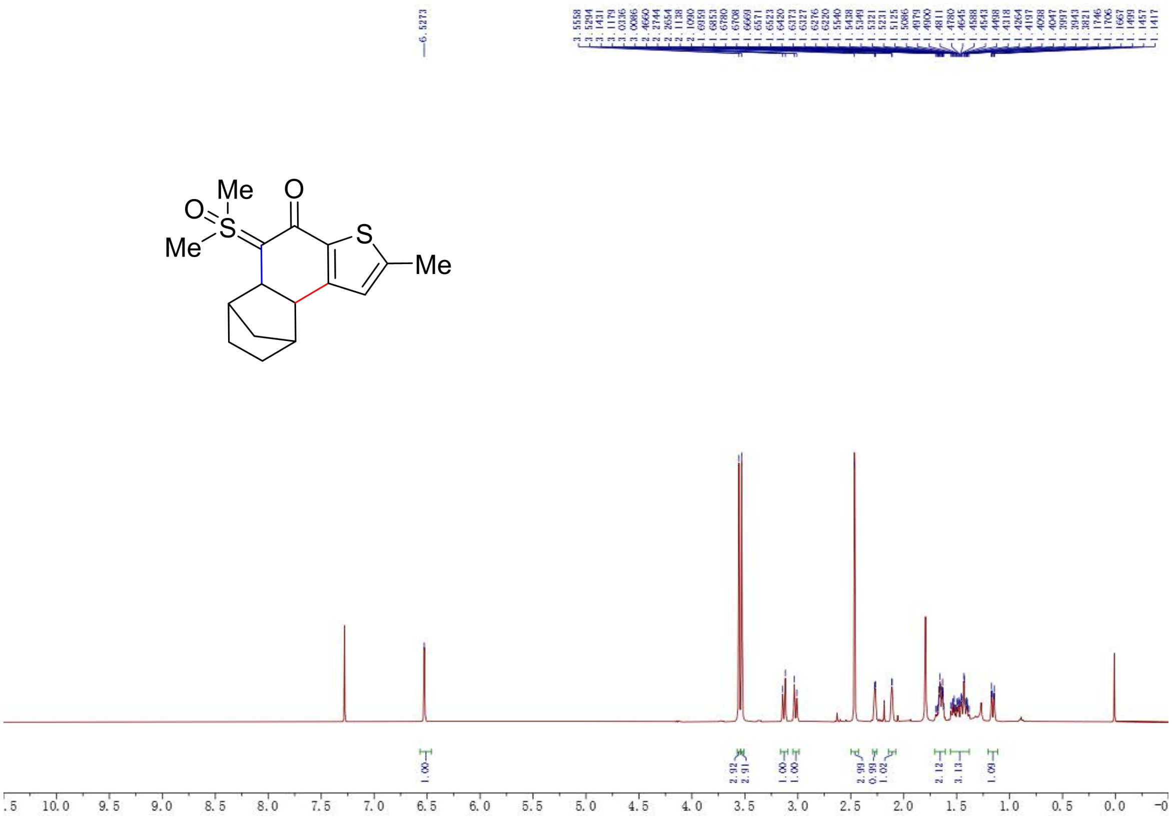

Supplementary Figure 147. ^{1}H NMR of the **5ac** (400 MHz, 25 °C in $CDCl_3$)

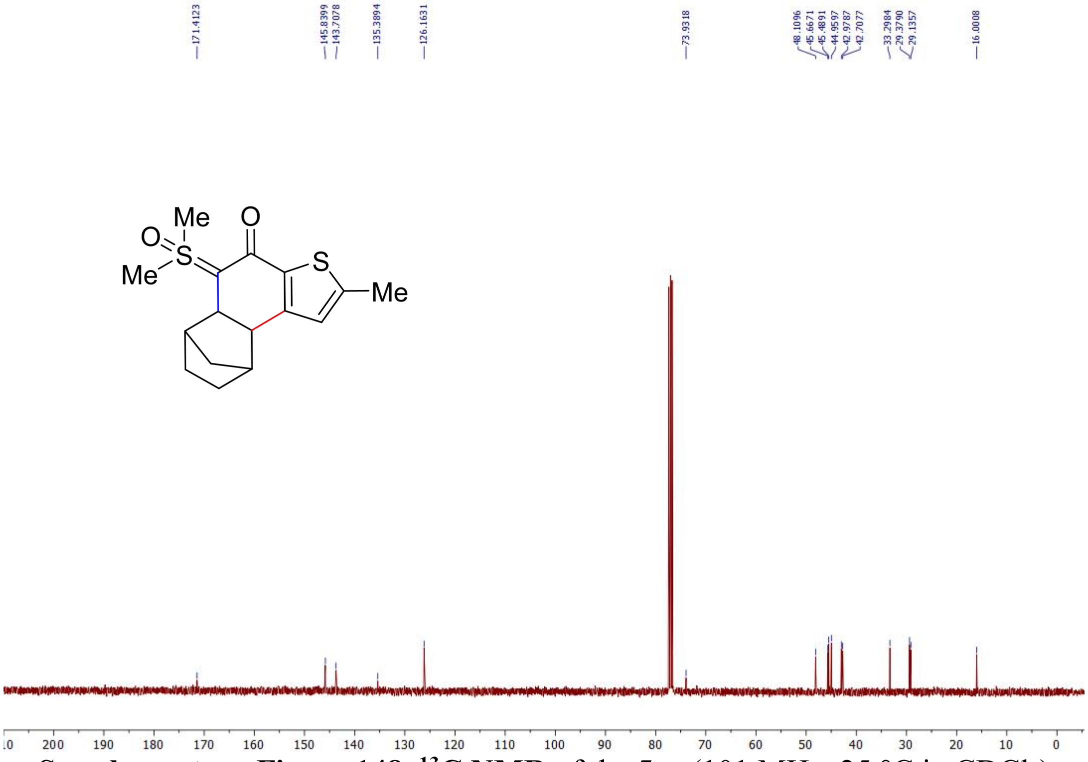

Supplementary Figure 148. ^{13}C NMR of the **5ac** (101 MHz, 25 °C in $CDCl_3$)

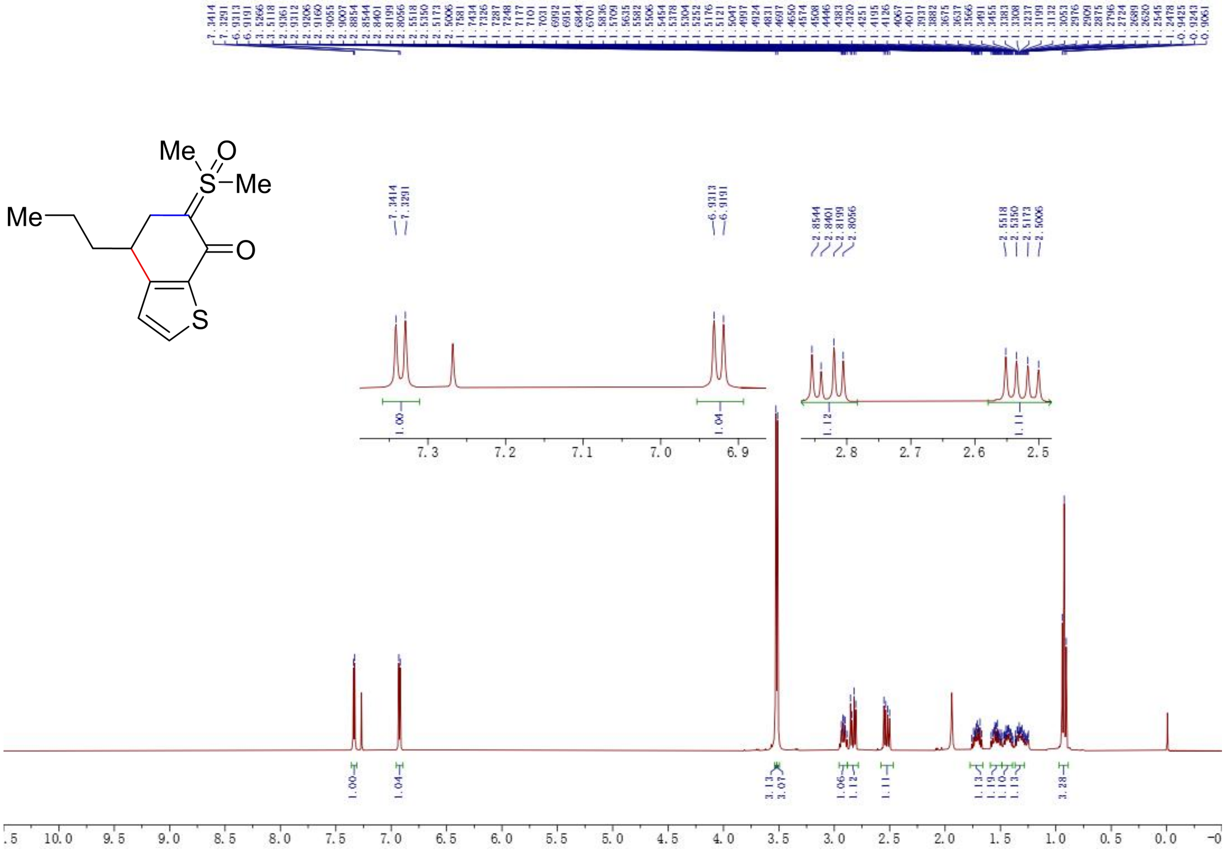

Supplementary Figure 149. ^{1}H NMR of the **5ad** (400 MHz, 25 °C in $CDCl_3$)

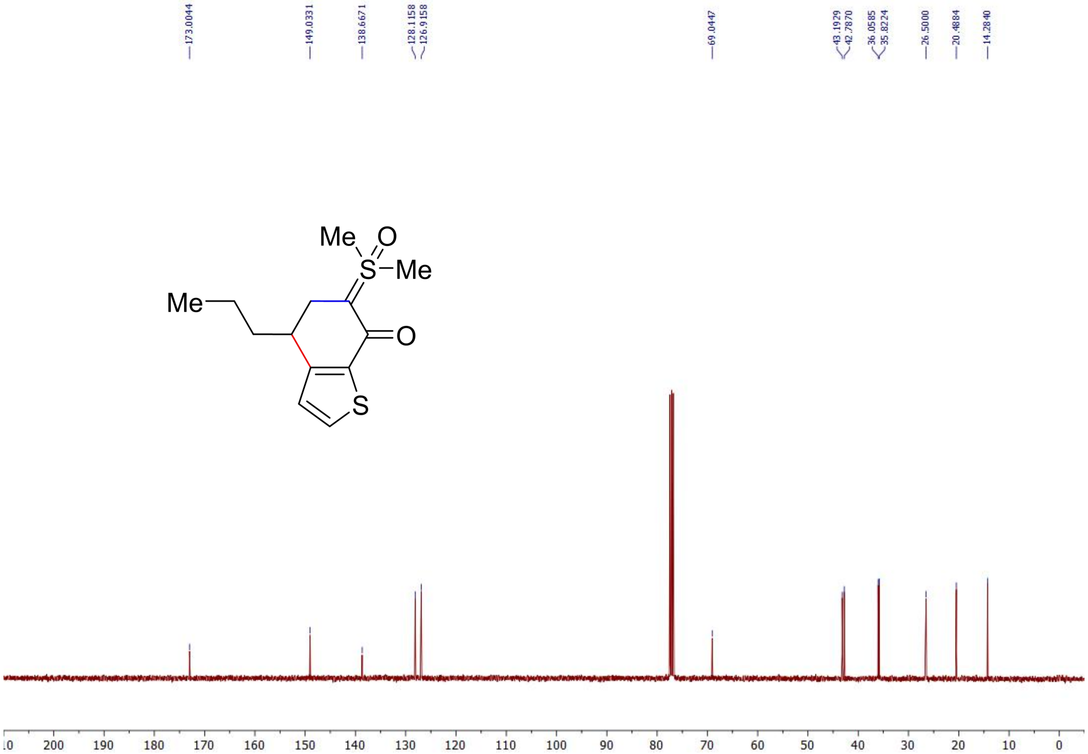

Supplementary Figure 150. ^{13}C NMR of the **5ad** (101 MHz, 25 °C in $CDCl_3$)

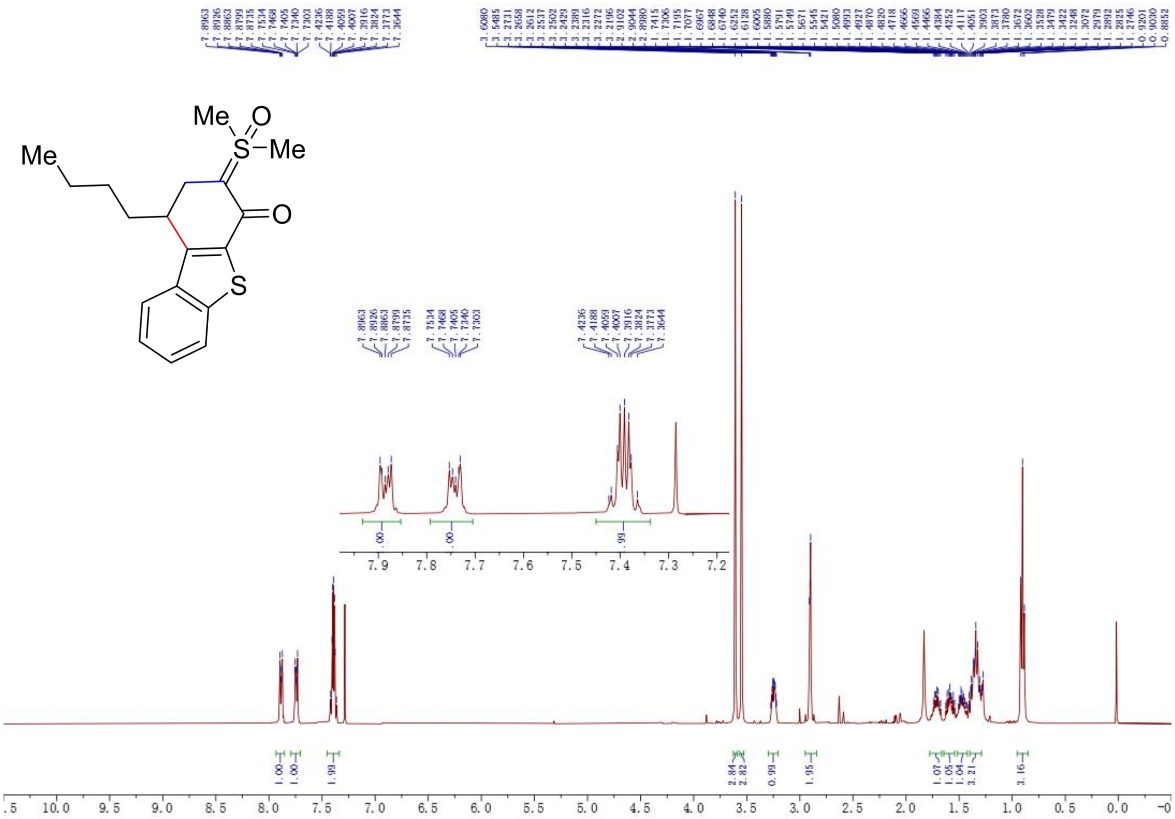

Supplementary Figure 151. ^{1}H NMR of the **5ae** (400 MHz, 25 °C in $CDCl_3$)

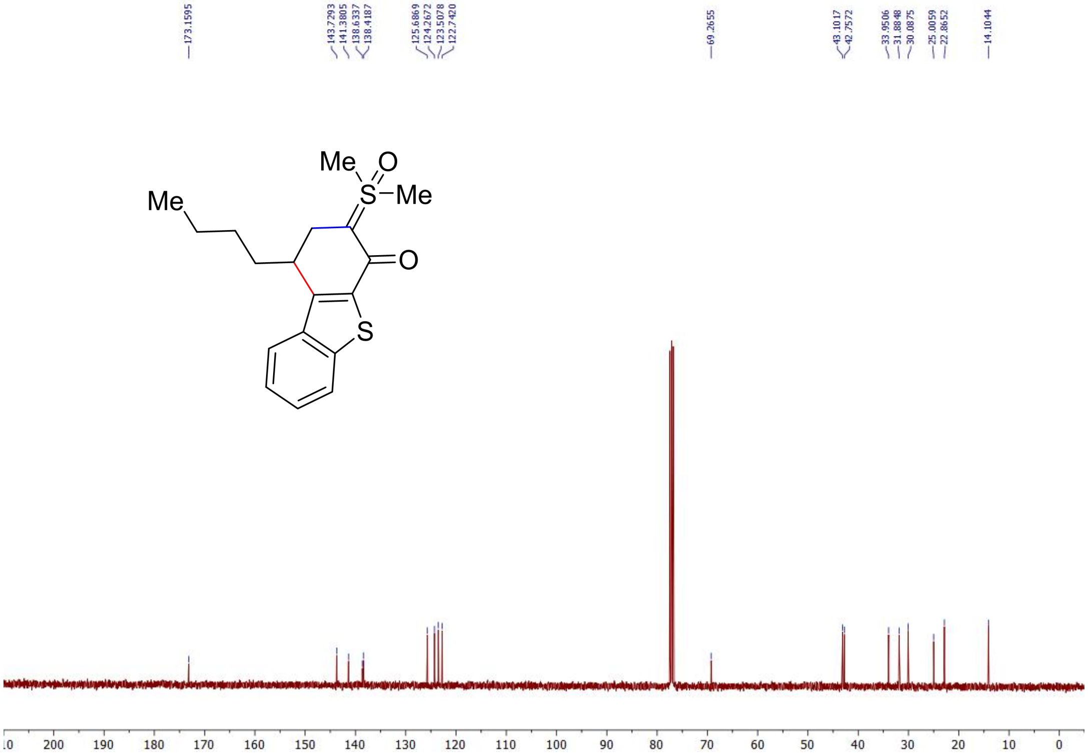

Supplementary Figure 152. ^{13}C NMR of the **5ae** (101 MHz, 25 °C in $CDCl_3$)

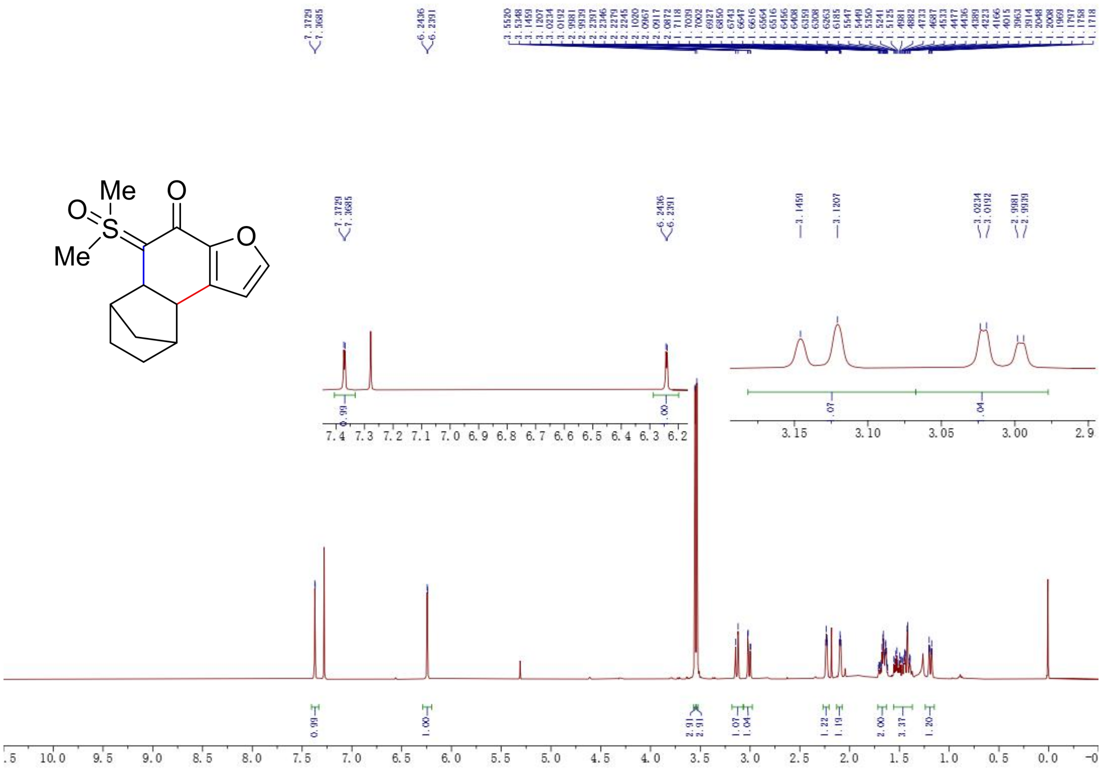

Supplementary Figure 153. ¹H NMR of the **5af** (400 MHz, 25 °C in $CDCl_3$)

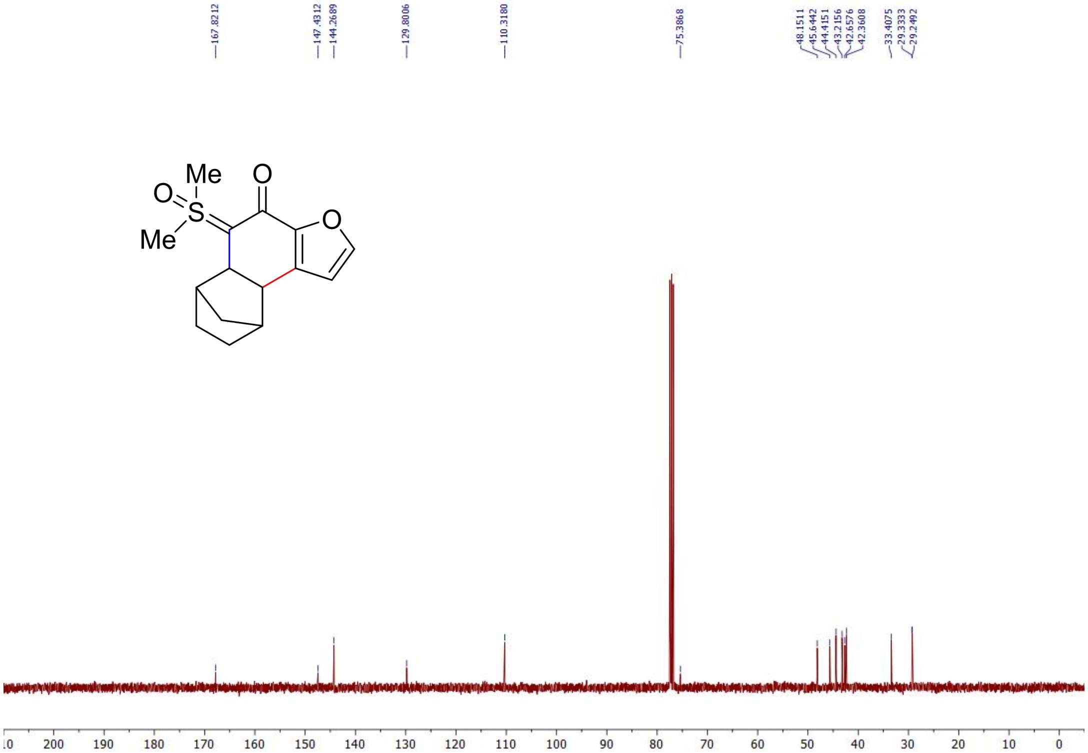

Supplementary Figure 154. ¹³C NMR of the **5af** (101 MHz, 25 °C in $CDCl_3$)

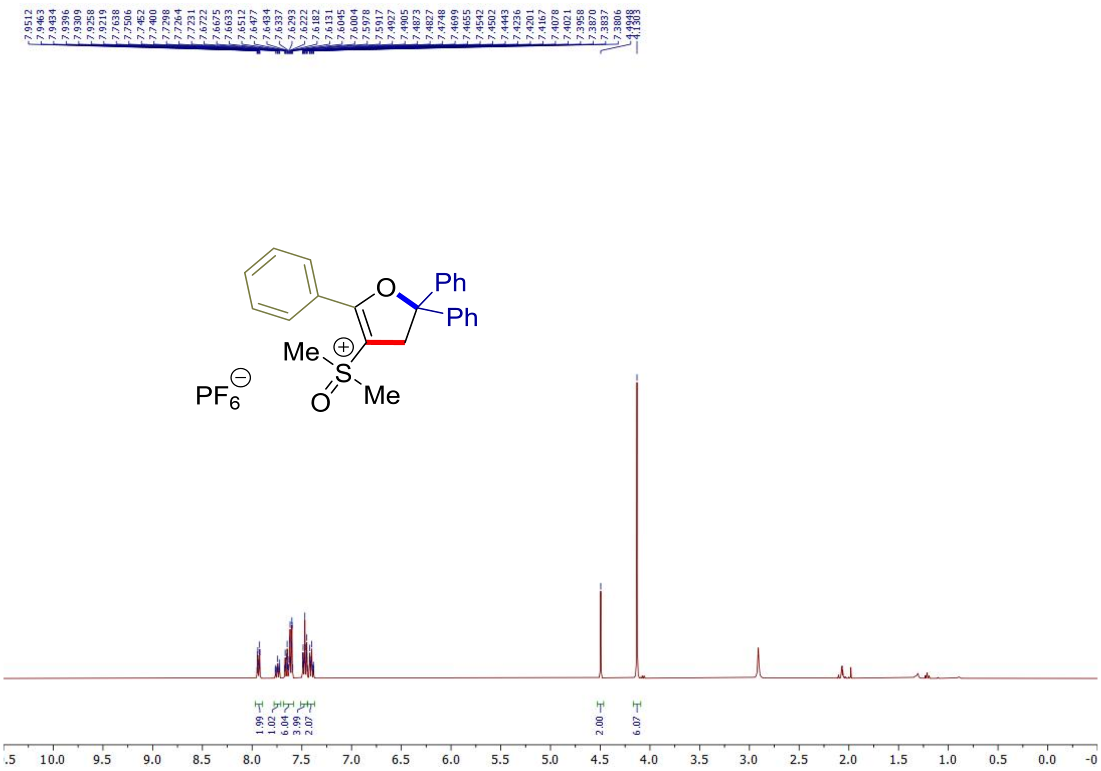

Supplementary Figure 155. ^{1}**H NMR** of the **7a** (400 MHz, 25 °C in Acetone-d_6)

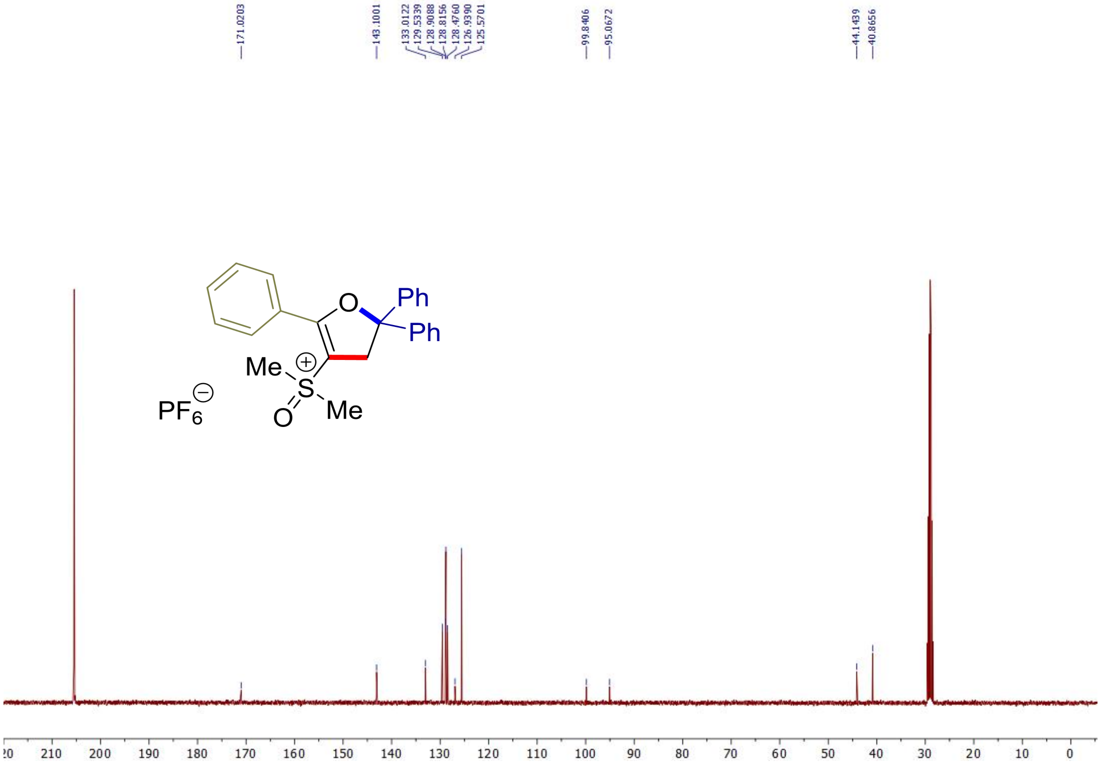

Supplementary Figure 156. ^{13}**C NMR** of the **7a** (101 MHz, 25 °C in Acetone-d_6)

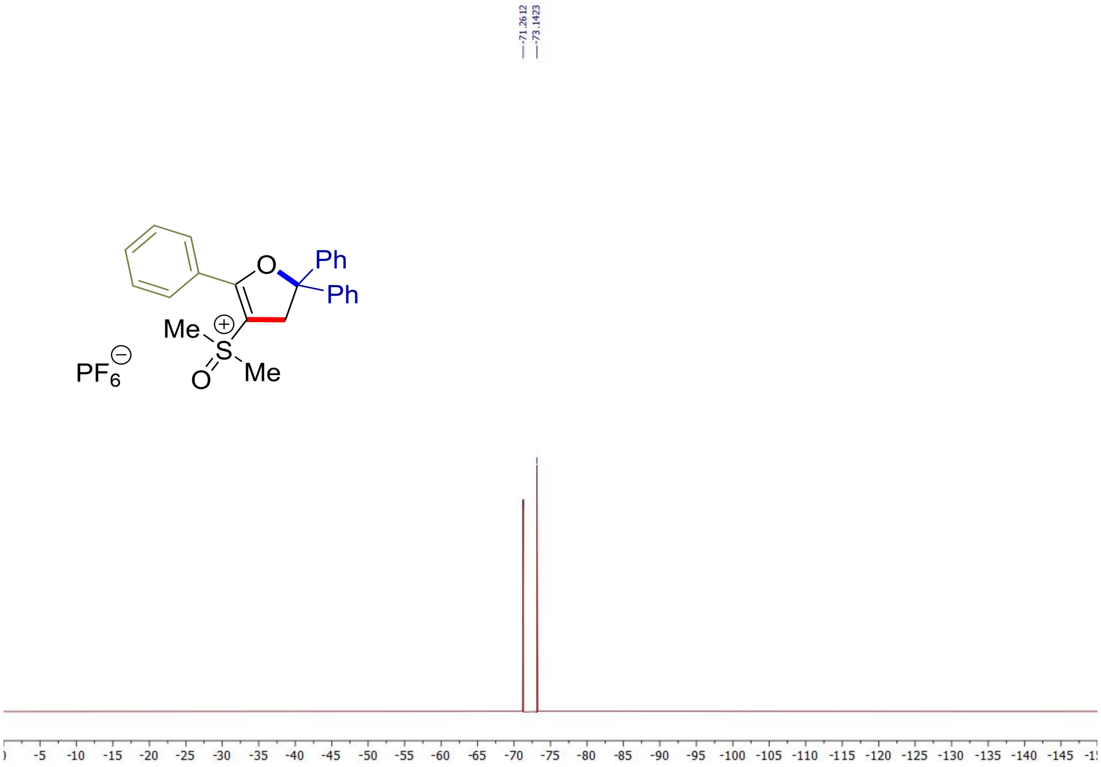

Supplementary Figure 157. ^{19}**F NMR** of the **7a** (376 MHz, 25 °C in Acetone-d_6)

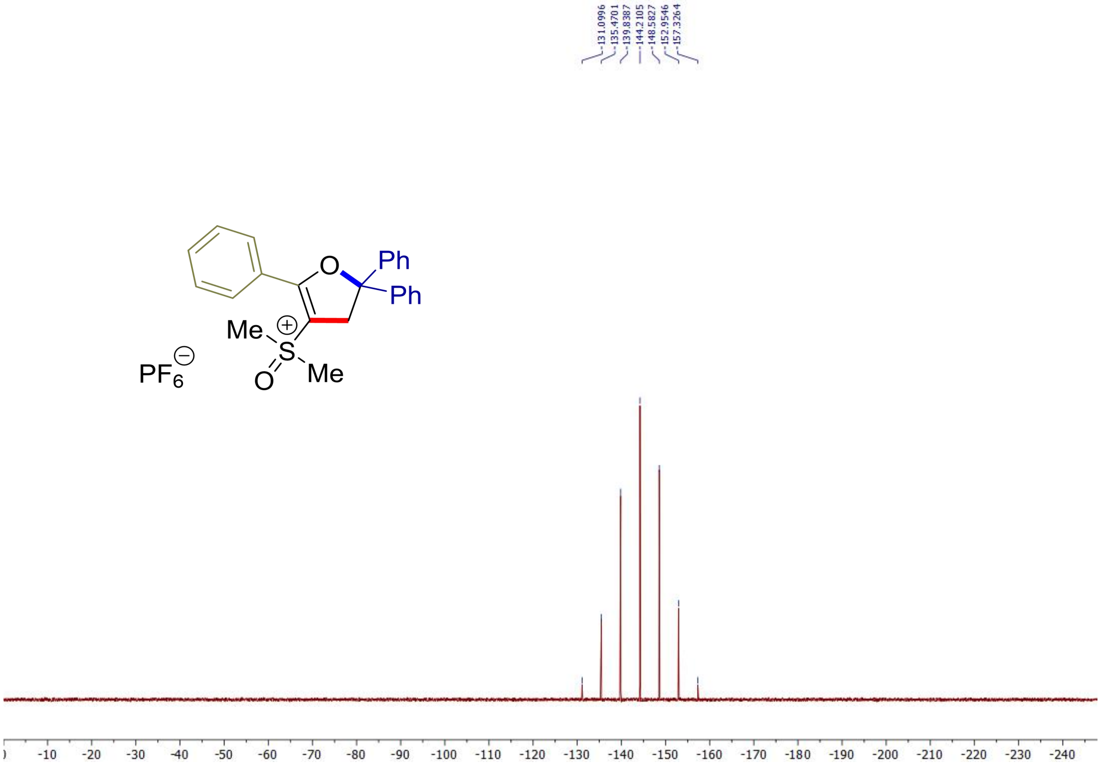

Supplementary Figure 158. ^{31}**P NMR** of the **7a** (162 MHz, 25 °C in Acetone-d_6)

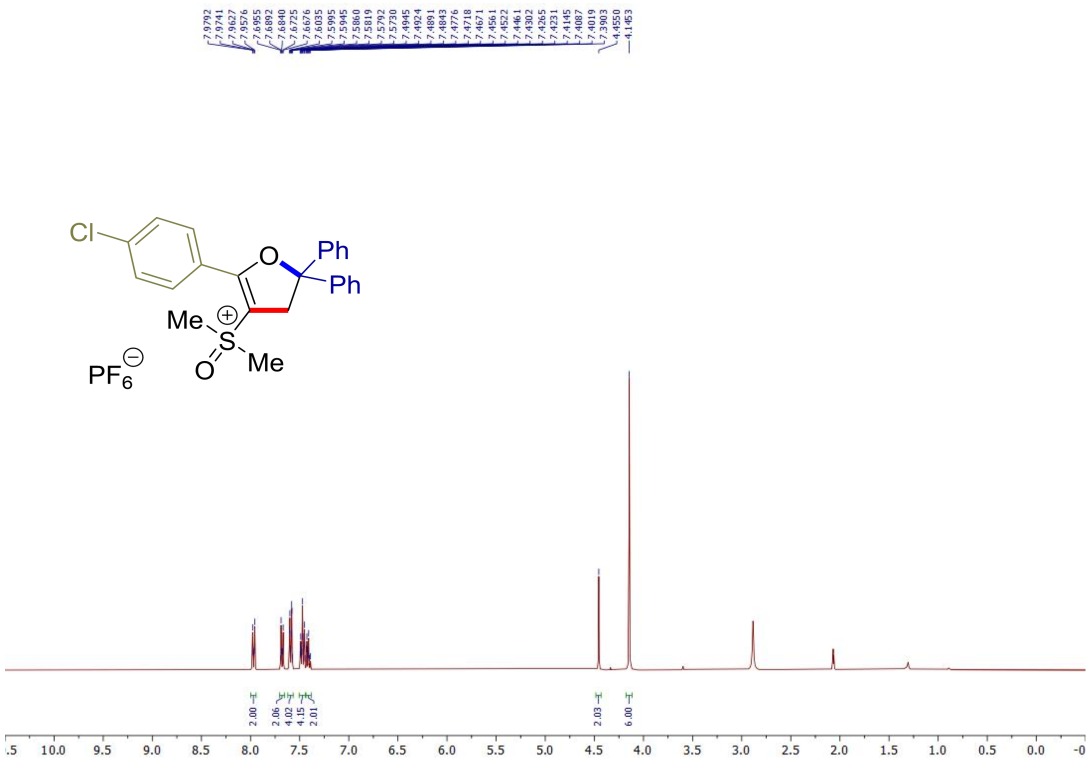

Supplementary Figure 159. ^{1}H NMR of the **7b** (400 MHz, 25 °C in Acetone-d_6)

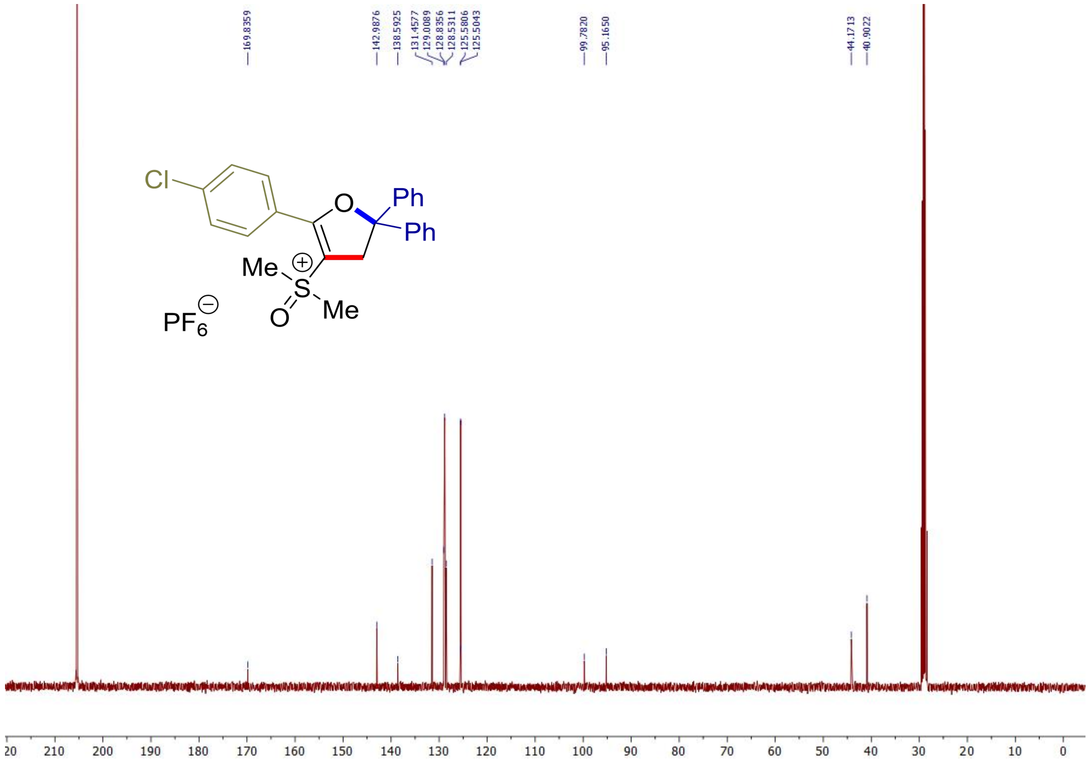

Supplementary Figure 160. ^{13}C NMR of the **7b** (101 MHz, 25 °C in Acetone-d_6)

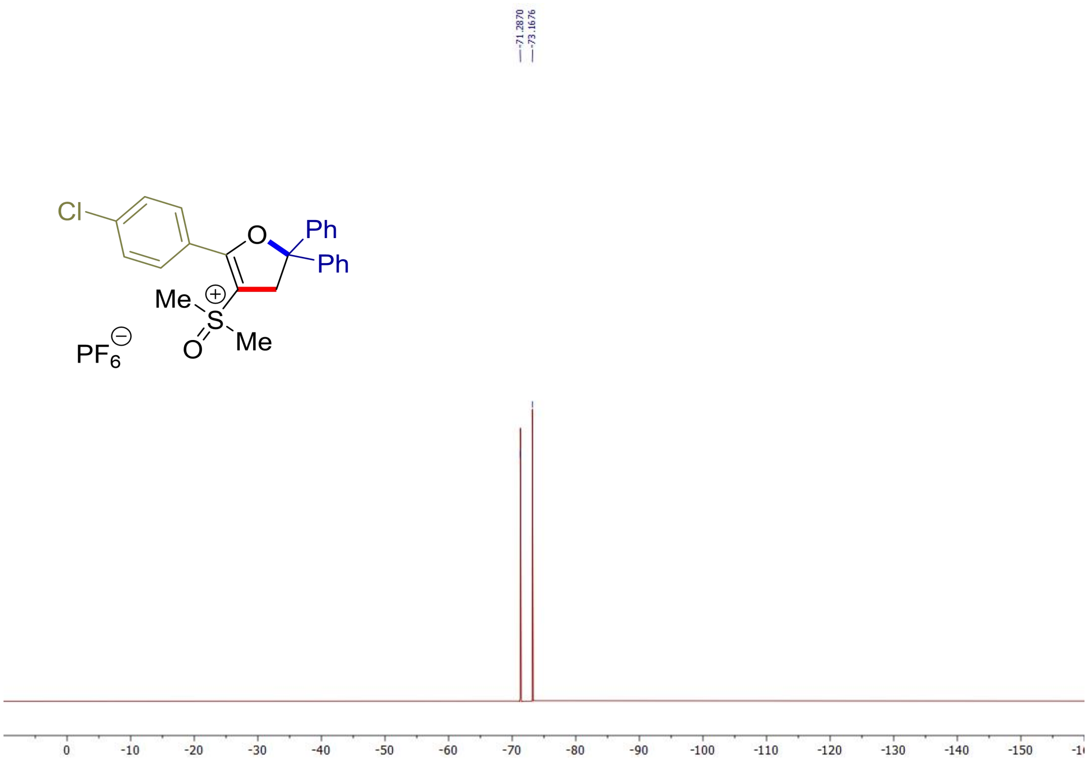

Supplementary Figure 161. ^{19}**F NMR** of the **7b** (376 MHz, 25 °C in Acetone-d_6)

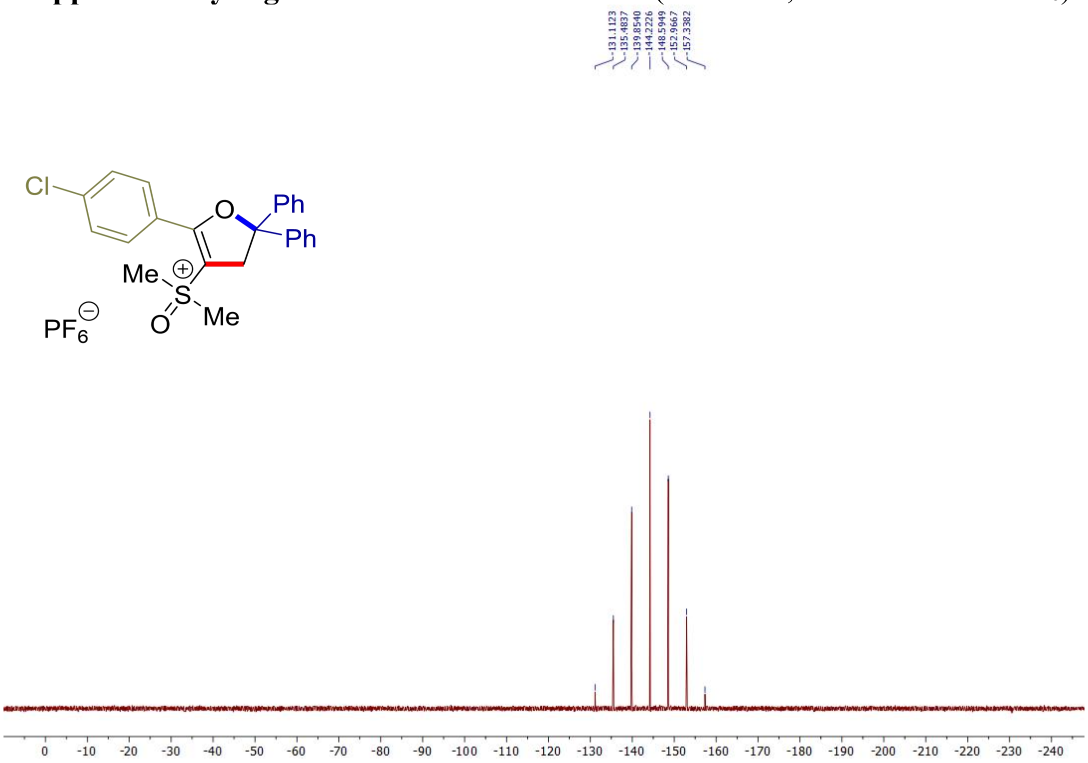

Supplementary Figure 162. ^{31}**P NMR** of the **7b** (162 MHz, 25 °C in Acetone-d_6)

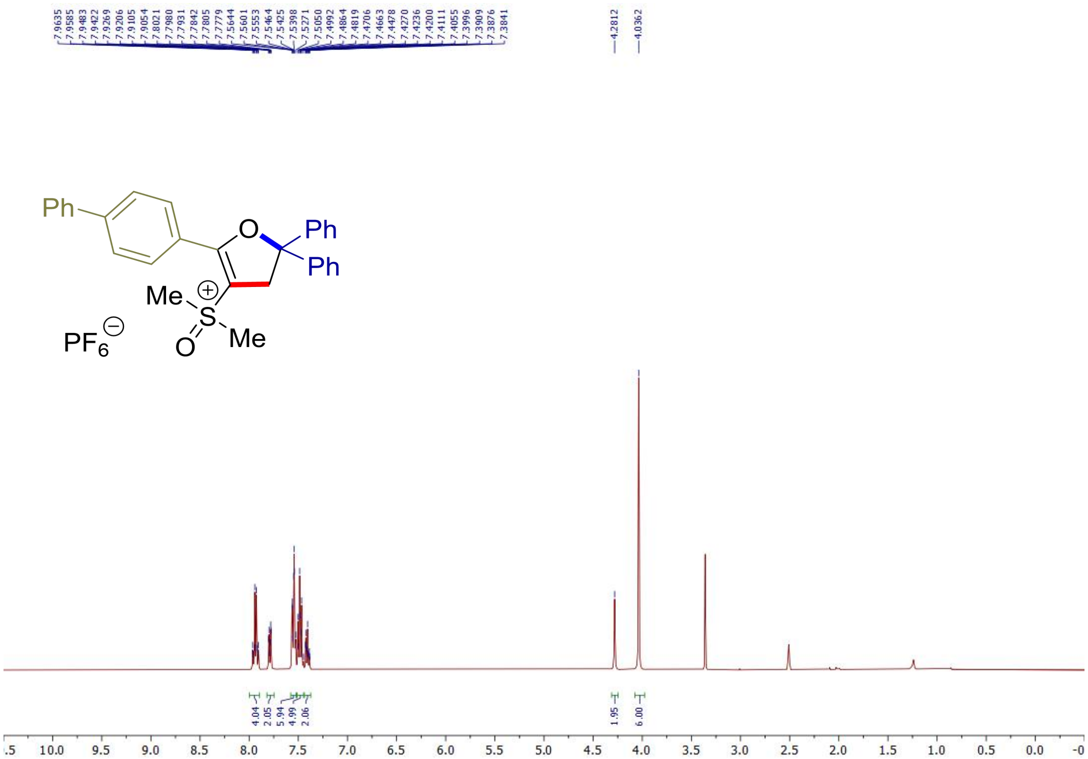

Supplementary Figure 163. **^{1}H NMR** of the **7c** (400 MHz, 25 °C in DMSO-d_6)

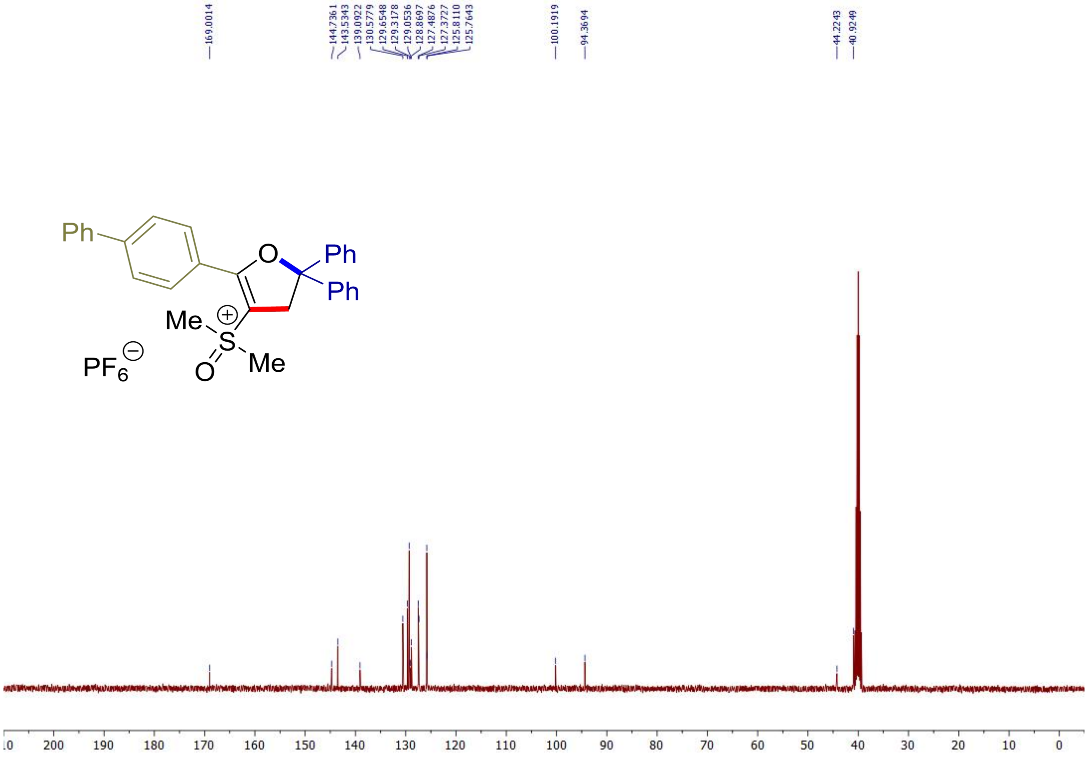

Supplementary Figure 164. **^{13}C NMR** of the **7c** (101 MHz, 25 °C in DMSO-d_6)

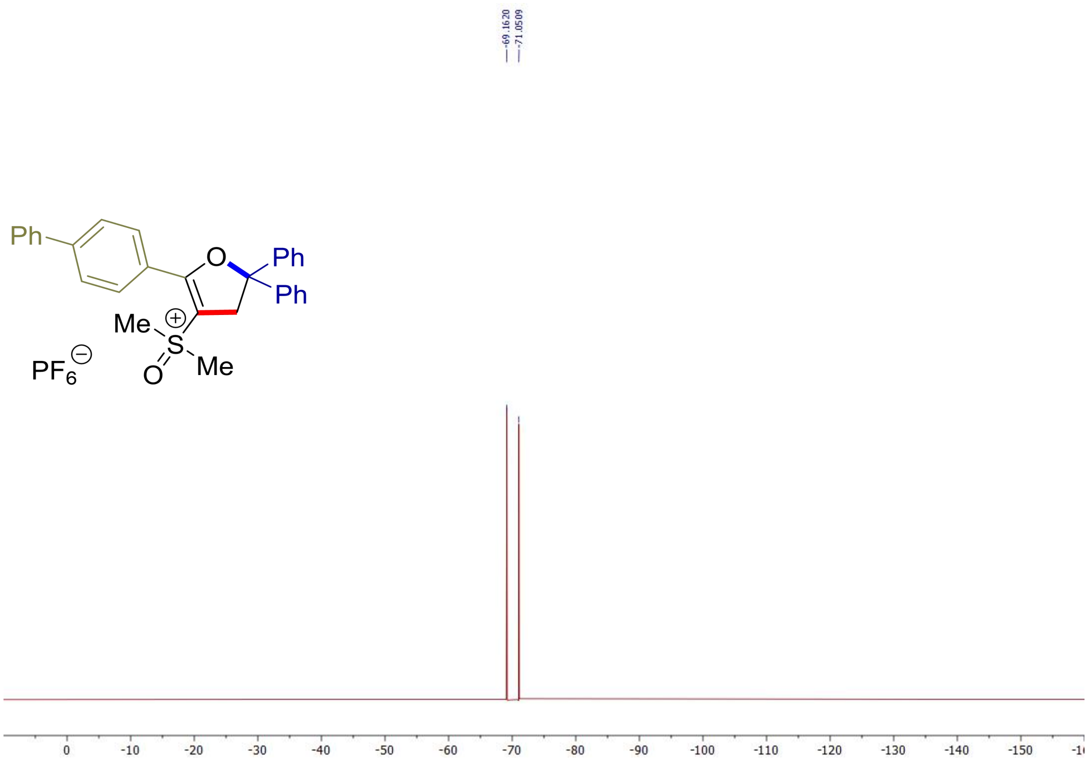

Supplementary Figure 165. ^{19}**F NMR** of the **7c** (376 MHz, 25 °C in DMSO-d_6)

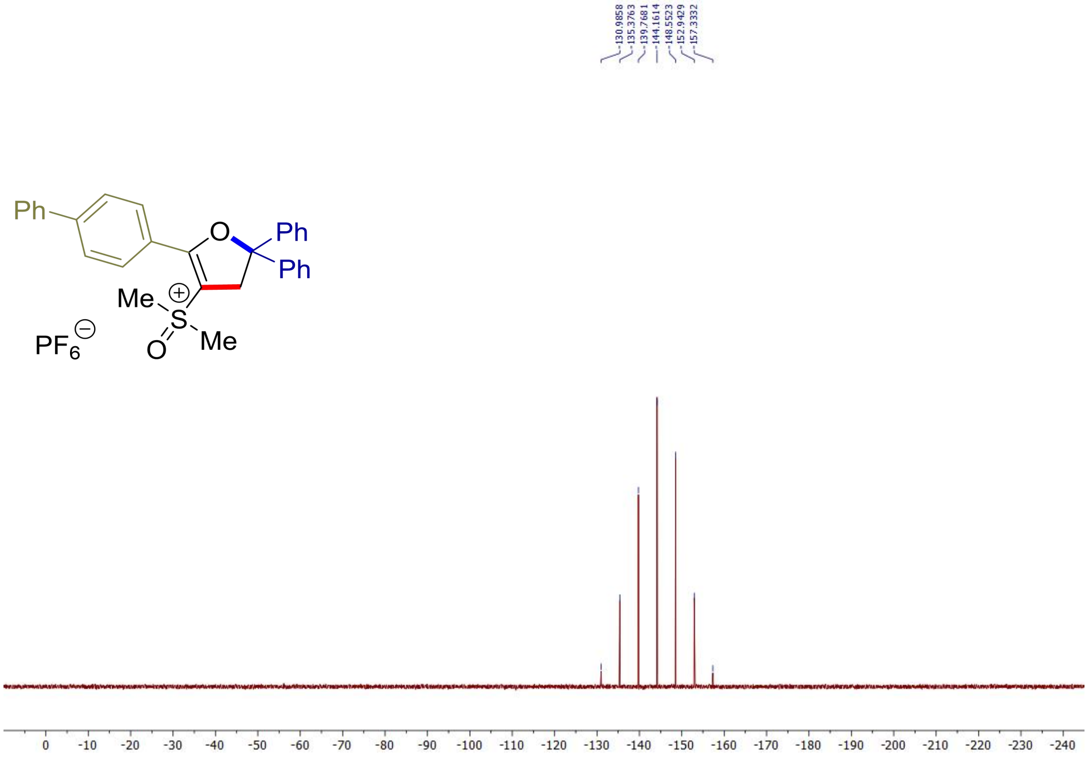

Supplementary Figure 166. ^{31}**P NMR** of the **7c** (162 MHz, 25 °C in DMSO-d_6)

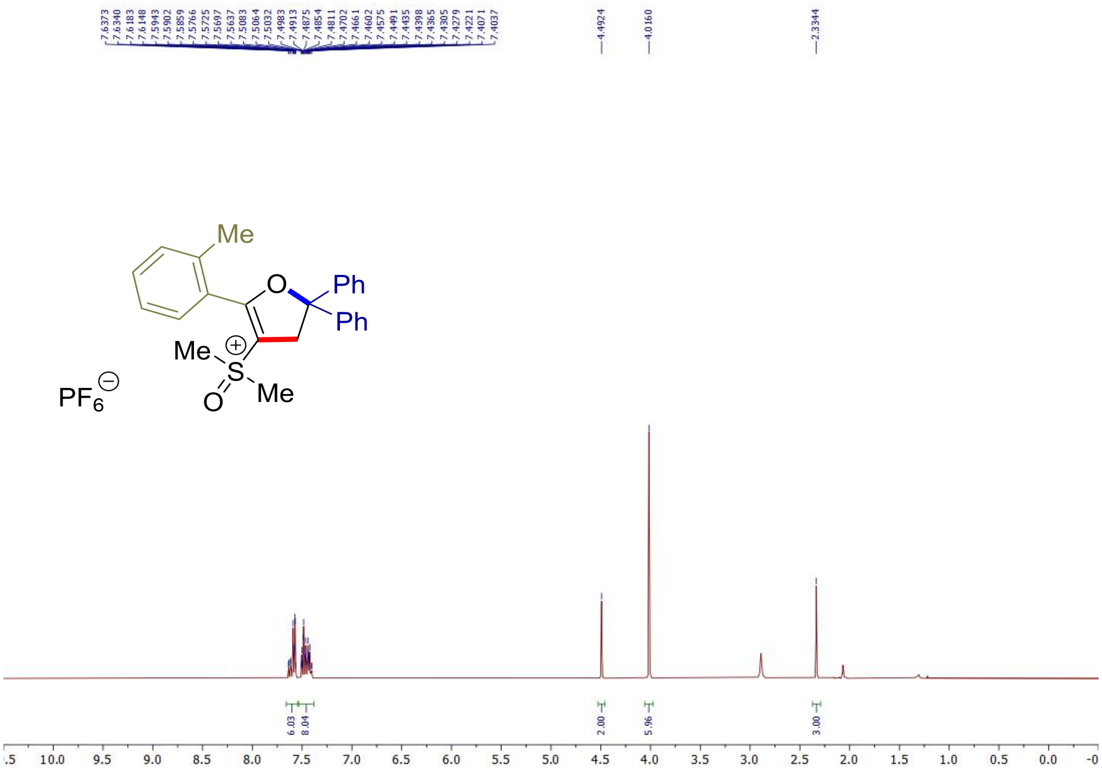

Supplementary Figure 167. ^{1}H NMR of the **7d** (400 MHz, 25 °C in Acetone-d_6)

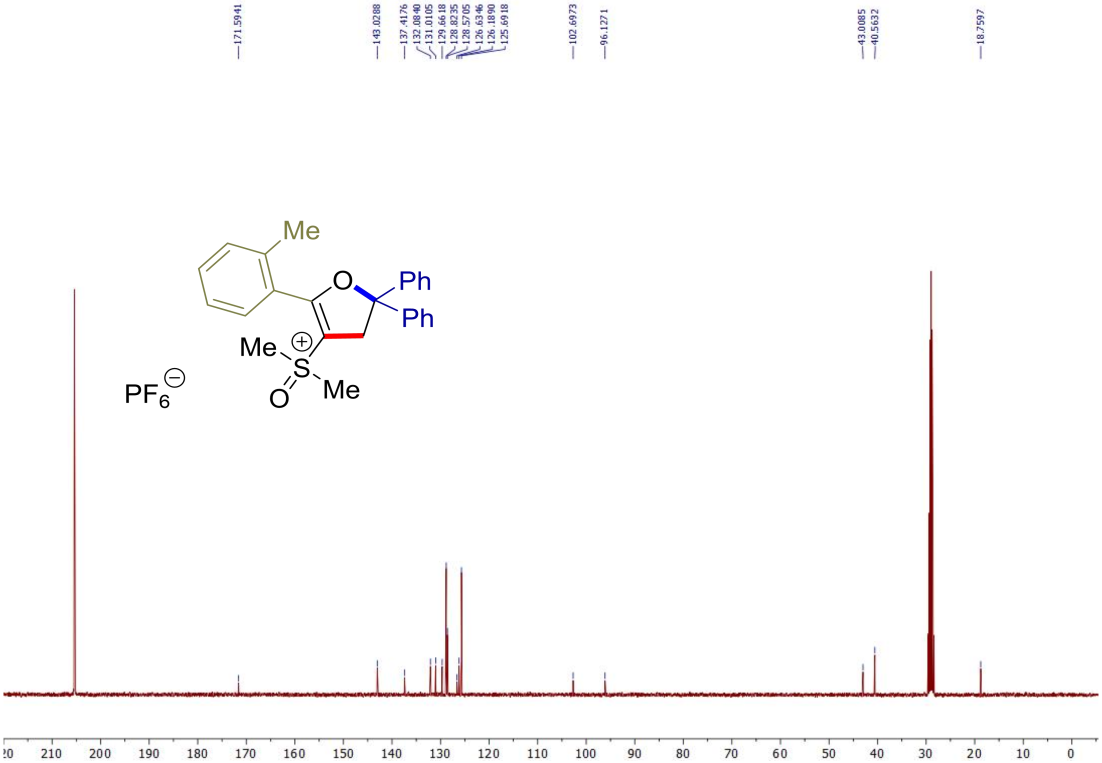

Supplementary Figure 168. ^{13}C NMR of the **7d** (101 MHz, 25 °C in Acetone-d_6)

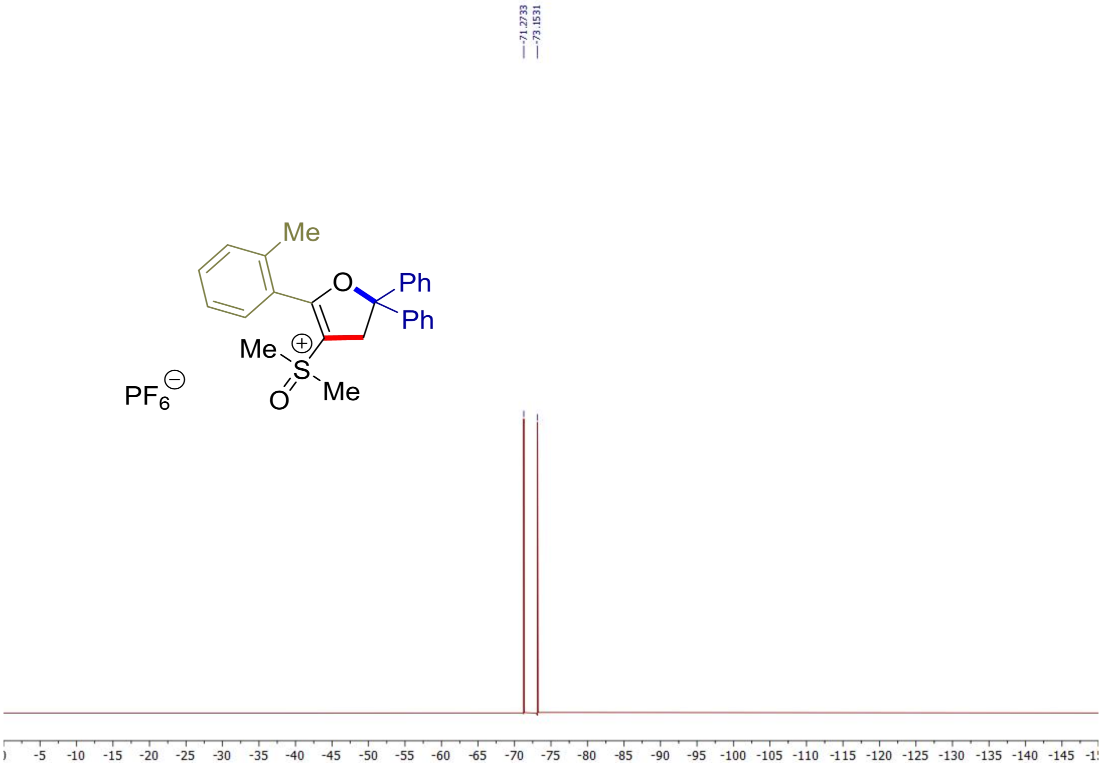

Supplementary Figure 169. ^{19}**F NMR** of the **7d** (376 MHz, 25 °C in Acetone-d_6)

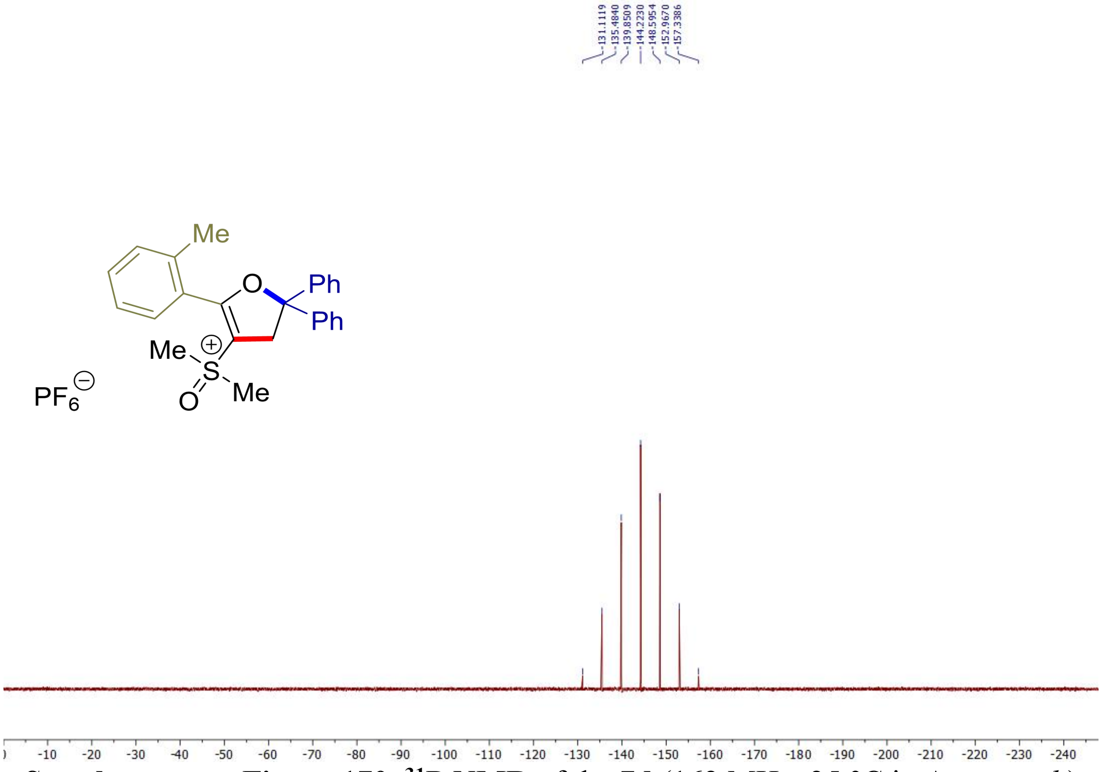

Supplementary Figure 170. ^{31}**P NMR** of the **7d** (162 MHz, 25 °C in Acetone-d_6)

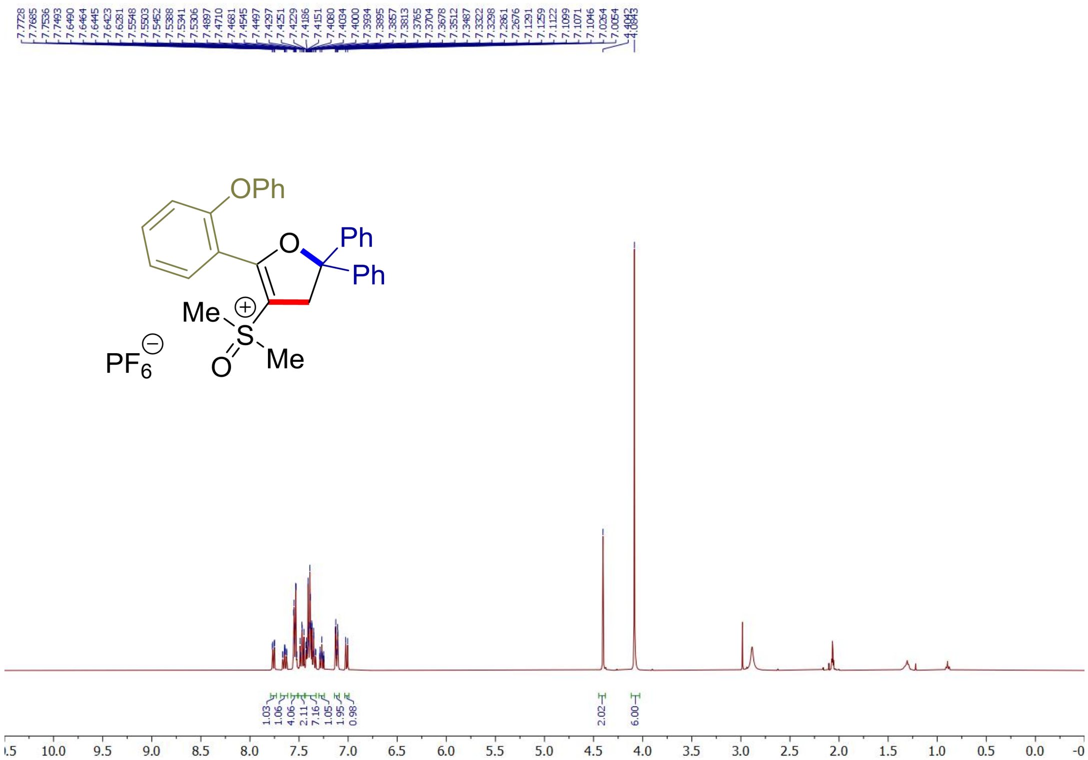

Supplementary Figure 171. ^{1}H NMR of the **7e** (400 MHz, 25 °C in Acetone-d_6)

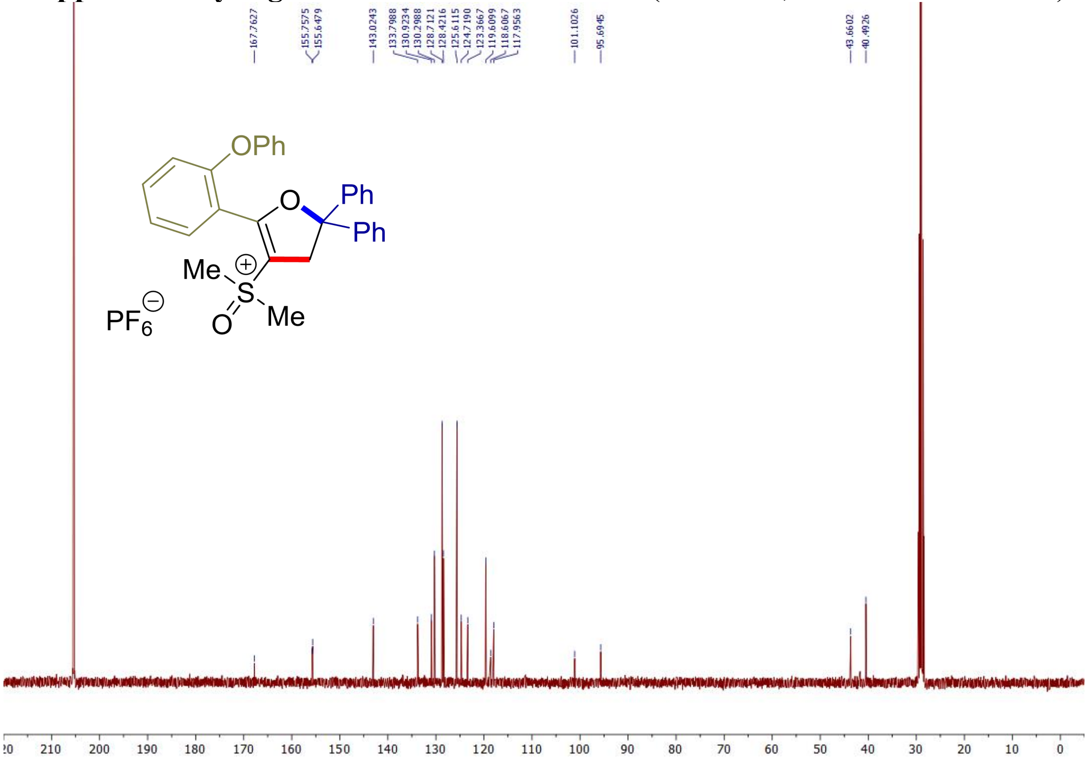

Supplementary Figure 172. ^{13}C NMR of the **7e** (101 MHz, 25 °C in Acetone-d_6)

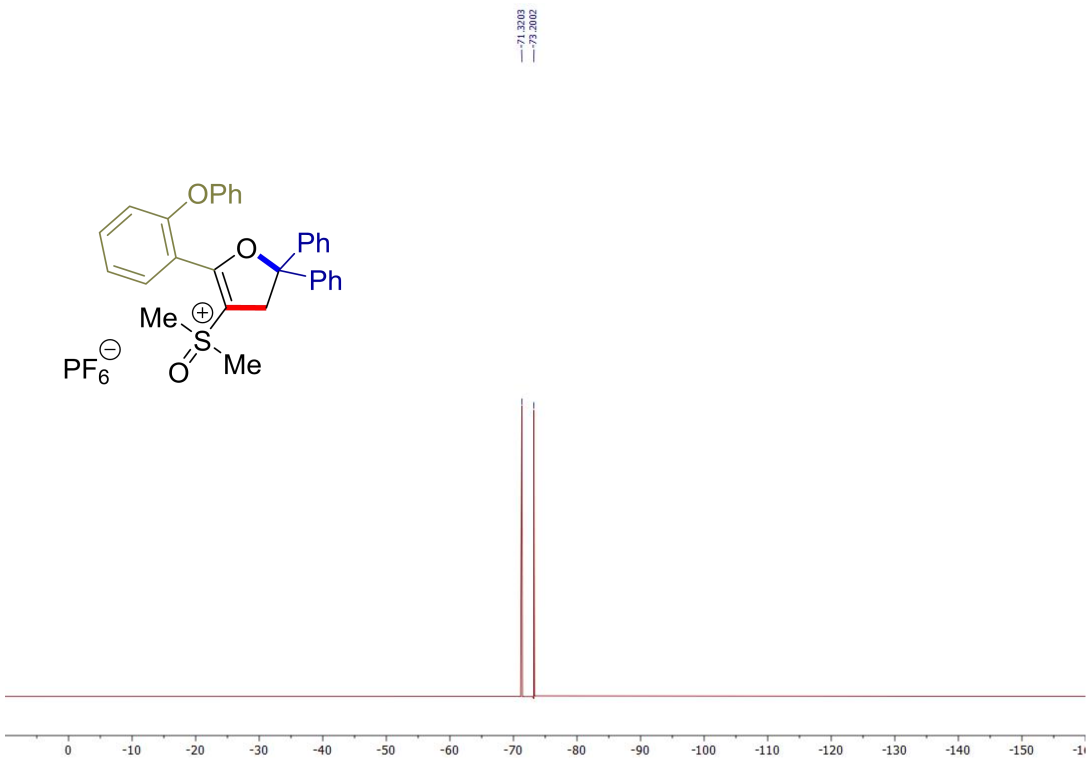

Supplementary Figure 173. ^{19}**F NMR** of the **7e** (376 MHz, 25 °C in Acetone-d_6)

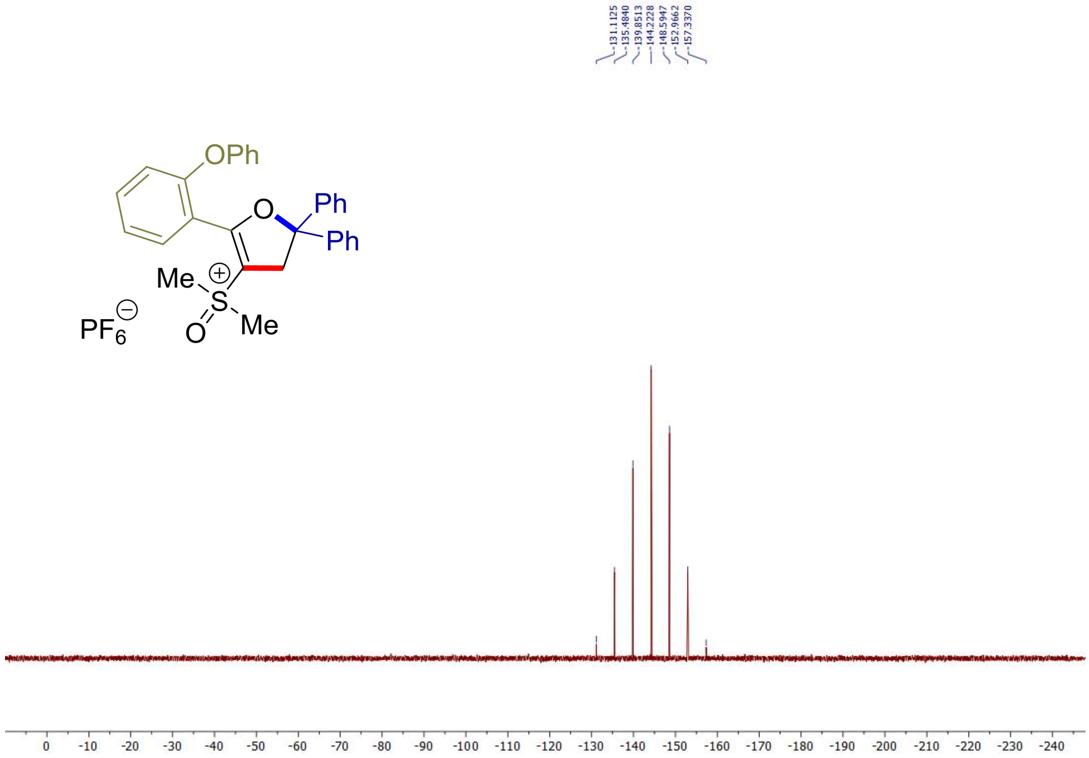

Supplementary Figure 174. ^{31}**P NMR** of the **7e** (162 MHz, 25 °C in Acetone-d_6)

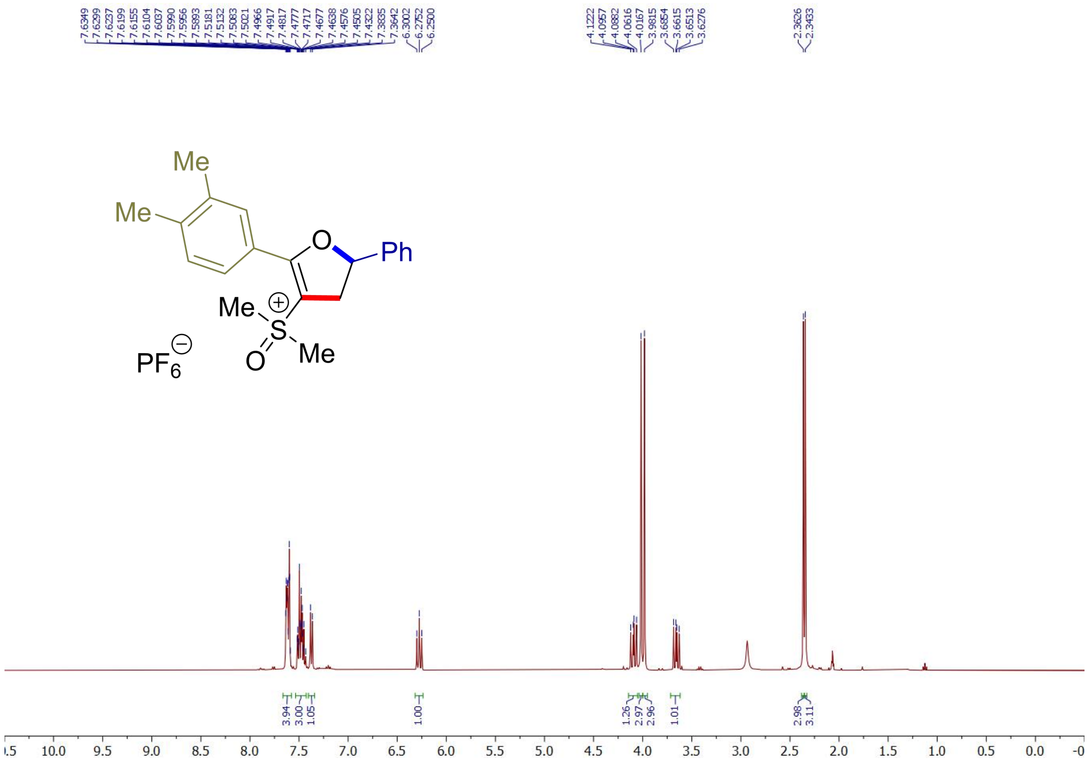

Supplementary Figure 175. ^{1}H NMR of the **7f** (400 MHz, 25 °C in Acetone-d_6)

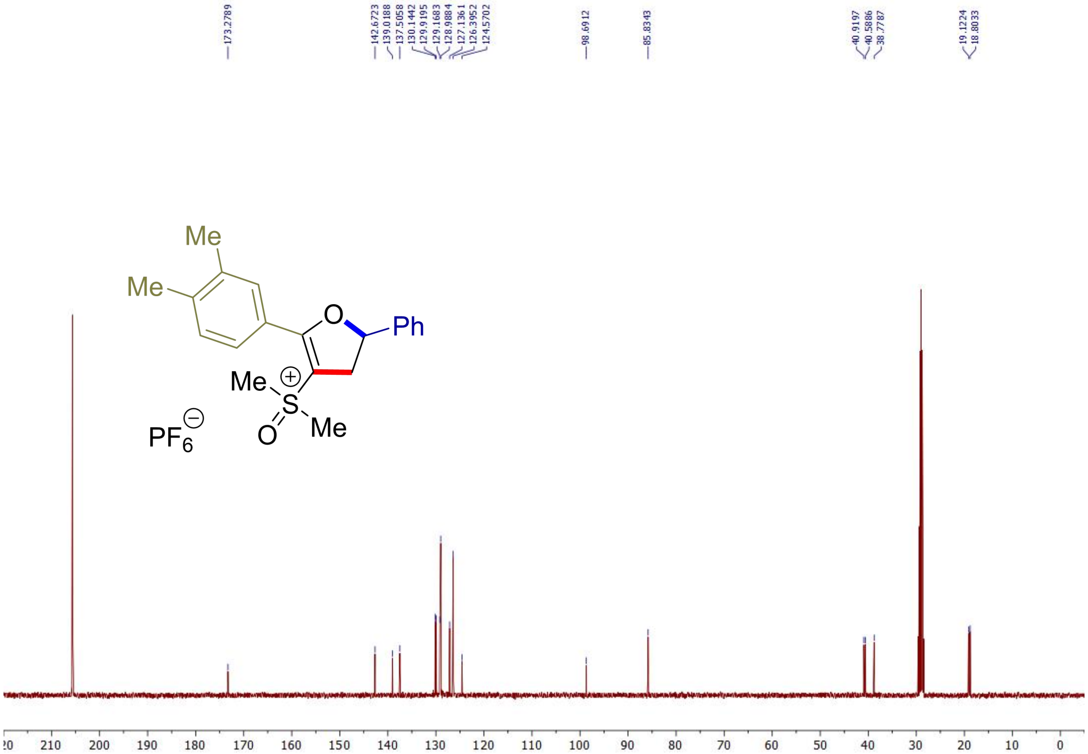

Supplementary Figure 176. ^{13}C NMR of the **7f** (101 MHz, 25 °C in Acetone-d_6)

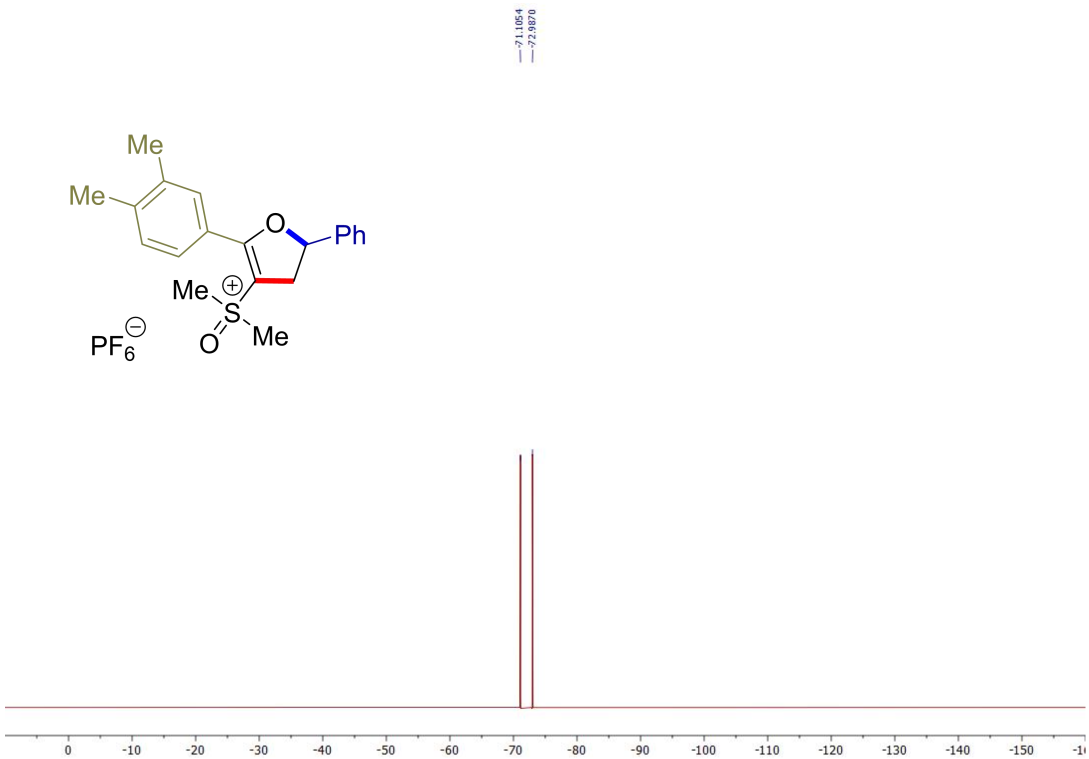

Supplementary Figure 177. ^{19}**F NMR** of the **7f** (376 MHz, 25 °C in Acetone-d_6)

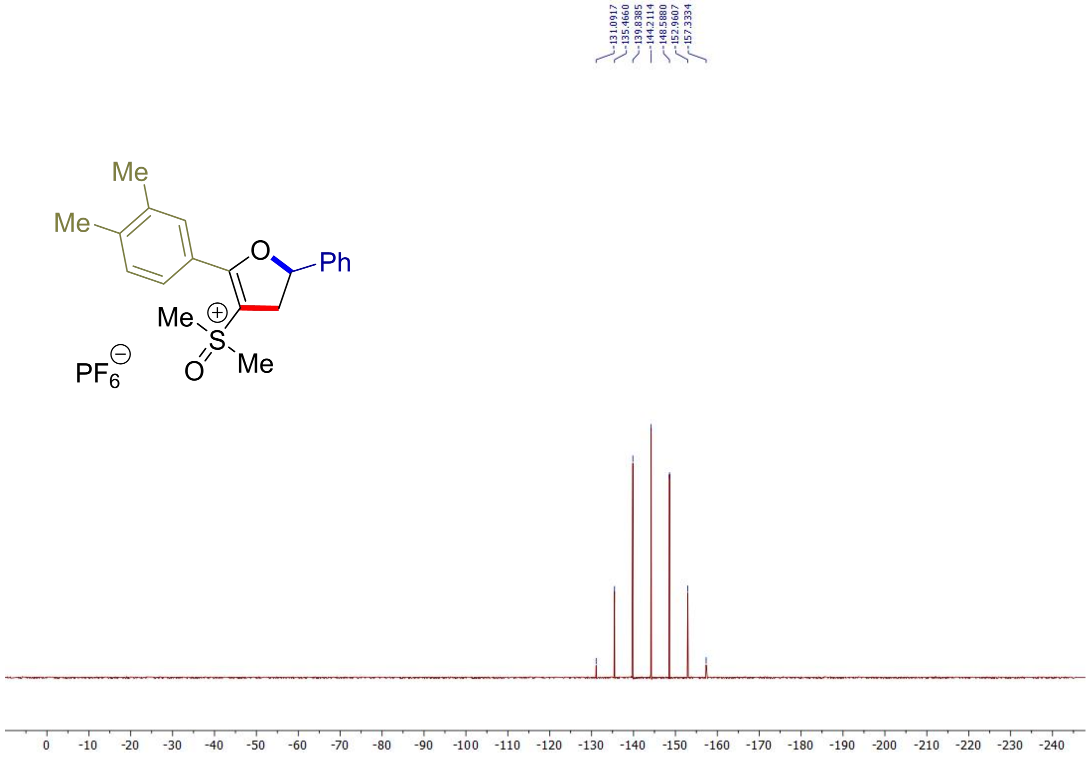

Supplementary Figure 178. ^{31}**P NMR** of the **7f** (162 MHz, 25 °C in Acetone-d_6)

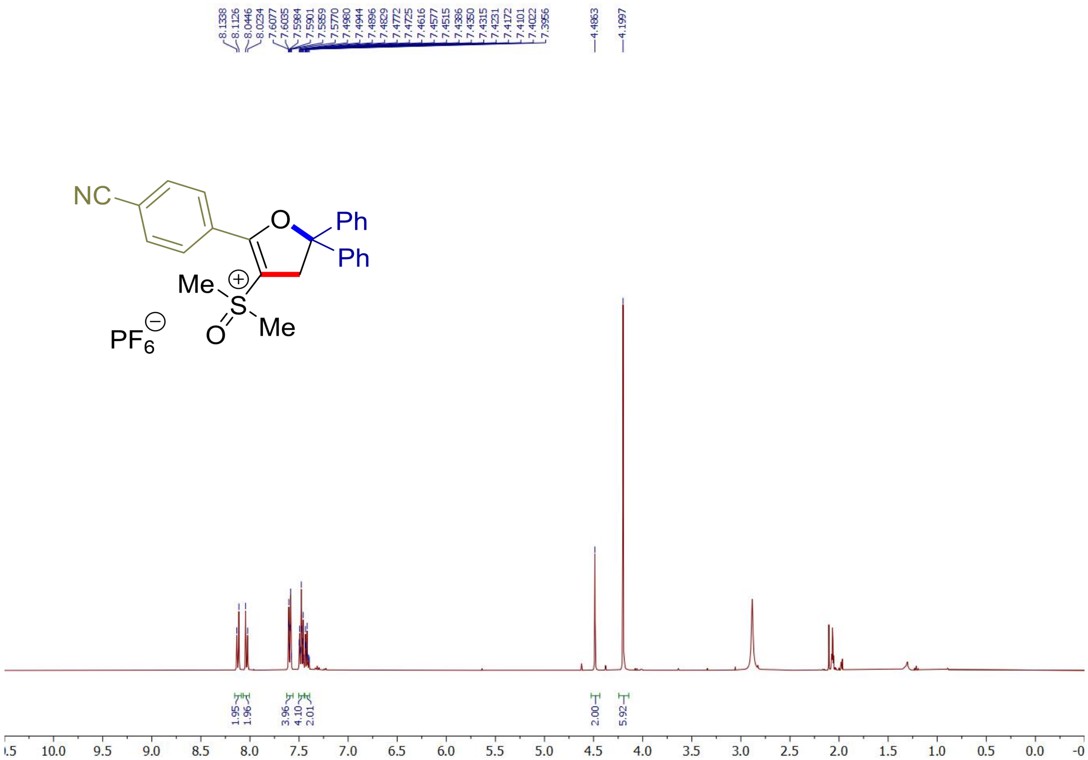

Supplementary Figure 179. ^{1}H NMR of the **7g** (400 MHz, 25 °C in Acetone-d_6)

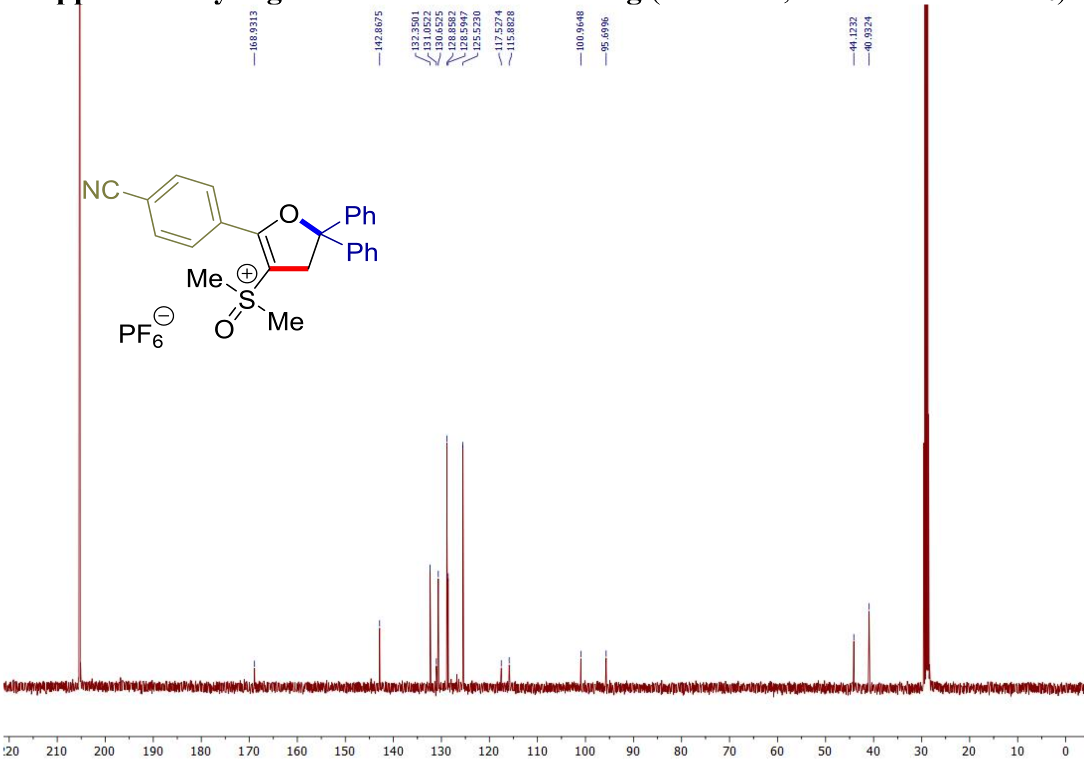

Supplementary Figure 180. ^{13}C NMR of the **7g** (101 MHz, 25 °C in Acetone-d_6)

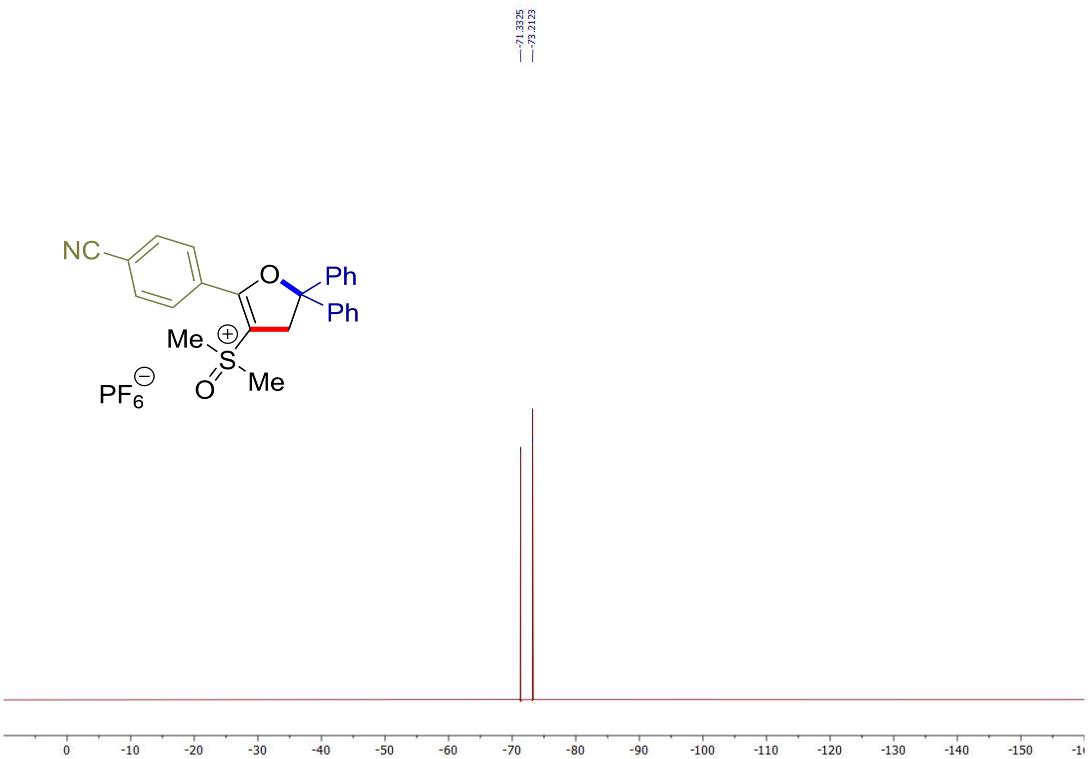

Supplementary Figure 181. **^{19}F NMR** of the **7g** (376 MHz, 25 °C in Acetone-d_6)

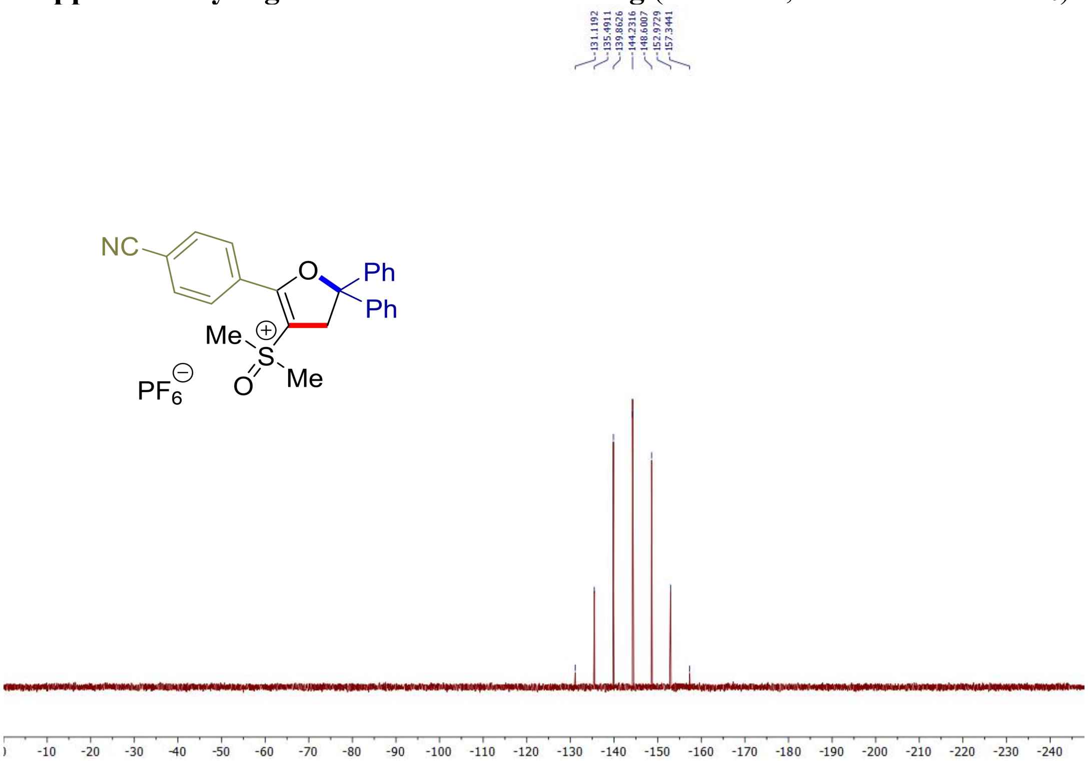

Supplementary Figure 182. **^{31}P NMR** of the **7g** (162 MHz, 25 °C in Acetone-d_6)

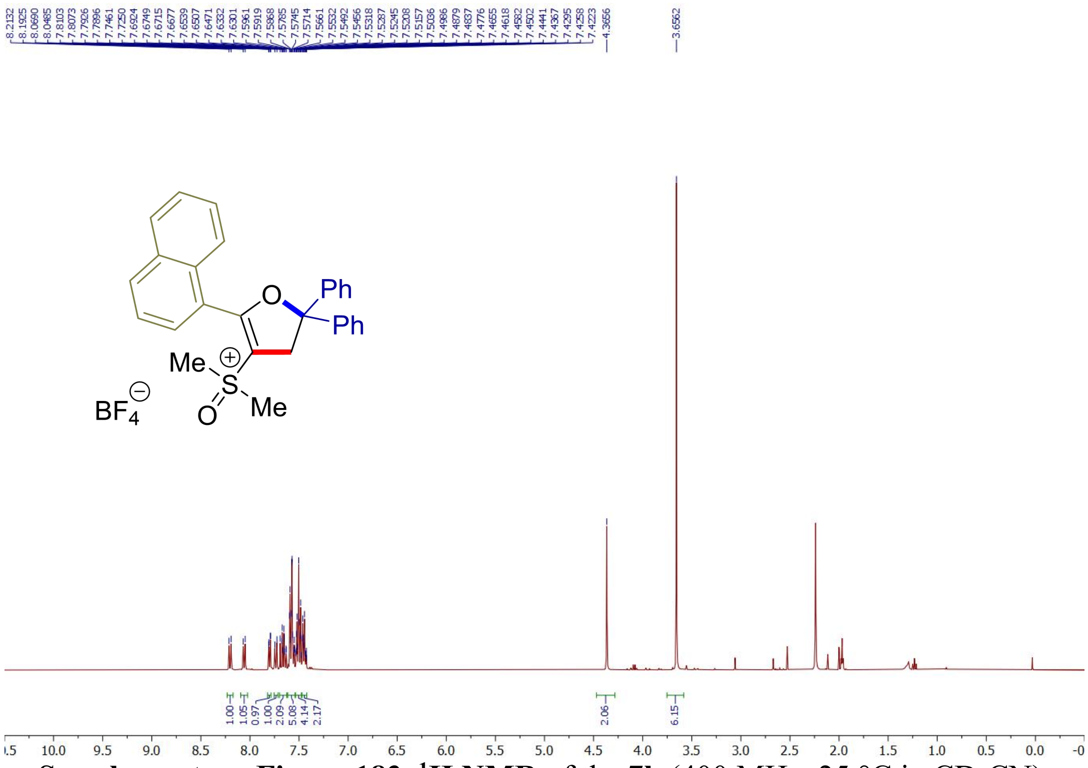

Supplementary Figure 183. ^{1}H NMR of the **7h** (400 MHz, 25 °C in CD_3CN)

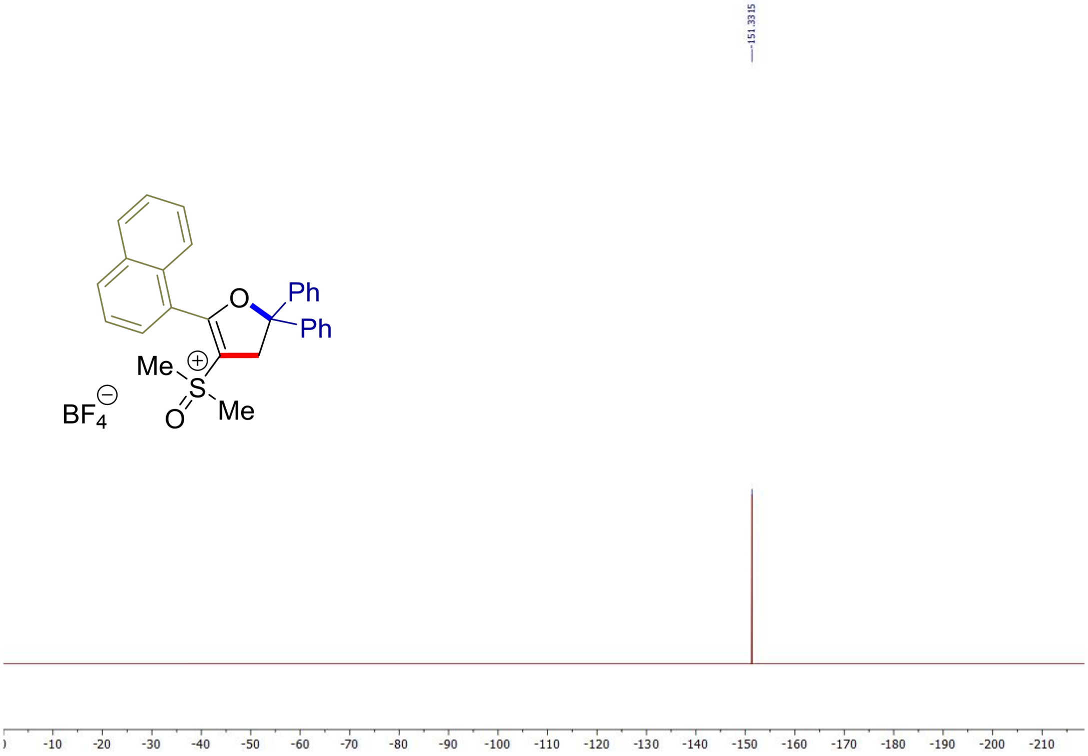

Supplementary Figure 184. ^{19}F NMR of the **7h** (376 MHz, 25 °C in CD_3CN)

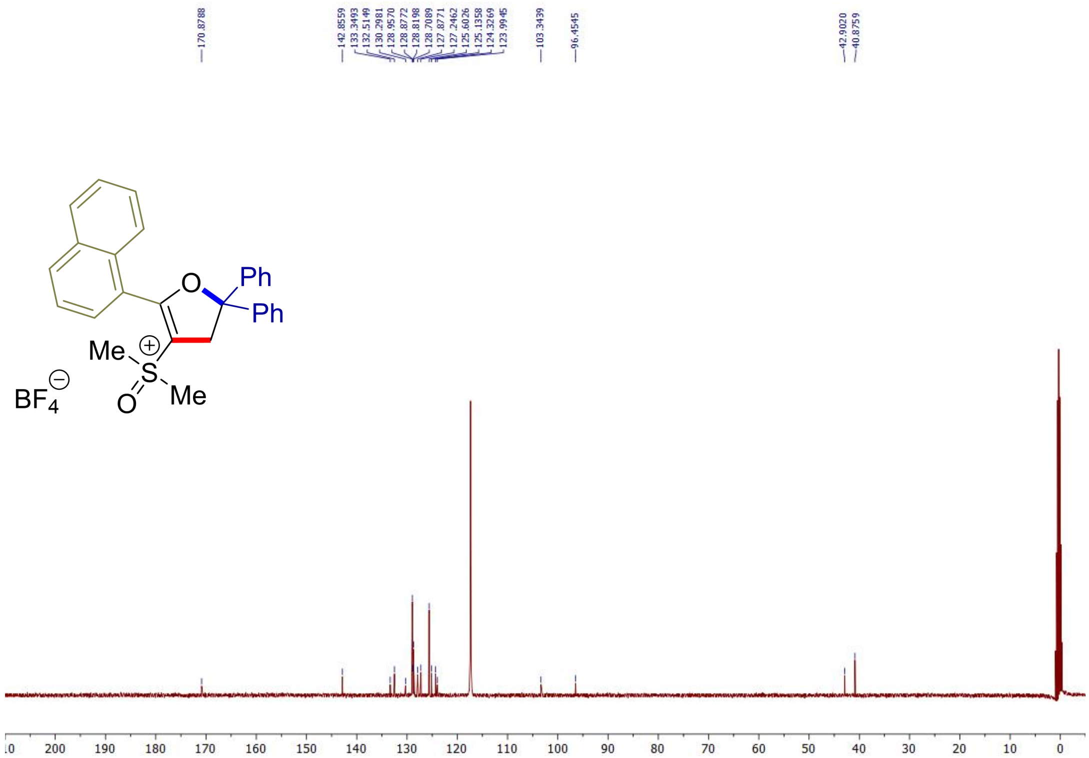

Supplementary Figure 185. ^{13}C NMR of the **7h** (101 MHz, 25 °C in CD_3CN)

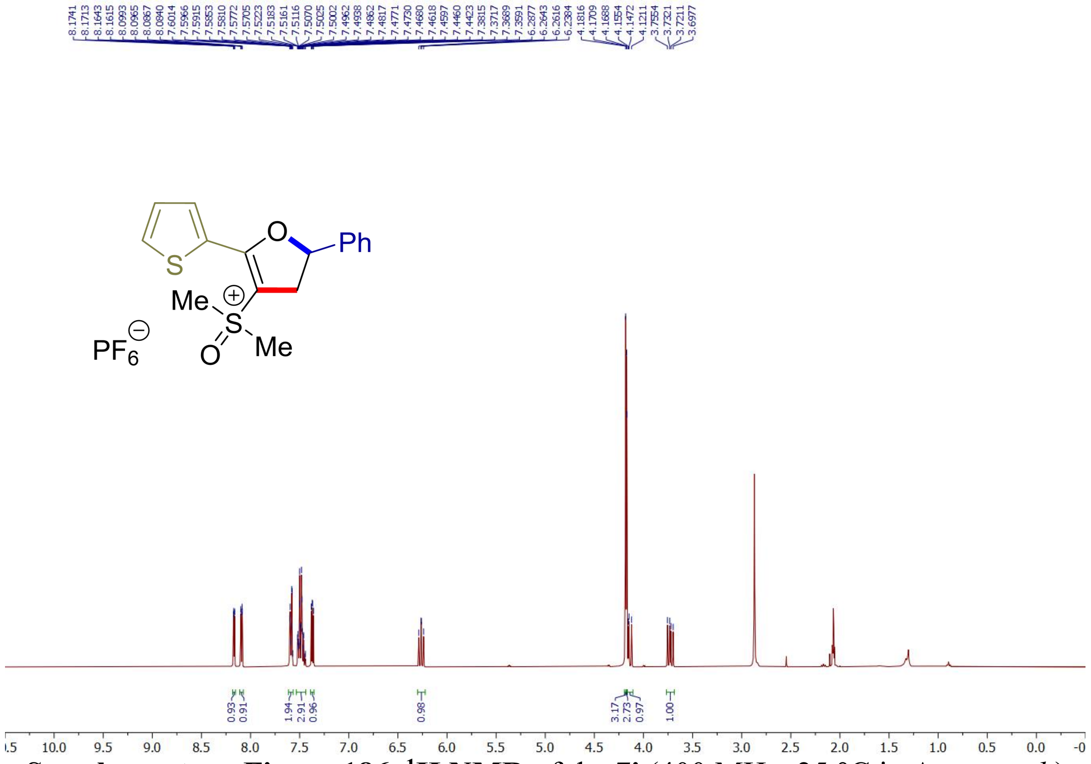

Supplementary Figure 186. ^{1}**H NMR** of the **7i** (400 MHz, 25 °C in Acetone-d_6)

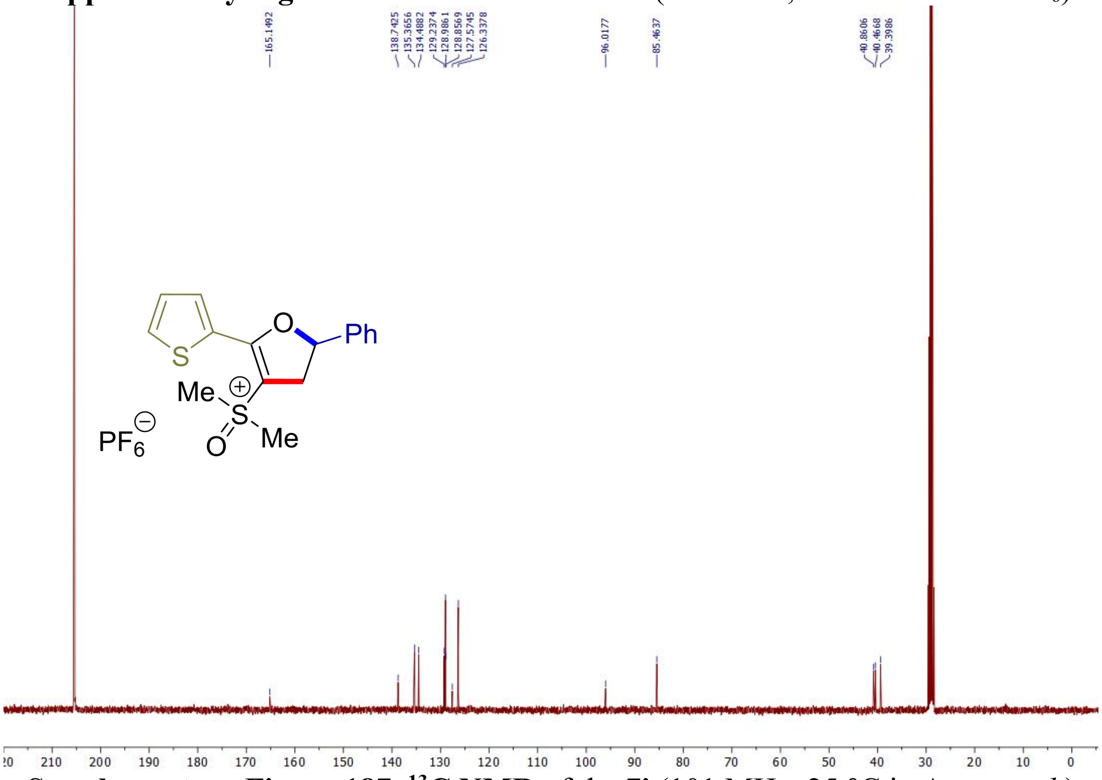

Supplementary Figure 187. ^{13}**C NMR** of the **7i** (101 MHz, 25 °C in Acetone-d_6)

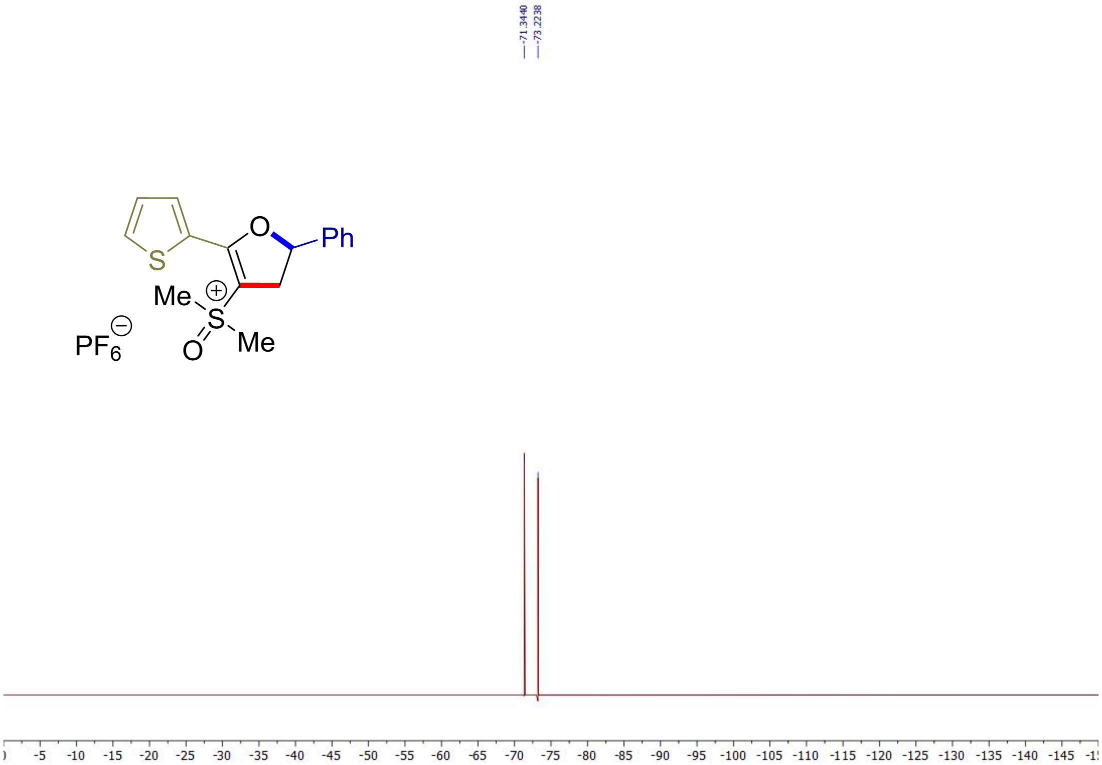

Supplementary Figure 188. ^{19}F NMR of the **7i** (376 MHz, 25 °C in Acetone-d_6)

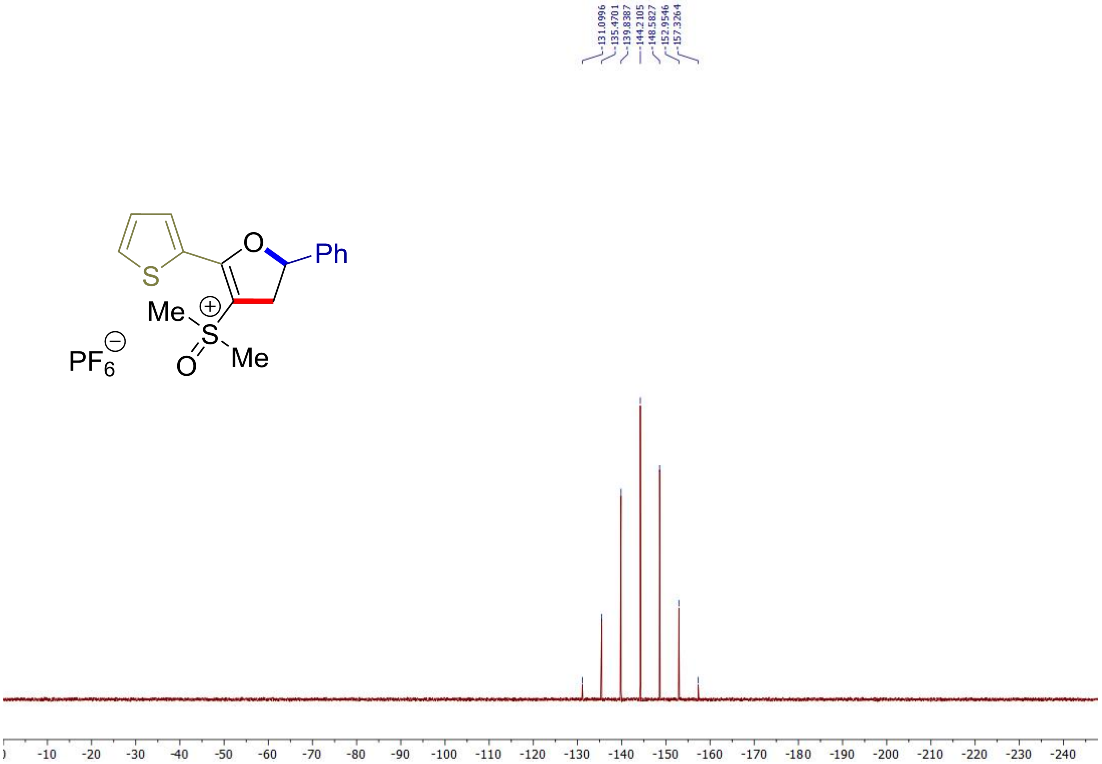

Supplementary Figure 189. ^{31}P NMR of the **7i** (162 MHz, 25 °C in Acetone-d_6)

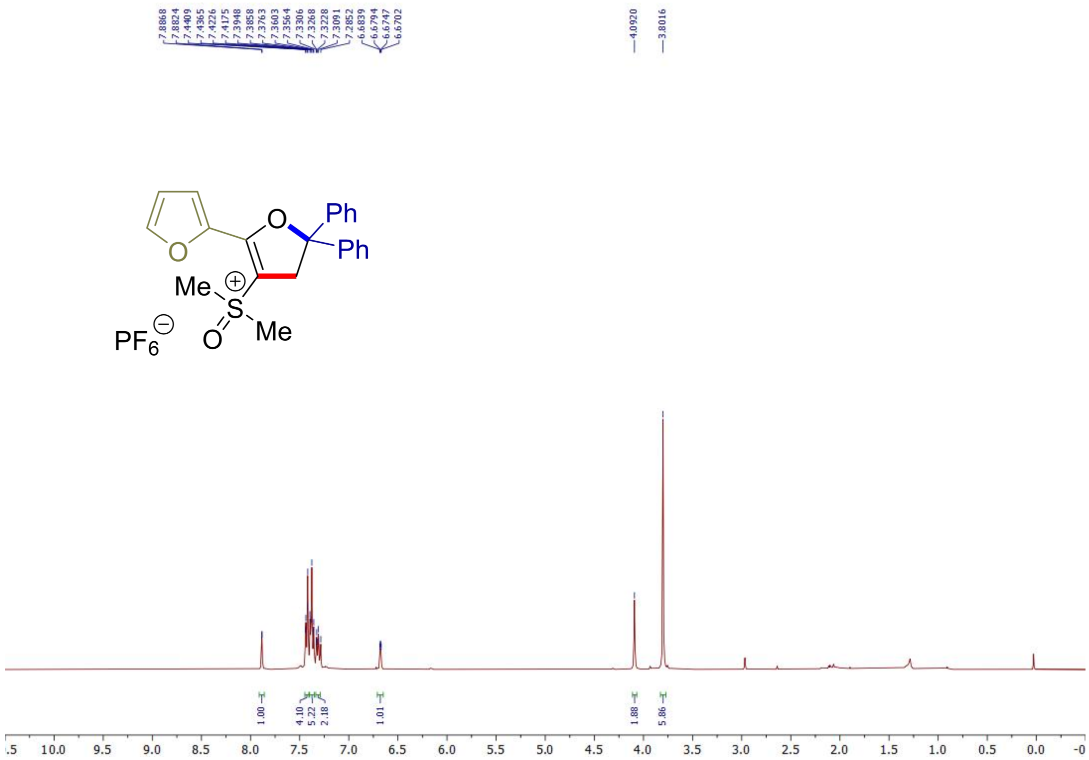

Supplementary Figure 190. ^{1}H NMR of the **7j** (400 MHz, 25 °C in $CDCl_3$)

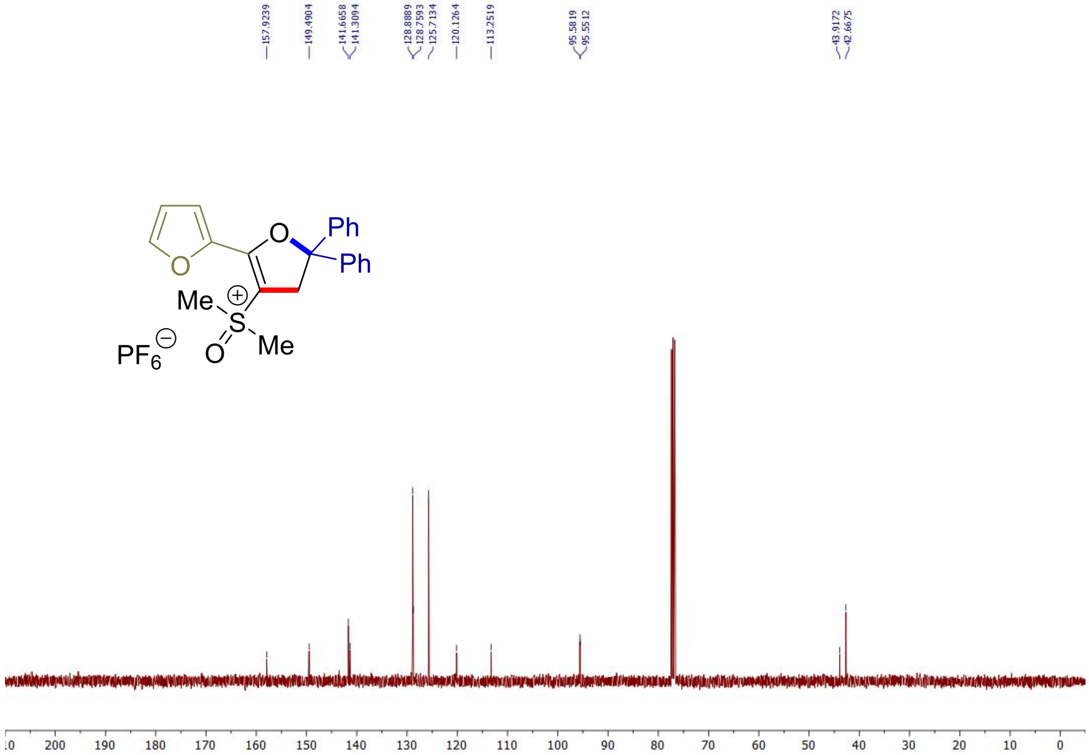

Supplementary Figure 191. ^{13}C NMR of the **7j** (101 MHz, 25 °C in $CDCl_3$)

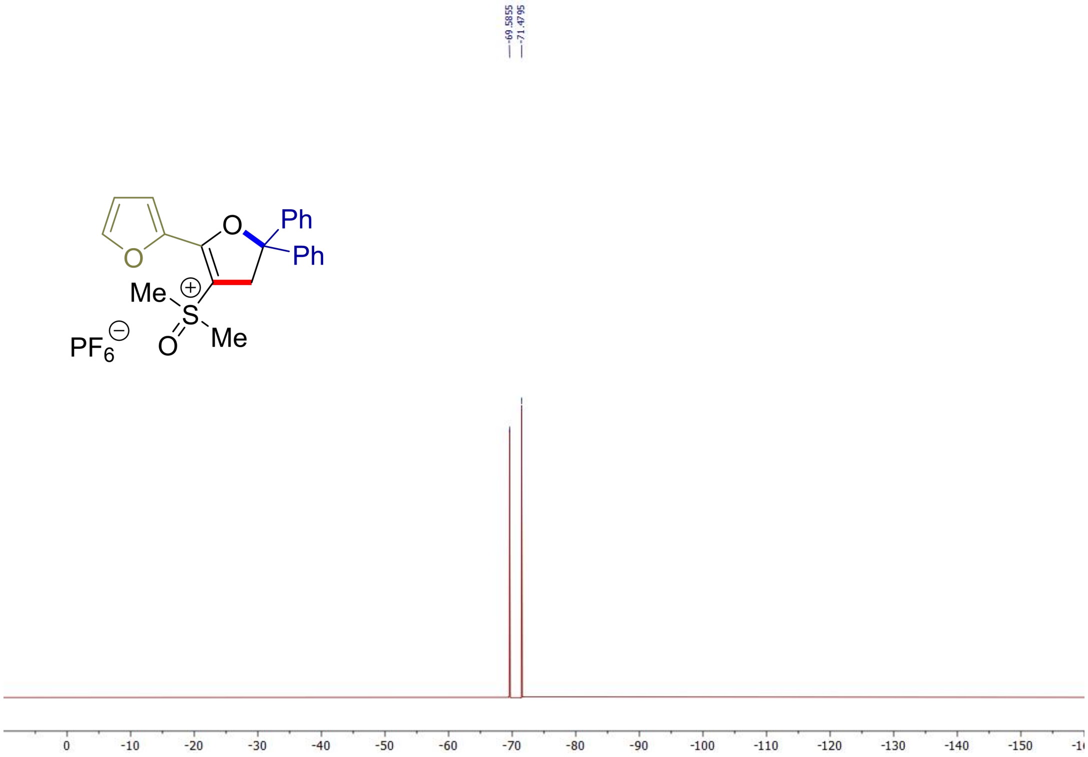

Supplementary Figure 192. ^{19}**F NMR** of the **7j** (376 MHz, 25 °C in $CDCl_3$)

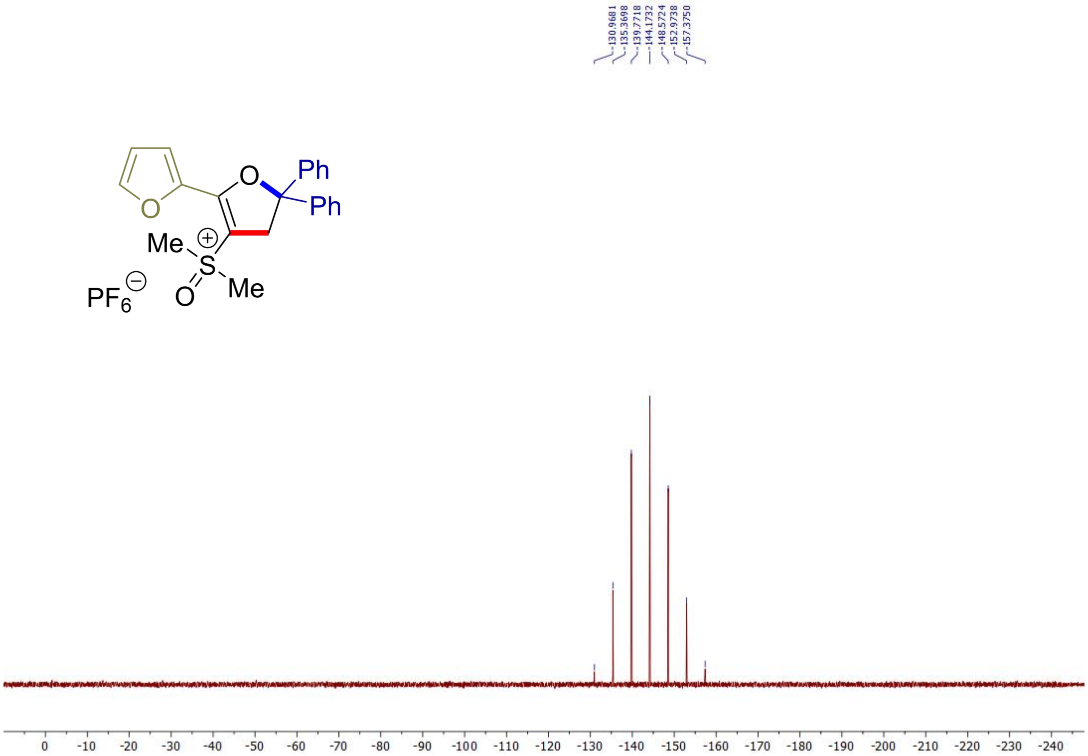

Supplementary Figure 193. ^{31}**P NMR** of the **7j** (162 MHz, 25 °C in $CDCl_3$)

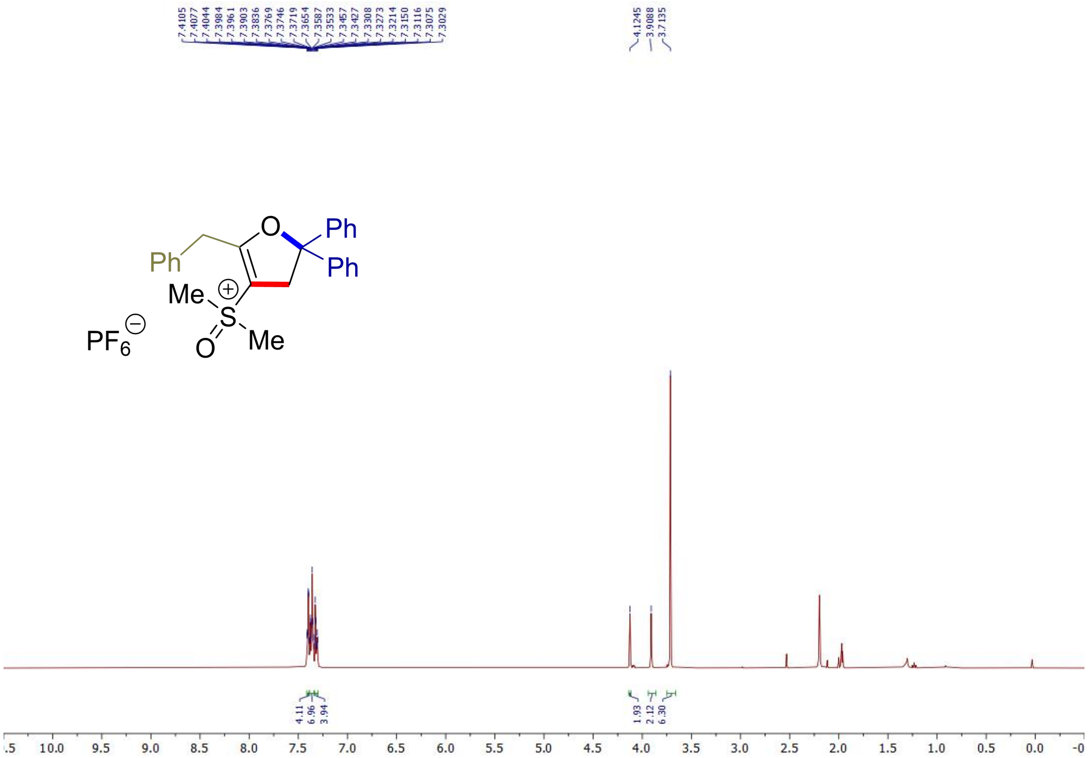

Supplementary Figure 194. ^{1}**H NMR** of the **7k** (376 MHz, 25 °C in CD_3CN)

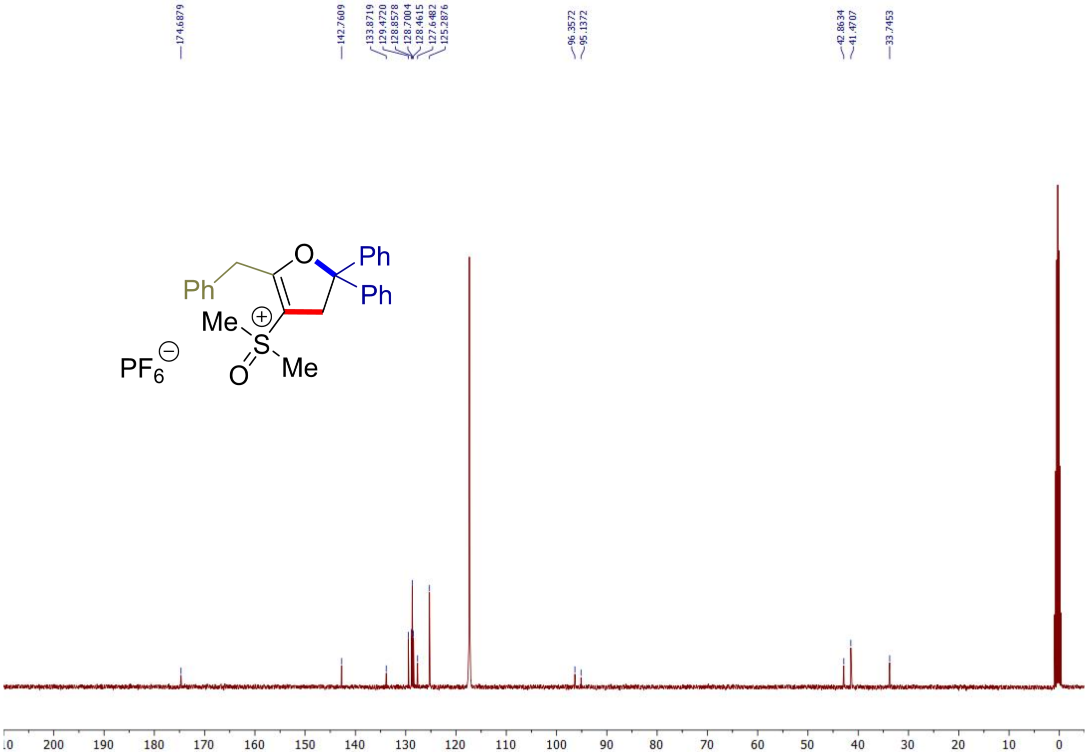

Supplementary Figure 195. ^{13}**C NMR** of the **7k** (101 MHz, 25 °C in CD_3CN)

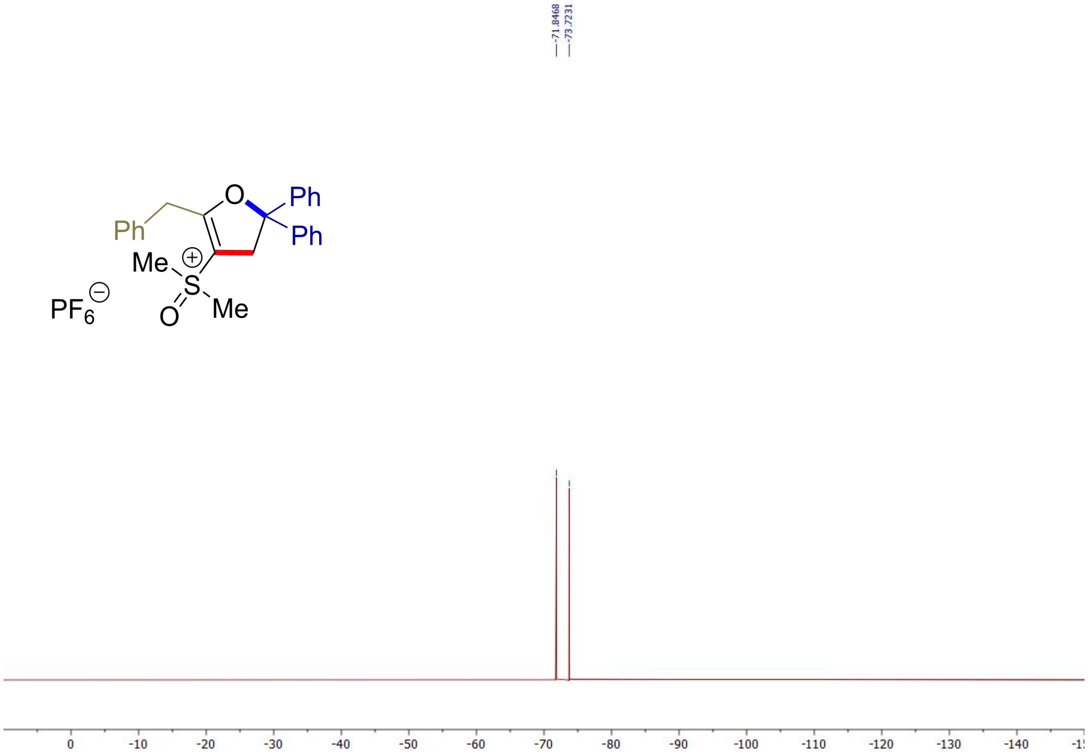

Supplementary Figure 196. ^{19}**F NMR** of the **7k** (376 MHz, 25 °C in CD_3CN)

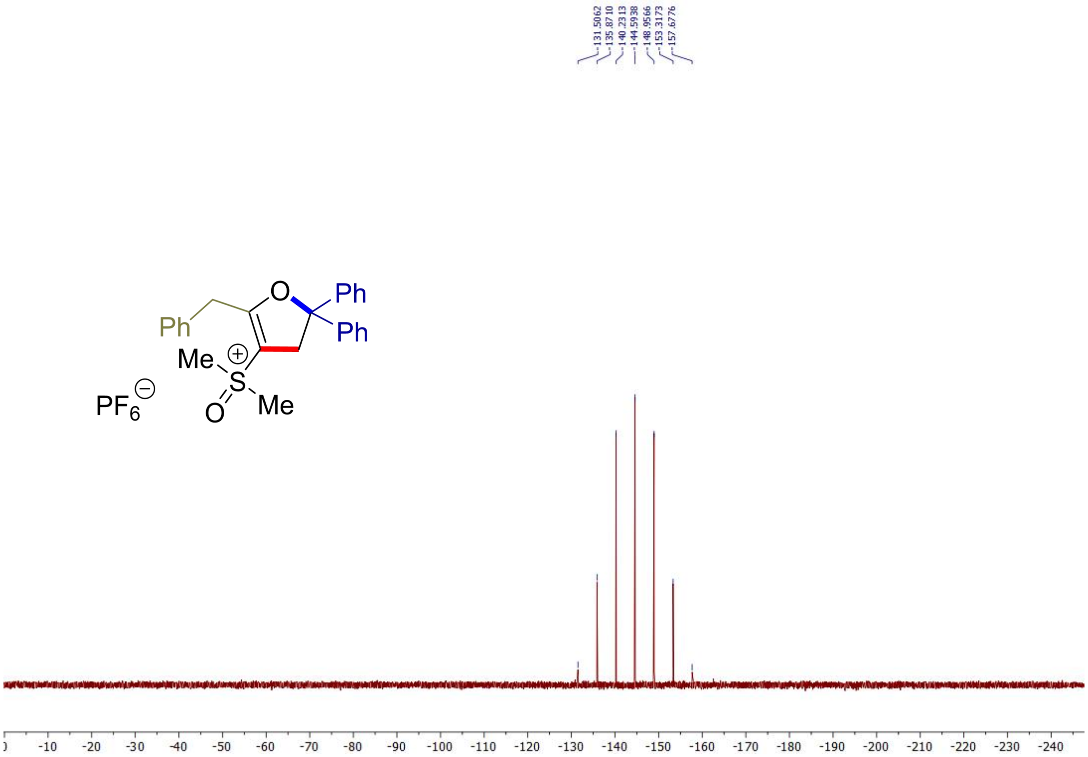

Supplementary Figure 197. ^{31}**P NMR** of the **7k** (162 MHz, 25 °C in CD_3CN)

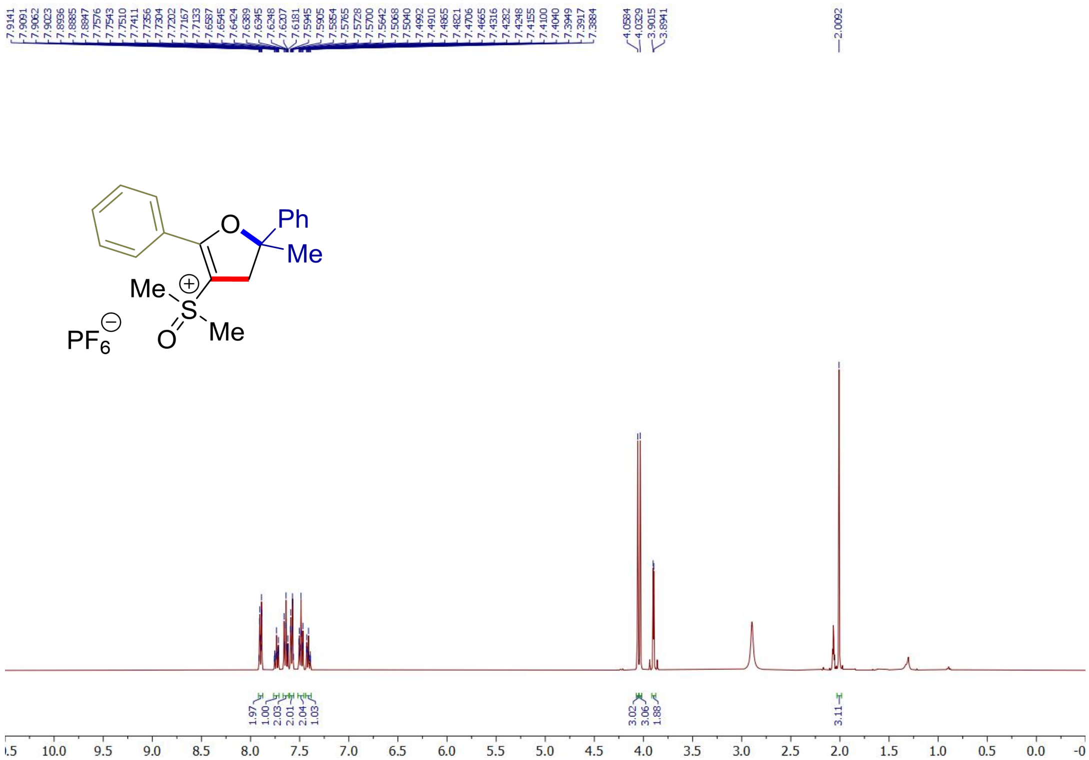

Supplementary Figure 198. ^{1}H NMR of the **7l** (376 MHz, 25 °C in Acetone-d_6)

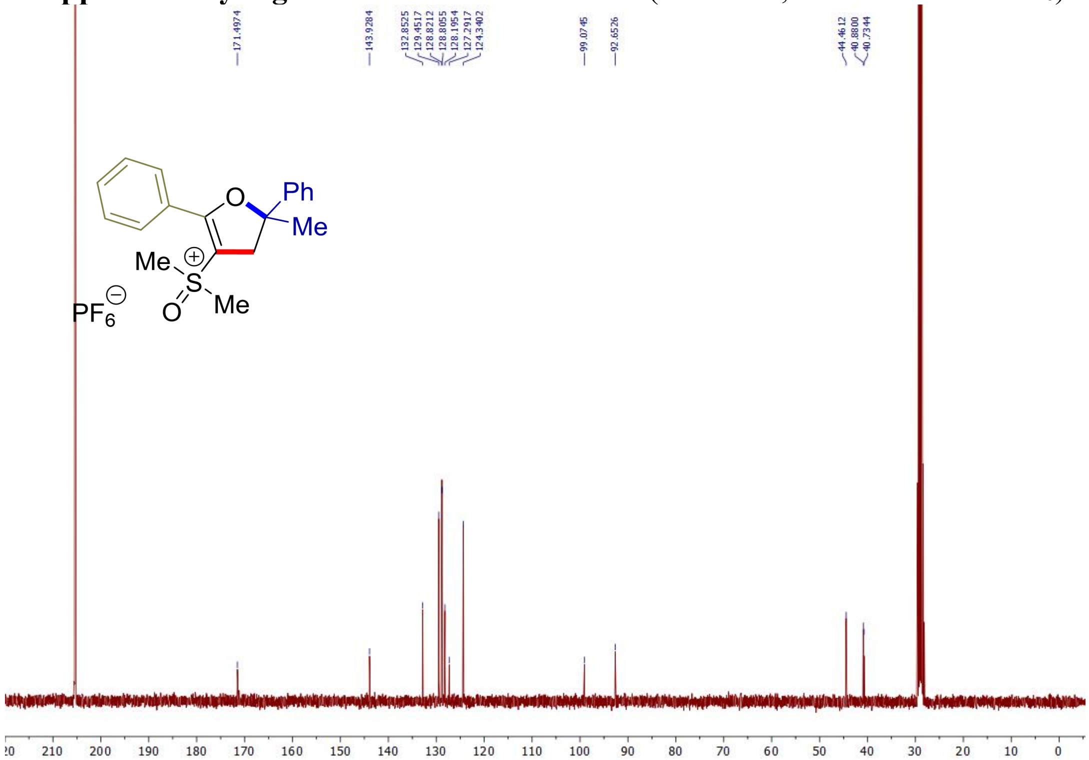

Supplementary Figure 199. ^{13}C NMR of the **7l** (101 MHz, 25 °C in Acetone-d_6)

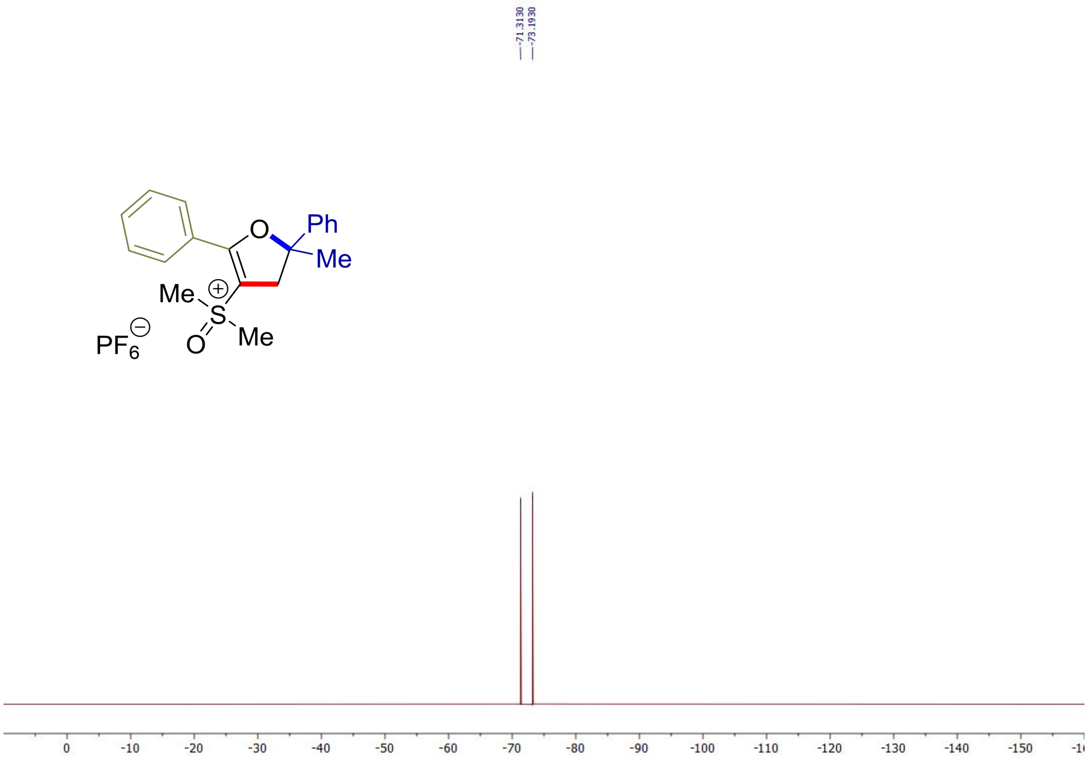

Supplementary Figure 200. ^{19}**F NMR** of the **7l** (376 MHz, 25 °C in Acetone-d_6)

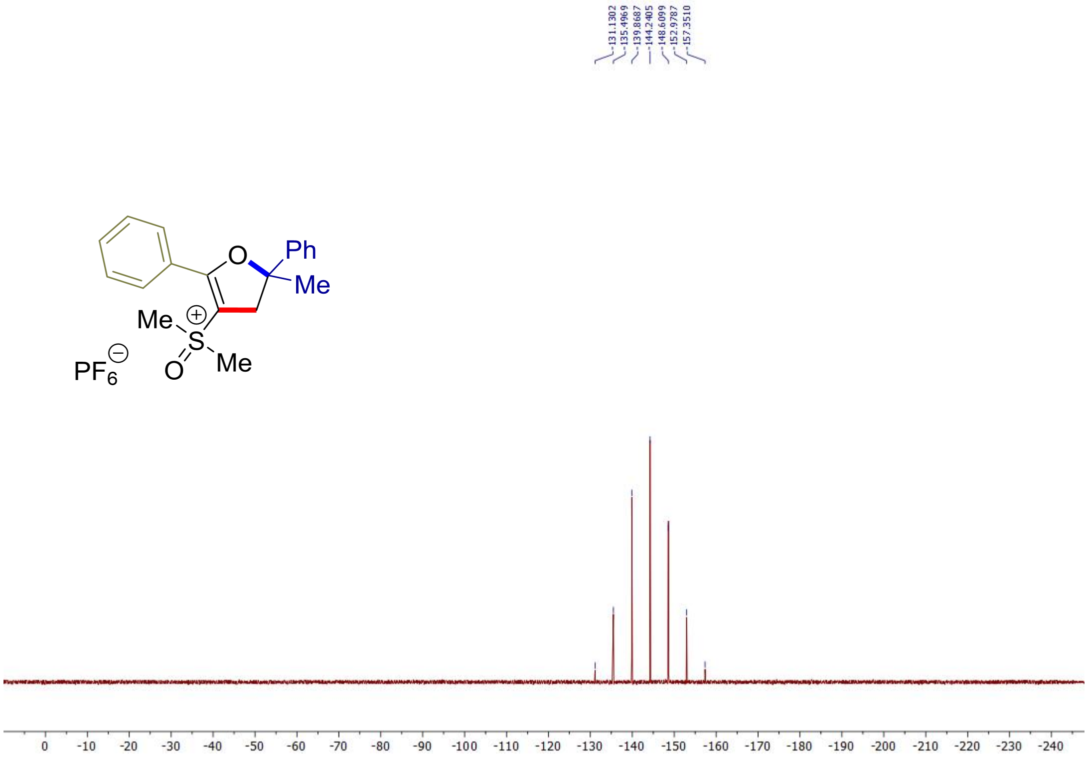

Supplementary Figure 201. ^{31}**P NMR** of the **7l** (162 MHz, 25 °C in Acetone-d_6)

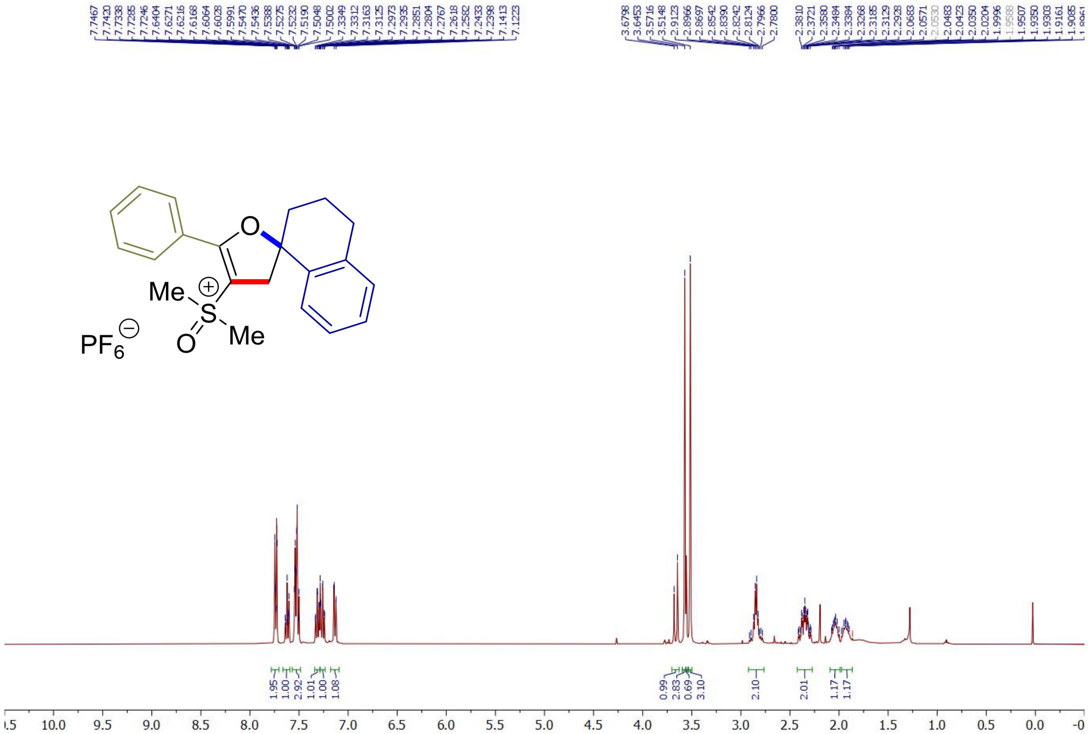

Supplementary Figure 202. ^{1}H NMR of the **7m** (400 MHz, 25 °C in $CDCl_3$)

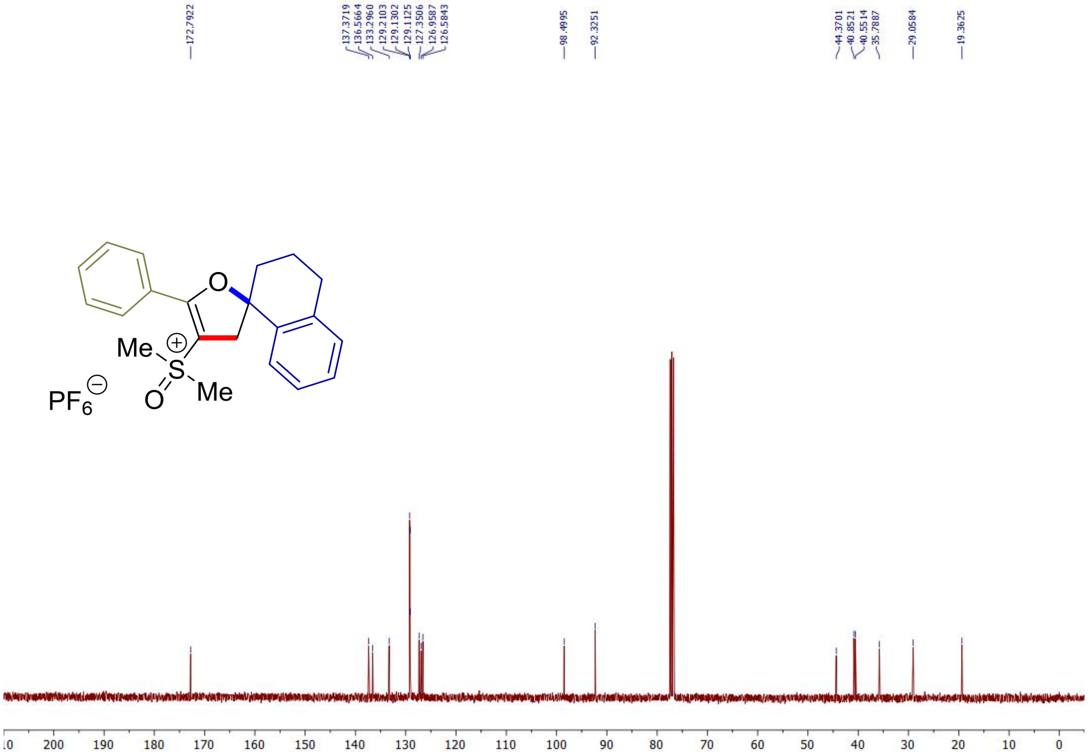

Supplementary Figure 203. ^{13}C NMR of the **7m** (101 MHz, 25 °C in $CDCl_3$)

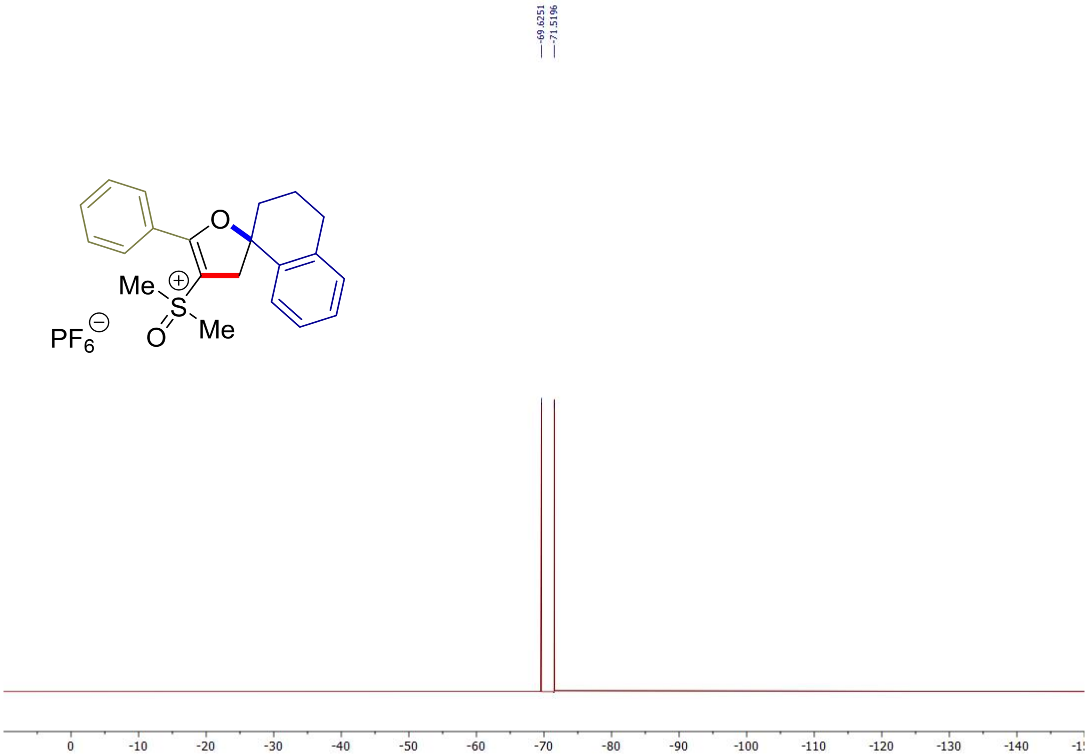

Supplementary Figure 204. ^{19}F NMR of the 7m (376 MHz, 25 °C in $CDCl_3$)

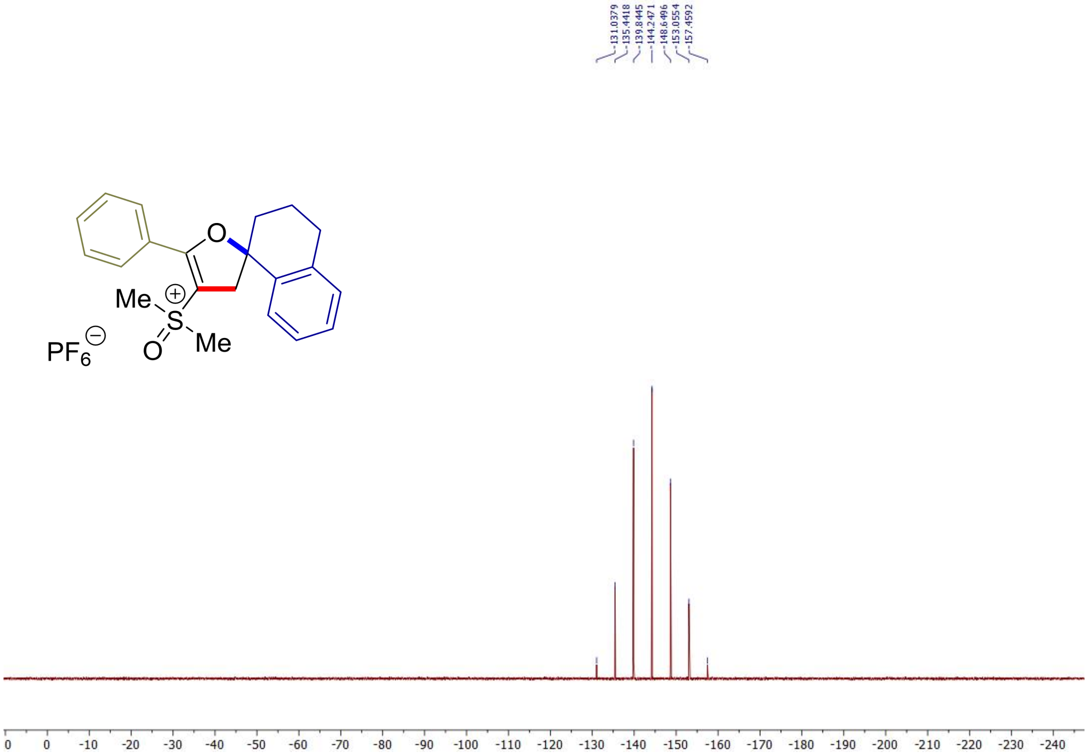

Supplementary Figure 205. ^{31}P NMR of the 7m (162 MHz, 25 °C in $CDCl_3$)

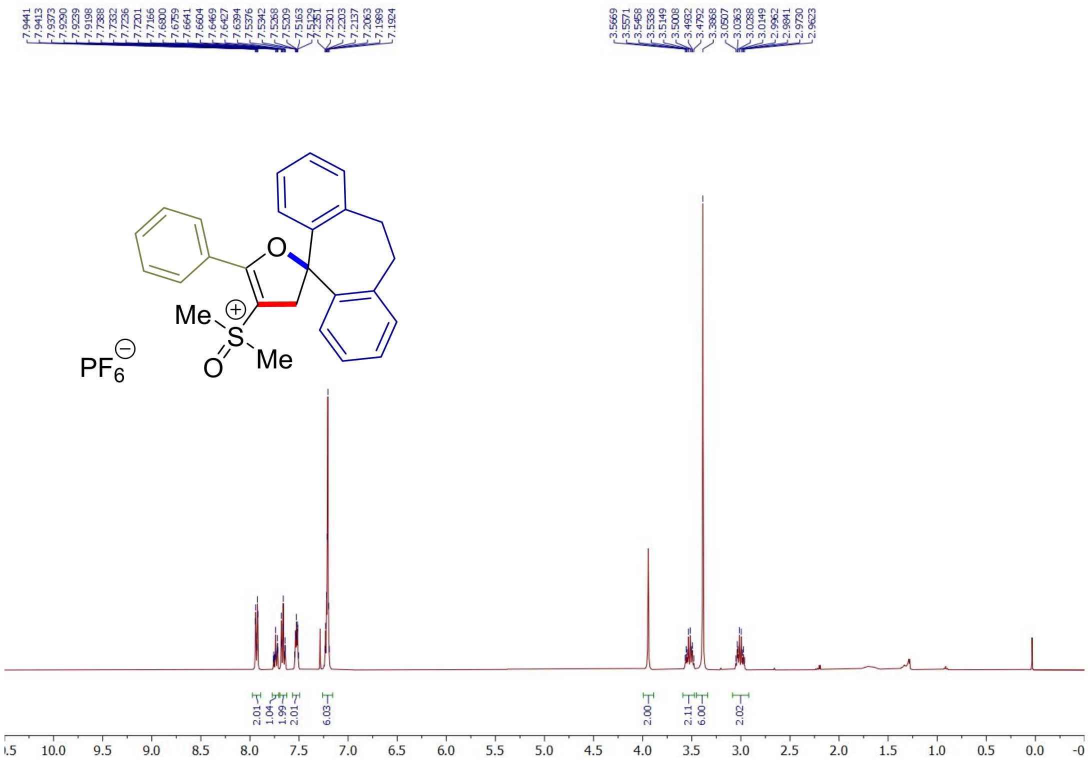

Supplementary Figure 206. ^{1}H NMR of the **7n** (400 MHz, 25 °C in $CDCl_3$)

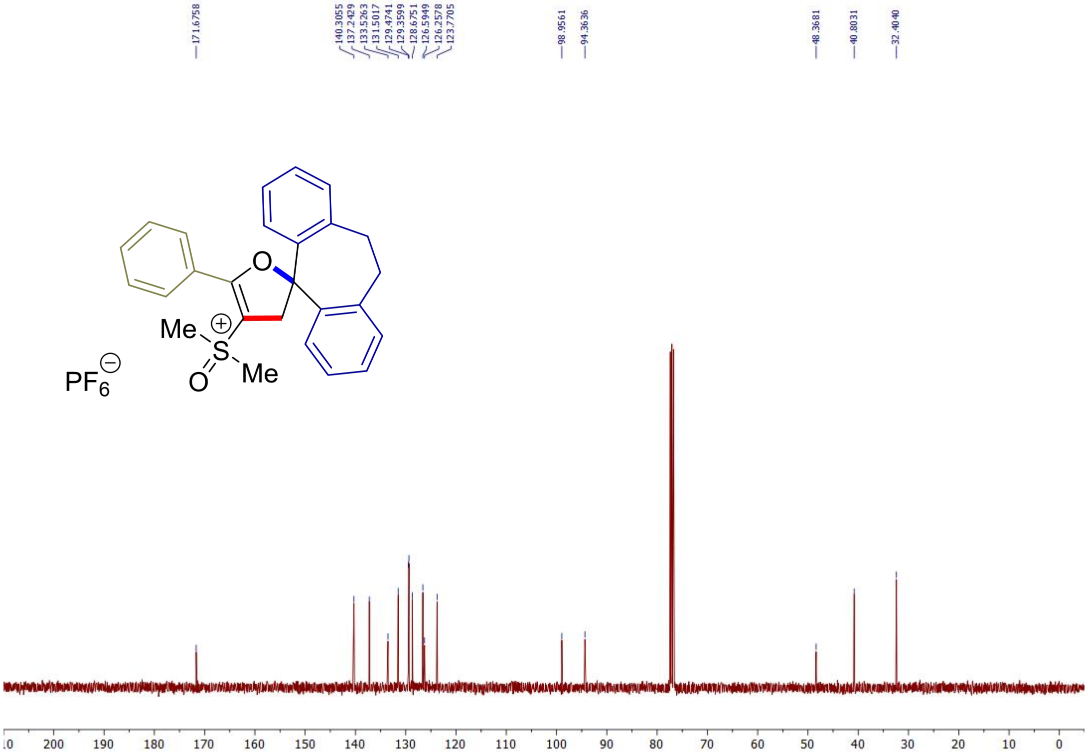

Supplementary Figure 207. ^{13}C NMR of the **7n** (101 MHz, 25 °C in $CDCl_3$)

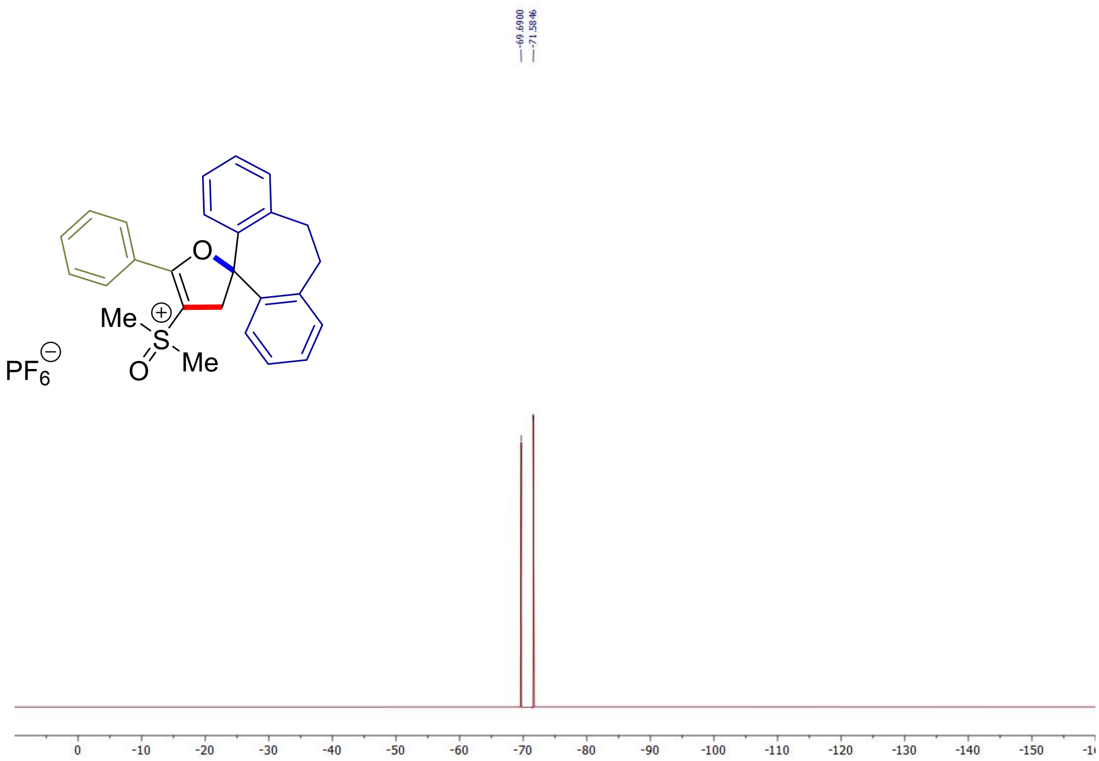

Supplementary Figure 208. **^{19}F NMR** of the **7n** (376 MHz, 25 °C in $CDCl_3$)

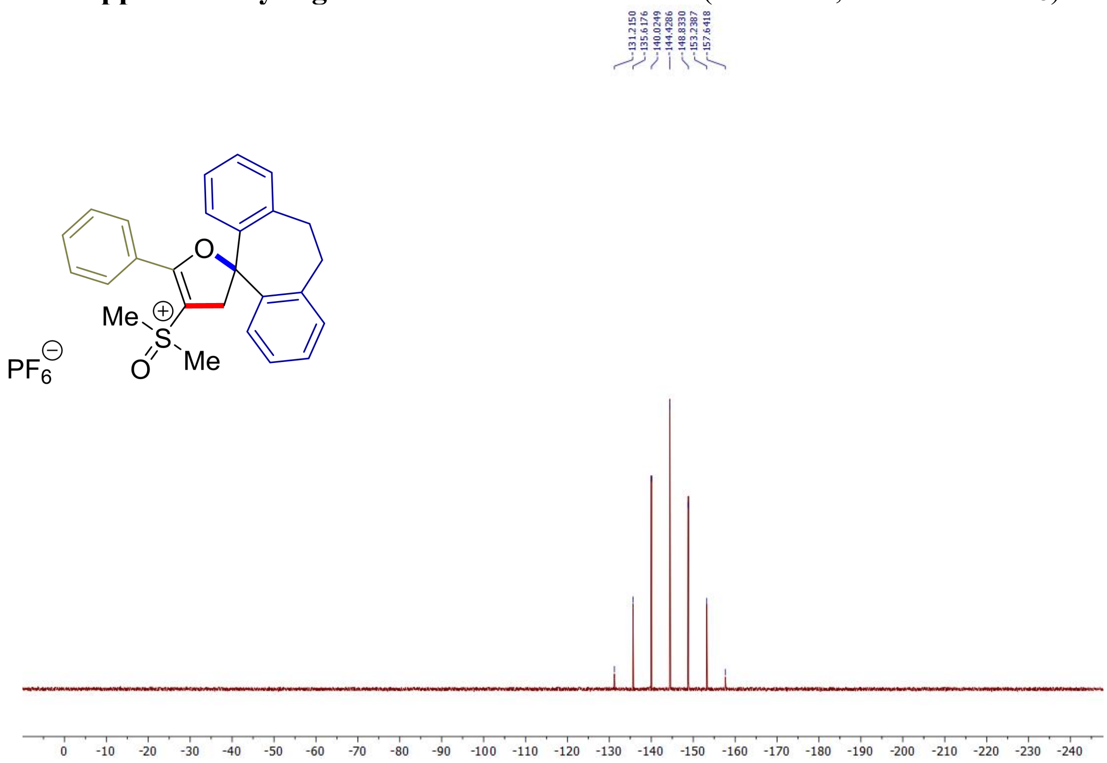

Supplementary Figure 209. **^{31}P NMR** of the **7n** (162 MHz, 25 °C in $CDCl_3$)

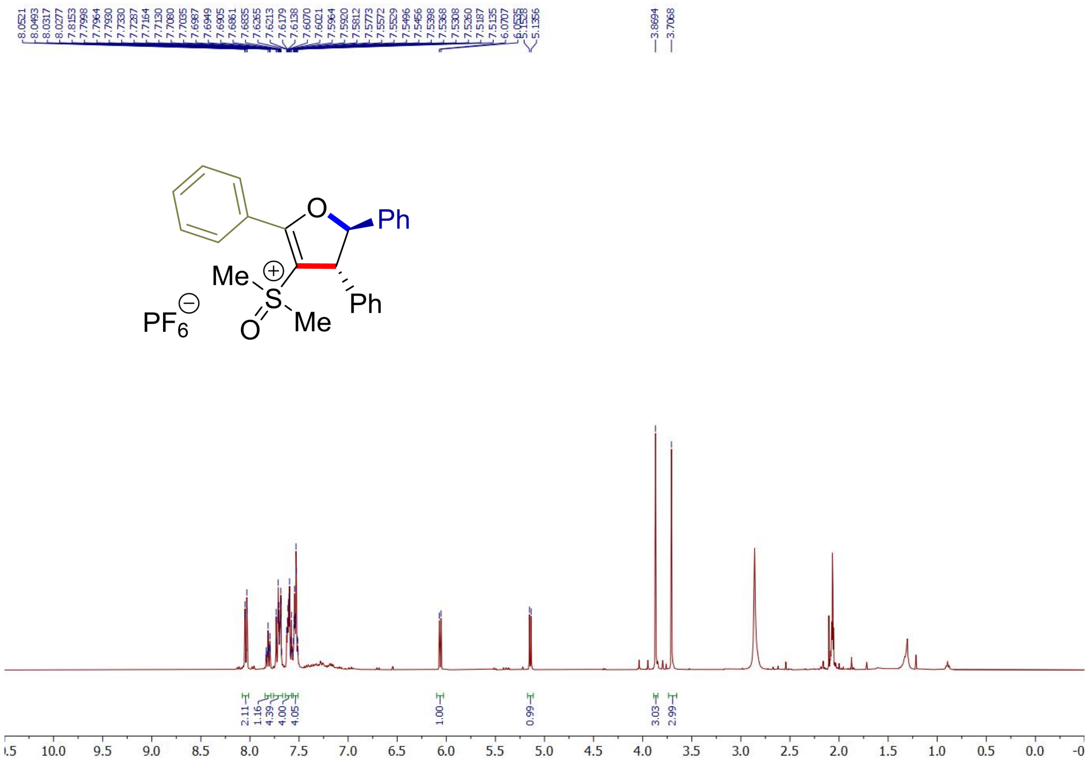

Supplementary Figure 210. ^{1}**H NMR** of the **7o** (400 MHz, 25 °C in Acetone-d_6)

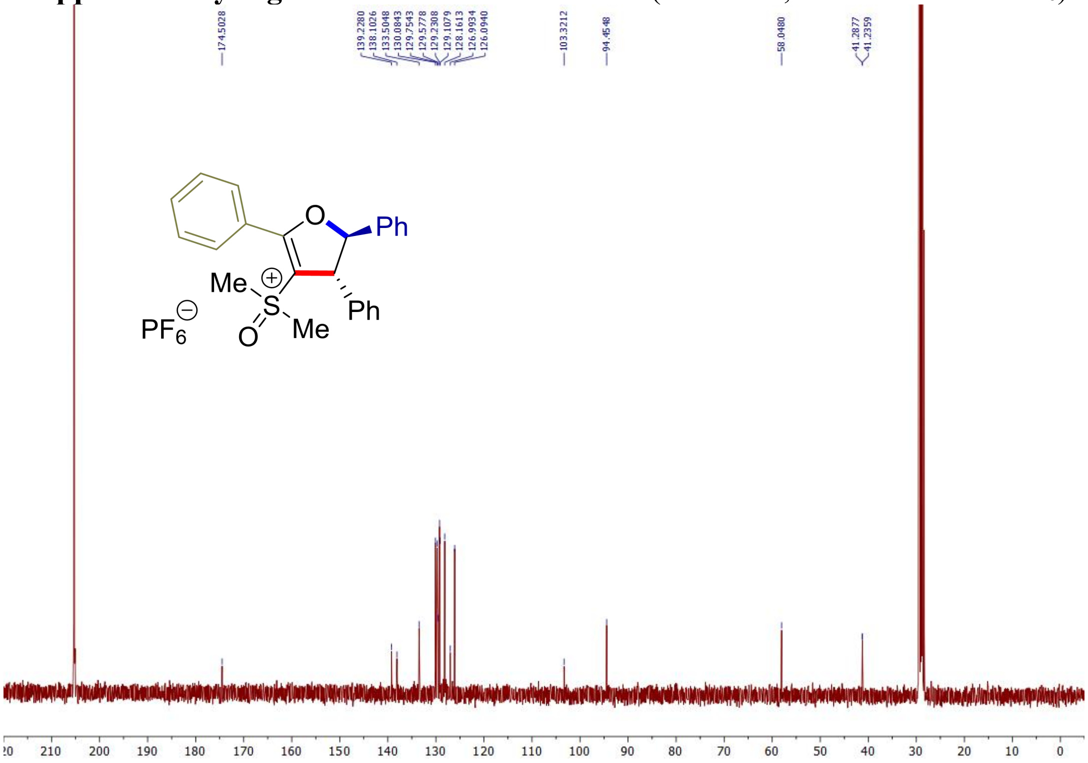

Supplementary Figure 211. ^{13}**C NMR** of the **7o** (101 MHz, 25 °C in Acetone-d_6)

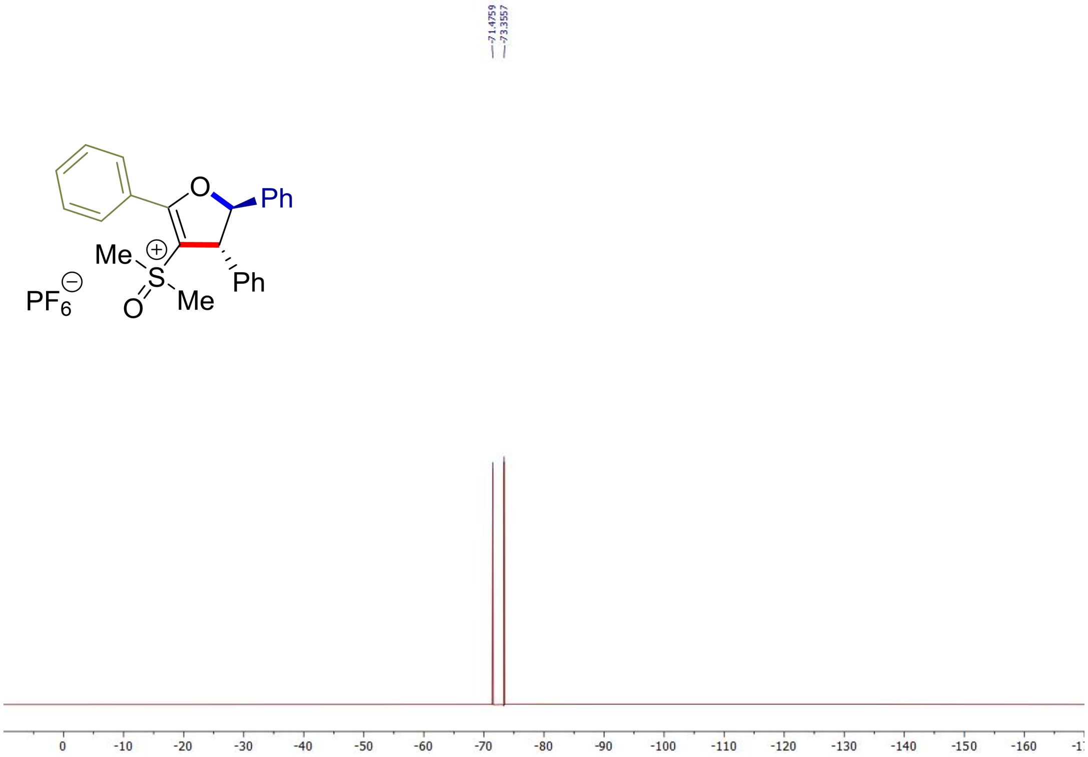

Supplementary Figure 212. ^{19}**F NMR** of the **7o** (376 MHz, 25 °C in Acetone-d_6)

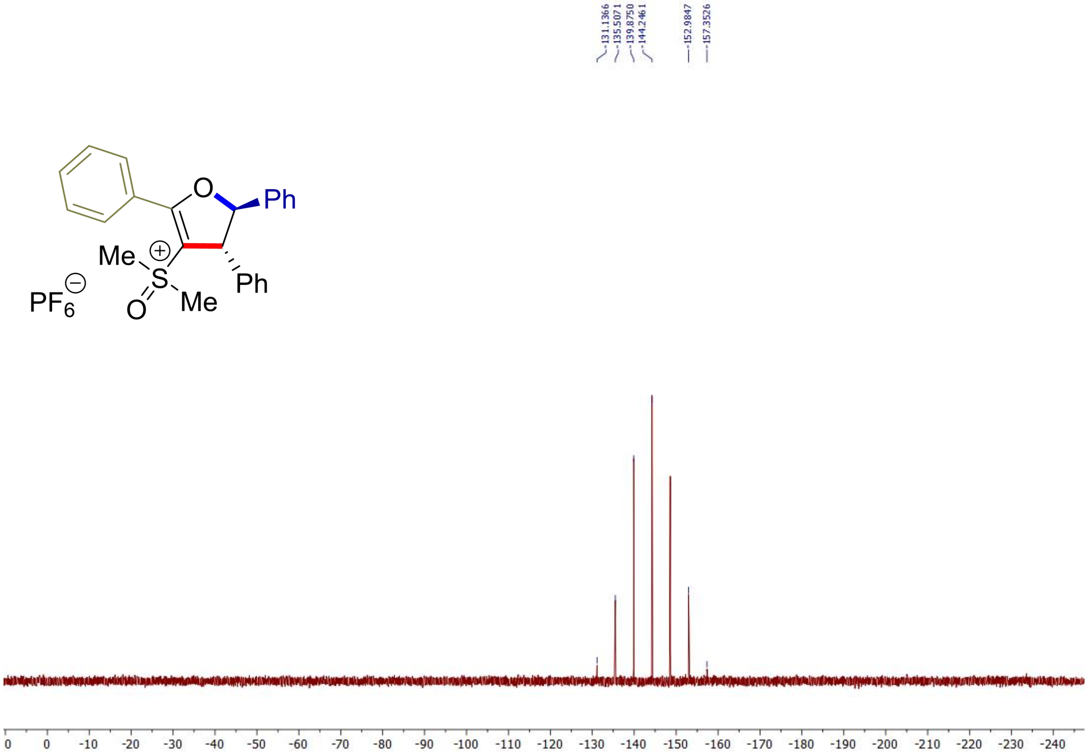

Supplementary Figure 213. ^{31}**P NMR** of the **7o** (162 MHz, 25 °C in Acetone-d_6)

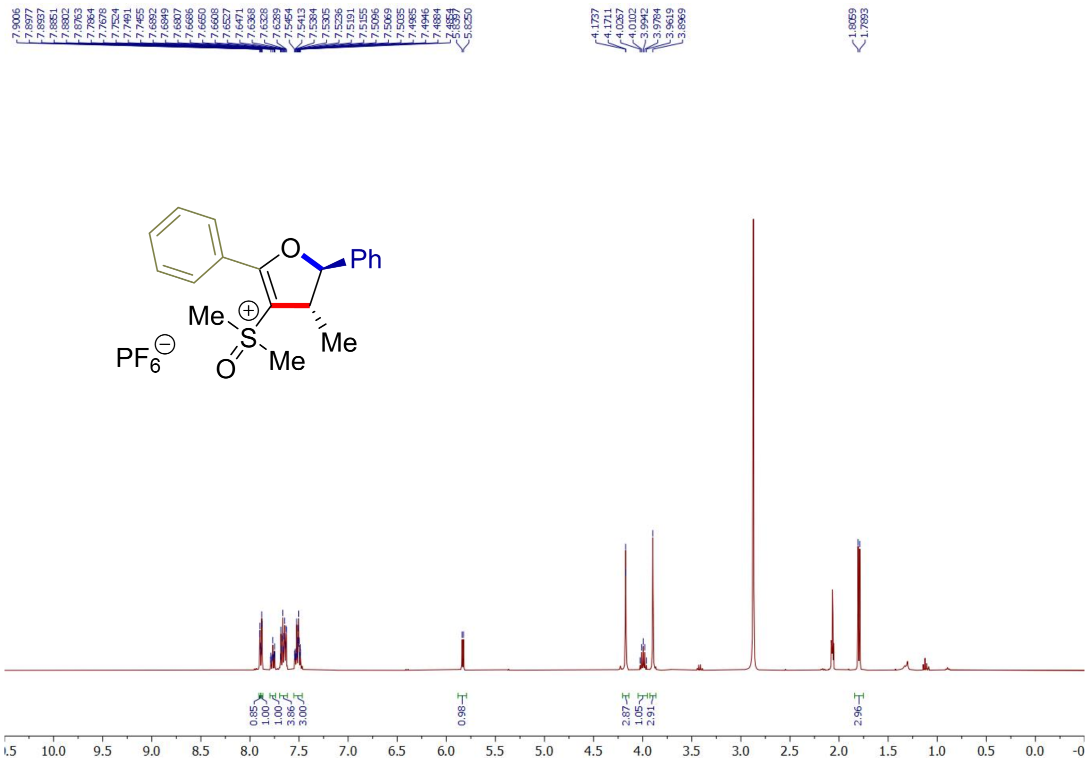

Supplementary Figure 214. **^{1}H NMR** of the **7p** (400 MHz, 25 °C in Acetone-d_6)

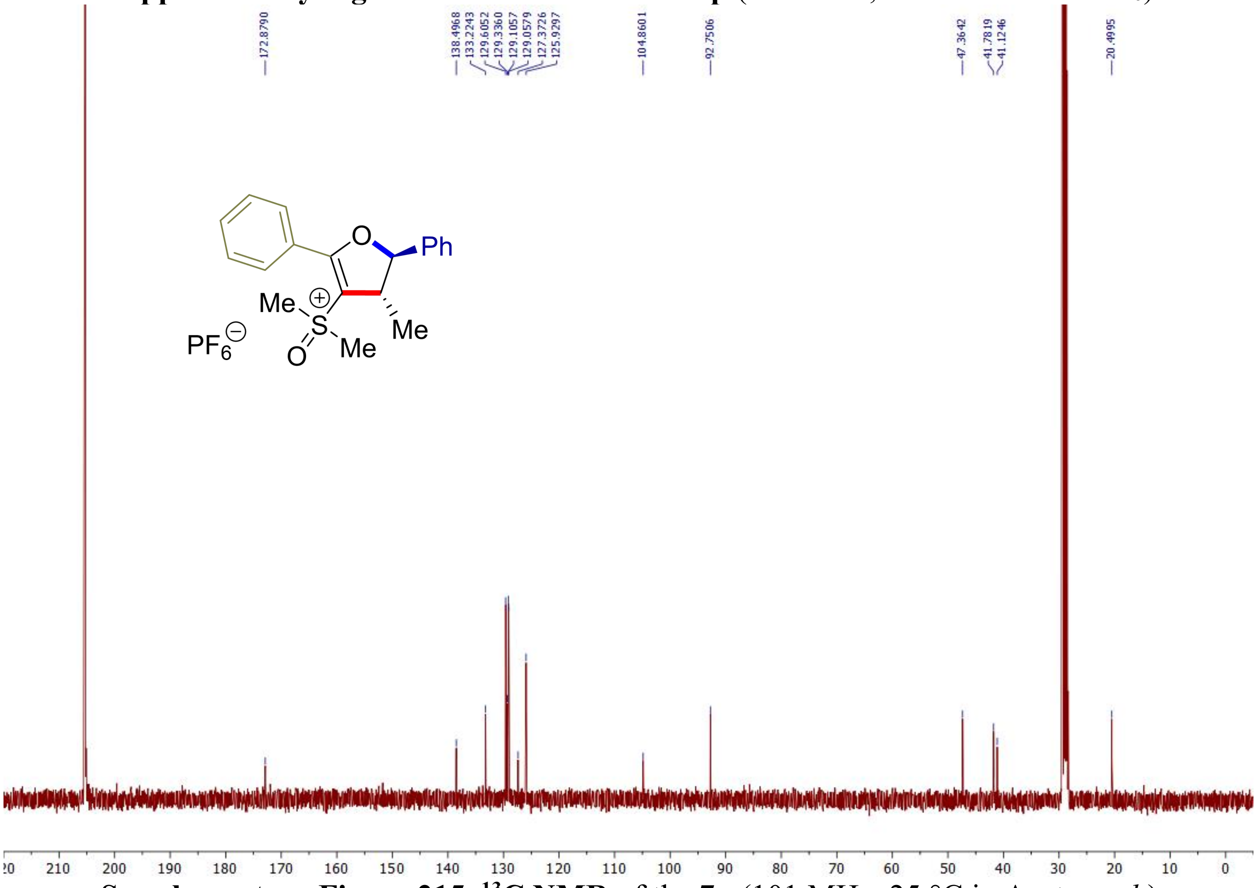

Supplementary Figure 215. **^{13}C NMR** of the **7p** (101 MHz, 25 °C in Acetone-d_6)

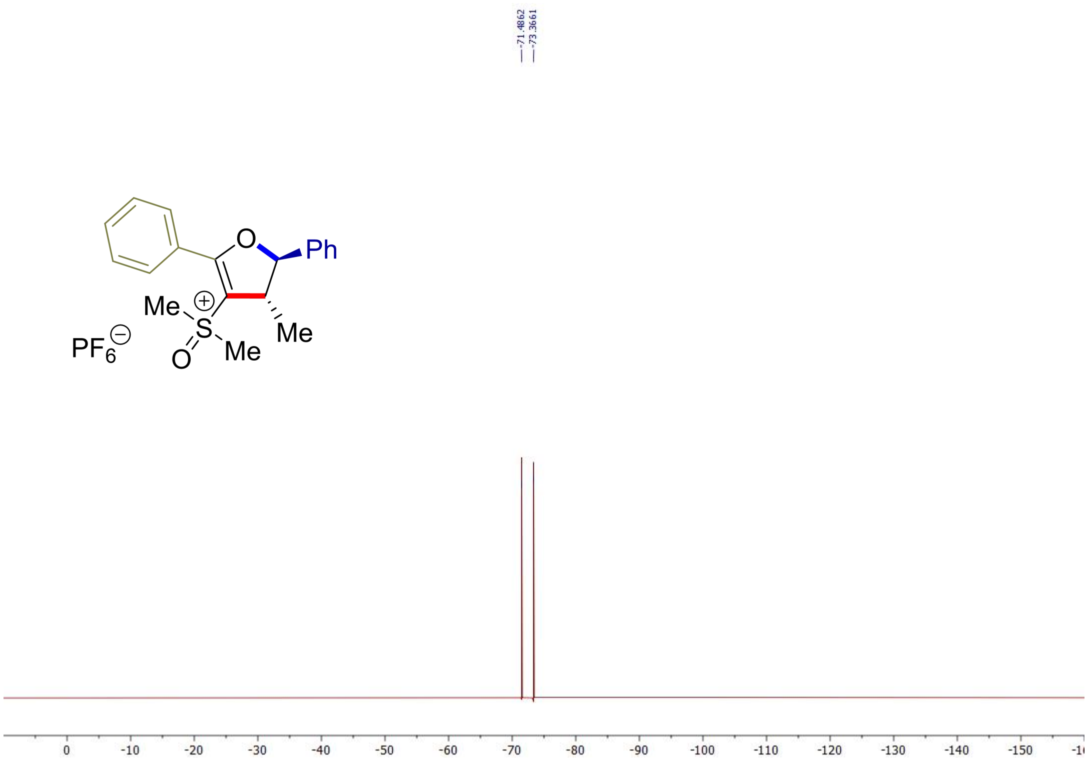

Supplementary Figure 216. ^{19}**F NMR** of the **7p** (376 MHz, 25 °C in Acetone-d_6)

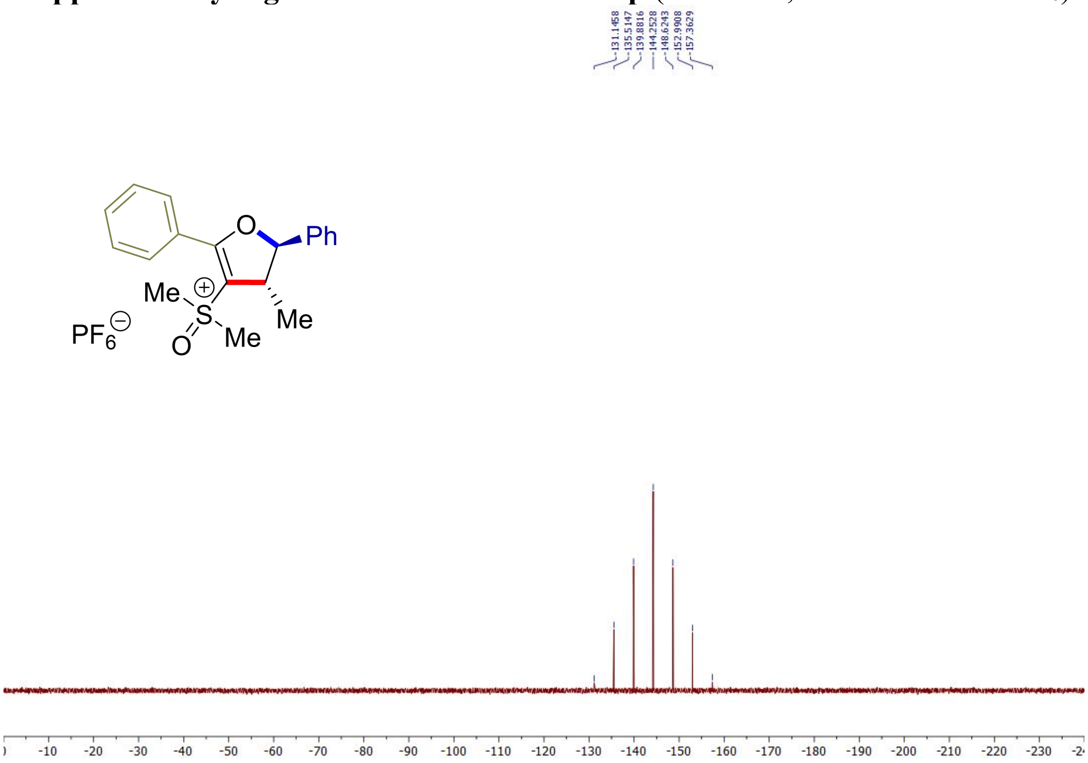

Supplementary Figure 217. ^{31}**P NMR** of the **7p** (162 MHz, 25 °C in Acetone-d_6)

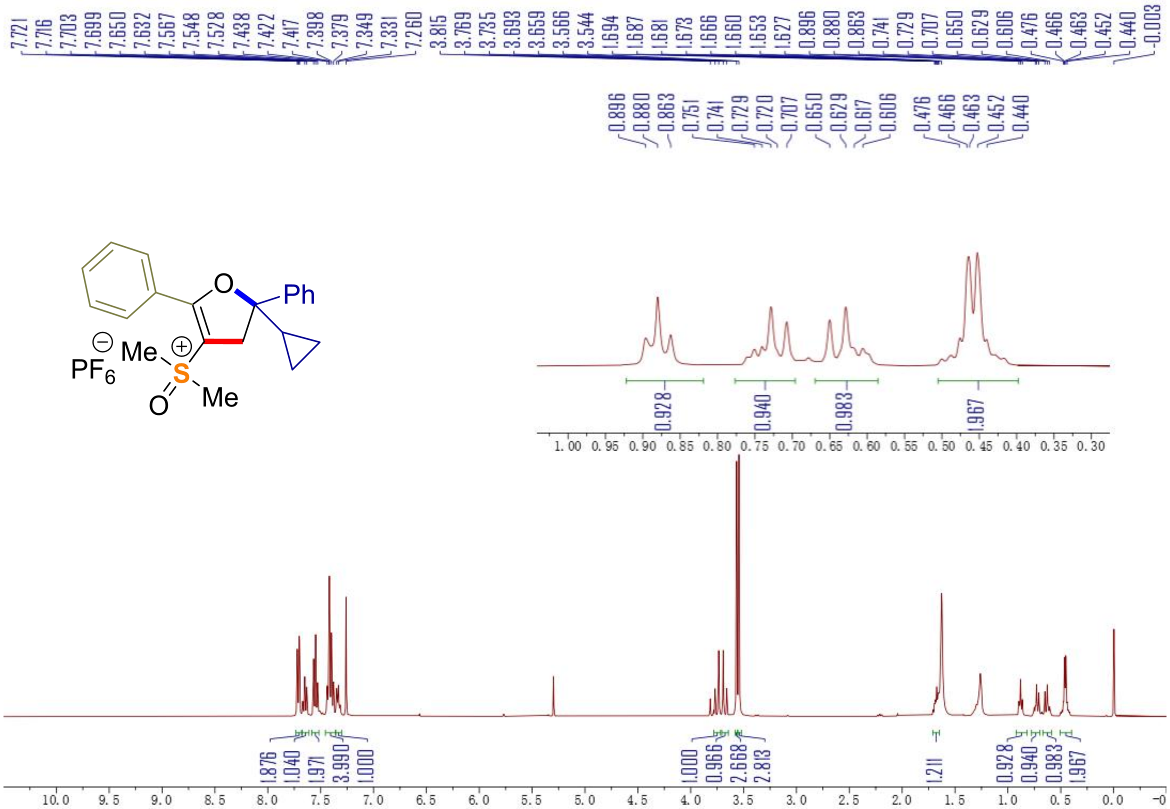

Supplementary Figure 218. ^{1}H NMR of the **7q** (400 MHz, 25 °C in $CDCl_3$)

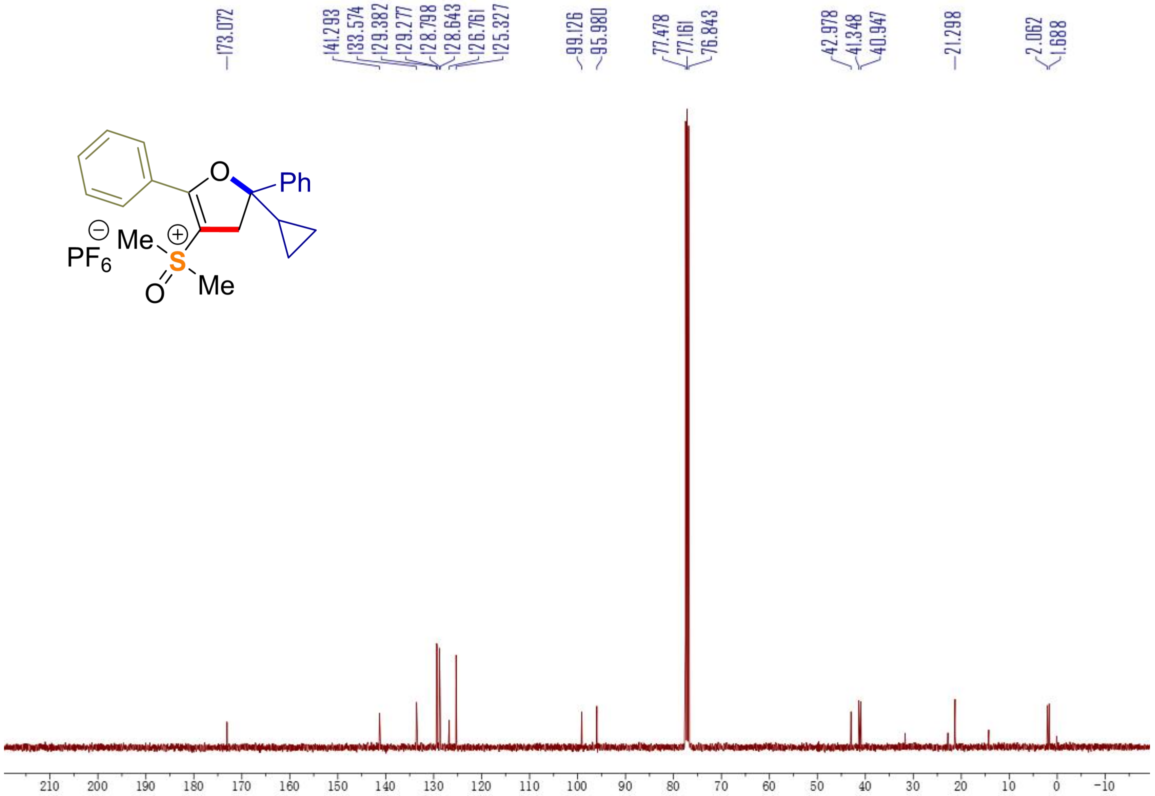

Supplementary Figure 219. ^{13}C-NMR of the **7q** (101 MHz, 25 °C in $CDCl_3$)

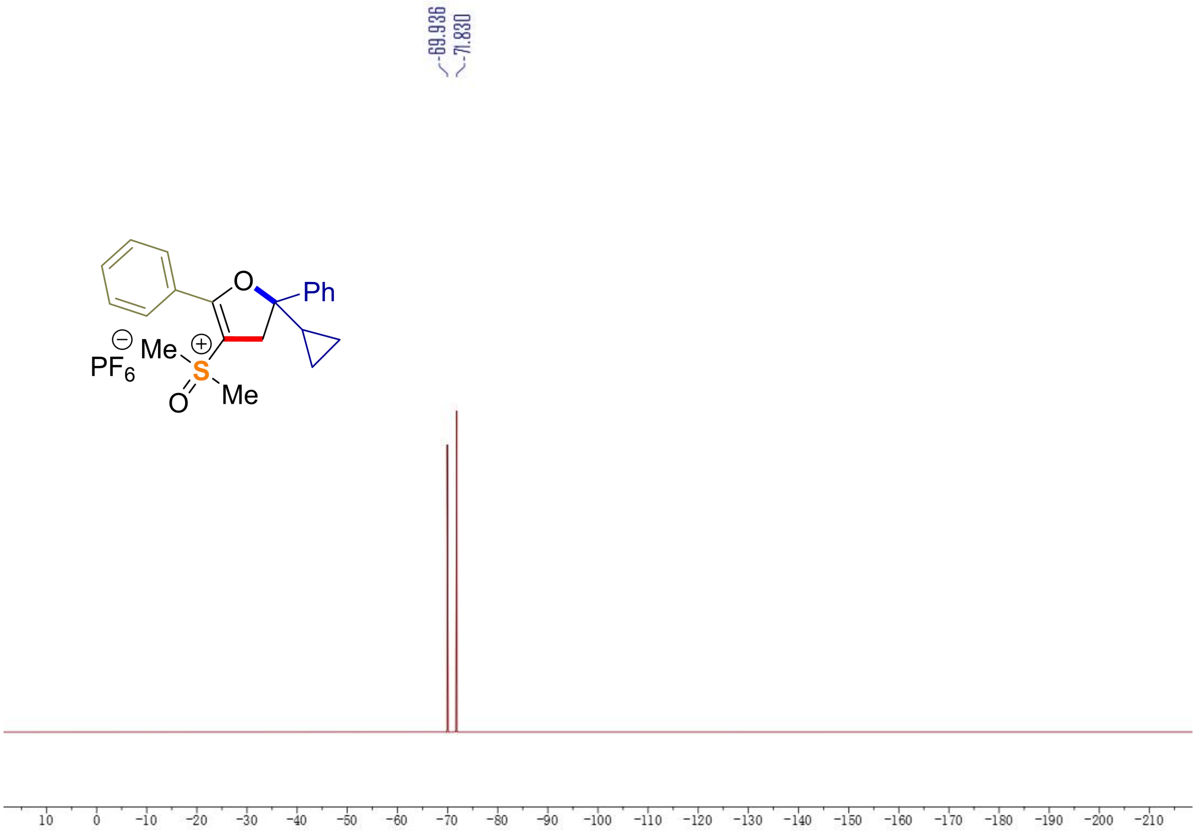

Supplementary Figure 220. ^{19}**F-NMR** of the **7q** (376 MHz, 25 °C in $CDCl_3$)

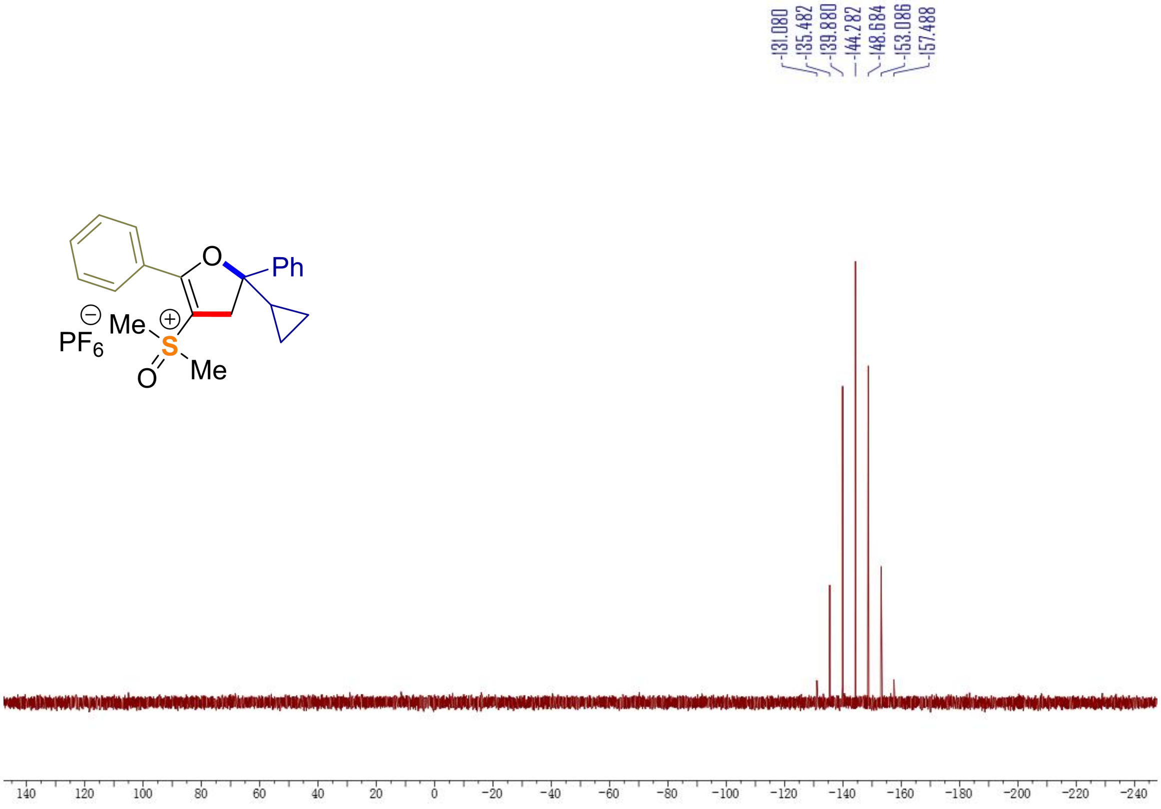

Supplementary Figure 221. ^{31}**P-NMR** of the **7q** (162 MHz, 25 °C in $CDCl_3$)

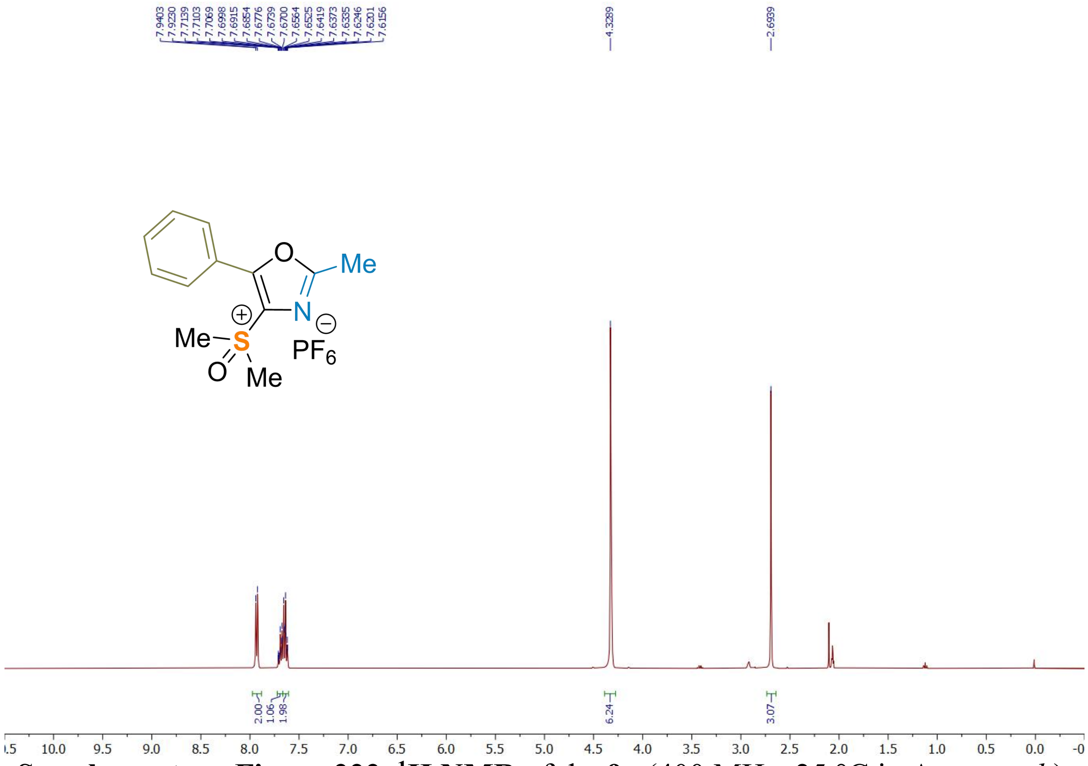

Supplementary Figure 222. ^{1}H NMR of the **9a** (400 MHz, 25 °C in Acetone-d_6)

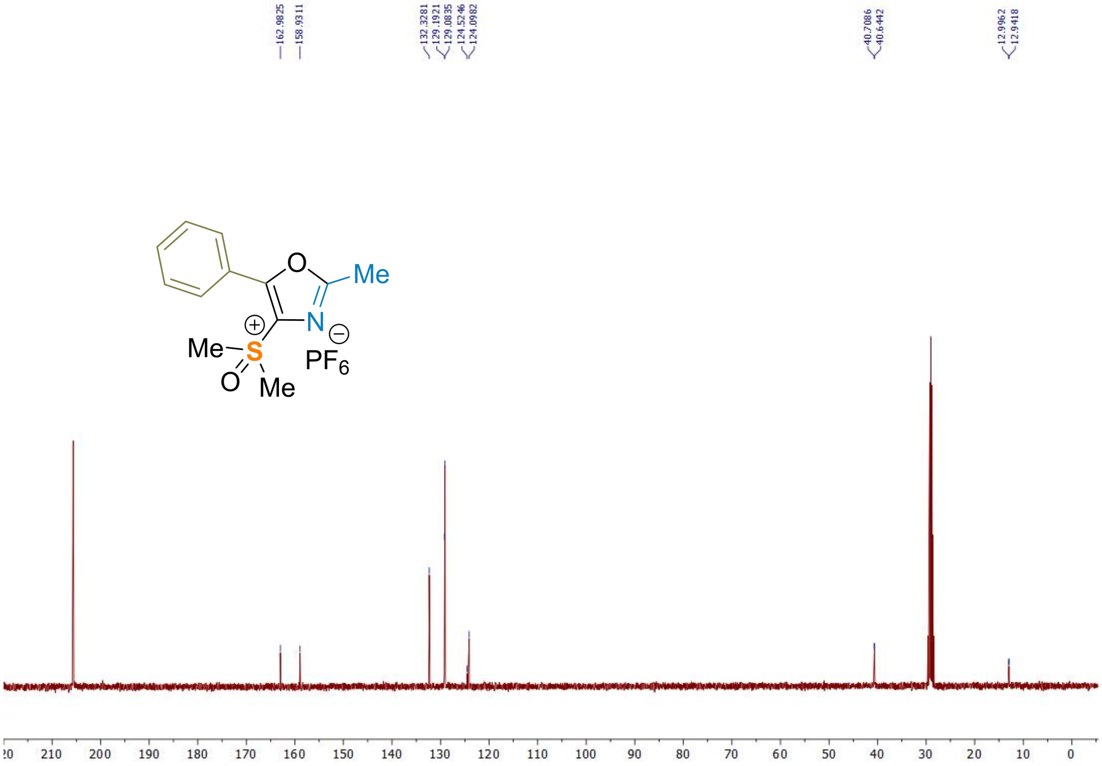

Supplementary Figure 223. ^{13}C NMR of the **9a** (101 MHz, 25 °C in Acetone-d_6)

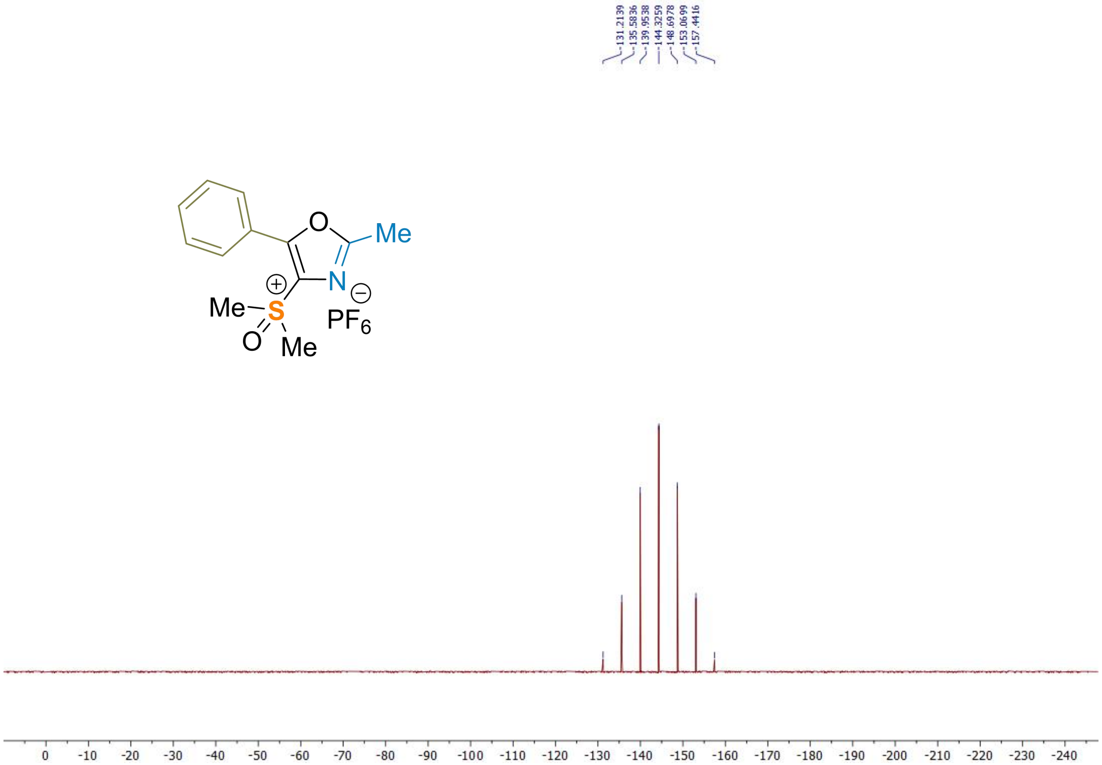

Supplementary Figure 224. ^{31}**P NMR** of the **9a** (162 MHz, 25 °C in Acetone-d_6)

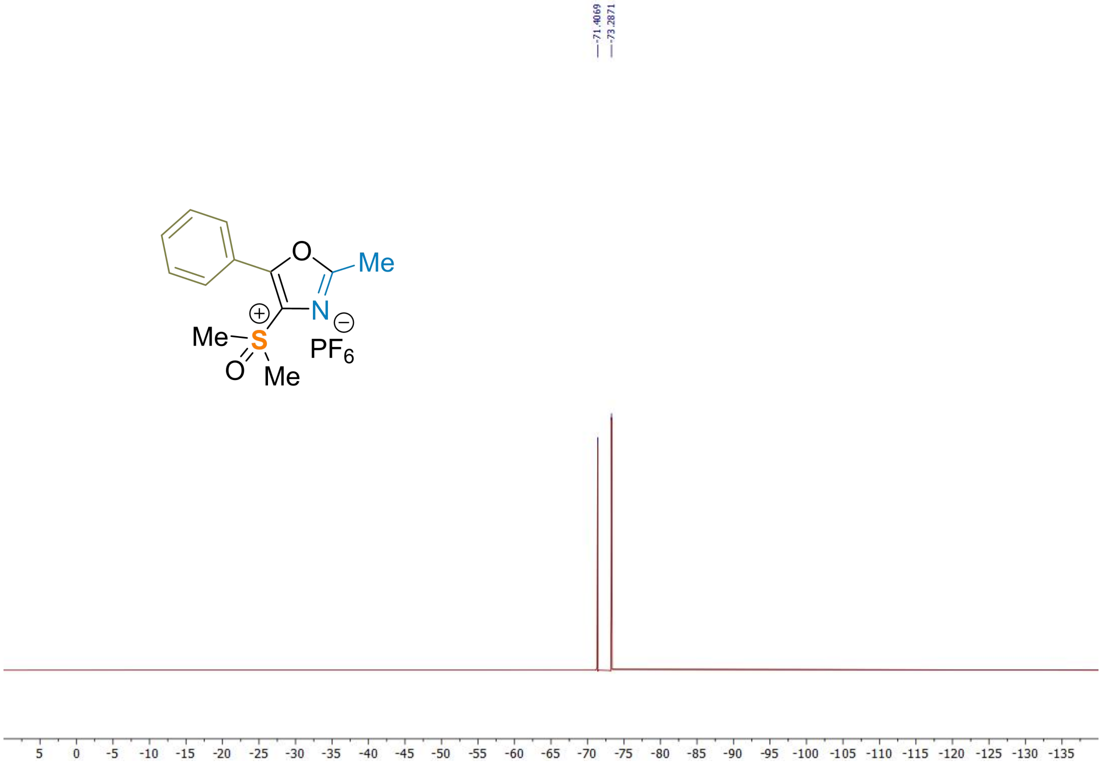

Supplementary Figure 225. ^{19}**F NMR** of the **9a** (376 MHz, 25 °C in Acetone-d_6)

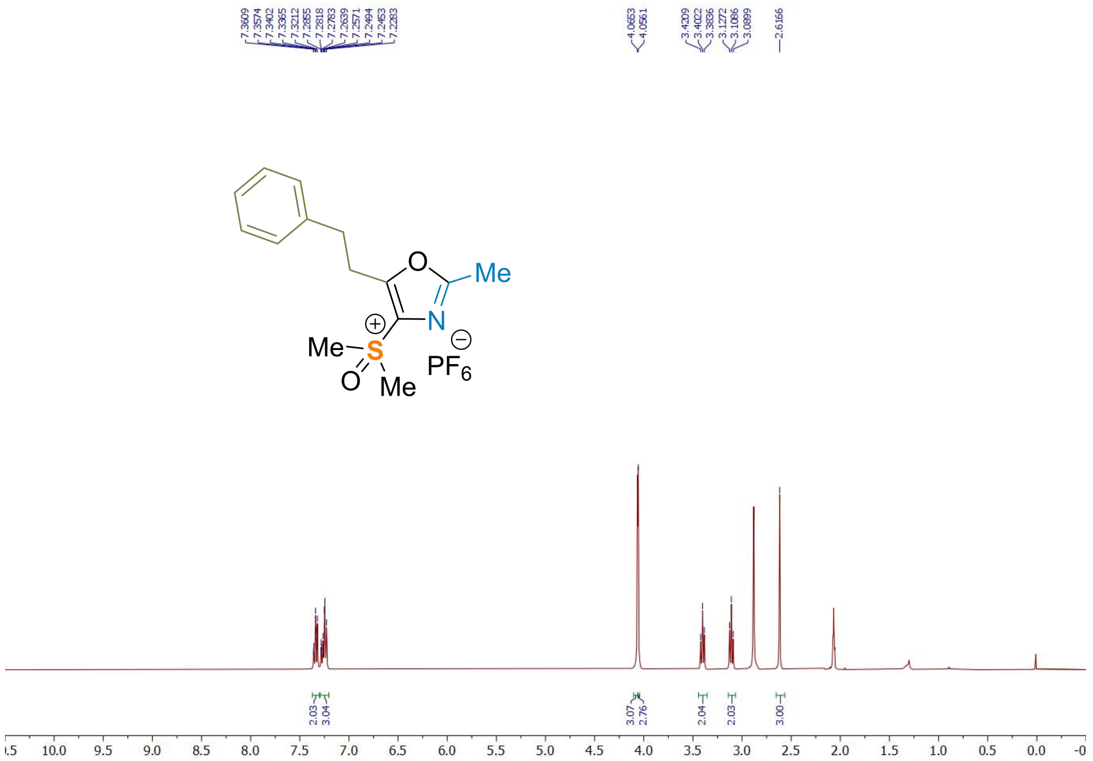

Supplementary Figure 226. **^{1}H NMR** of the **9b** (400 MHz, 25 °C in Acetone-d_6)

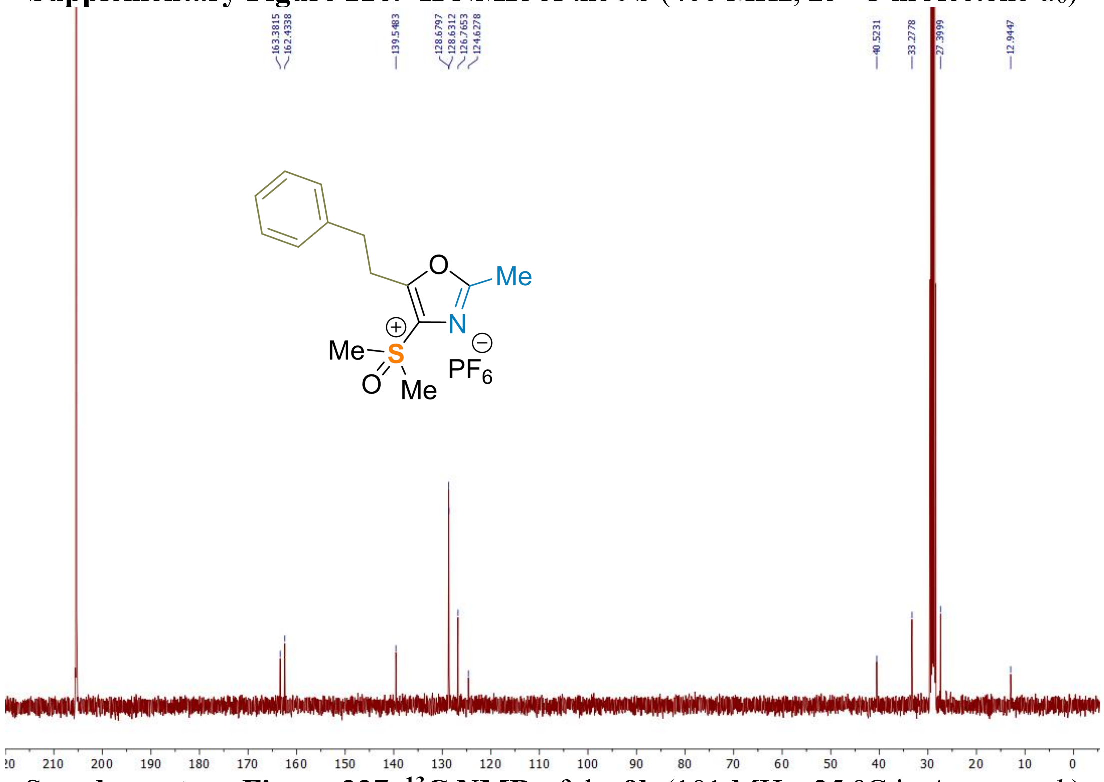

Supplementary Figure 227. **^{13}C NMR** of the **9b** (101 MHz, 25 °C in Acetone-d_6)

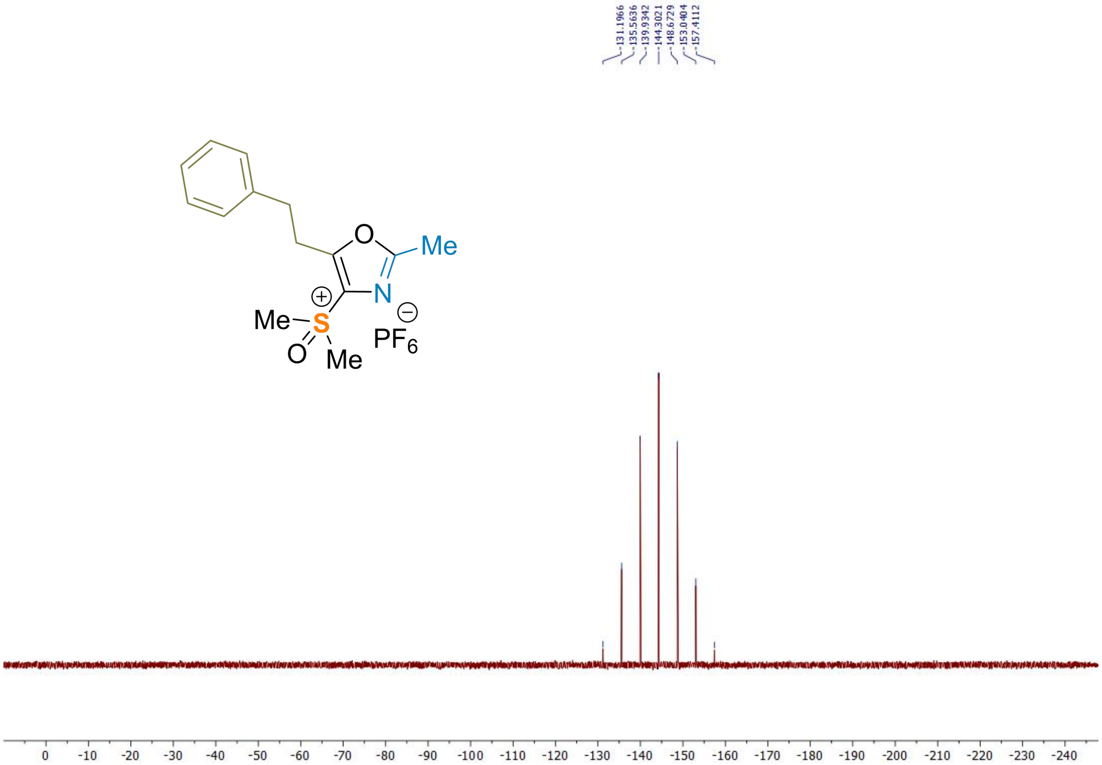

Supplementary Figure 228. ^{31}**P NMR** of the **9b** (162 MHz, 25 °C in Acetone-d_6)

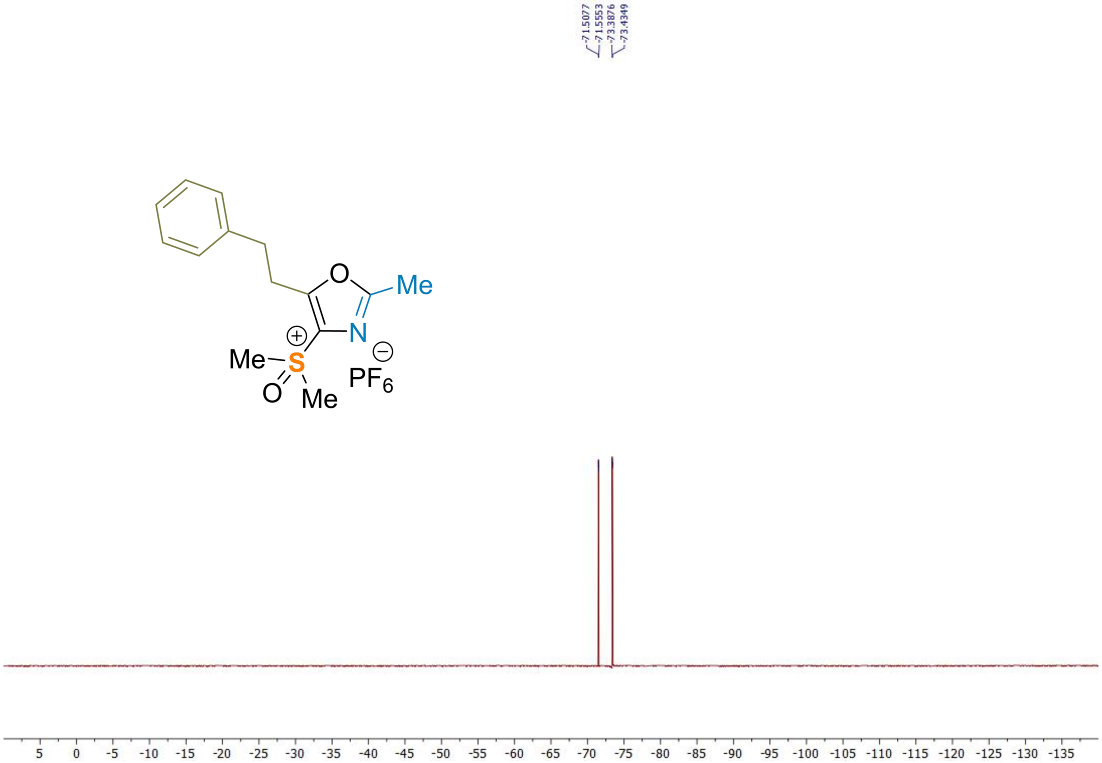

Supplementary Figure 229. ^{19}**F NMR** of the **9b** (376 MHz, 25 °C in Acetone-d_6)

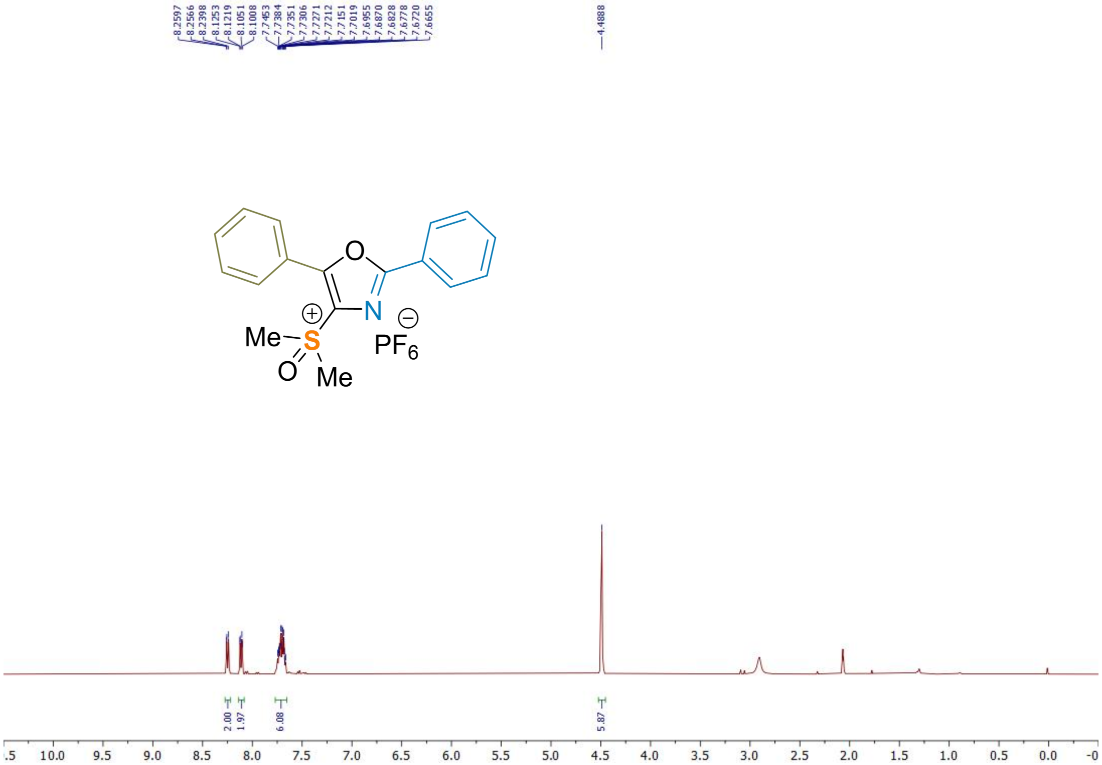

Supplementary Figure 230. **^{1}H NMR** of the **9c** (400 MHz, 25 °C in Acetone-d_6)

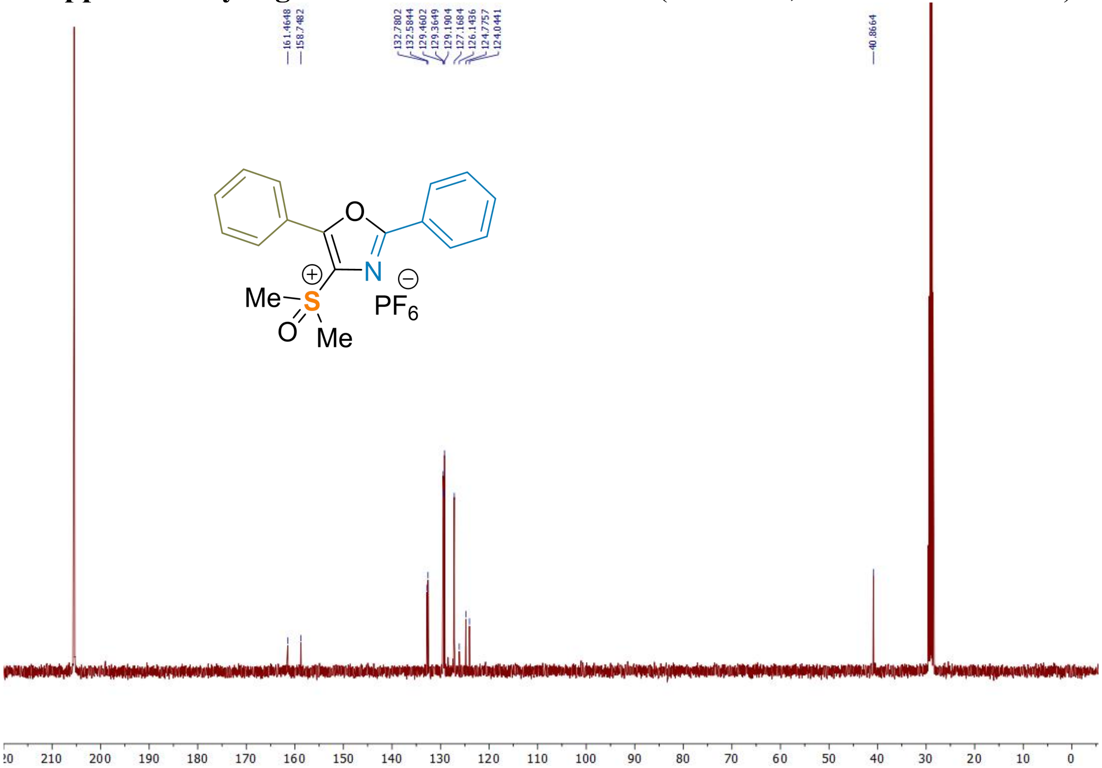

Supplementary Figure 231. ^{13}C NMR of the **9c** (101 MHz, 25 °C in Acetone-d_6)

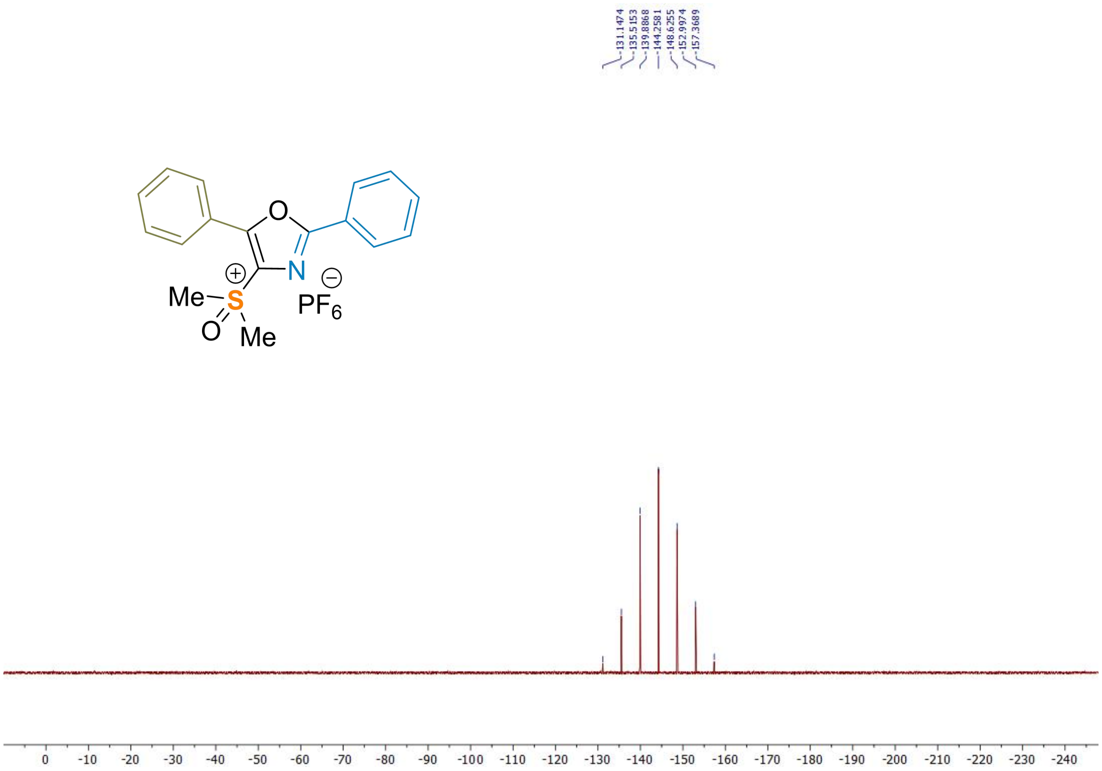

Supplementary Figure 232. ^{31}**P NMR** of the **9c** (162 MHz, 25 °C in Acetone-d_6)

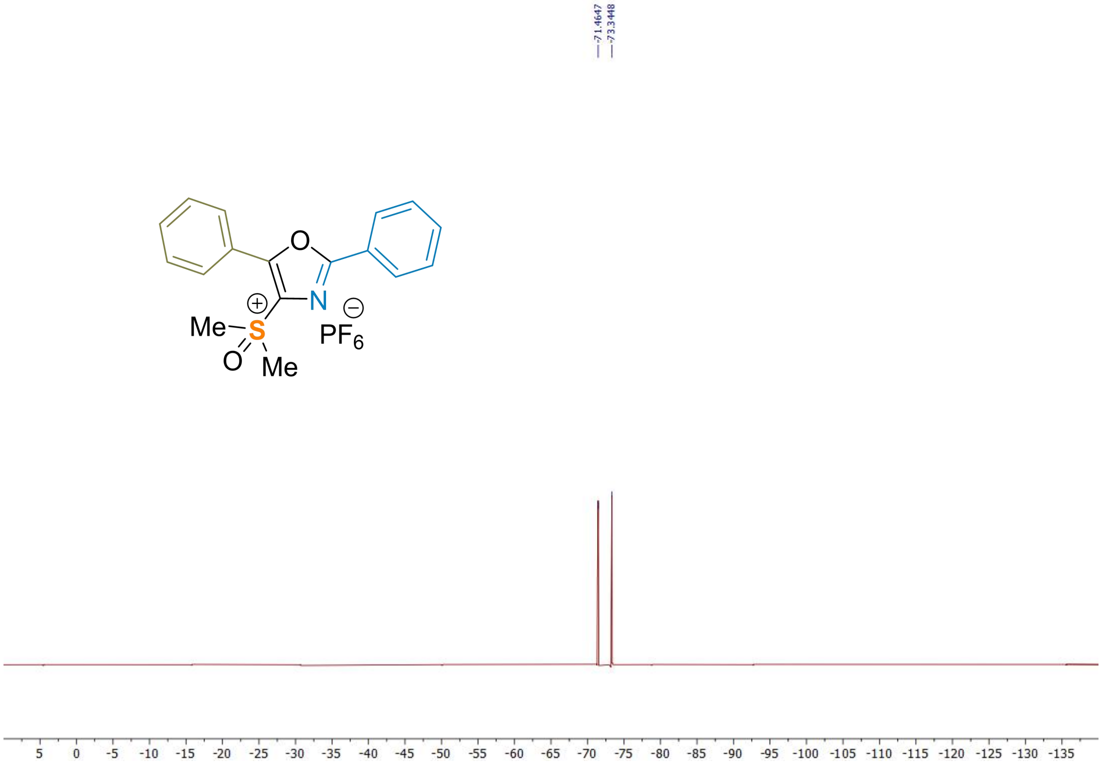

Supplementary Figure 233. ^{19}**F NMR** of the **9c** (376 MHz, 25 °C in Acetone-d_6)

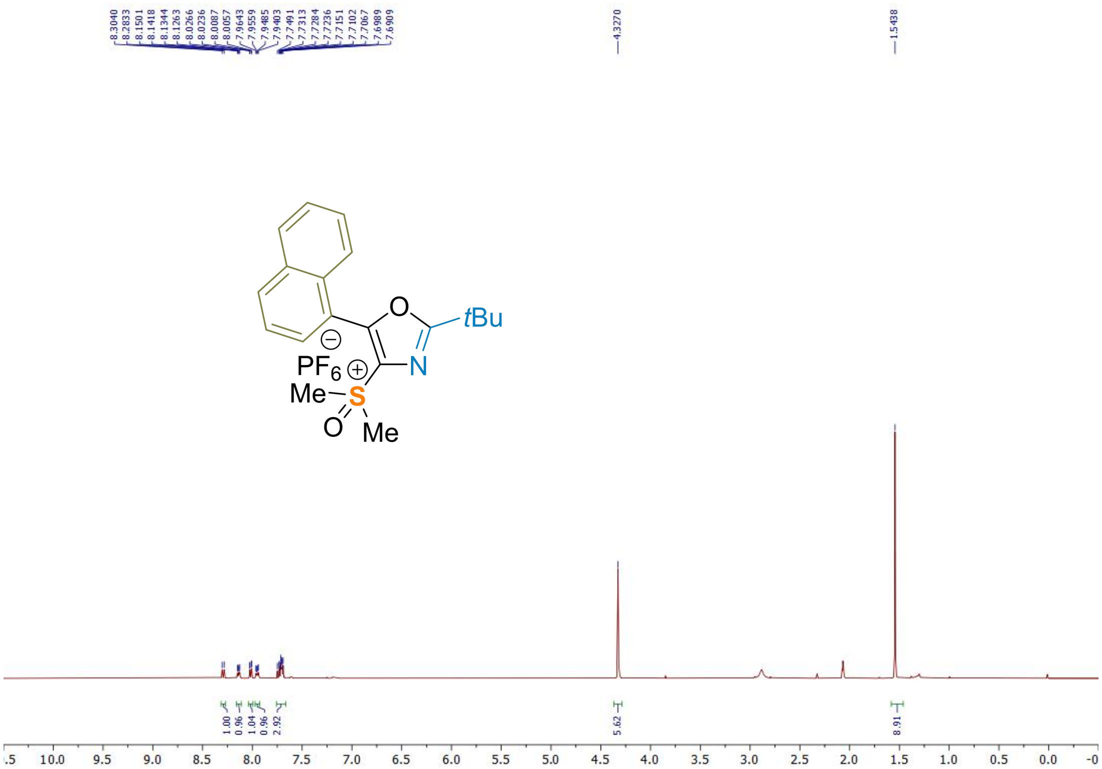

Supplementary Figure 234. **^{1}H NMR** of the **9d** (400 MHz, 25 °C in Acetone-d_6)

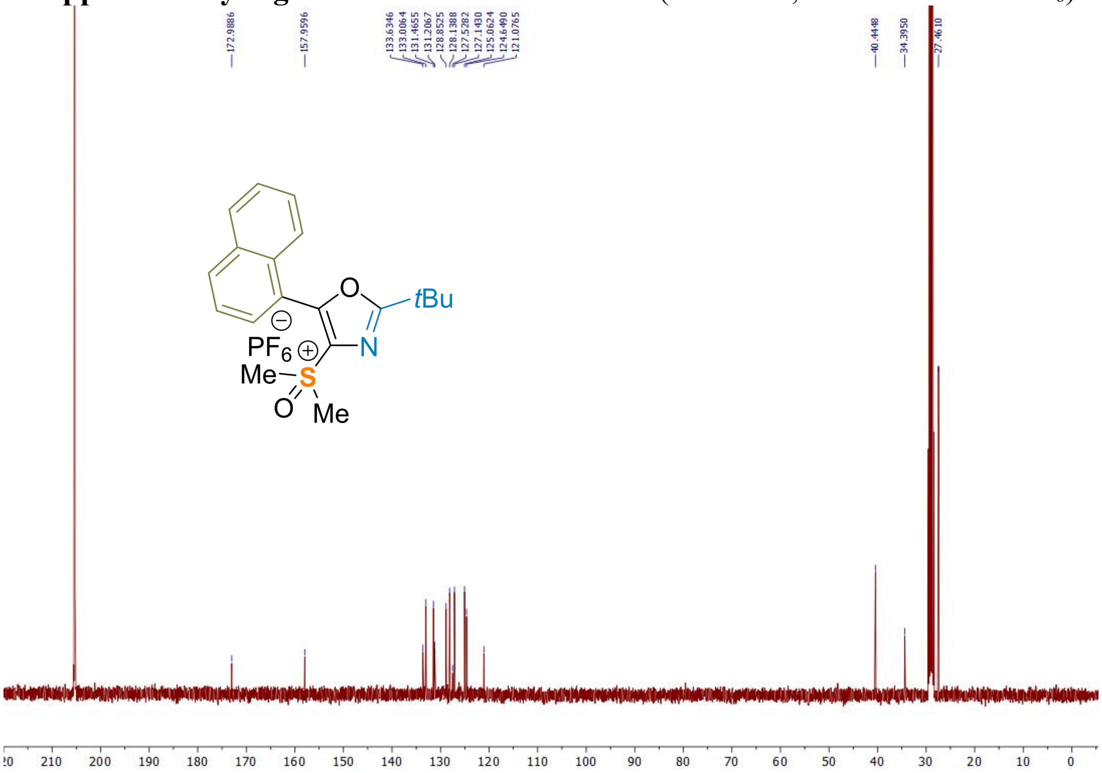

Supplementary Figure 235. ^{13}C NMR of the **9d** (101 MHz, 25 °C in Acetone-d_6)

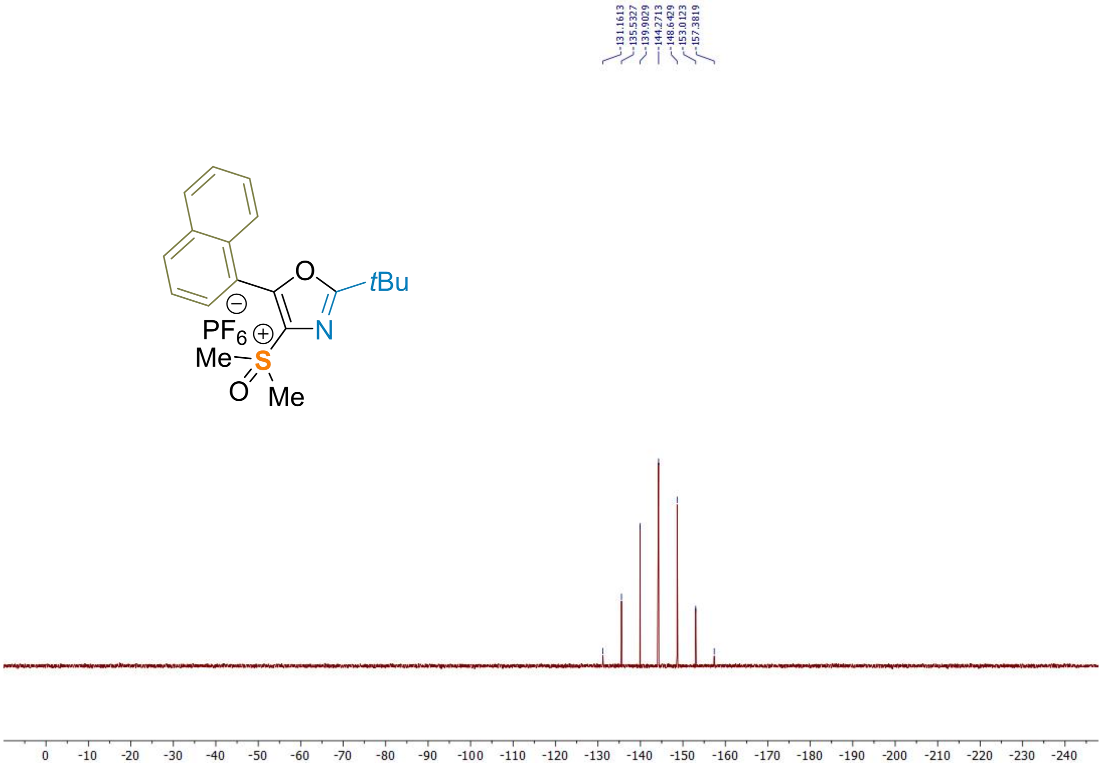

Supplementary Figure 236. ^{31}**P NMR** of the **9d** (162 MHz, 25 °C in Acetone-d_6)

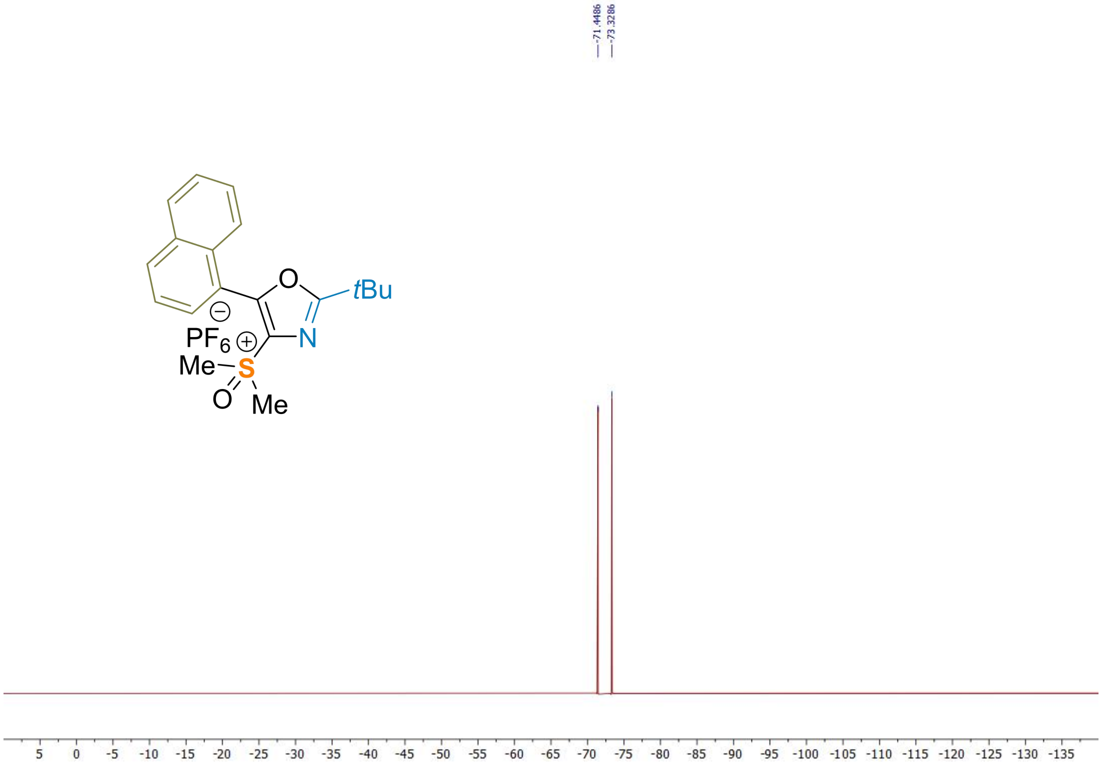

Supplementary Figure 237. ^{19}**F NMR** of the **9d** (376 MHz, 25 °C in Acetone-d_6)

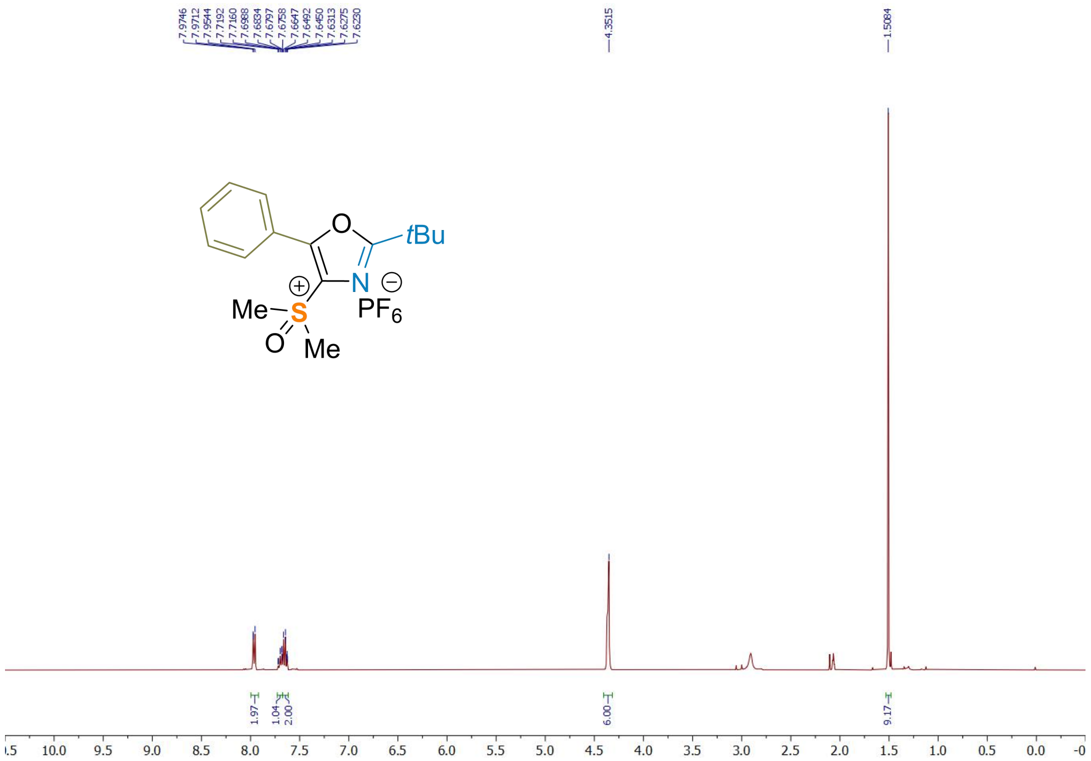

Supplementary Figure 238. ^{1}H NMR of the **9e** (400 MHz, 25 °C in Acetone-d_6)

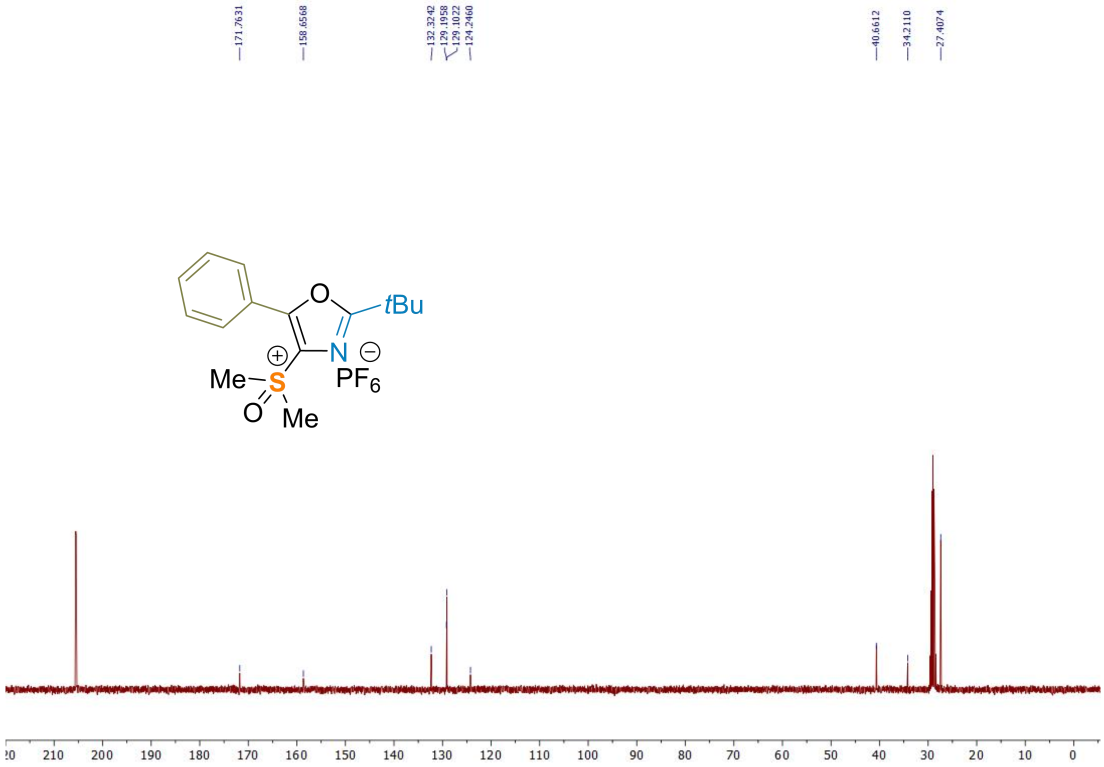

Supplementary Figure 239. ^{13}C NMR of the **9e** (101 MHz, 25 °C in Acetone-d_6)

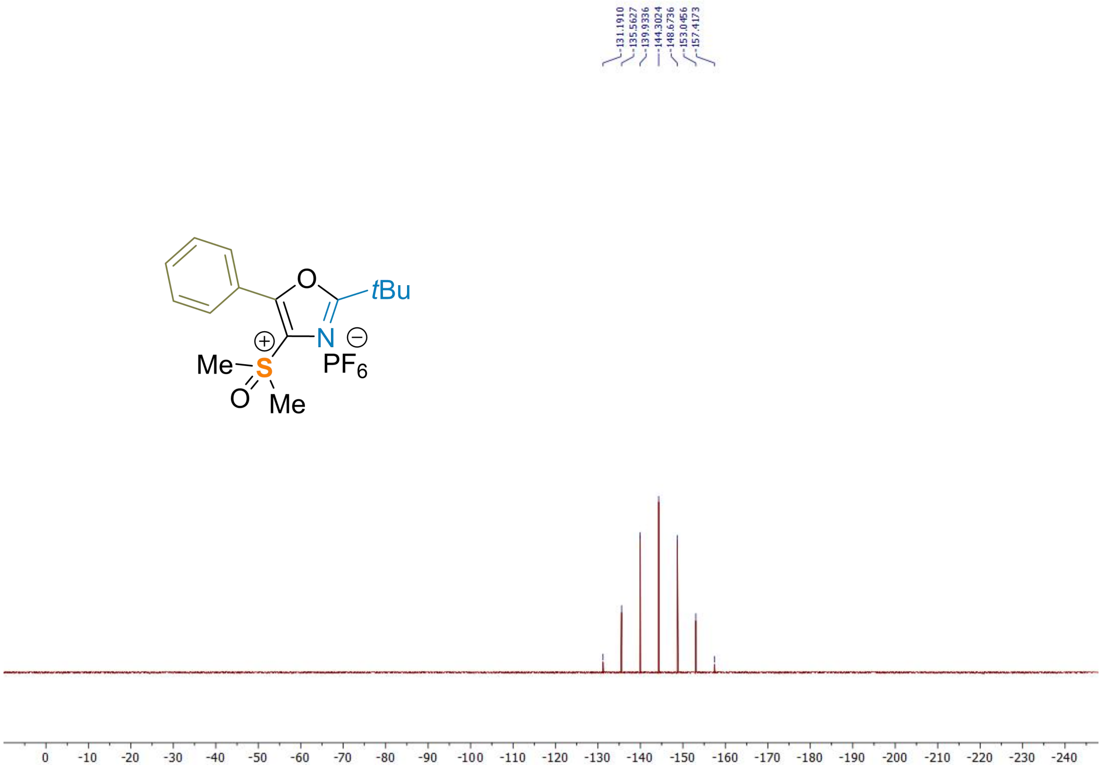

Supplementary Figure 240. ^{31}**P NMR** of the **9e** (162 MHz, 25 °C in Acetone-d_6)

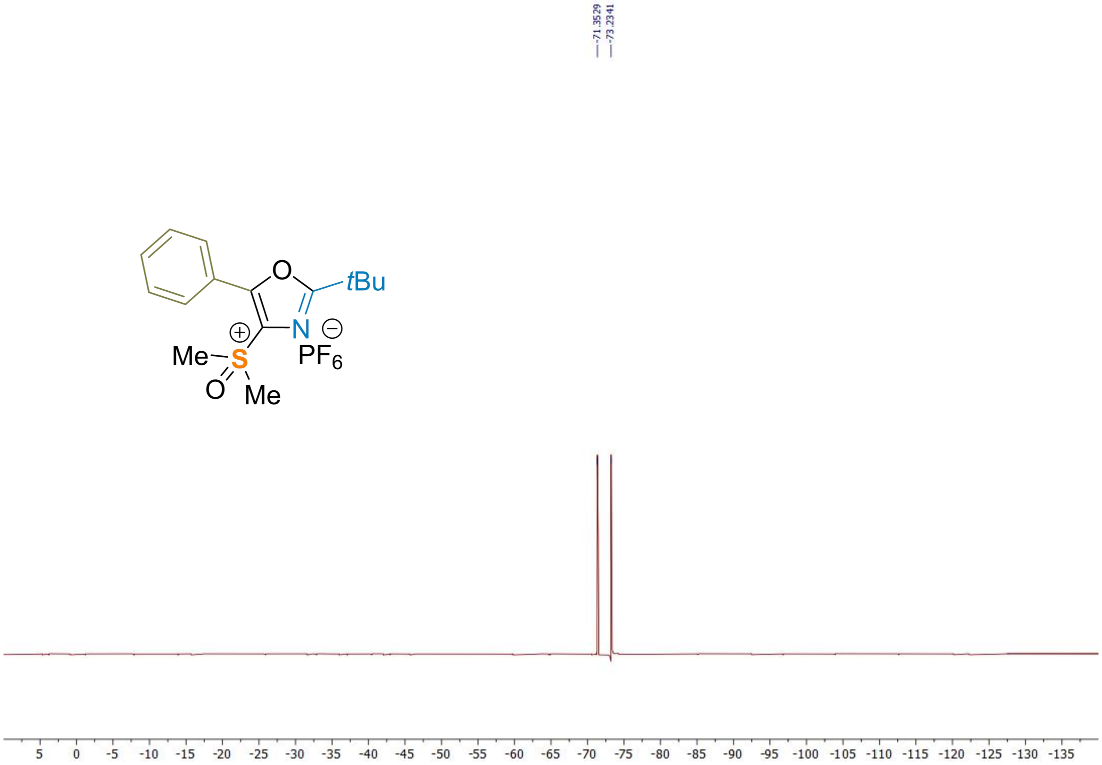

Supplementary Figure 241. ^{19}**F NMR** of the **9e** (376 MHz, 25 °C in Acetone-d_6)

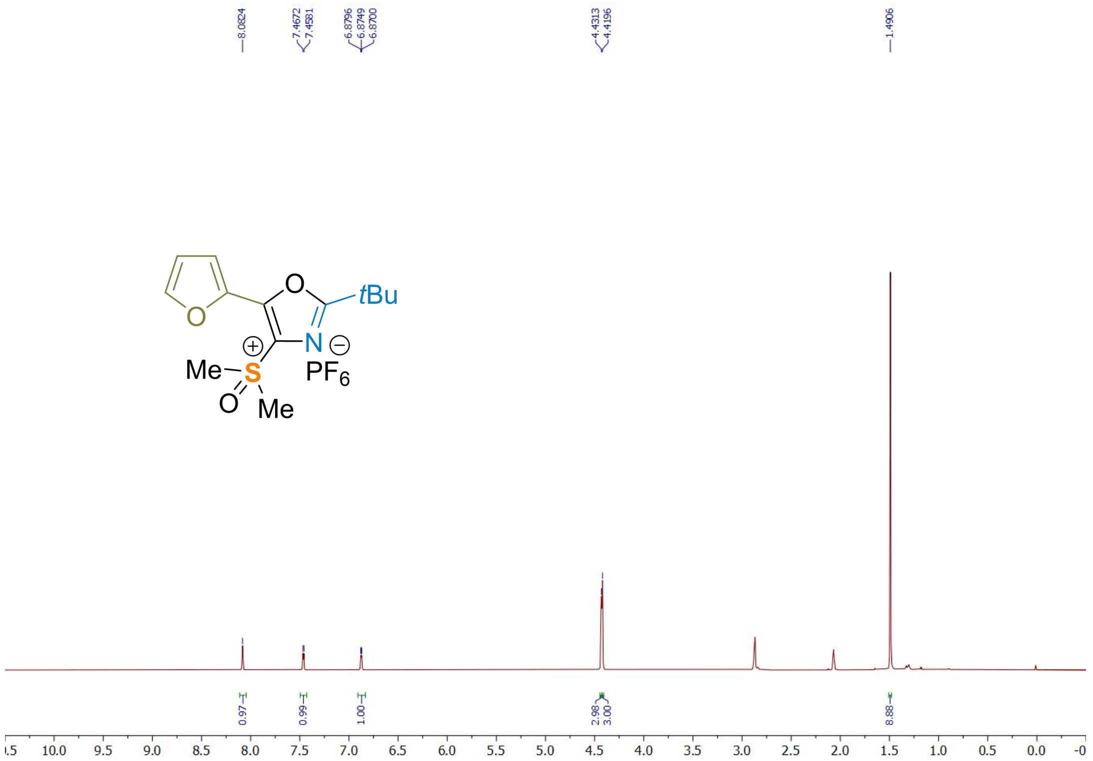

Supplementary Figure 242. ^{1}**H NMR** of the **9f** (400 MHz, 25 °C in Acetone-d_6)

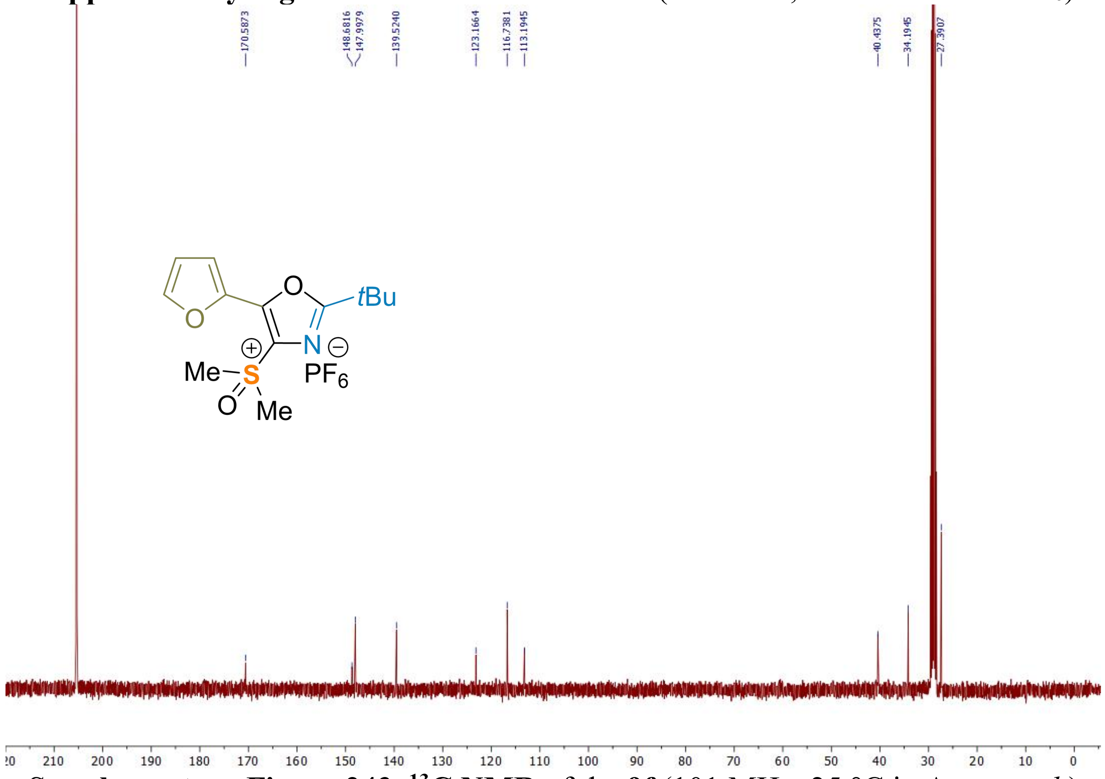

Supplementary Figure 243. ^{13}**C NMR** of the **9f** (101 MHz, 25 °C in Acetone-d_6)

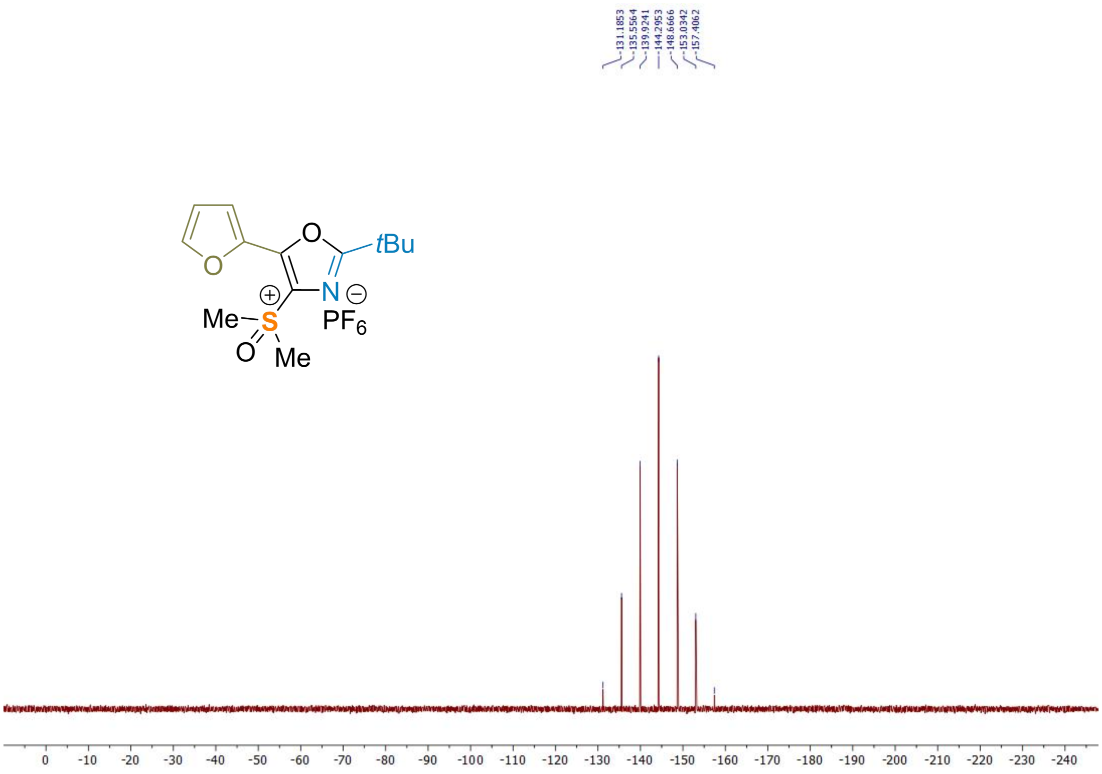

Supplementary Figure 244. ^{31}**P NMR** of the **9f** (162 MHz, 25 °C in Acetone-d_6)

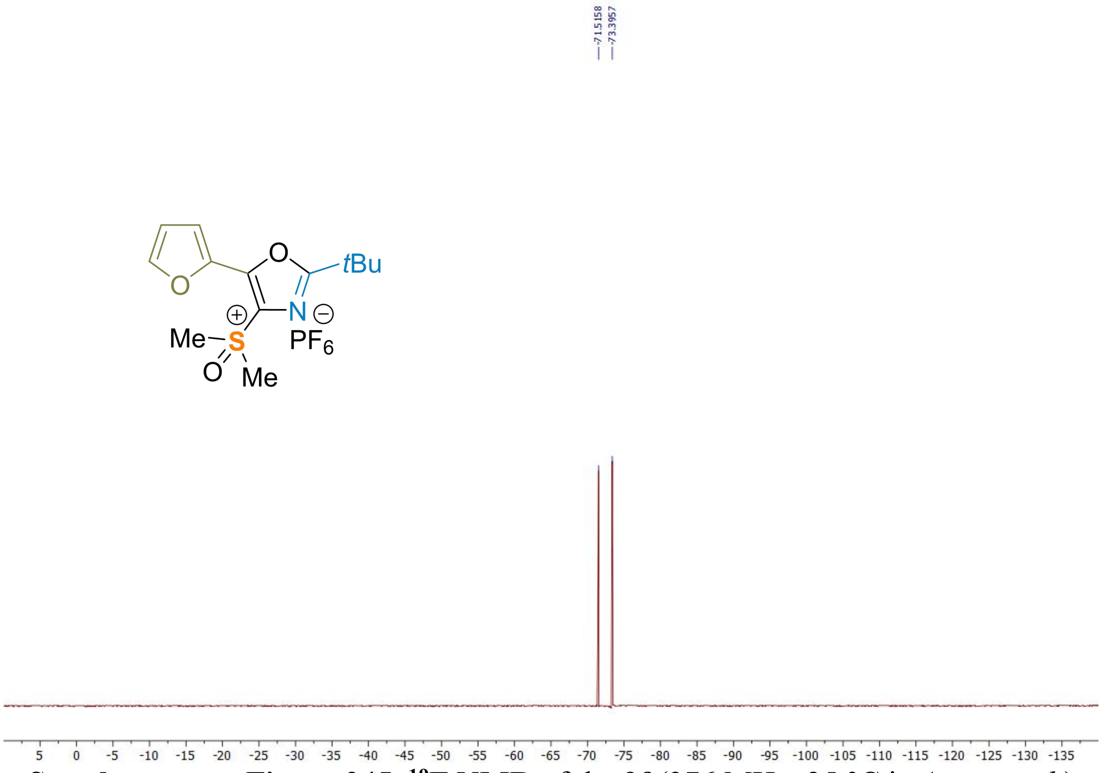

Supplementary Figure 245. ^{19}**F NMR** of the **9f** (376 MHz, 25 °C in Acetone-d_6)

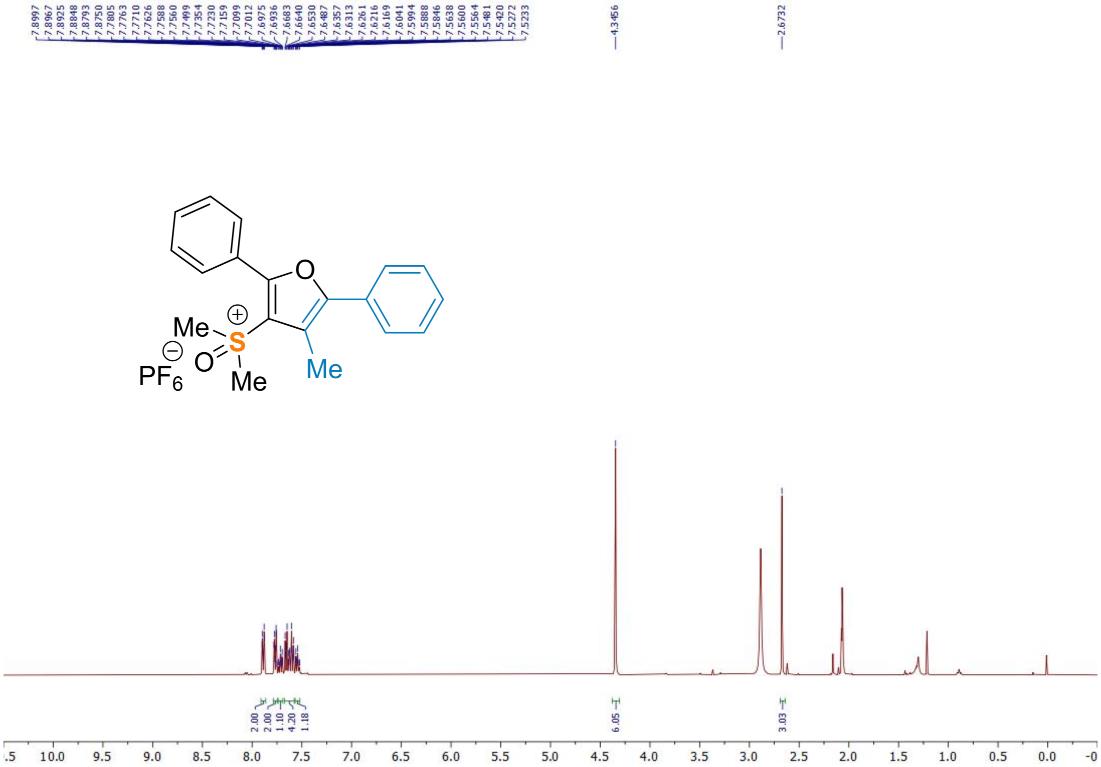

Supplementary Figure 246. ^{1}H NMR of the **10a** (400 MHz, 25 °C in Acetone-d_6)

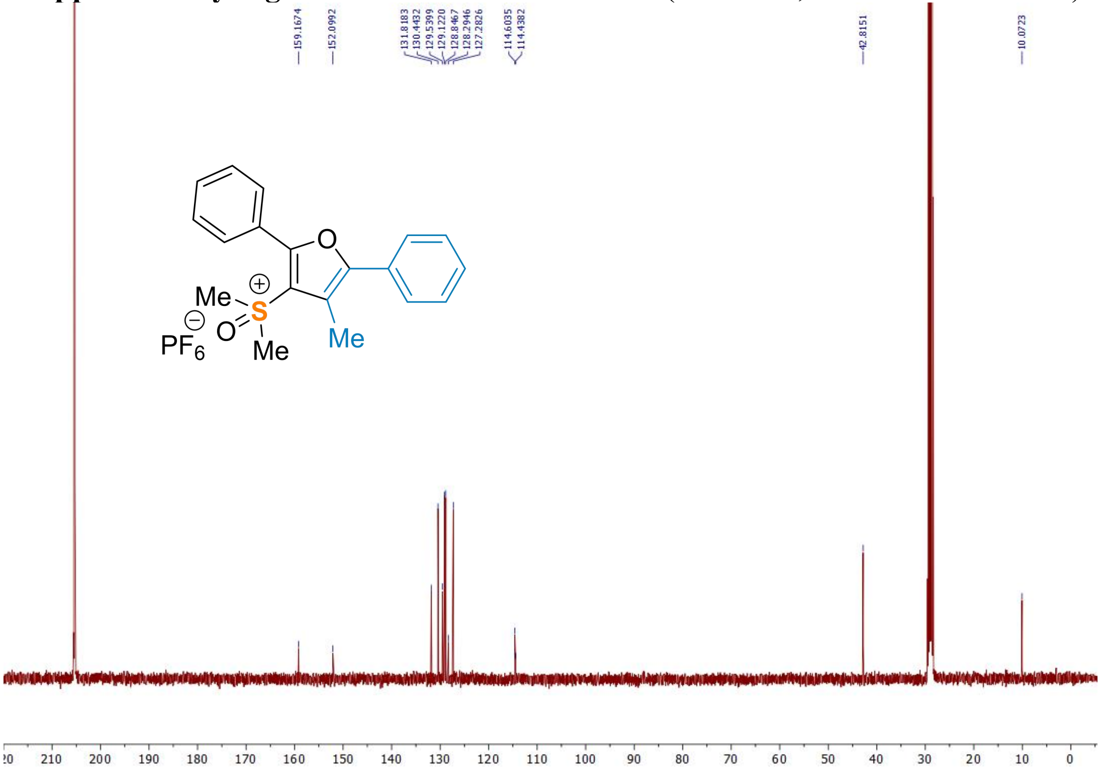

Supplementary Figure 247. ^{13}C NMR of the **10a** (101 MHz, 25 °C in Acetone-d_6)

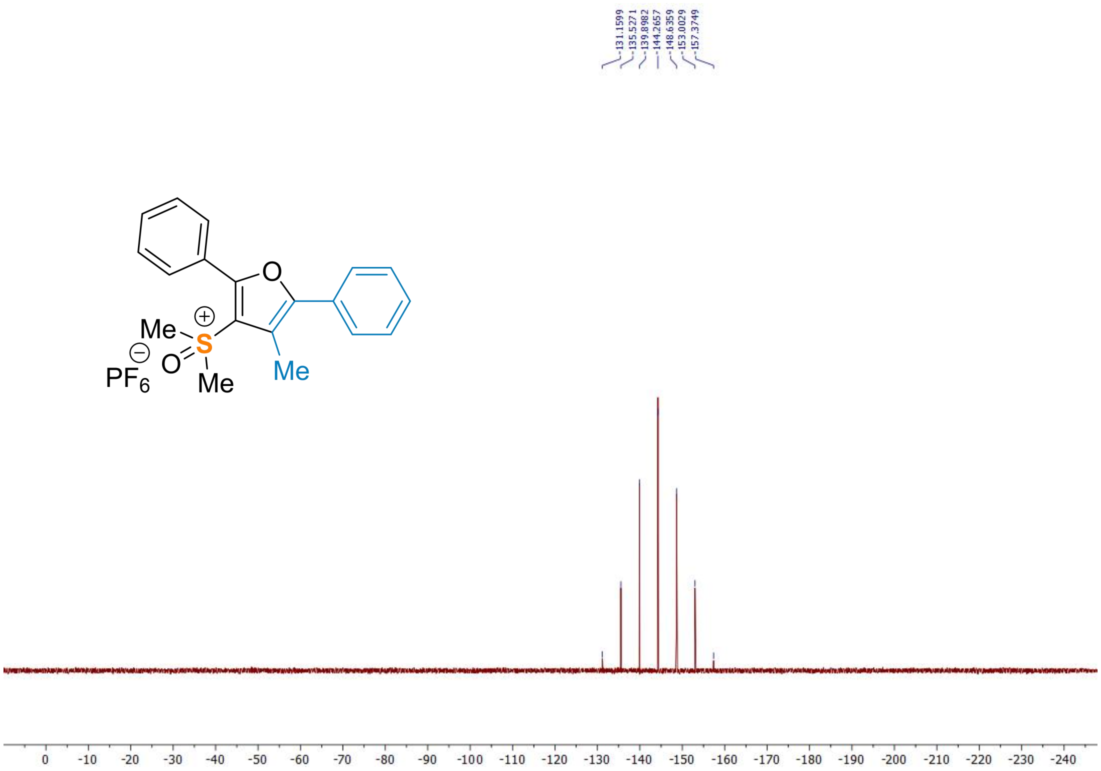

Supplementary Figure 248. ^{31}**P NMR** of the **10a** (162 MHz, 25 °C in Acetone-d_6)

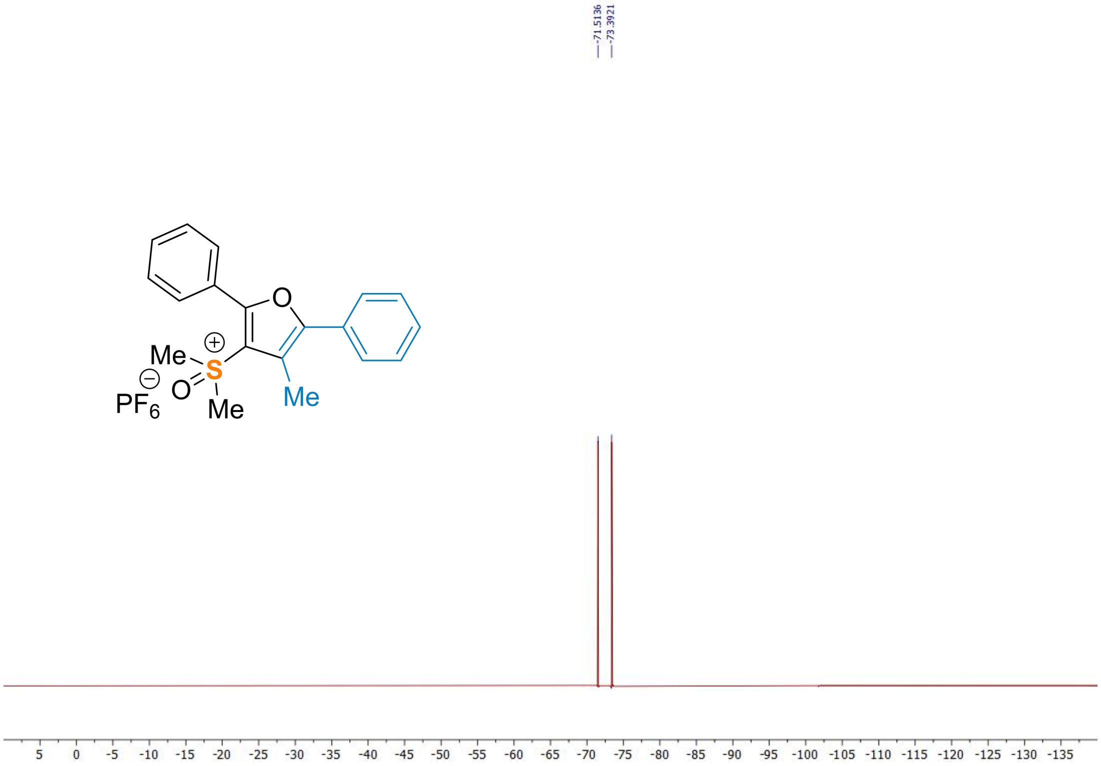

Supplementary Figure 249. ^{19}**F NMR** of the **10a** (376 MHz, 25 °C in Acetone-d_6)

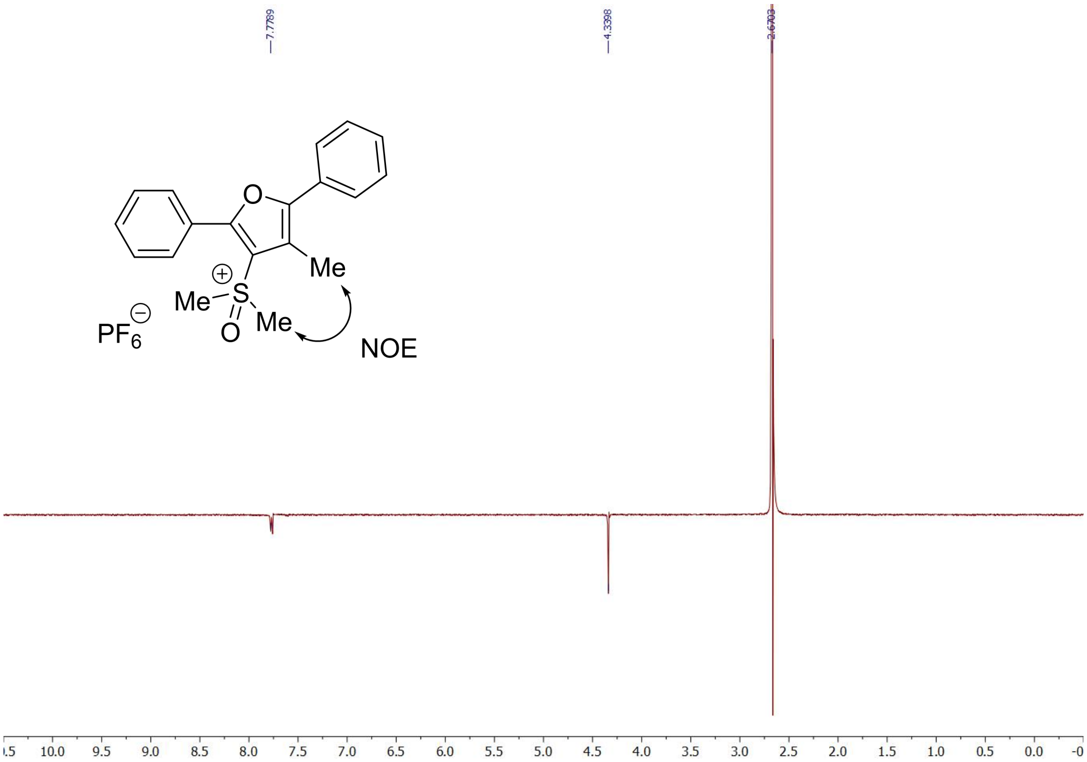

Supplementary Figure 250. nOe spectra of the **10a** (25 °C in Acetone-d_6)

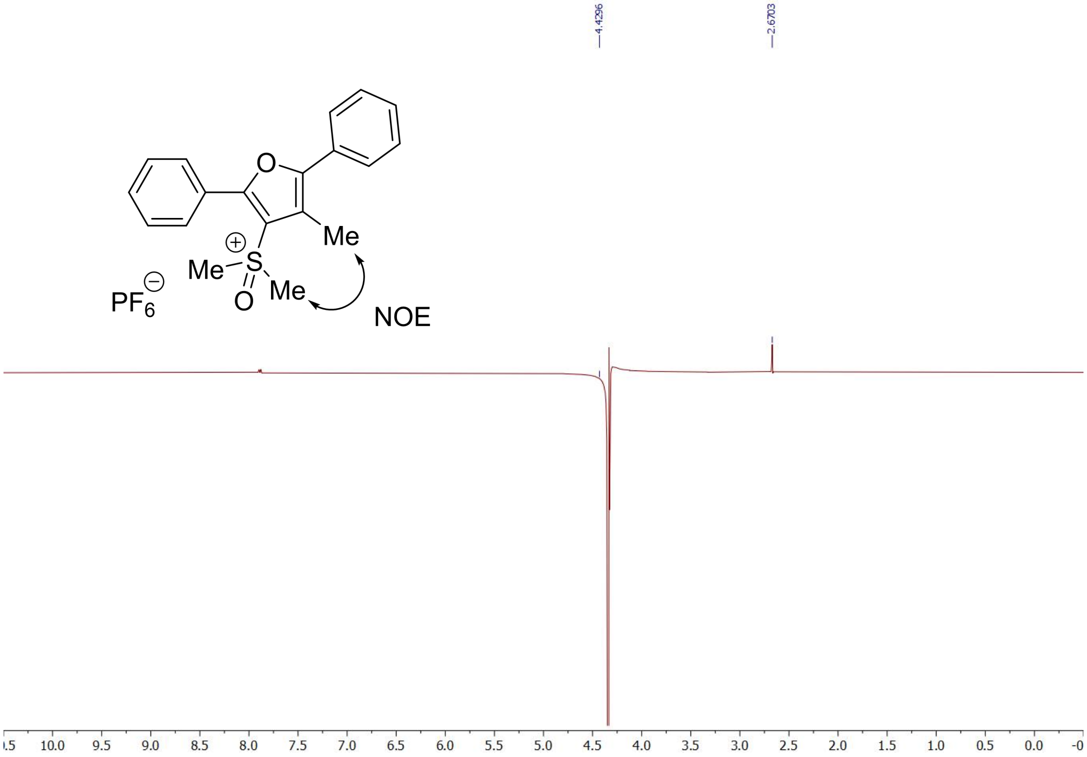

Supplementary Figure 251. nOe spectra of the **10a** (25 °C in Acetone-d_6)

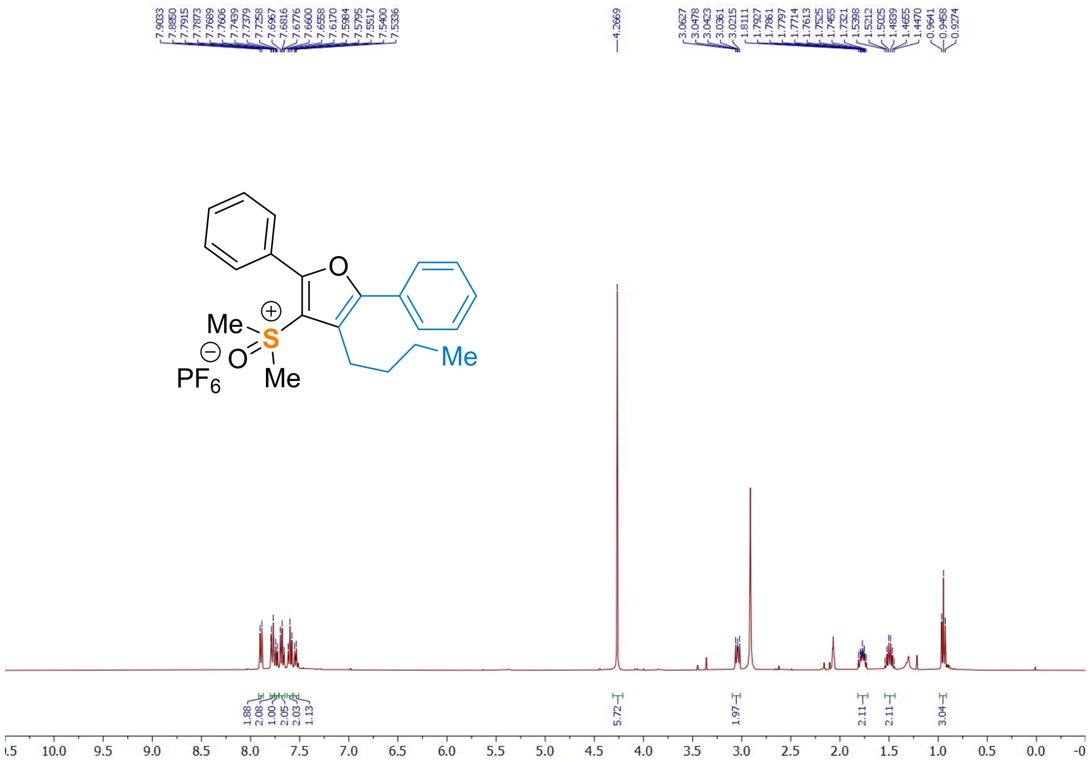

Supplementary Figure 252. ^{1}H NMR of the **10b** (400 MHz, 25 °C in Acetone-d_6)

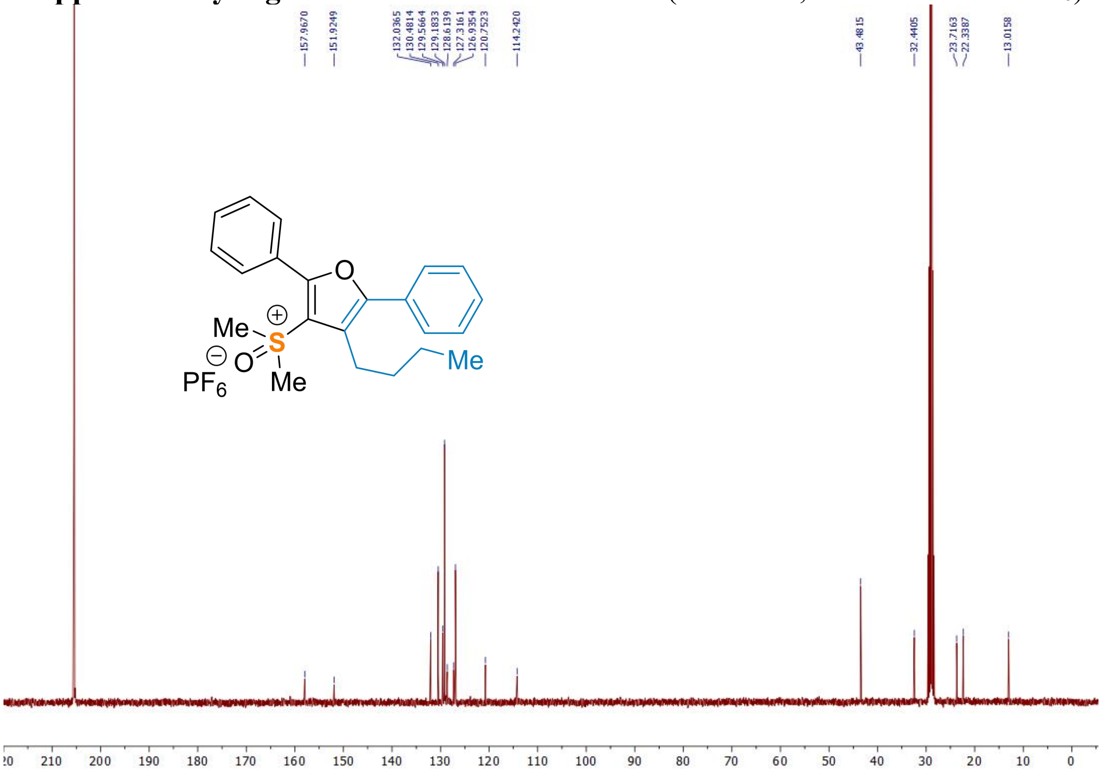

Supplementary Figure 253. ^{13}C NMR of the **10b** (101 MHz, 25 °C in Acetone-d_6)

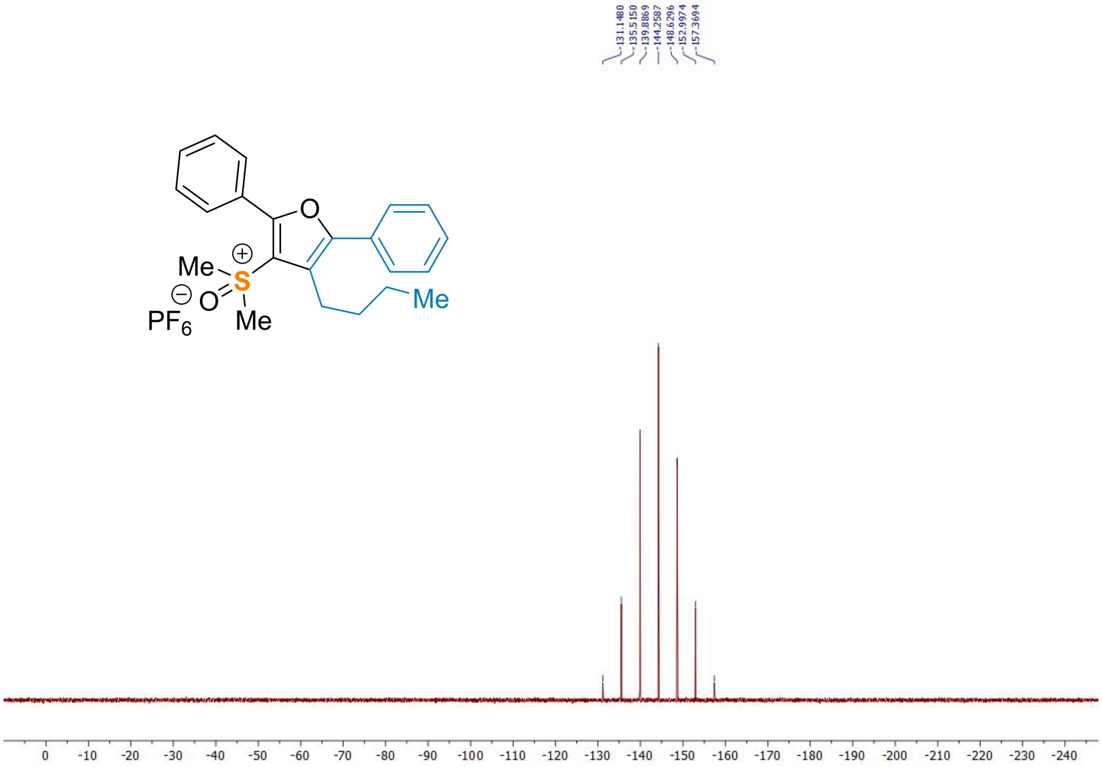

Supplementary Figure 254. ^{31}P NMR of the **10b** (162 MHz, 25 °C in Acetone-d_6)

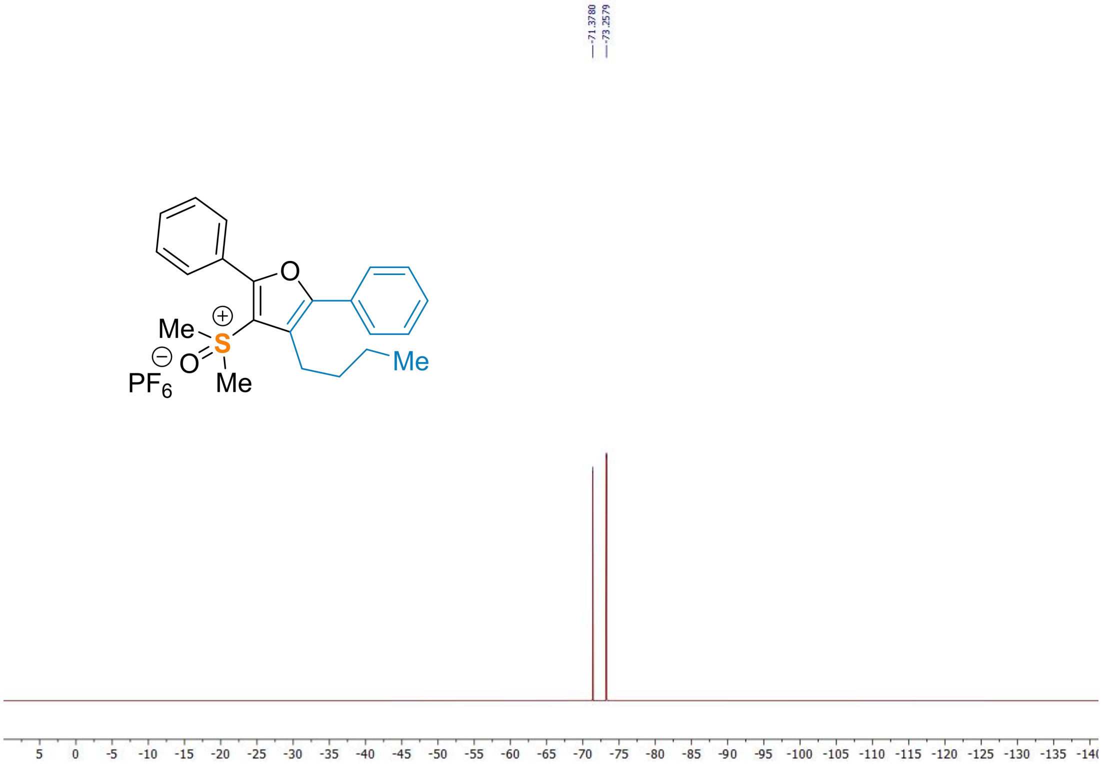

Supplementary Figure 255. ^{19}F NMR of the **10b** (376 MHz, 25 °C in Acetone-d_6)

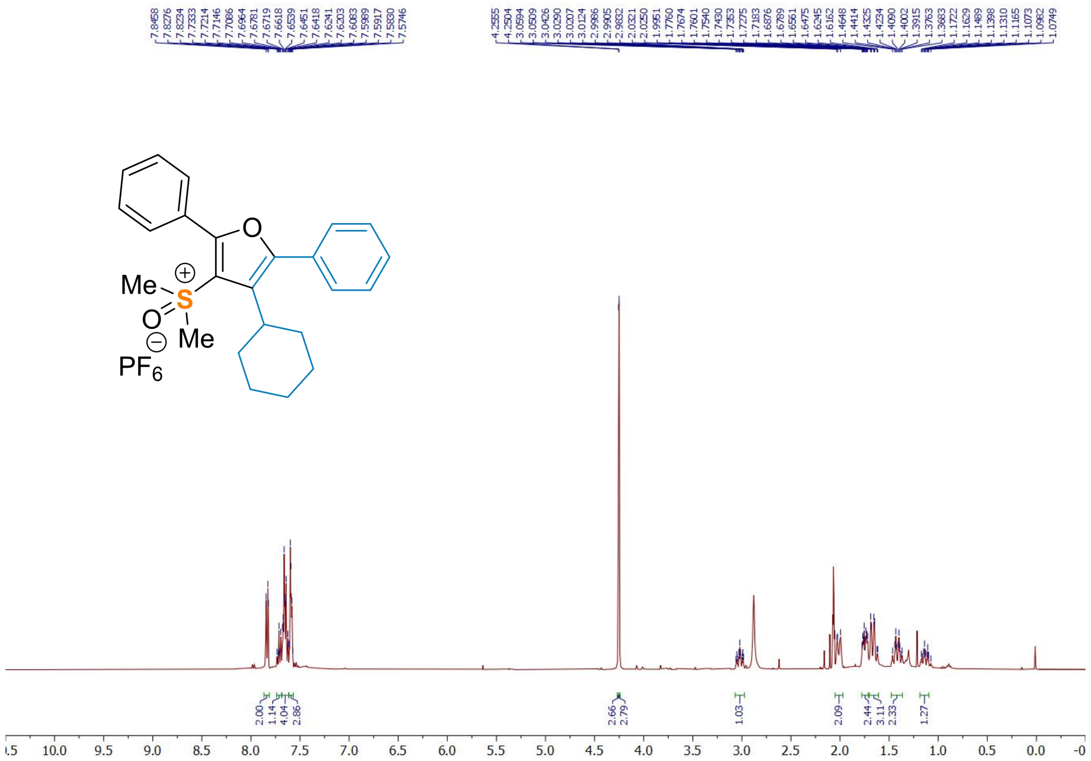

Supplementary Figure 256. ^{1}H NMR of the **10c** (400 MHz, 25 °C in Acetone-d_6)

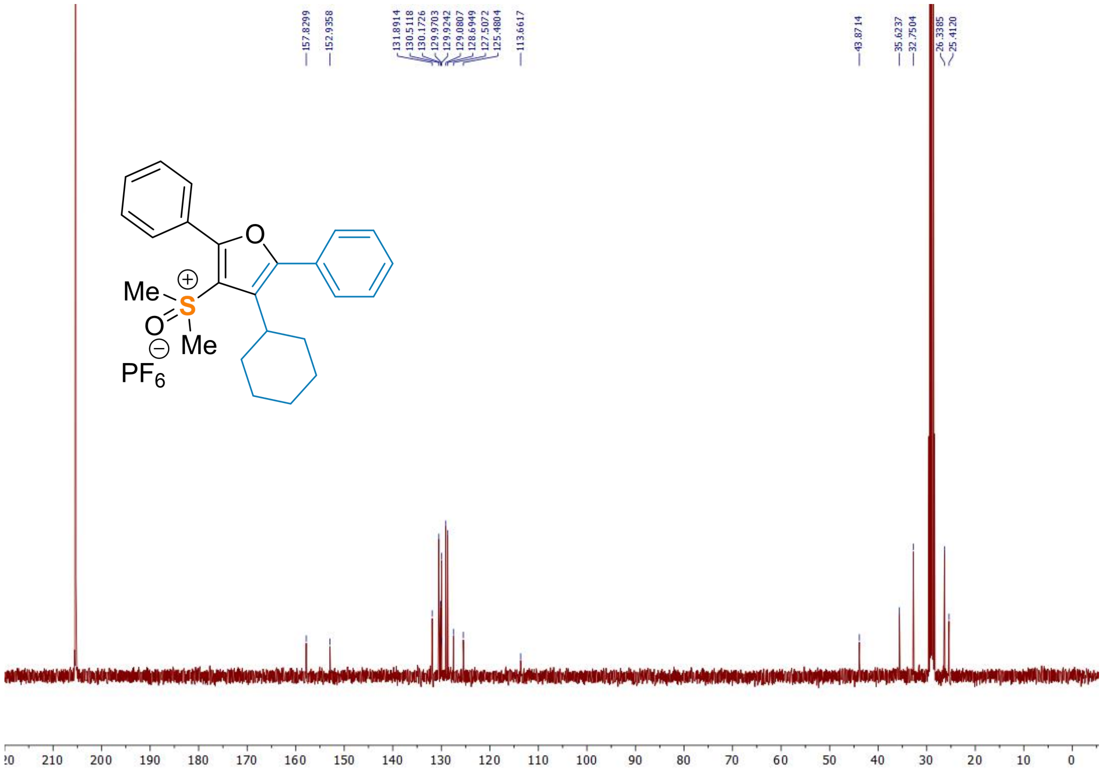

Supplementary Figure 257. ^{13}C NMR of the **10c** (101 MHz, 25 °C in Acetone-d_6)

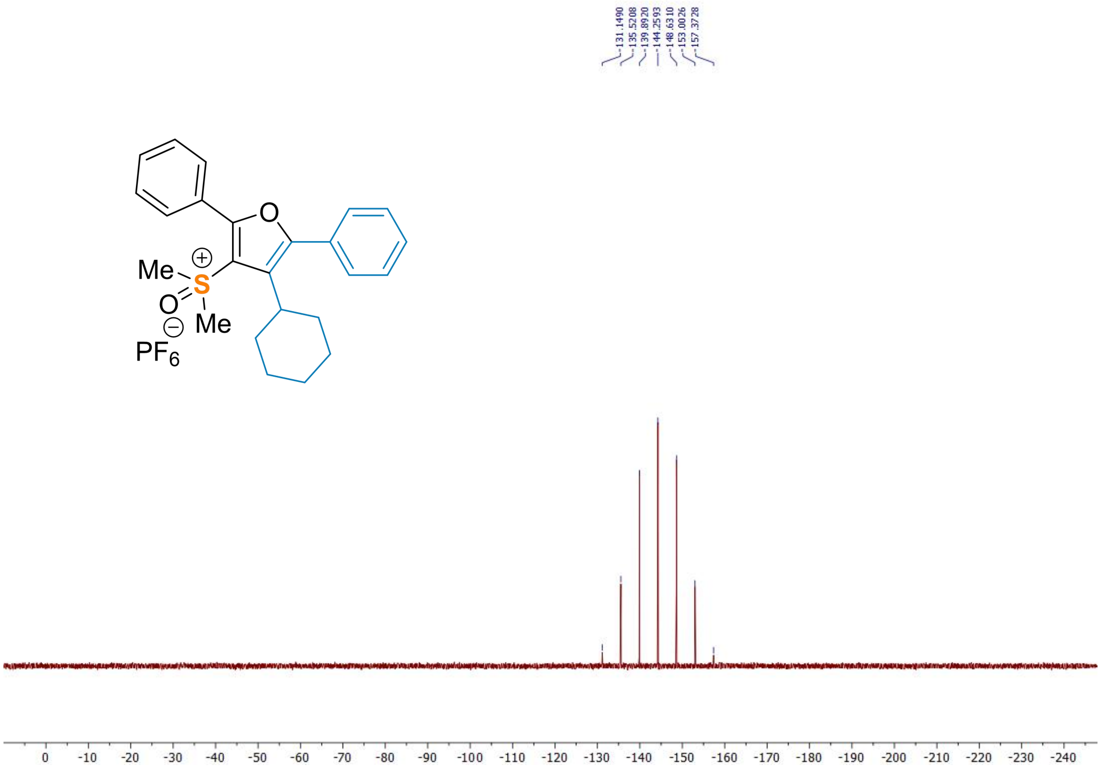

Supplementary Figure 258. ^{31}**P NMR** of the **10c** (162 MHz, 25 °C in Acetone-d_6)

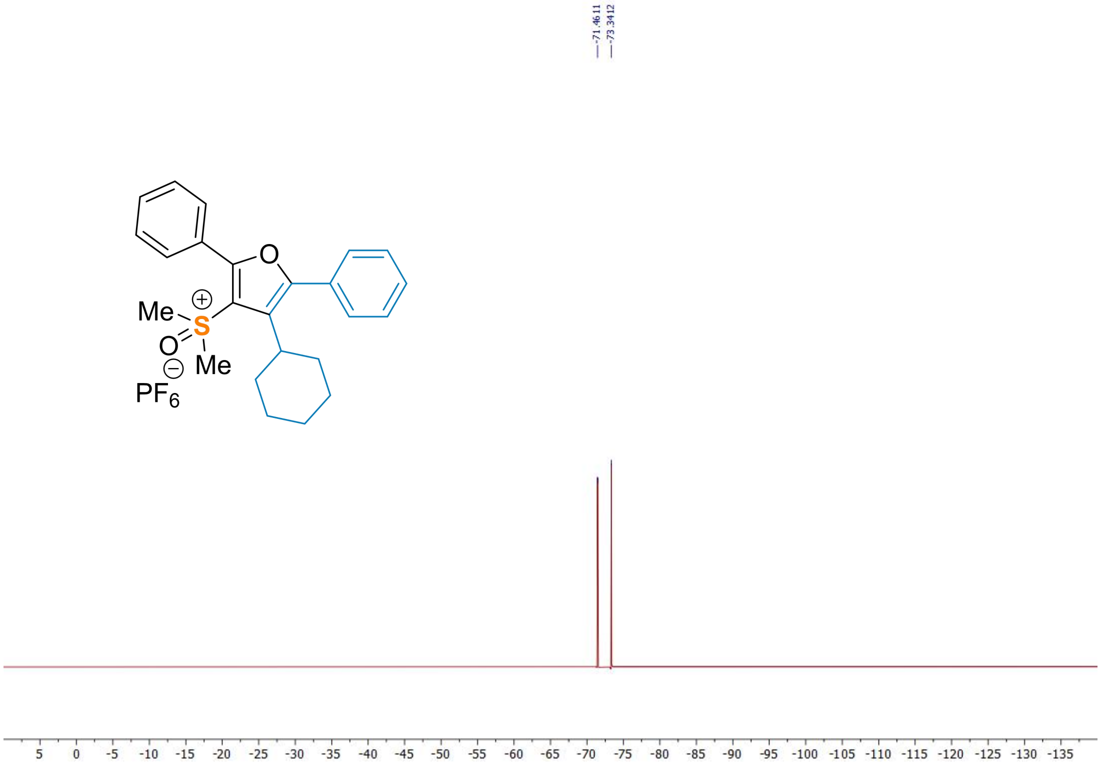

Supplementary Figure 259. ^{19}**F NMR** of the **10c** (376 MHz, 25 °C in Acetone-d_6)

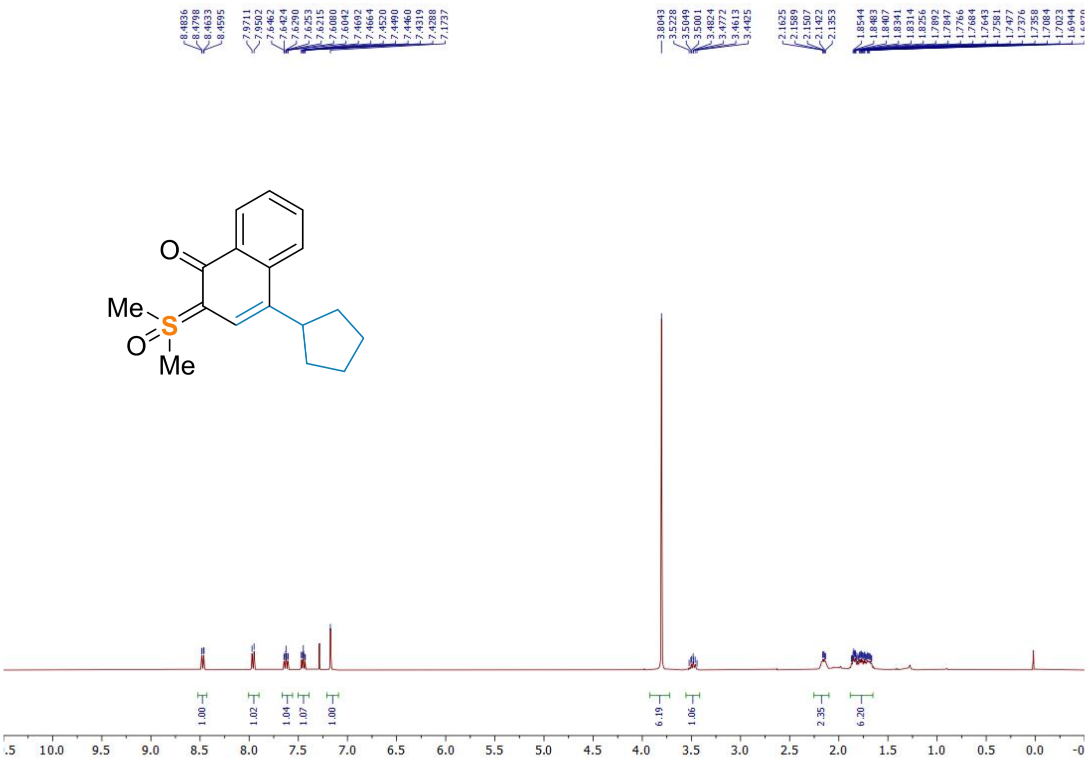

Supplementary Figure 260. **^{1}H NMR** of the **11a** (400 MHz, 25 °C in $CDCl_3$)

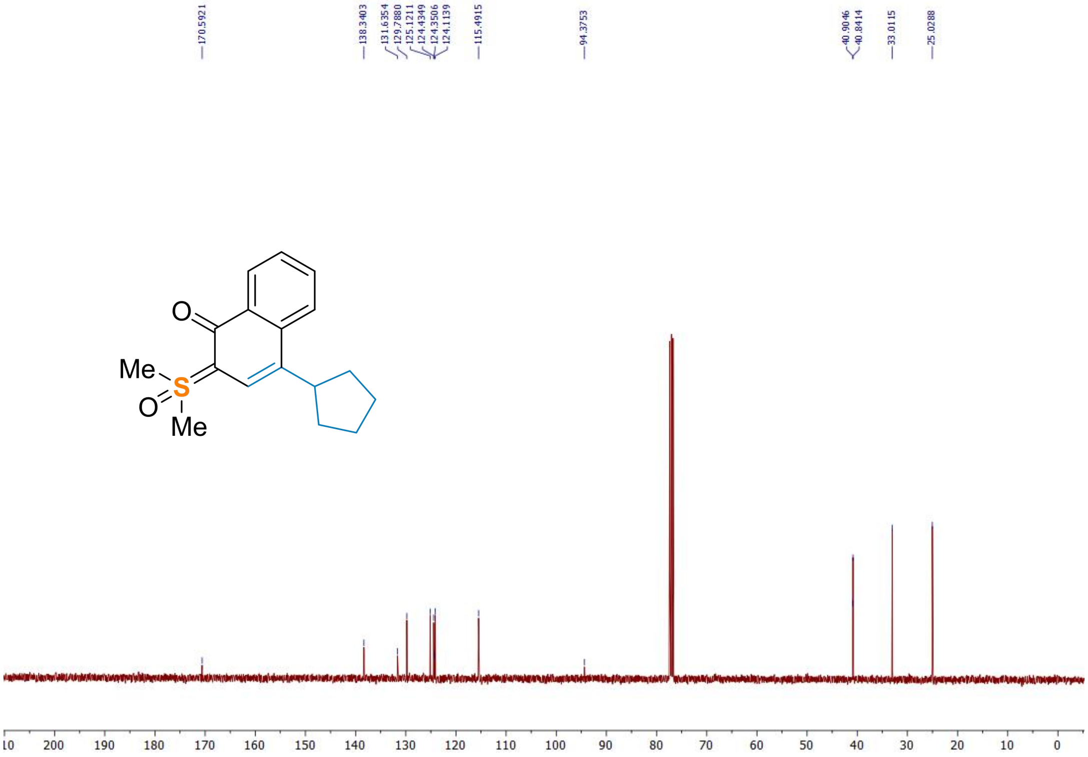

Supplementary Figure 261. **^{13}C NMR** of the **11a** (101 MHz, 25 °C in $CDCl_3$)

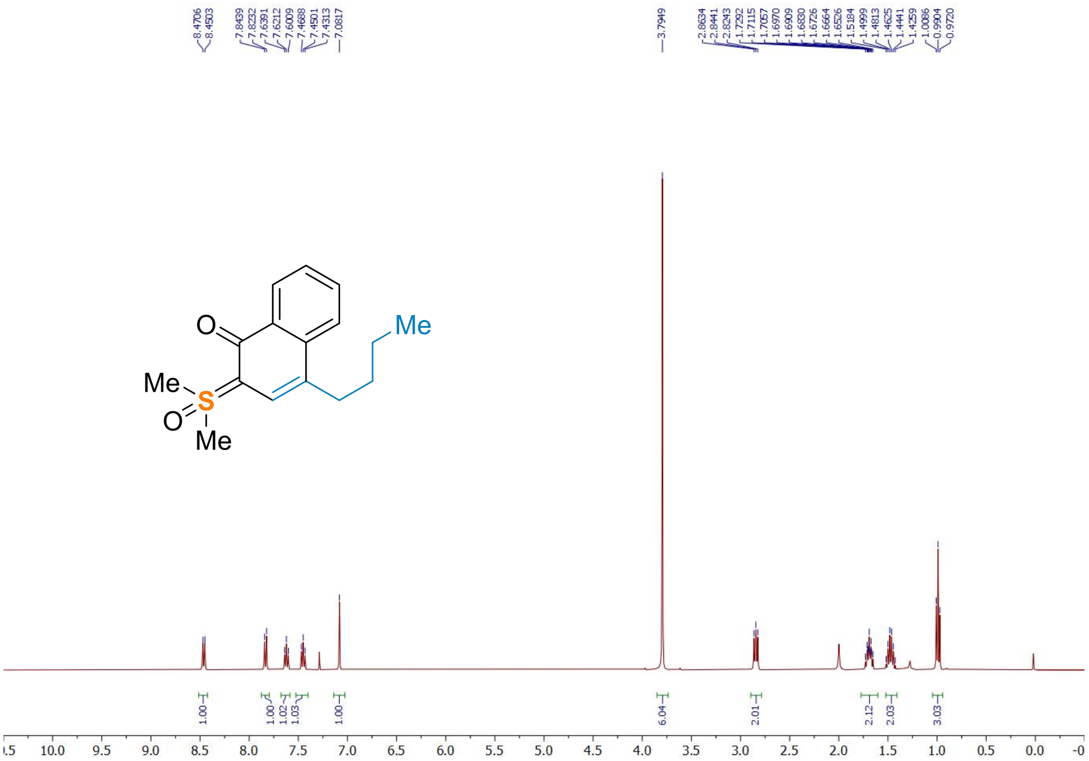

Supplementary Figure 262. ^{1}**H NMR** of the **11b** (400 MHz, 25 °C in $CDCl_3$)

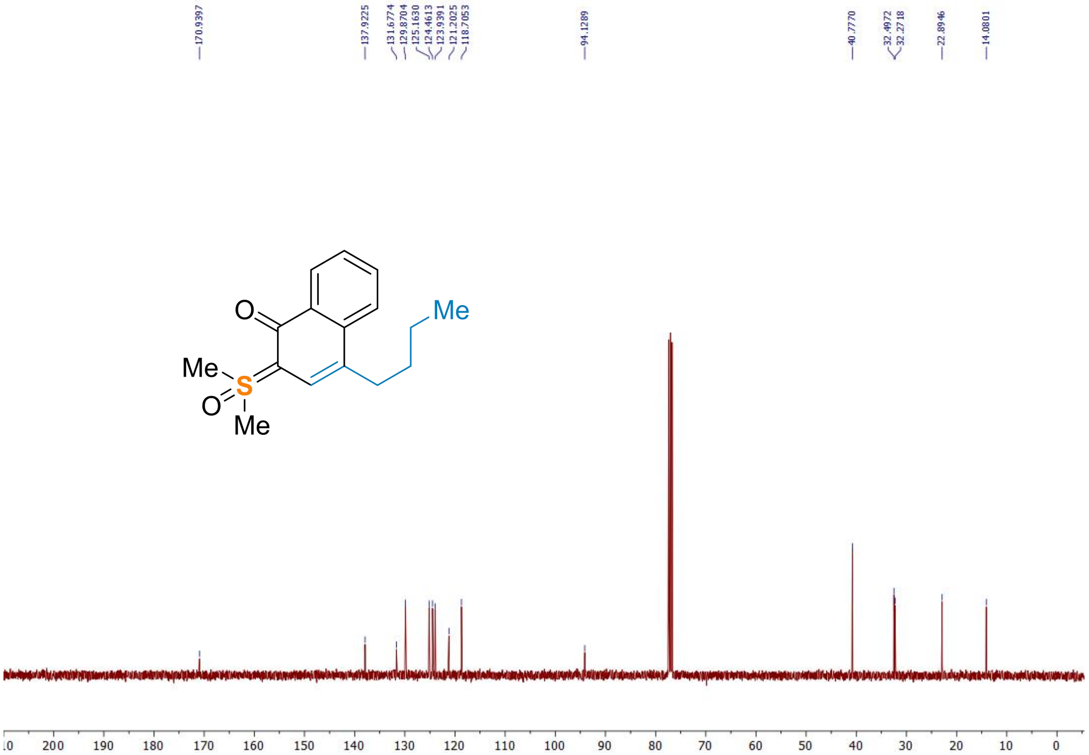

Supplementary Figure 263. ^{13}**C NMR** of the **11b** (101 MHz, 25 °C in $CDCl_3$)

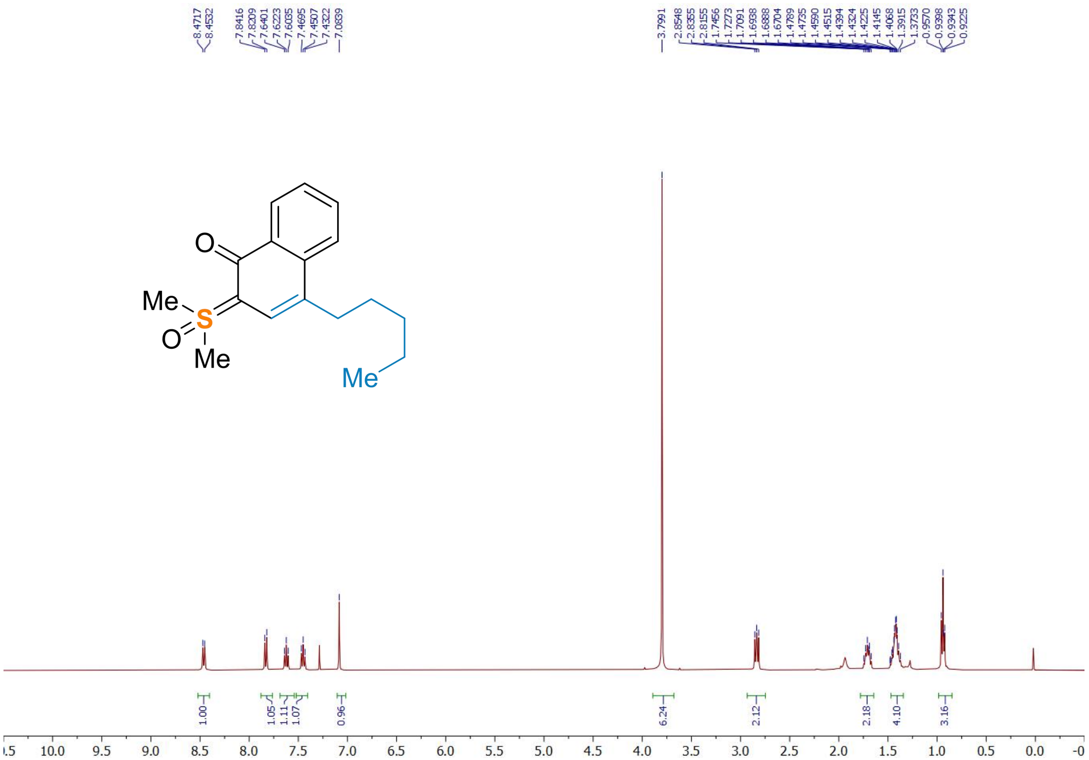

Supplementary Figure 264. ^{1}**H NMR** of the **11c** (400 MHz, 25 °C in $CDCl_3$)

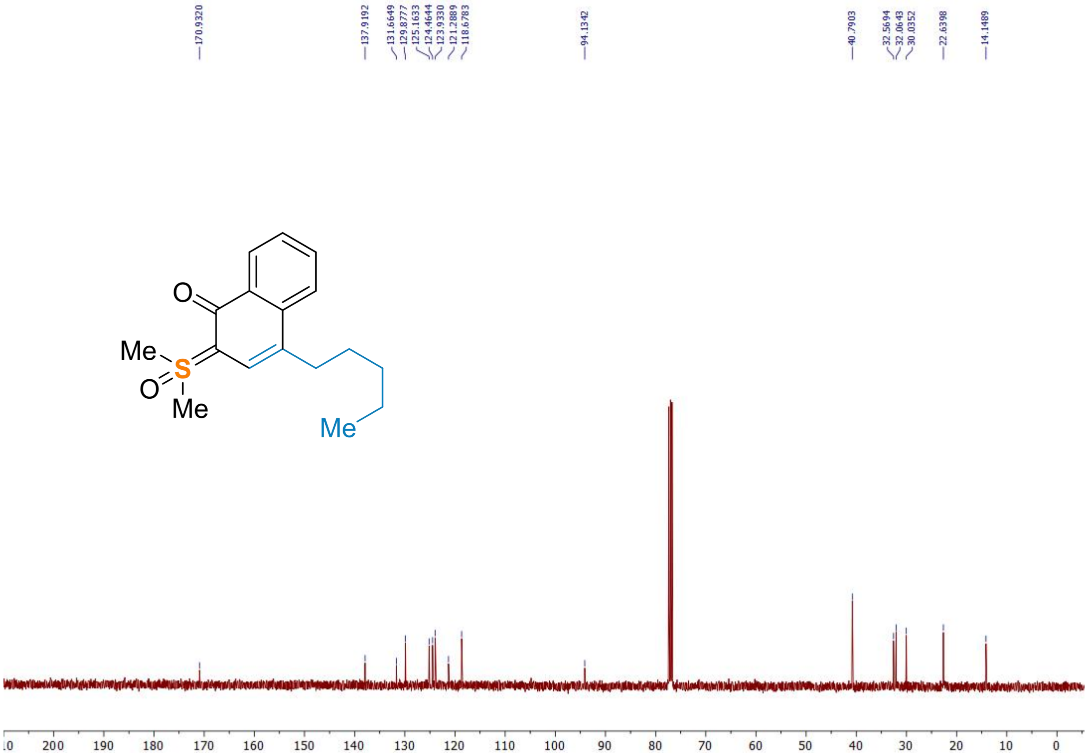

Supplementary Figure 265. ^{13}**C NMR** of the **11c** (101 MHz, 25 °C in $CDCl_3$)

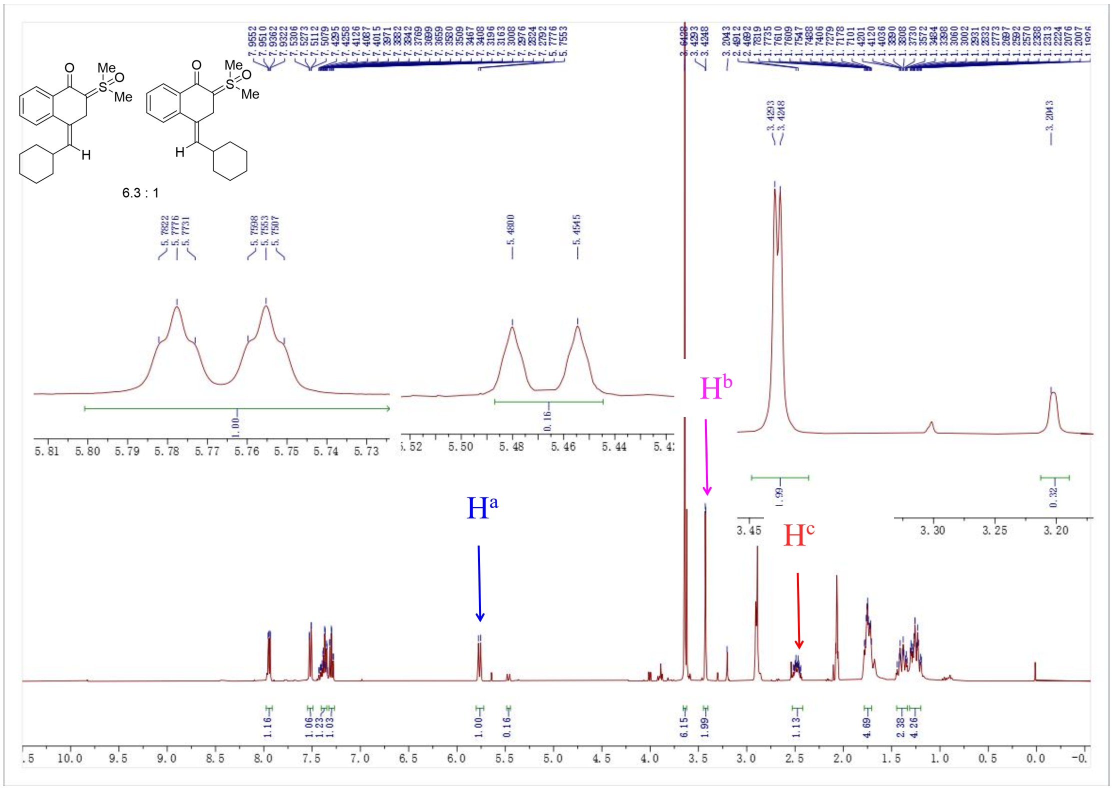

Supplementary Figure 266. ^{1}**H NMR** of the **12** (400 MHz, 25 °C in Acetone-d_6)

major: *Z* isomer

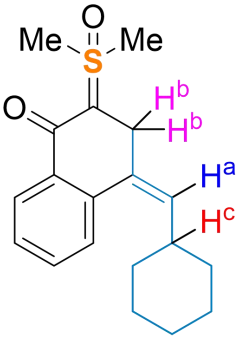

minor: *E* isomer

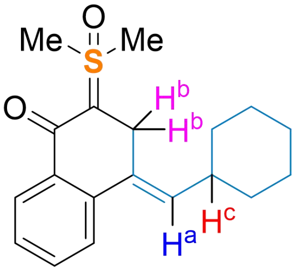

5.77 (dt, J = 8.9, 1.8 Hz, 1H^a)
3.43 (d, J = 1.8 Hz, 2H^b)
$J_{H^a\text{-}H^c}$ = 8.9 Hz
$J_{H^b\text{-}H^a}$ = 1.8 Hz

5.47 (d, J = 10.2 Hz Hz, 0.16*1H^a)
3.20 (s, 0.32*2H$^{b)}$
$J_{H^a\text{-}H^c}$ = 10.2 Hz
$J_{H^b\text{-}H^a}$ not detected

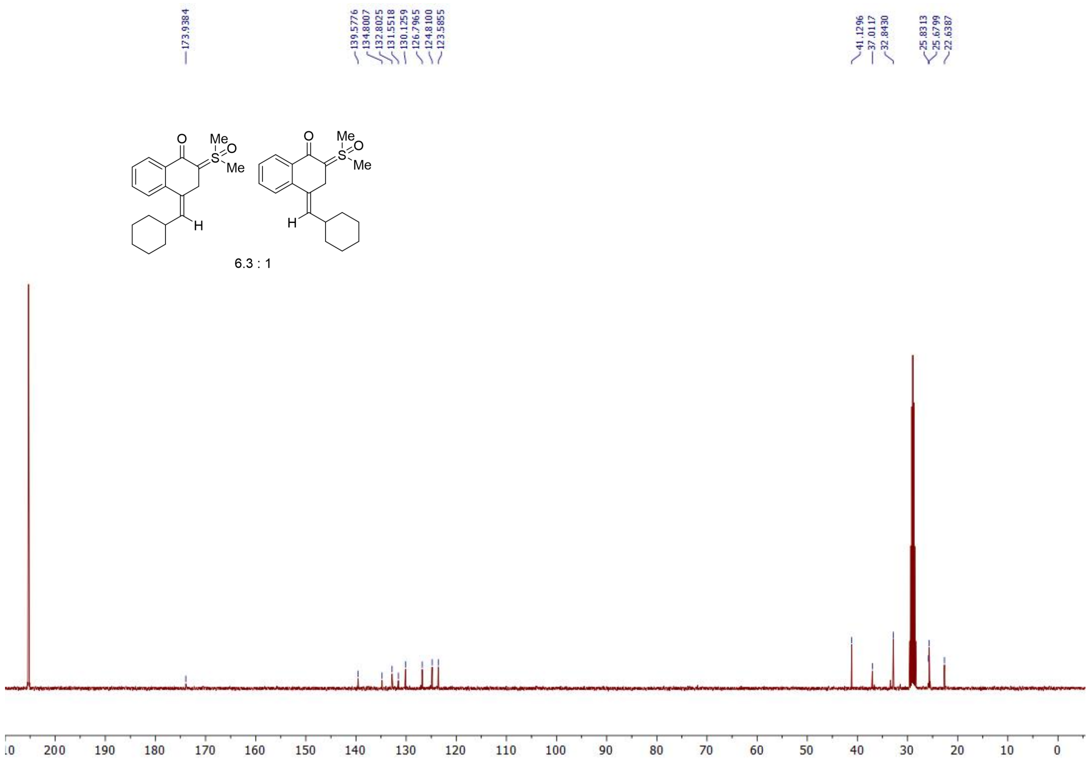

Supplementary Figure 267. ^{13}C NMR of the **12** (101 MHz, 25 °C in Acetone-d_6)

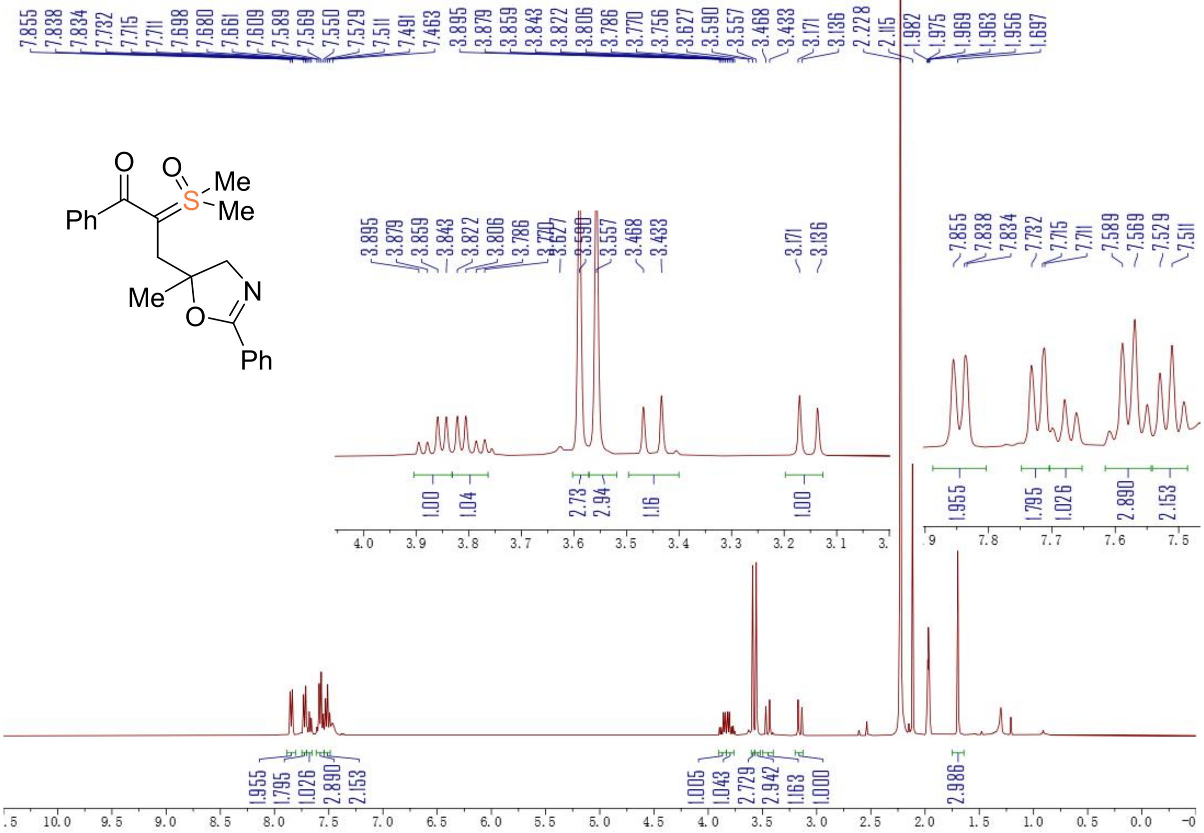

Supplementary Figure 268. ^{1}H NMR of the **13** (400 MHz, 25 °C in Acetonitrile-d_3)

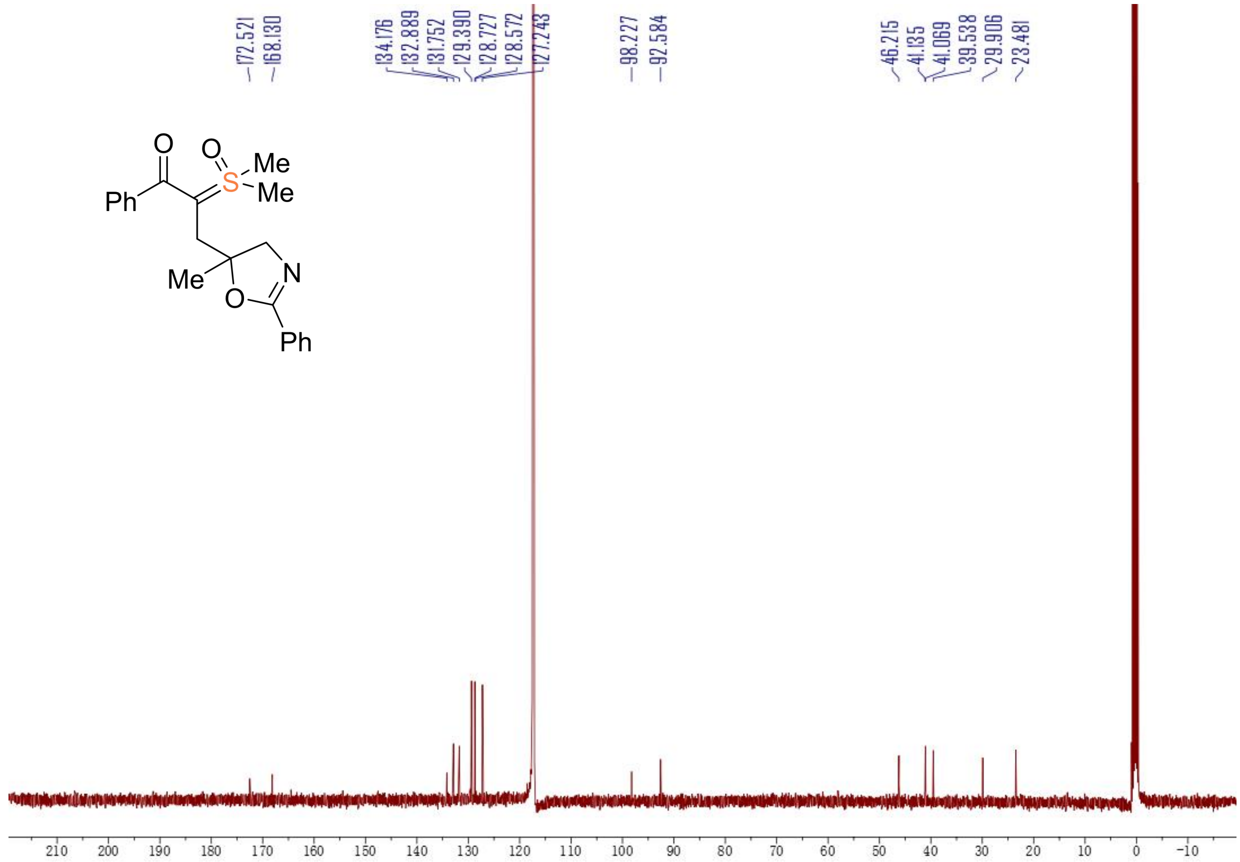

Supplementary Figure 269. ^{13}C NMR of the **13** (101 MHz, 25 °C in Acetonitrile-d_3)

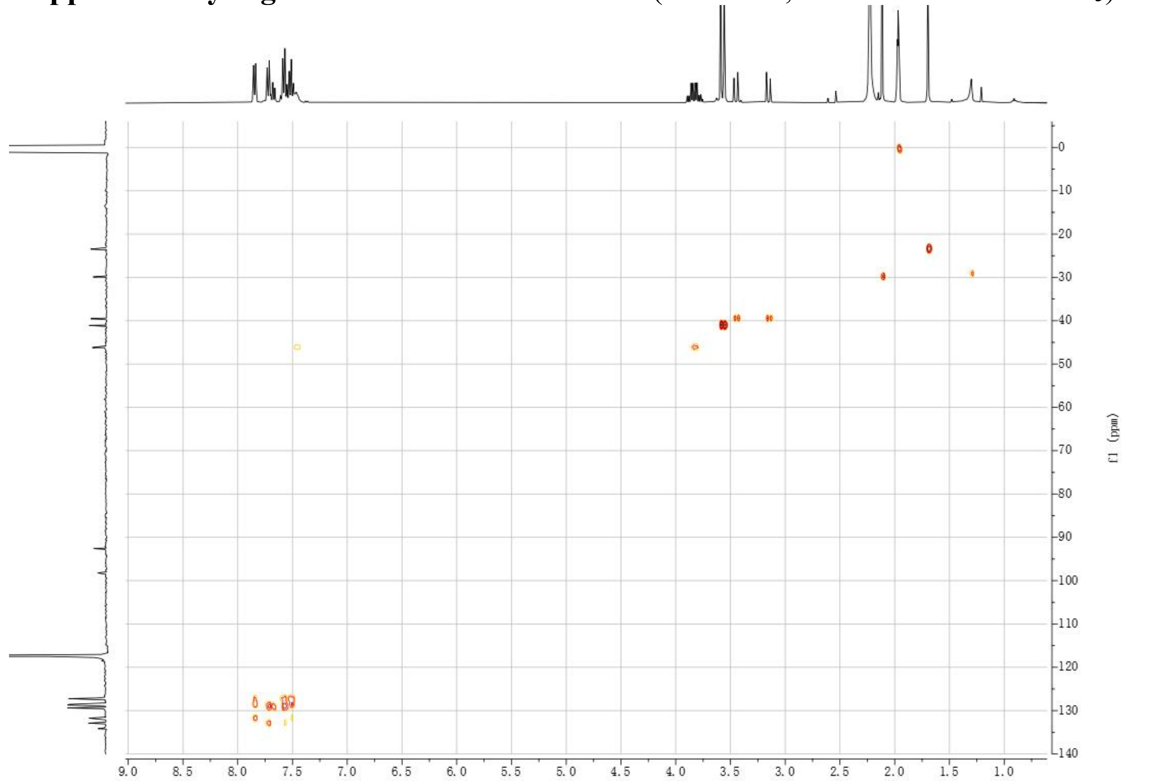

Supplementary Figure 270. ^{1}H-^{13}C HSQC of the **13** (101 MHz, 25 °C in Acetonitrile-d_3)

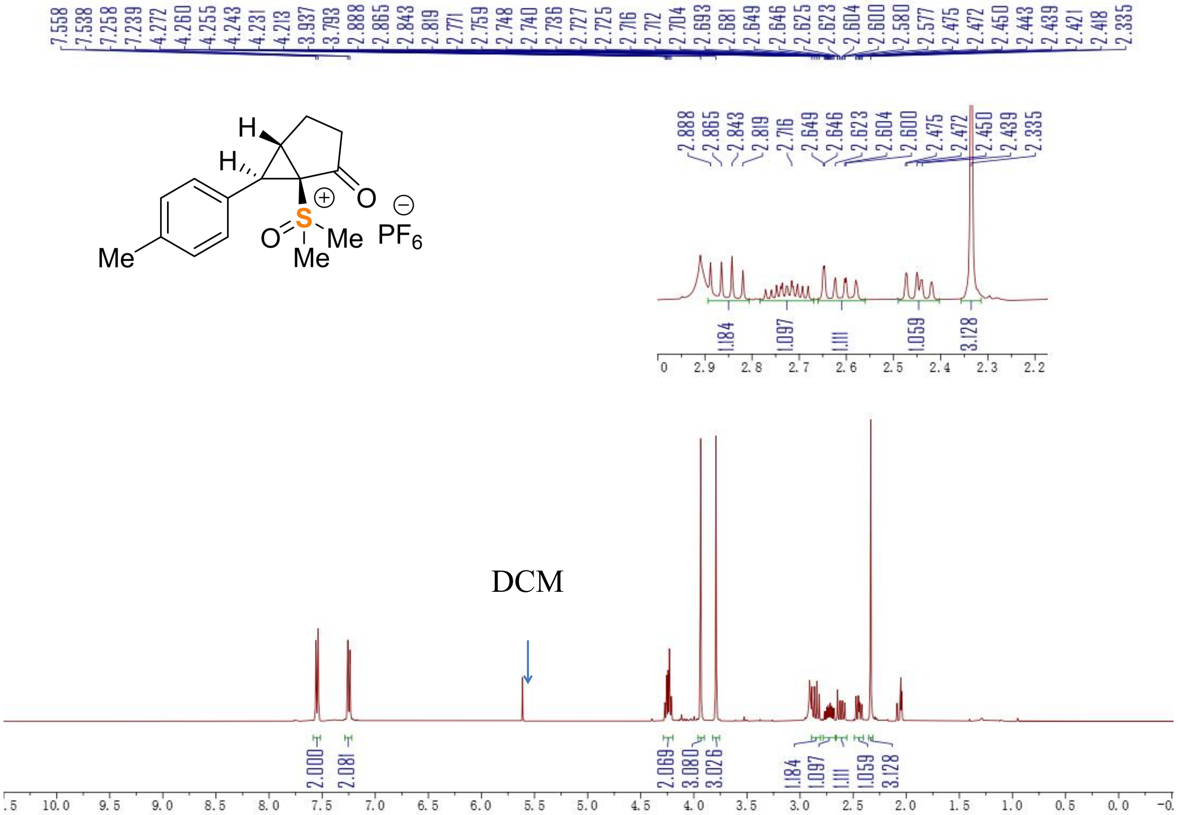

Supplementary Figure 271. ^{1}H NMR of the **14a** (400 MHz, 25 °C in Acetone-d_6)

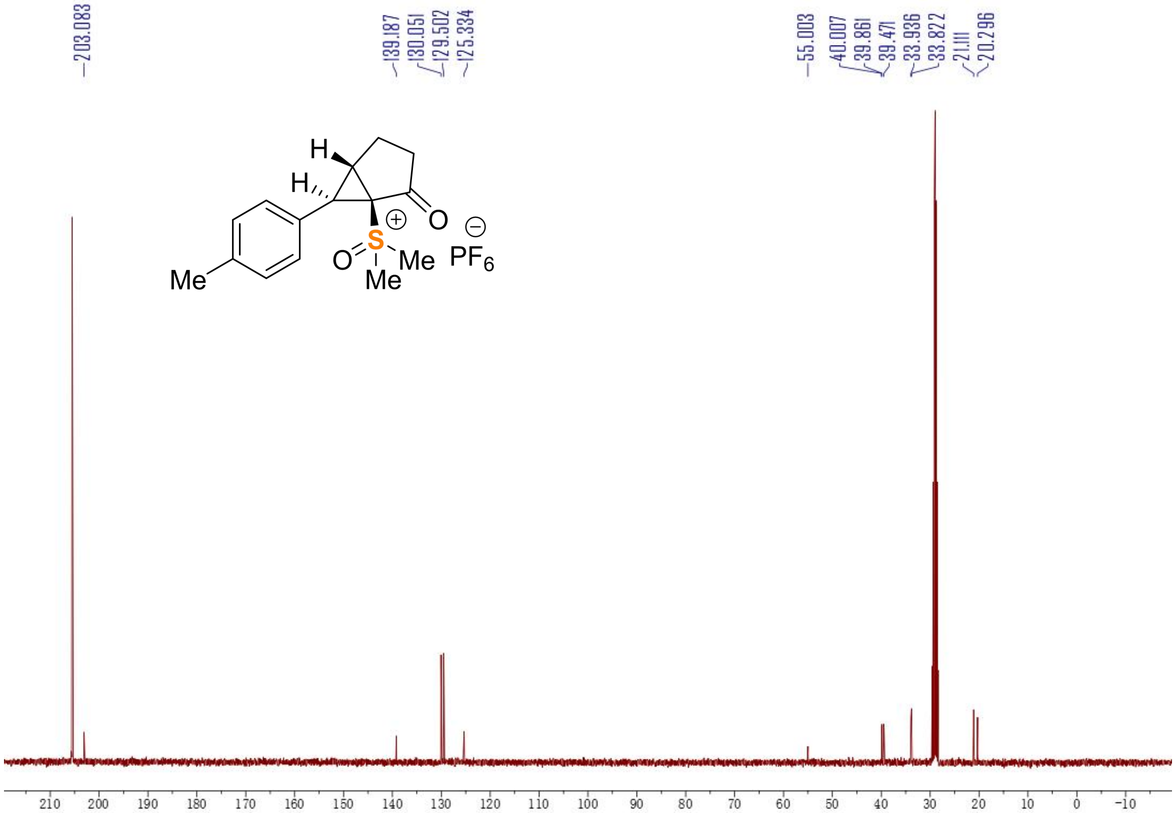

Supplementary Figure 272. ^{13}C NMR of the **14a** (400 MHz, 25 °C in Acetone-d_6)

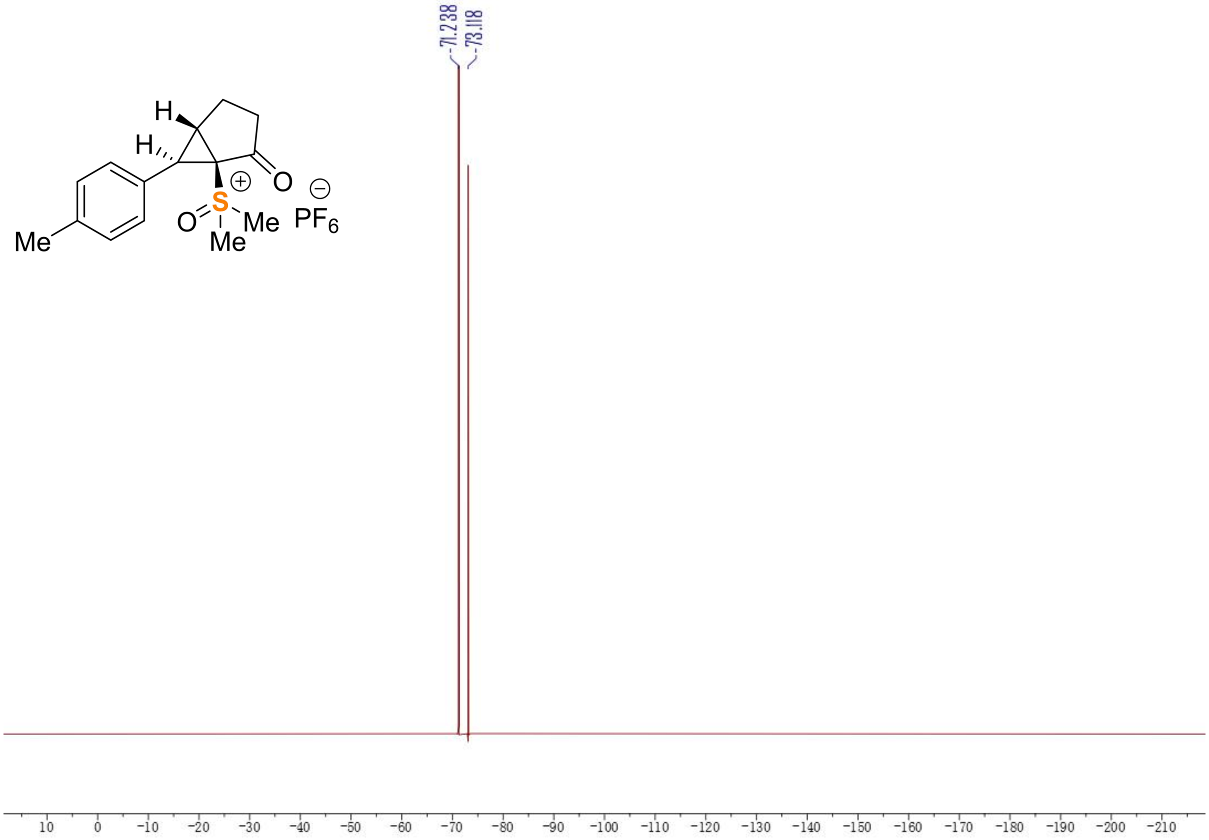

Supplementary Figure 273. ^{19}**F NMR** of the **14a** (376 MHz, 25 °C in Acetone-d_6)

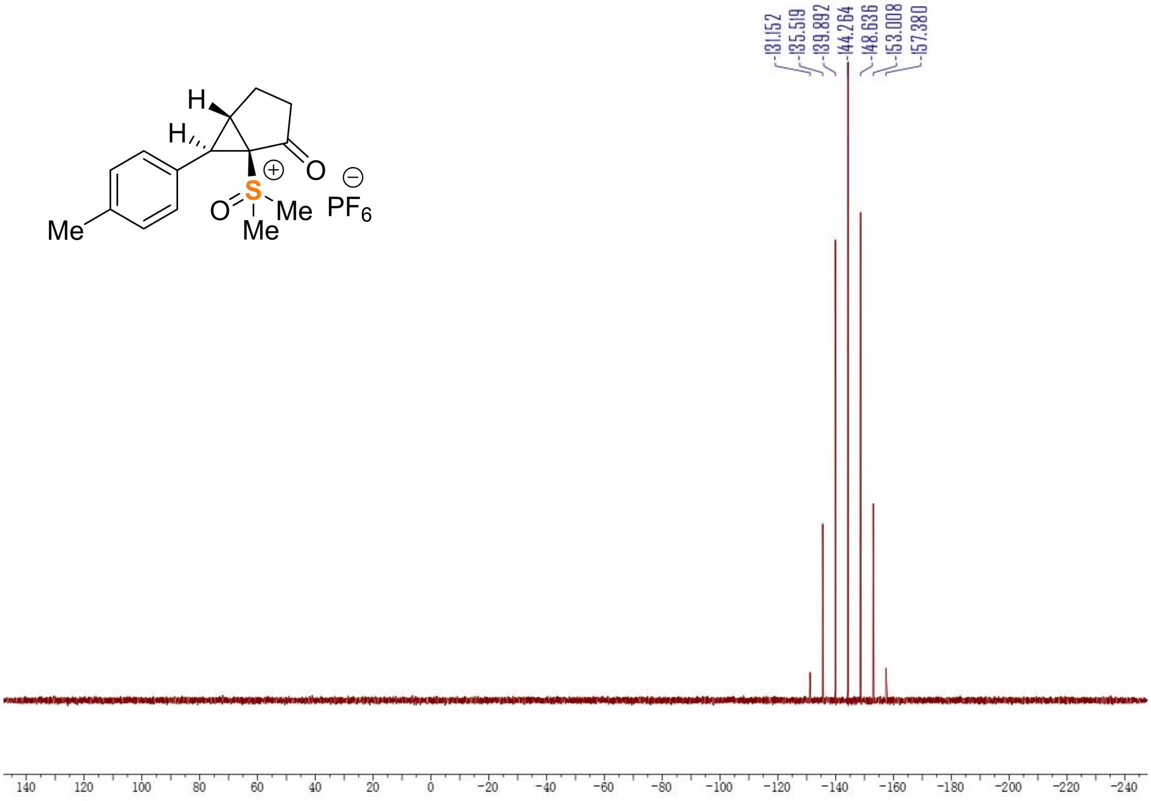

Supplementary Figure 274. ^{31}**P NMR** of the **14a** (162MHz, 25 °C in Acetone-d_6)

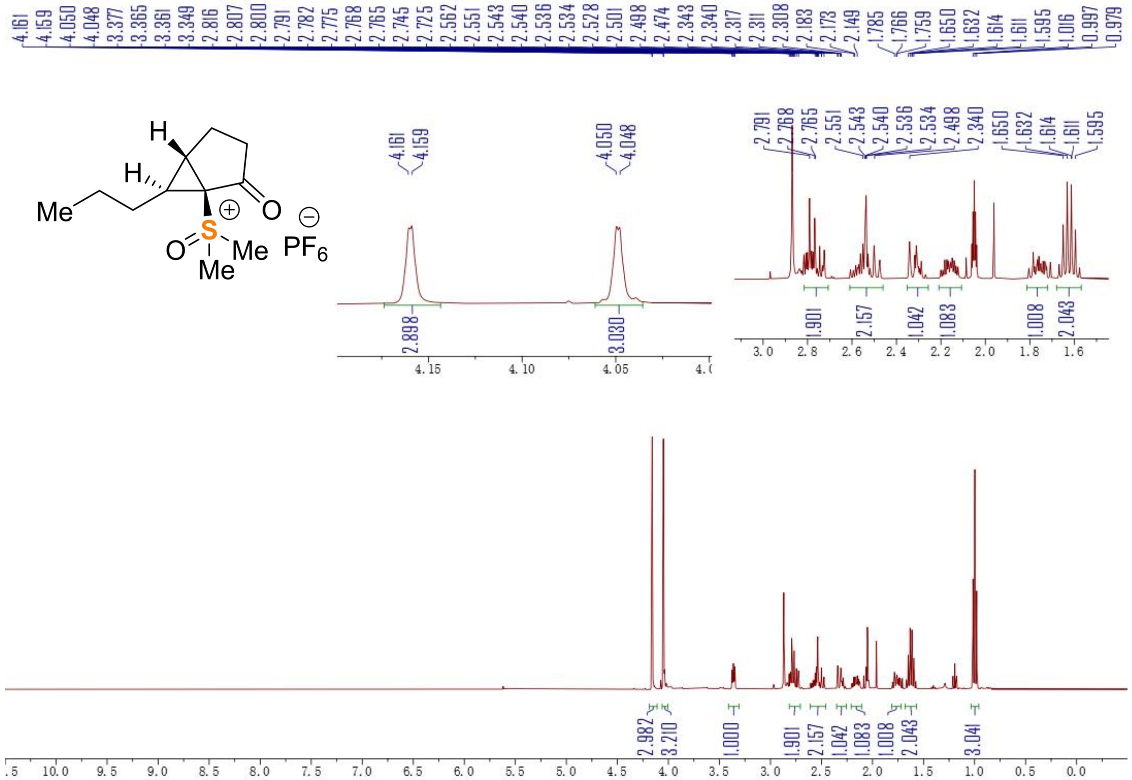

Supplementary Figure 275. ^{1}**H NMR** of the **14b** (400 MHz, 25 °C in Acetone-d_6)

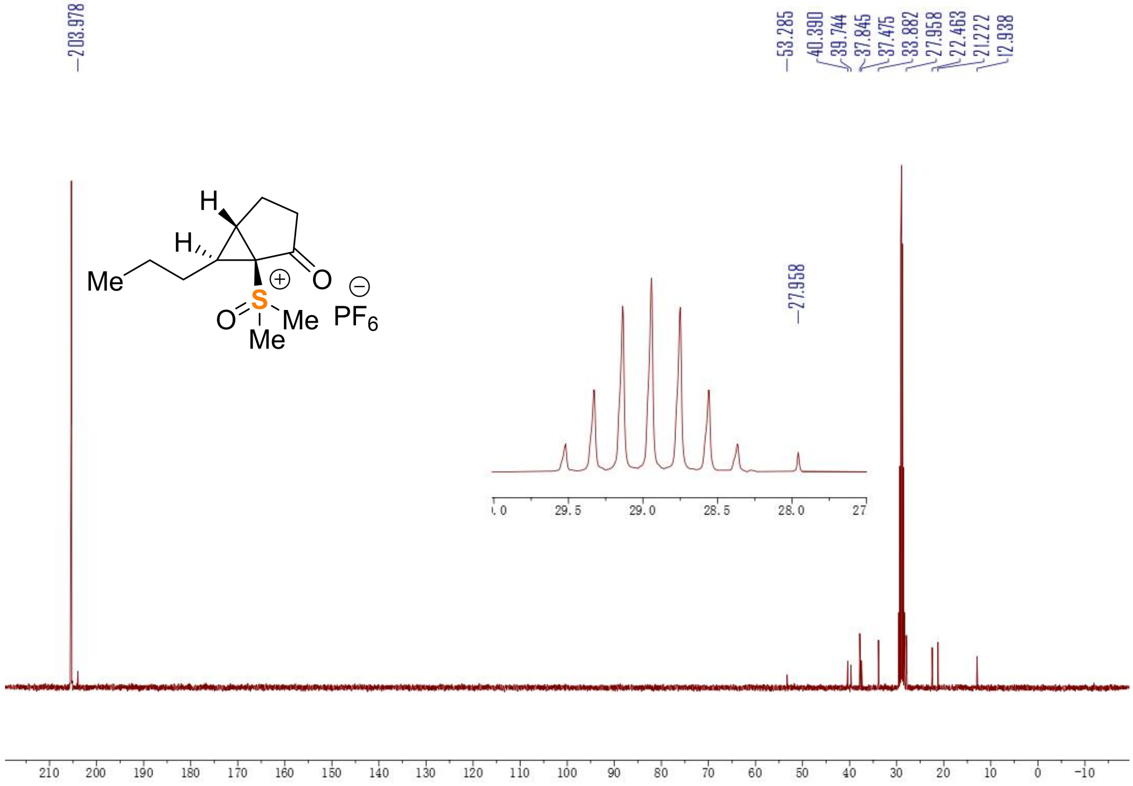

Supplementary Figure 276. ^{13}**C NMR** of the **14b** (400 MHz, 25 °C in Acetone-d_6)

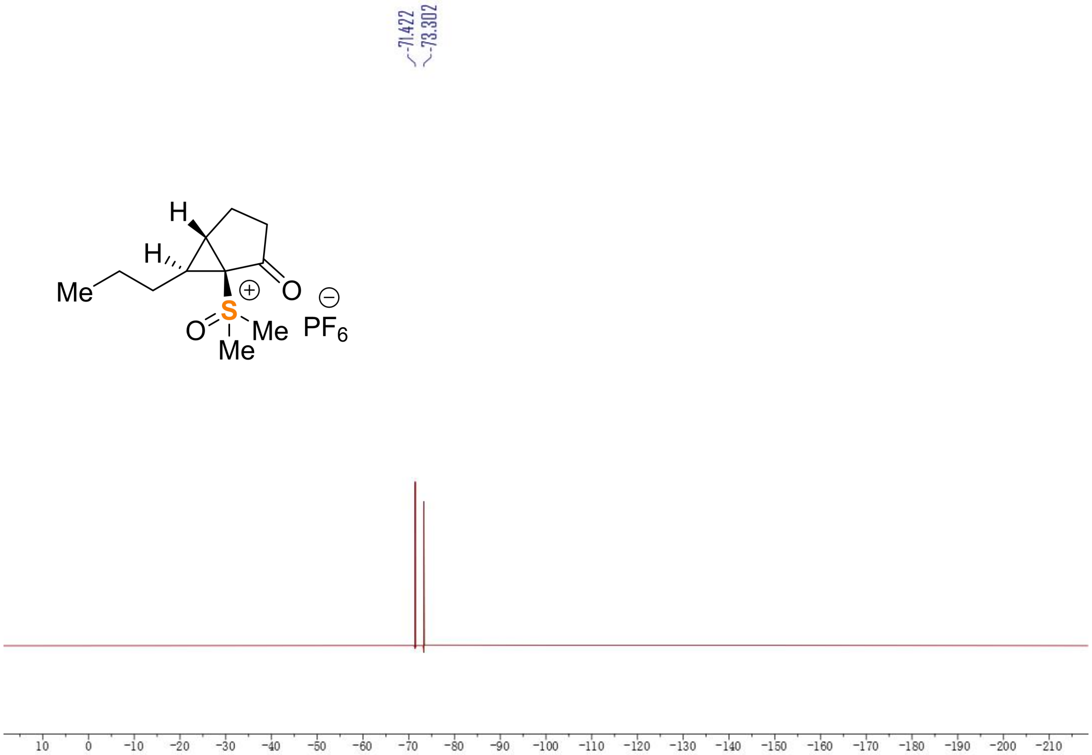

Supplementary Figure 277. ^{19}**F NMR** of the **14b** (376 MHz, 25 °C in Acetone-d_6)

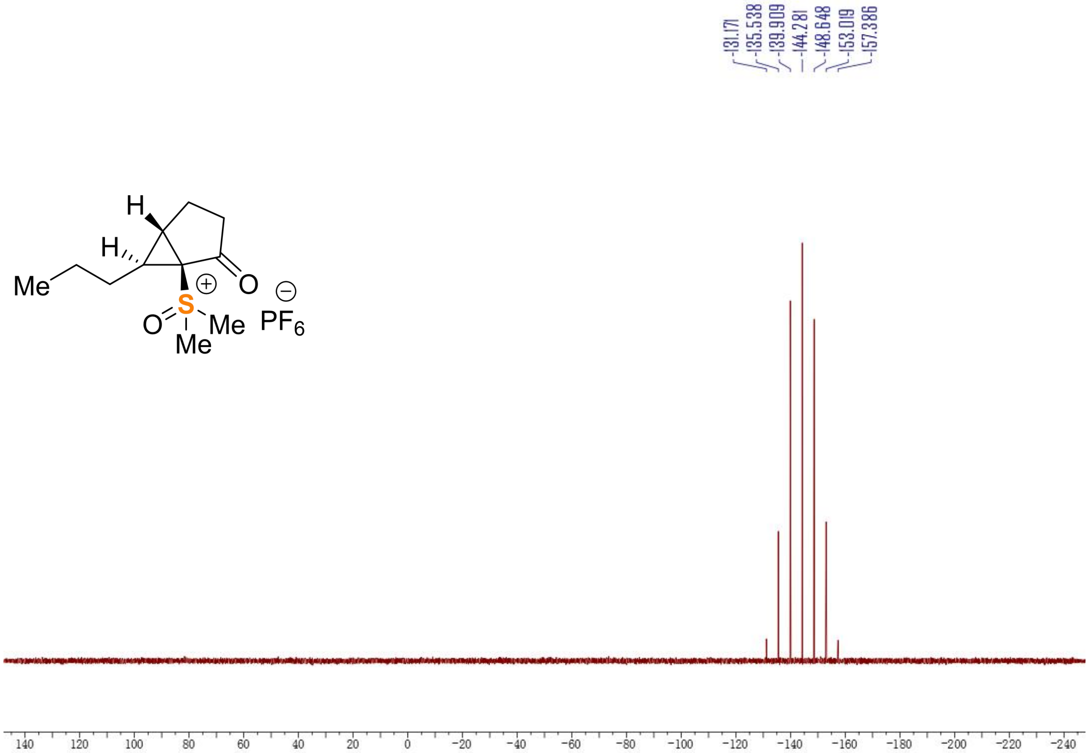

Supplementary Figure 278. ^{31}**P NMR** of the **14b** (162MHz, 25 °C in Acetone-d_6)

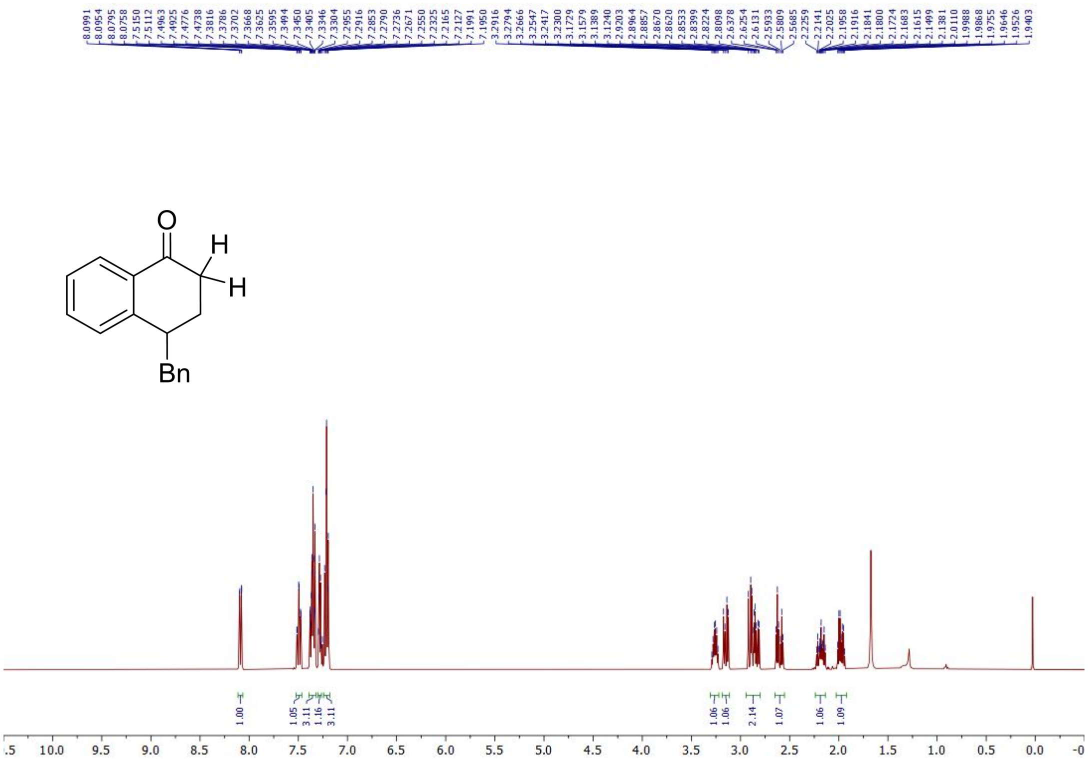

Supplementary Figure 279. ^{1}H NMR of the **15** (400 MHz, 25 °C in $CDCl_3$)

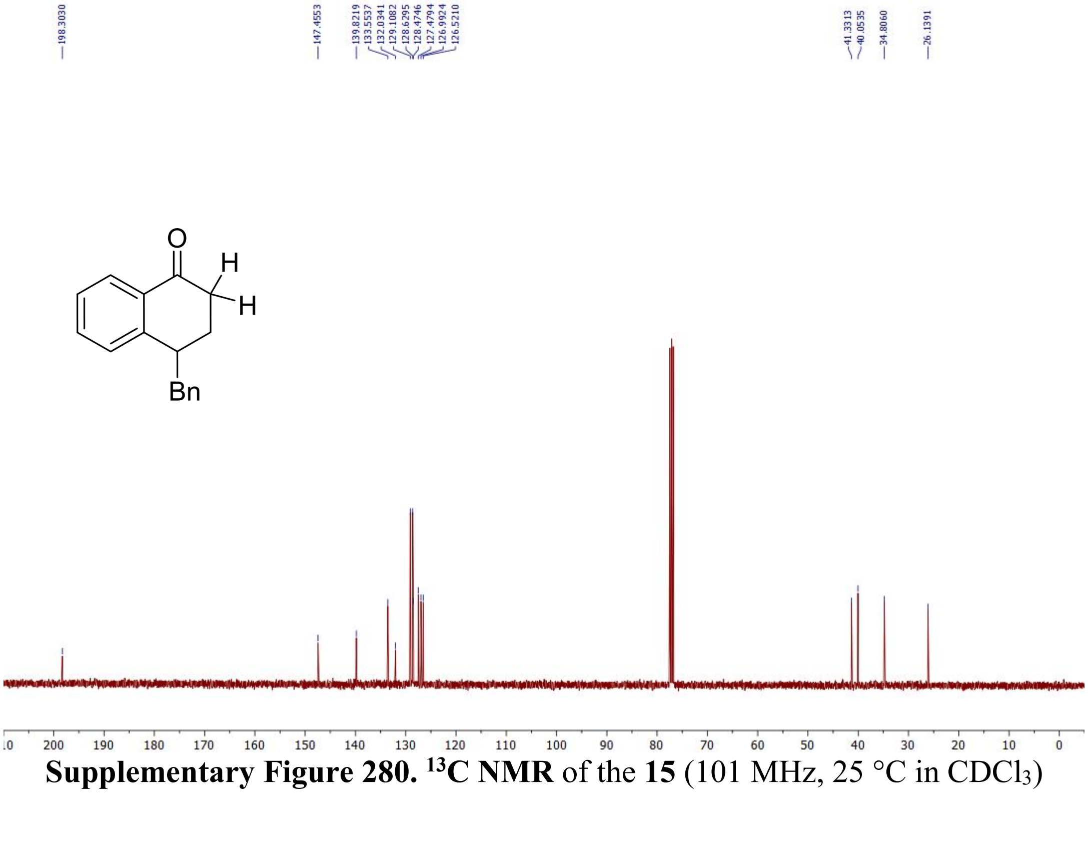

Supplementary Figure 280. ^{13}C NMR of the **15** (101 MHz, 25 °C in $CDCl_3$)

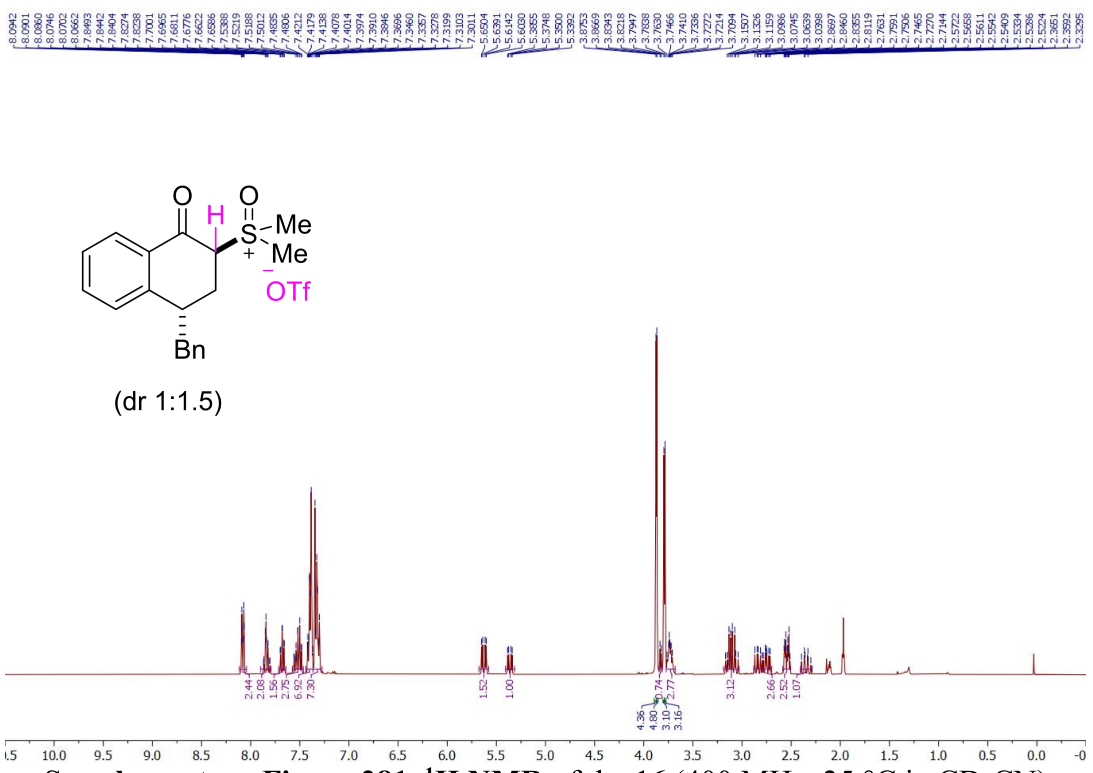

Supplementary Figure 281. ^{1}H NMR of the **16** (400 MHz, 25 °C in CD_3CN)

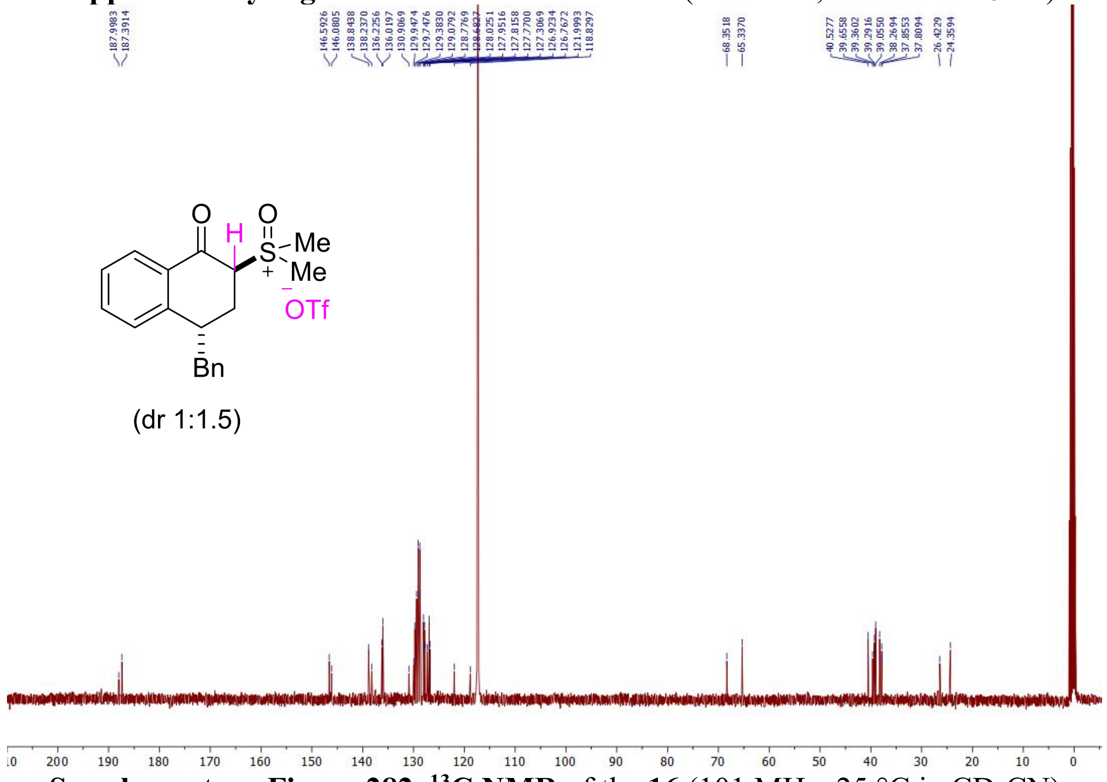

Supplementary Figure 282. ^{13}C NMR of the **16** (101 MHz, 25 °C in CD_3CN)

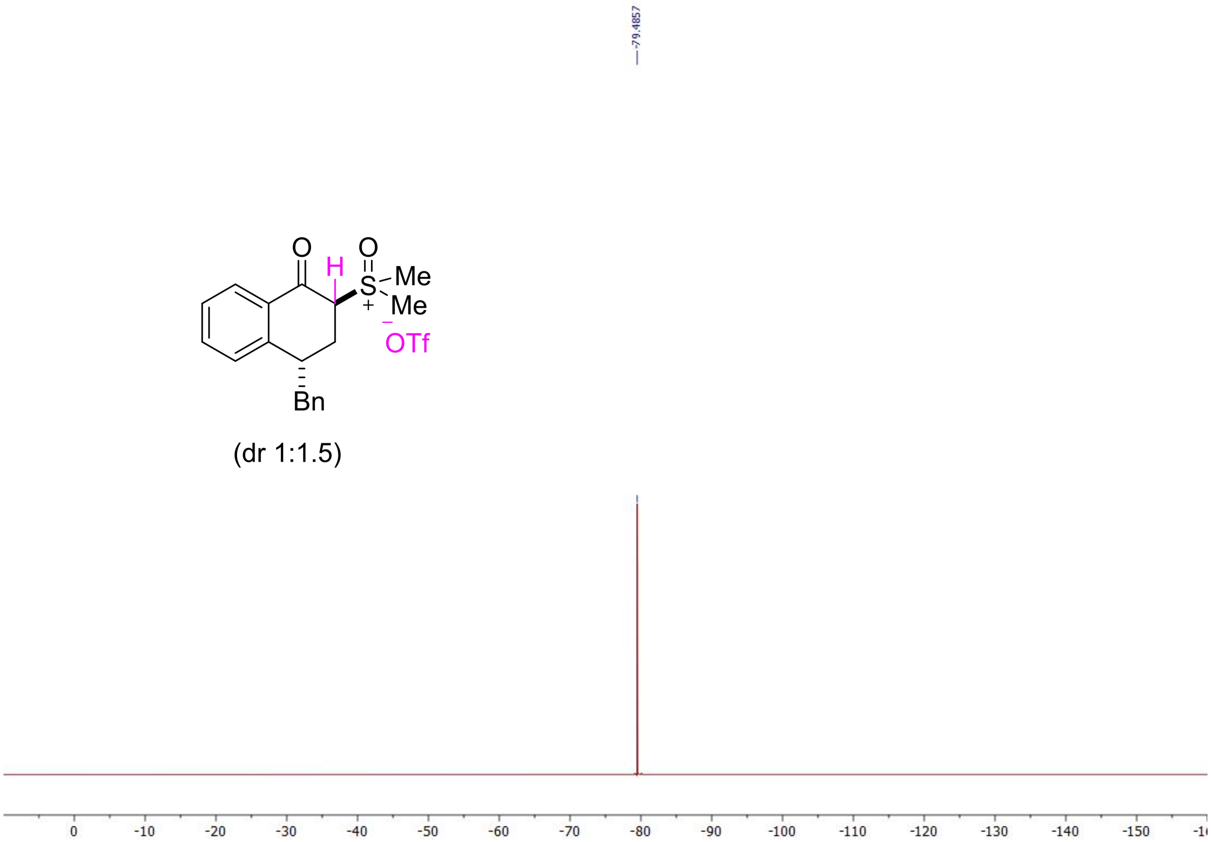

Supplementary Figure 283. ^{19}F NMR of the **16** (376 MHz, 25 °C in CD_3CN)

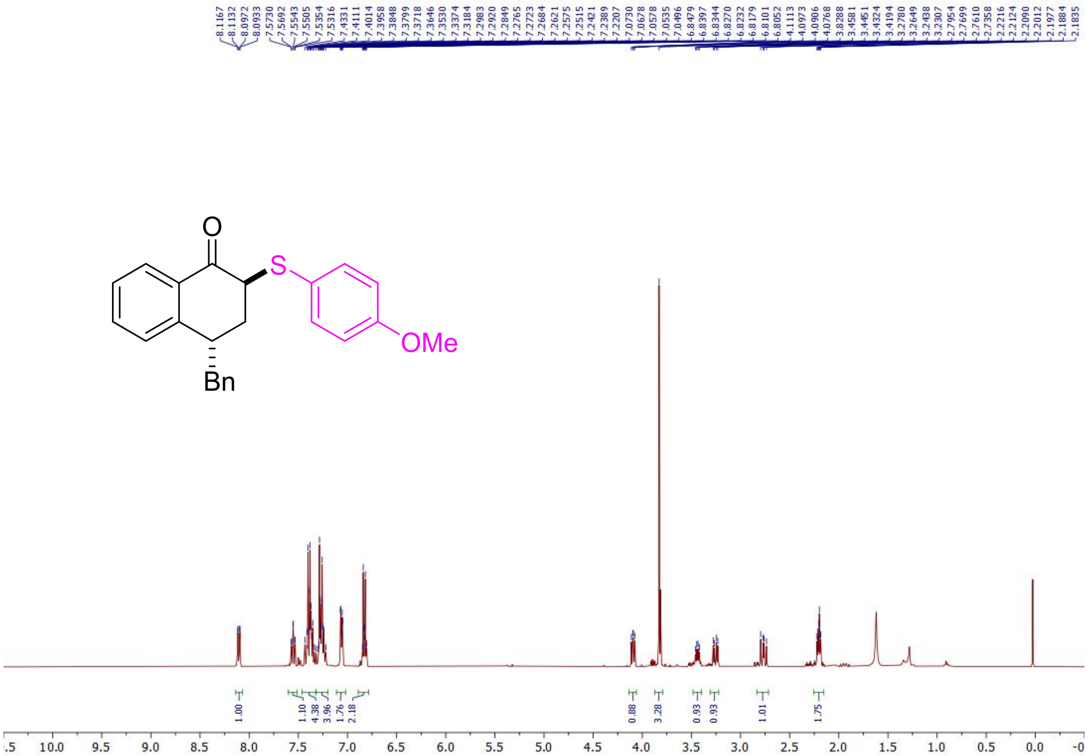

Supplementary Figure 284. ^{1}H NMR of the **17** (400 MHz, 25 °C in $CDCl_3$)

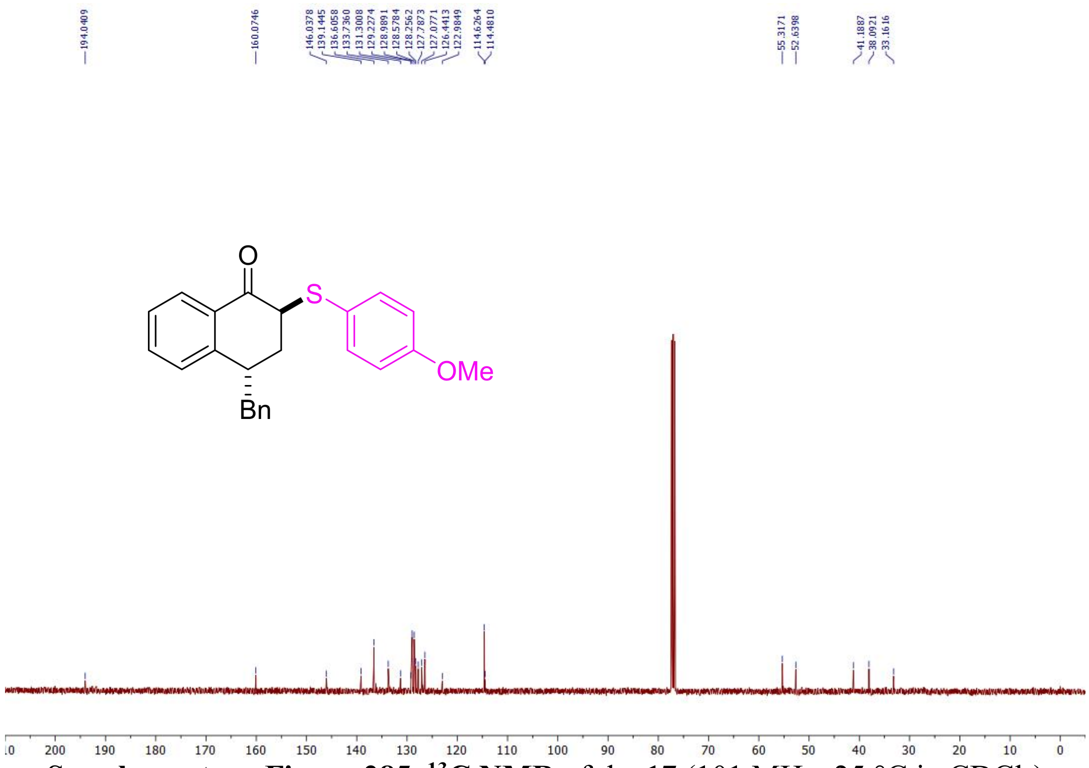

Supplementary Figure 285. ^{13}**C NMR** of the **17** (101 MHz, 25 °C in $CDCl_3$)

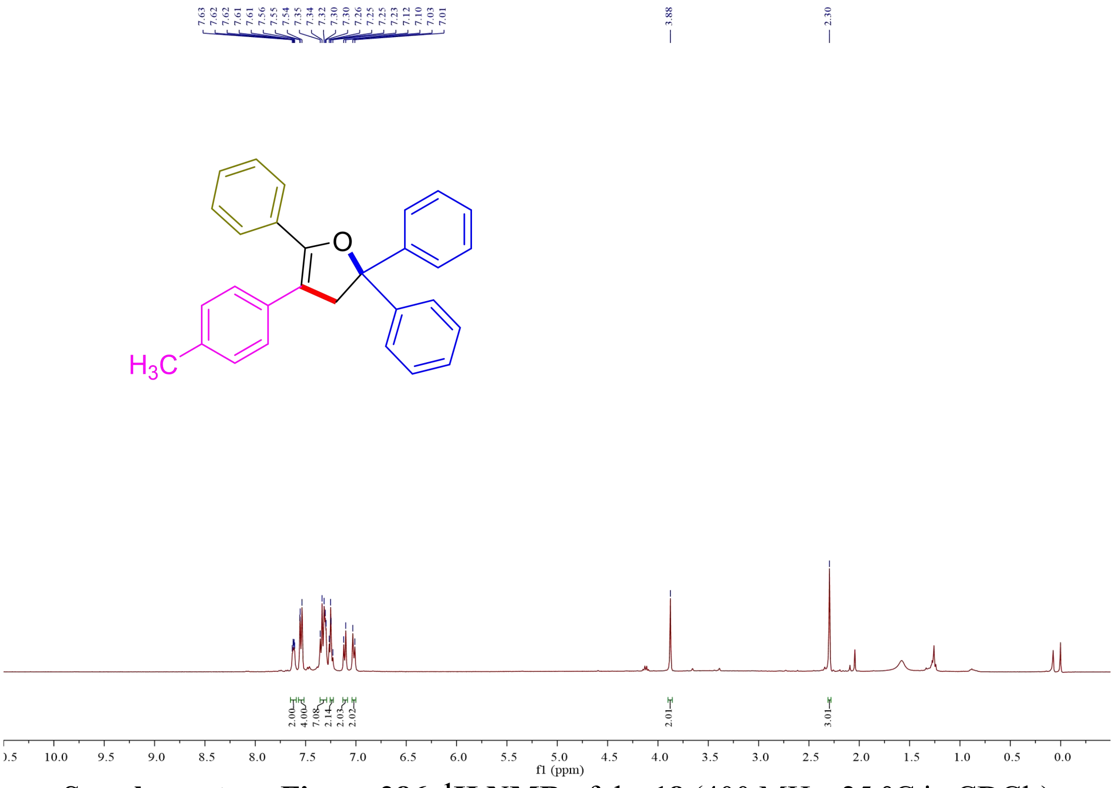

Supplementary Figure 286. ^{1}**H NMR** of the **18** (400 MHz, 25 °C in $CDCl_3$)

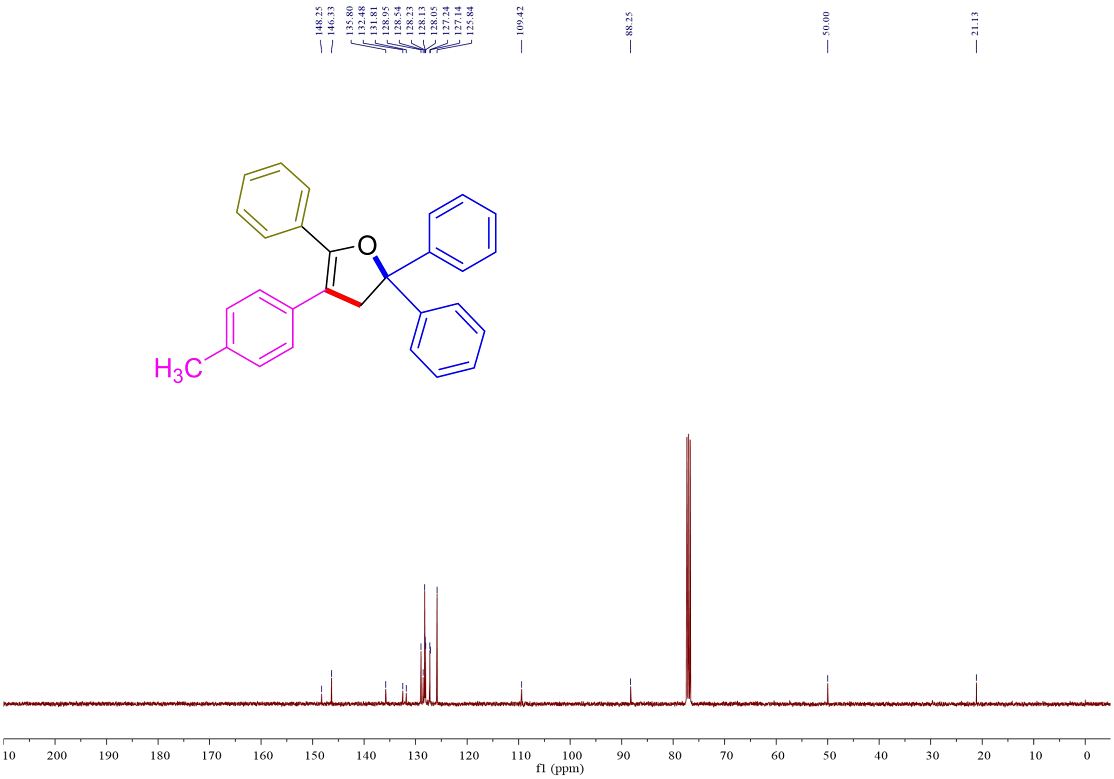

Supplementary Figure 287. ^{13}C NMR of the **18** (101 MHz, 25 °C in $CDCl_3$)

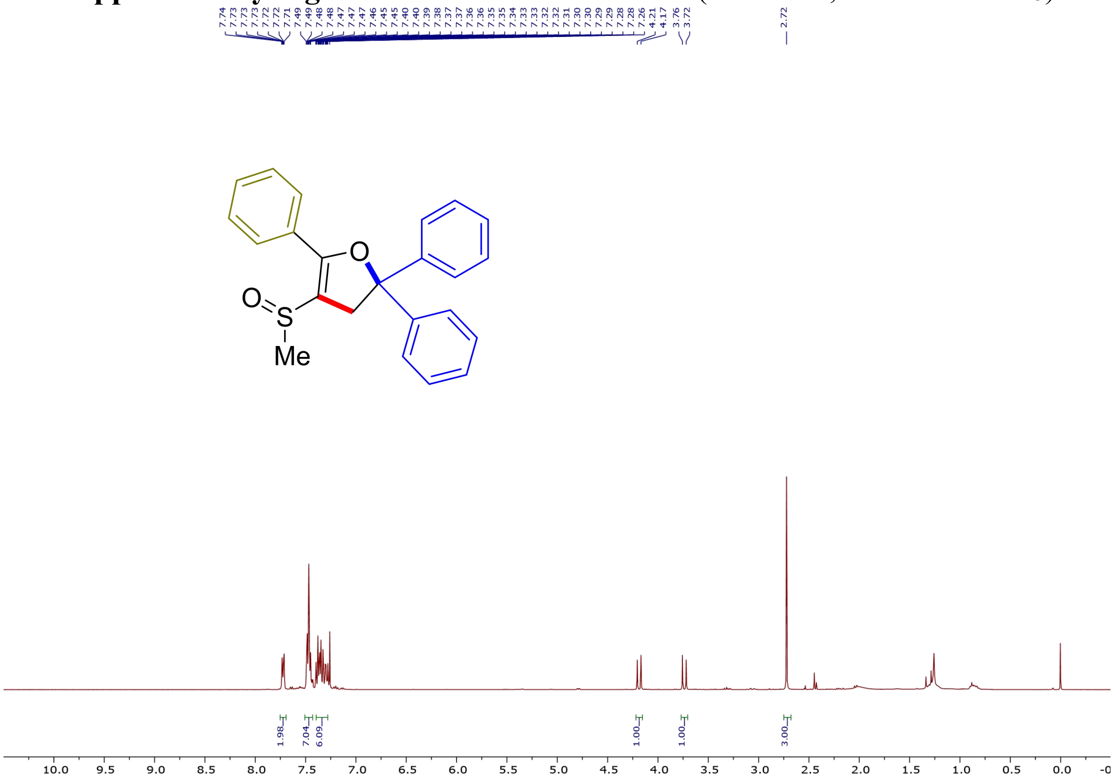

Supplementary Figure 288. ^{1}H NMR of the **19** (400 MHz, 25 °C in $CDCl_3$)

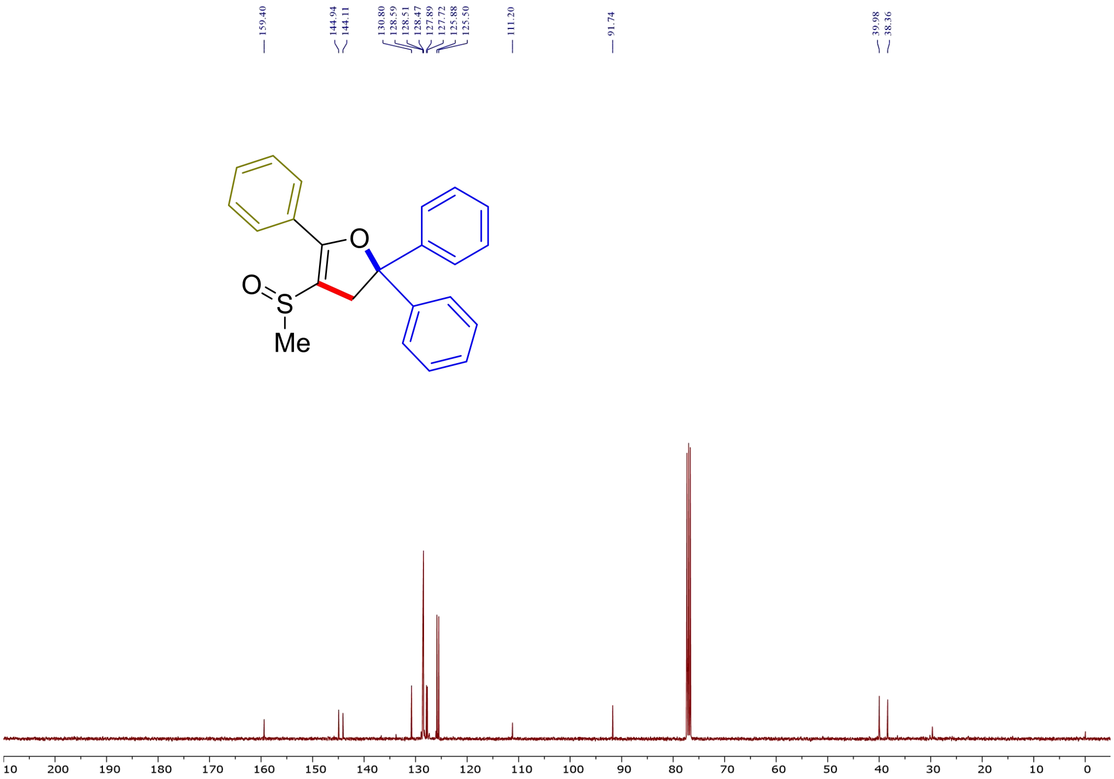

Supplementary Figure 289. ^{13}C NMR of the **19** (101 MHz, 25 °C in $CDCl_3$)

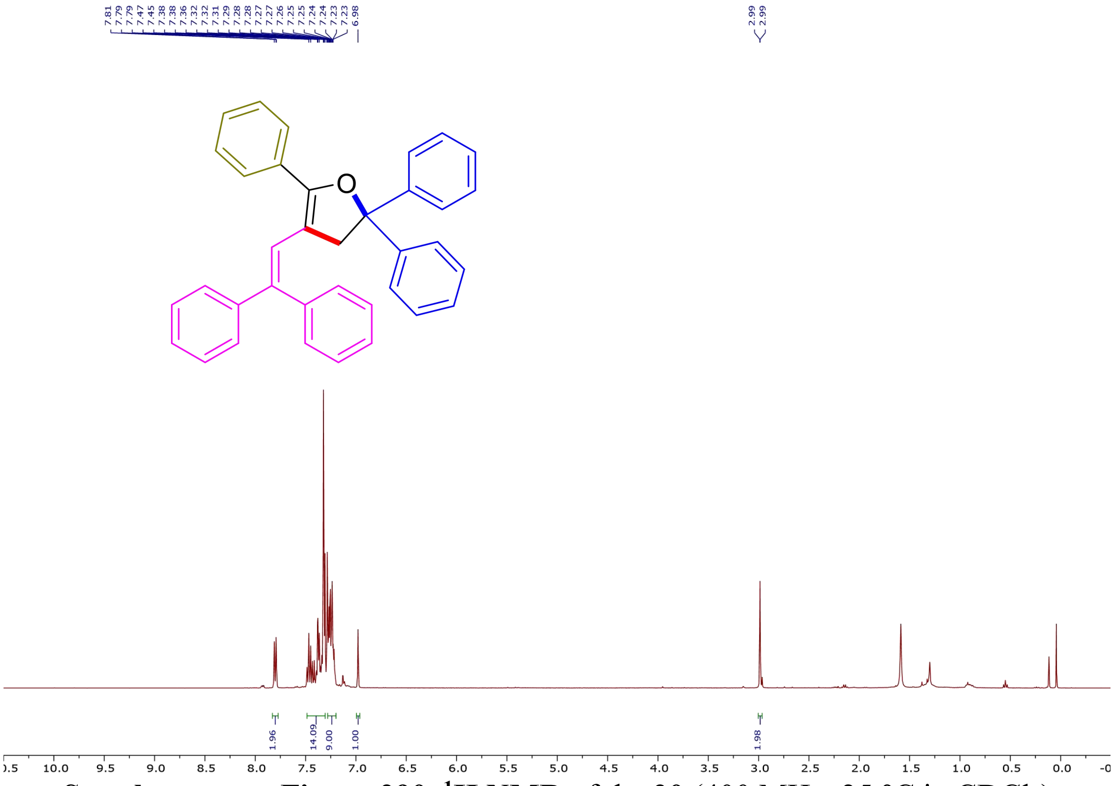

Supplementary Figure 290. ^{1}H NMR of the **20** (400 MHz, 25 °C in $CDCl_3$)

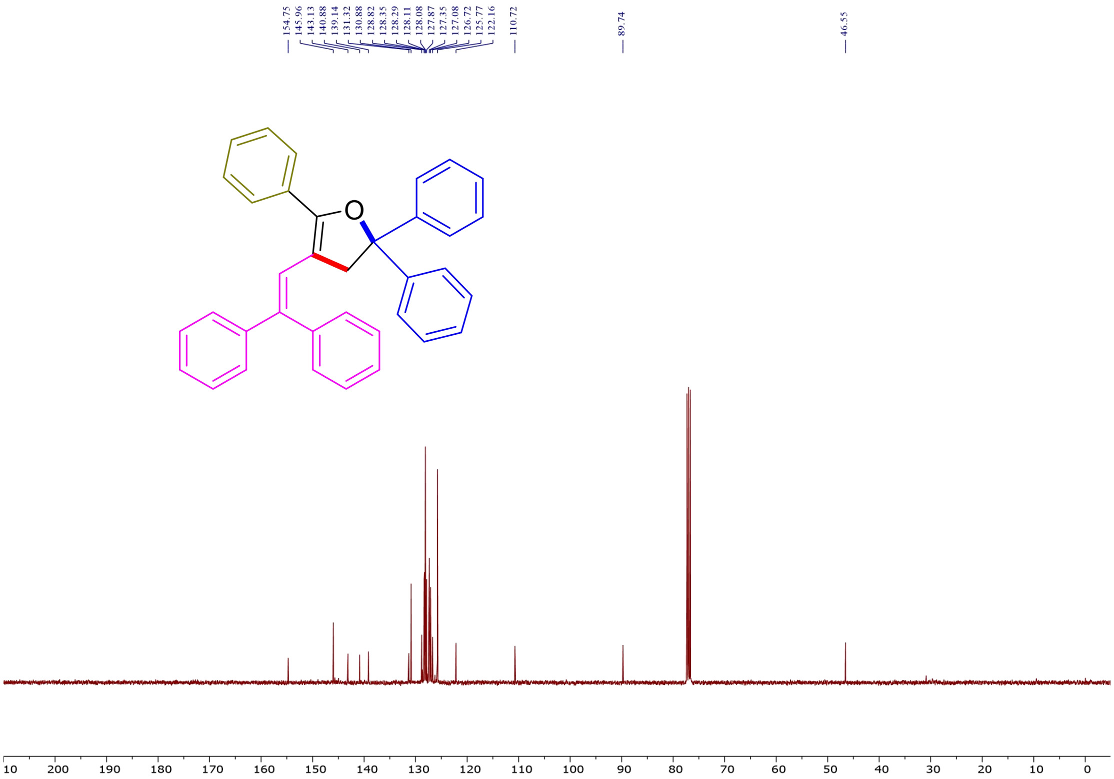

Supplementary Figure 291. ^{13}**C NMR** of the **20** (101 MHz, 25 °C in $CDCl_3$)

4. Supplementary References.

[1]. Clare, D. *et al.* Chemospecific cyclizations of α-carbonyl sulfoxonium ylides on aryls and heteroaryls. *Angew. Chem., Int. Ed.* **2019**, *58*, 16198−16202.

[2]. H. Yamanaka, S. *et al.* Studies on pyrimidine derivatives. XXIII. Synthesis of acylmethylpyrimidines and related compounds via imidoyl-substituted oxosulfonium ylides. *Chem. Pharm. Bull. (Tokyo).* **1981**, *29*, 2837–2843.

[3]. a) Tyllick, C. *et al.* Cycloalkylation studies. 1. A practical synthetic approach to the 2,3:6,7-dibenzobicyclo[3.2.2]nona-2,6-diene system. *J. Org. Chem.* **1991**, *56*, 2938–2940. b) Quiclet-Sire, B. *et. al.* Oxime Derivatives as α-Electrophiles. From α-Tetralone Oximes to Tetracyclic Frameworks. *Org. Lett.* **2011**, *13*, 3266–3269.

[4]. Lin, S. *et. al.* Aminoxyl-Catalyzed Electrochemical Diazidation of Alkenes Mediated by a Metastable Charge-Transfer Complex. *J. Am. Chem. Soc.* **2019**, *141*, 2825–2831.

[5] Gaussian 16, Revision A.03, M. J. Frisch, G. W. Trucks, H. B. Schlegel, G. E. Scuseria, M. A. Robb, J. R. Cheeseman, G. Scalmani, V. Barone, G. A. Petersson, H. Nakatsuji, X. Li, M. Caricato, A. V. Marenich, J. Bloino, B. G. Janesko, R. Gomperts, B. Mennucci, H. P. Hratchian, J. V. Ortiz, A. F. Izmaylov, J. L. Sonnenberg, D. Williams-Young, F. Ding, F. Lipparini, F. Egidi, J. Goings, B. Peng, A. Petrone, T. Henderson, D. Ranasinghe, V. G. Zakrzewski, J. Gao, N. Rega, G. Zheng, W. Liang, M. Hada, M. Ehara, K. Toyota, R. Fukuda, J. Hasegawa, M. Ishida, T. Nakajima, Y. Honda, O. Kitao, H. Nakai, T. Vreven, K. Throssell, J. A. Montgomery, Jr., J. E. Peralta, F. Ogliaro, M. J. Bearpark, J. J. Heyd, E. N. Brothers, K. N. Kudin, V. N. Staroverov, T. A. Keith, R. Kobayashi, J. Normand, K. Raghavachari, A. P. Rendell, J. C. Burant, S. S. Iyengar, J. Tomasi, M. Cossi, J. M. Millam, M. Klene, C. Adamo, R. Cammi, J. W. Ochterski, R. L. Martin, K. Morokuma, O. Farkas, J. B. Foresman, and D. J. Fox, Gaussian, Inc., Wallingford CT, **2016**.

[6] J. D. Chai, M. Head-Gordon, Long-range corrected hybrid density functionals with damped atom-atom dispersion corrections, *Phys. Chem. Chem. Phys.* **2008**, 10, 6615.

[7] T. Lu, F. Chen. Multiwfn: a multifunctional wavefunction analyzer. *J. Comput. Chem.* **2012**, *33*, 580.
